# Characterisation of visual guidance of steering to intercept targets following curving trajectories using Qualitative Inconsistency Detection

**DOI:** 10.1038/s41598-022-24625-4

**Published:** 2022-11-24

**Authors:** Albertha A. M. van Opstal, Remy Casanova, Frank T. J. M. Zaal, Reinoud J. Bootsma

**Affiliations:** 1 Institute of Movement Sciences, Aix Marseille University, CNRS, Marseille, France; 2 Department of Human Movement Sciences, University Medical Center Groningen, Groningen, The Netherlands

**Keywords:** Human behaviour, Sensorimotor processing

## Abstract

This study explored the informational variables guiding steering behaviour in a locomotor interception task with targets moving along circular trajectories. Using a new method of analysis focussing on the temporal co-evolution of steering behaviour and the potential information sources driving it, we set out to invalidate reliance on plausible informational candidates. Applied to individual trials rather than ensemble averages, this Qualitative Inconsistency Detection (QuID) method revealed that steering behaviour was not compatible with reliance on information grounded in any type of change in the agent-centred target-heading angle. First-order changes in the environment-centred target’s bearing angle could also not adequately account for the variations in behaviour observed under the different experimental conditions. Capturing the observed timing of unfolding steering behaviour ultimately required a combination of (velocity-based) first-order and (acceleration-based) second-order changes in bearing angle. While this result may point to reliance on fractional-order based changes in bearing angle, the overall importance of the present findings resides in the demonstration of the necessity to break away from the existing practice of trying to fit behaviour into a priori postulated functional strategies based on categorical differences between operative heuristic rules or control laws.

## Introduction

Locomotor interception can be observed in many different settings, ranging from a lion chasing a gazelle across a plain to a group of kids playing tag in a school yard or a football player running to intercept a pass on a sports field. Surprisingly however, notwithstanding a considerable body of work, the question of how we accomplish such tasks remains unanswered. Indeed, while it is now widely accepted that locomotor interception is ordinarily controlled online, that is, on the basis of currently available information^1–21^, the exact way information is used to regulate behaviour during locomotor interception is still subject to debate. In the present contribution we build on a recently developed method of analysis^3^ and demonstrate how it may contribute to empirically settling this debate. However, in order to proceed we first need to lay out the foundations of the debate.

Empirical research into the online control of locomotor interception of moving targets has resulted in the identification of a set of functional strategies^22–26^. Operational differences between such strategies are typically characterised by the pertinent angular agent-target relation considered, both in terms of the specific *type of angle* selected (agent-centred or environment-centred) and this angle’s specific *time-derivative order* relied upon to guide behaviour. The latter’s portrayal in the literature furthermore varies as a function of the level of description chosen, focussing either on the heuristics-based desired steady-state behaviour or on the dynamics-based underlying mechanism that may bring this desired behavioural state to come about.

**Figure 1:**
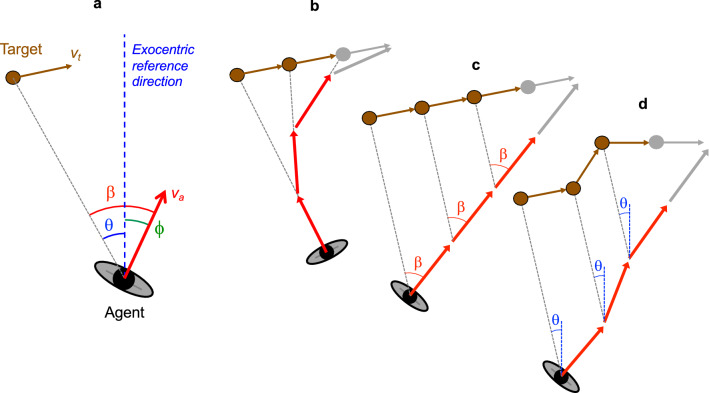
(**a**) Definition of variables in a plan view of an agent moving through an environment containing a target moving in the same plane. Instantaneous velocity vectors are represented by arrows (red for agent, brown for target). Agent heading ϕ and target bearing θ are defined with respect to an exocentric reference direction (dashed blue line). Target-heading angle β is defined by the eccentricity of the target with respect to the agent’s direction of locomotion so that β = ϕ − θ. (**b**, **c**, and **d**) Typical timestep-based representations of interception heuristics: (**b**) Zero Target-Heading Angle (ZTHA) or classical pursuit, (**c**) Constant Target-Heading Angle (CTHA) or classical interception, and (**d**) Constant Bearing Angle (CBA). Note that for an agent following a straight-line trajectory, as depicted in (**c**), not only THA β is constant but BA θ (not depicted) is also constant. The straight-line steady-state behaviour therefore does not allow disambiguating β-based and θ-based influences on behaviour. With all three examples being based on instantaneous enactment, at each time step, of a given heuristic rule, such steady-state representations mask the underlying dynamics characterised by transients towards such steady-state behaviour. In this light it is interesting to note that for the kind of target motion depicted in (**d**) a dθ/dt-nulling dynamics would generally not lead to the agent behaviour depicted, as the dynamics only drive θ towards constancy and not towards any particular value.

Let us illustrate this with some concrete examples, focussing on interception of targets moving in the agent’s plane of motion, as in the above-mentioned examples. The angles considered in this situation are the (agent-centred) target-heading angle β and the (environment-centred) target’s bearing angle θ (see Fig. 1a for definitions). At least three different strategies have been distinguished in this situation. In a classical pursuit strategy (heuristically referred to as ZTHA, for zero target-heading angle), the agent continuously seeks to move in the current direction of the target, that is, to maintain target-heading angle β at zero. With regard to underlying control, this behaviour has conventionally been instantiated by a steering dynamics based on nulling (i.e., minimising the magnitude) of β^27–30^. As β = d^0^β/dt^0^, such a strategy may thus be qualified as a β-based zeroth-order strategy. In a classical interception strategy (heuristically referred to as CTHA, for constant target-heading angle), on the other hand, the agent continuously seeks move so as to maintain target-heading angle β constant at some situation-specific non-zero value. In terms of dynamics, this behaviour has conventionally been instantiated by nulling the rate of change in β (i.e., nulling d^1^β/dt^1^)^1,2,6,7,15,23^ and may thus be qualified as a β-based first-order strategy. Often confounded with this classical interception strategy^1,2,6,7,15,31–36^ is the strategy (heuristically referred to as CBA, for constant bearing angle) of seeking to maintain the target’s bearing angle θ constant. This latter strategy has conventionally been instantiated by nulling the rate of change in θ (i.e., nulling d^1^θ/dt^1^)^3,23^ and may thus the qualified as a θ-based first-order strategy. We note that when the agent moves faster than the target all three of these strategies can result in successful interception. Graphical illustrations of ZTHA, CTHA and CBA heuristics typically rely on timestep-based representations to demonstrate the prototypical steady-state locomotor paths that they are associated with (see Fig. 1b–d for examples). It is, however, important to bear in mind that such illustrations fail to bring out important aspects of real interception behaviour, the most important here being their inadequacy to capture the transients towards such steady states that in fact constitute the signatures of the underlying dynamics. Indeed, while the steady-state behaviour associated with a given heuristic rule (cf. move so as to maintain some desired situation) is merely confirmatory, it is in the (transient) evolution of behaviour over time that the operative underlying control mechanism leading the system towards such a steady state may reveal itself.

Operationally, studies examining whether observed behaviour was based on a particular locomotor interception strategy have essentially relied on measures of global correspondence between observed and expected behavioural characteristics. In line with the foregoing, the expected behavioural characteristics typically include the constancy (steady-state behaviour) that is expected to emerge^31–38^, but occasionally have also been extended to include transient aspects, as captured by fitting of a dynamical model^1,2,6,7,15,23^. In practice, these analyses are generally performed on ensemble averages, estimated to best present the global behaviour under a given experimental condition.

The general position arising from the above cited studies has been that human locomotor interception relies on a first-order strategy, with some discussion remaining on whether it be β-based or θ-based^4,5,22,23,37,38^. Yet, several recent findings suggest that such a categorical classification of locomotor interception behaviour is not without problems. Focussing on individual trials rather than ensemble averages has started to reveal a far more subtle picture. For instance, interception characteristics related to early systematic influences of zeroth-order information were reported for (near) constant speed targets moving along straight trajectories^3–5^, while later systematic influences of second-order information were reported for targets moving along curved trajectories^3^. It thus appears that particular experimental conditions may give rise to richer interception behaviour than reasoning from categorical differences between interception strategies would lead one to expect. We therefore take another approach here. Rather than seeking to distinguish between predefined potential strategies, we take a step back and reconsider the empirical starting points for a useful analysis, following the methodological logic proposed by Bootsma et al.^3^ that we will refer to as Qualitative Inconsistency Detection (QuID).

The QuID method starts with acknowledging that online control models of locomotor interception seek to capture how an agent modifies its current state on the basis of currently available task-relevant information. Formally, this comes down to defining the elements of the general control law d*a*/dt = *f *(*I*, *a*) where *a* is the current state of the agent, *I* is the current state of the informational variable used in the control of the task and *f* is the function characterizing how current states of *a* and *I* determine the required change in *a*, that is d*a*/dt. Without necessitating hypotheses on the specifics of function *f* other than that informational variable *I* is being nulled, use of informational variable *I* can be empirically (in)validated by examining whether a change in the *direction of action variable a* observed at some time t is compatible with the *direction of drive provided by informational variable I* at t−Δt, where Δt is a visuomotor delay. For instance, each time that an agent is seen to change locomotor direction (e.g., from moving leftward to moving rightward or vice versa), the potential driving role of any plausible informational variable can be evaluated by examining its direction of drive a Δt moment earlier. Providing a dichotomic answer to the question whether at a particular moment in time a particular informational variable could have driven the agent in the correct direction, the evaluation method in fact hunts for qualitative inconsistencies. Detection of a qualitative inconsistency allows ruling out the possibility that the particular informational variable considered may have guided the ongoing control process, at least at that point in time. Such behavioural event-anchored analysis may furthermore be usefully complemented by an examination of the direction and magnitude of drive provided by a particular informational variable over a period of time following a behavioural event. Indeed, if an action gives rise to a substantial change in the direction of drive provided by a given informational variable without provoking a fitting adaptive action, this also constitutes a qualitative inconsistency for this informational variable. In short, the QuID method evaluates reliance on plausible informational variables to drive behaviour by examining, for each informational variable, whether it systematically fulfils two essential requirements: an observable change in direction of the action variable should be proceeded by an appropriately oriented information-based drive some time before, and any substantial incongruously oriented information-based drive should result in an observable change in direction of action variable *a*. It is important to note that, under the assumption that the same control law applies over a wide range of conditions, such qualitative inconsistencies may surface only under specific conditions^3–5,23^.

**Figure 2:**
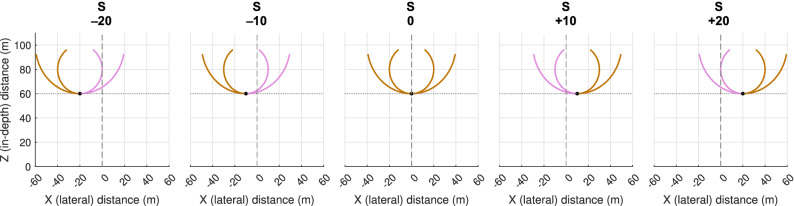
Initial conditions and target trajectories. The participant’s position at the onset of an experimental trial corresponded to the X–Z coordinate system’s (0,0) origin. At that moment in time, a target (represented by a black dot) appeared at a lateral position S of − 20, − 10, 0, + 10 or + 20 m, at a constant in-depth distance of 60 m. From each initial target position, targets could move along two different circular trajectories (20-m and 40-m radii), either leftward or rightward. The resulting 20 experimental target trajectories were subsequently mirror-collapsed, recoding target motion direction to inward or outward. On inward trajectories (pink) the target initially moved toward the X = 0 axis corresponding to the participant’s initial movement direction, while on outward trajectories (brown) the target initially moved away from this axis. Note that for S = 0 all target trajectories were (by definition) outward.

In the present contribution we therefore applied the QuID method to assess the potential role of the zeroth-order, first-order and second-order time derivatives of target-heading angle β and target’s bearing angle θ over the course of each individual interception action in a simulated interception-by-steering task^4,5^ with targets moving at constant speed along curved (here circular) trajectories. We hypothesised that control of steering would be θ-based rather than β-based^4,5,23^ and that curving target trajectories would elicit a shift from initial reliance on first-order (d^1^θ/dt^1^) information to a combination of first- and second-order (d^2^θ/dt^2^) information after around 1 s^3^. As graphically illustrated in Fig. 2, participants moving at 20 m/s were to drive through cylindrical targets that could appear at five different initial lateral positions in the environment. Targets moved at 10 m/s initially either leftward or rightward following circular trajectories of 20-m or 40-m radius. For global analysis purposes, for each of the two target trajectory radii (coded R40 and R20), the experimental conditions were mirror-collapsed for initial positions (coded S20, S10, and S0) with target movement direction recoded to inward (IN) and outward (OUT), giving rise to 10 collapsed conditions (only OUT motion for S0).

## Results

Participants performed well on the task, as attested to by the overall 96.6% success rate. The time from trial onset until the moment of contact with the target (i.e., action duration) varied over target trajectory conditions (means between 3.86 to 5.80 s). Within each target trajectory condition, however, action duration was quite stable over participants and trials (SDs ≤ 0.10 s; see Table 1 for details).

**Table 1:** Participants overall Success rate and Action duration (*M* ± *SD*) under the (mirror-collapsed) experimental target trajectory conditions.

Trajectory code	Radius	Departure position	Direction	Success rate (%)	Action duration (s)
S20/R20-IN	20 m	± 20 m	Inward	97.0	4.73 ± 0.08
S20/R40-IN	40 m	± 20 m	Inward	100.0	3.86 ± 0.04
S10/R20-IN	20 m	± 10 m	Inward	96.4	4.70 ± 0.07
S10/R40-IN	40 m	± 10 m	Inward	99.4	4.17 ± 0.05
S0/R20-OUT	20 m	0 m	Outward	94.0	4.77 ± 0.07
S0/R40-OUT	40 m	0 m	Outward	99.4	4.66 ± 0.09
S10/R20-OUT	20 m	± 10 m	Outward	91.1	4.94 ± 0.09
S10/R40-OUT	40 m	± 10 m	Outward	100.0	5.22 ± 0.10
S20/R20-OUT	20 m	± 20 m	Outward	90.5	5.16 ± 0.10
S20/R40-OUT	40 m	± 20 m	Outward	98.2	5.80 ± 0.07

### Exemplary trials

**Figure 3:**
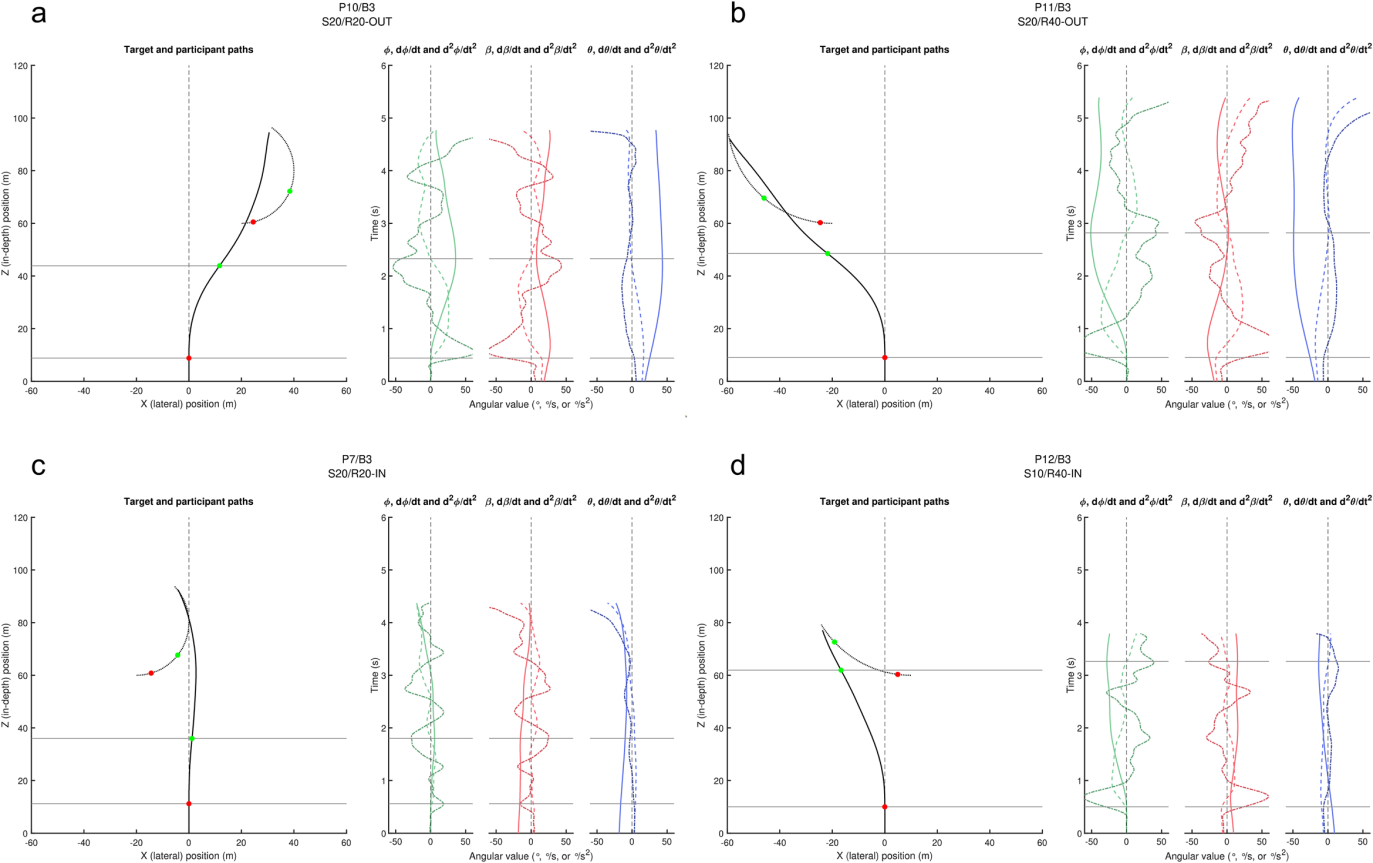
QuID-plots for four exemplary trials: (**a**) P10, Block 3 S20/R20-OUT (rightward), (**b**) P11, Block 3 S20/R40-OUT (leftward), (**c**) P7, Block 3 S20/R40-IN (rightward), (**d**) P12, Block 3 S10/R40-IN (leftward). Left graph in each panel: spatial paths followed by the target (dotted grey line) and the participant (black line). Steering events are marked by colour-coded dots. Right graphs in each panel: time evolution (bottom to top) over the course of the trial of the participant’s heading direction ϕ (in green), the target-heading angle β (in red) and the target’s bearing angle θ (in blue) together with their first-order (dashed) and second-order (dash-dotted) time derivatives. Horizontal grey lines situate the steering events spatially (left graph) and temporally (right graphs). For each panel an enlarged figure version is available as Supplementary Fig. S1 online.

We begin our analysis of information sources potentially driving steering behaviour by examining four exemplary trials (Fig. 3; the full set of 1680 trials is available online as Supplementary Information), focussing on the temporal co-evolution of steering behaviour and potential information sources driving it. For each trial (i.e., each panel in Fig. 3), the spatial paths followed by the target and the participant are presented in the left graph. Target and participant positions at the onset of the first and second steering actions are marked by, respectively, red and green dots. Spatially situating the participant turns in the left graph by horizontal grey lines, these turns are temporally situated by the corresponding horizontal lines in right graphs, presenting the time evolution (bottom to top) of the participant’s heading direction ϕ (in green), the target-heading angle β (in red) and the target’s bearing angle θ (in blue) together with their first-order and second-order time derivatives.

We first focus on the potential driving role of β-based information, starting from dβ/dt information. Figure 3a presents an exemplary S20/R20-OUT trial. With the target initially moving in an outward (here rightward) direction, from the onset of the trial onwards angle β opens and a positive dβ/dt signals a rightward drive. As the first steering action (lower horizontal line) is indeed rightward, this observed behaviour is therefore compatible with it being driven by dβ/dt. This first steering action however rapidly leads angle β to close and thereby dβ/dt to switch sign. The persistent resulting (now leftward) drive signalled by sign-switched dβ/dt does not lead to any corrective leftward steering action for well over 1 s, which is not compatible with dβ/dt-driven steering. Analogous behaviour is observed in another exemplary trial (Fig. 3b, representing a S20/R40-OUT, here leftward, trial): Initially signalling a leftward drive, the (indeed) leftward first steering action again leads dβ/dt to rapidly switch sign. The persistent resulting (now rightward) drive signalled by sign-switched dβ/dt does not lead to any corrective rightward steering action for well over 2 s. The phenomenon can also be observed in Fig. 3d, representing a S10/R40-IN trial. In fact, we detected dβ/dt sign-switching within 0.5 s after onset of the first steering action in 51.9% of all trials, with its presence being particularly prominent under conditions with outward target motion. In 94.1% of all cases the corrective drive signalled by sign-switched dβ/dt information did not give rise to a steering action within at least a 1-s period. Overall, this qualitatively inconsistent drive pattern disqualifies dβ/dt as the informational variable driving the system. In the light of what follows, it is important to note that neither β nor d^2^β/dt^2^ information can replace or supplement dβ/dt information. Indeed, prior to the first steering action, β information signals a drive in the direction opposite to that of the upcoming steering action in almost all trials with inward target trajectories (e.g., Fig. 3c,d). In almost all trials with outward target trajectories, following the first steering action β information signalled a drive in the direction opposite to that of the following steering action. The same picture emerges for d^2^β/dt^2^ information that following the first steering action generally tended to signal a direction of drive opposite to that subsequently produced, as is observable in all four examples presented in Fig. 3.

Contrary to the β-based signals that revealed strong covariation with the ϕ-based signals (particularly obvious for the second derivatives), θ-based signals evolved on a considerably slower timescale. As can be seen in the exemplary trial of Fig. 3a, from the onset of the trial onward dθ/dt signals a (rightward) direction of drive that is compatible with the observed (rightward) direction of the first steering action; the steering action subsequently leads dθ/dt to evolve gradually, with a sign-switch occurring shortly before the onset of the second steering action. Steering behaviour in this trial can thus be understood as being driven by dθ/dt information. While the same phenomena are observed for the exemplary trials of Fig. 3b,d, this is not the case for the exemplary trial of Fig. 3c. Here, the dθ/dt sign-switching occurs after onset of the second steering action, providing evidence against reliance on dθ/dt information at that time. The only informational variable actually capable of providing the leftward drive required to explain this trial’s steering behaviour at that time is d^2^θ/dt^2^. Although d^2^θ/dt^2^ also provided a drive compatible with the observed steering behaviour for the first steering action, we may not conclude that d^2^θ/dt^2^ did in fact adequately account for steering behaviour over the whole trial, as it reveals the same rapid sign-switching phenomenon as observed for dβ/dt, signalling a drive in the opposite direction already long before the observed second steering event. In the end, and in line with Bootsma et al.’s^3^ conclusions, the overall picture emerging from inspection of the exemplary trials was that participants early on relied on dθ/dt information and later on relied on a combination of dθ/dt information and d^2^θ/dt^2^ information. In order to examine the generality of these θ-based results, we turn to the event-anchored (i.e., time-locked) analysis of the full dataset.

### Steering events

In the curated (21 trials removed) full 1659-trial dataset, we identified 3115 salient steering actions (corresponding to those marked in the exemplary trials; see Methods section for criteria). This amounts to an average of 1.88 steering actions per trial. To pinpoint the timing of onset of the steering actions, we binned these events using 0.5-s durations; the first time bin thus contained the steering events observed from 0 s (trial onset) to 0.5 s, the second from 0.5 s to 1.0 s, etc. As can be seen from Table 2, half (50.8%) of the total number of steering events were observed within the first second (i.e., first two 0.5-s bins) after onset of a trial, followed by a 1-s period (3rd and 4th bin) with little activity. A second wave of steering events was observed during the period from 2 to 4 s (i.e., 5th to 8th 0.5-s bins), after which their number declined to zero.

**Table 2:** Number of steering events observed over all 1659 retained trials for each of the 10 different target trajectory conditions per time bin of 0.5 s duration from the onset of a trial onward.

Time bin	1	2	3	4	5	6	7	8	9	10	11	12	
Bin upper limit	0.5 s	1.0 s	1.5 s	2.0 s	2.5 s	3.0 s	3.5 s	4.0 s	4.5 s	5.0 s	5.5 s	6.0 s	
Trajectory code													Total
S20/R20-IN	29	104	12	29	118	15	5	4	4	4	0	0	324
S20/R40-IN	20	120	23	5	3	12	51	4	0	0	0	0	238
S10/R20-IN	73	80	1	28	99	27	2	0	6	0	0	0	316
S10/R40-IN	77	89	1	1	1	18	42	33	0	0	0	0	262
S0/R20-OUT	101	61	2	20	67	68	9	4	1	0	0	0	333
S0/R40-OUT	108	54	1	0	1	13	23	70	37	0	0	0	307
S10/R20-OUT	126	40	0	8	58	72	26	4	0	0	0	0	334
S10/R40-OUT	118	49	0	0	0	17	41	59	37	12	0	0	333
S20/R20-OUT	107	59	1	6	55	67	36	2	1	1	0	0	335
S20/R40-OUT	121	45	0	0	5	22	35	56	32	12	5	0	333
Total	880	701	41	97	407	331	270	236	118	29	5	0	3115

For each steering event we determined, for each informational variable (here θ, dθ/dt, and d^2^θ/dt^2^) separately, whether, at a 100-ms visuomotor delay^3^ before its moment of occurrence, the upcoming direction of steering (i.e., leftward or rightward) corresponded to the direction of steering signalled by the informational variable. Concretely, we determined whether 100 ms before onset of the identified steering action potential informational variables θ, dθ/dt, and d^2^θ/dt^2^ correctly signalled this upcoming event. Thus, upcoming negative values of dϕ/dt (leftward steering) were considered to be correctly signalled by a negative value of the informational variable examined and upcoming positive values of dϕ/dt (rightward steering) were considered to be correctly signalled by a positive value of the informational variable examined. Applying this procedure to all steering events allowed evaluation of the overall capacity of each individual informational variable to correctly drive the full set of steering events identified.

**Figure 4:**
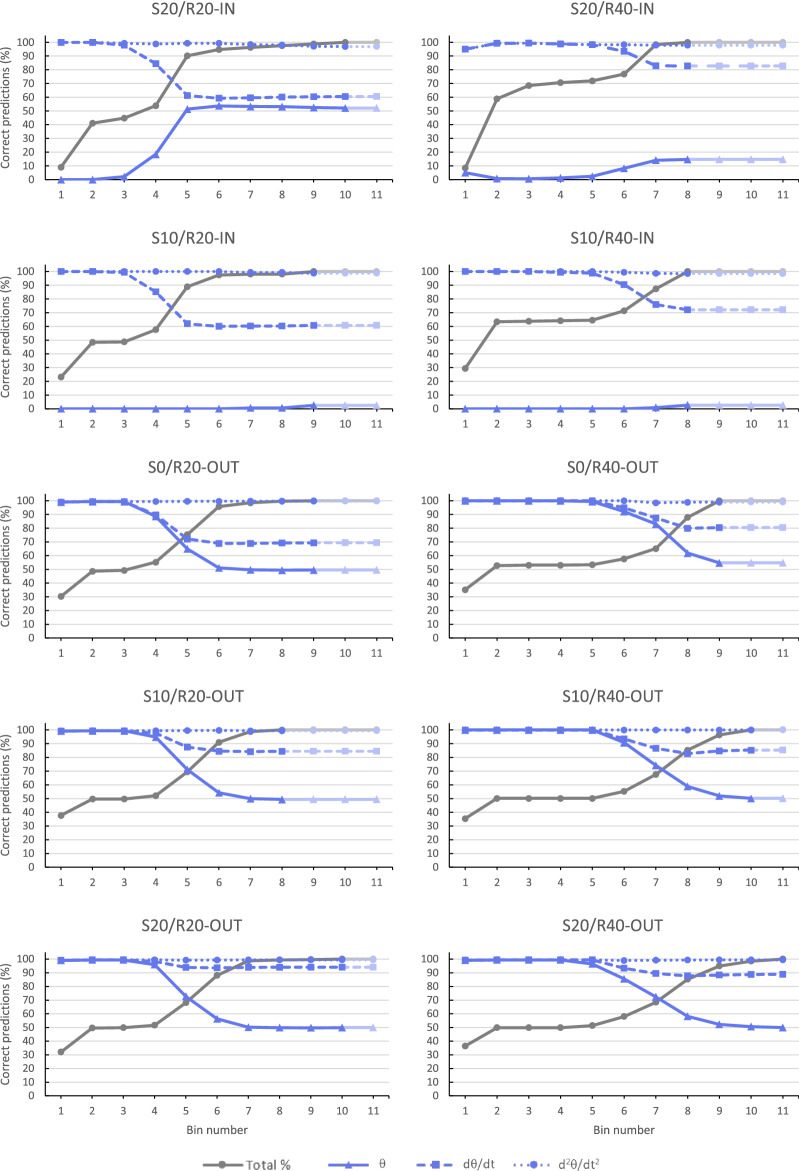
Cumulative percentage correct predictions over 0.5 s time bins of upcoming steering direction signalled a visuomotor delay Δt = 0.1 s earlier by θ (full blue line), dθ/dt (dashed blue line), or d^2^θ/dt^2^ (dotted blue line) for each of the 10 experimental target trajectory conditions (see Table 1 for trajectory codes). The dark grey line presents the cumulative percentage over (binned) time of the total number of observed steering events. Attenuated colours mark (empty) time bins extending beyond the condition’s observed action duration. Qualitatively similar results were obtained for Δt = 0.2 s.

Figure 4 presents, for each target trajectory condition separately, the cumulative percentage over binned time of steering events that could be correctly explained by unique reliance on θ, dθ/dt, or d^2^θ/dt^2^. Using cumulative (rather than absolute) percentages allowed avoiding the distortion effects of comparisons between bins with many steering events and bins with few steering events. The overall results clearly ruled out unique reliance on θ, as it systematically signalled steering in the wrong (i.e., contrary to observed) direction early on for all inward-moving target trajectories. Cumulated over all time bins, it never exceeded 60% of correct predictions for any target condition. Unique reliance on dθ/dt, on the other hand, correctly predicted the observed direction of steering early on, with cumulative percentage correct predictions seen to begin declining from (close to) 100% after 1.5 to 2.0 s for the R20 target trajectories and after 3.0 s for the R40 target trajectories. Taking into account the plateauing of the number of steering events between 1 and 2 s for the R20 target trajectories and between 1 and 2.5 s for the R40 conditions, we must conclude that our data only support potential unique reliance on dθ/dt over the first second of the interceptive actions. Indeed, for steering events occurring after 1 s into the trial, only 55.6% were correctly accounted for by reliance on dθ/dt (35.2% for R20 trials, 73.6% for R40 trials). Finally, unique reliance on d^2^θ/dt^2^ correctly predicted upcoming direction of steering for all target trajectories over the full lengths of the times series.

## Discussion

In this contribution we explored the informational variables relied upon to control steering in a locomotor interception task with targets following curved trajectories. Contrary to current practice, we did not seek to confirm the operation of any of the a priori postulated strategies based on categorical differences between operative heuristic rules or control laws. Using a new method of analysis focussing on the temporal co-evolution of steering behaviour and the potential information sources driving it, we rather set out to invalidate reliance on plausible informational candidates. Applied to individual trials rather than ensemble averages, this Qualitative Inconsistency Detection (QuID) method allowed ruling out reliance on target-heading angle β-based informational candidates and pointed to reliance on a (perhaps time-evolving) combination of first- and second-order time derivatives of the target’s bearing angle (i.e., d^1^θ/dt^1^ and d^2^θ/dt^2^). In the following we discuss each of these points in more detail.

In line with earlier studies on human interception-by-steering^4,5,20,22,37–39^, a β-based zeroth-order (ZTHA) strategy of target pursuit was clearly not observed. Our results also allowed ruling out the β-based first-order (CTHA) strategy of reliance on dβ/dt information. Indeed, especially in the initially outward-moving target motion conditions, steering actions were often accompanied by sign switches in dβ/dt (see Fig. 3 for examples), with dβ/dt values subsequently remaining in the switched state for long periods at large magnitudes. The finding that such prolonged periods of a strong dβ/dt-induced drive to change direction did not lead to compensatory steering behaviour rules out dβ/dt-nulling as a feasible option for the control of steering under the present circular target motion conditions. We note that under uniform rectilinear target motion conditions dβ/dt-nulling has also been ruled out as a feasible option for the control of steering, as it cannot explain the sign-switch observed during the initial steering action for targets moving outward from an initial position straight-ahead of the participant^4,5,23^. Under such conditions the outward-moving target immediately creates an opening of the β angle, with the agent thus lagging the target. Yet, participants subsequently do not steer so as to adequately null this opening (i.e., null dβ/dt); they in fact systematically steer ahead of (i.e., lead) the target, giving rise to a sign-switch in both dβ/dt and β. Acknowledging this problem, it has been suggested^37^ that a dβ/dt-nulling strategy could still be maintained if one introduced the additional constraint of always keeping target-heading angle β at a lead value. Interestingly, the present results (e.g., see Fig. 3a) reveal that, under the specific conditions of circular target motion, participants do not systematically steer ahead of (i.e., lead) the target, as β mostly continues to lag the target, thereby invalidating the generalisability of such an enhanced dβ/dt-nulling strategy.

Taking a step back, we noted that the QuID plots of Fig. 3 revealed strong (negative) covariation between ϕ-based signals and β-based signals (cross correlation of second-order ϕ and β signals: median *r* = − 0.943, IQR = 0.067), while θ-based signals evolved at a much slower timescale (cross correlation of second-order ϕ and θ signals: median *r* =  + 0.711, IQR = 0.146). This is due to the fact that target-heading angle β is directly affected by changes in heading direction ϕ, while the target’s bearing angle θ is not. Indeed, when freezing out the effects of target motion, it is clear from the graphical definitions depicted in Fig. 1a that β is affected by both agent rotation and agent translation while θ is affected only by agent translation. Yet, the apparently parsimonious θ-based first-order (CBA) strategy of driving interception behaviour by nulling dθ/dt^17,23^ did not adequately capture the present results either: d^2^θ/dt^2^ information was required to complement dθ/dt information later on, after around 1 s into a trial.

**Figure 5:**
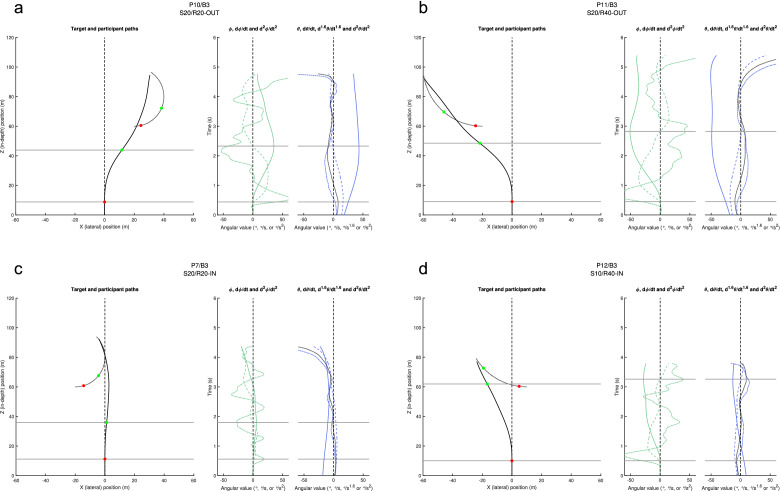
Modified QuID-plots for the four exemplary trials: (**a**) P10, Block 3 S20/R20-OUT (rightward), (**b**) P11, Block 3 S20/R40-OUT (leftward), (**c**) P7, Block 3 S20/R40-IN (rightward), (**d**) P12, Block 3 S10/R40-IN (leftward). Compared with Fig. 3, the time-evolutions of target-heading angle β related signals have been removed and a 1.6th-order time derivative of the target’s bearing angle θ has been added (dotted black) to the θ related signals.

The present finding in a velocity-constrained interception-by-steering task of behavioural effects implicating reliance on both dθ/dt and d^2^θ/dt^2^ information when targets follow curved trajectories replicates and extends our earlier results obtained in a direction-constrained lateral locomotor interception task^3^. As in both cases explanation of the observed interception behaviour implicates the operation of some kind of combination of dθ/dt and d^2^θ/dt^2^ information, one may wonder why, if indeed accessible, interception of curving trajectories would not simply fully rely on d^2^θ/dt^2^ information. For the interception of fly balls following trajectories curving in the sagittal plane, a second-order informational strategy of nulling acceleration of the optical elevation angle ε (i.e., nulling d^2^ε/dt^2^) has indeed been proposed^9,11,12,14^. Interestingly, however, in the light of the human visual system’s low sensitivity to optical acceleration^40–43^ reliance on detection of some intermediate form of change in optical velocity has been argued to be possible and sufficient^9^. Such intermediate forms of change could be parsimoniously captured through opening up the space between classical integer-order derivatives by considering fractional-order derivatives^3,44–48^. In this perspective, interception could be controlled by nulling an informational variable dθ^α^/dt^α^ where derivative order α can be any real value, in the present case situated between 1 (first-order derivative) and 2 (second-order derivative). To illustrate the feasibility of reliance on such a fractional-order derivative of θ, Fig. 5 again presents the four exemplary trials depicted in Fig. 3, but this time with a 1.6th-order fractional θ derivative added. As becomes clear from inspection of these new QuID plots, in all cases such an intermediate θ derivative correctly predicted the upcoming changes in steering direction marked by the horizontal lines. Moreover, it largely eliminated the rapid and prolonged sign-switching observed for d^2^θ/dt^2^ following the first steering event. We stress that the choice for an α = 1.6 fractional order derivative was made here purely for illustrative purposes. More systematically determining the exact fractional order(s) required for fully explaining the behaviours observed under all different target trajectory conditions^3^ is beyond the scope of the present study.

In the present framework we evaluated the potential of β-based and θ-based informational variables to guide locomotor interception behaviour under the assumption that control was grounded in continuously nulling (i.e., magnitude minimising) of any such informational variable(s). The analytic method deployed to this effect consisted of hunting for qualitative inconsistencies in the relation between observed and informationally-specified steering behaviour. Focussing to this end on quintessential changes in steering behaviour (i.e., from initially moving straight-ahead to moving leftward or rightward and, subsequently, from moving leftward to moving rightward or vice versa), the QuID method exploits transient regimes to pin down underlying operative control, by a process of elimination. In so doing, it differs fundamentally from methods focussing on steady-state regimes that reason on the basis of expected constancy of a particular informational variable^31–38^. In this light it is important to realise that online nulling of an informational variable does not necessarily lead to this variable becoming zero at some point, that is, to the system attaining a steady state. Indeed, empirical work on interception-by-steering of uniformly moving targets^4,5,22,37,38^ has revealed that under particular target trajectory conditions β and/or θ may become constant, as implicated by CTHA and CBA heuristics, but that under other target trajectory conditions interception may still be achieved without this being the case. Interestingly, in the present study with targets following circular trajectories, steering behaviour typically never settled into a clearly visible steady-state regime. This persistence of transients in steering behaviour over the course of action can in fact be readily understood as directly resulting from the non-uniformity of the target’s motion: for targets following circular trajectories, target motion continuously changes direction and thereby incessantly affects the state of informational variables, logically leading to continual (information-driven) changes in steering direction. Nevertheless, even without ever fully reaching a steady state, the combined dθ/dt and d^2^θ/dt^2^ nulling strategy identified in the present study allowed participants to intercept the target on the vast majority of the trials. Inspection of Fig. 5 does reveal that the exemplary 1.6th fractional order θ derivative did in fact approach zero in the later stages of action, before “blowing up” shortly before contact^38^. This latter effect no doubt arises from participants’ exploitation of the considerable (2-m radius) physical extent of the target, allowing them to contact the target without precisely aiming for its centre all the time.

In the end, the identified strategy of nulling some kind of combination of d^1^θ/dt^1^ and d^2^θ/dt^2^ information, that may in fact operate as a single informational variable of fractional-order dθ^α^/dt^α^ with 1 < α < 2, adequately captured the observed characteristics of the evolution over time of steering behaviour at the level of the individual trials under all curving target trajectory conditions that we tested. In our opinion this extraordinarily powerful result, fully compatible with comparable earlier work^3^, demonstrates the operational validity of the control by online information-nulling framework adopted. Of course, this does not imply that alternative theoretical frameworks for understanding trajectory formation in locomotor interception should simply be discarded. However, we do believe that we may reasonably argue that, in order to challenge the empirically well supported and conceptually parsimonious^8,19,20^ online information-nulling framework, alternative frameworks such as the model-based approach^49–51^ first need to move beyond anecdotal validation of their potential and demonstrate a comparably comprehensive explanatory ability of locomotor interception behaviour under varying target motion conditions. We note that the (to our knowledge) only locomotor interception study empirically assessing differential predictions from online- and model-based control perspectives concluded in favour of online control^20^.

## Methods

### Participants

Fourteen (post)graduate students from Aix-Marseille University (eight men and six women, aged 19.6 ± 1.3 years, *M* ± *SD*) participated in this experiment. They all had normal or corrected-to-normal vision. All participants provided informed consent before participating in the study. The study was approved by the French National Ethics Committee for Research in Sports Sciences (CERSTAPS) and conducted according to University regulations and the Declaration of Helsinki.

### Experimental set-up

The experiment took place in a large virtual reality facility (https://www.crvm.eu/). The setting consisted of four projection surfaces: a 3 × 3-m floor surface and three 4-m high × 3-m wide walls. The two sidewalls were set at 90° angles with respect to the front wall. The basic driving simulator, consisting of a seat, a set of (here non-operative) pedals, and a steering wheel, was positioned in the middle of the floor surface, with the steering wheel at a distance of 1.10 m from the front wall. Stereopsis was ensured with Volfoni® EDGE VR 3D Active glasses (120 Hz, 60 Hz per eye). These glasses were equipped with a configuration of reflective markers, allowing real-time motion capture of the head by an 8-camera Advanced Realtime Tracking (ART, Weilheim, Germany) opto-electronic system. The visual scene was refreshed at 60 Hz, taking into account the position and orientation of the participant’s head relative to the virtual environment.

### Task and procedure

Using in-house developed software (ICE), we simulated a virtual environment consisting of a large grass-like flat plain, containing both fine and gross texture, bordered by distant mountains. The seated participant was instructed that on each trial the goal was to steer the “car” so as to intercept (i.e., drive into) a horizontally-moving yellow cylinder (2-m radius, 3-m high). Prior to trial onset, participants moving at 20 m/s were to align locomotor direction with a yellow line by bringing the centre of the car (i.e., the seat) within a maximal lateral distance of 3 cm from the middle of the line, whilst moving in a direction that deviated less than 0.1° from the line orientation. Following successful alignment, the yellow line disappeared and a red portal appeared 40 m ahead. Participants were instructed to keep their steering wheel centred when moving toward the portal, thus steering straight toward it. During that period the steering wheel was deactivated with wheel orientation recalibrated to zero, so that when the participant crossed the portal and the target appeared they were moving straight ahead with ϕ = 0° and dϕ/dt = 0°/s. A trial ended when the participant drove into the target’s circumference (successful interception), or when the car’s in-depth (Z-axis) position exceeded the target’s in-depth position by 20 m.

Targets moved at 10 m/s along circular trajectories of 20-m or 40-m radius, starting at a constant in-depth (Z) distance of 60 m, from five possible lateral (X) departure positions: − 20, − 10, 0, + 10, + 20 m. Targets could move either leftward or rightward, giving rise to a total of 2 × 5 × 2 = 20 different target trajectories. The full set of 20 target trajectories was presented in a random order in each block of trials. Participants completed six blocks, for a total of 120 trials. In order to familiarise them with the environment and steering equipment, participants completed a block of 12 training trials in which they were to intercept targets moving along straight trajectories before start of the experiment.

### Data analysis

Participant position (x, z) and heading angle (ϕ) were sampled at 100 Hz and filtered using a fourth-order Butterworth filter with a 4-Hz cut-off frequency. For each individual trial, the presence of any salient steering action was determined following a two-criterion inclusion protocol. The first criterion was that the agent’s heading angle (ϕ) changed by at least 10° over the course of the trial. The second criterion was that at some point, after a minimal 100-ms duration into the trial, the rate of change in heading angle (dϕ/dt) exceeded an absolute value of 4°/s. Both criteria were met in 1659 of the total of 1680 trials; 21 trials were thus excluded. For each included trial, the time of onset of a steering action was determined by searching backward in time from the moment of occurrence of dϕ/dt > 4°/s to the moment that dϕ/dt first exceeded 1°/s for the first identified event or 0°/s for later events within the same trial, adding the criterion that the steering direction on a subsequent event needed to be opposite to the direction of the previous steering event. Finally, to ensure that the observed change in steering direction was sufficiently substantial, we required that heading angle changed at least 4° following a change in steering direction. If not, the event was not taken into account. To avoid extreme values in the final part of the interception action, we limited the timeframe for our search to maximally 200 ms before the moment of interception.

## Supplementary Information

Supplementary Figure 1.

Supplementary Information 2.

Supplementary Information 3.

Supplementary Information 4.

Supplementary Information 5.

Supplementary Information 6.

Supplementary Information 7.

Supplementary Information 8.

Supplementary Information 9.

Supplementary Information 10.

Supplementary Information 11.

Supplementary Information 12.

Supplementary Information 13.

Supplementary Information 14.

Supplementary Information 15.

Supplementary Information 16.

## Data Availability

All data generated or analysed during this study are included in graphical form in this published article and its supplementary information files. The numerical datasets are available from the corresponding author on reasonable request.
