# Supplementary material for: Characterisation of visual guidance of steering to intercept targets following curving trajectories using Qualitative Inconsistency Detection
Source: Sci Rep. 2022 Nov 24;12:20246. doi: 10.1038/s41598-022-24625-4 (PMC9691627; doi:10.1038/s41598-022-24625-4)

Steering to intercept targets following curving trajectories: Insights into visual guidance from Qualitative Inconsistency Detection

Daphne van Opstal, Remy Casanova, Frank T.J.M. Zaal, and Reinoud J. Bootsma

Supplementary Information Figure S1. Page-size landscape-oriented enlargements of the four individual panels of main text Fig. 3, with **Fig. S1a** representing the S20/R20-OUT (rightward) trial of Participant 10 in Block 3, **Fig. S1b** representing the S20/R40-OUT (leftward) trial of Participant 11 in Block 3, **Fig. S1c** representing the S20/R40-IN (rightward) trial of Participant 7 in Block 3, and **Fig. S1d** representing the S10/R40-IN (leftward) trial of Participant 12 in Block 3.

Figure Legend

Left graph in each panel (page): Spatial paths followed by the target (dotted grey line) and the participant (black line). Steering events are marked by colour-coded dots.

Right graphs in each panel (page): Time evolution (bottom to top) over the course of the trial of the participant's heading direction ϕ (in green), the target-heading angle β (in red) and the target's bearing angle θ (in blue) together with their first-order (dashed) and second-order (dash-dotted) time derivatives.

Horizontal grey lines situate the steering events spatially (left graph) and temporally (right graphs).

P10/B3
S20/R20-OUT

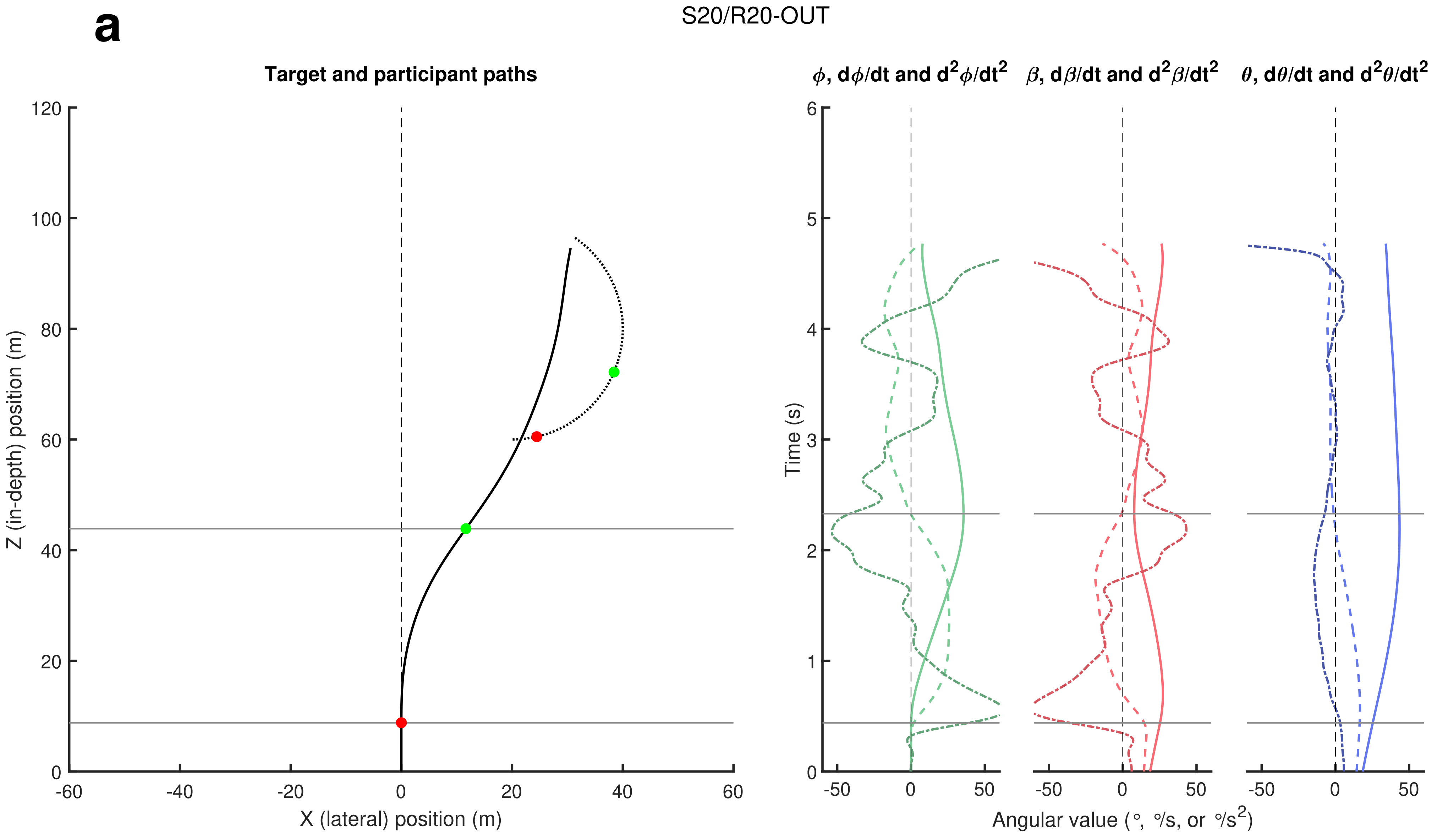

P11/B3
S20/R40-OUT

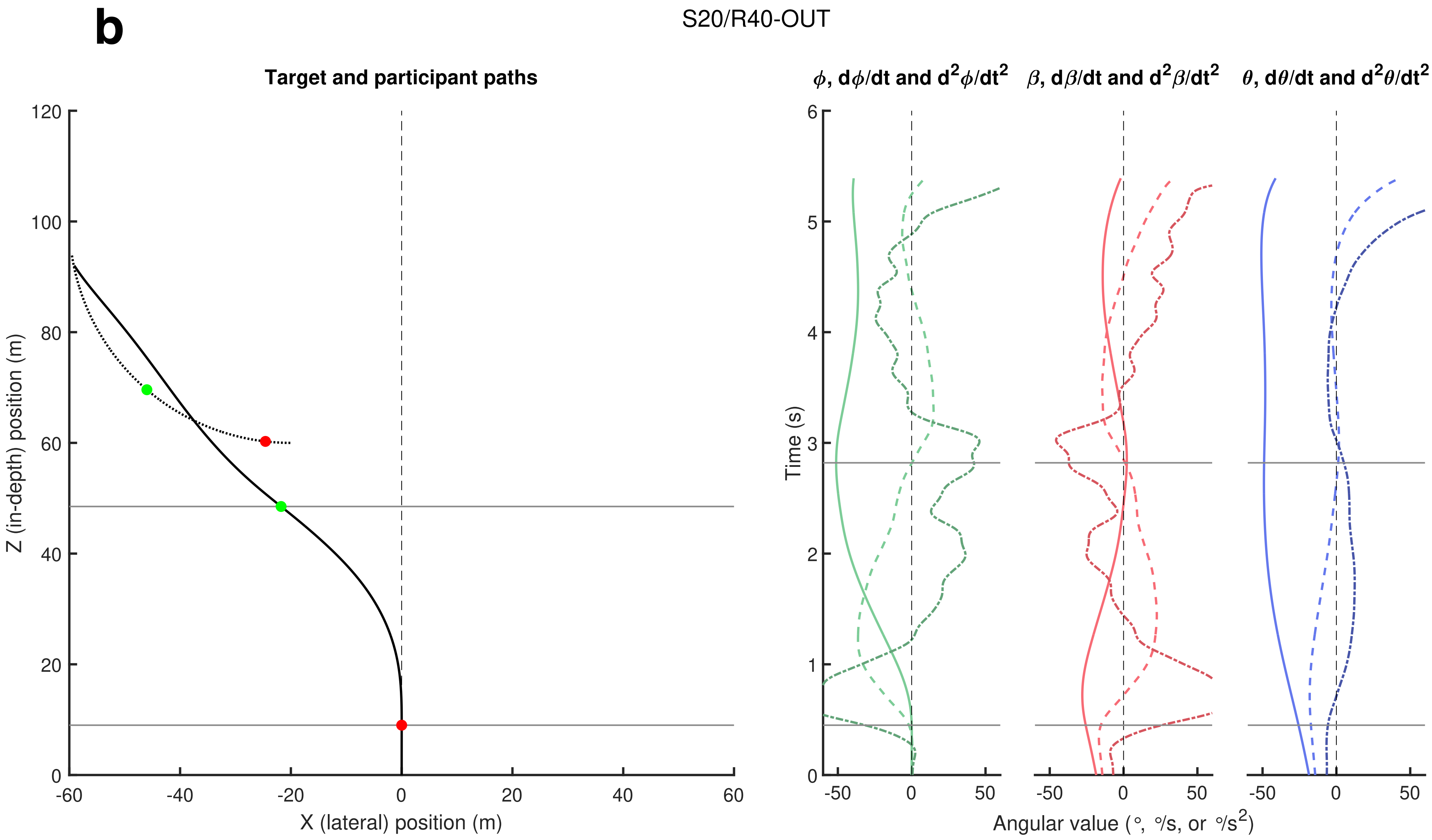

P7/B3
S20/R20-IN

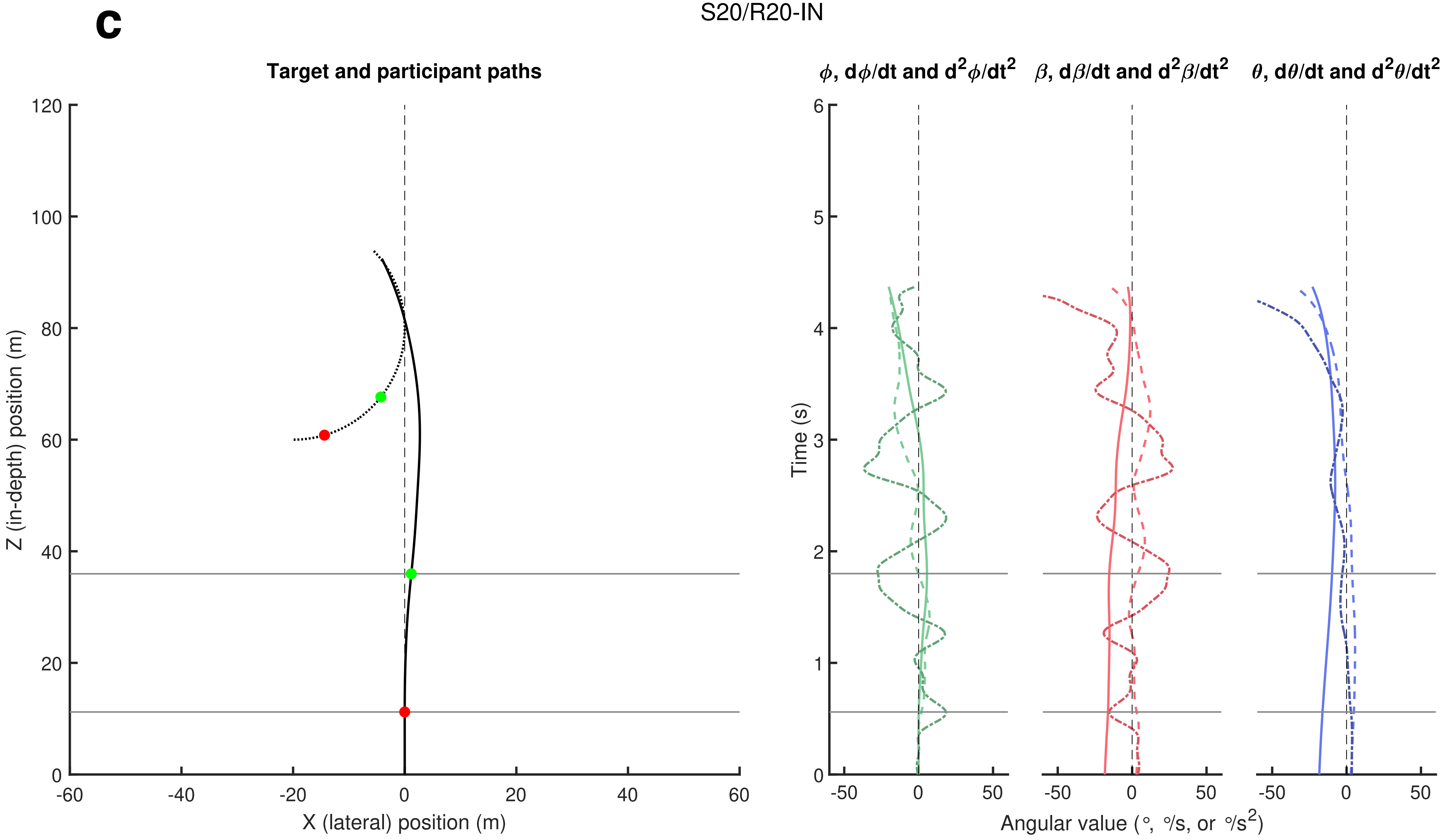

P12/B3
S10/R40-IN

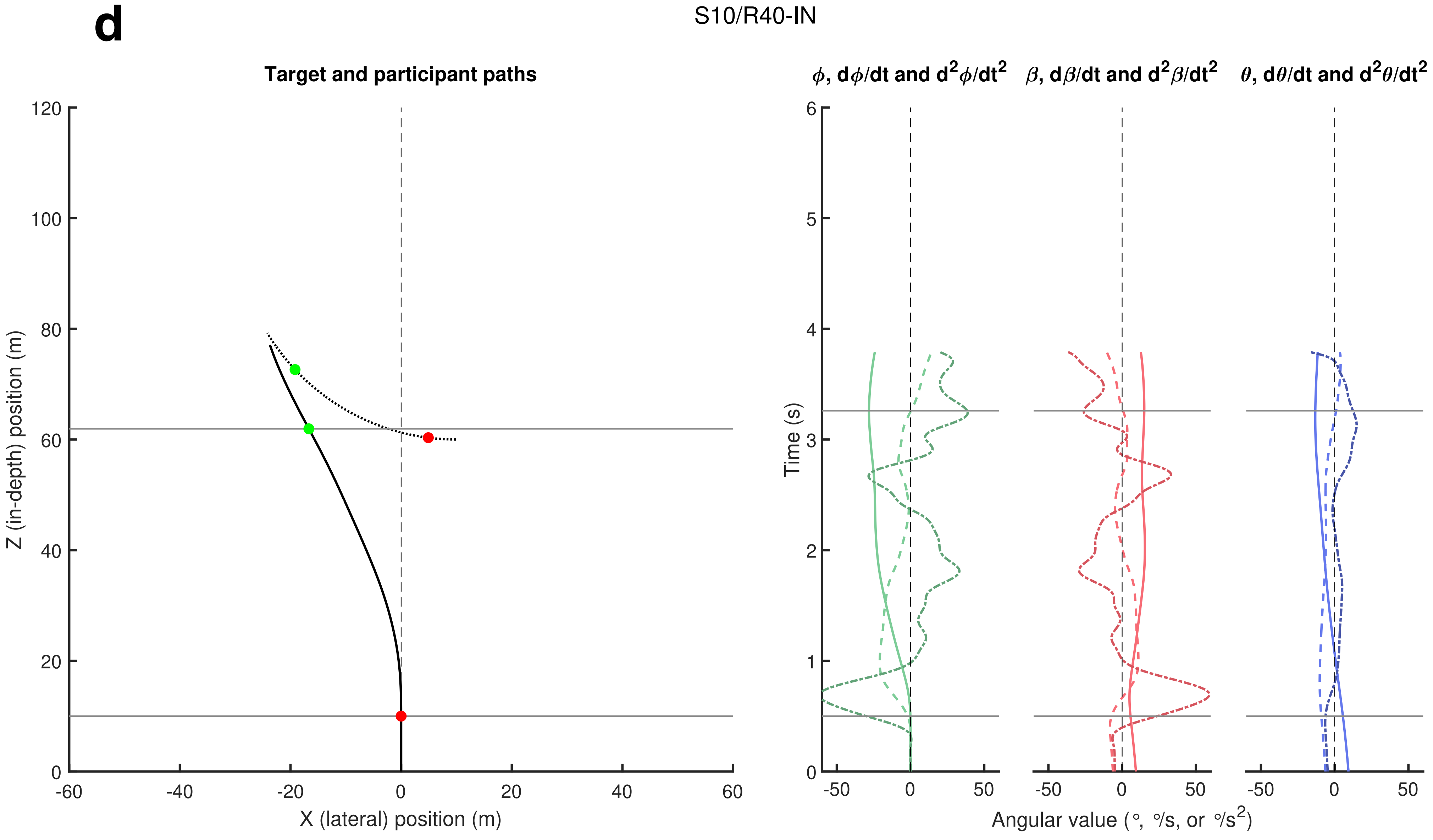

Supplement: Supplementary file 1 — Supplementary Figure 1. [file 41598_2022_24625_MOESM1_ESM.pdf]
