# Supplementary material for: Characterisation of visual guidance of steering to intercept targets following curving trajectories using Qualitative Inconsistency Detection
Source: Sci Rep. 2022 Nov 24;12:20246. doi: 10.1038/s41598-022-24625-4 (PMC9691627; doi:10.1038/s41598-022-24625-4)
Supplement: Supplementary file 2 — Supplementary Information 2. [file 41598_2022_24625_MOESM2_ESM.pdf]

Characterisation of visual guidance of steering to intercept targets following curving trajectories using Qualitative Inconsistency Detection

Albertha A.M. van Opstal, Remy Casanova, Frank T.J.M. Zaal, and Reinoud J. Bootsma

Supplementary information content:

Supplementary Fig. 1: Page-size landscape-oriented enlargements of the four individual panels of Fig. 3 presented in the main text, with Fig.S1a representing the S20/R20-OUT (rightward) trial of Participant 10 in Block 3, Fig.S1b representing the S20/R40-OUT (leftward) trial of Participant 11 in Block 3, Fig.S1c representing the S20/R40-IN (rightward) trial of Participant 7 in Block 3, and Fig.S1d representing the S10/R40-IN (leftward) trial of Participant 12 in Block 3.

SI-3 = Supplementary Fig. P1: Page-size landscape-oriented QuID plots for all 120 individual trials of the Participant 1.

SI-4 = Supplementary Fig. P2: Page-size landscape-oriented QuID plots for all 120 individual trials of the Participant 2.

SI-5 = Supplementary Fig. P3: Page-size landscape-oriented QuID plots for all 120 individual trials of the Participant 3.

SI-6 = Supplementary Fig. P4: Page-size landscape-oriented QuID plots for all 120 individual trials of the Participant 4.

SI-7 = Supplementary Fig. P5: Page-size landscape-oriented QuID plots for all 120 individual trials of the Participant 5.

SI-8 = Supplementary Fig. P6: Page-size landscape-oriented QuID plots for all 120 individual trials of the Participant 6.

SI-9 = Supplementary Fig. P7: Page-size landscape-oriented QuID plots for all 120 individual trials of the Participant 7.

SI-10 = Supplementary Fig. P8: Page-size landscape-oriented QuID plots for all 120 individual trials of the Participant 8.

SI-11 = Supplementary Fig. P9: Page-size landscape-oriented QuID plots for all 120 individual trials of the Participant 9.

SI-12 = Supplementary Fig. P10: Page-size landscape-oriented QuID plots for all 120 individual trials of the Participant 10.

SI-13 = Supplementary Fig. P11: Page-size landscape-oriented QuID plots for all 120 individual trials of the Participant 11.

SI-14 = Supplementary Fig. P12: Page-size landscape-oriented QuID plots for all 120 individual trials of the Participant 12.

SI-15 = Supplementary Fig. P13: Page-size landscape-oriented QuID plots for all 120 individual trials of the Participant 13.

SI-16 = Supplementary Fig. P14: Page-size landscape-oriented QuID plots for all 120 individual trials of the Participant 14.
