# Supplementary material for: Characterisation of visual guidance of steering to intercept targets following curving trajectories using Qualitative Inconsistency Detection
Source: Sci Rep. 2022 Nov 24;12:20246. doi: 10.1038/s41598-022-24625-4 (PMC9691627; doi:10.1038/s41598-022-24625-4)

Supplementary Figure S2-P6. Page-size landscape-oriented QuID plots for all 120 individual trials of Participant 6 (one QuID plot per page)

Figure (subplot) titles: Located top-centre on each Figure page, with identification code of the specific trial (e.g., p. 2: P6/B1 S20/R20-IN) where P refers to Participant number (1 to 14), B refers to Block number (1 to 6) and S20/R20-IN refers to target trajectory characteristics (see Table 1 in main text).

Figure legends. Identical for all 120 subplots

Left graph on each page: Spatial paths followed by the target (dotted grey line) and the participant (black line). Steering events are marked by colour-coded dots.

Right graphs on each page: Time evolution (bottom to top) over the course of the trial of the participant's heading direction ϕ (in green), the target-heading angle β (in red) and the target's bearing angle θ (in blue) together with their first-order (dashed) and second-order (dash-dotted) time derivatives.

Horizontal grey lines situate the steering events spatially (left graph) and temporally (right graphs).

P6/B1
S20/R20-IN

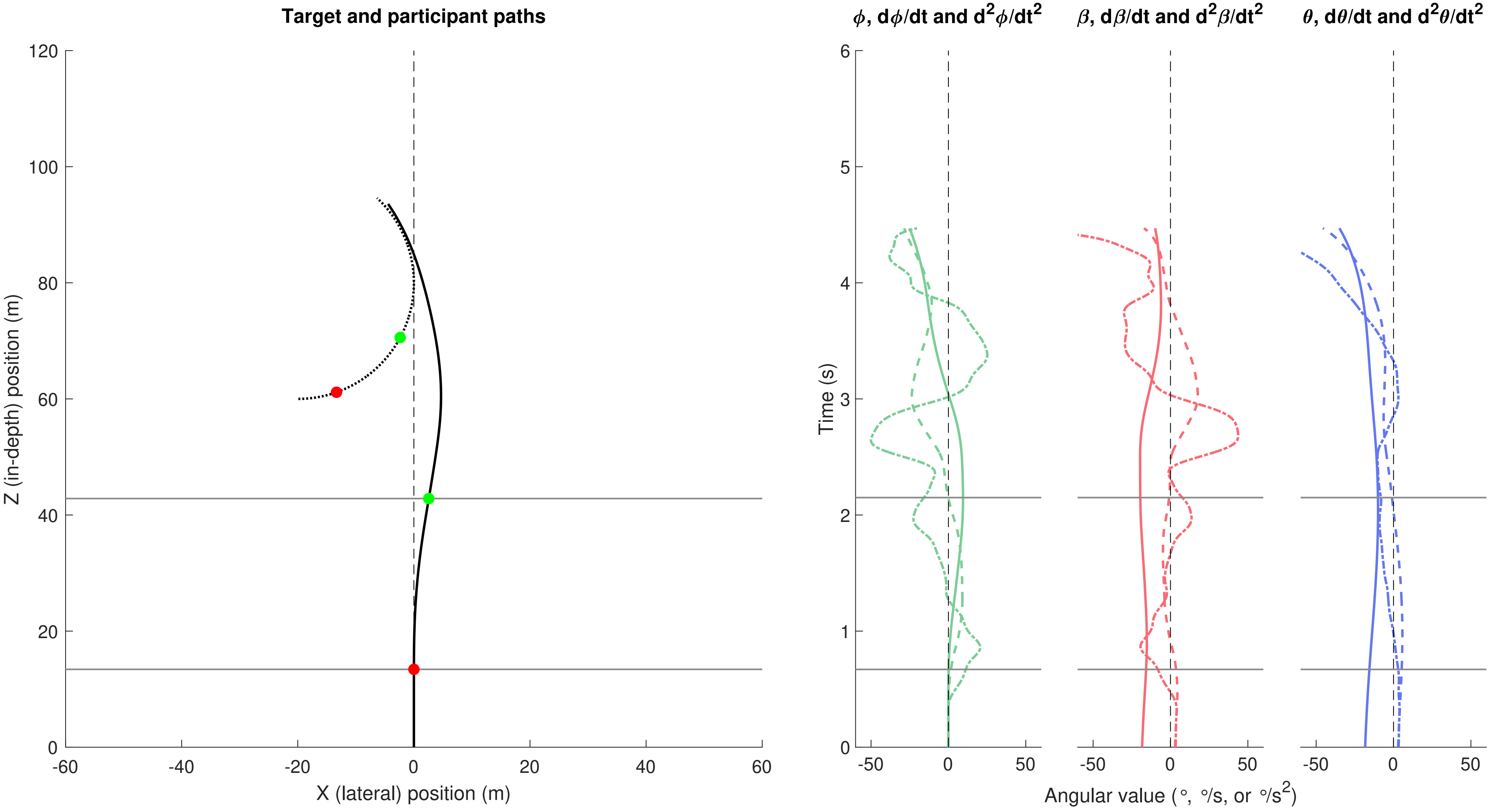

P6/B1
S20/R20-OUT

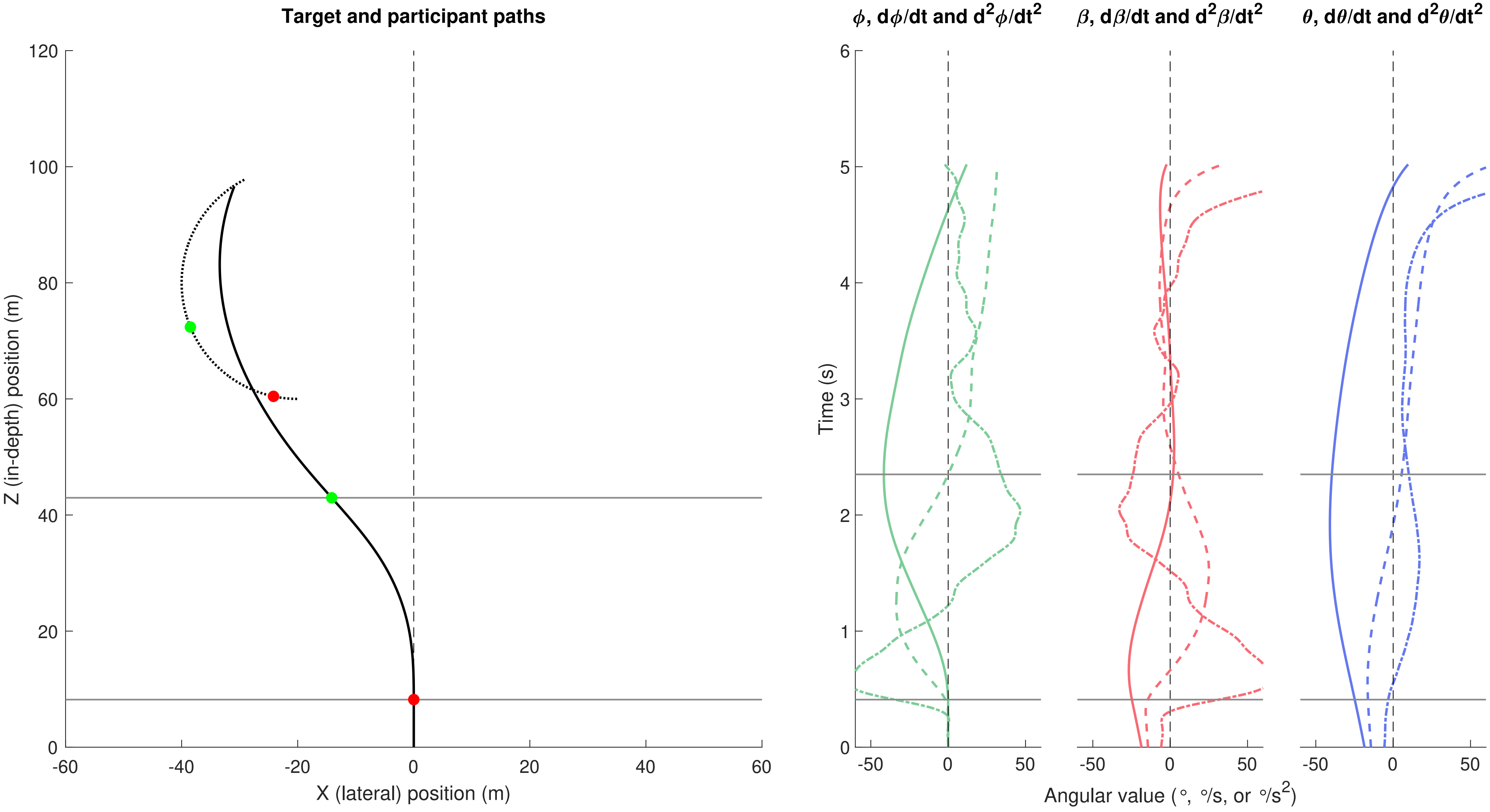

P6/B1
S20/R40-IN

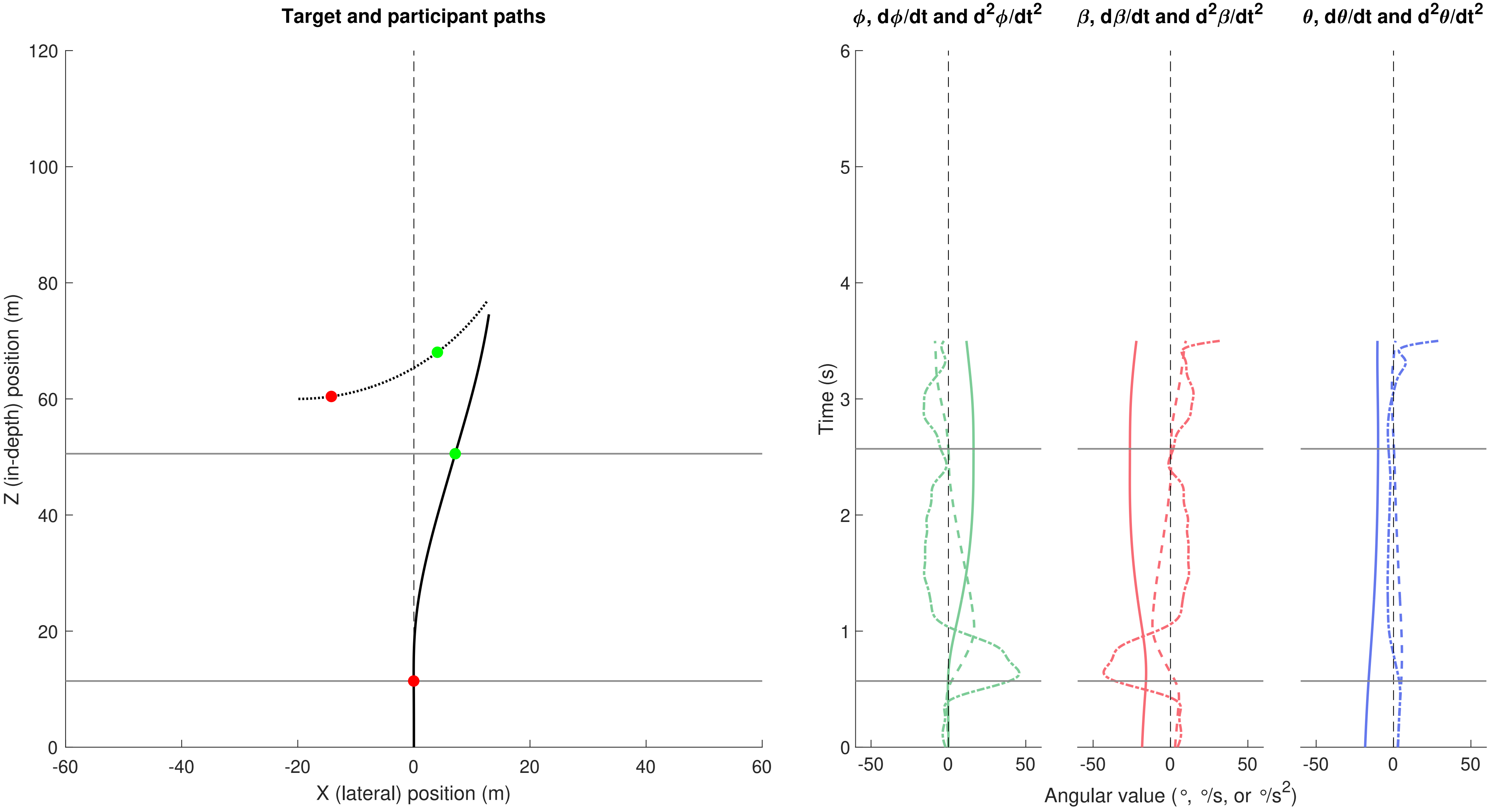

P6/B1
S20/R40-OUT

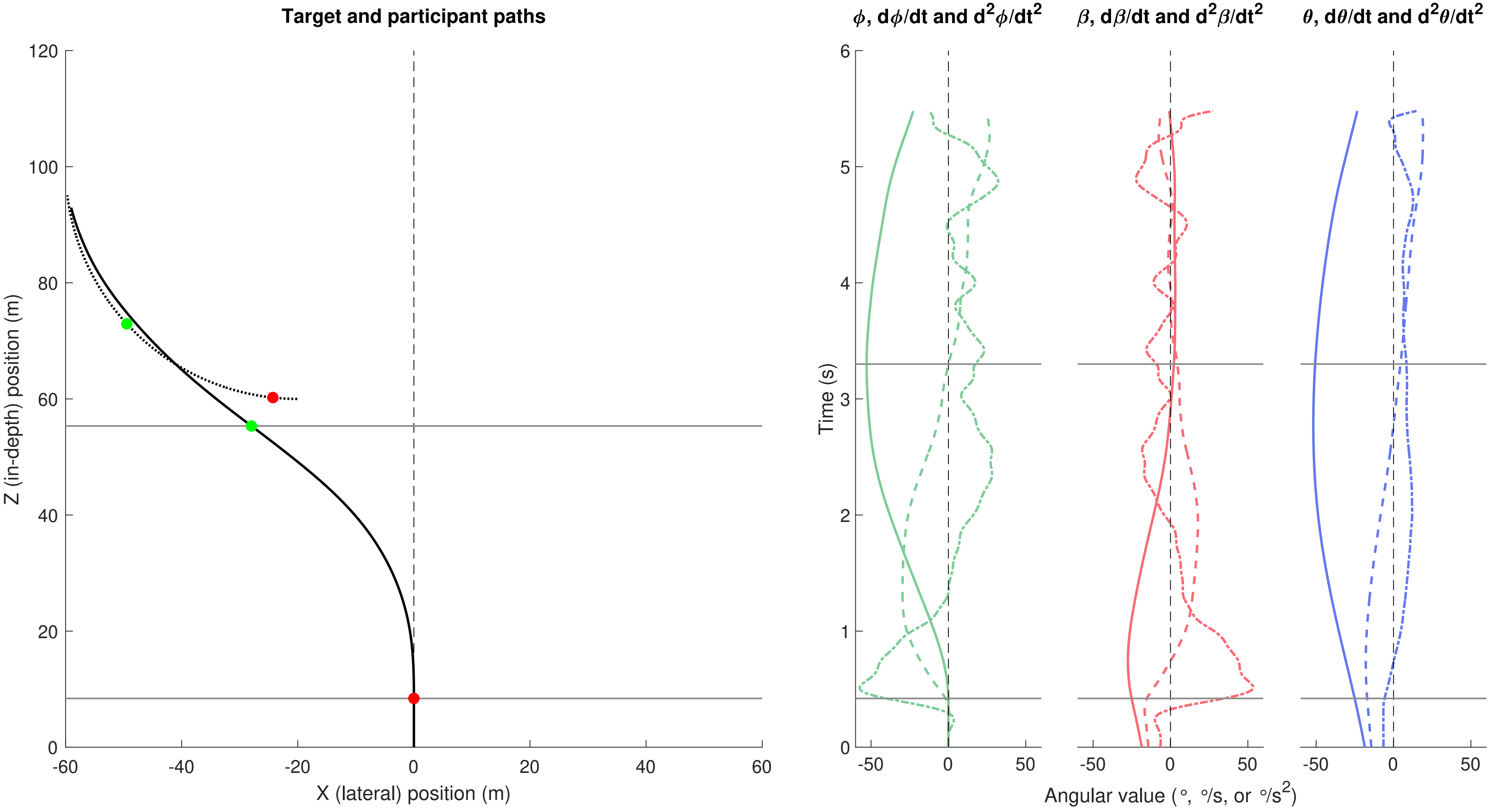

P6/B1
S10/R20-IN

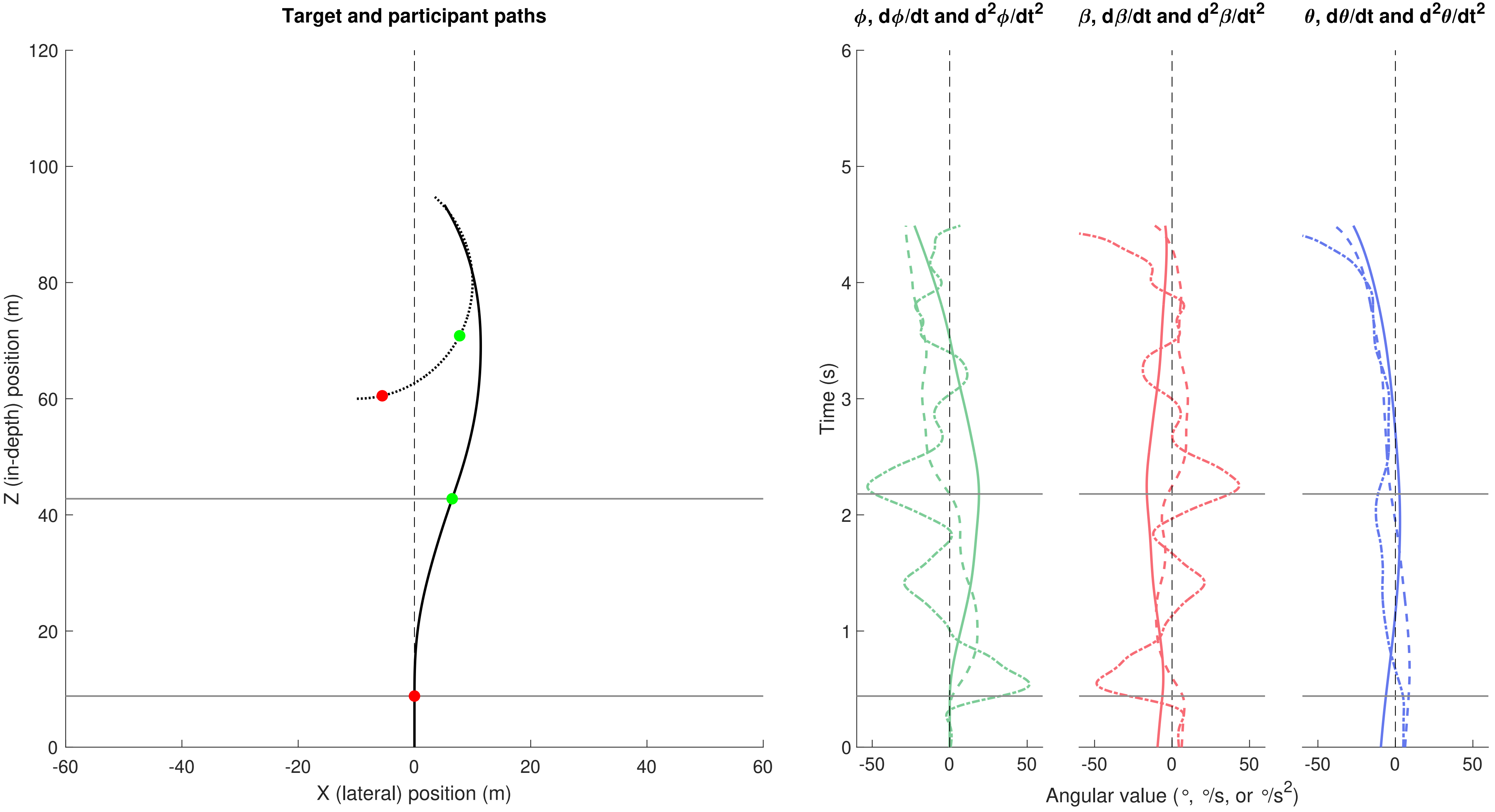

P6/B1
S10/R20-OUT

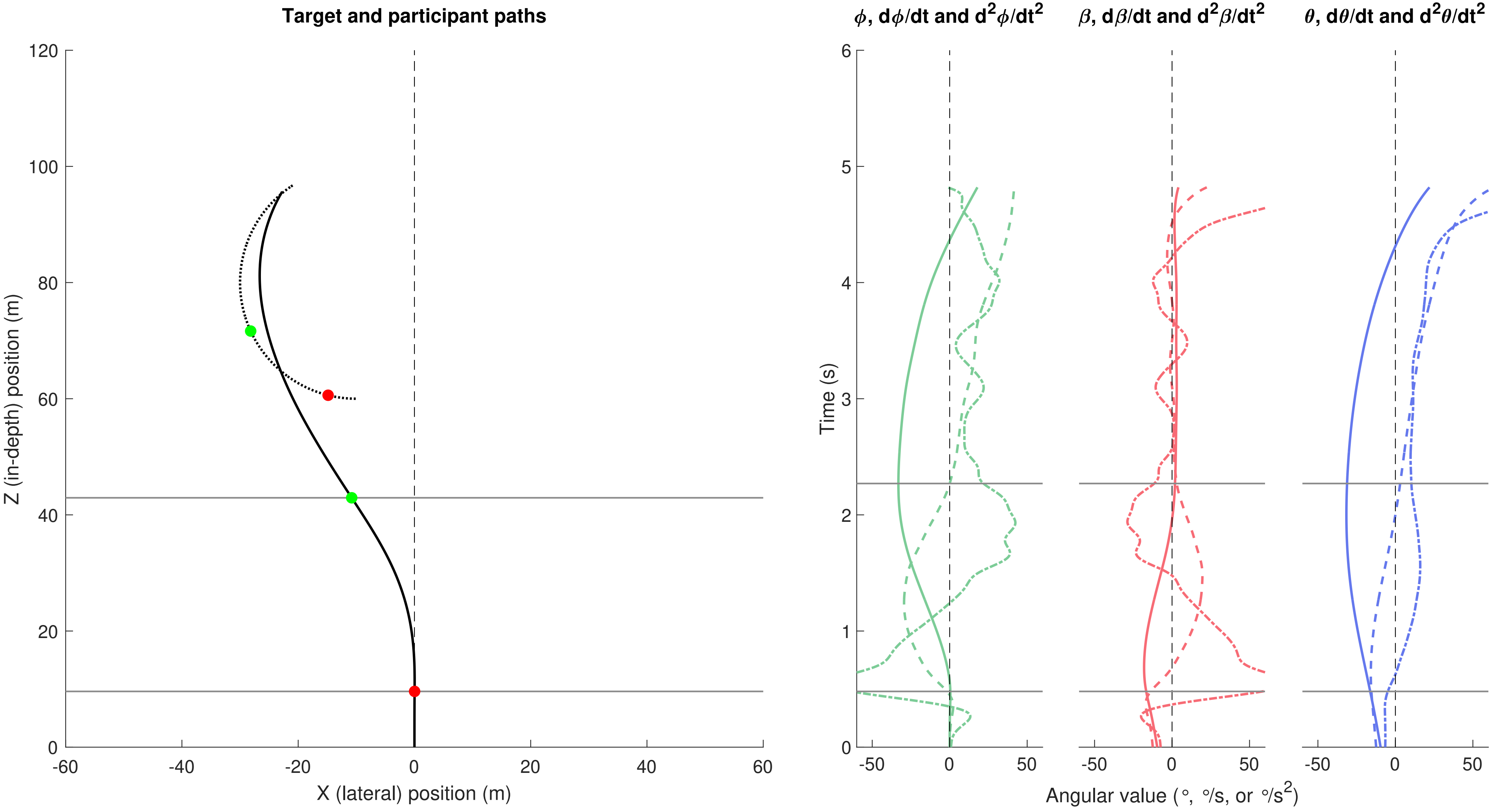

Participant 6

P6/B1
S10/R40-IN

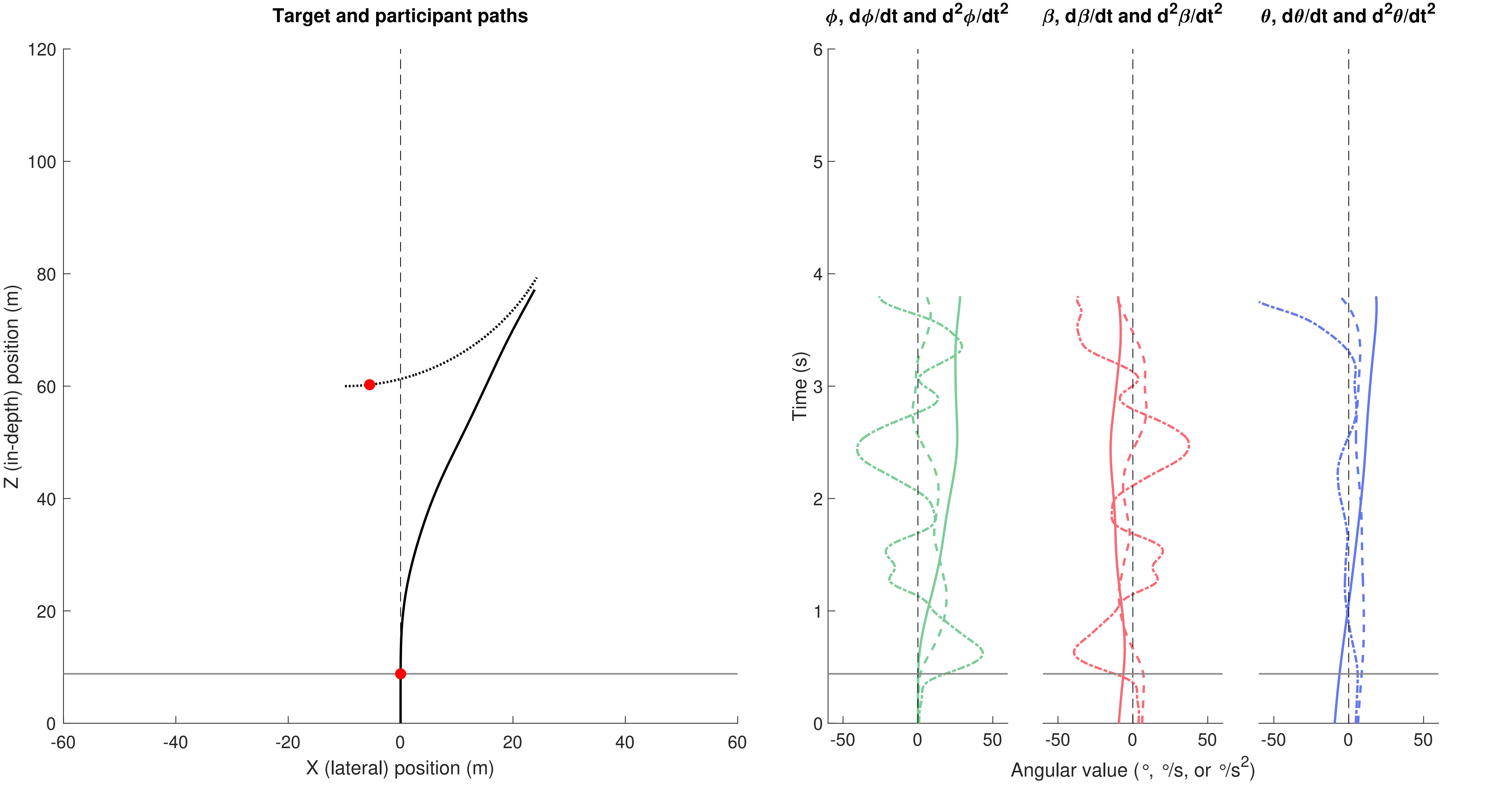

P6/B1
S10/R40-OUT

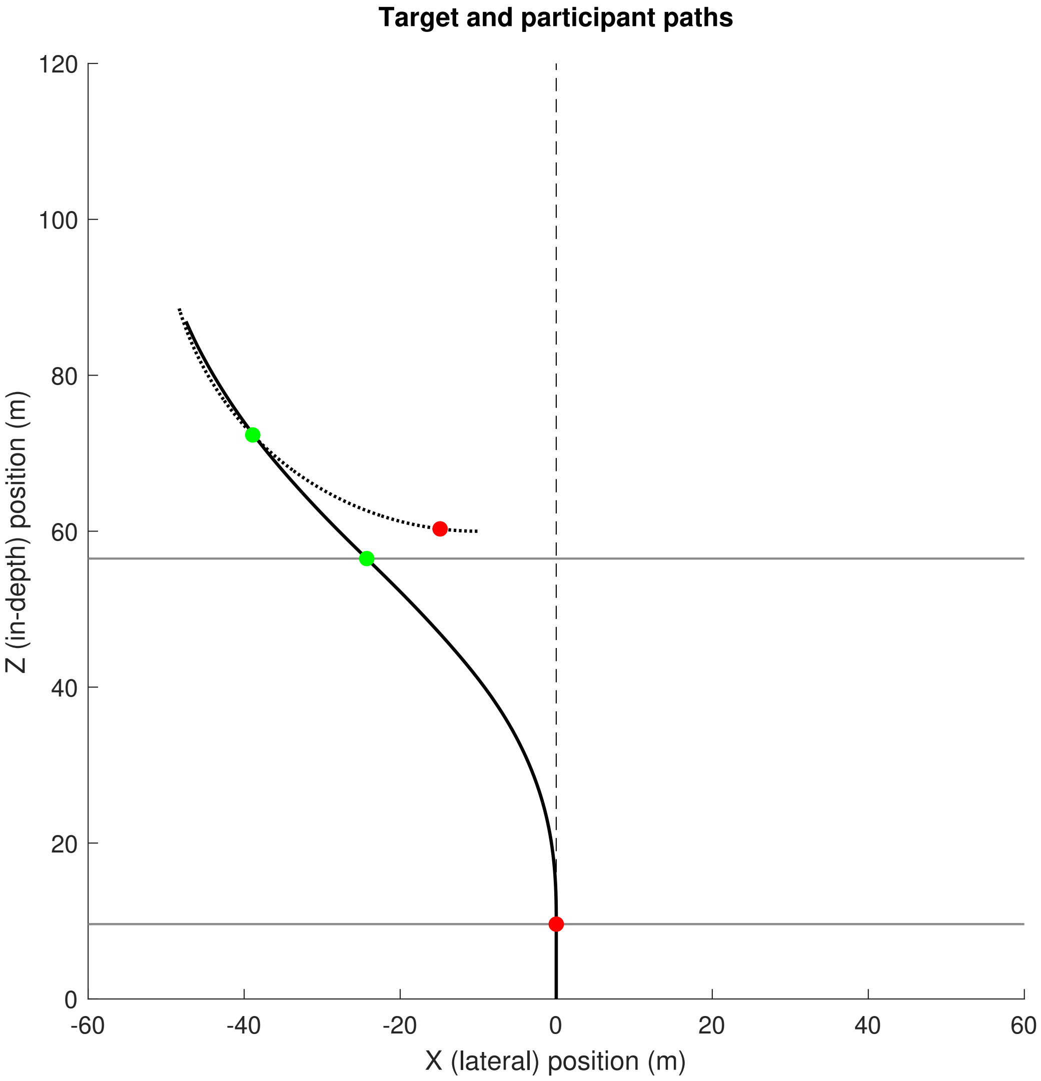

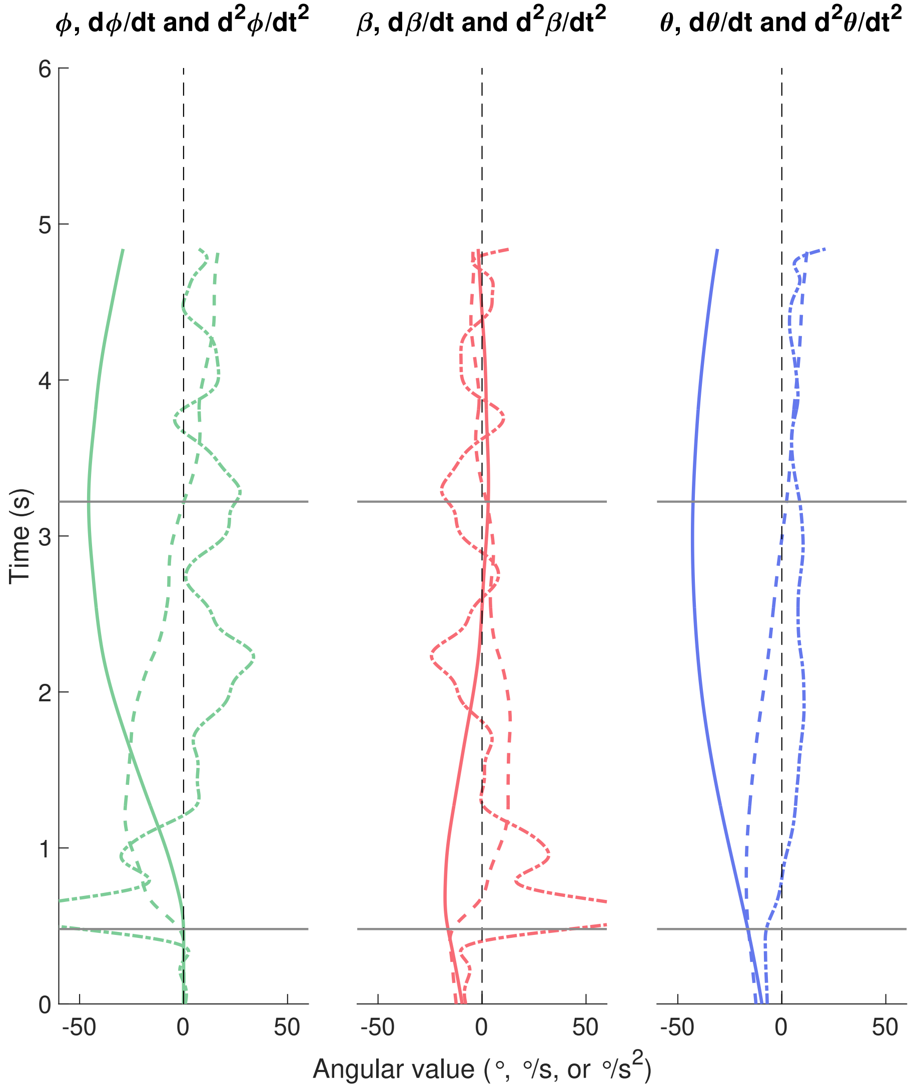

P6/B1
S0/R20-OUT

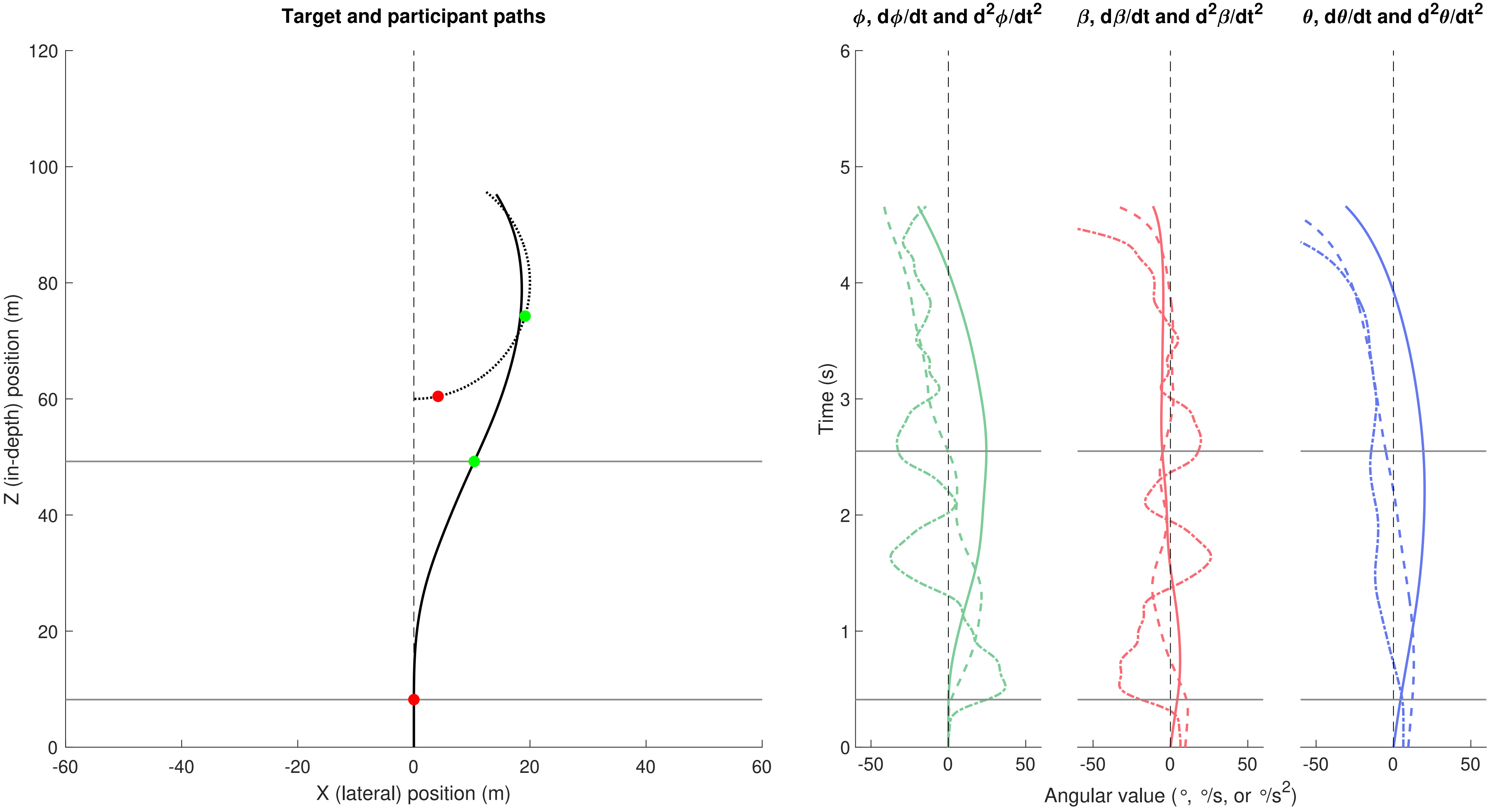

Participant 6

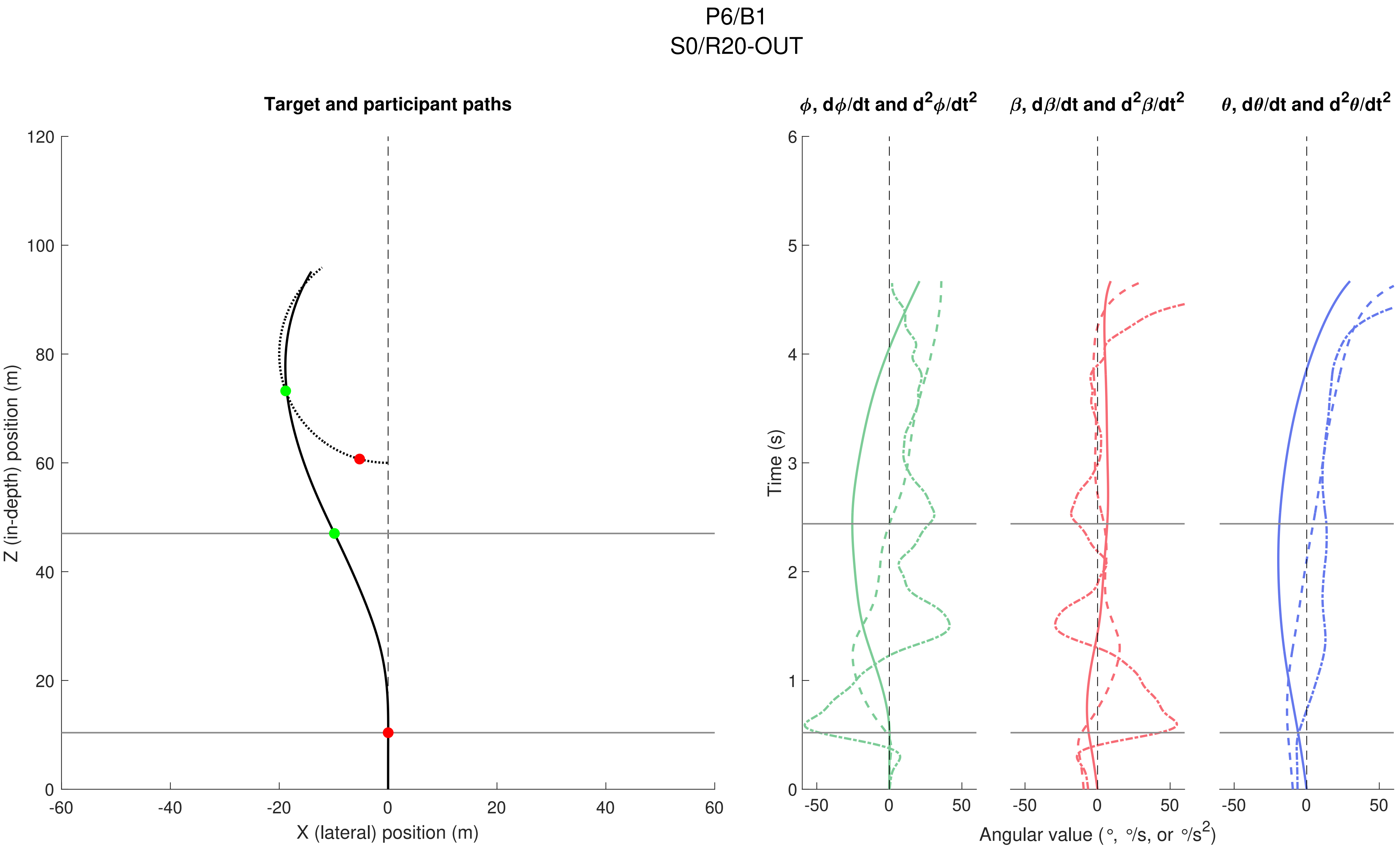

P6/B1
S0/R40-OUT

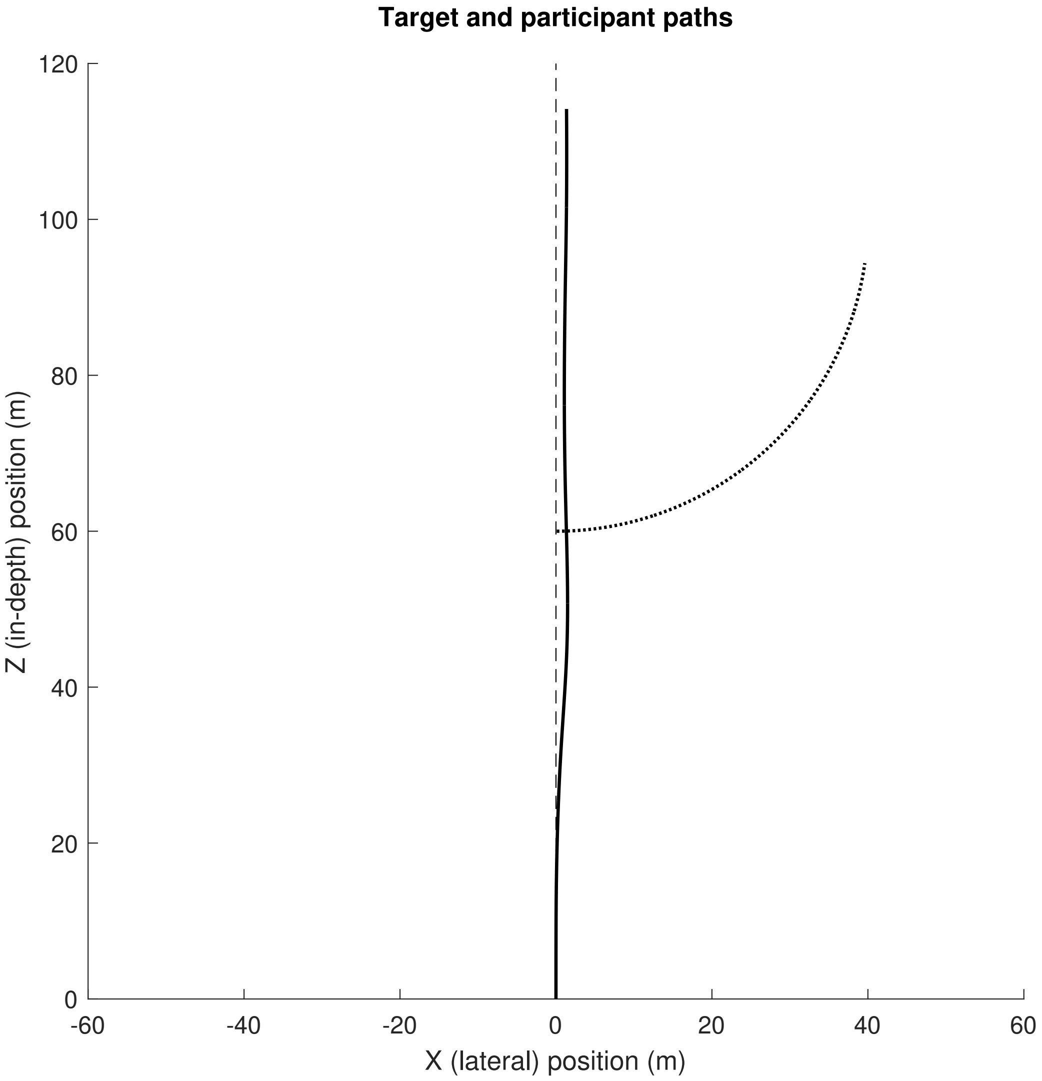

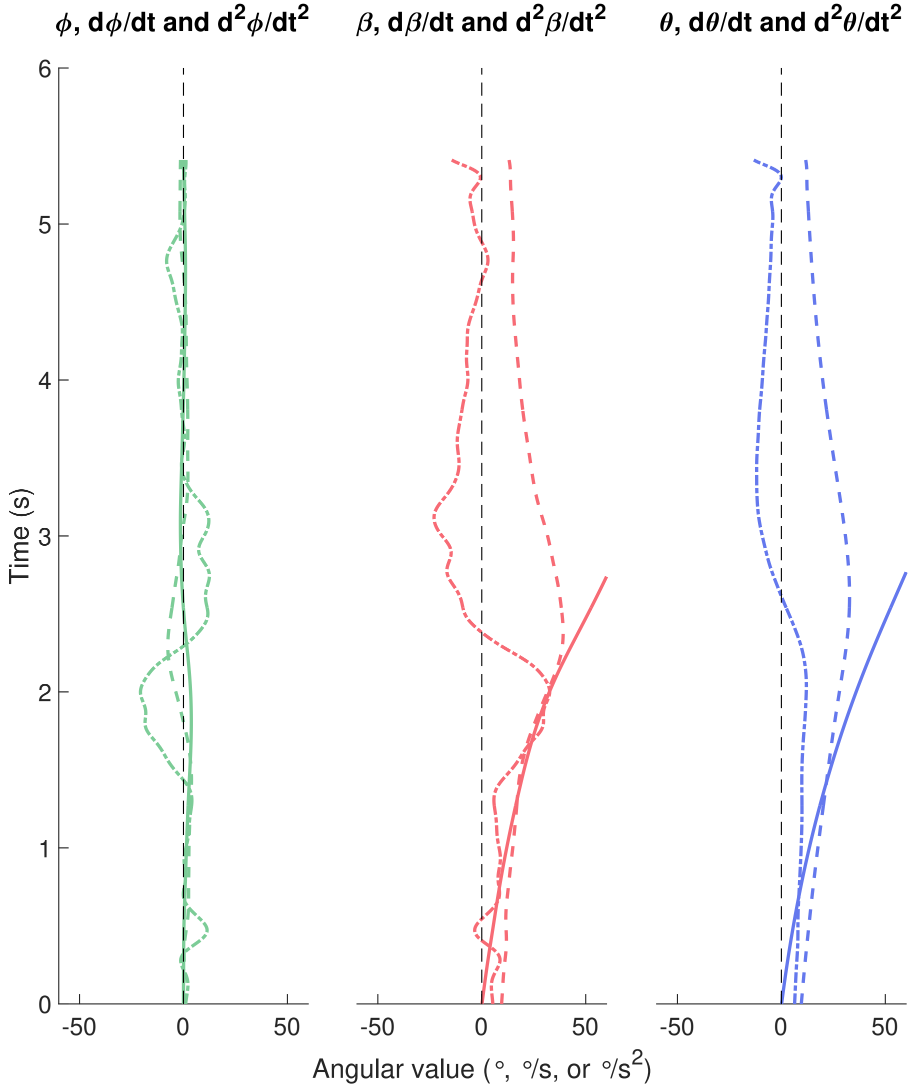

P6/B1
S0/R40-OUT

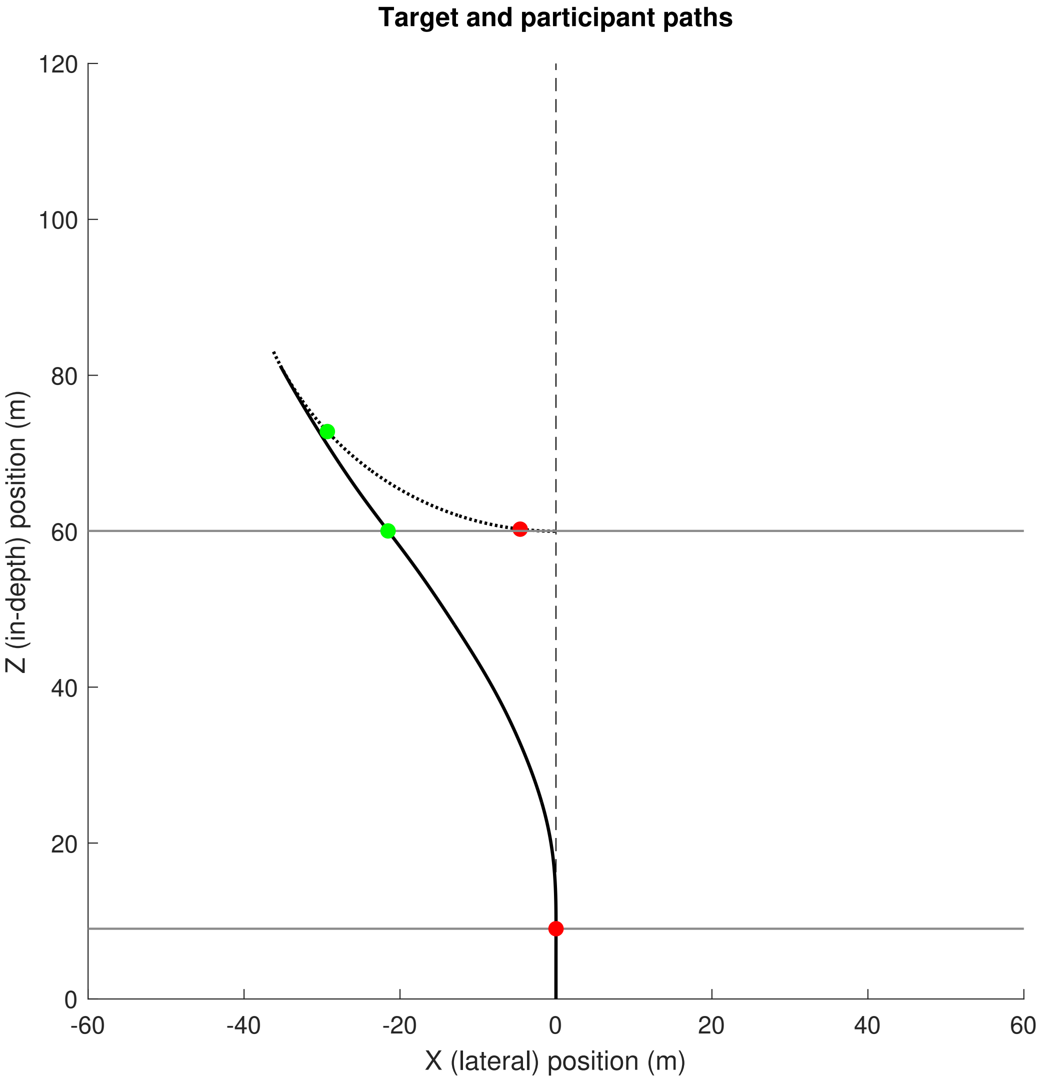

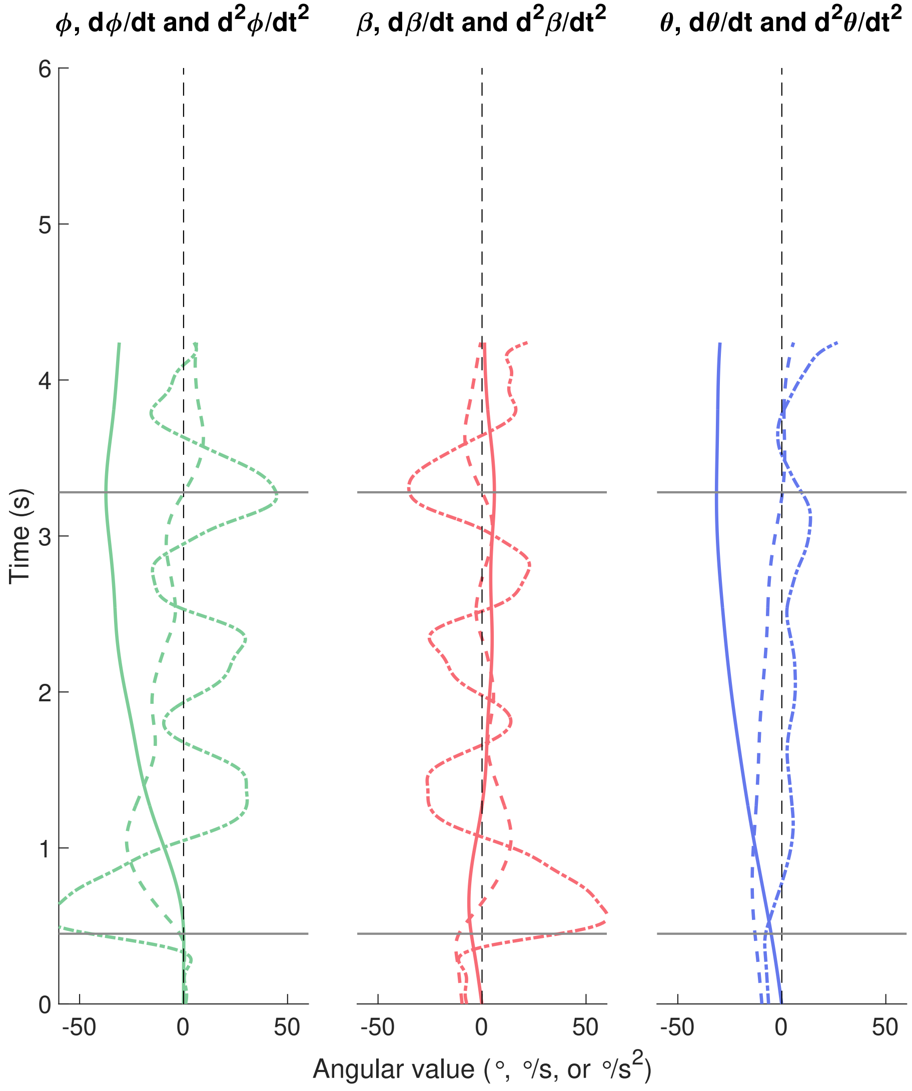

P6/B1
S10/R20-OUT

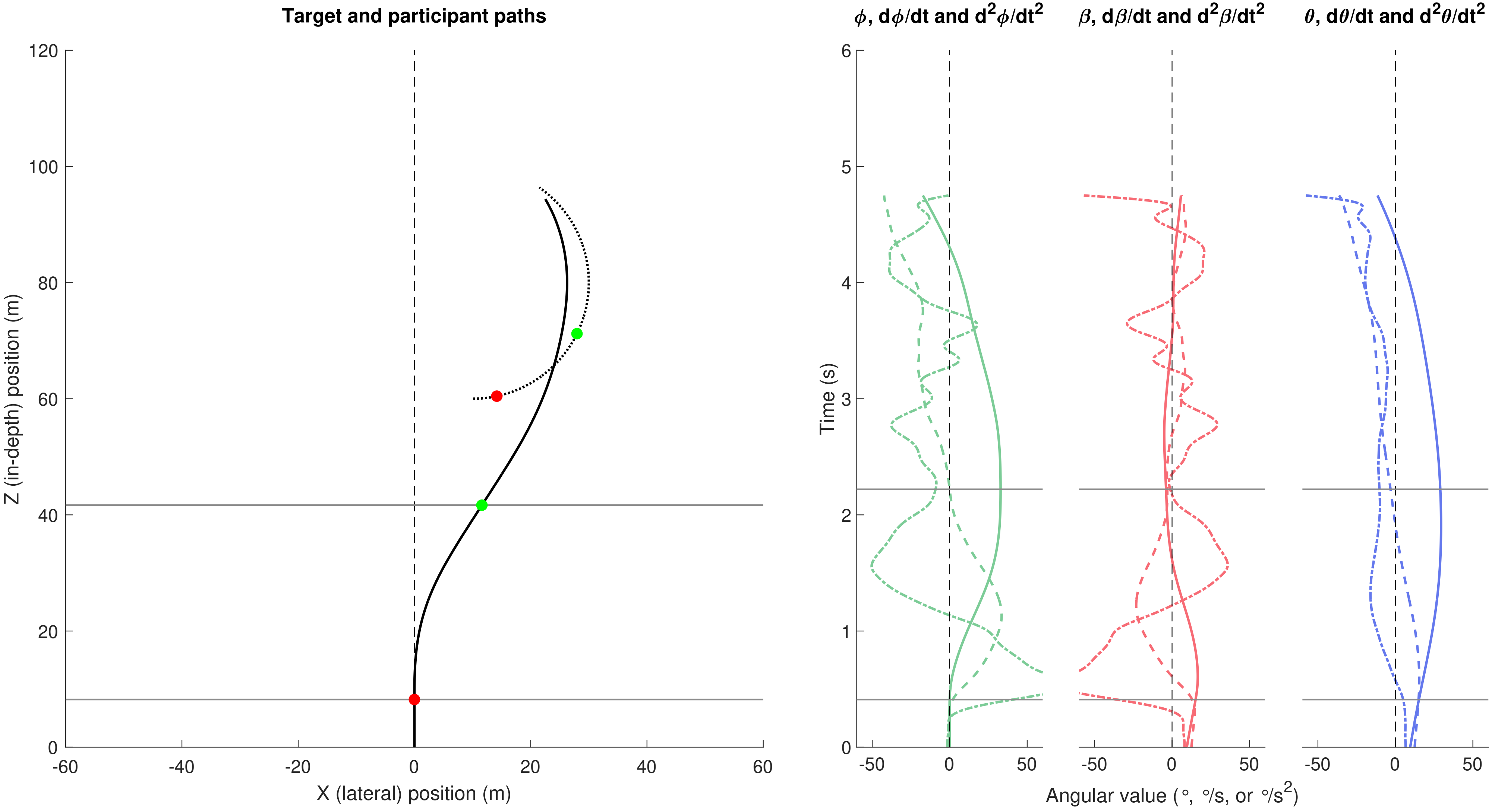

P6/B1
S10/R20-IN

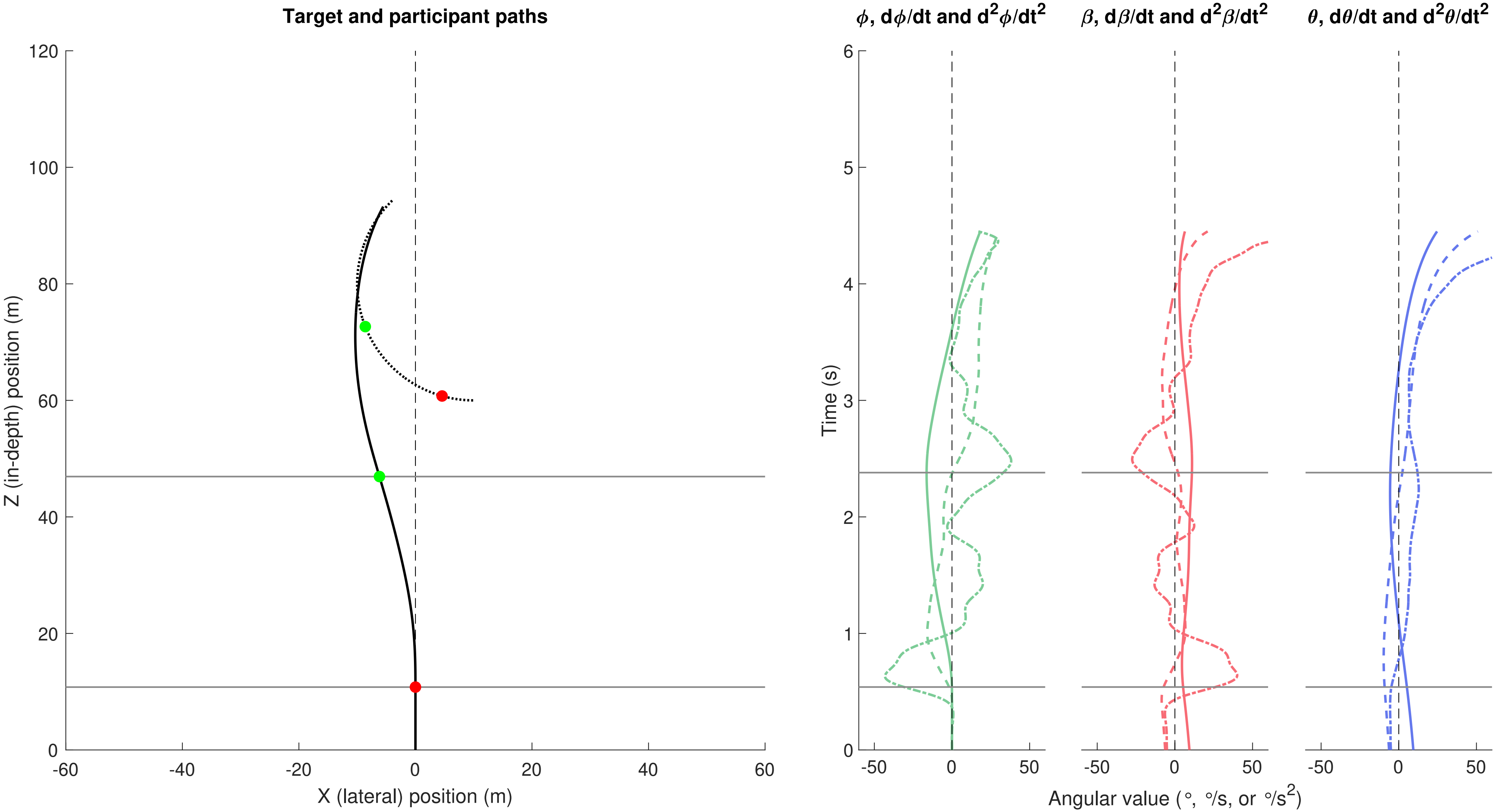

P6/B1
S10/R40-OUT

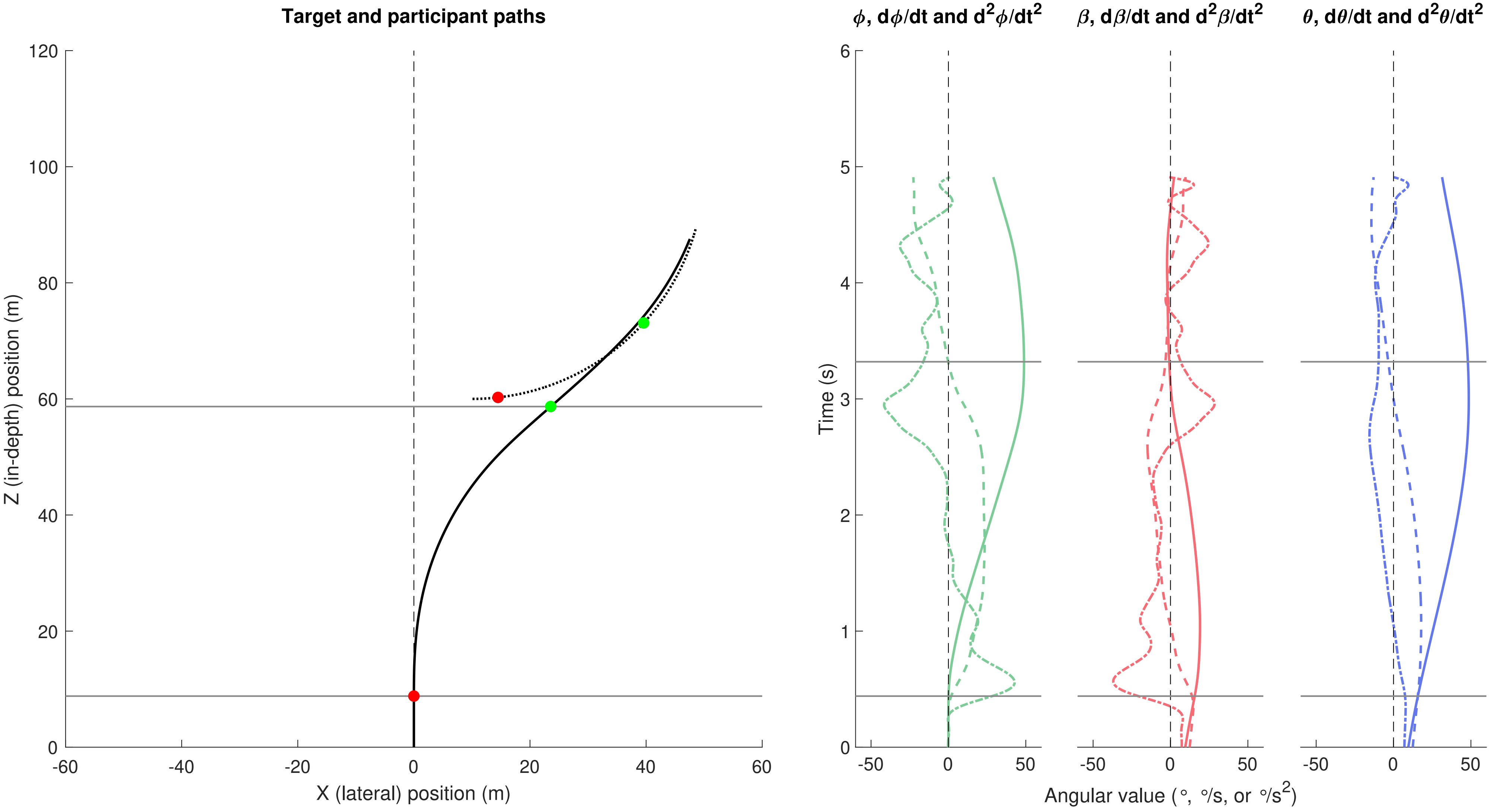

P6/B1
S10/R40-IN

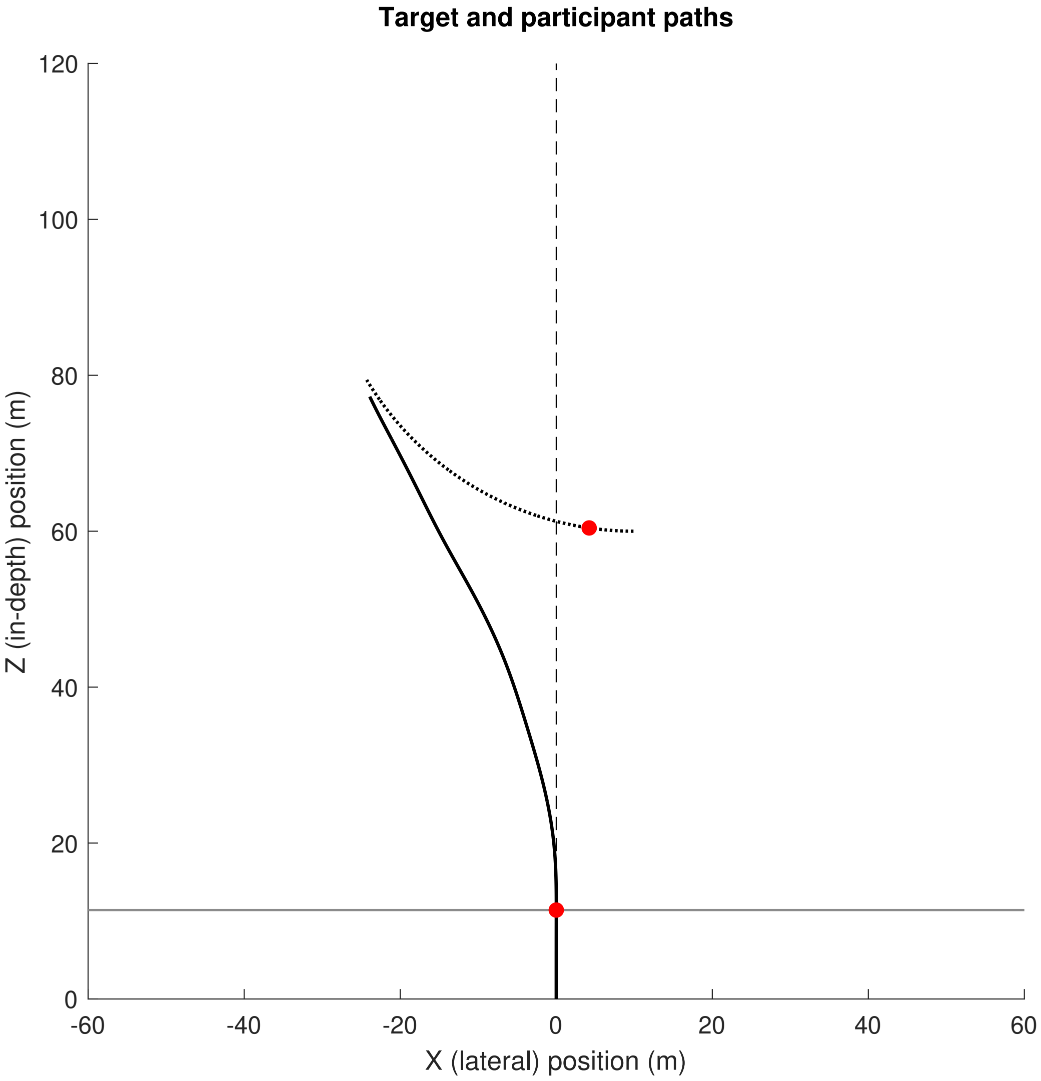

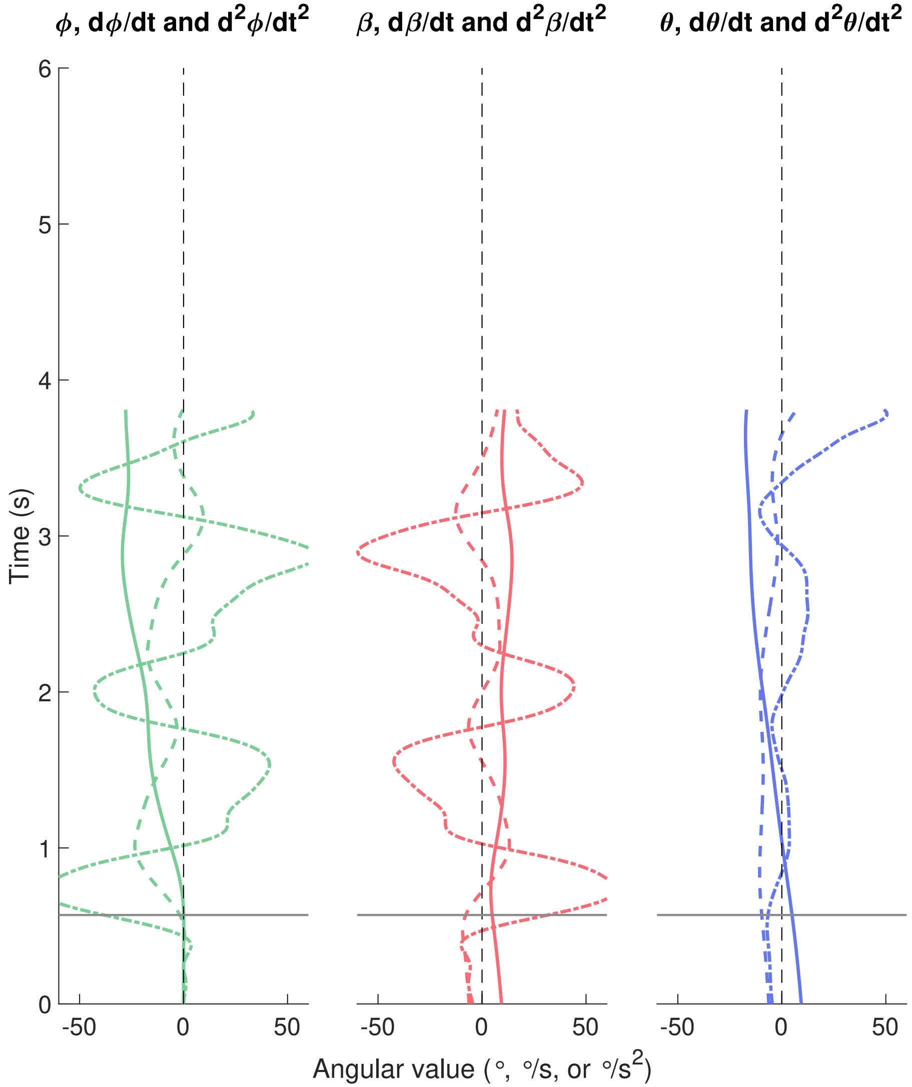

P6/B1
S20/R20-OUT

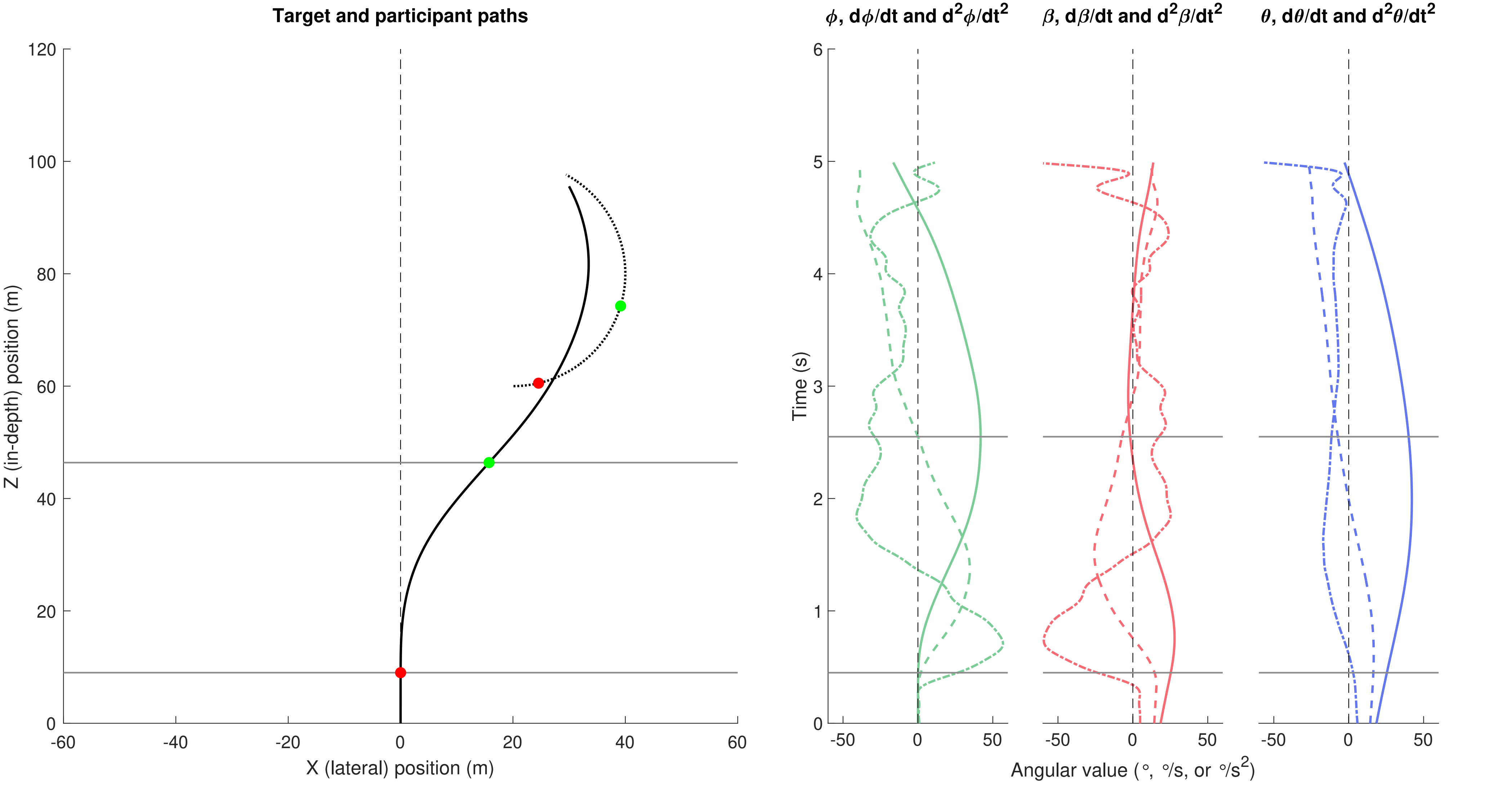

P6/B1
S20/R20-IN

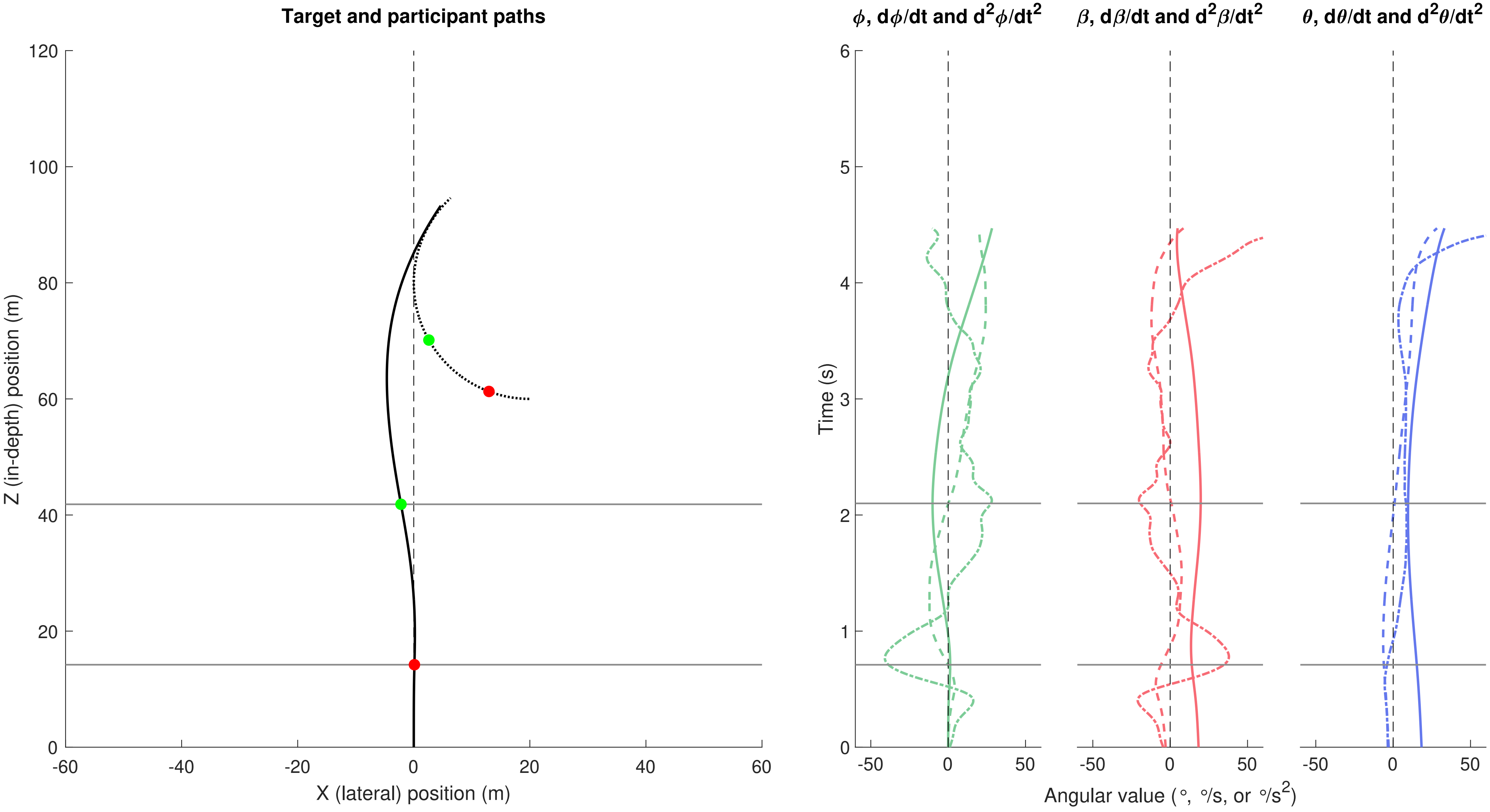

P6/B1
S20/R40-OUT

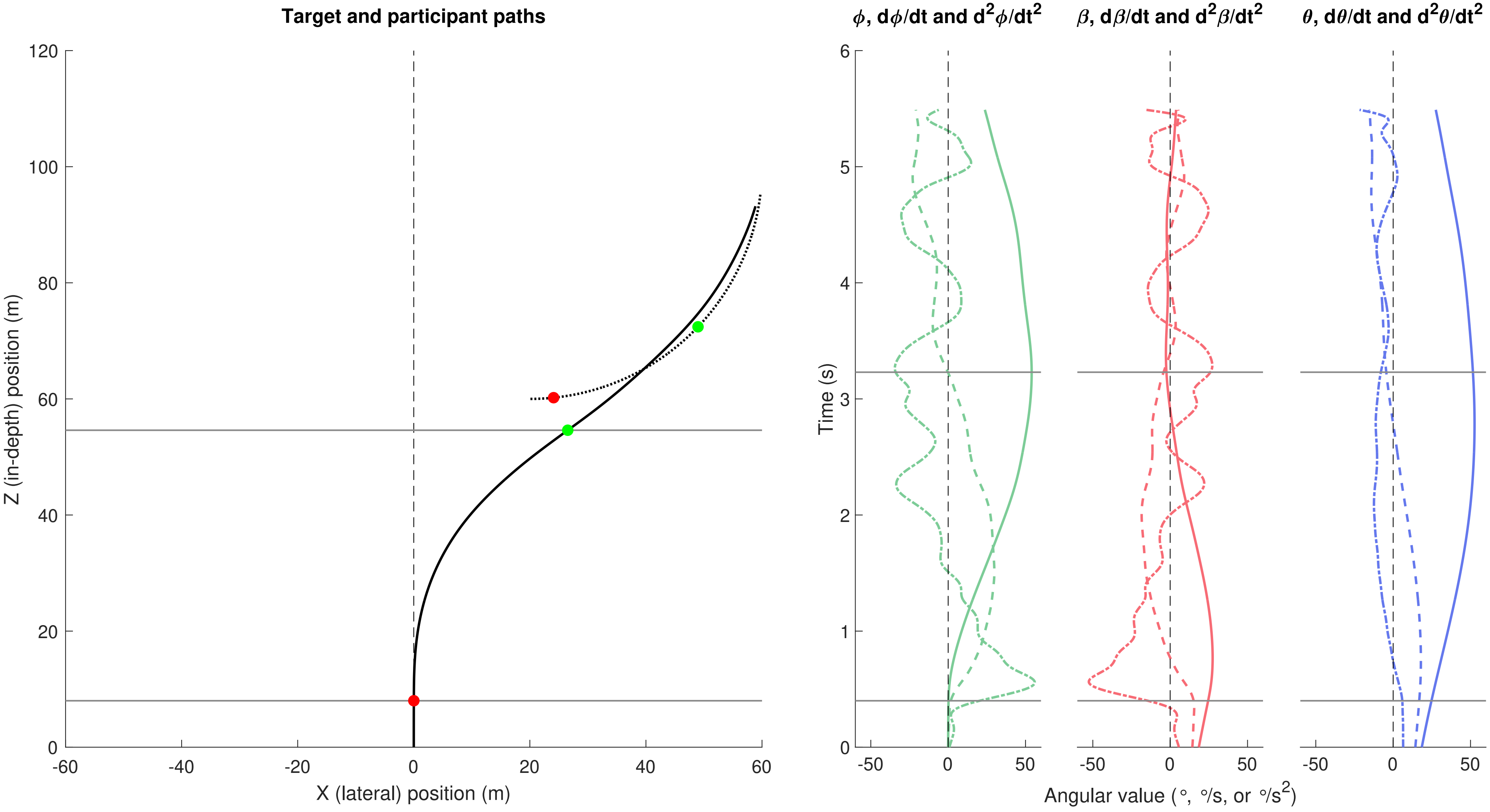

Participant 6

P6/B1
S20/R40-IN

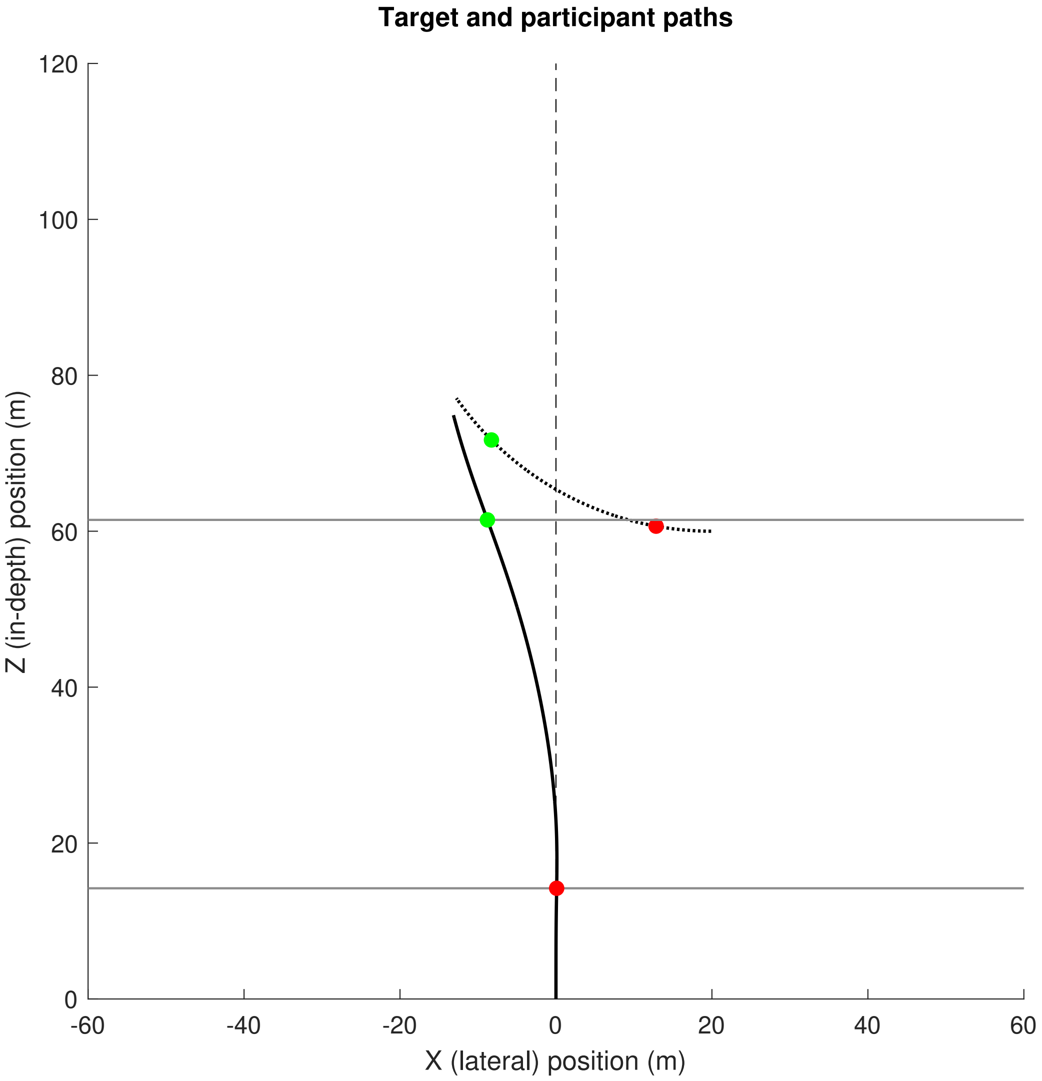

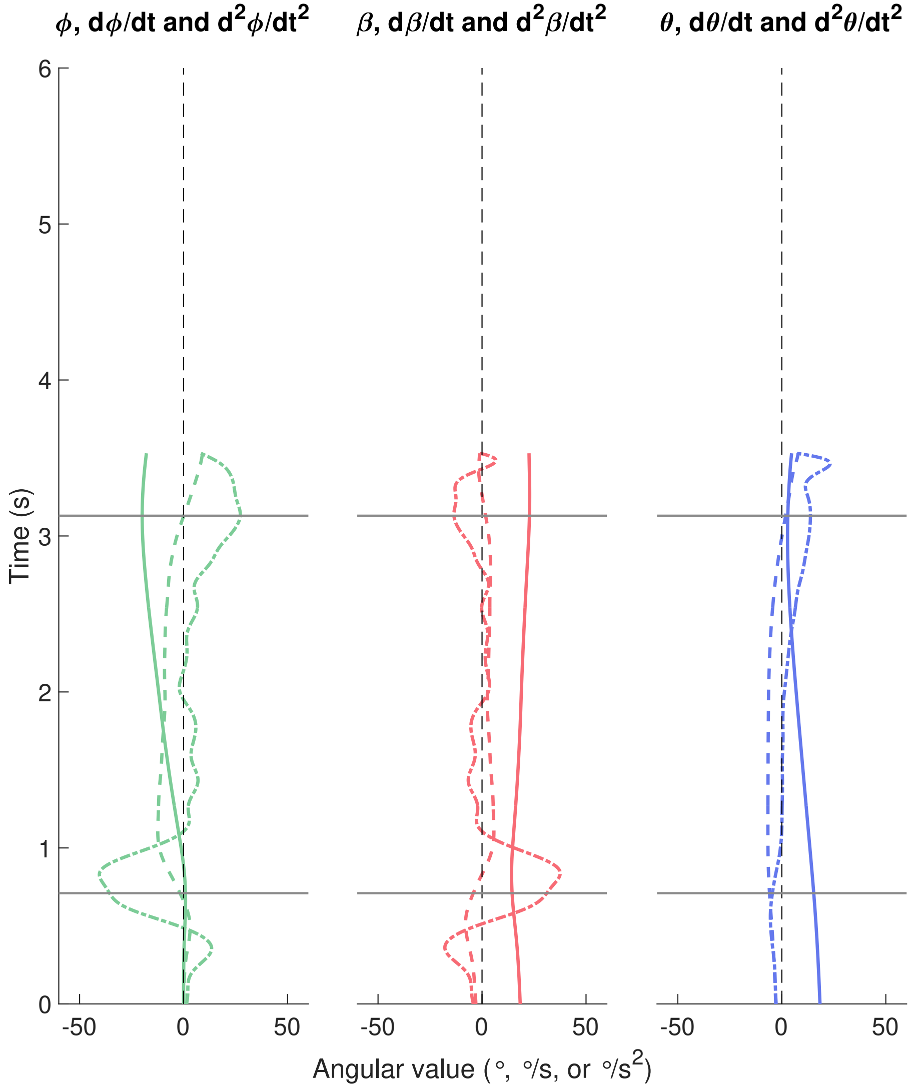

P6/B2
S20/R20-IN

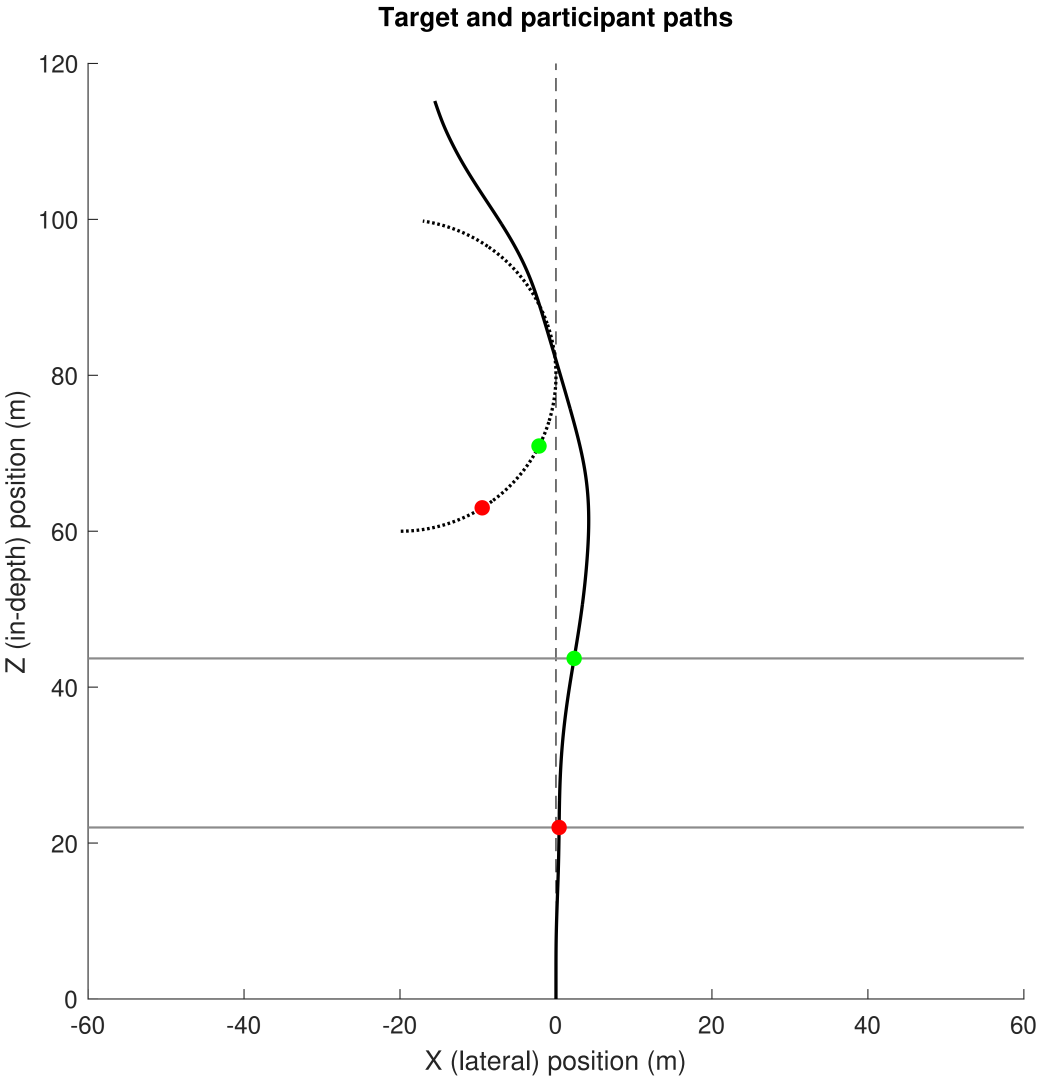

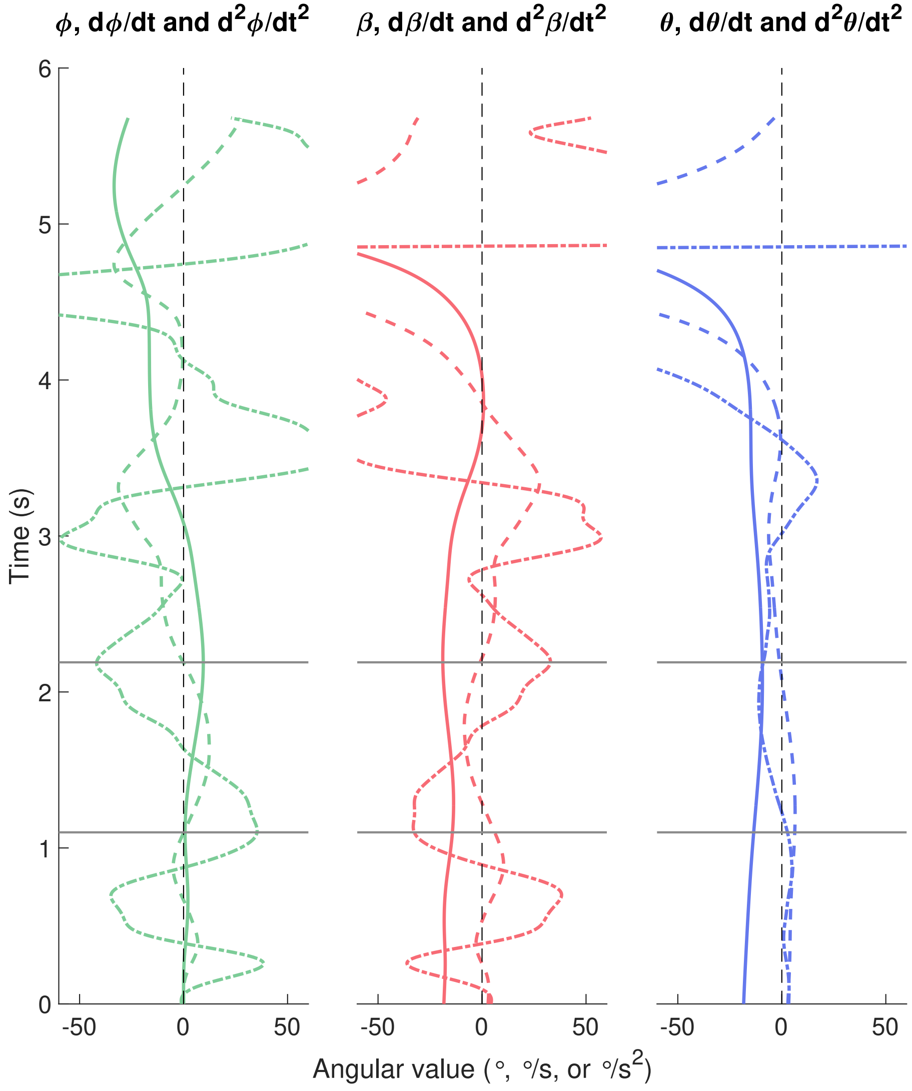

P6/B2
S20/R20-OUT

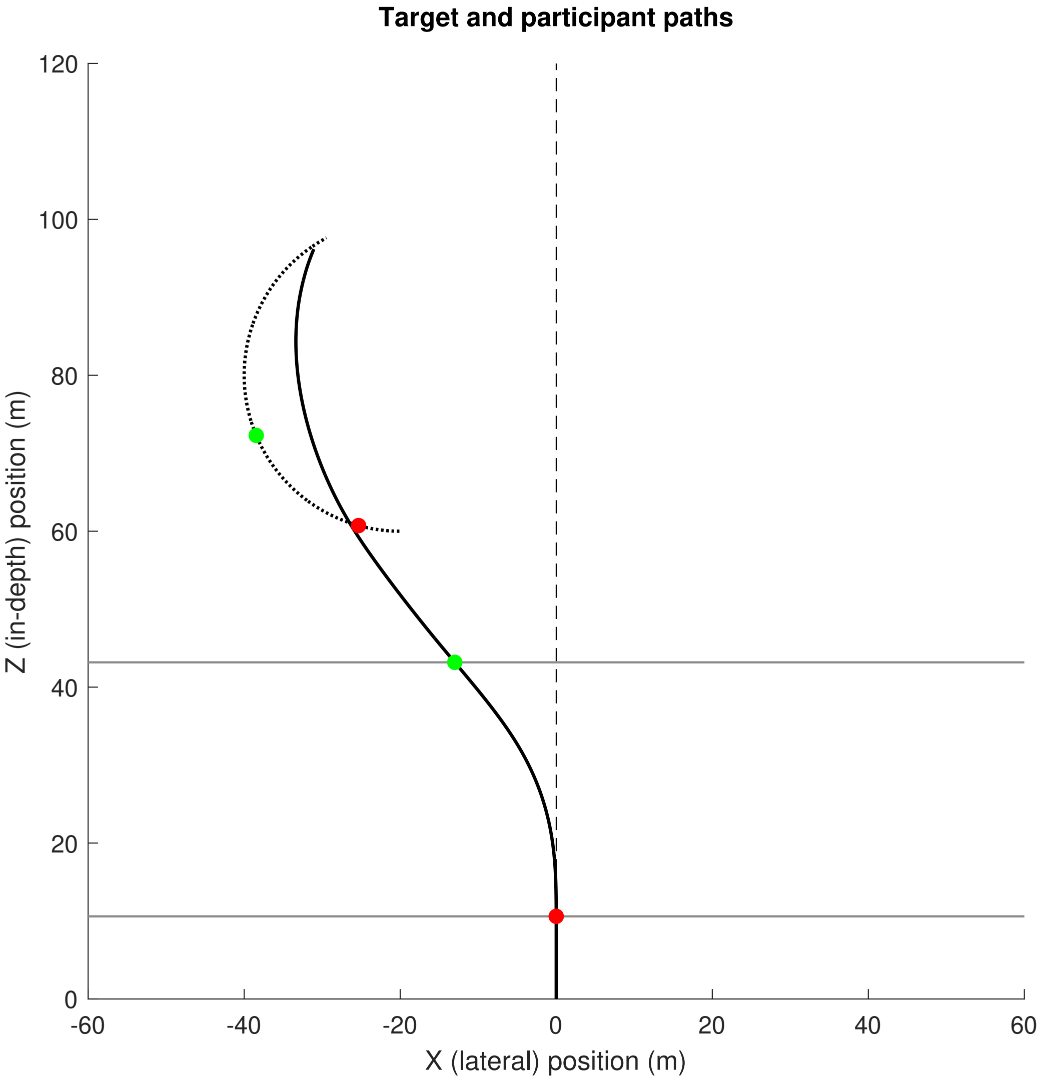

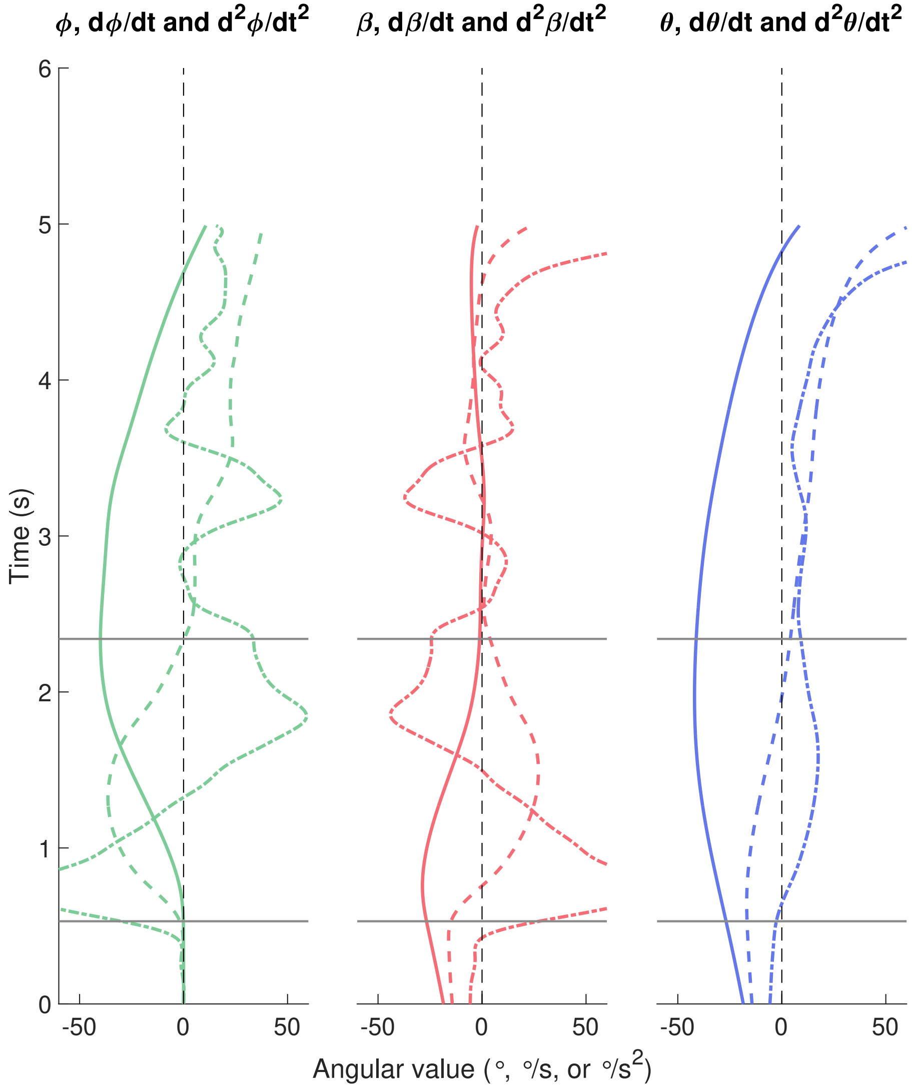

P6/B2
S20/R40-IN

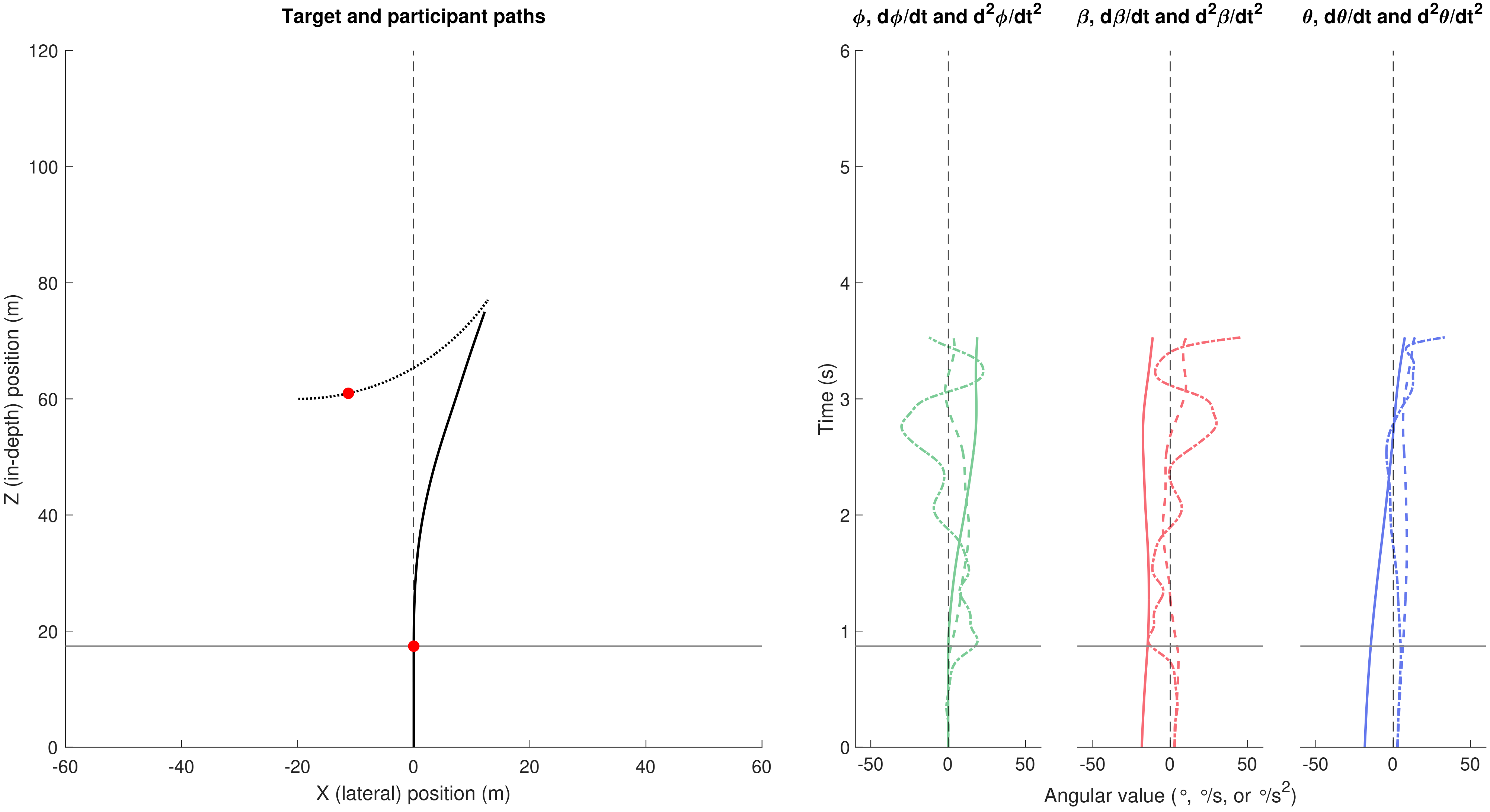

P6/B2
S20/R40-OUT

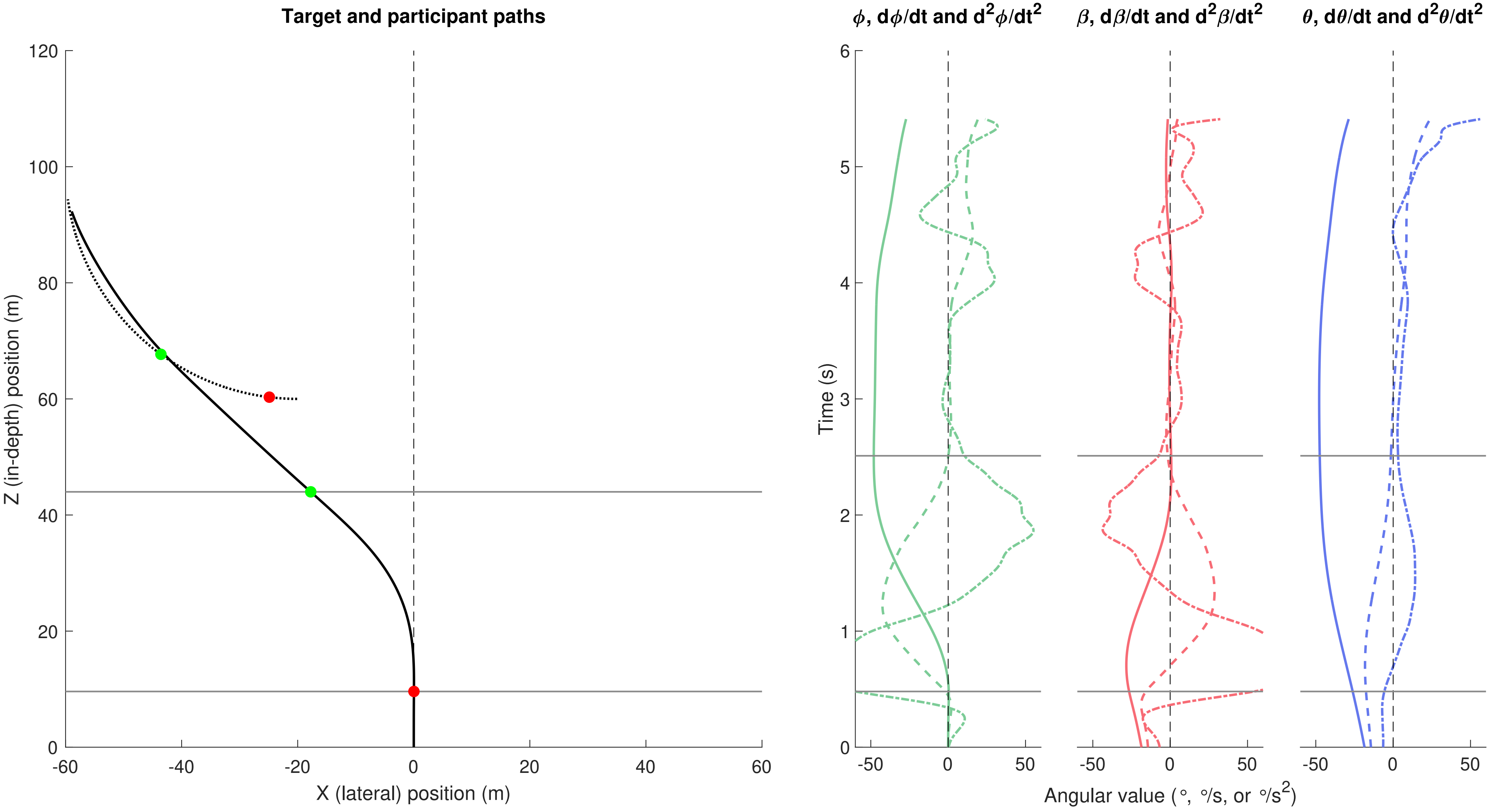

P6/B2
S10/R20-IN

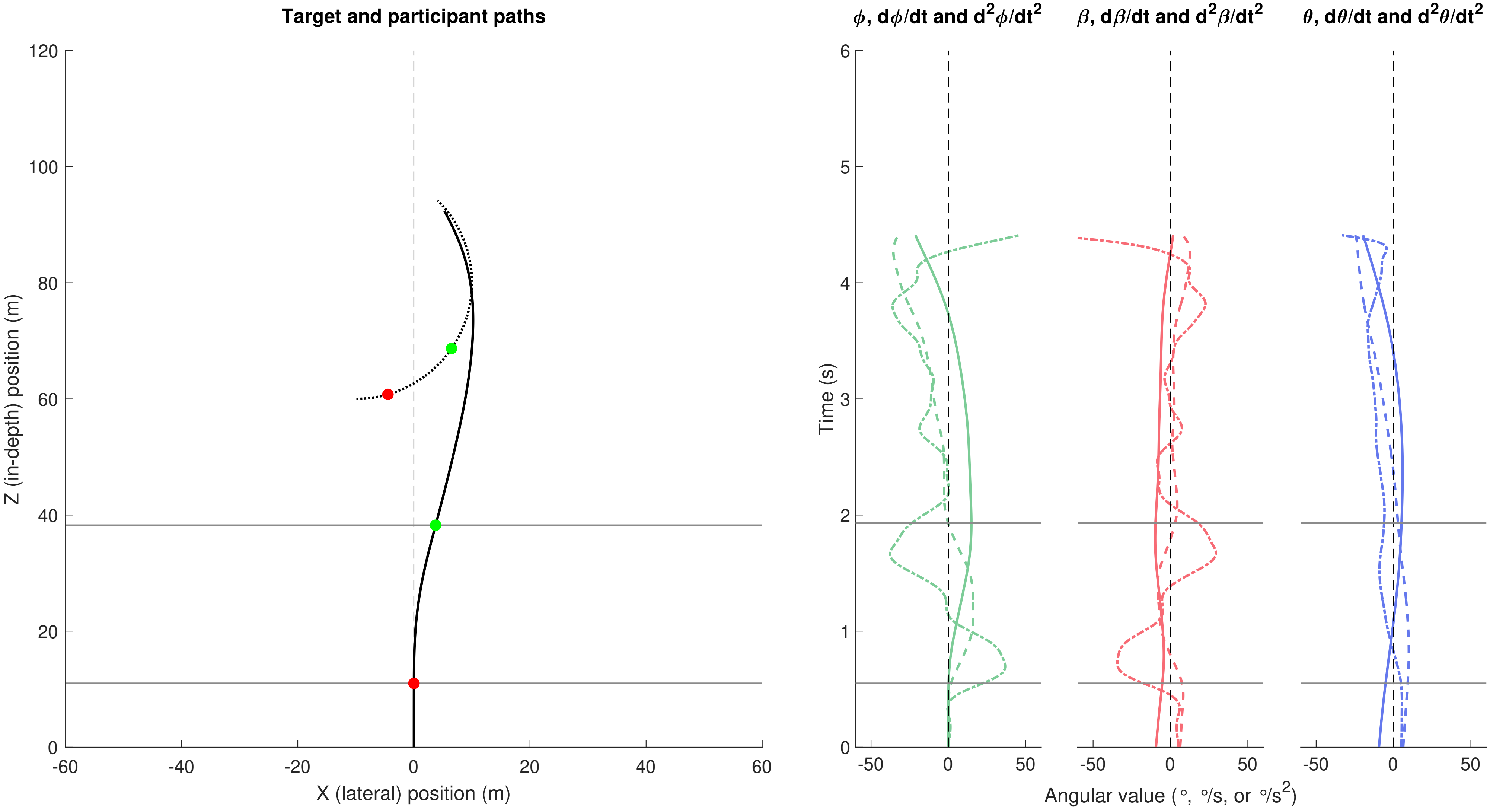

P6/B2
S10/R20-OUT

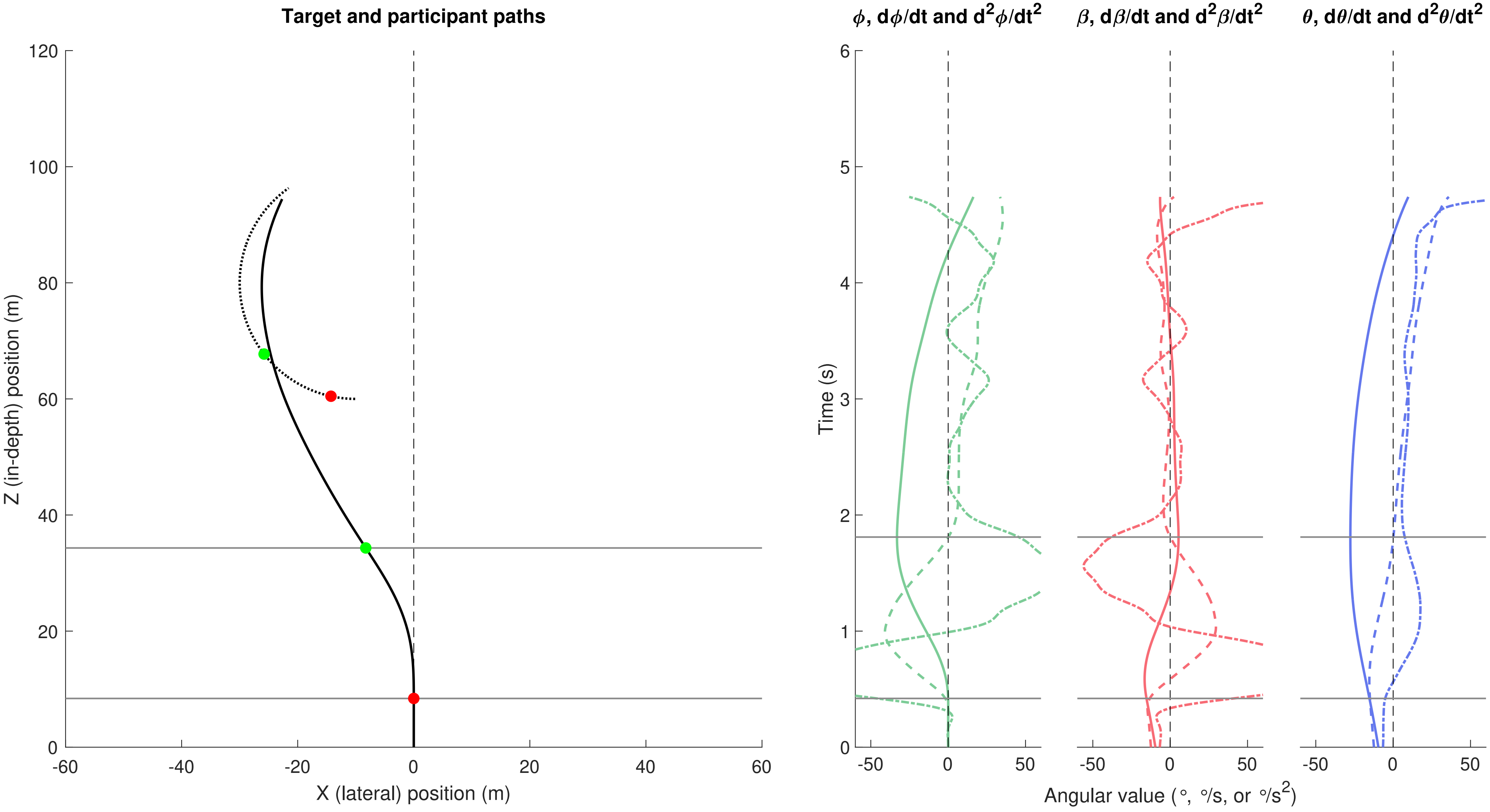

P6/B2
S10/R40-IN

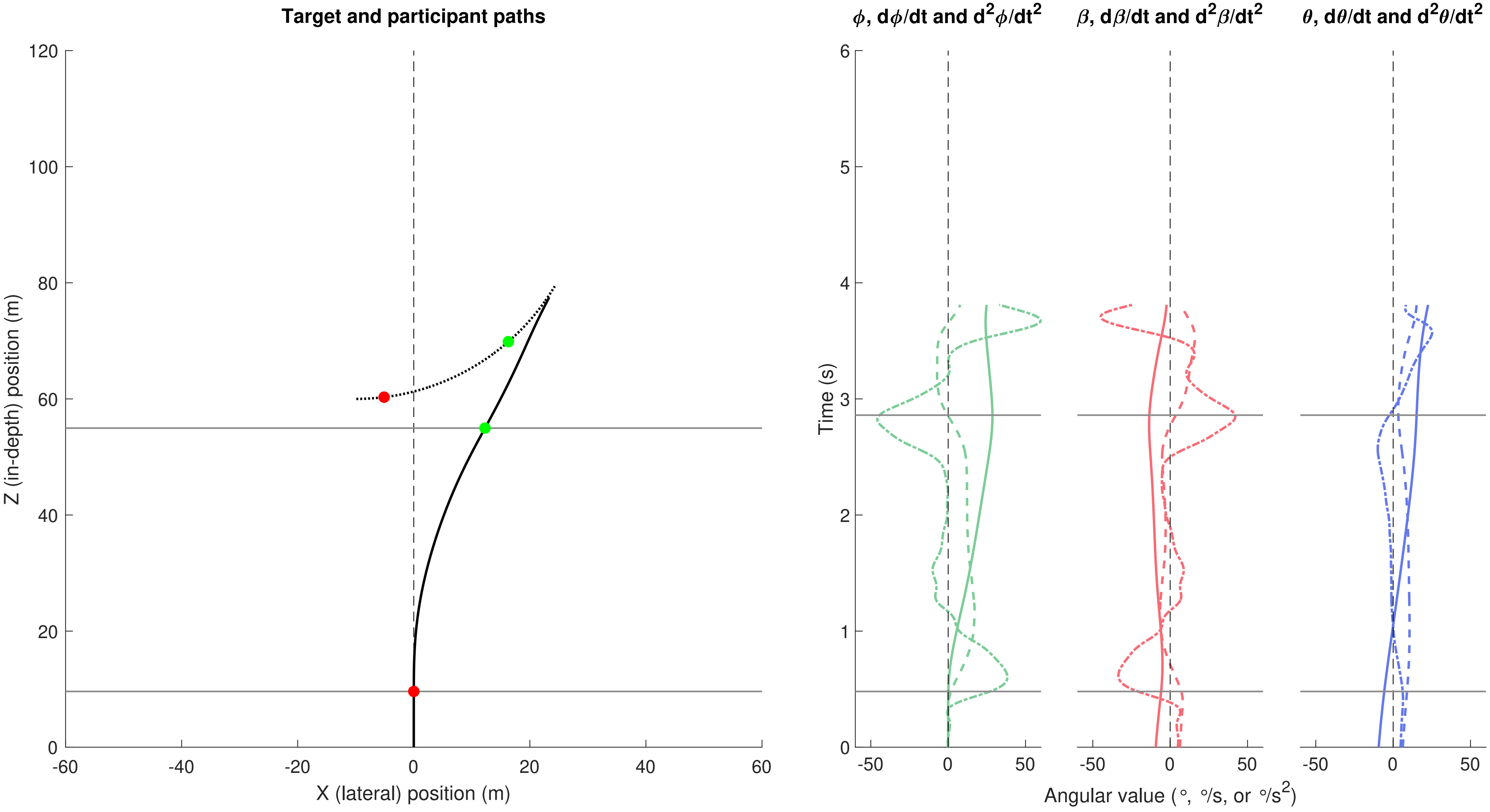

P6/B2
S10/R40-OUT

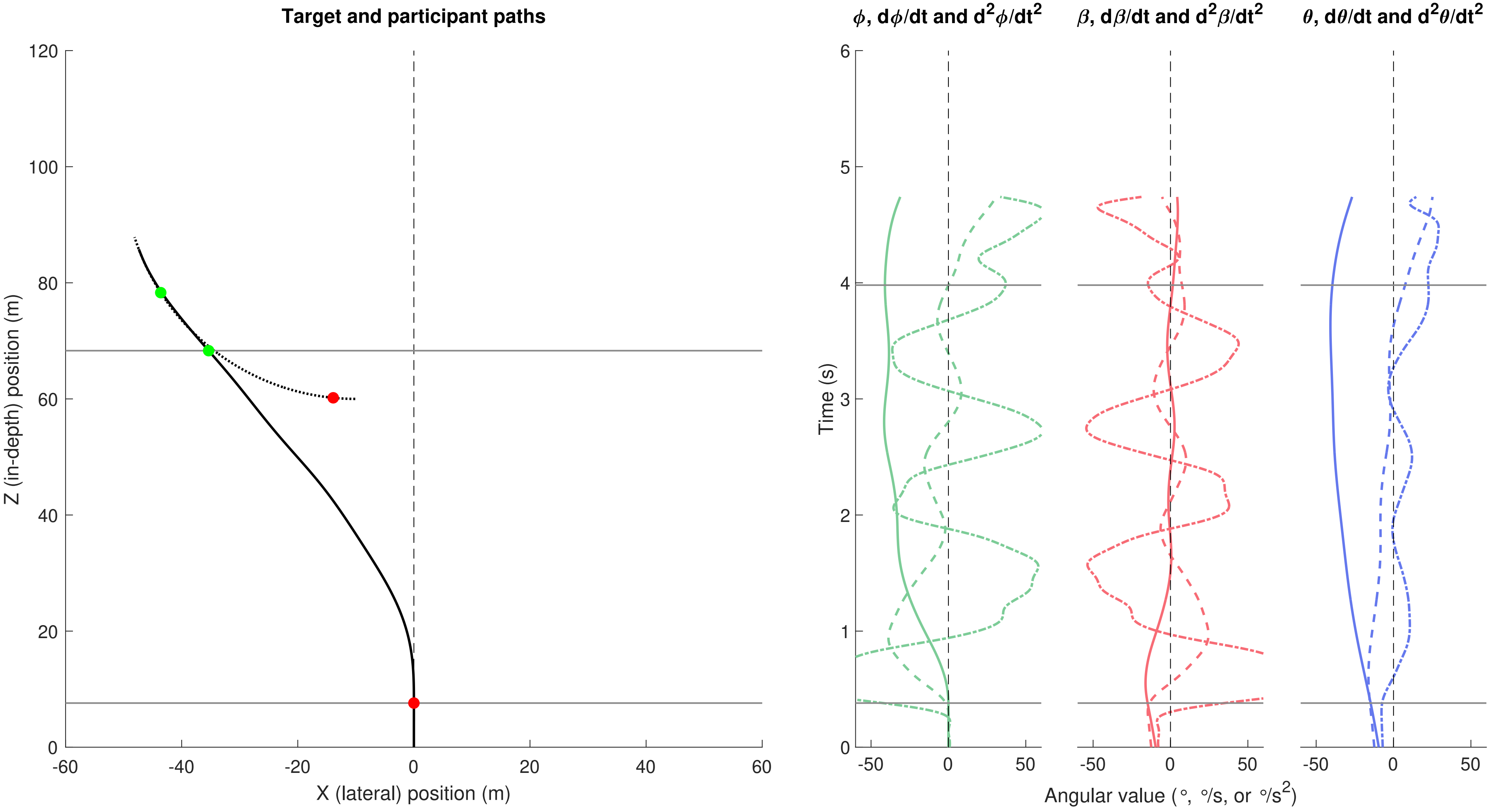

P6/B2
S0/R20-OUT

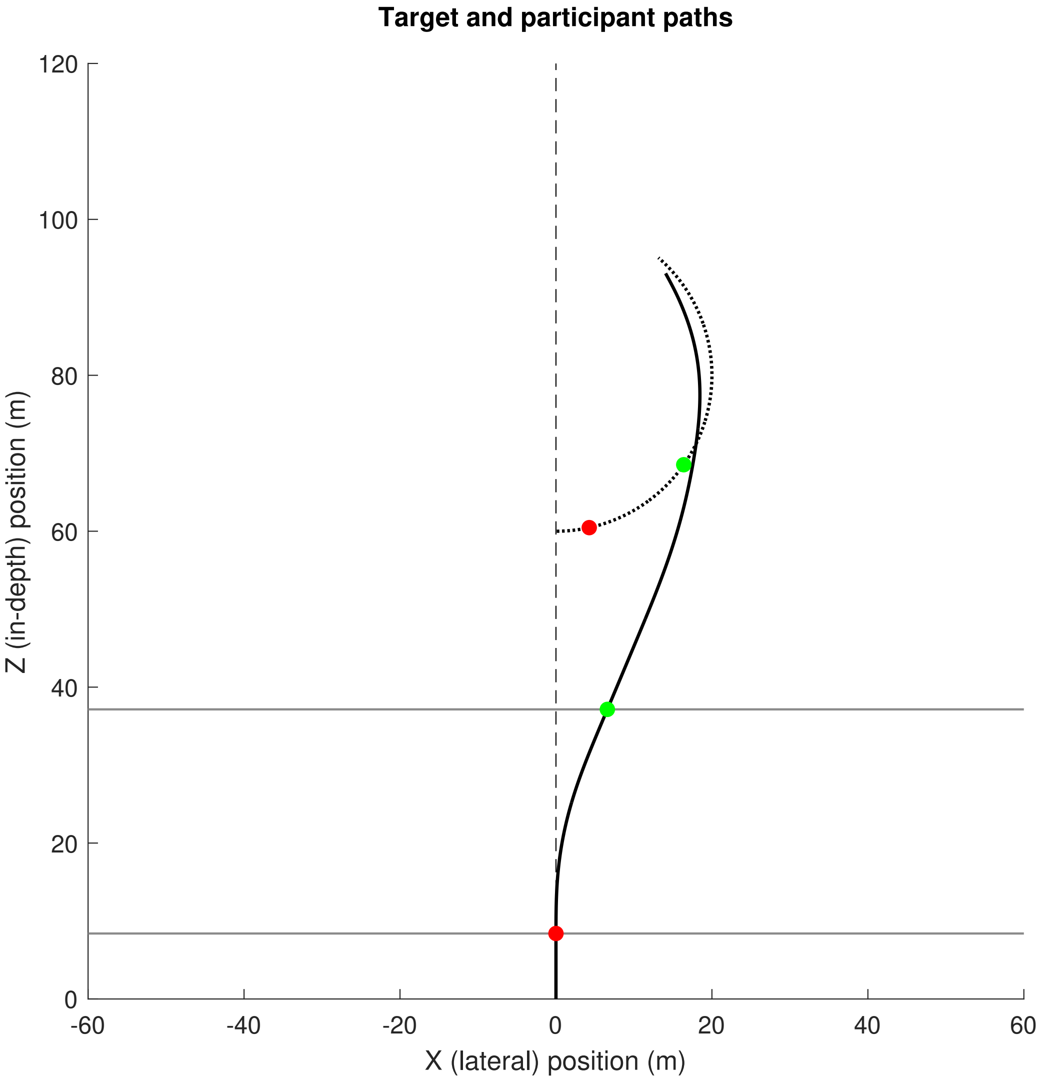

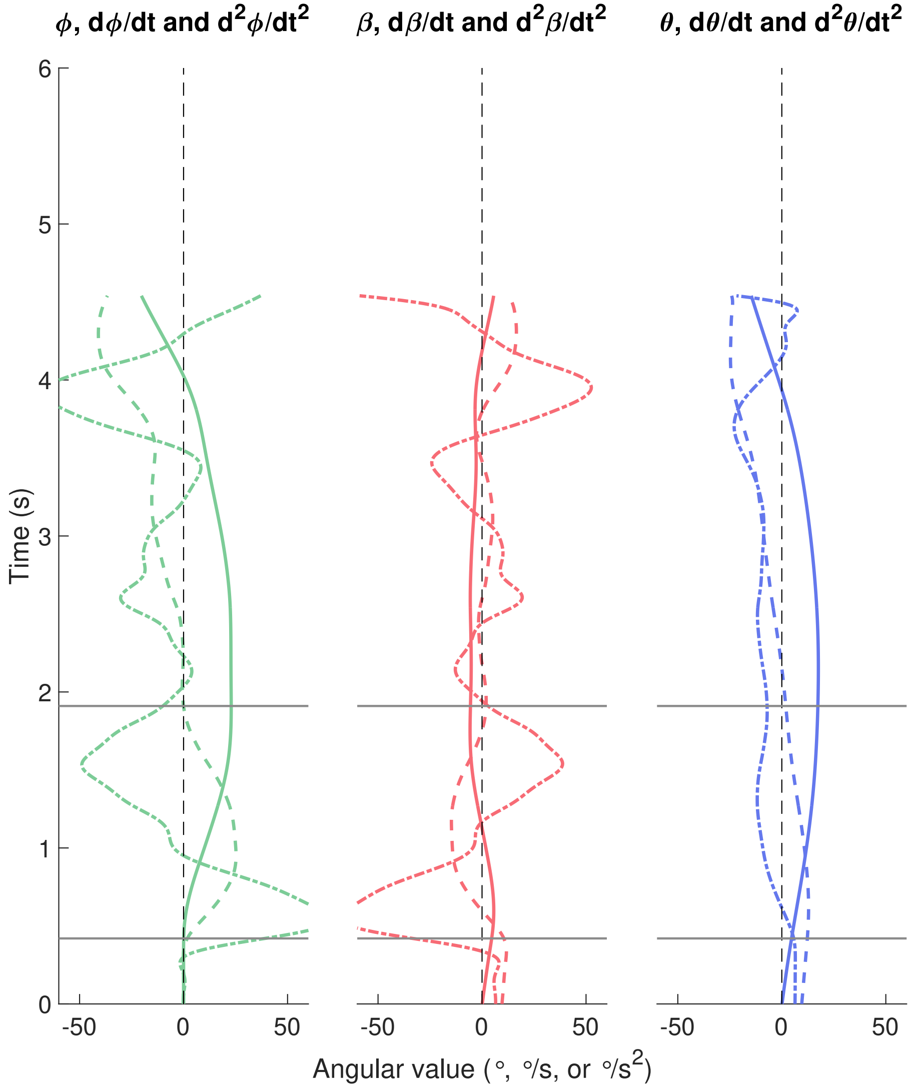

P6/B2
S0/R20-OUT

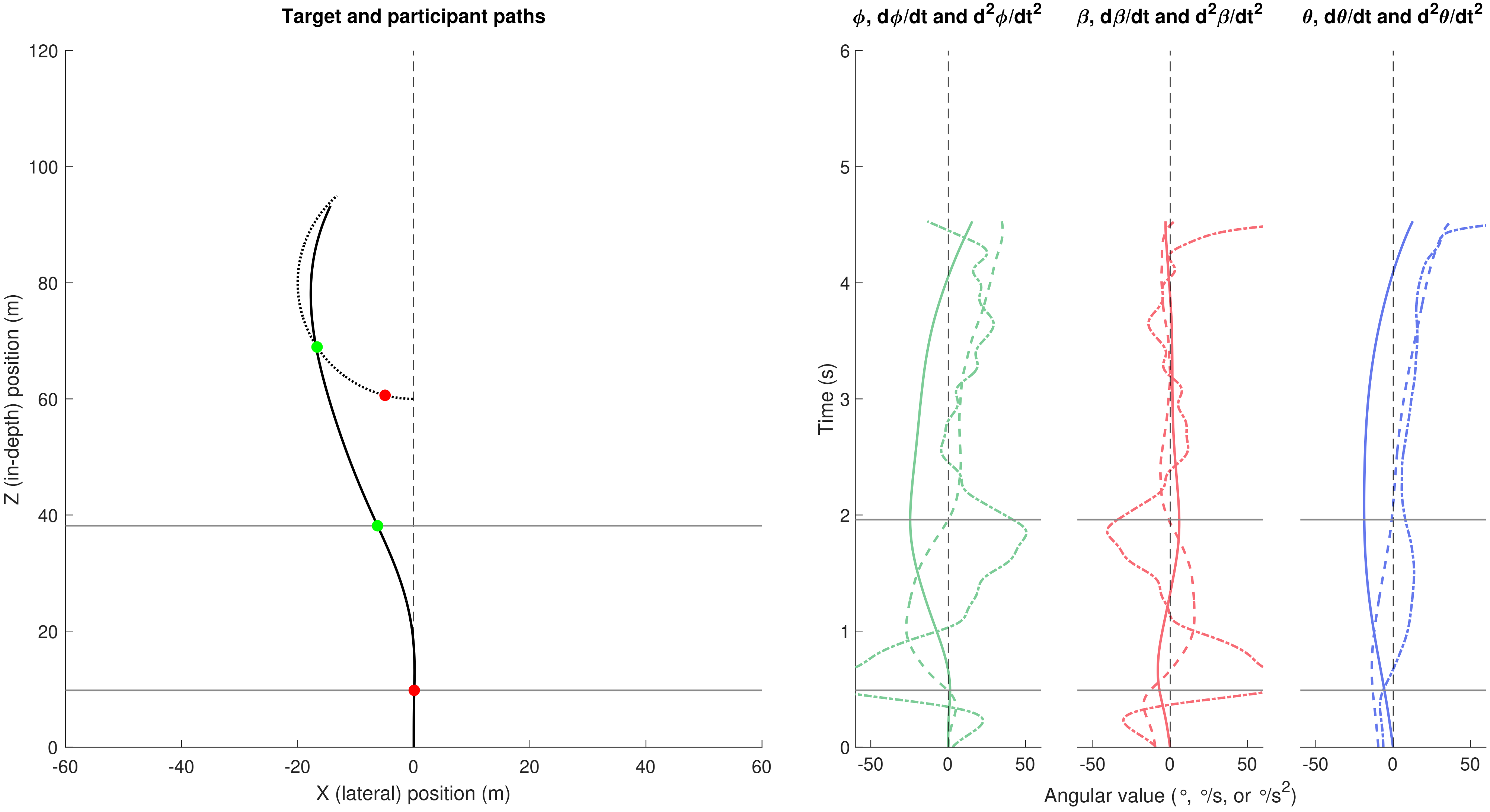

P6/B2
S0/R40-OUT

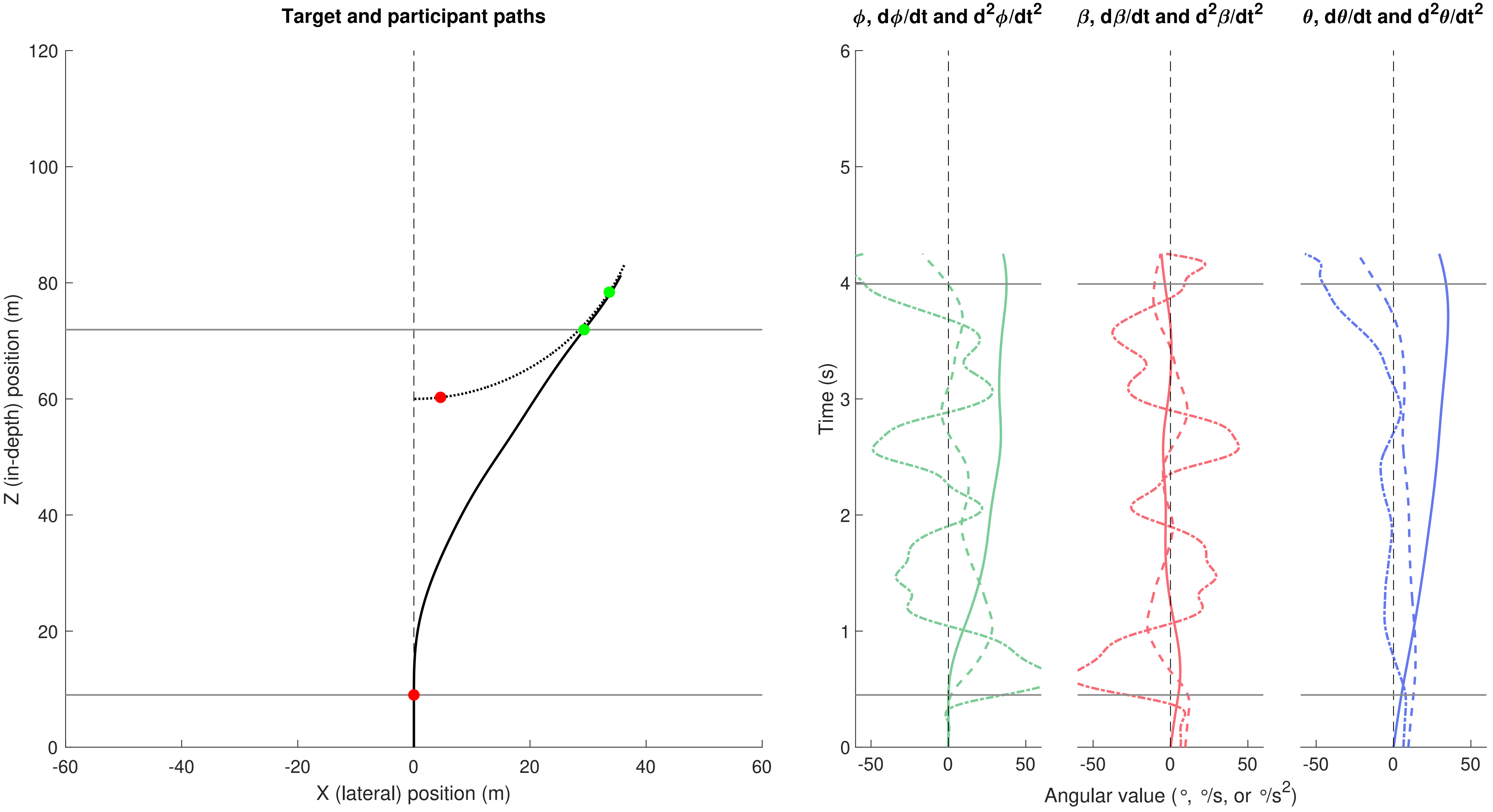

P6/B2
S0/R40-OUT

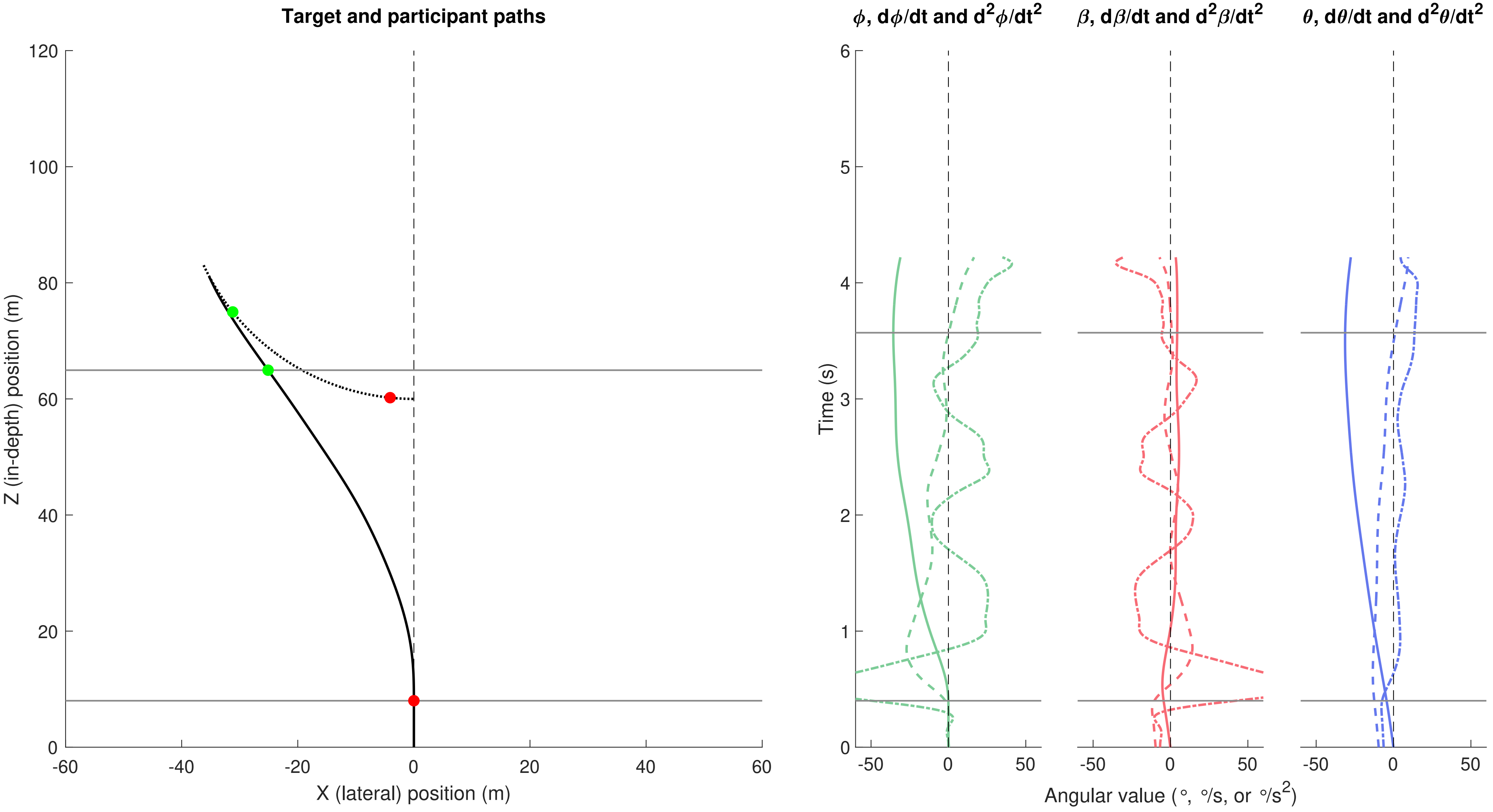

P6/B2
S10/R20-OUT

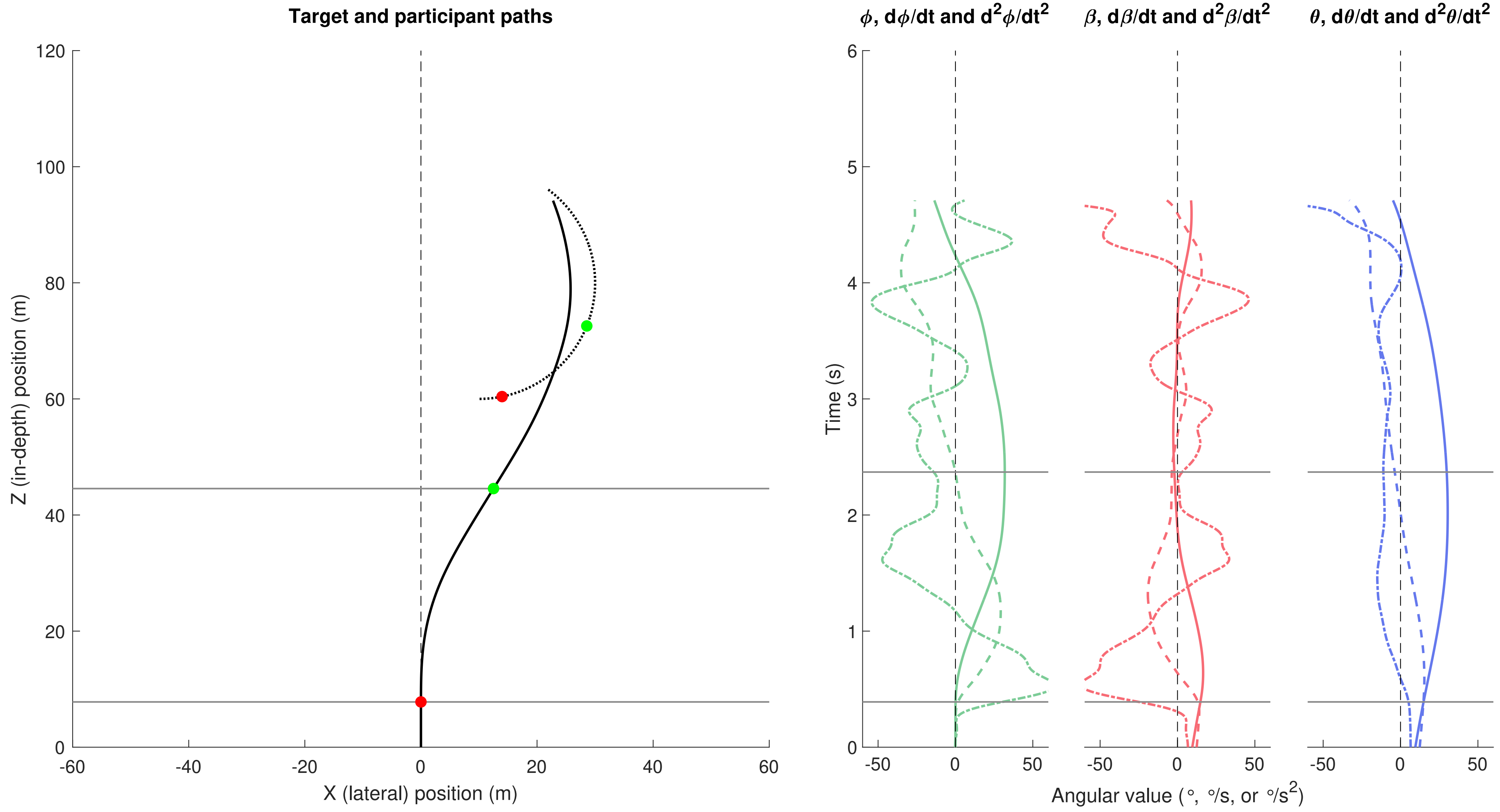

P6/B2
S10/R20-IN

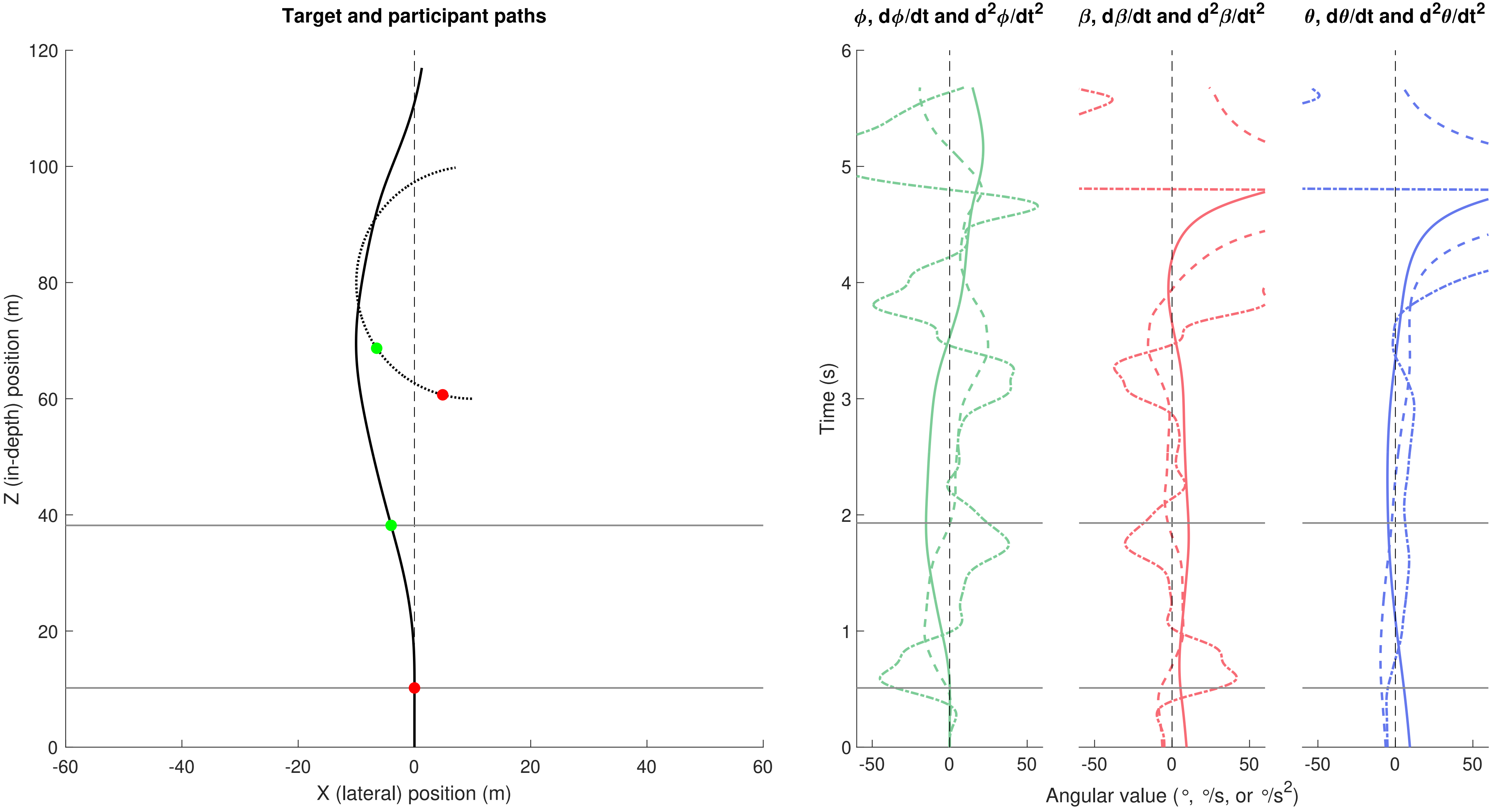

P6/B2
S10/R40-OUT

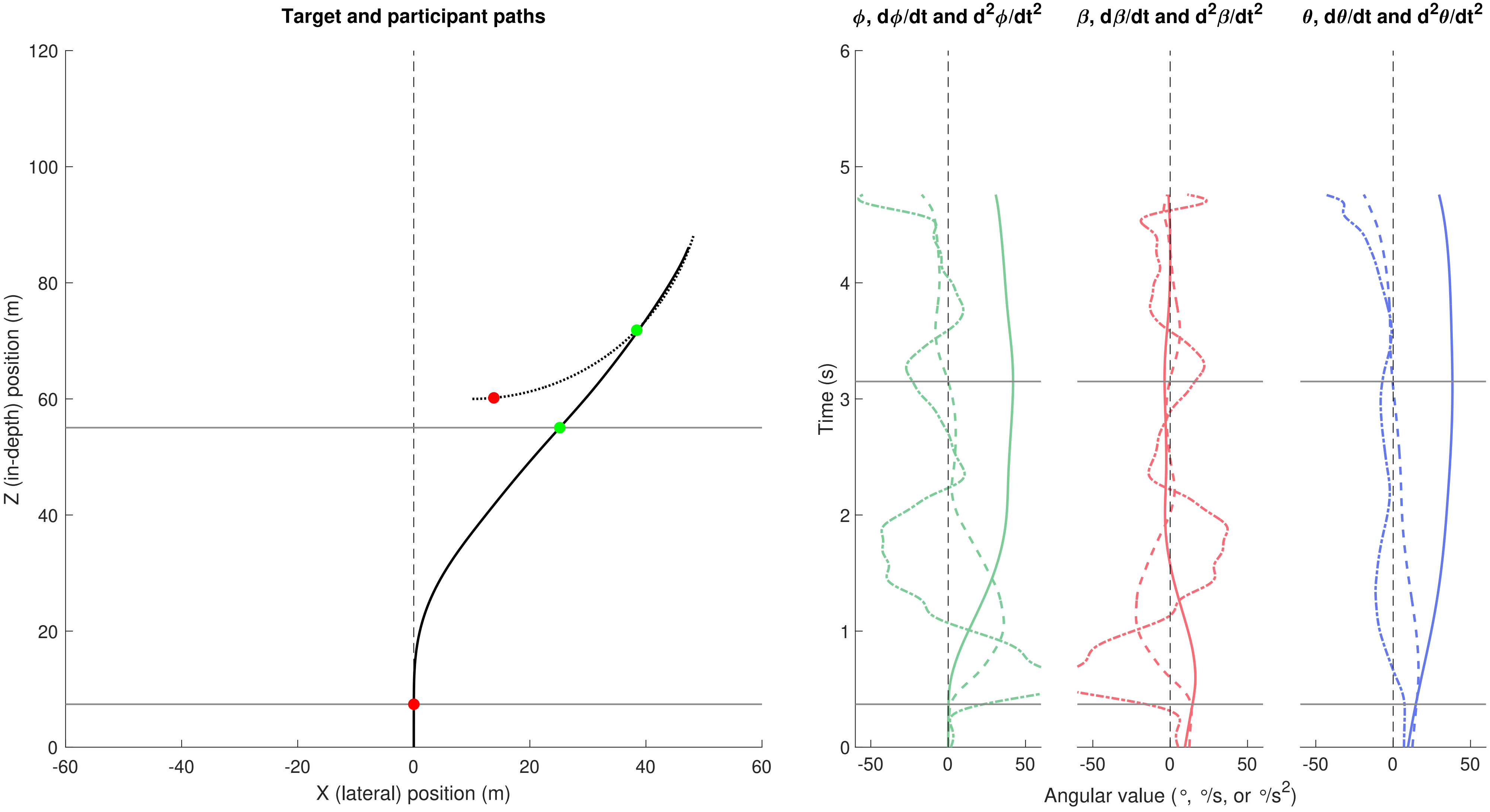

P6/B2
S10/R40-IN

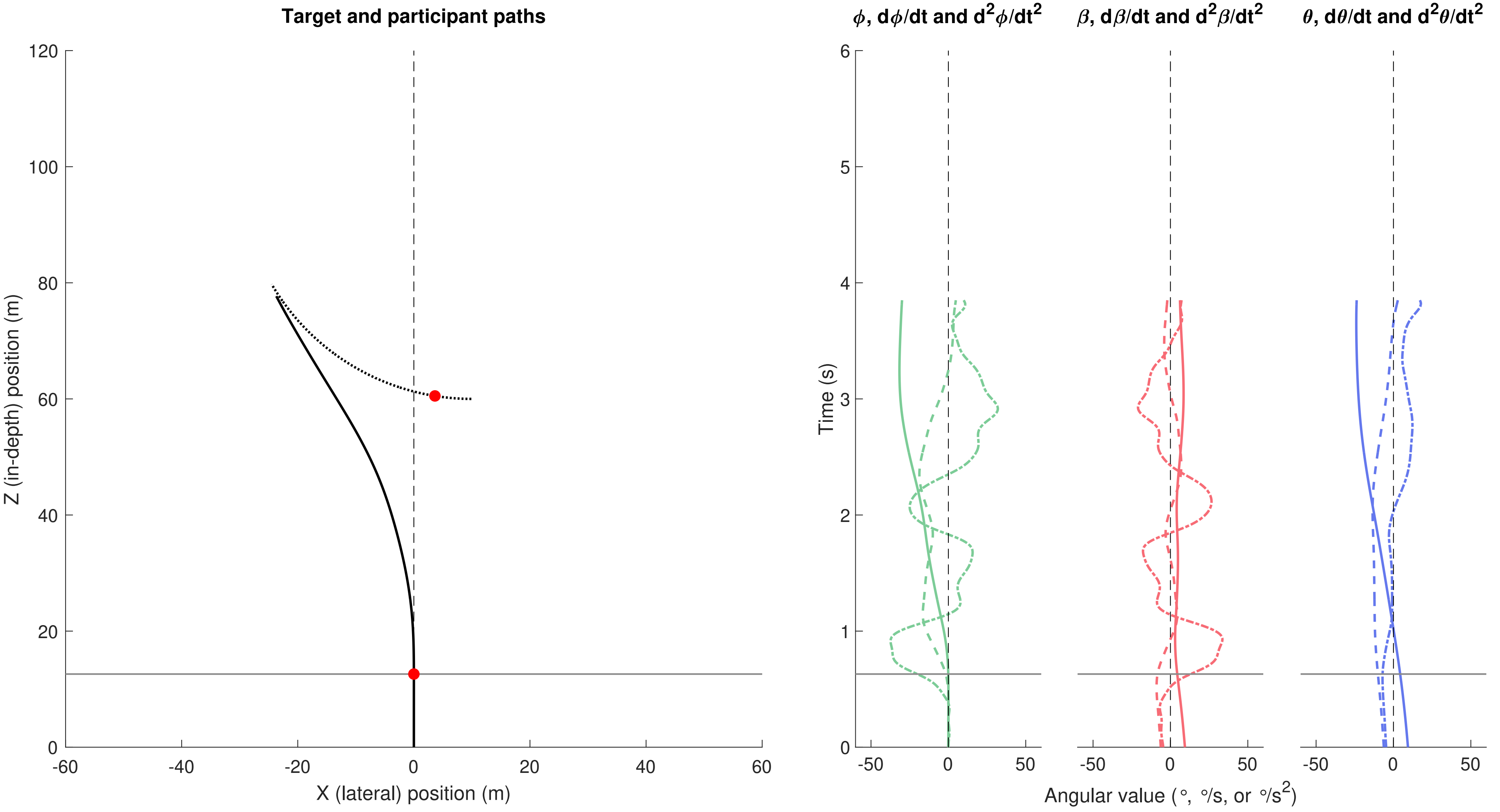

P6/B2
S20/R20-OUT

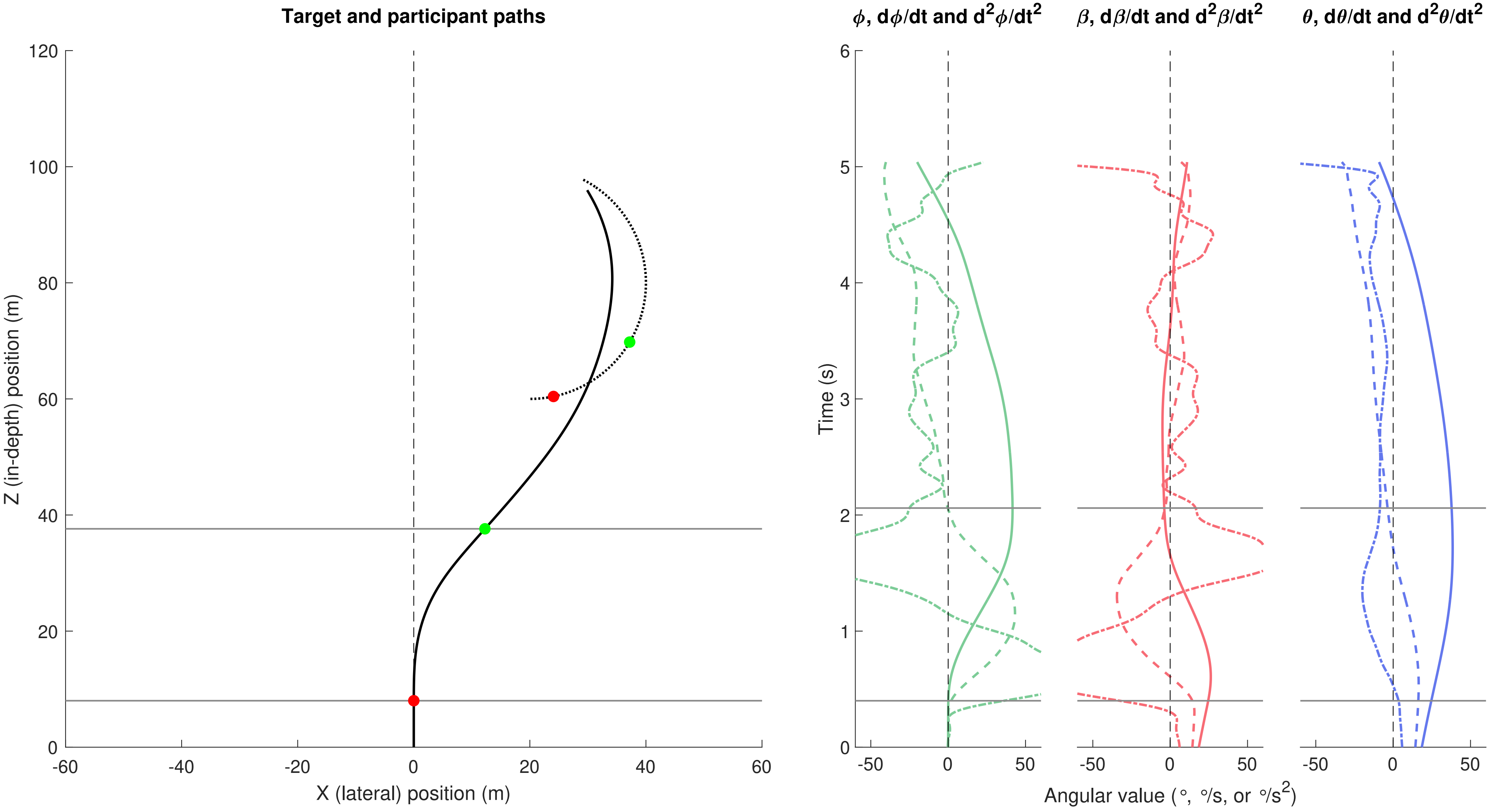

P6/B2
S20/R20-IN

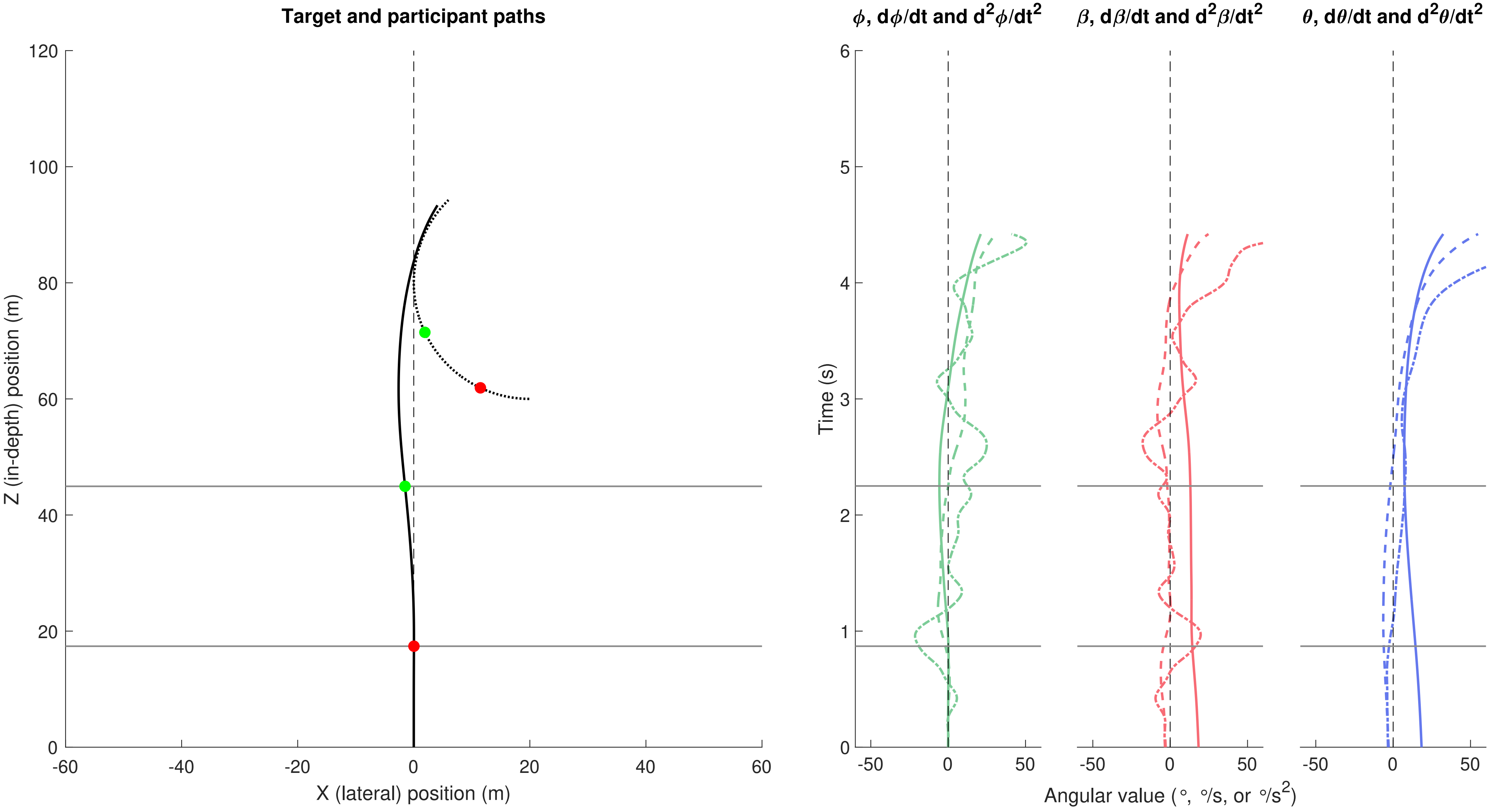

P6/B2
S20/R40-OUT

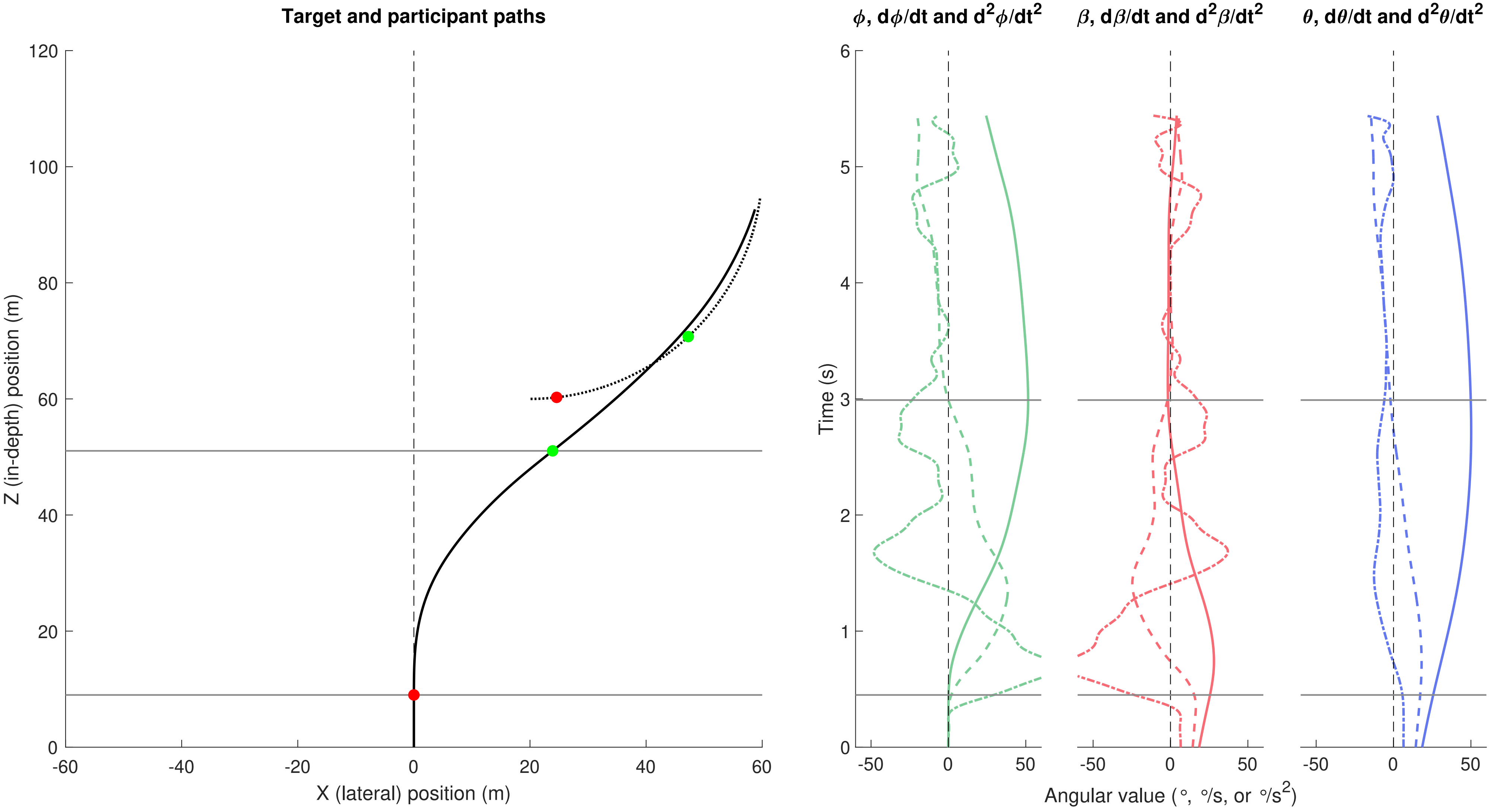

P6/B2
S20/R40-IN

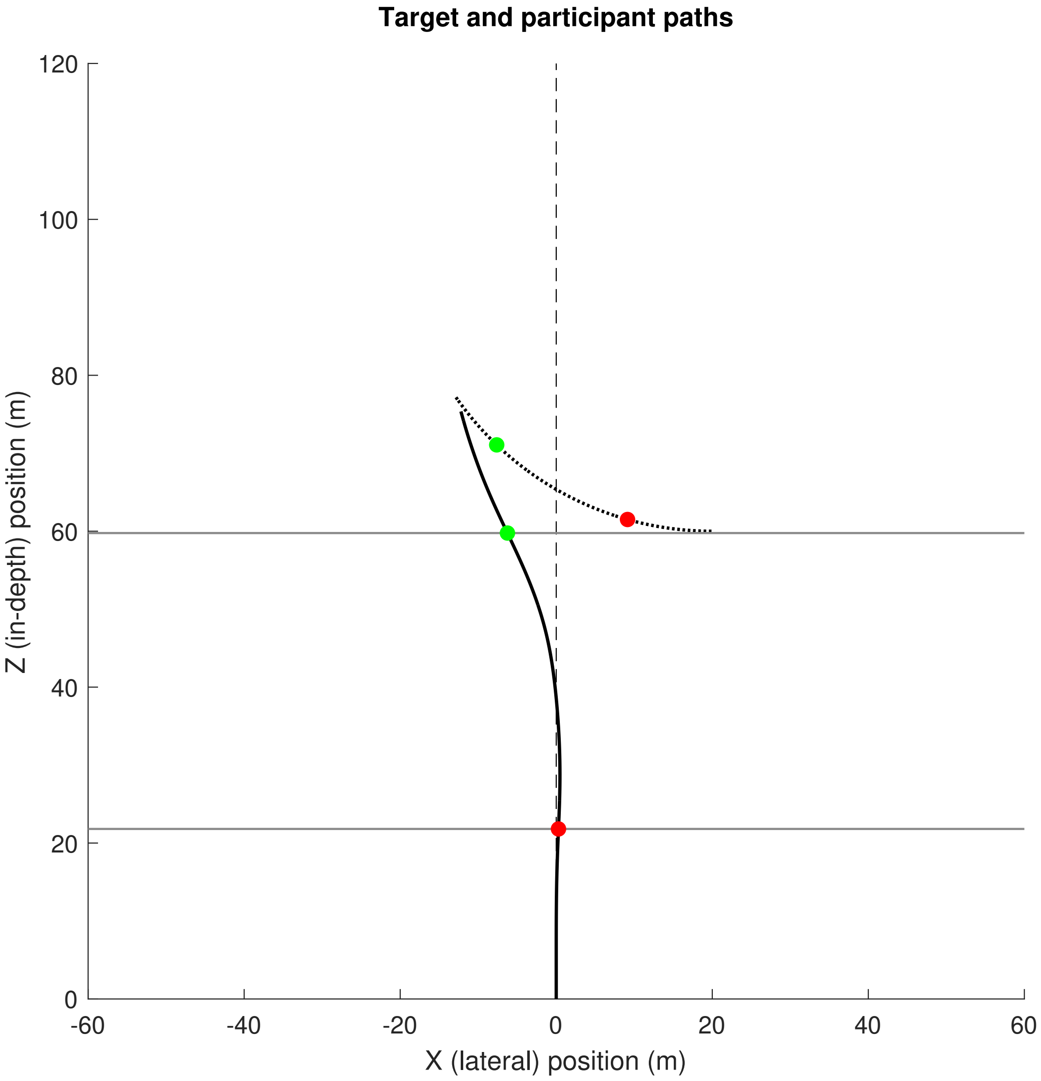

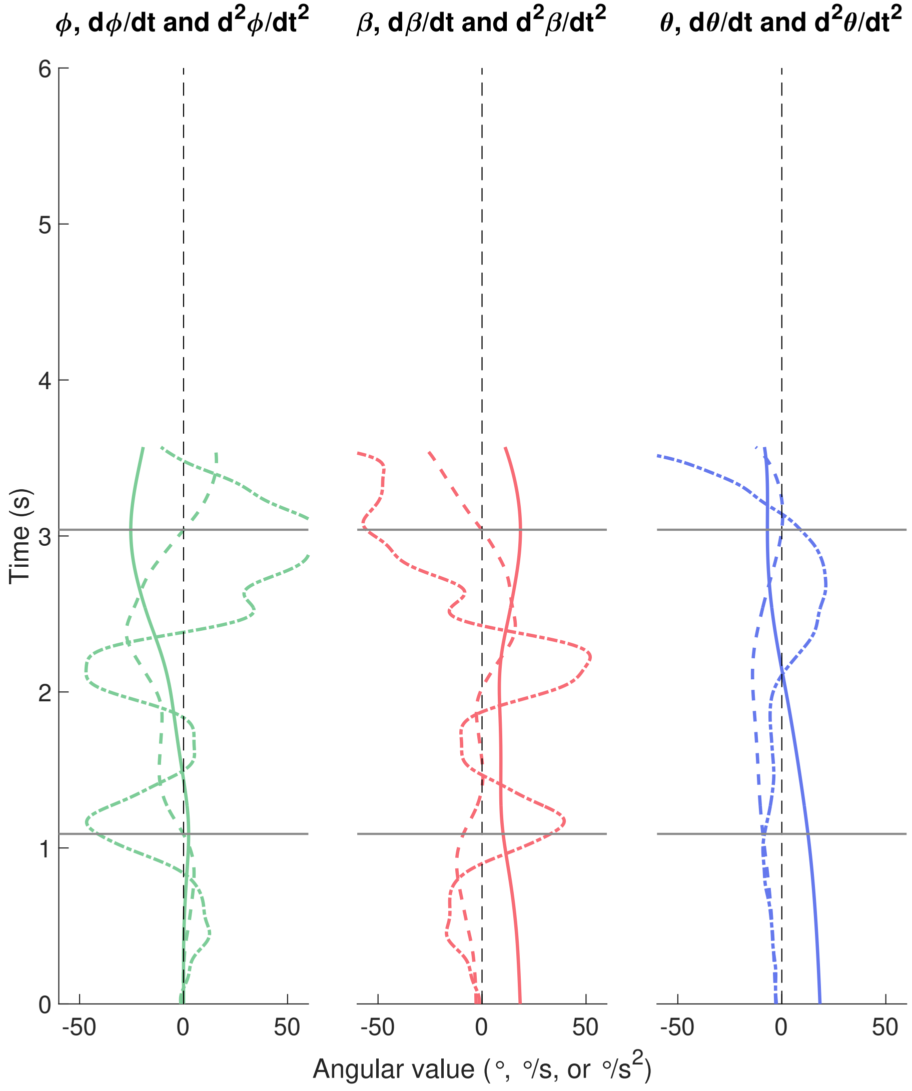

P6/B3
S20/R20-IN

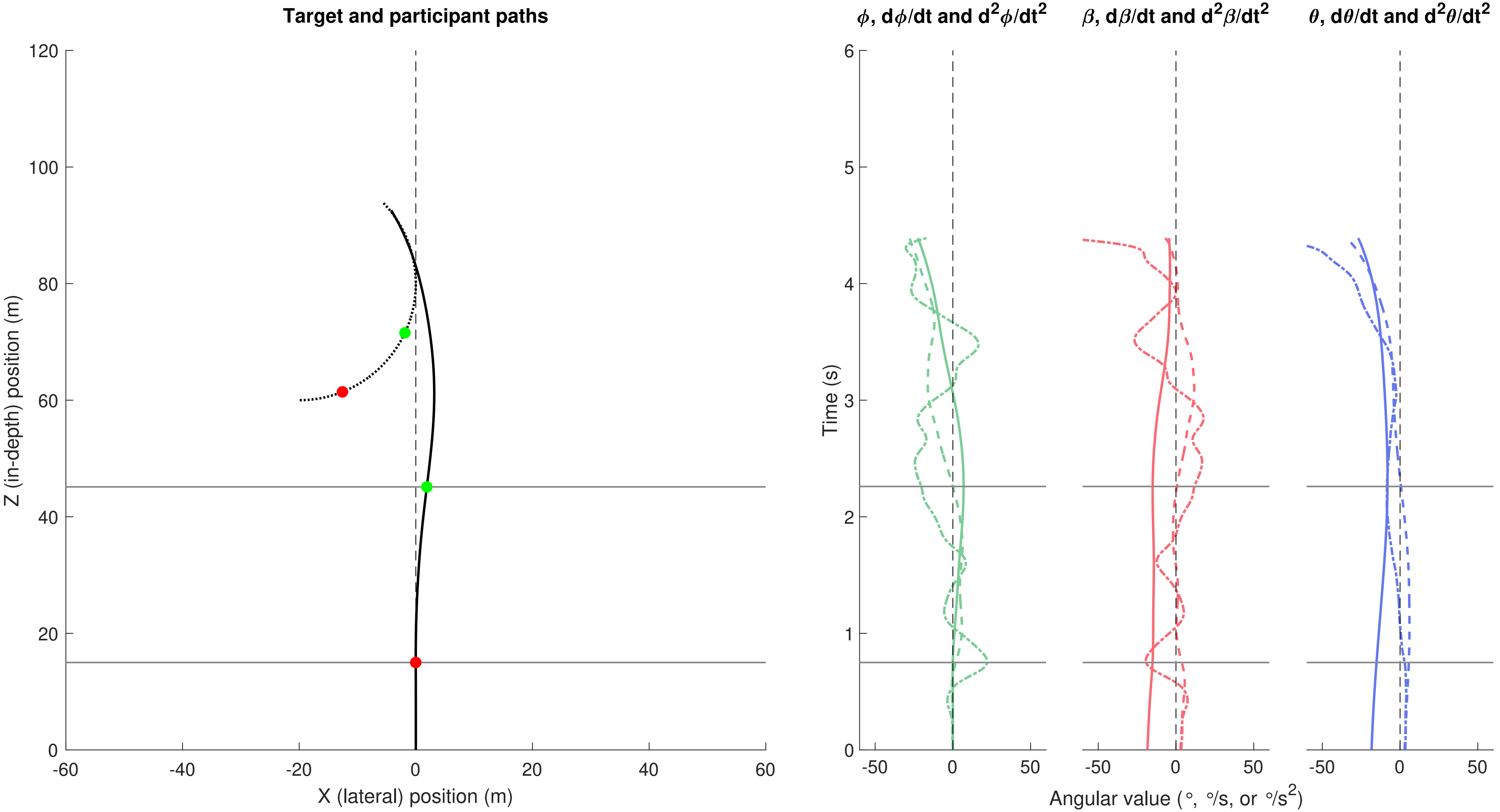

P6/B3
S20/R20-OUT

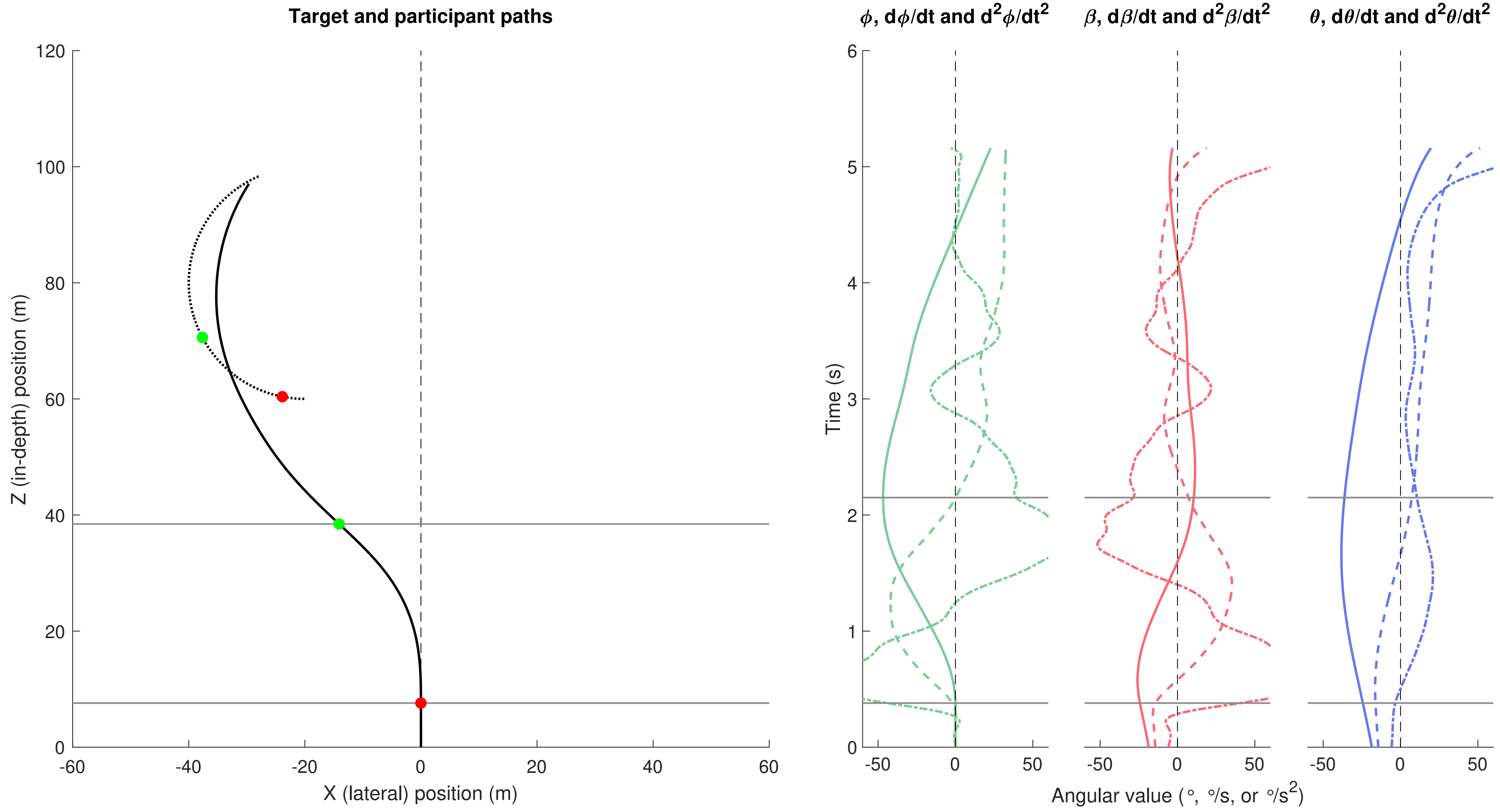

P6/B3
S20/R40-IN

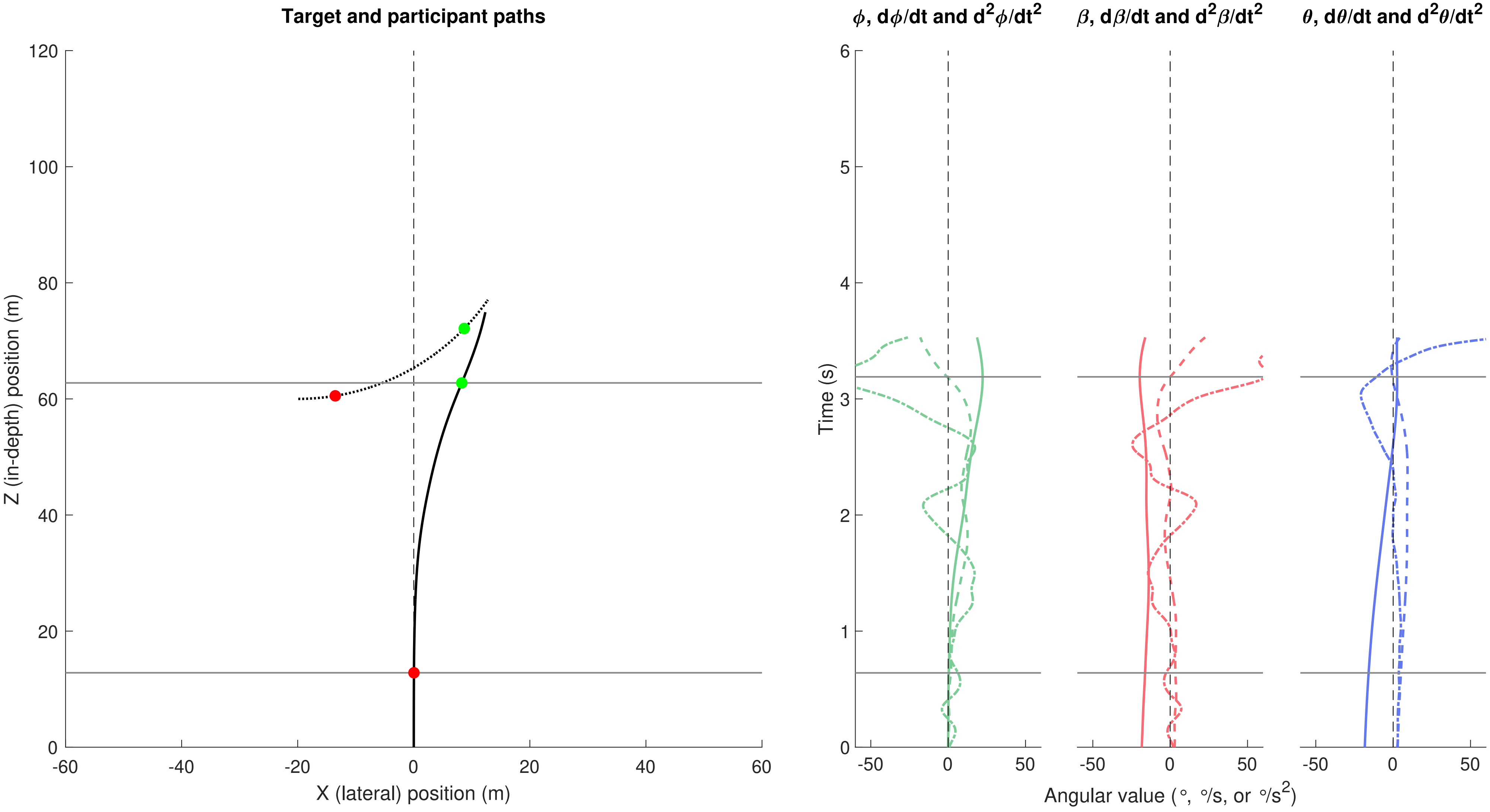

P6/B3
S20/R40-OUT

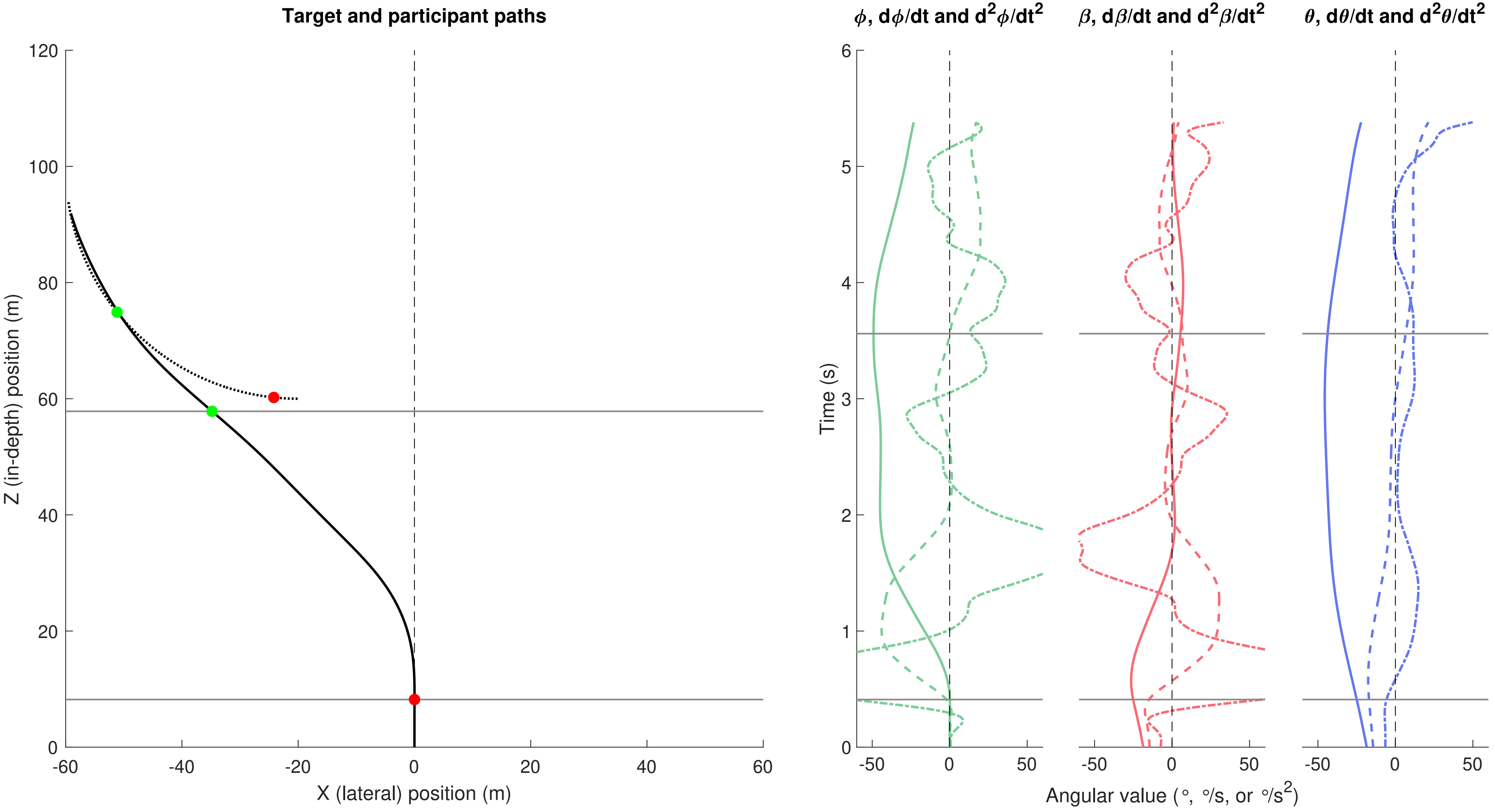

P6/B3
S10/R20-IN

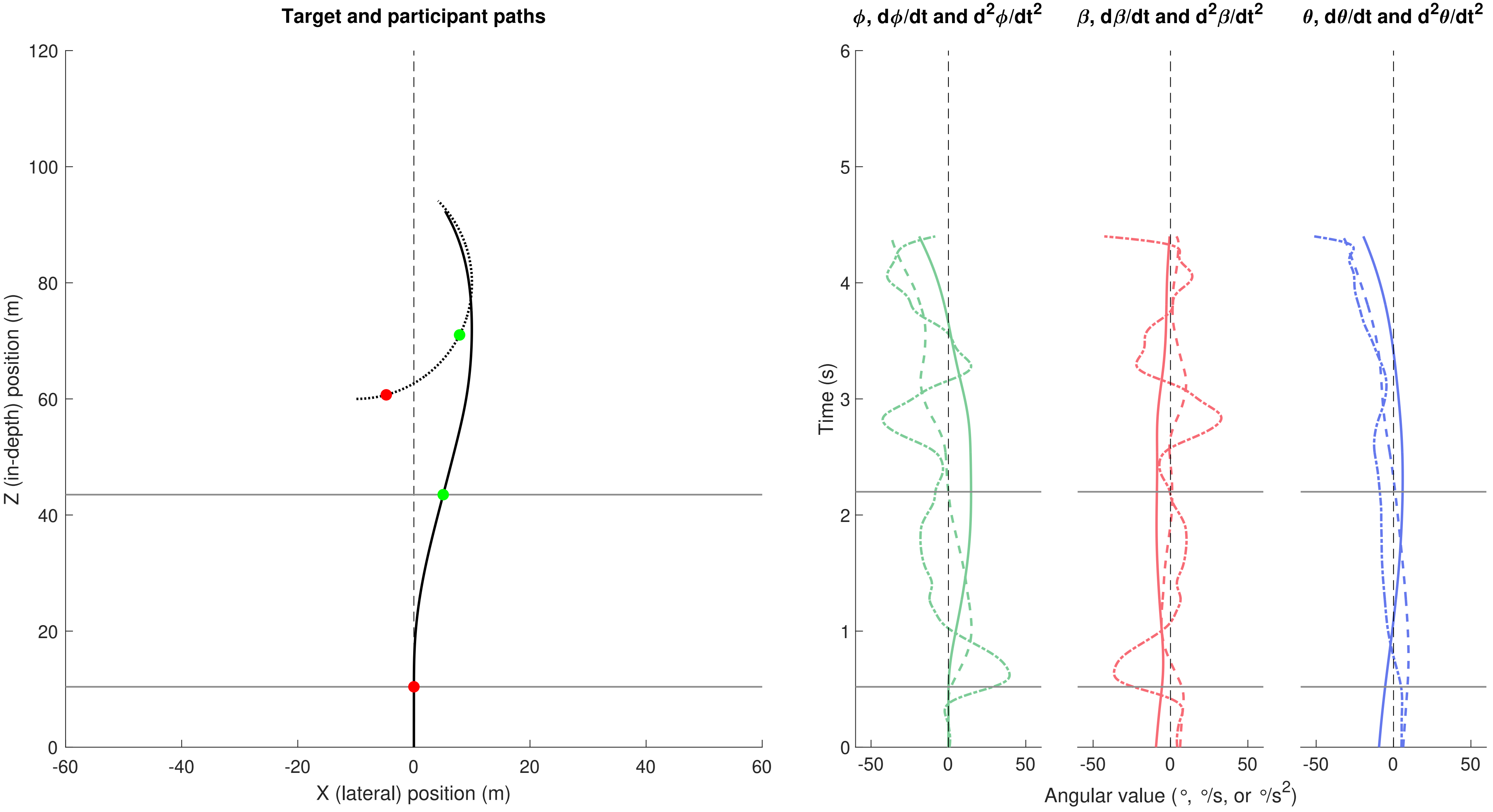

P6/B3
S10/R20-OUT

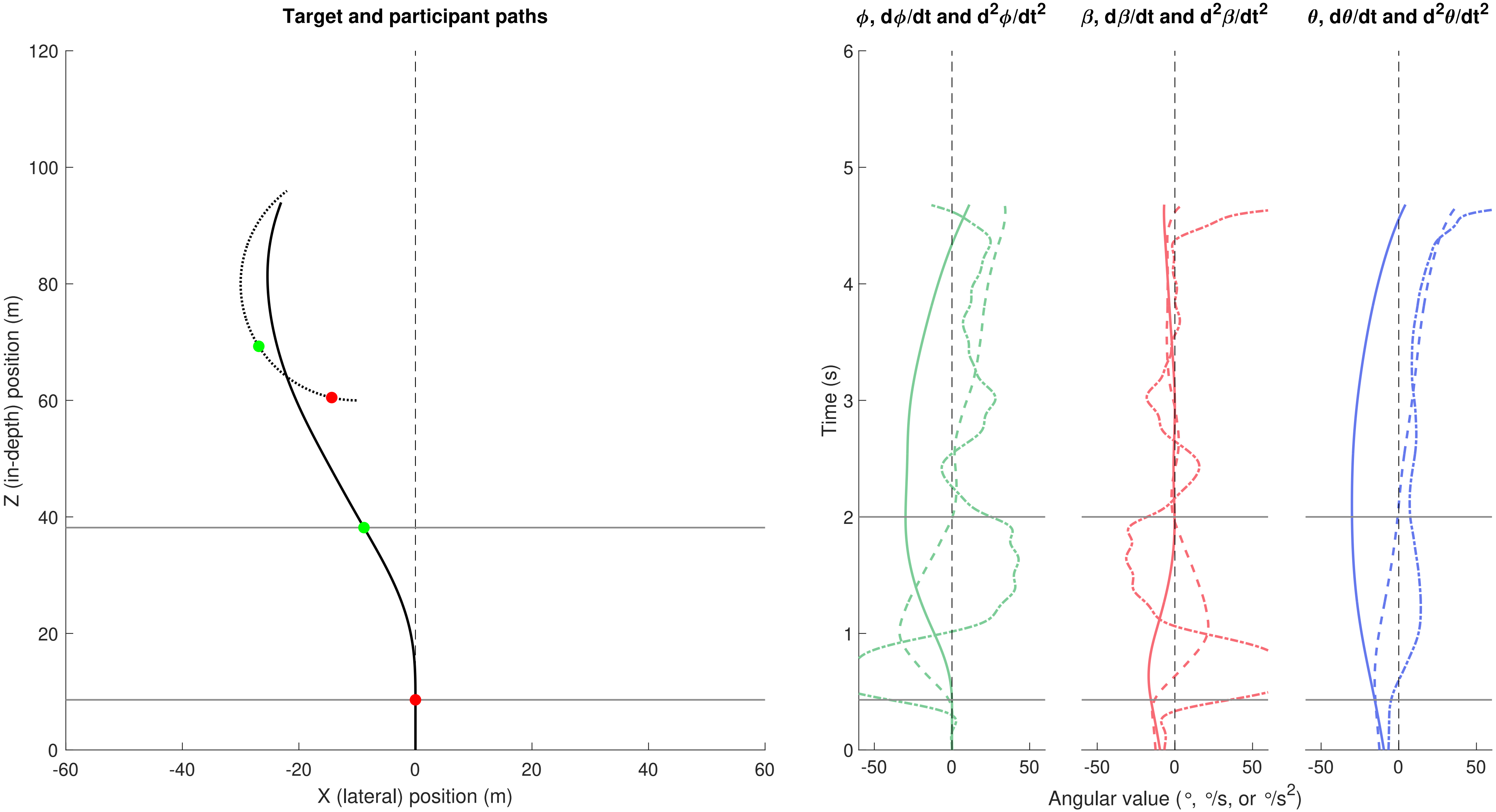

P6/B3
S10/R40-IN

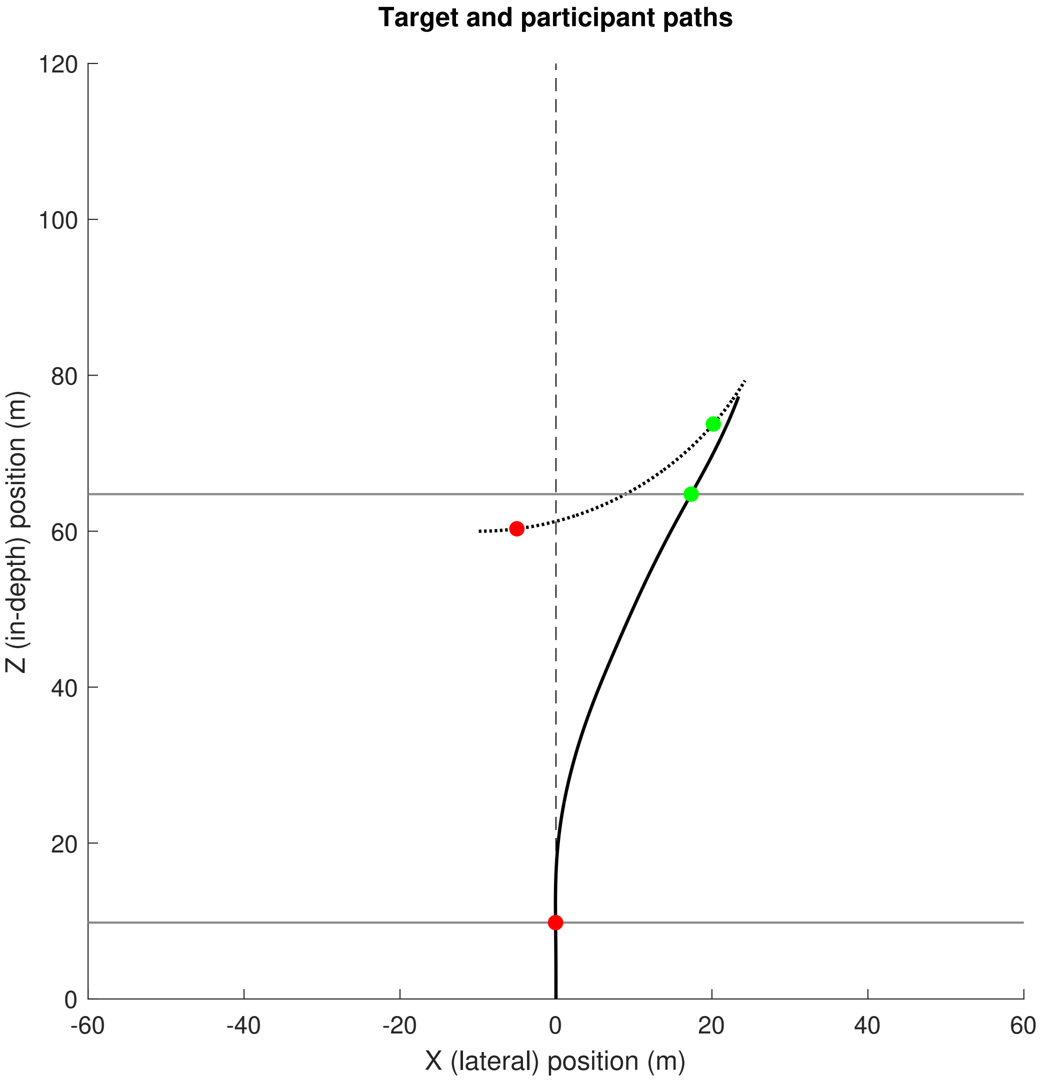

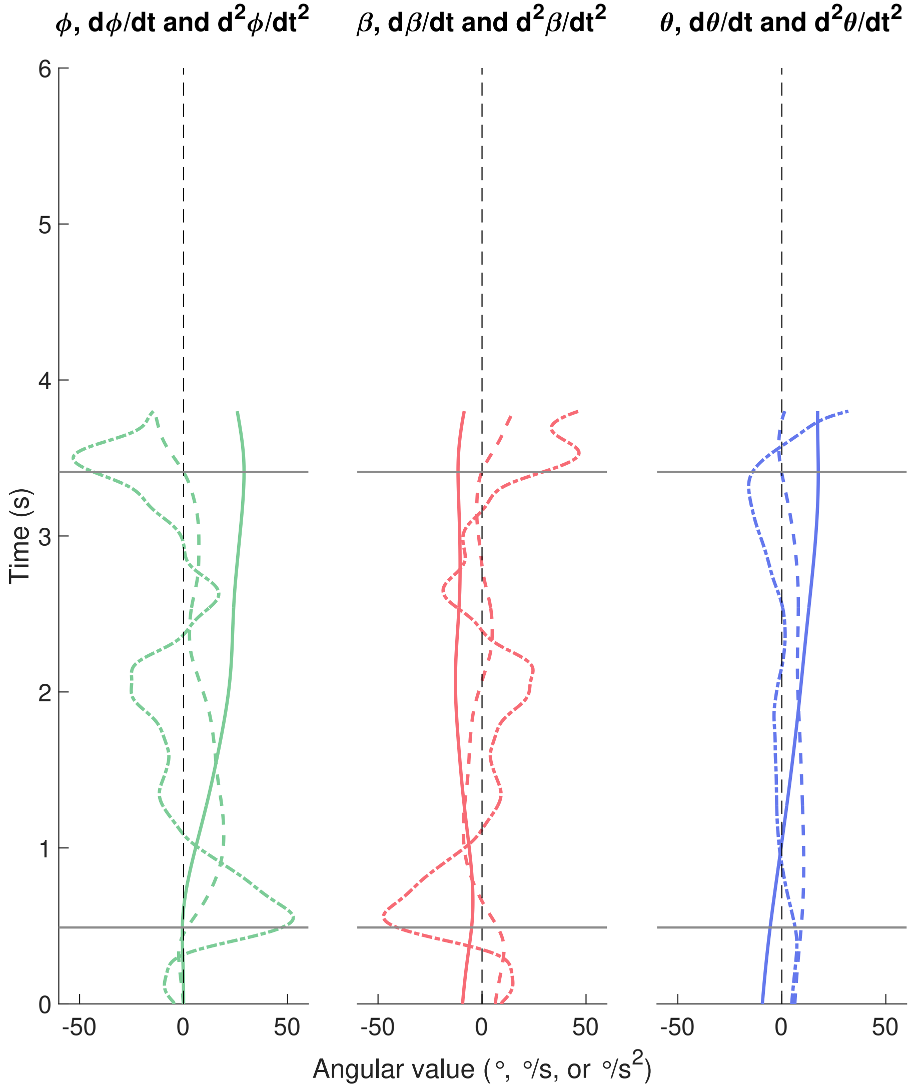

P6/B3
S10/R40-OUT

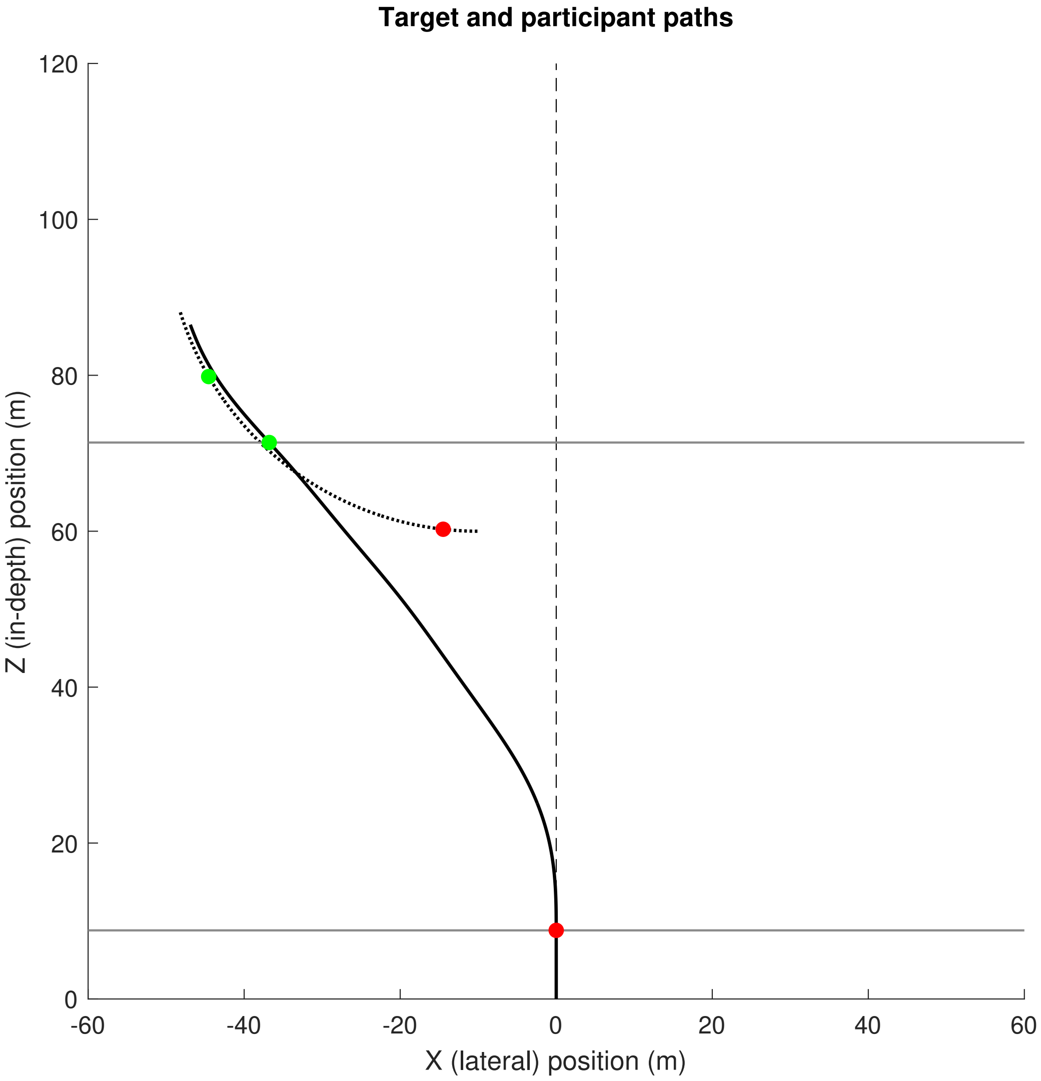

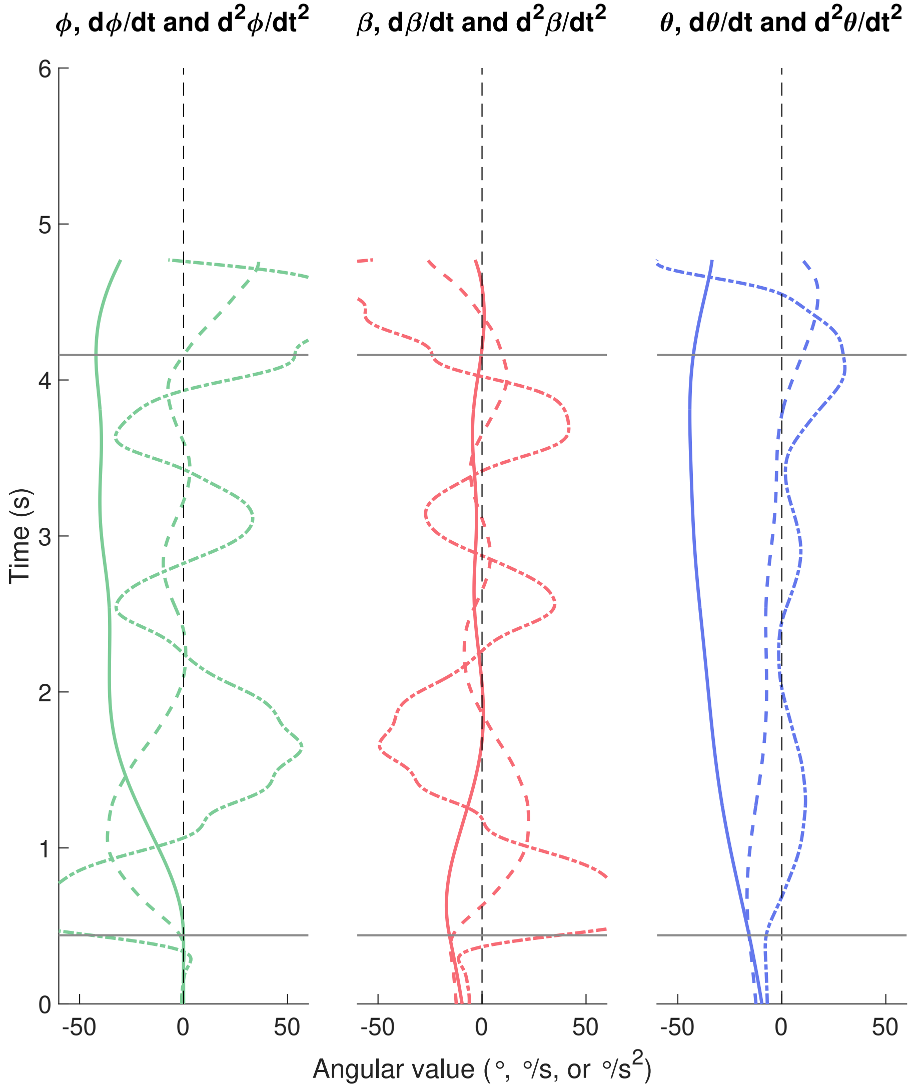

P6/B3
S0/R20-OUT

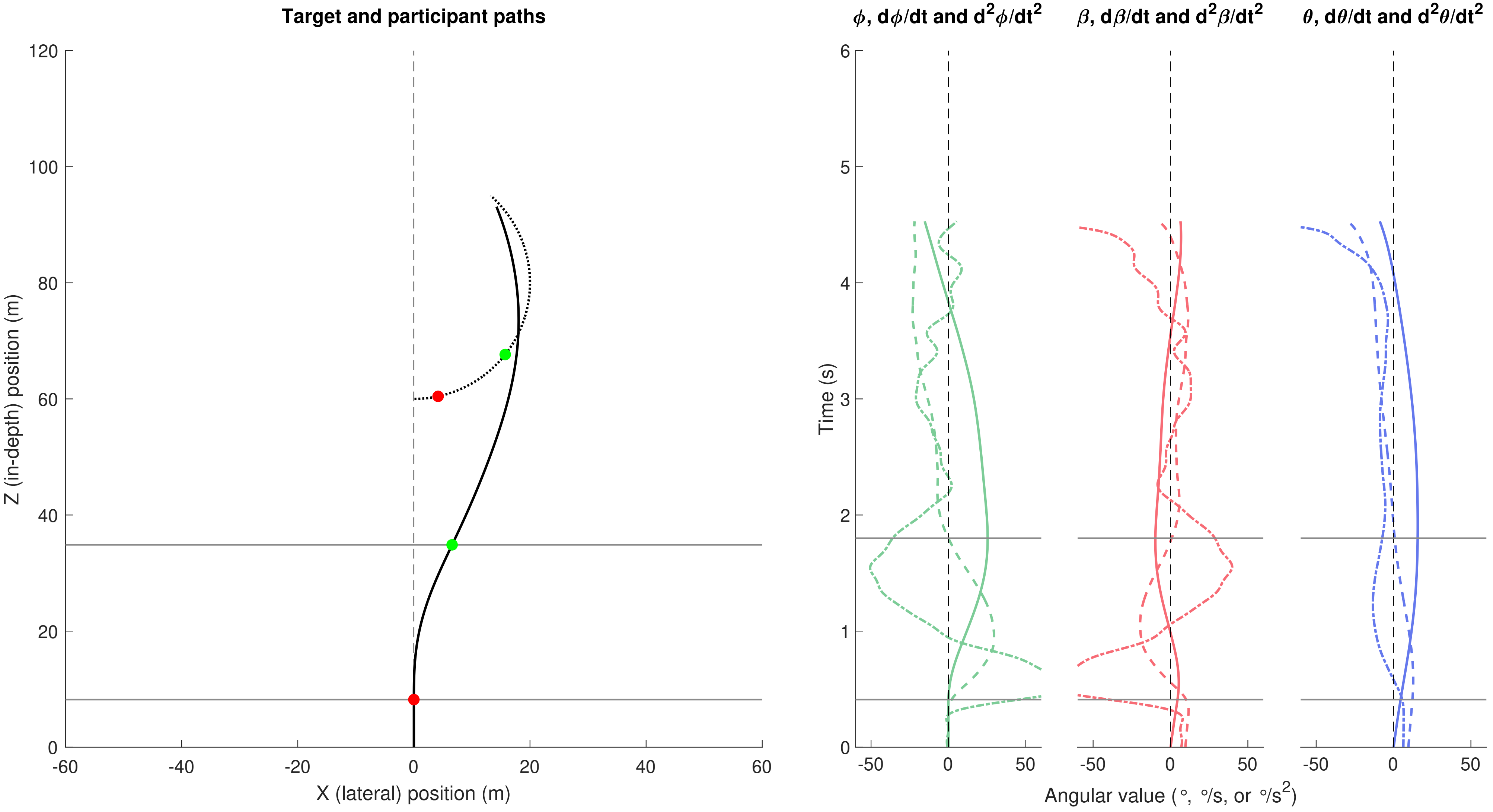

P6/B3
S0/R20-OUT

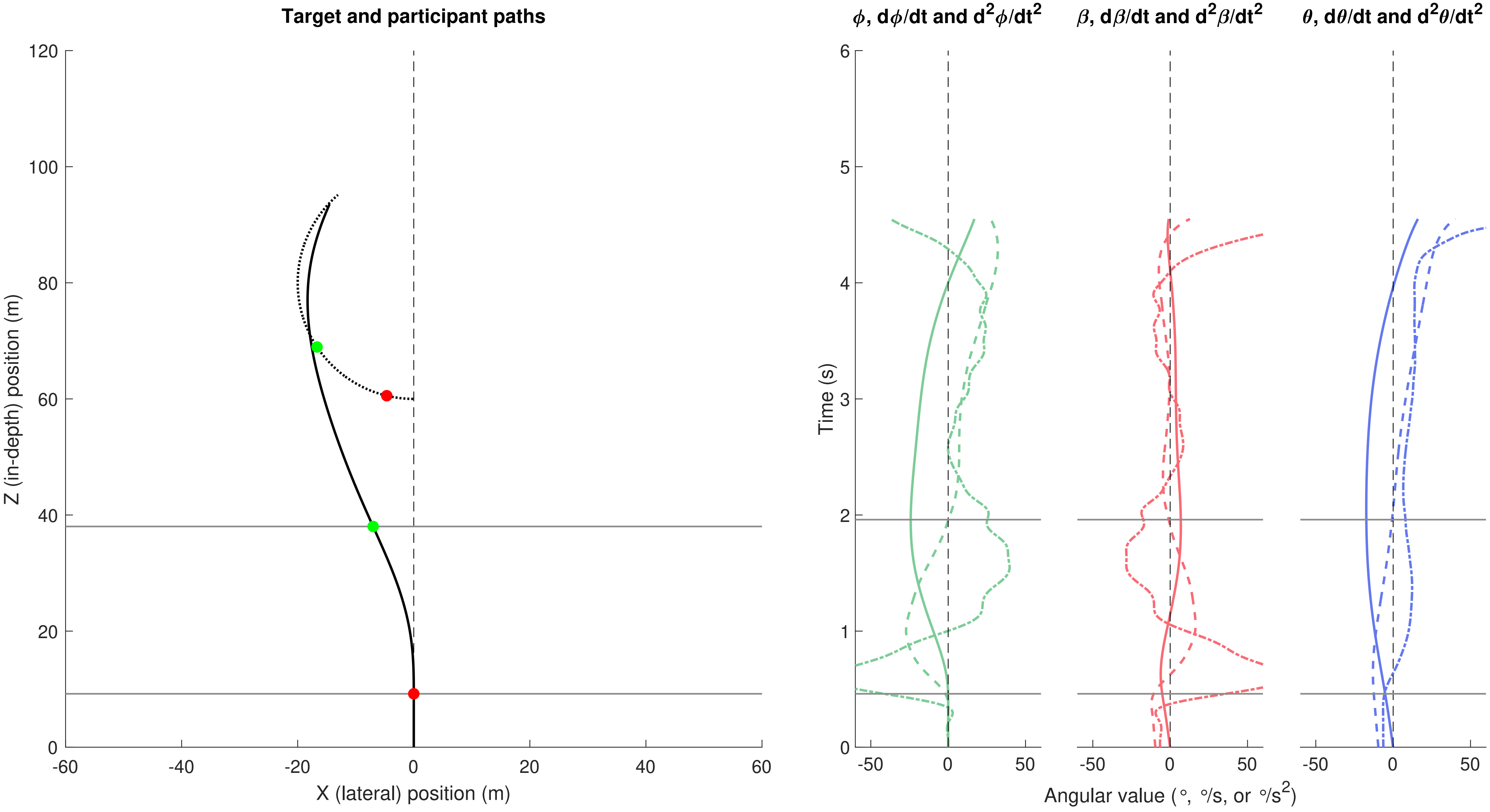

P6/B3
S0/R40-OUT

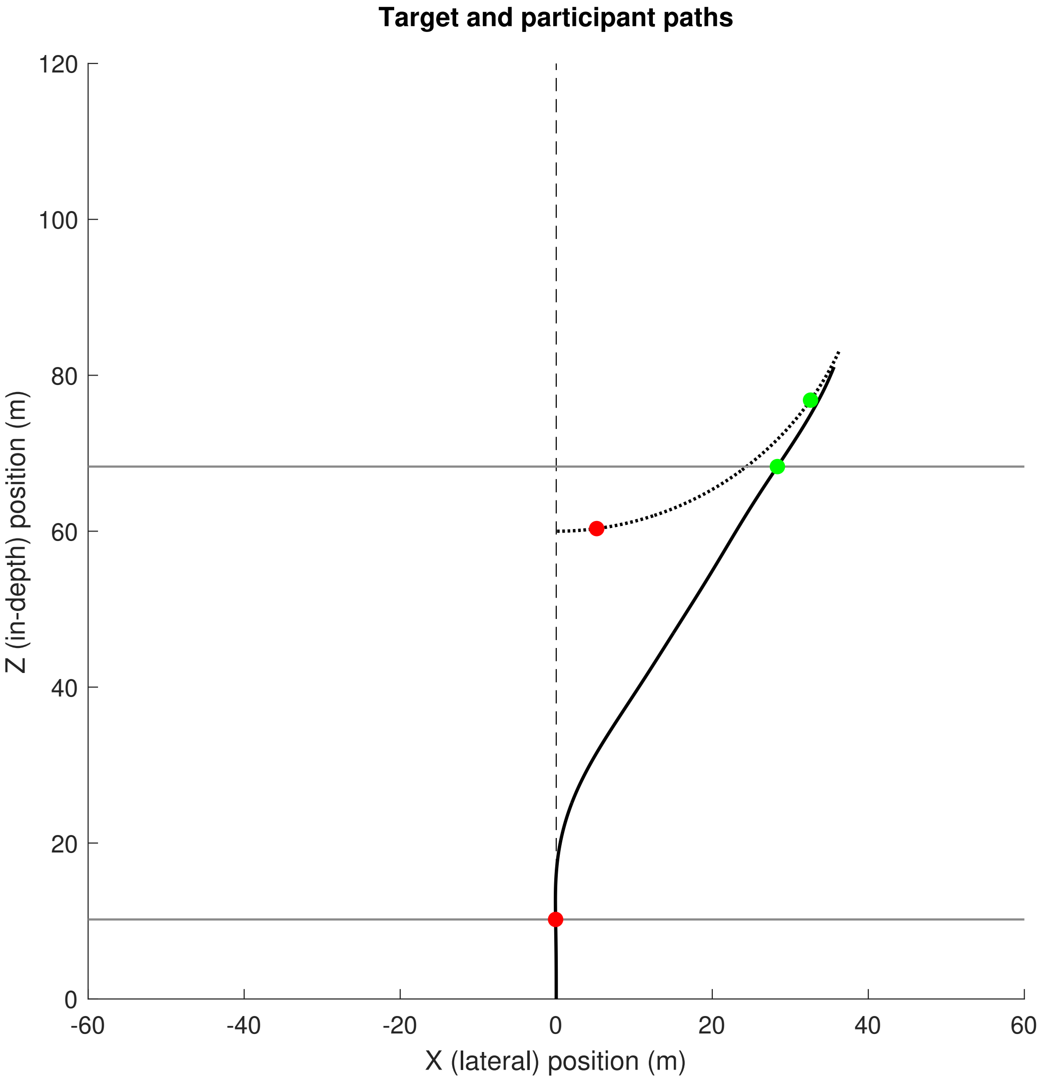

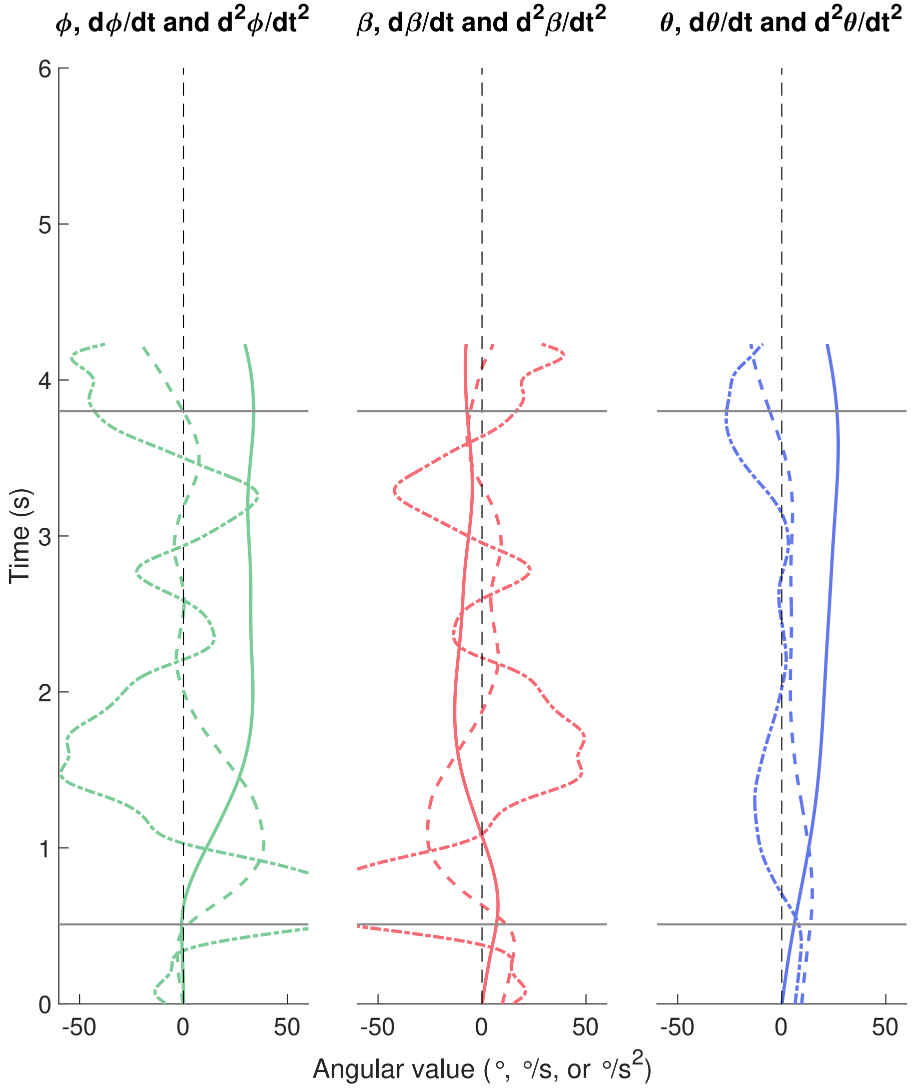

P6/B3
S0/R40-OUT

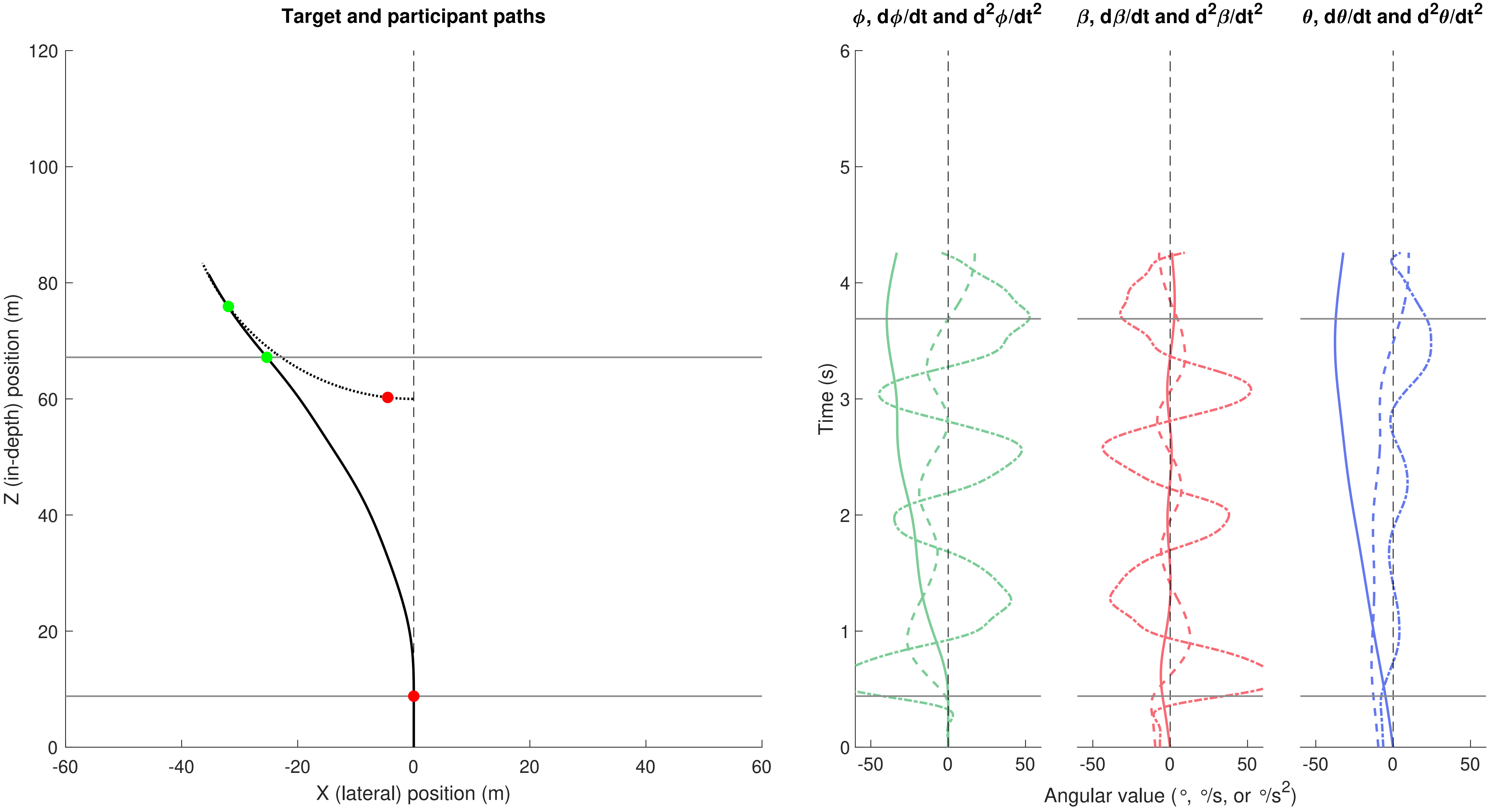

P6/B3
S10/R20-OUT

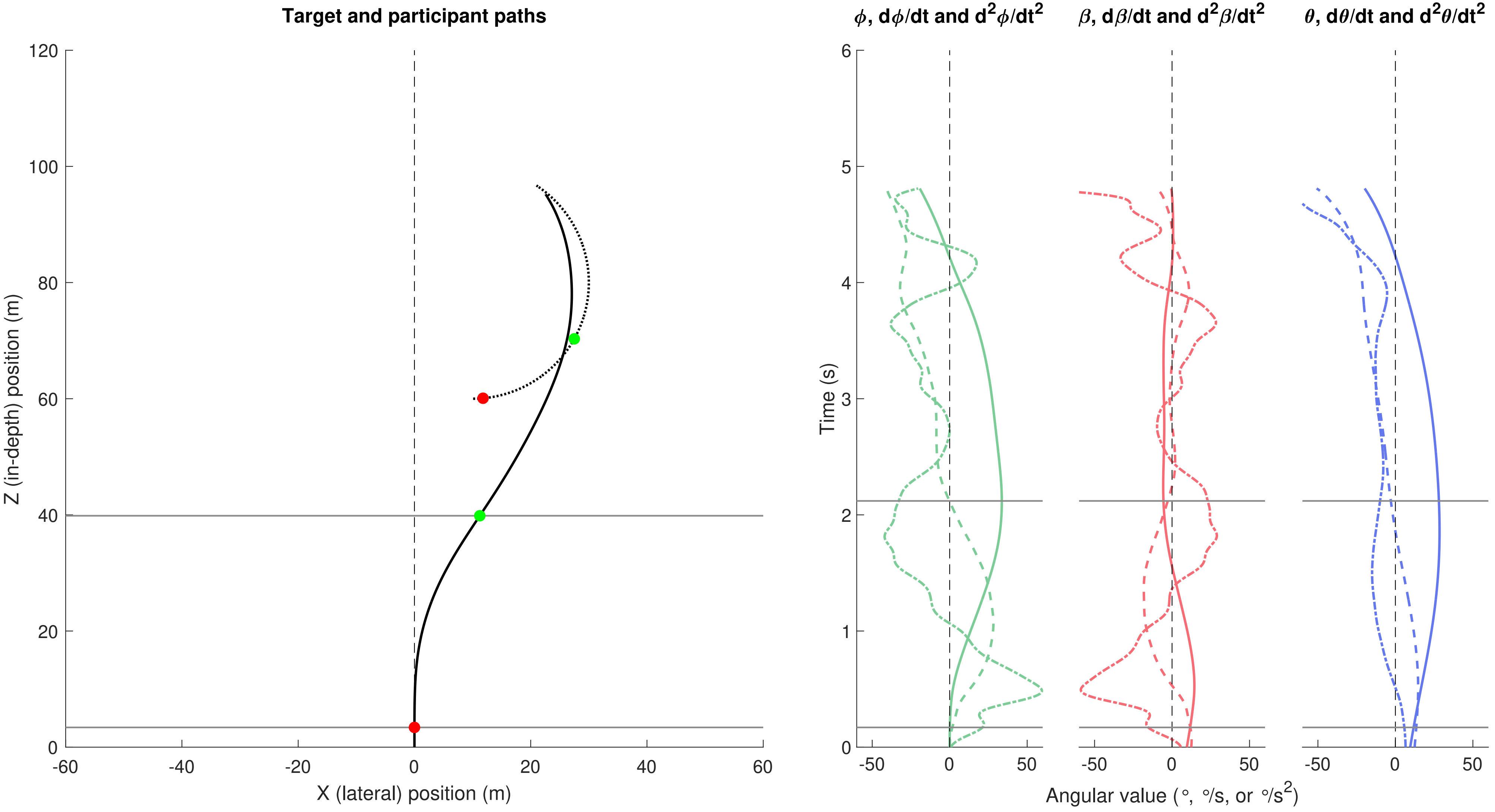

P6/B3
S10/R20-IN

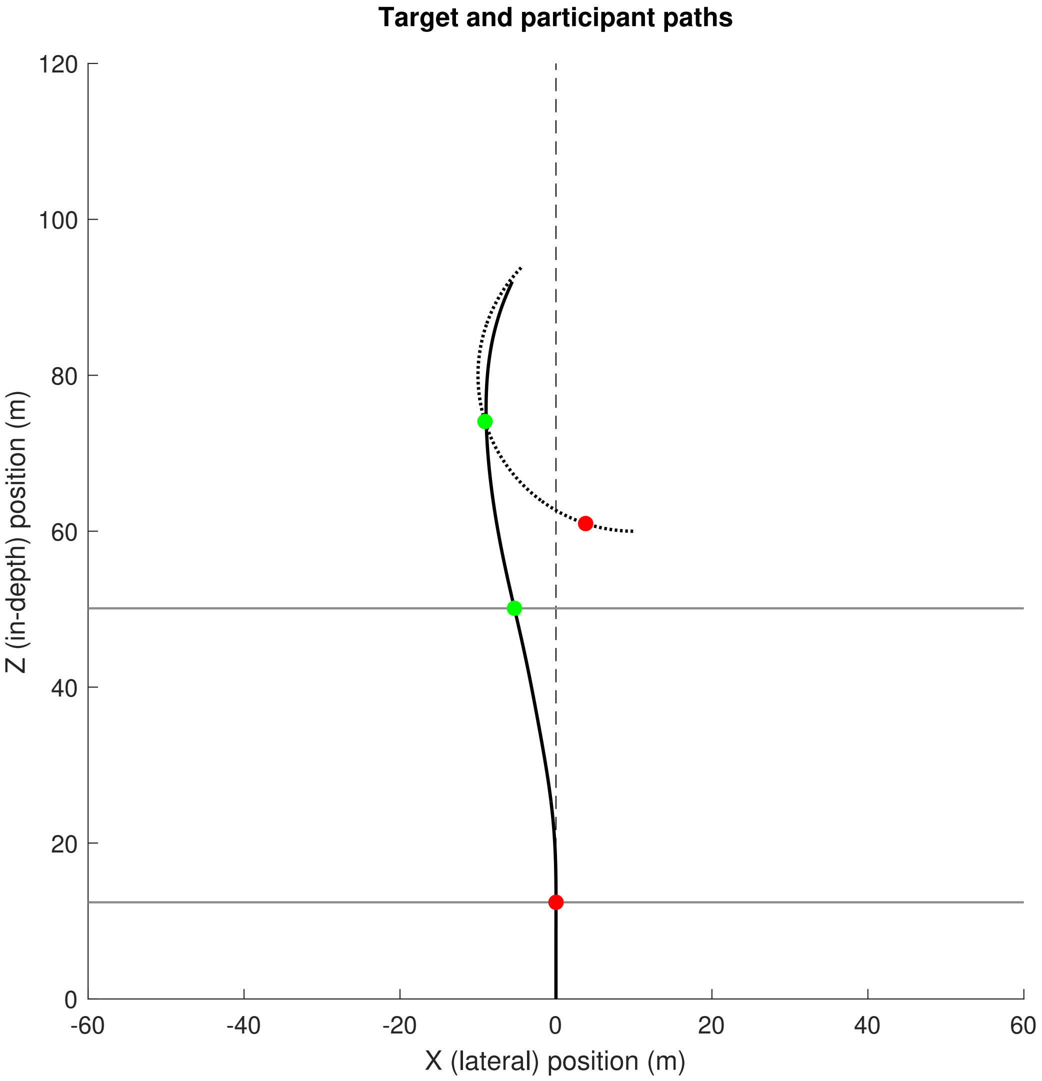

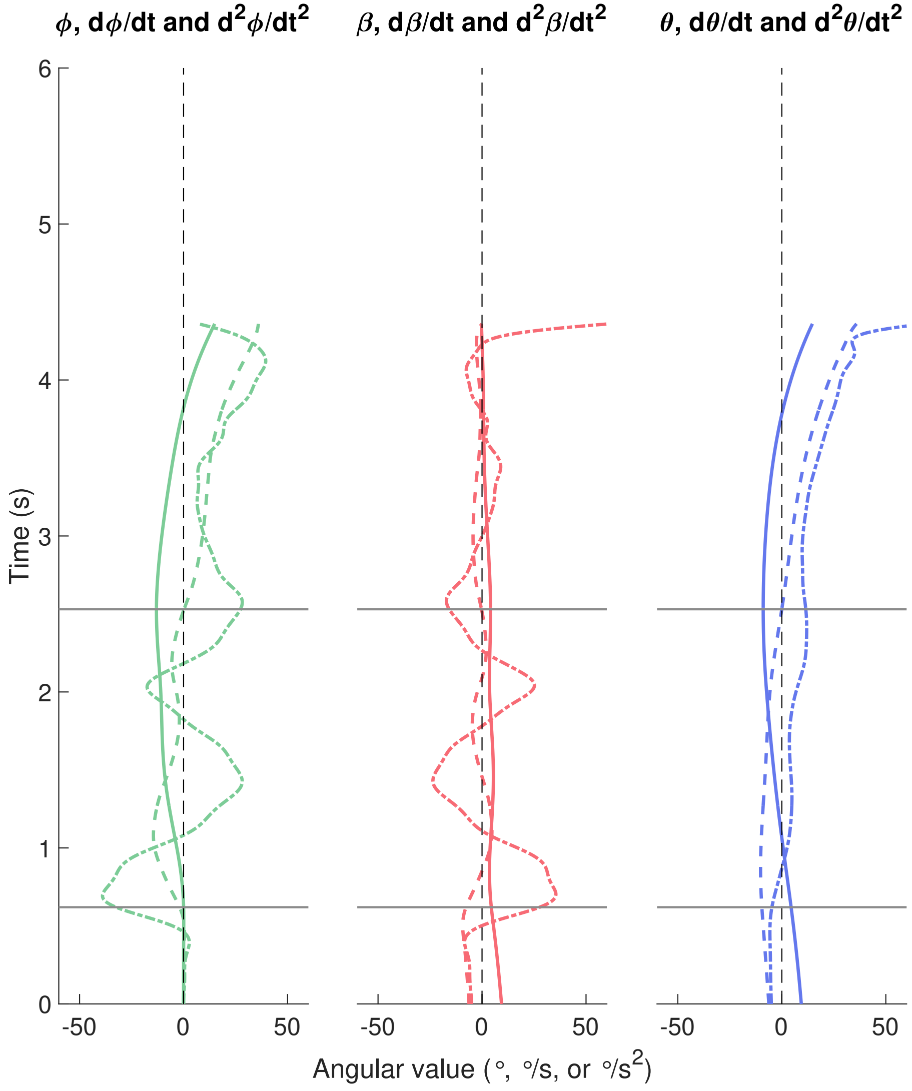

P6/B3
S10/R40-OUT

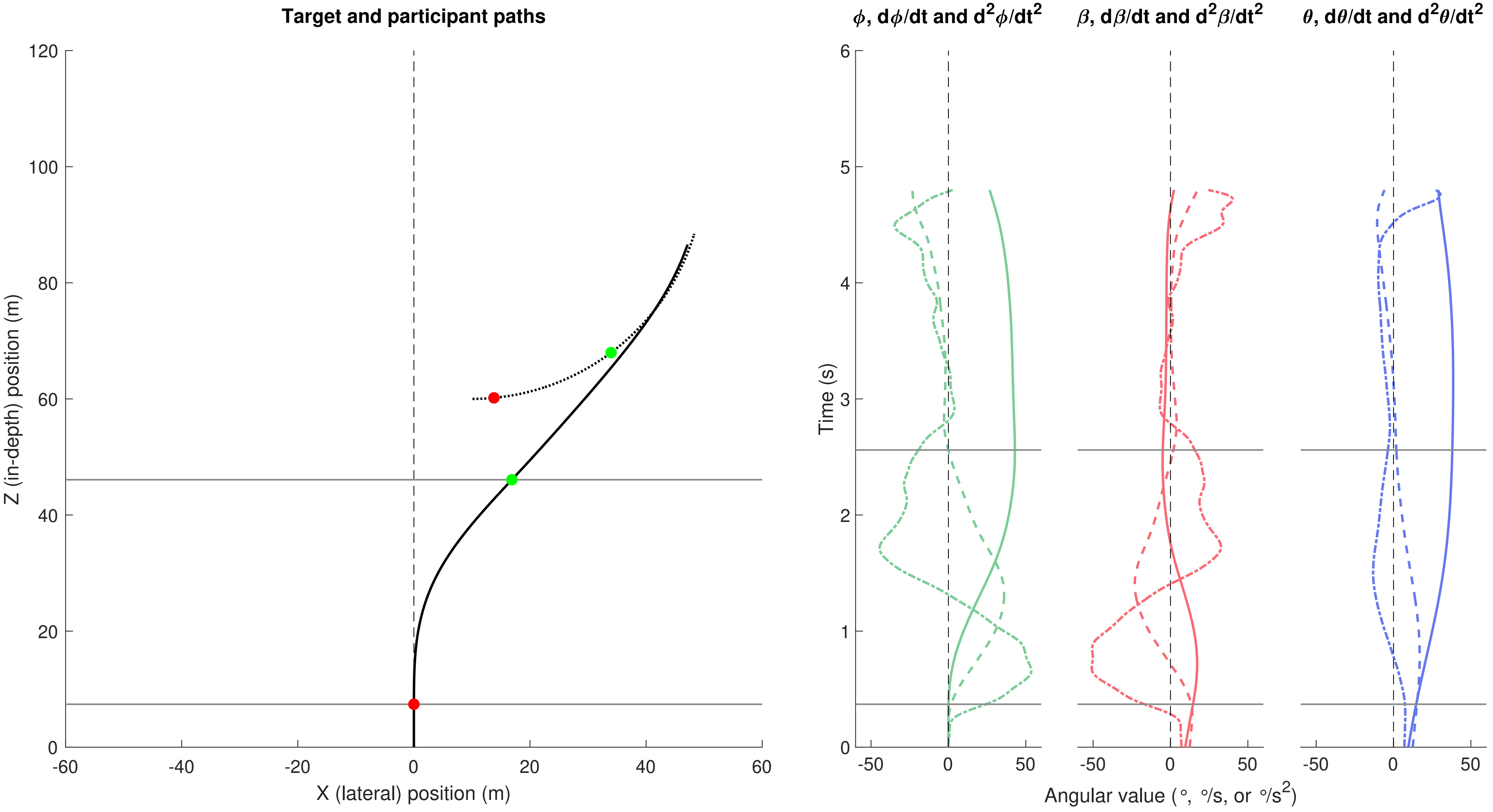

P6/B3
S10/R40-IN

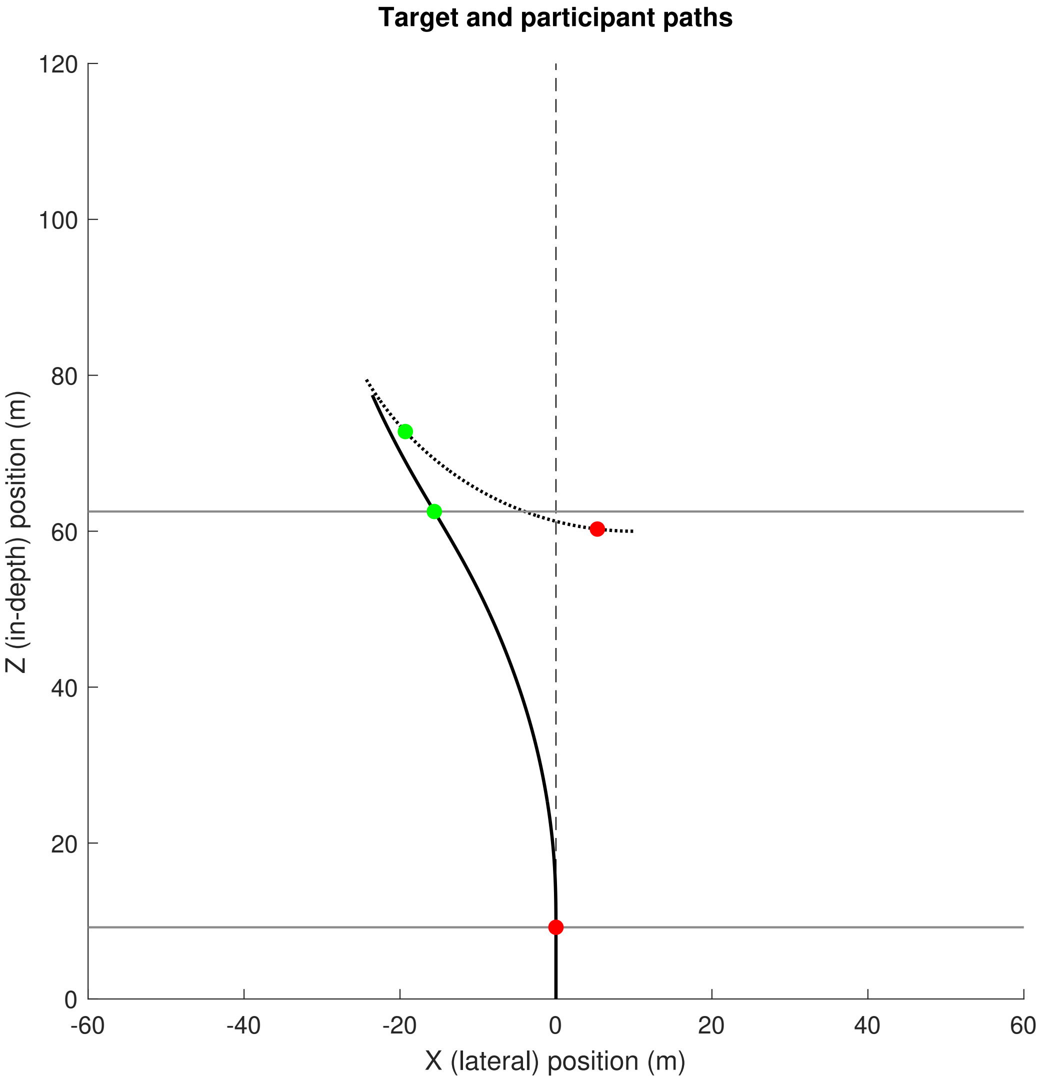

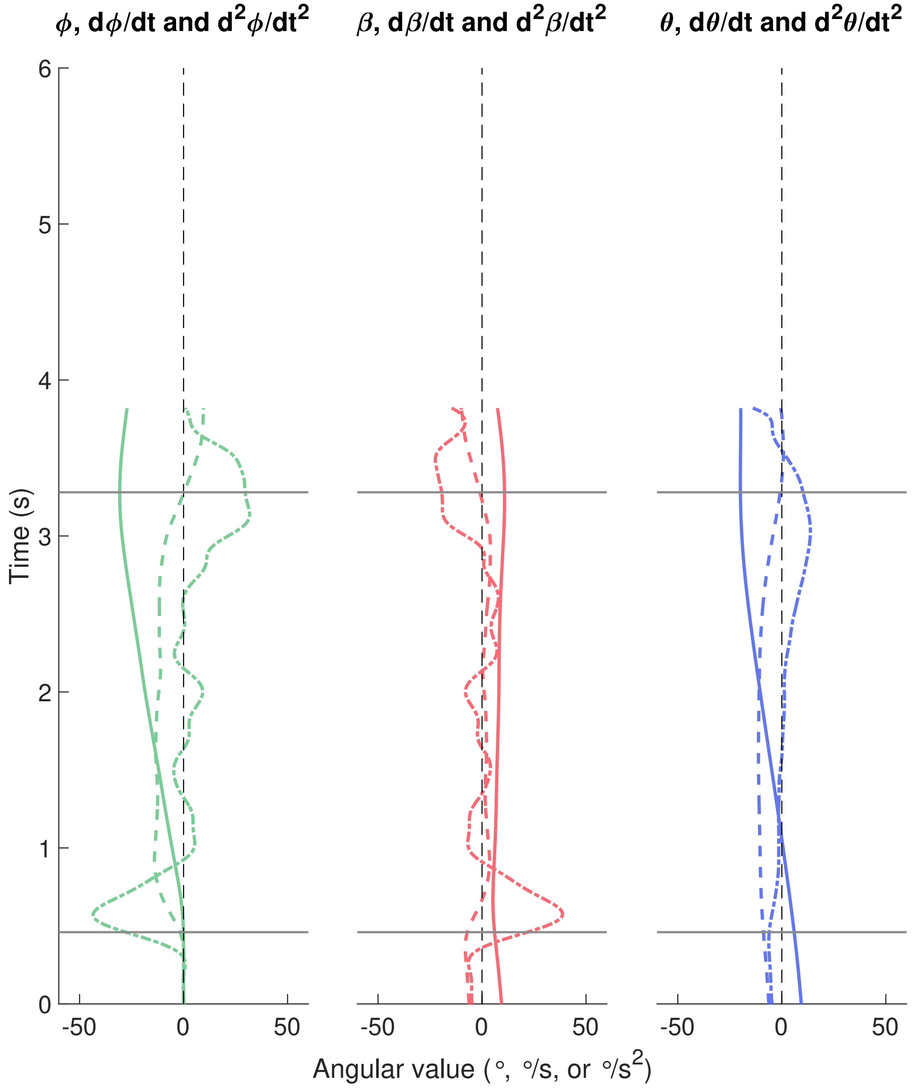

P6/B3
S20/R20-OUT

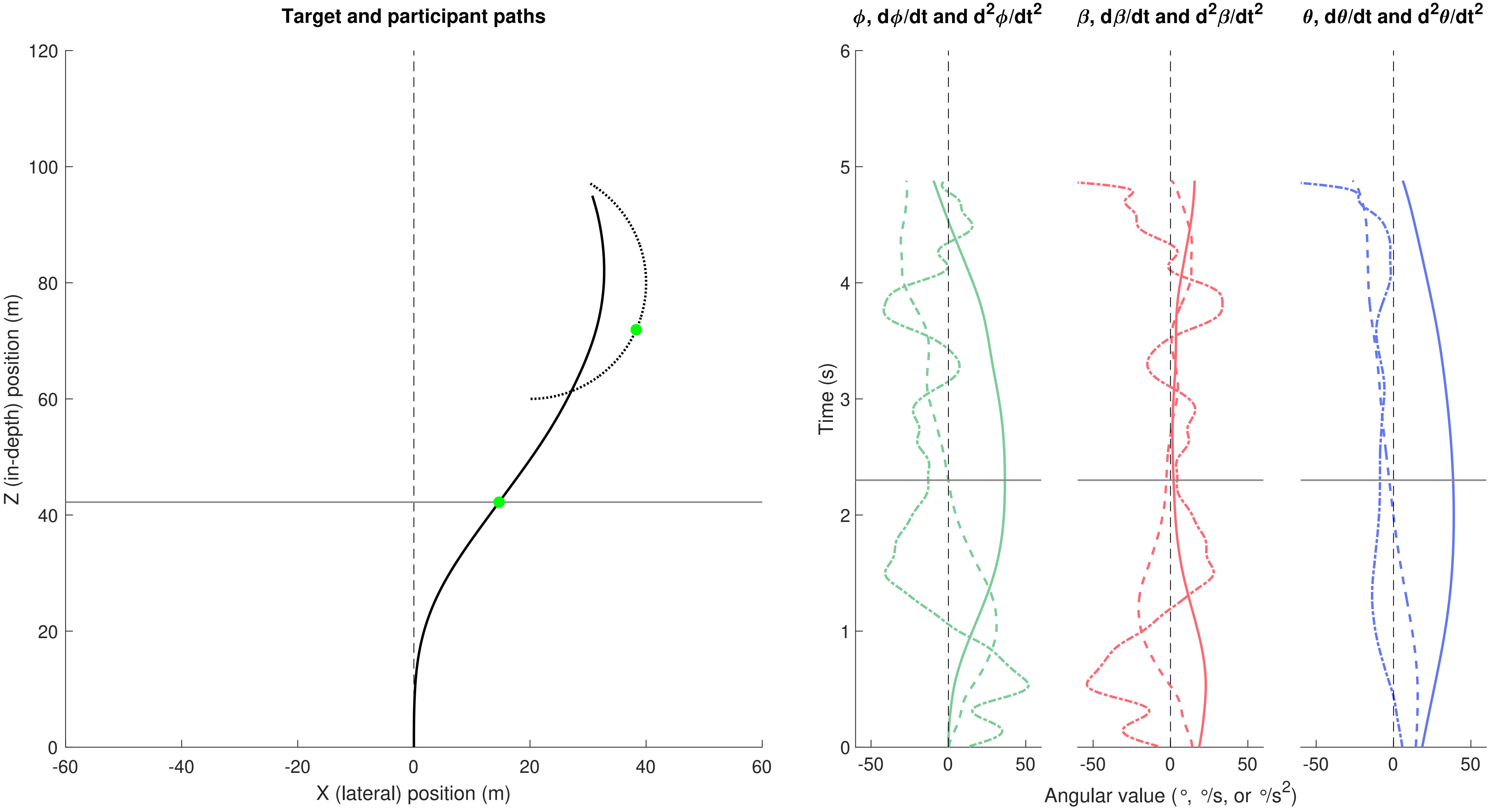

P6/B3
S20/R20-IN

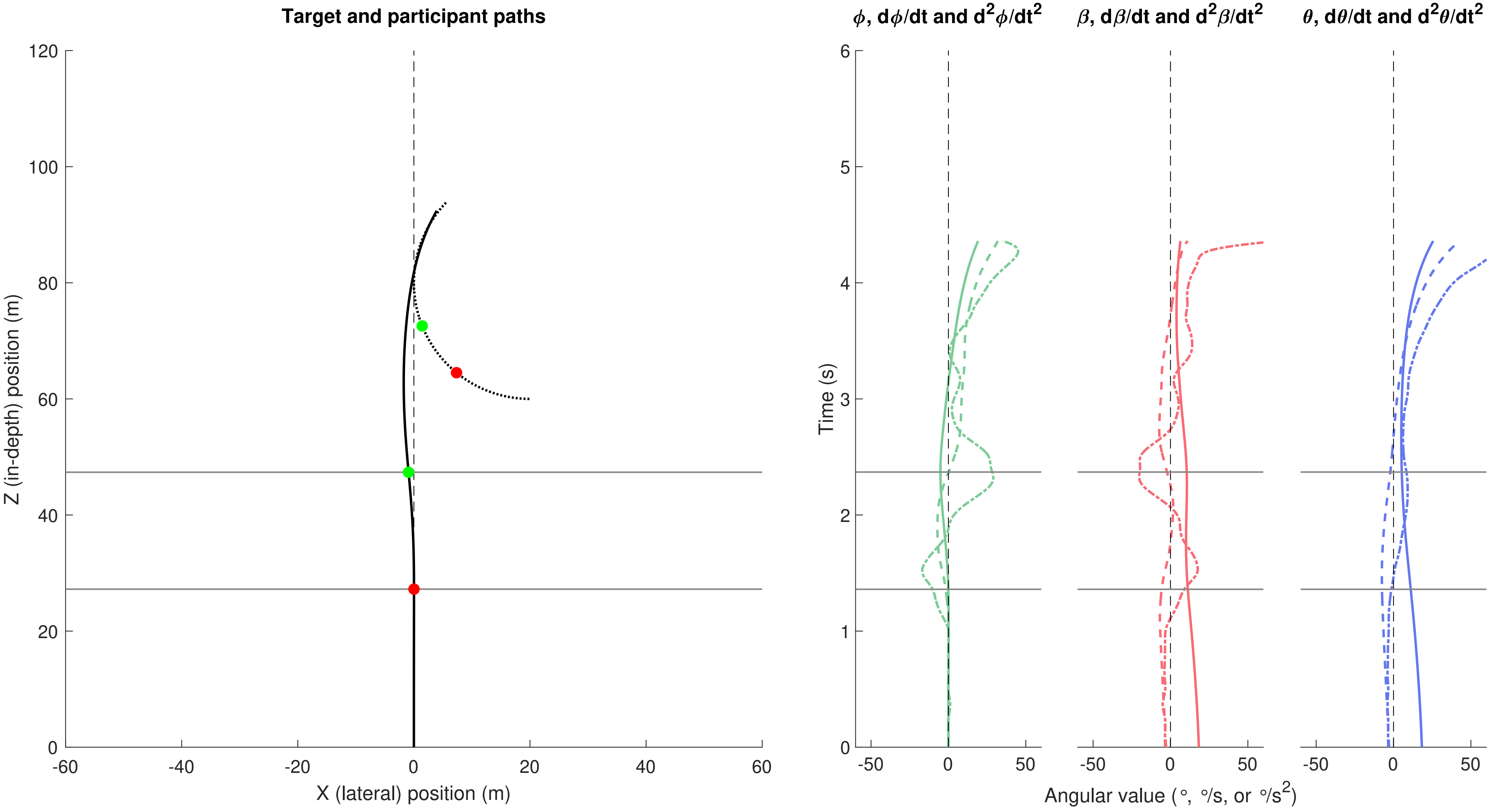

P6/B3
S20/R40-OUT

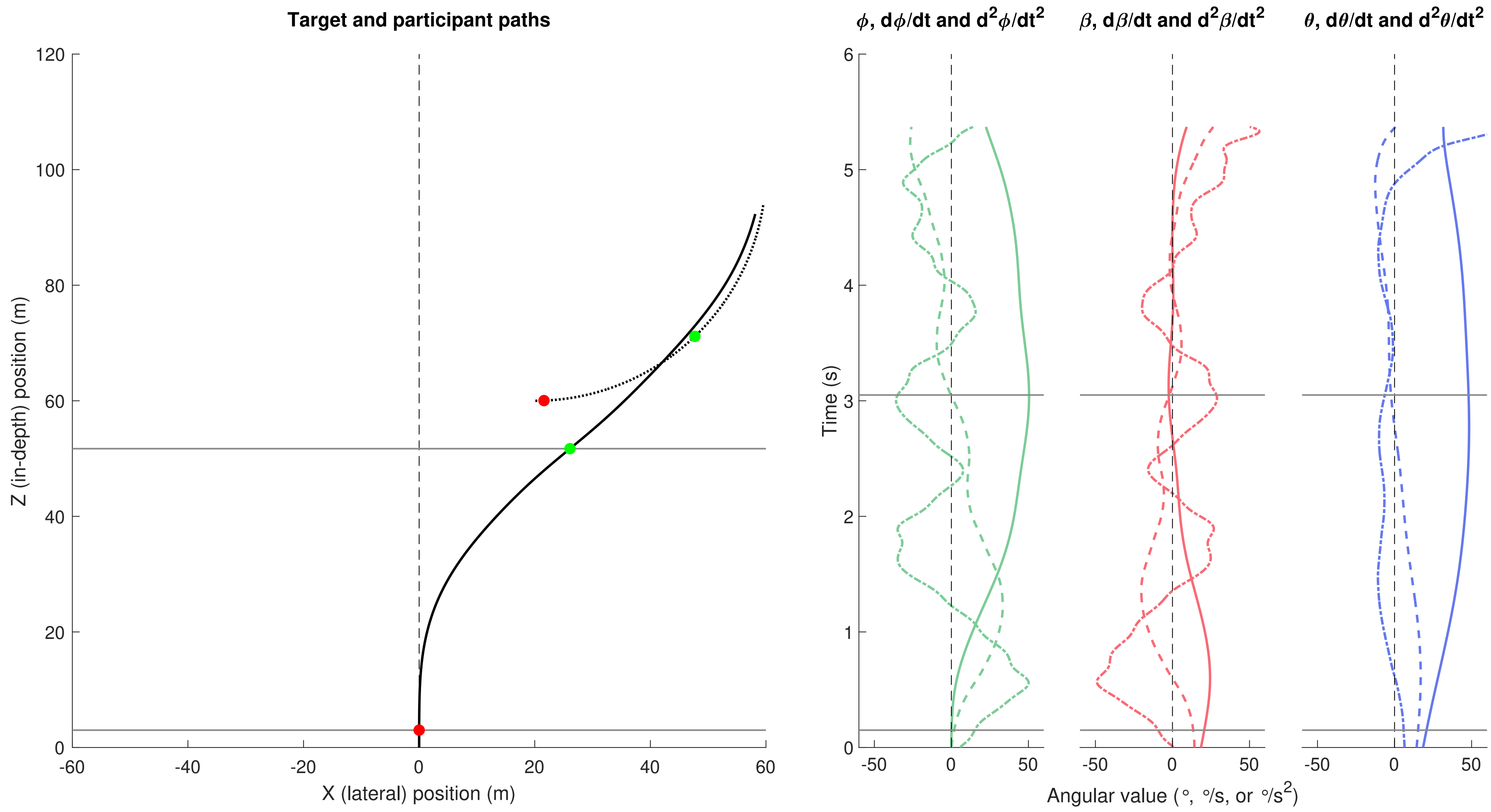

P6/B3
S20/R40-IN

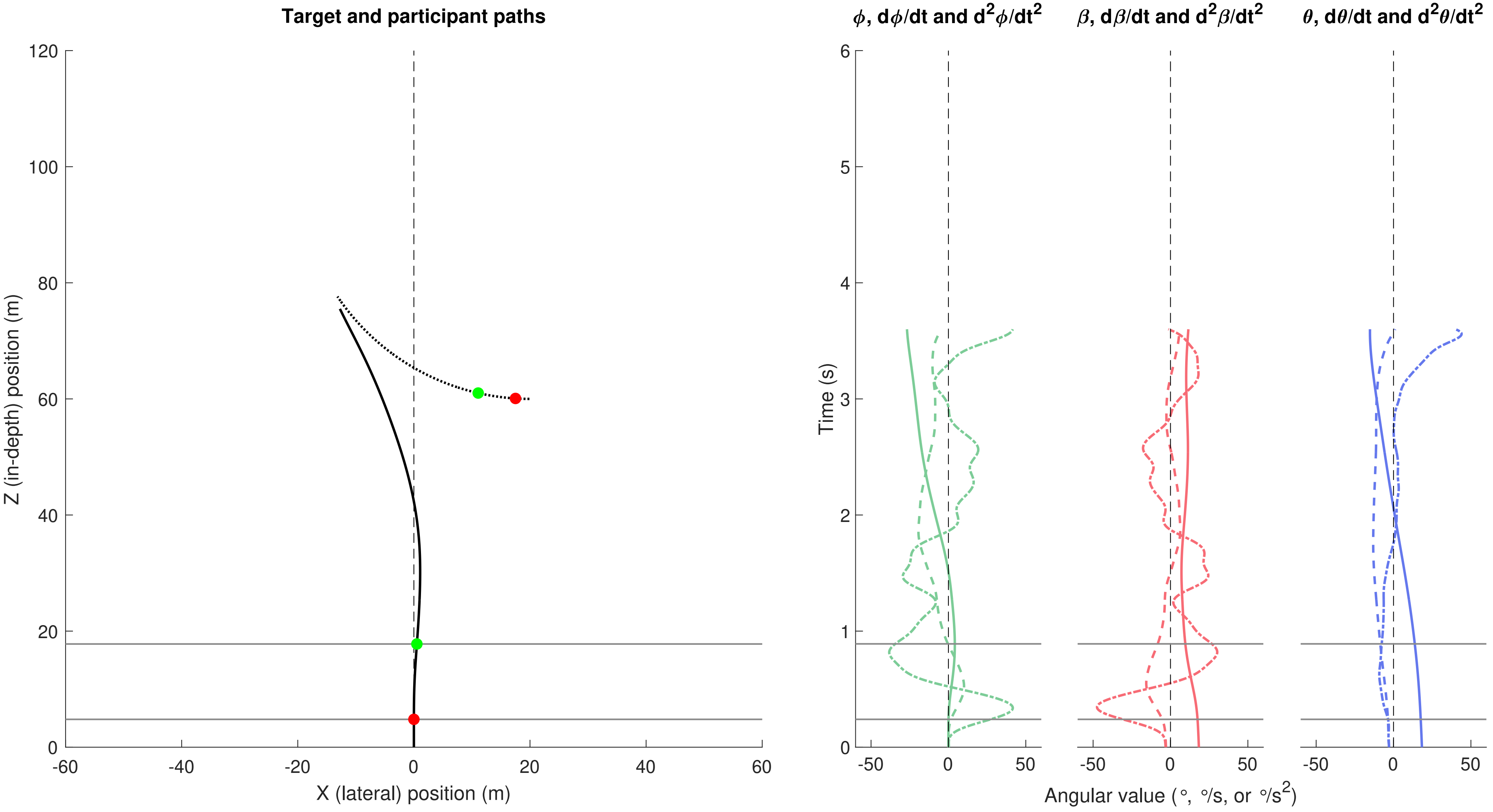

P6/B4
S20/R20-IN

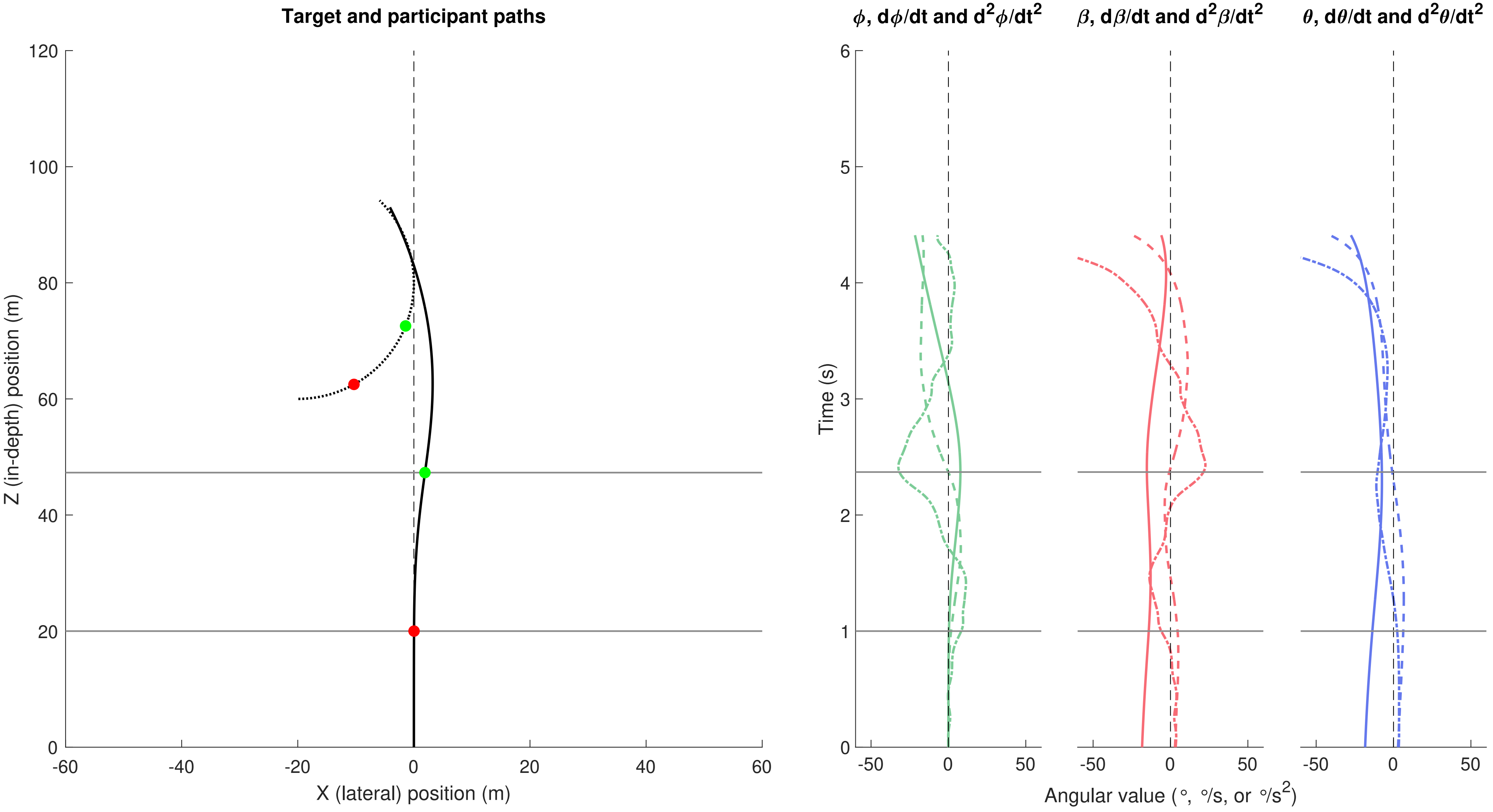

P6/B4
S20/R20-OUT

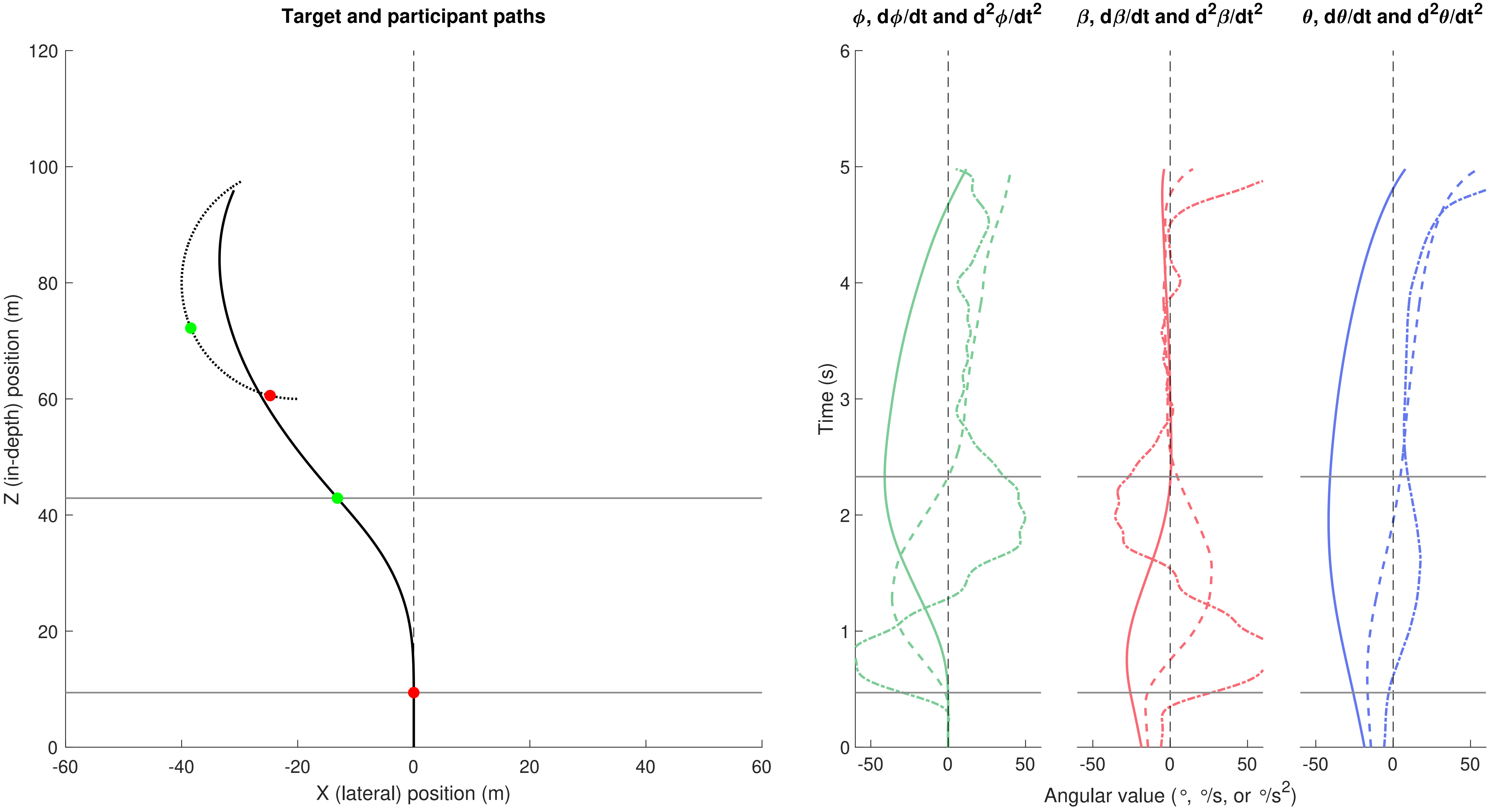

P6/B4
S20/R40-IN

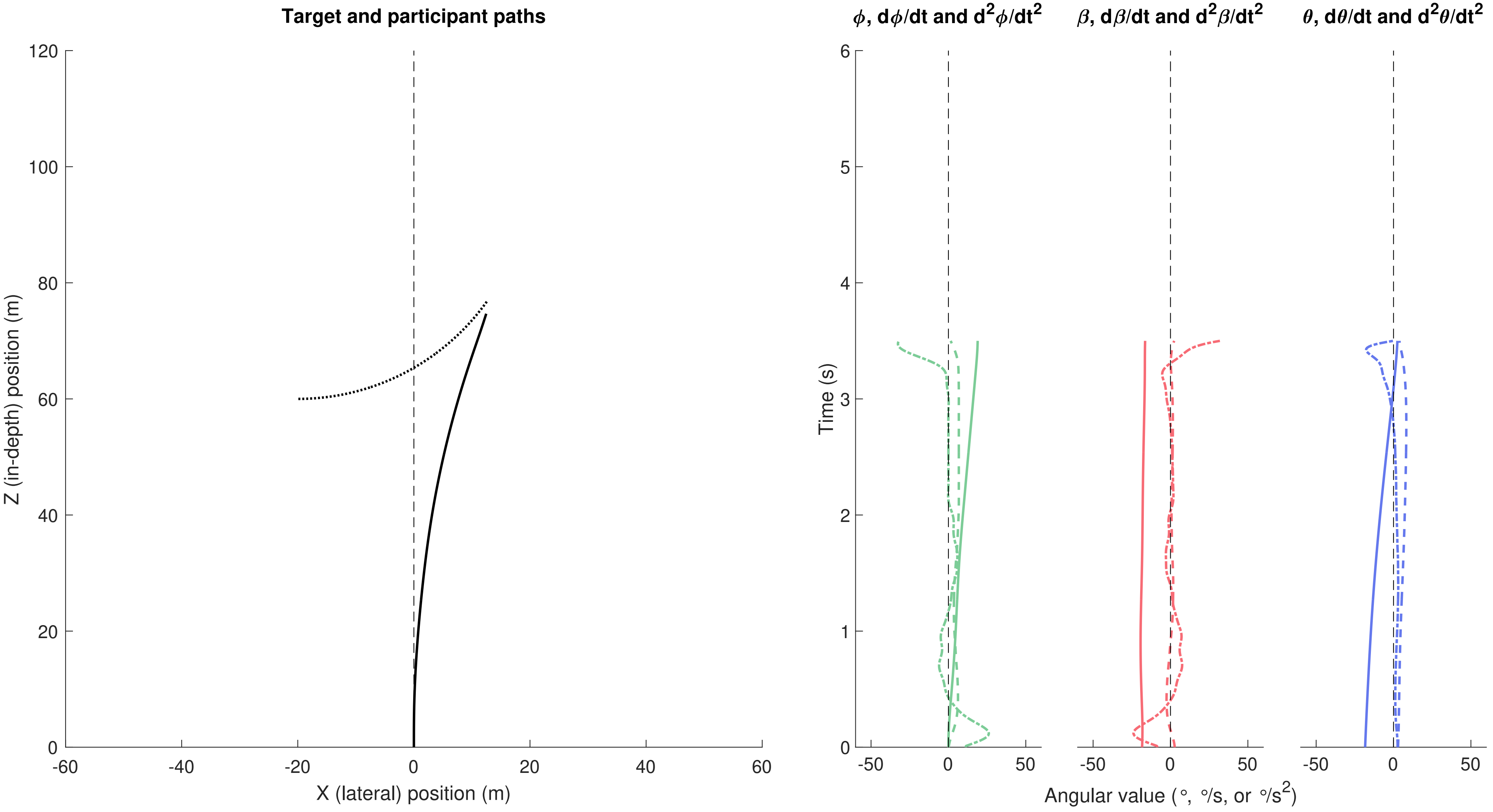

P6/B4
S20/R40-OUT

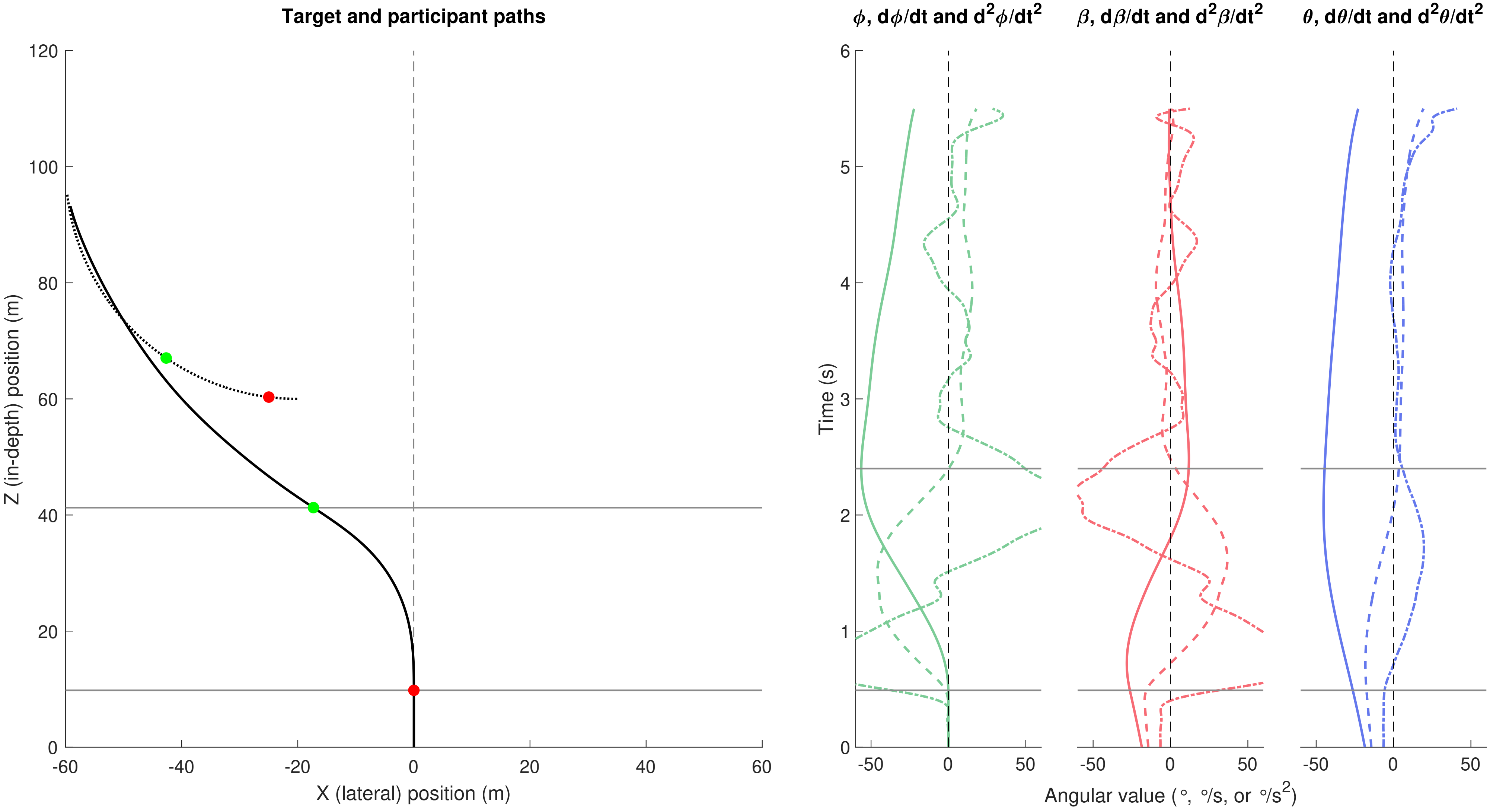

P6/B4
S10/R20-IN

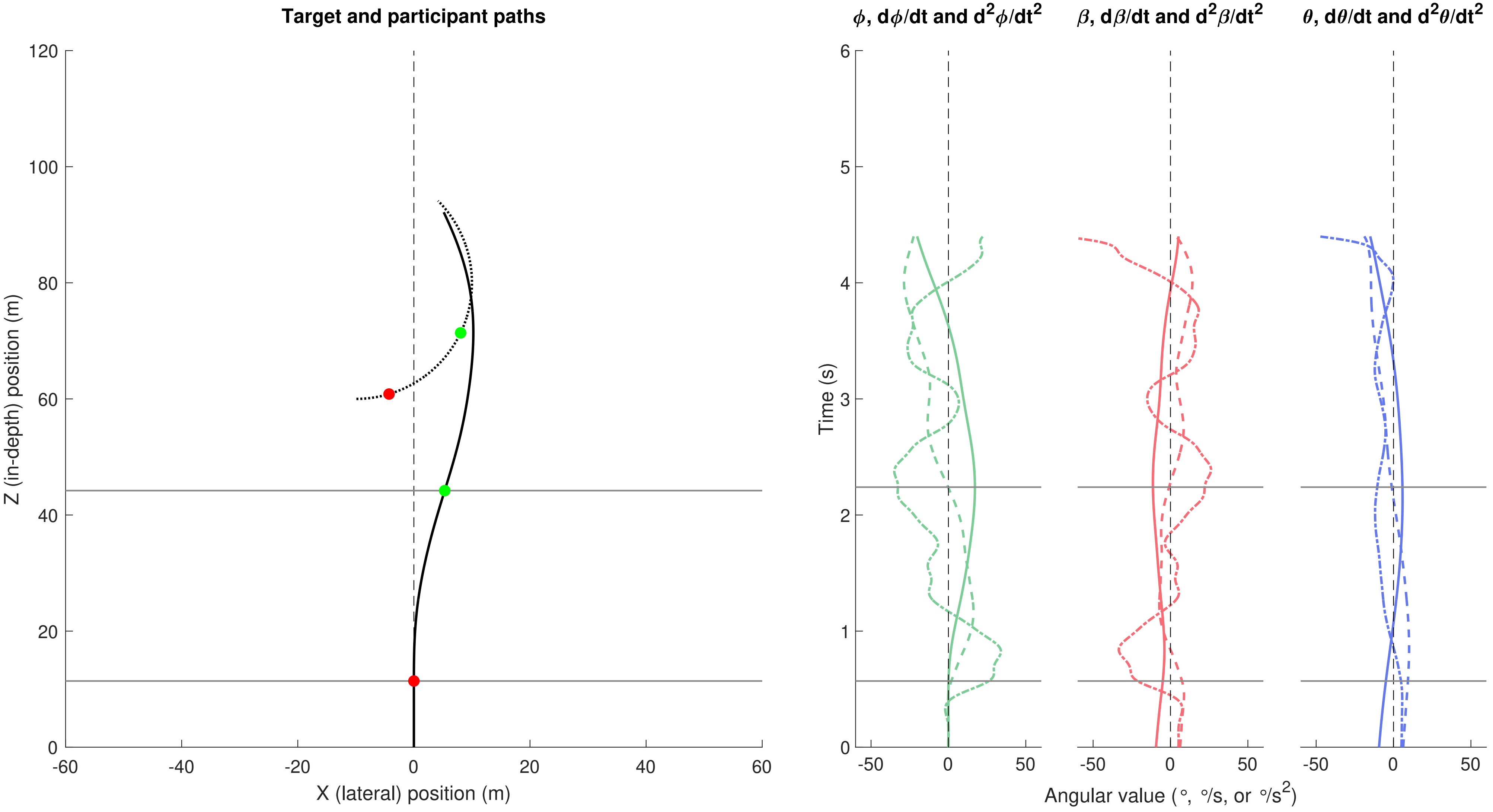

P6/B4
S10/R20-OUT

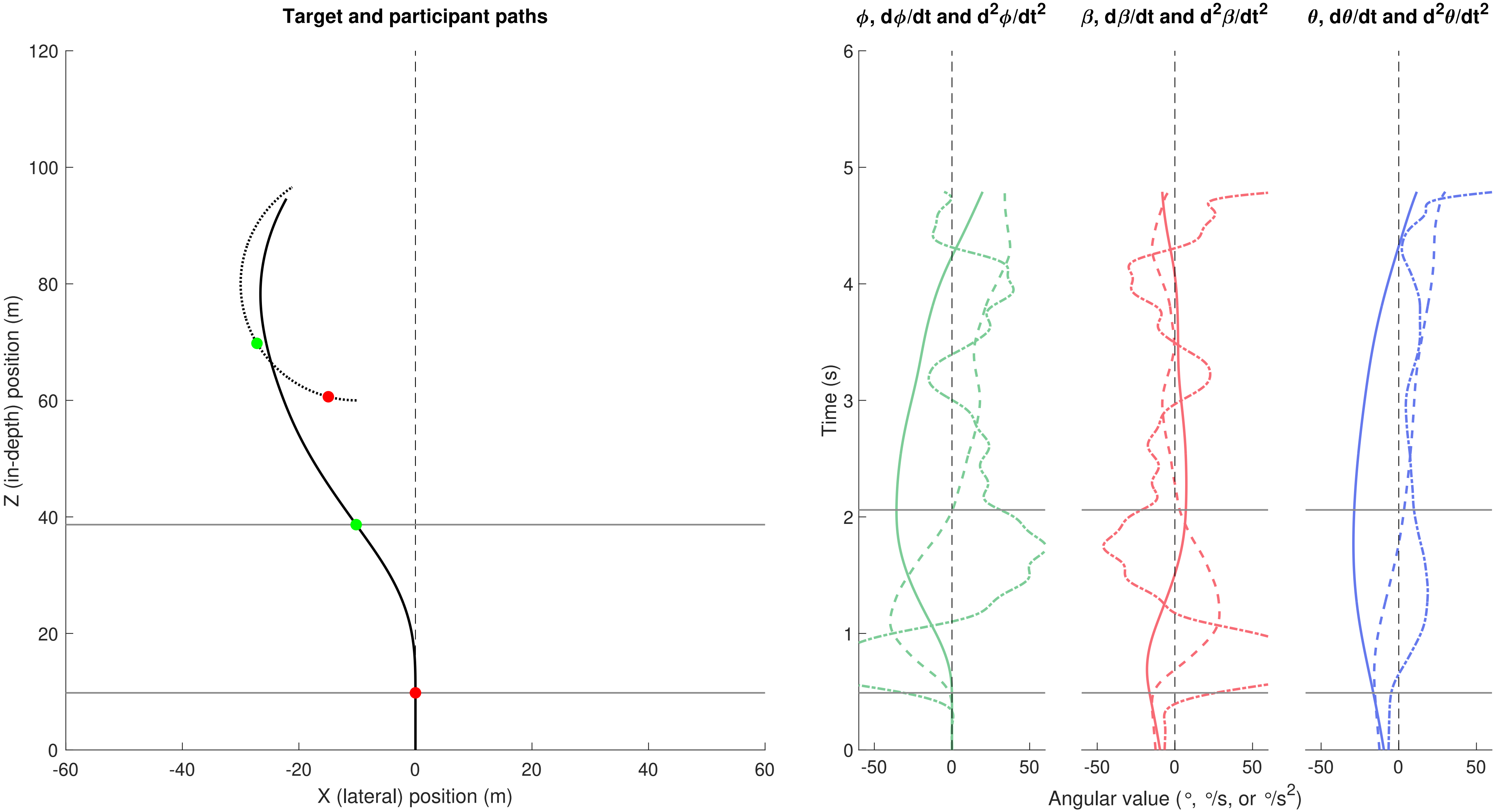

P6/B4
S10/R40-IN

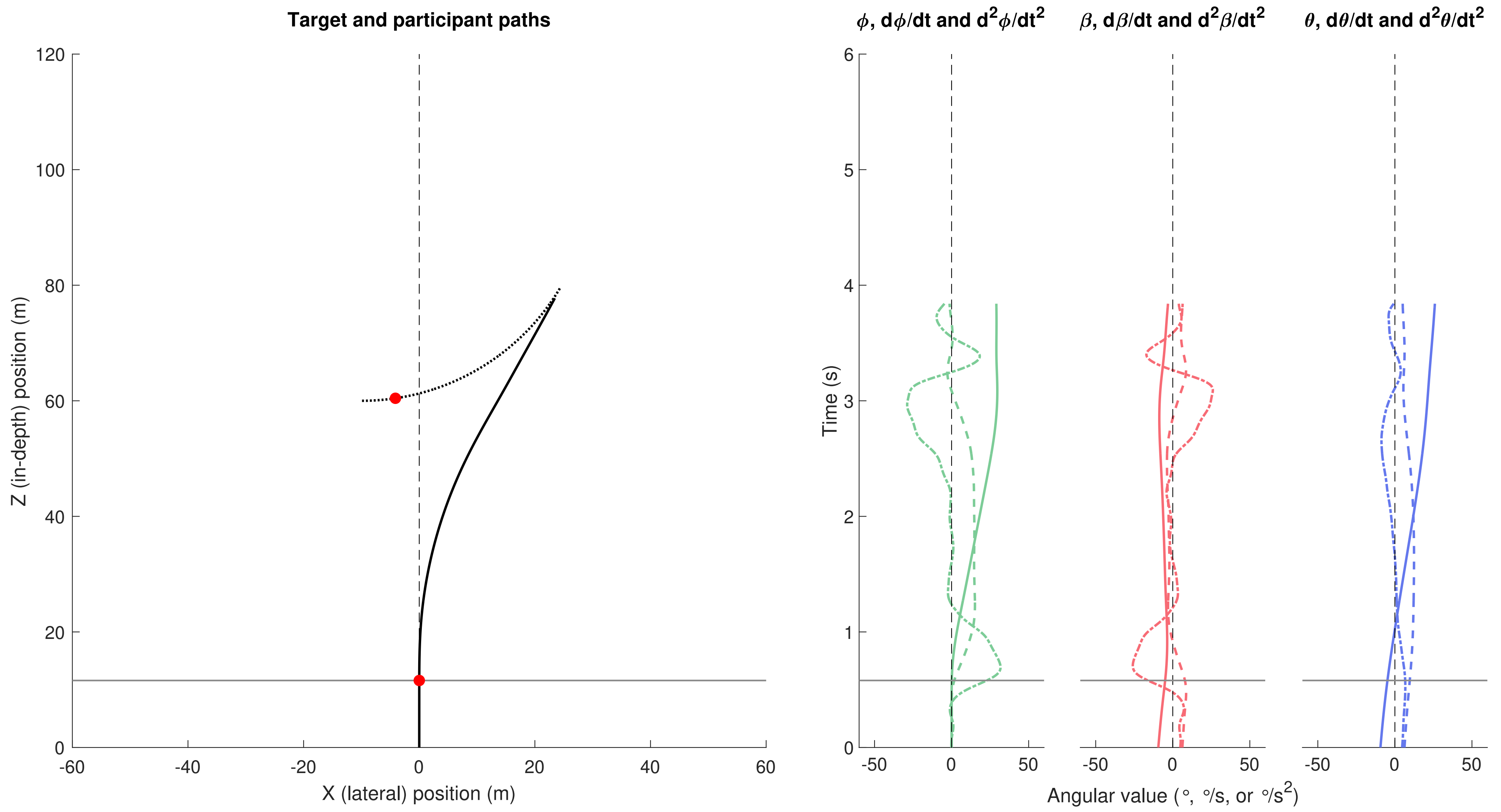

P6/B4
S10/R40-OUT

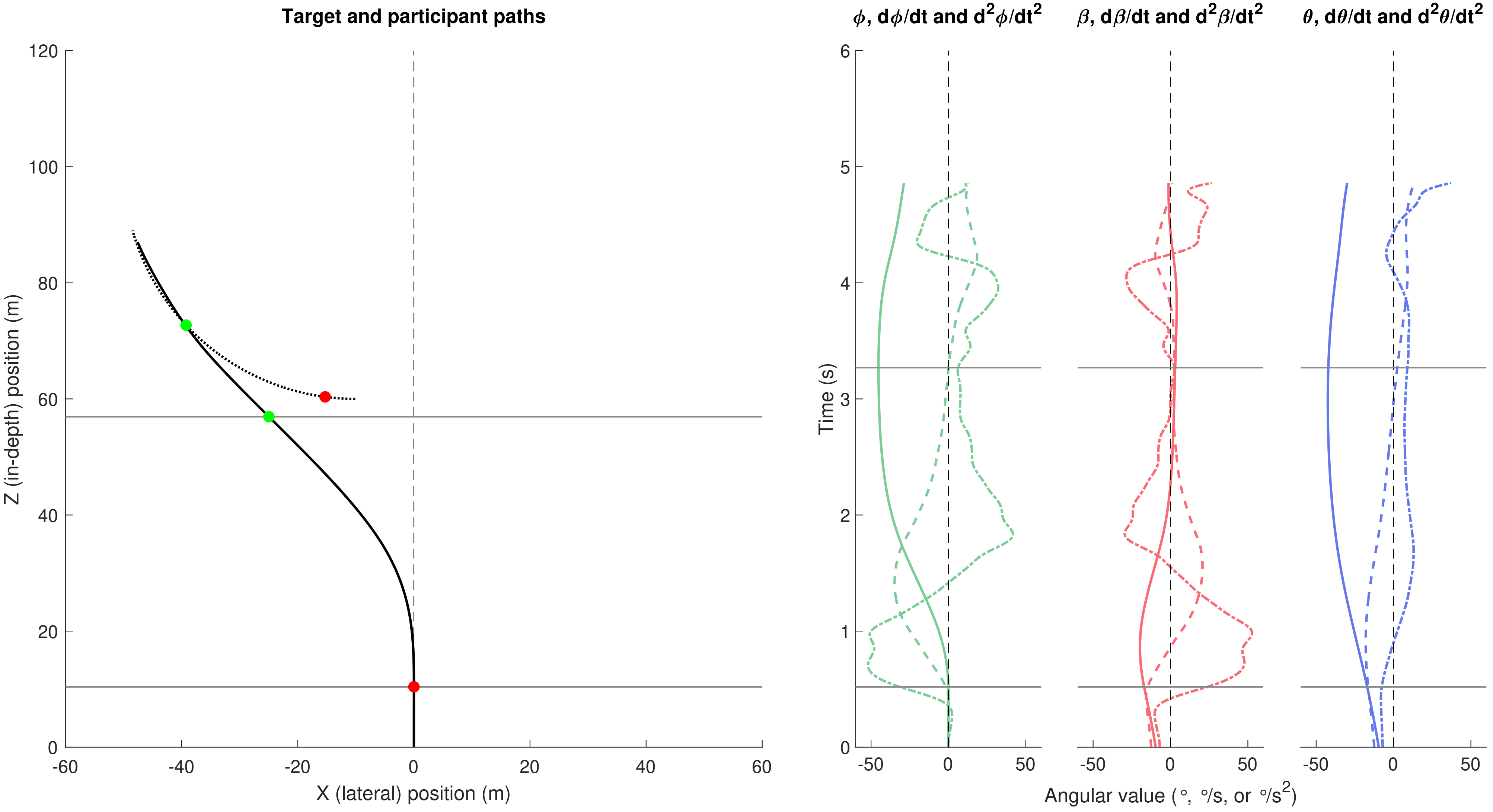

P6/B4
S0/R20-OUT

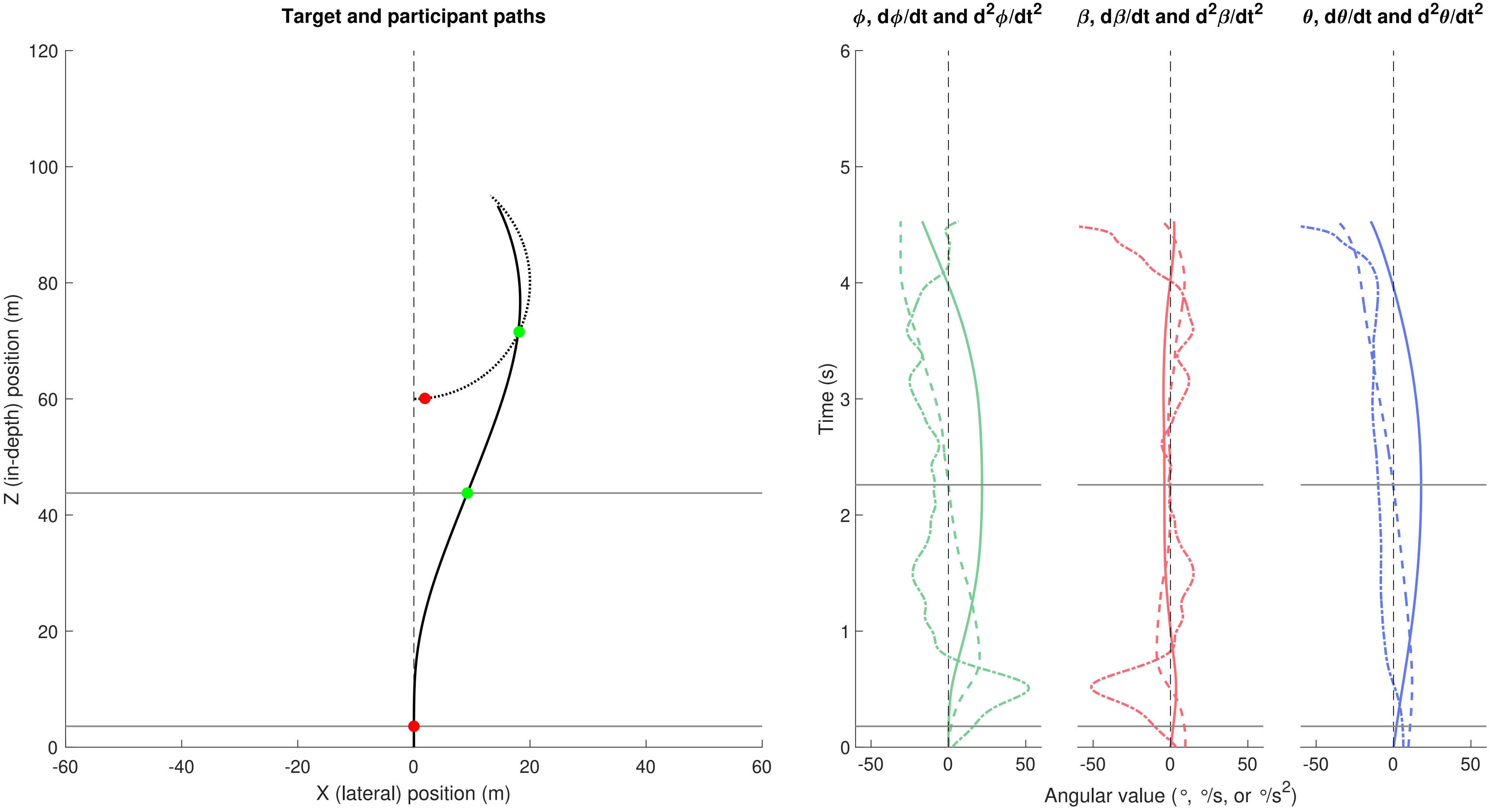

P6/B4
S0/R20-OUT

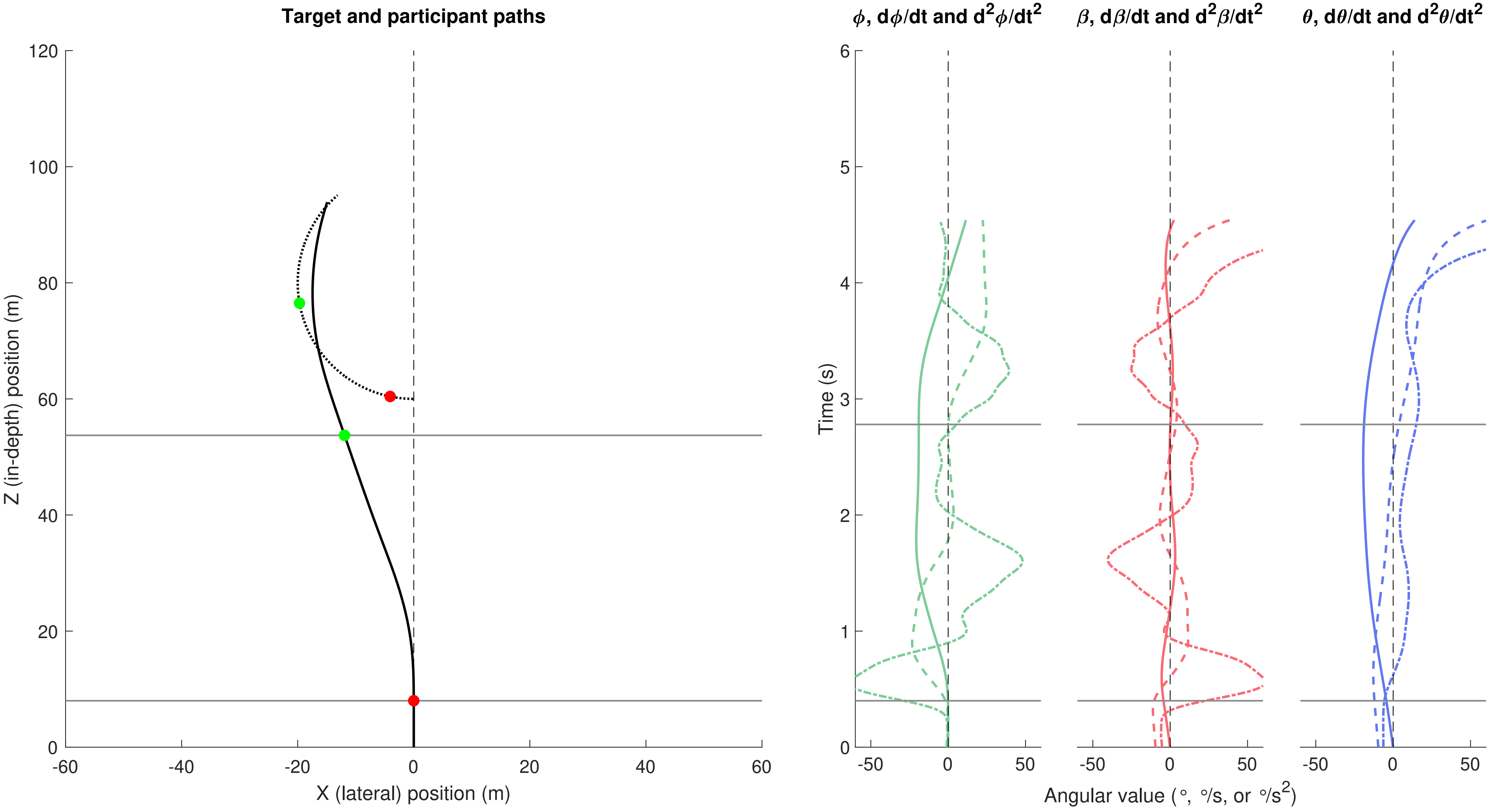

P6/B4
S0/R40-OUT

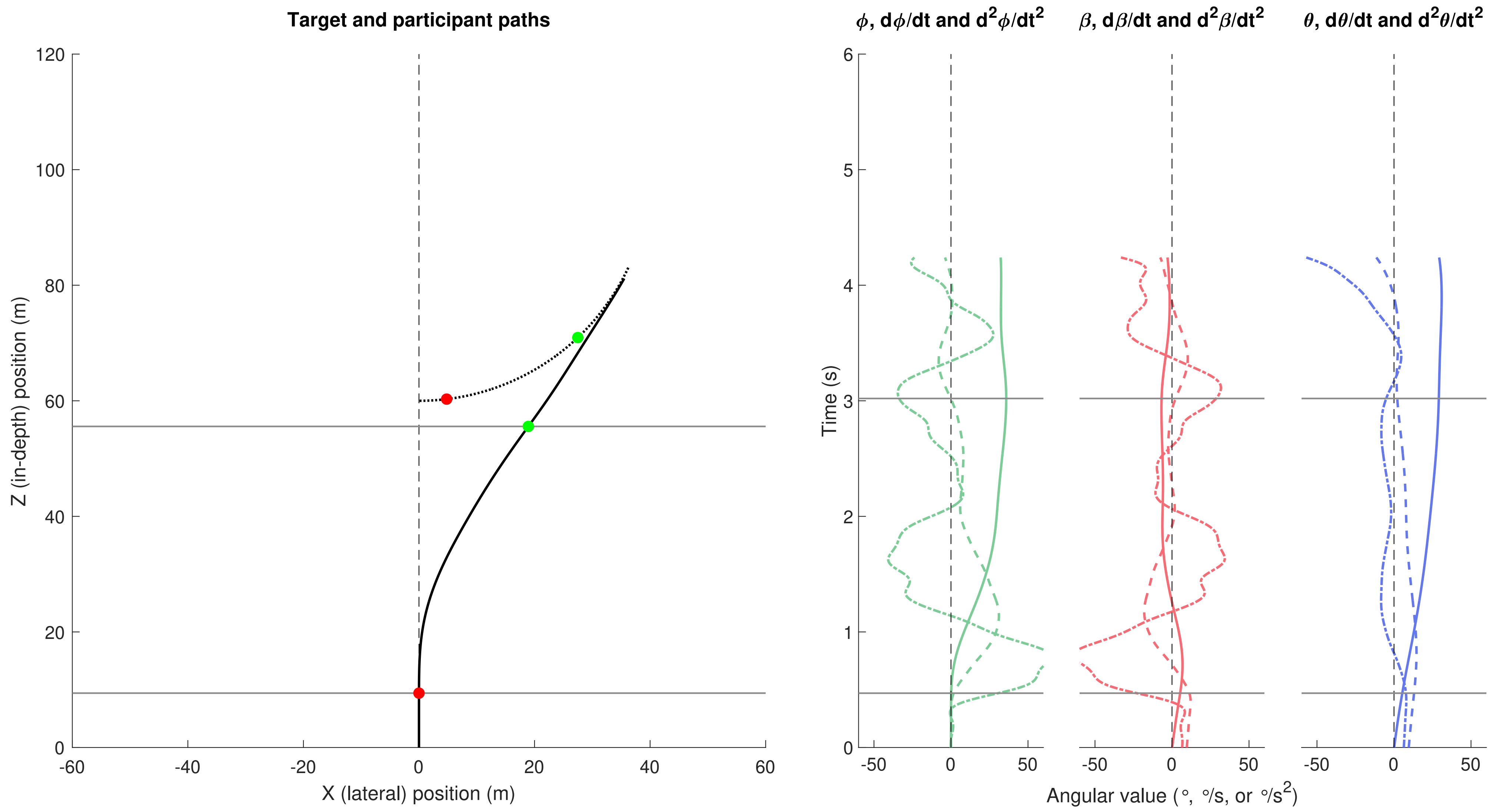

P6/B4
S0/R40-OUT

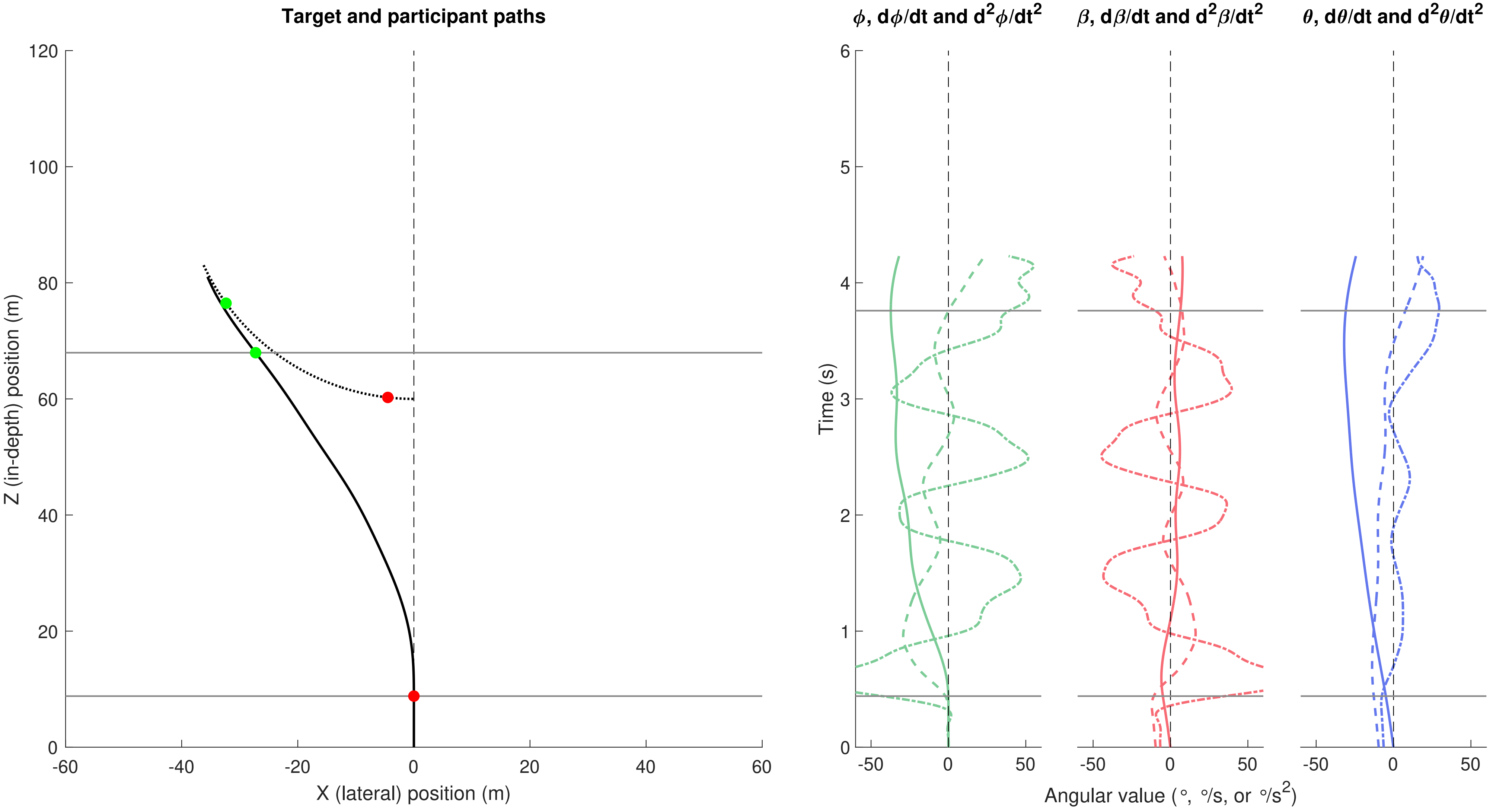

P6/B4
S10/R20-OUT

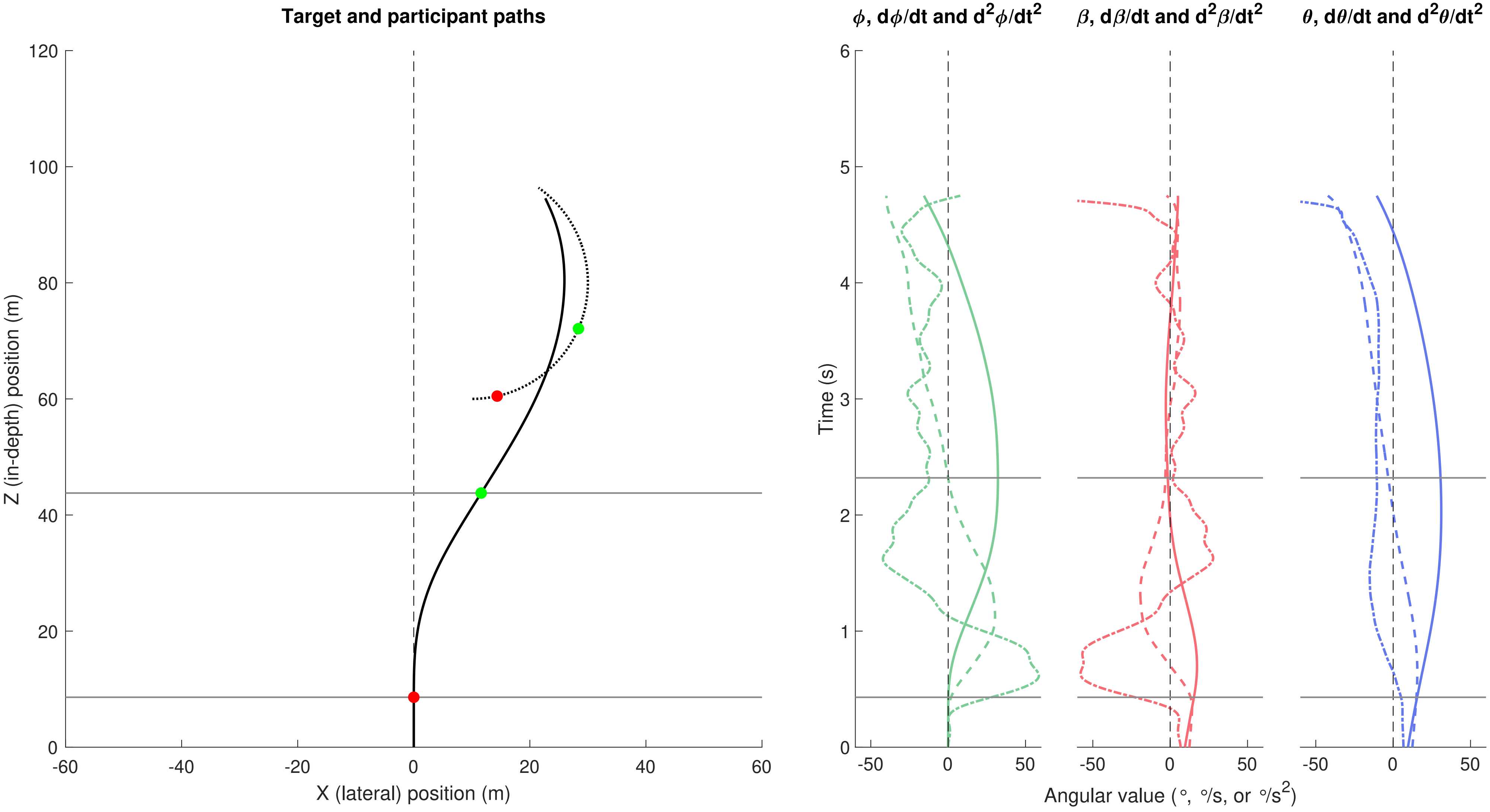

Participant 6

P6/B4
S10/R20-IN

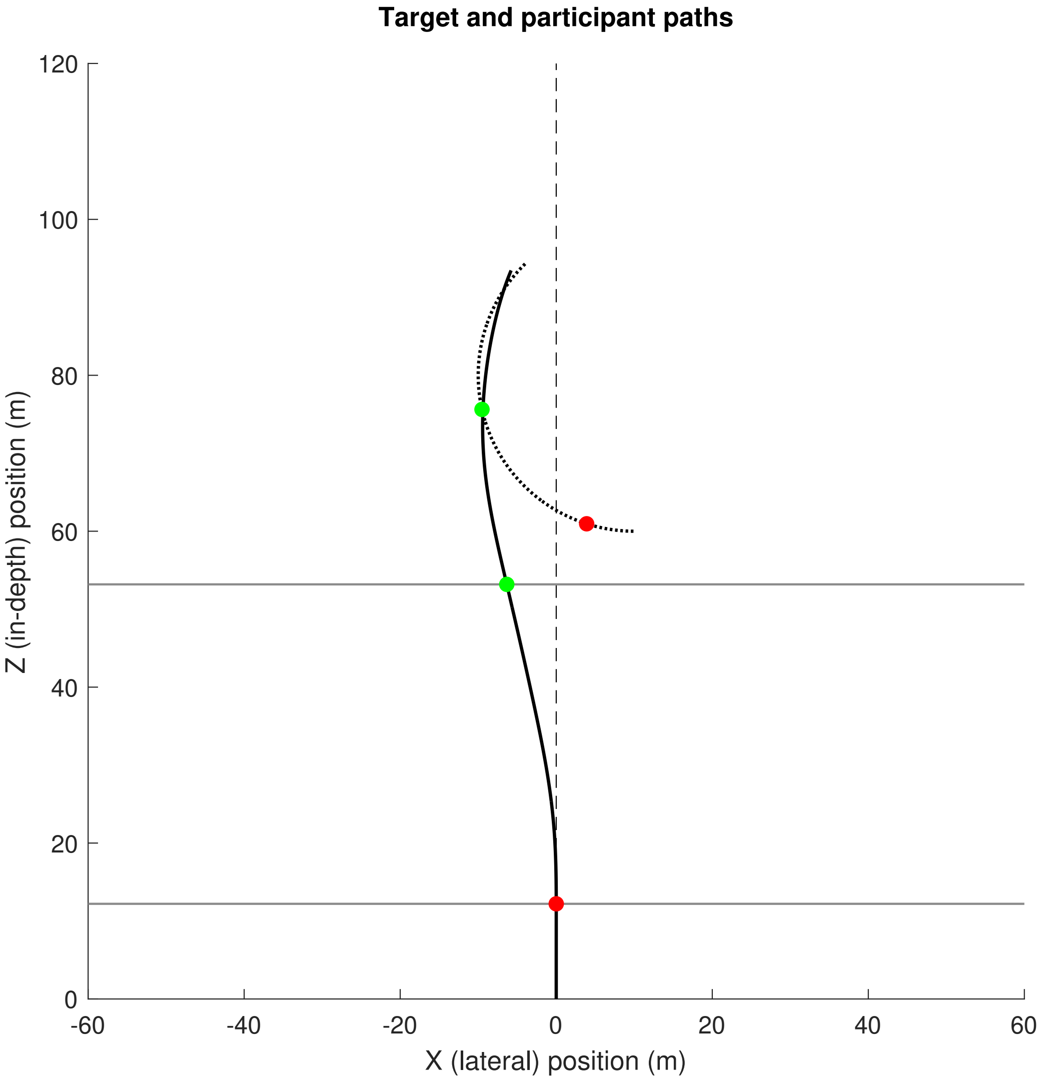

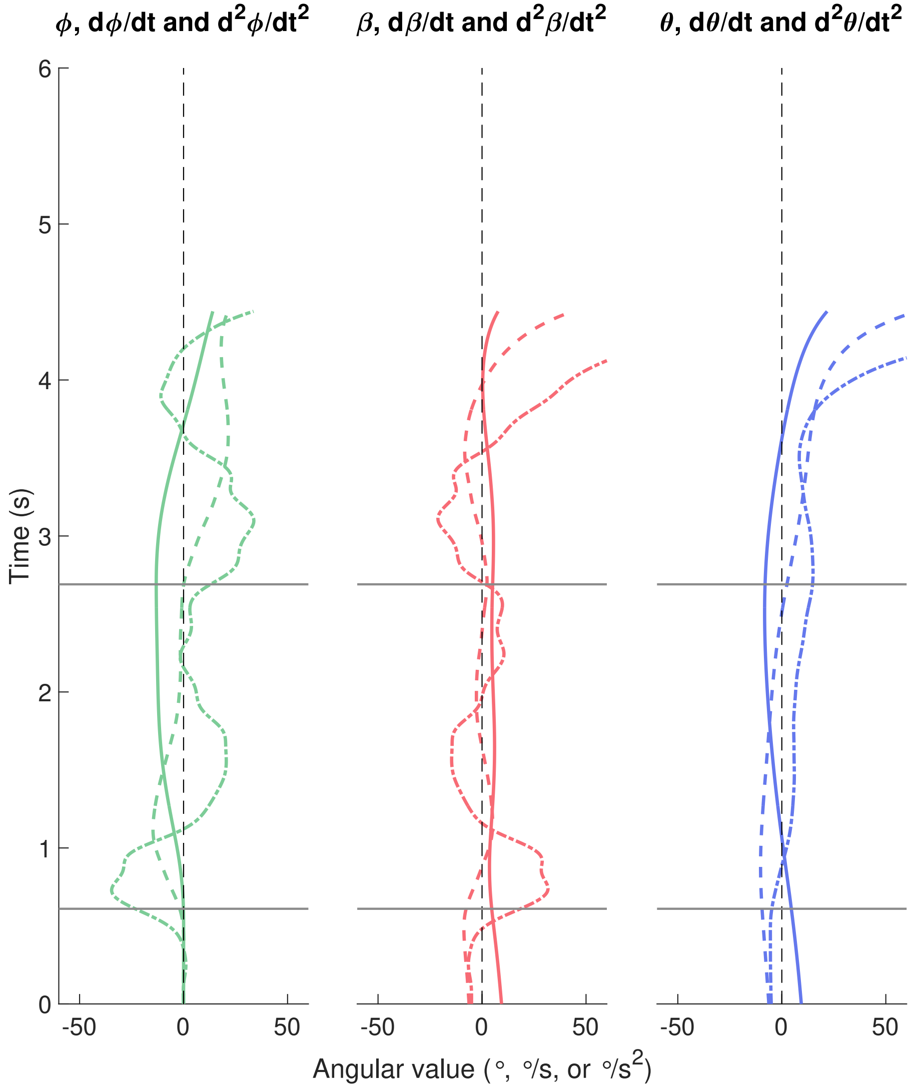

P6/B4
S10/R40-OUT

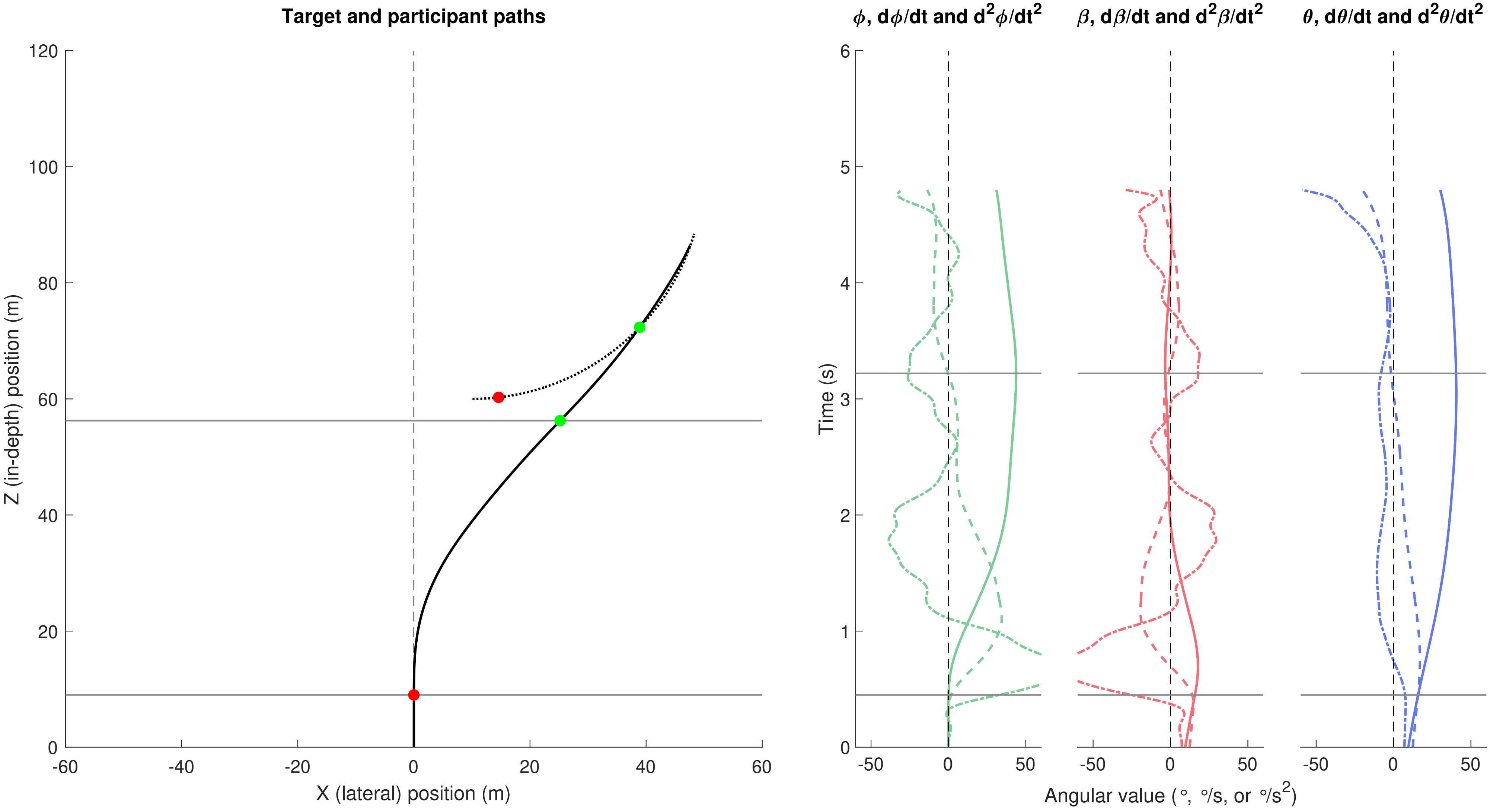

P6/B4
S10/R40-IN

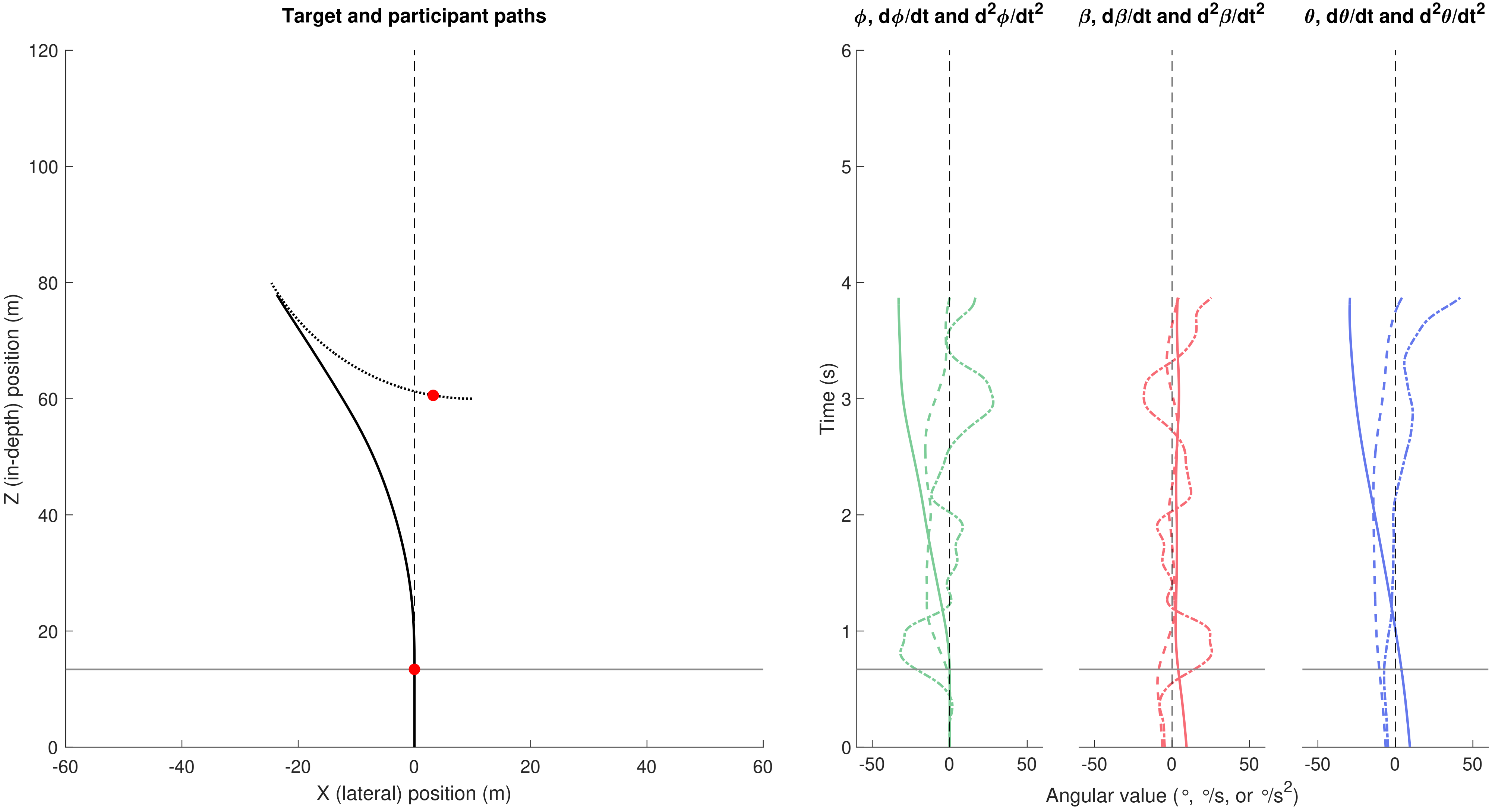

P6/B4
S20/R20-OUT

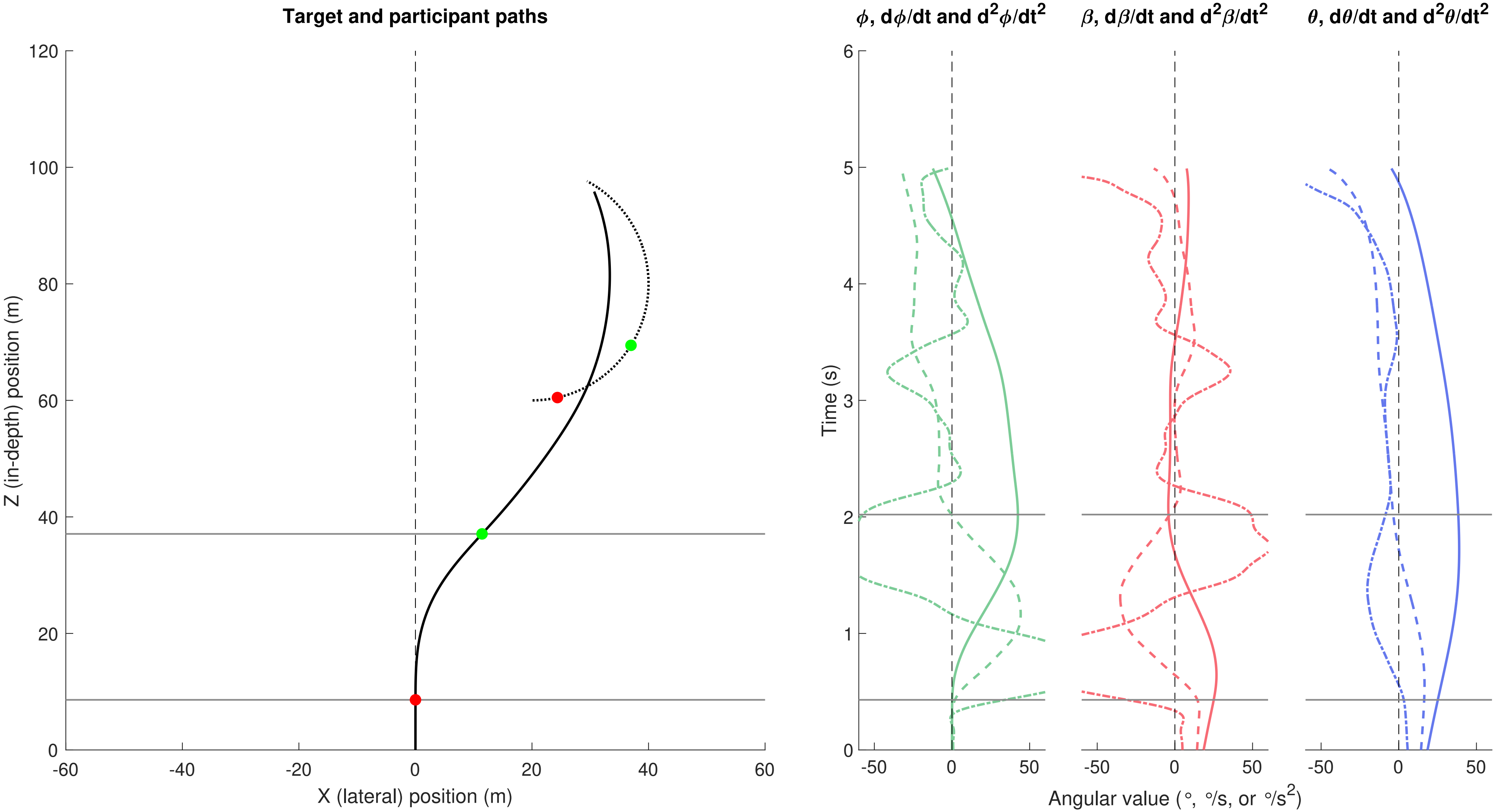

P6/B4
S20/R20-IN

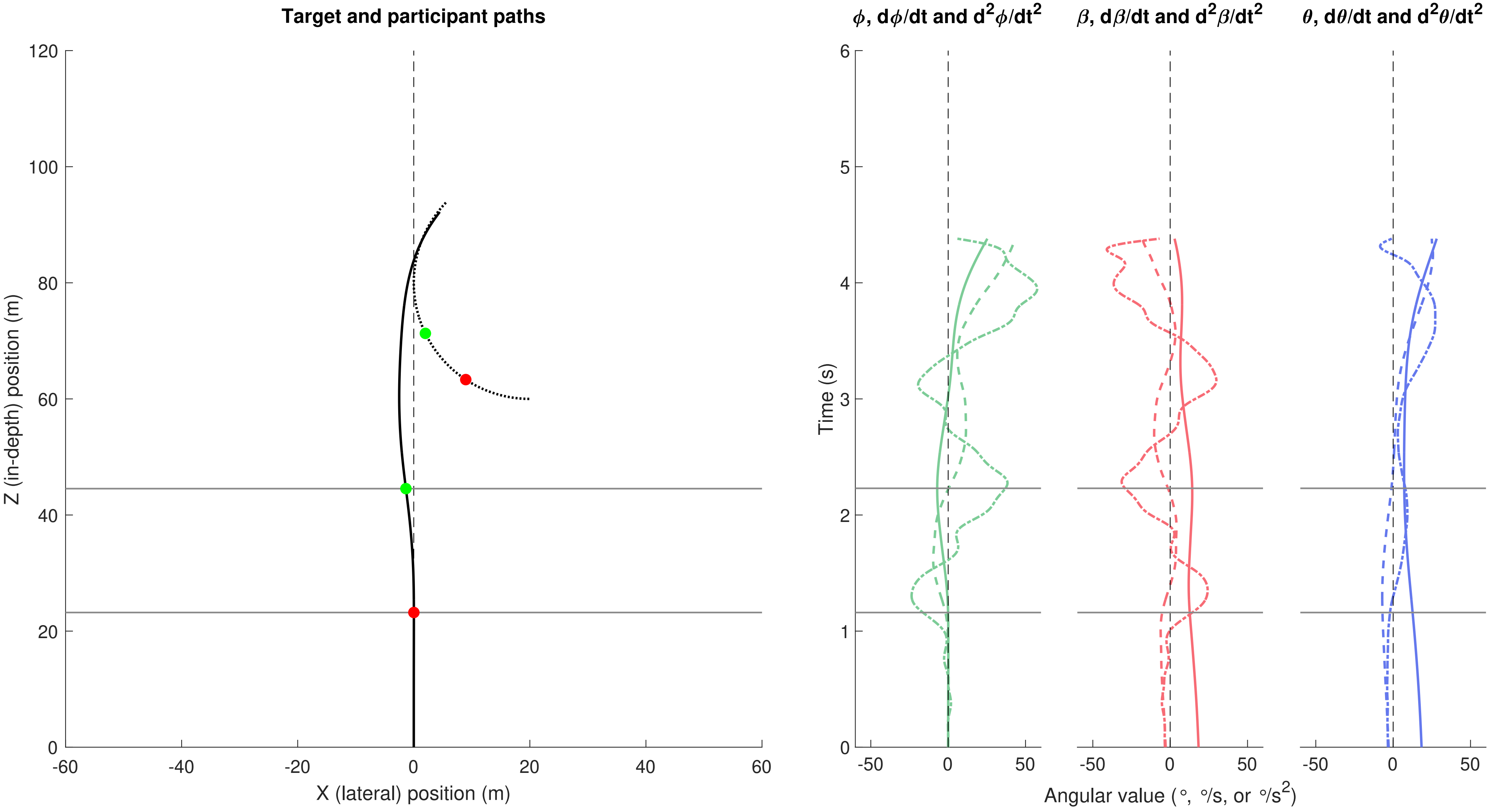

P6/B4
S20/R40-OUT

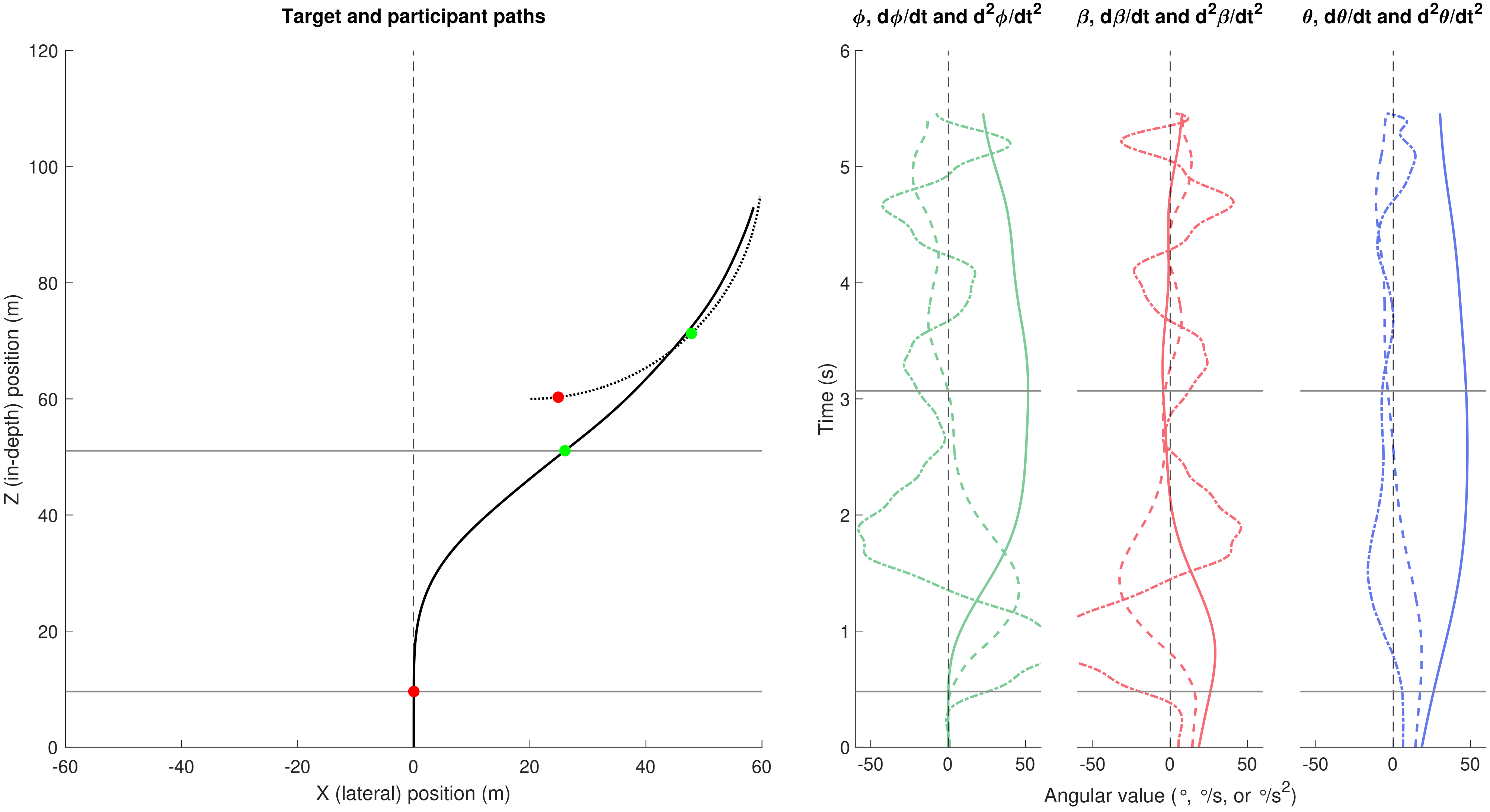

P6/B4
S20/R40-IN

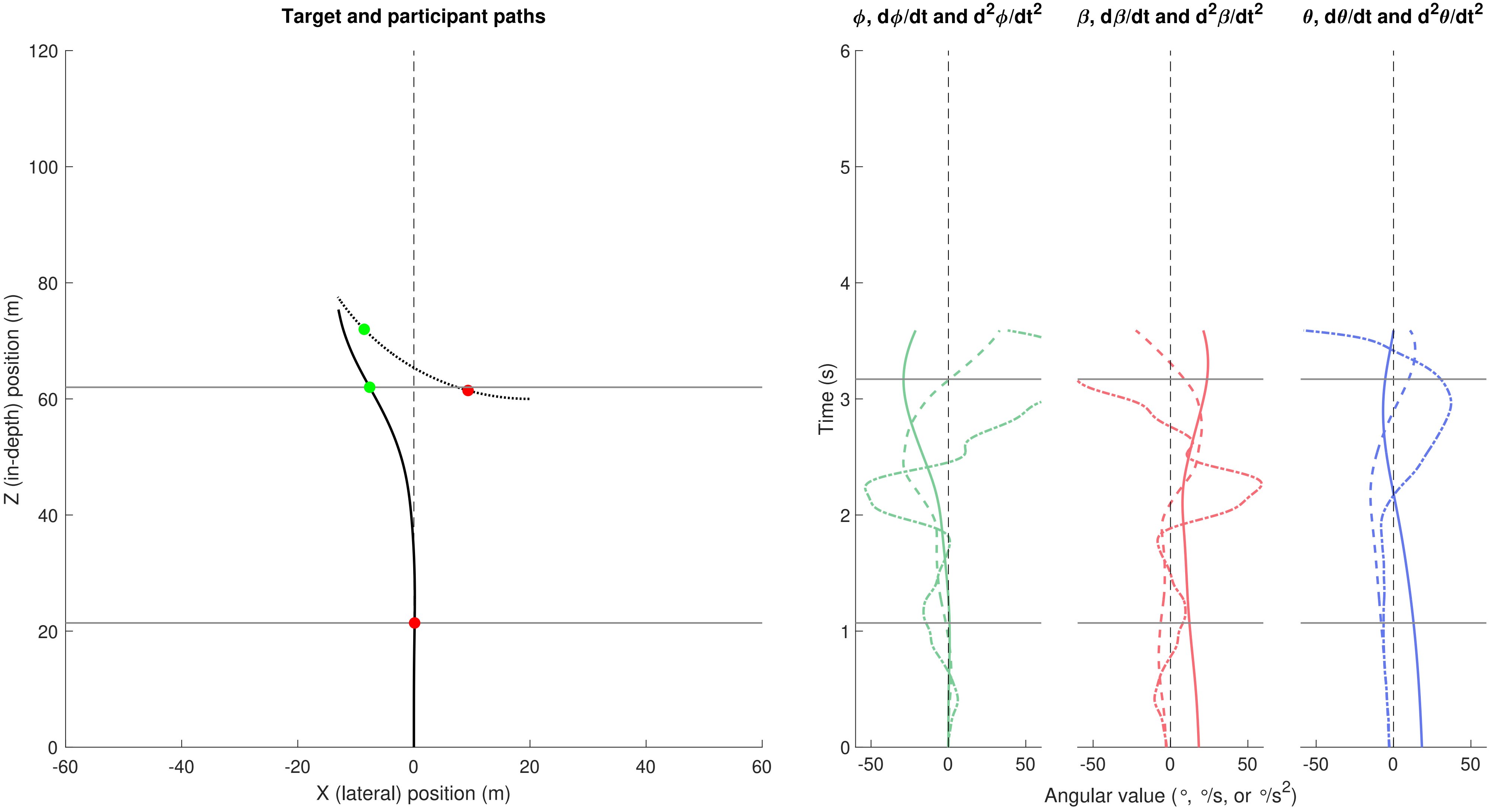

P6/B5
S20/R20-IN

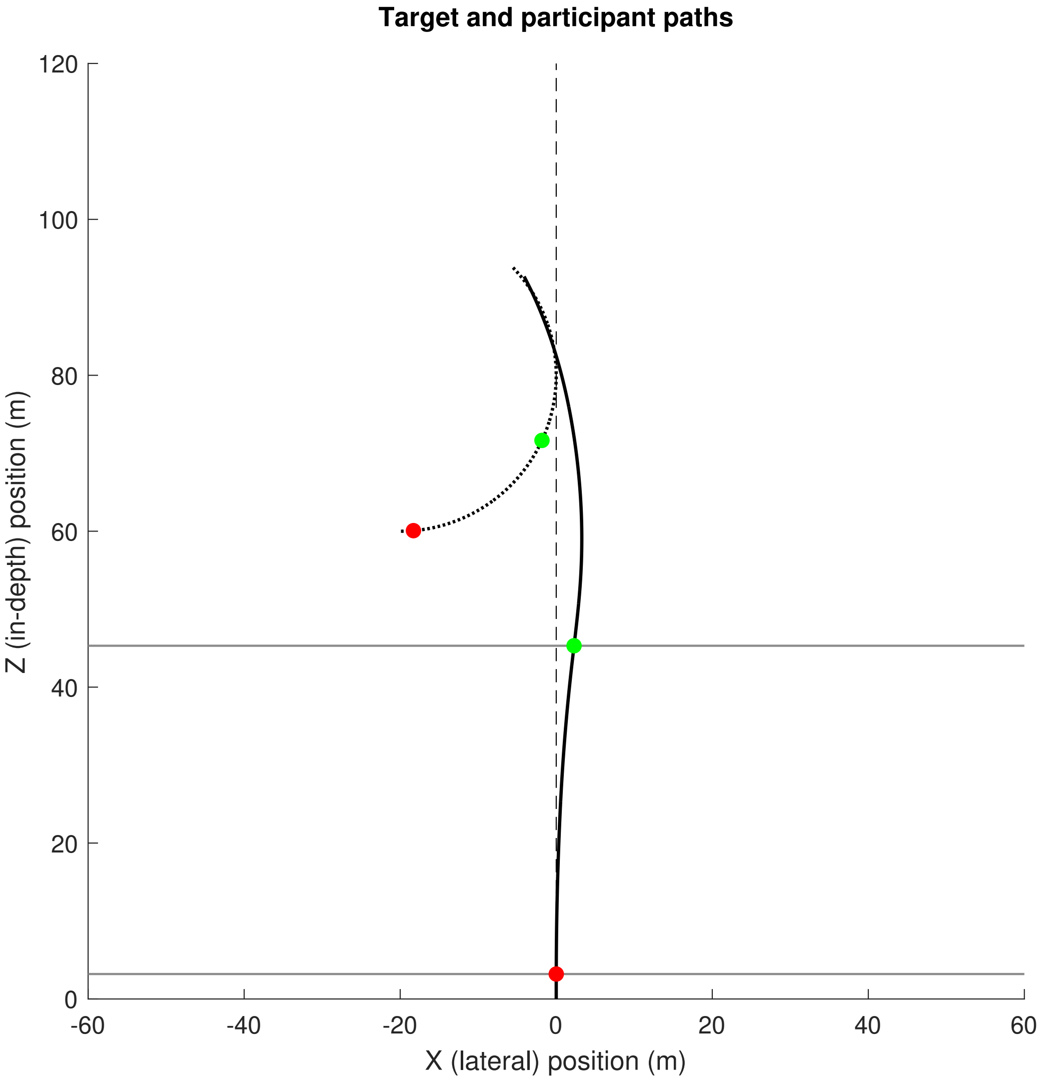

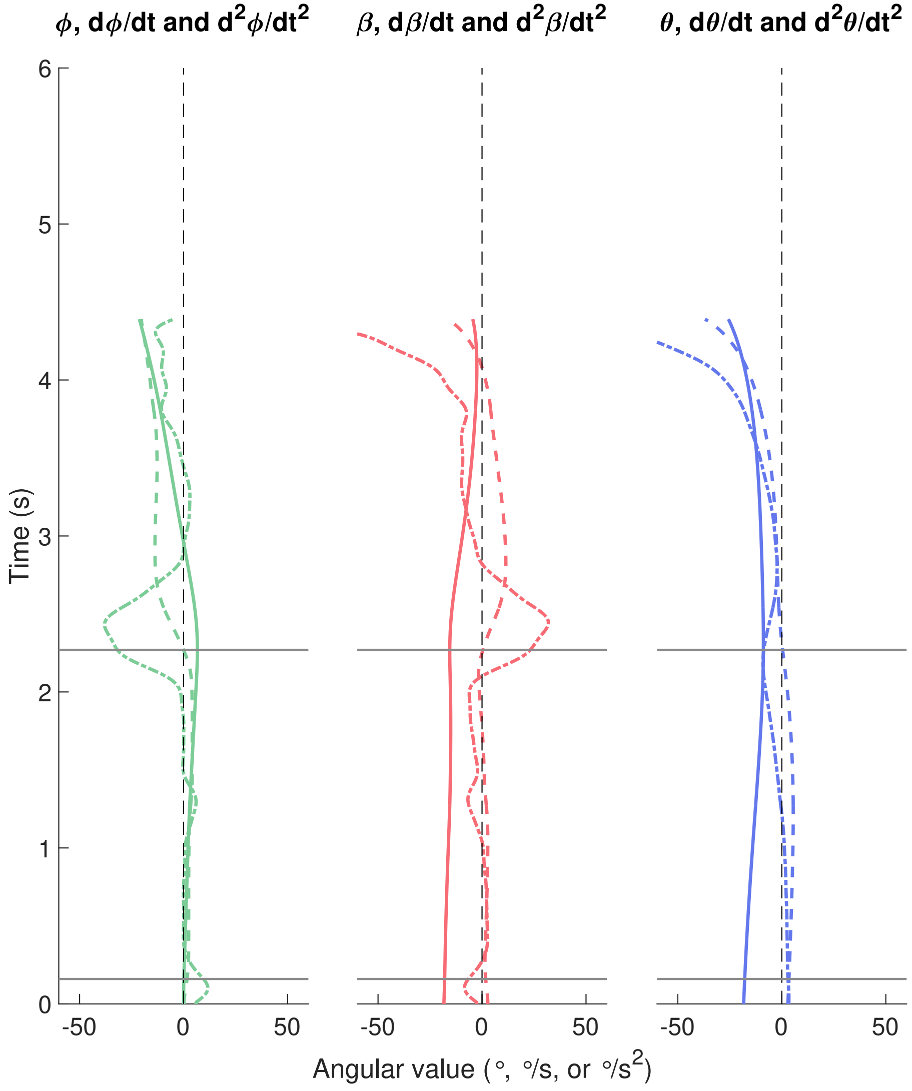

P6/B5
S20/R20-OUT

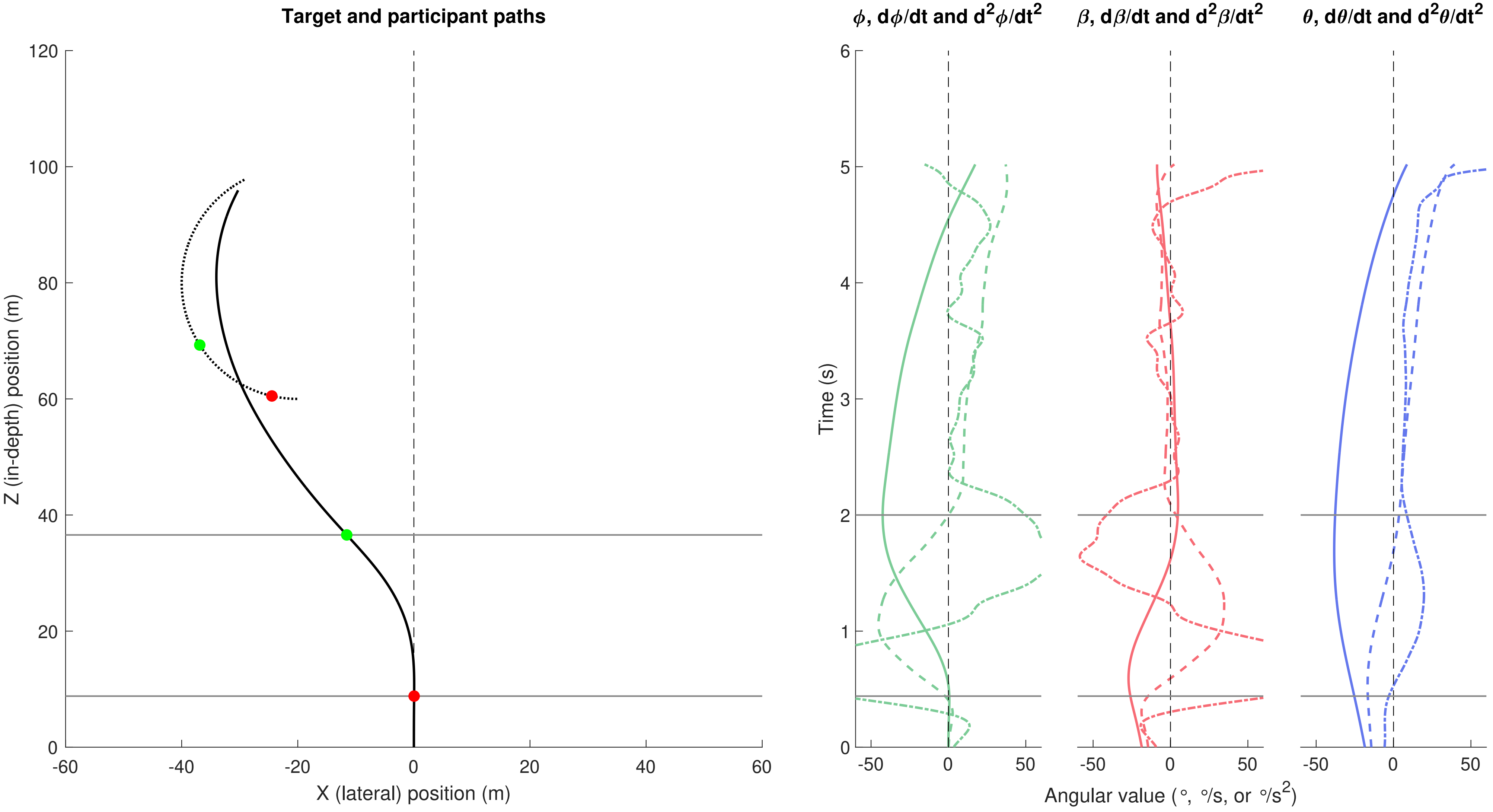

P6/B5
S20/R40-IN

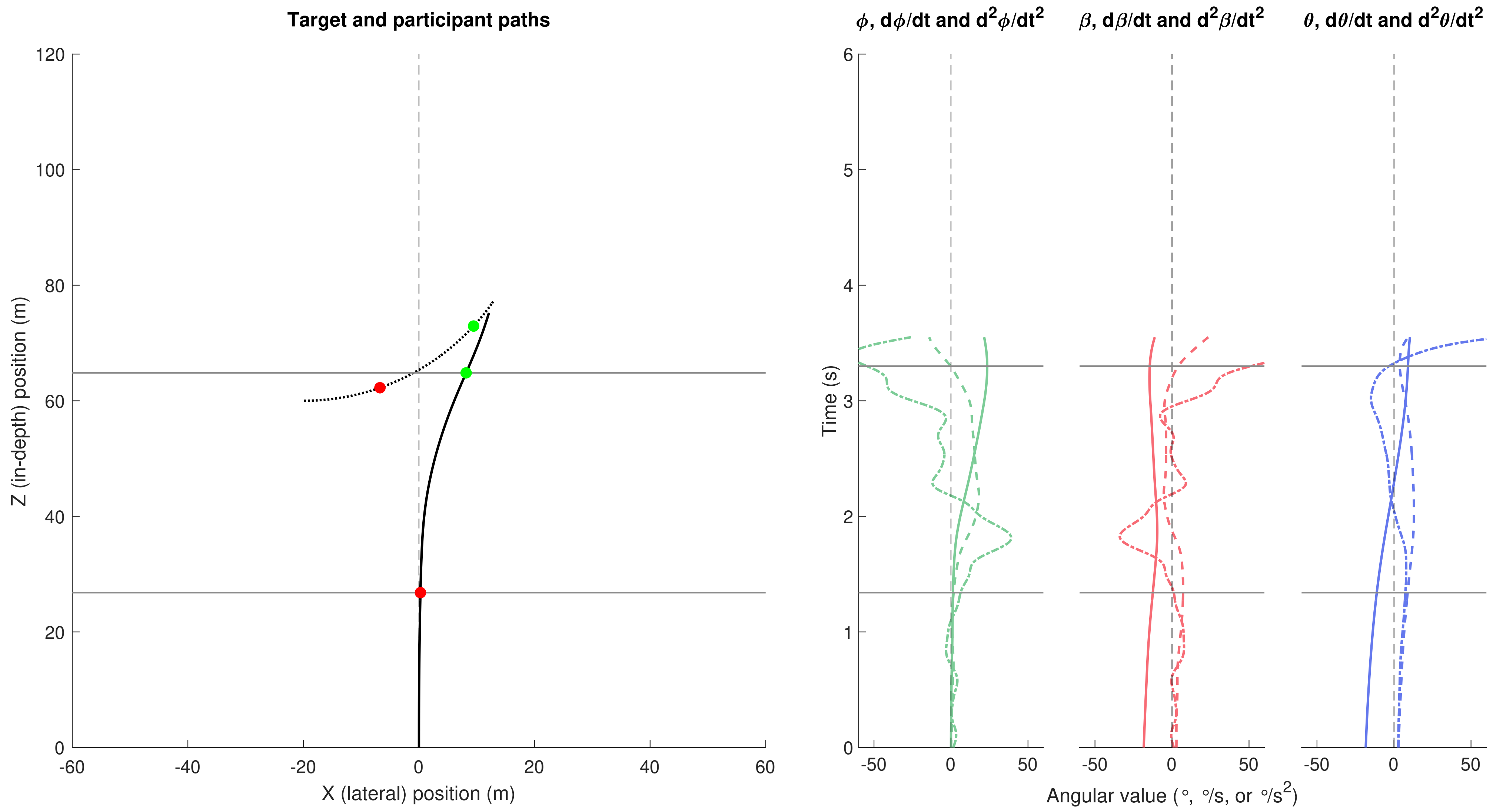

P6/B5
S20/R40-OUT

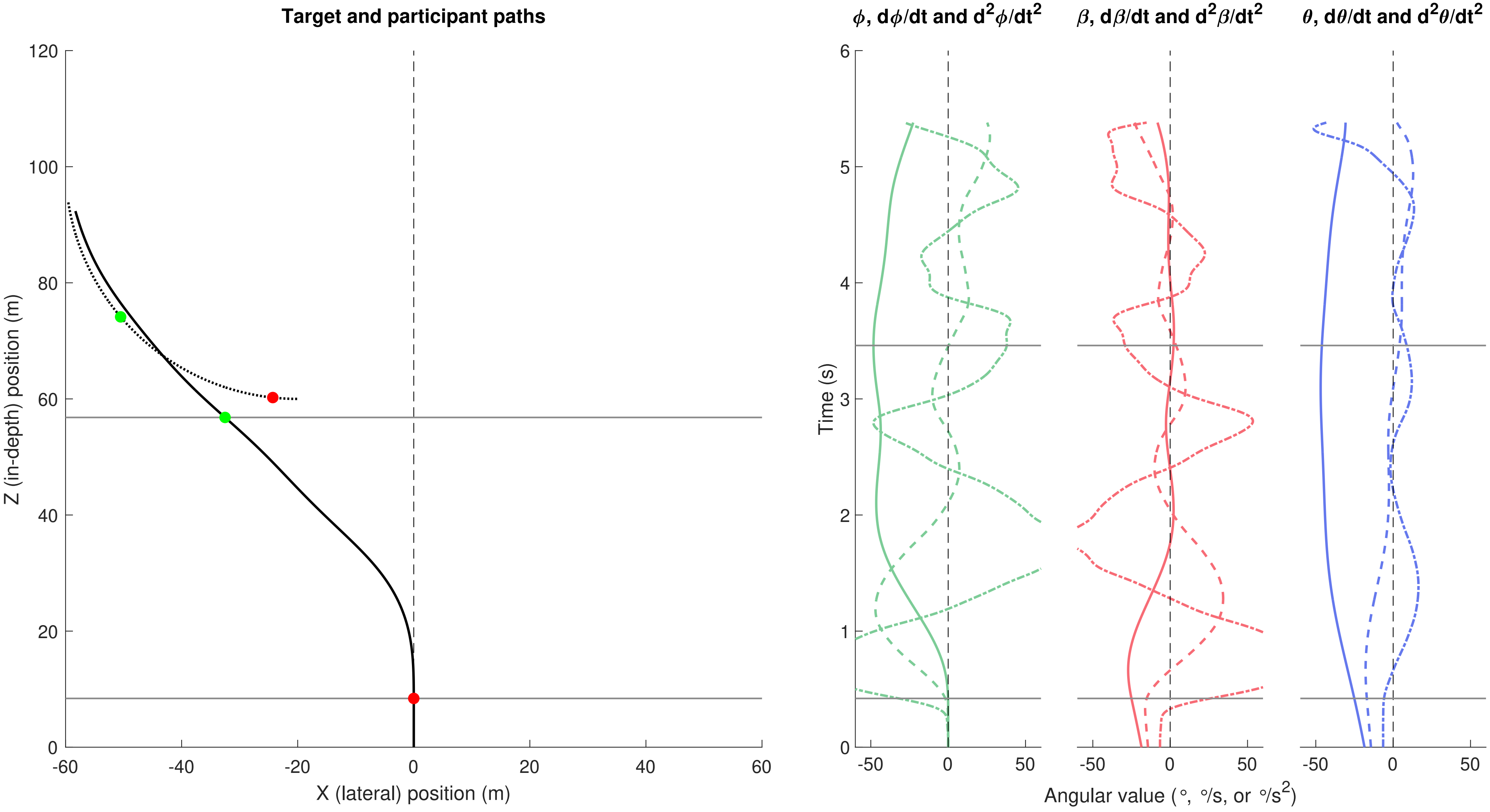

P6/B5
S10/R20-IN

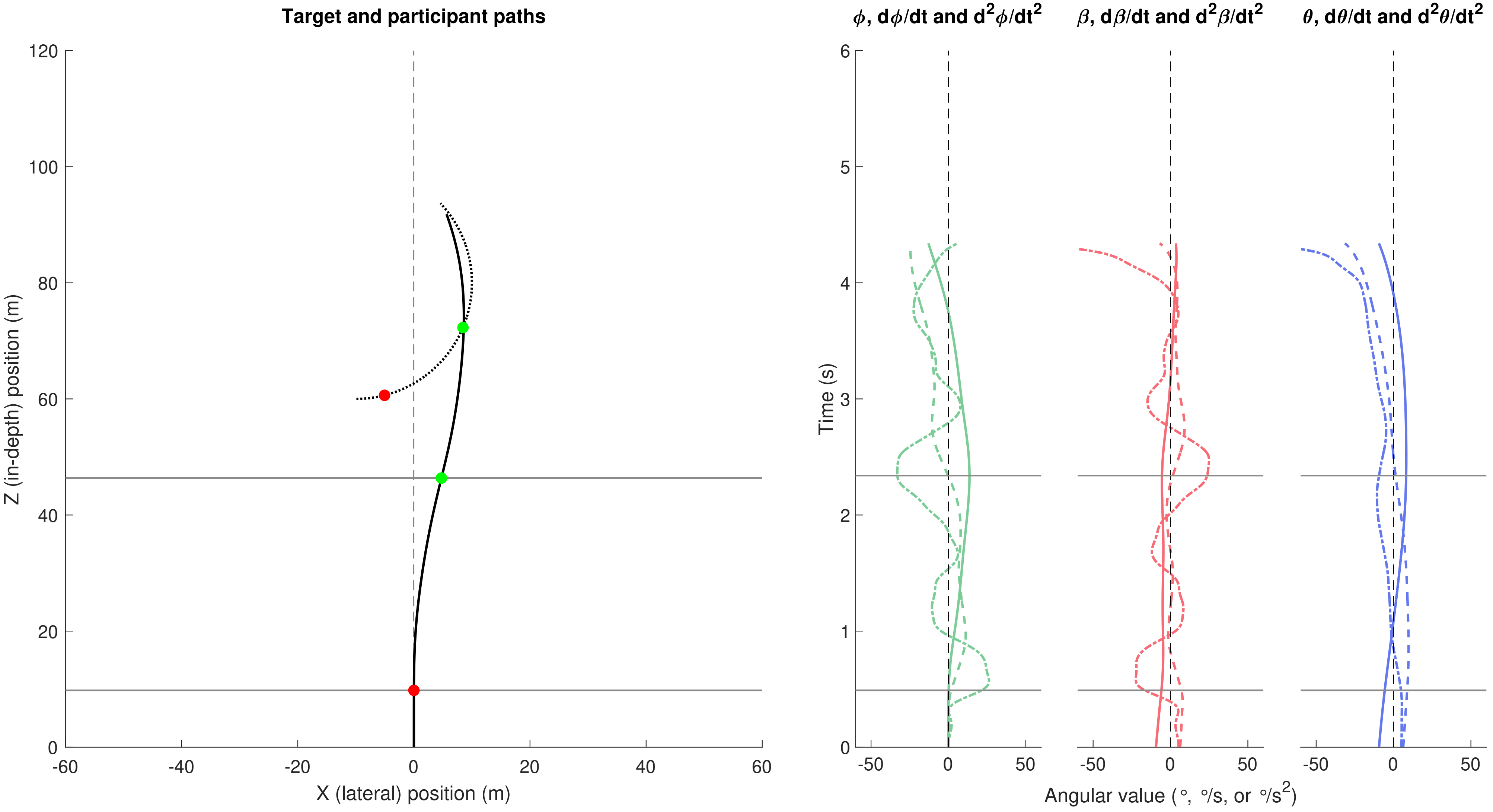

P6/B5
S10/R20-OUT

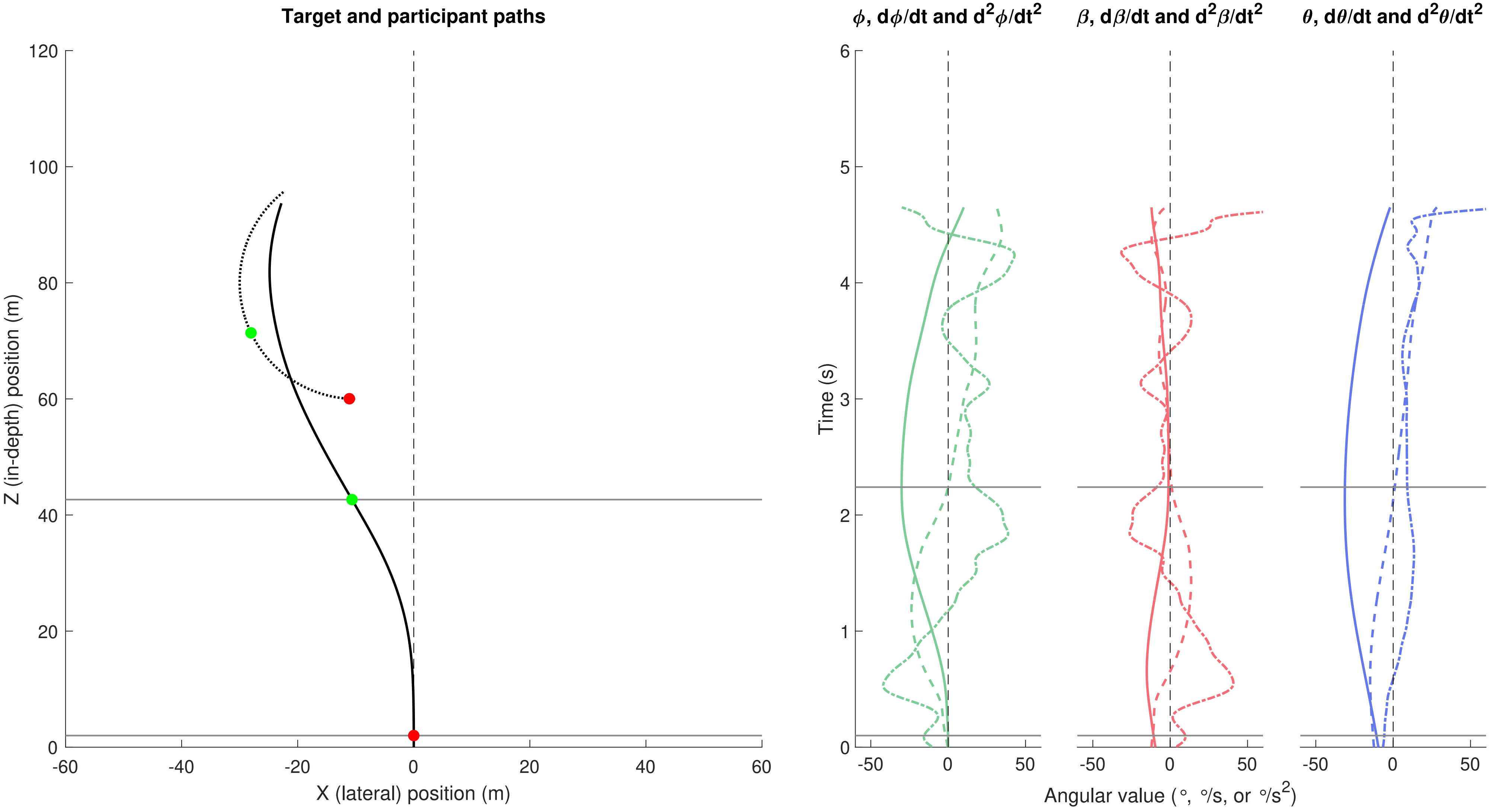

P6/B5
S10/R40-IN

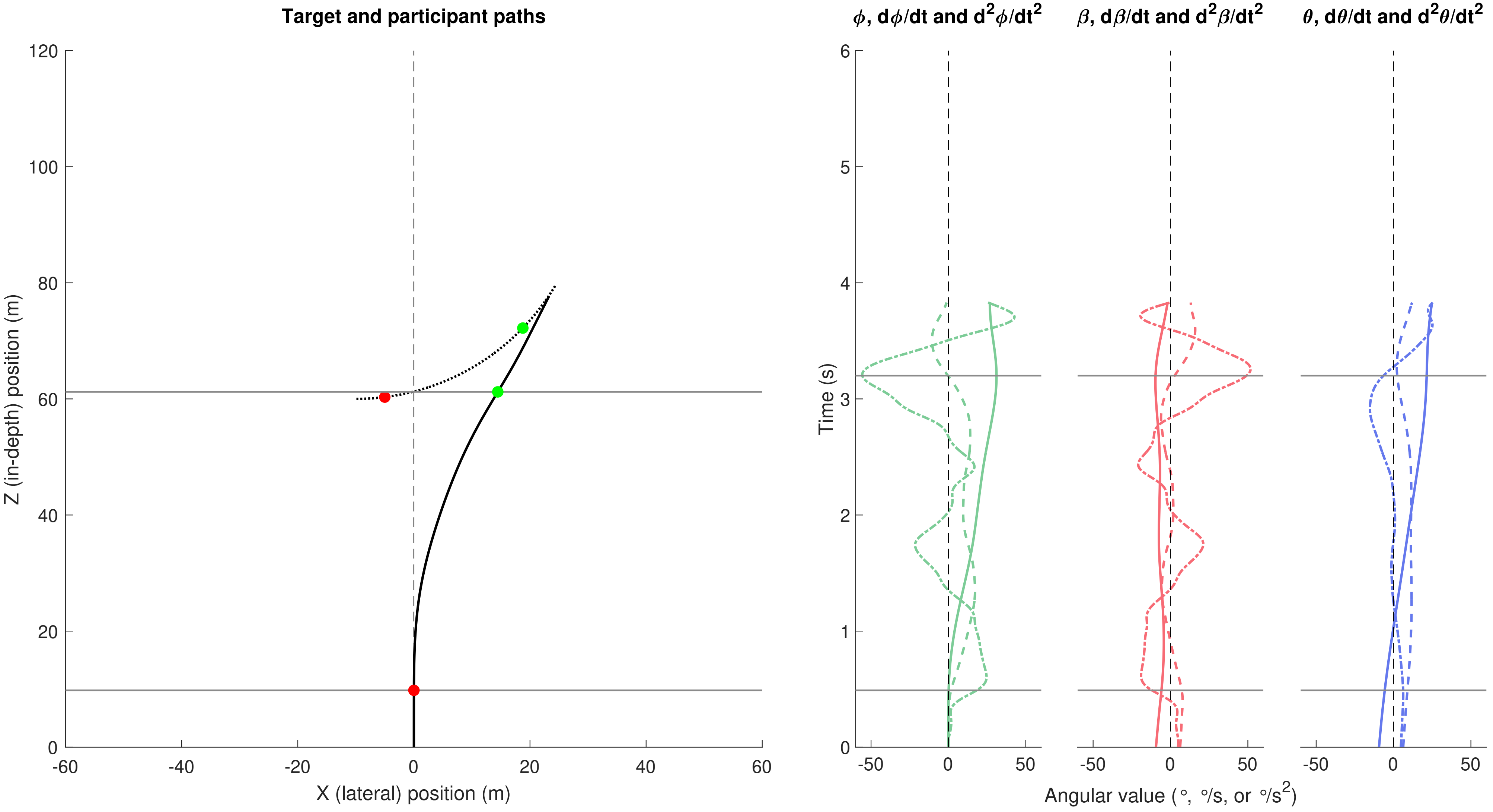

P6/B5
S10/R40-OUT

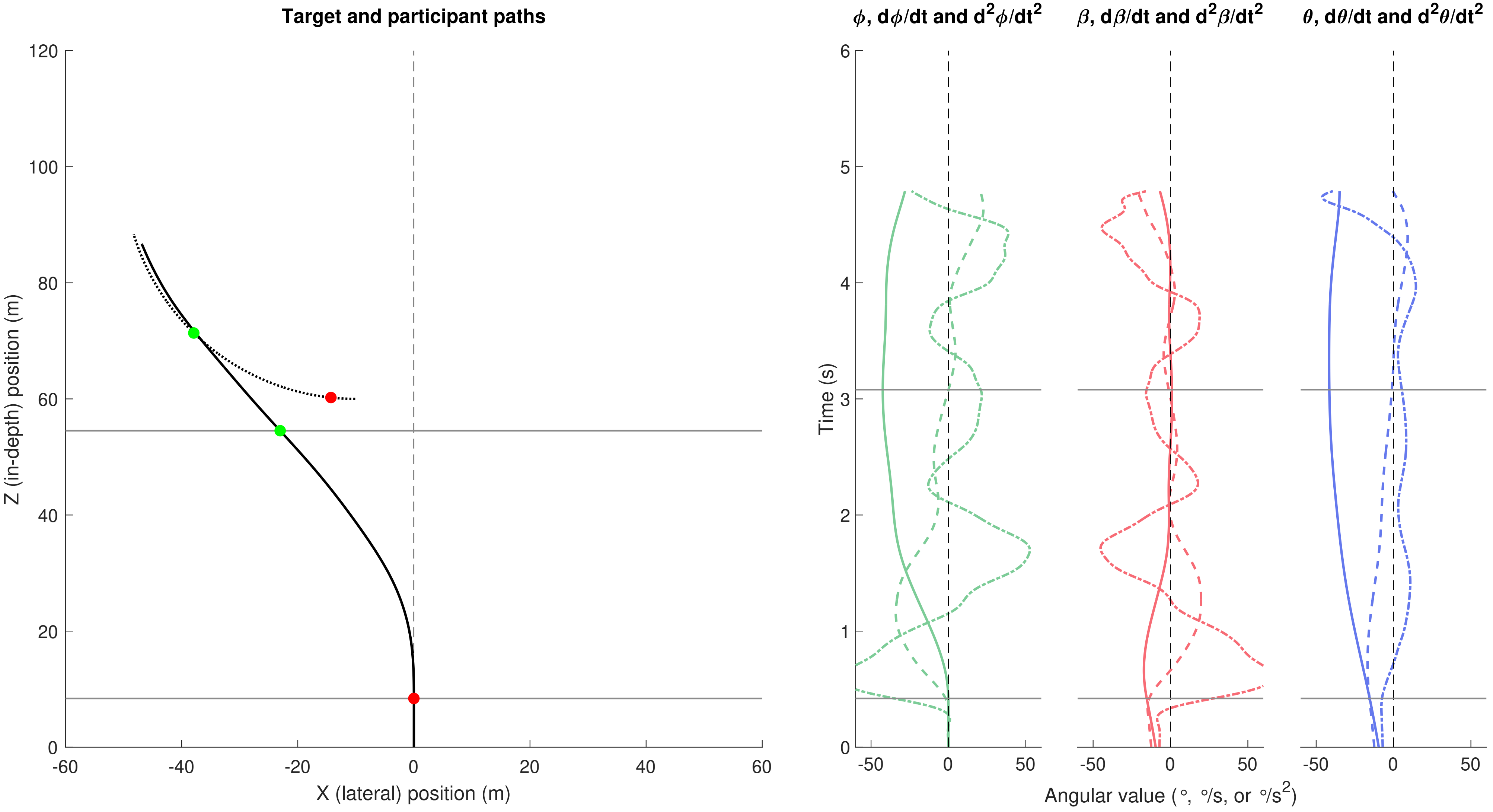

P6/B5
S0/R20-OUT

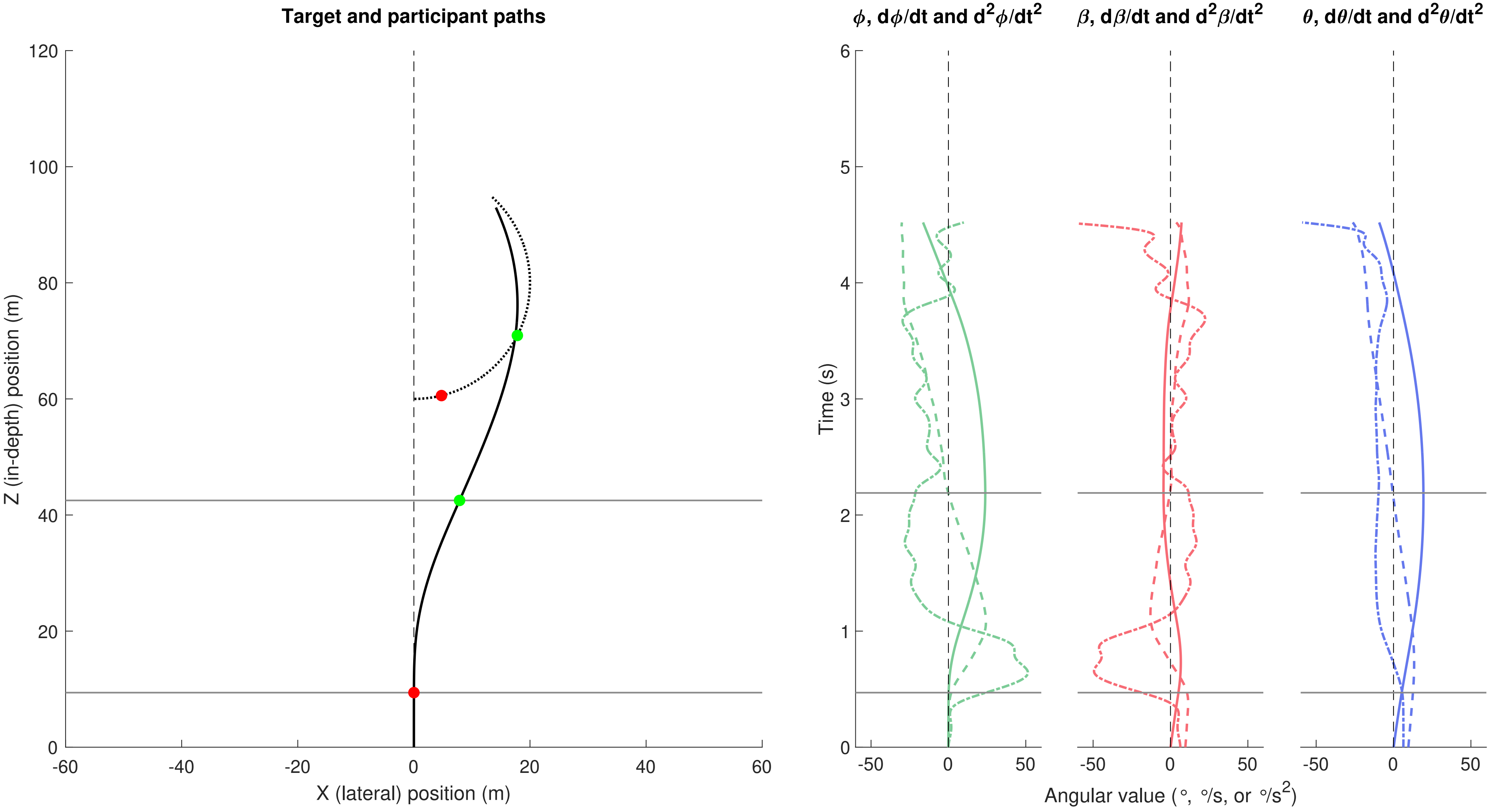

P6/B5
S0/R20-OUT

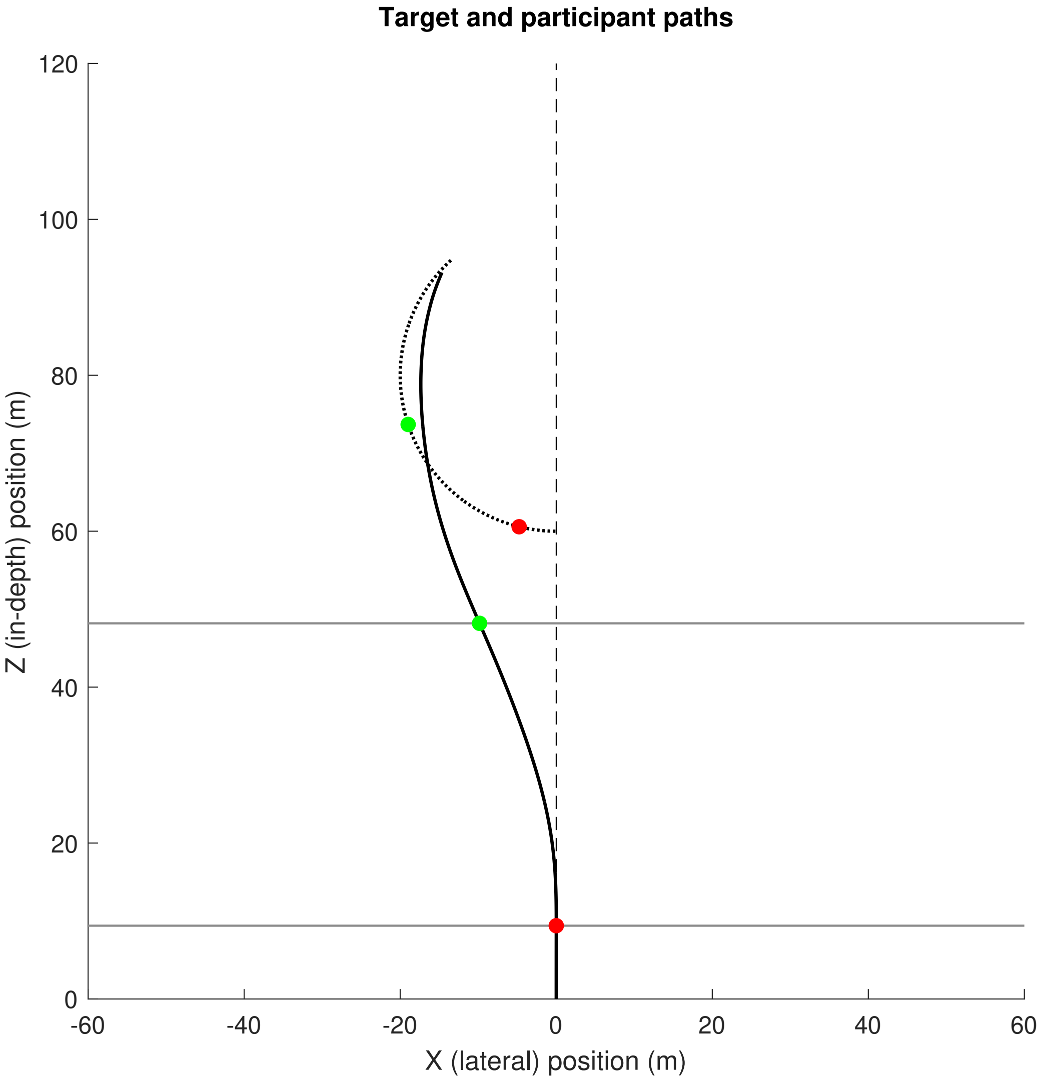

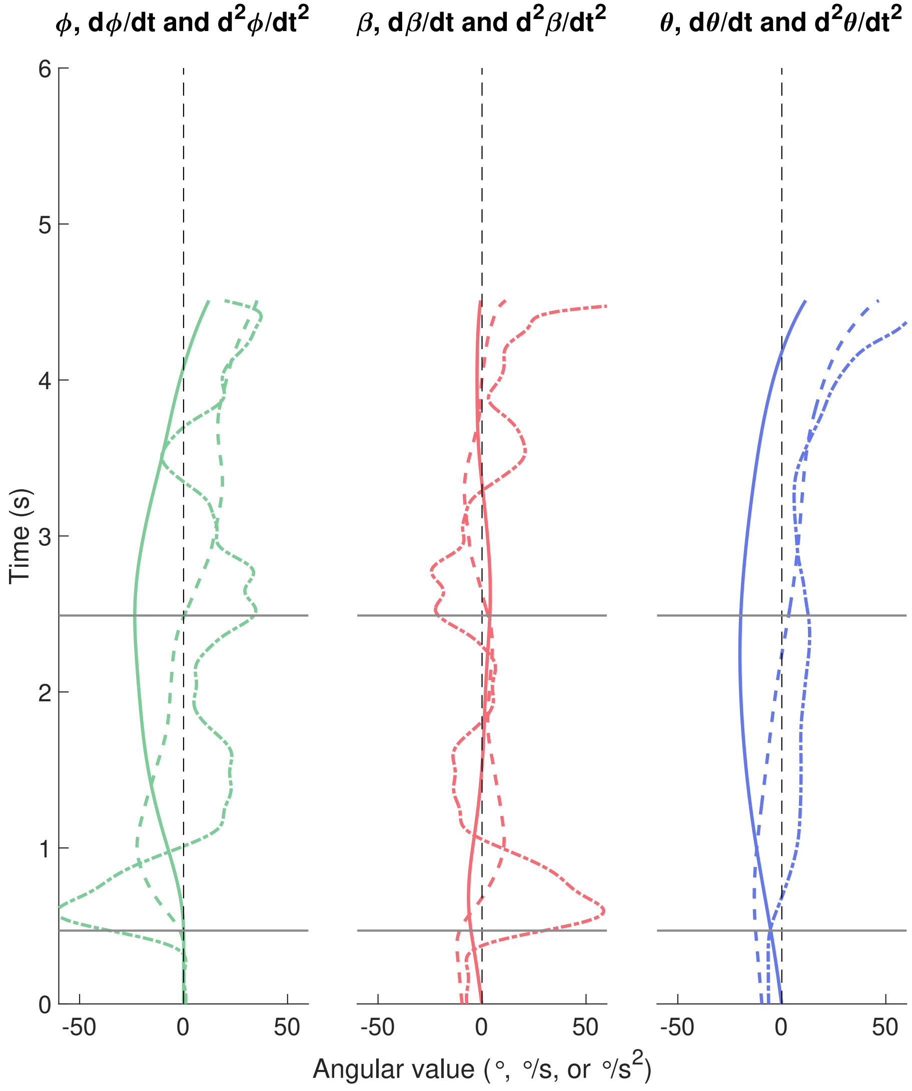

P6/B5
S0/R40-OUT

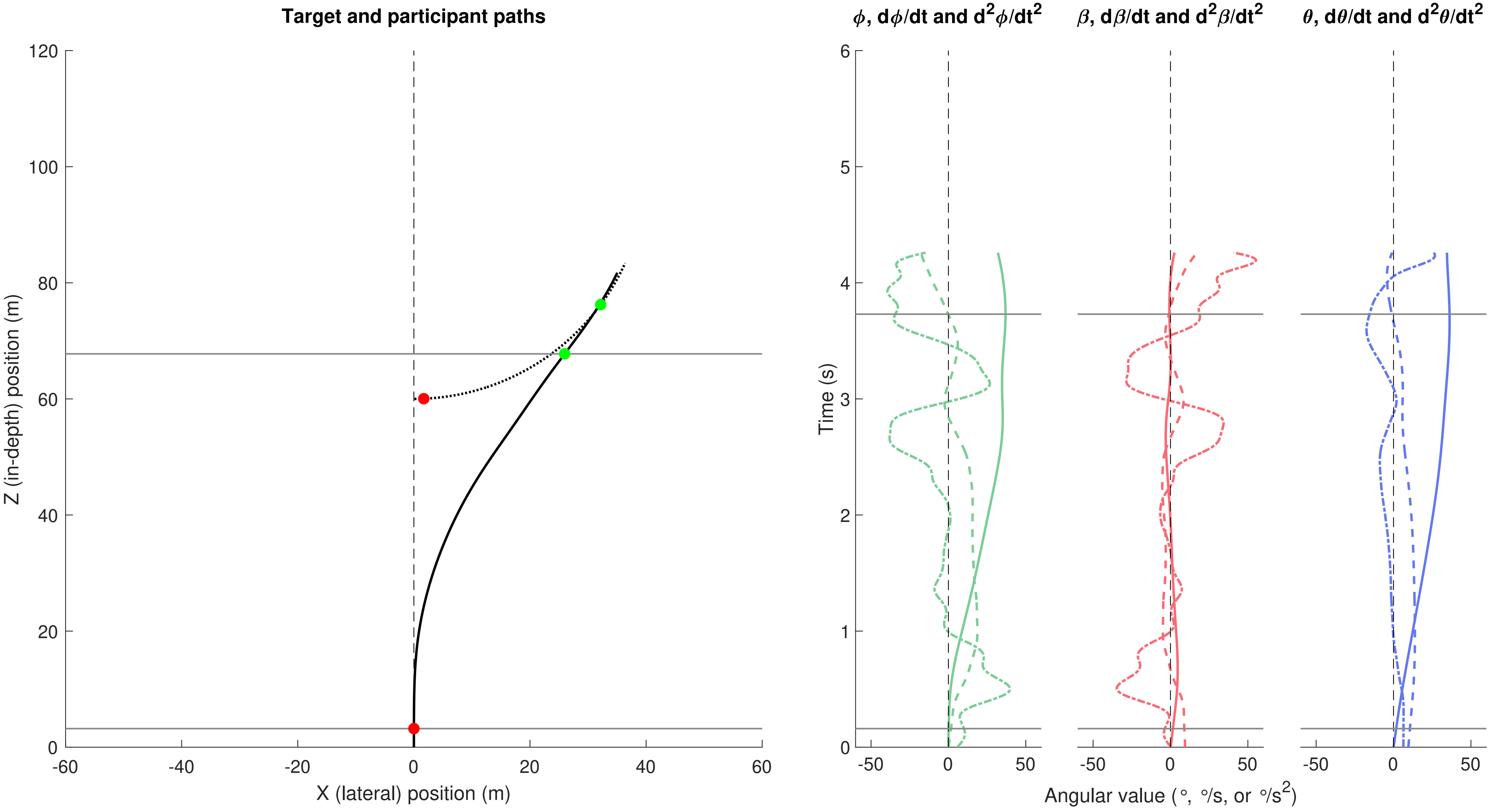

P6/B5
S0/R40-OUT

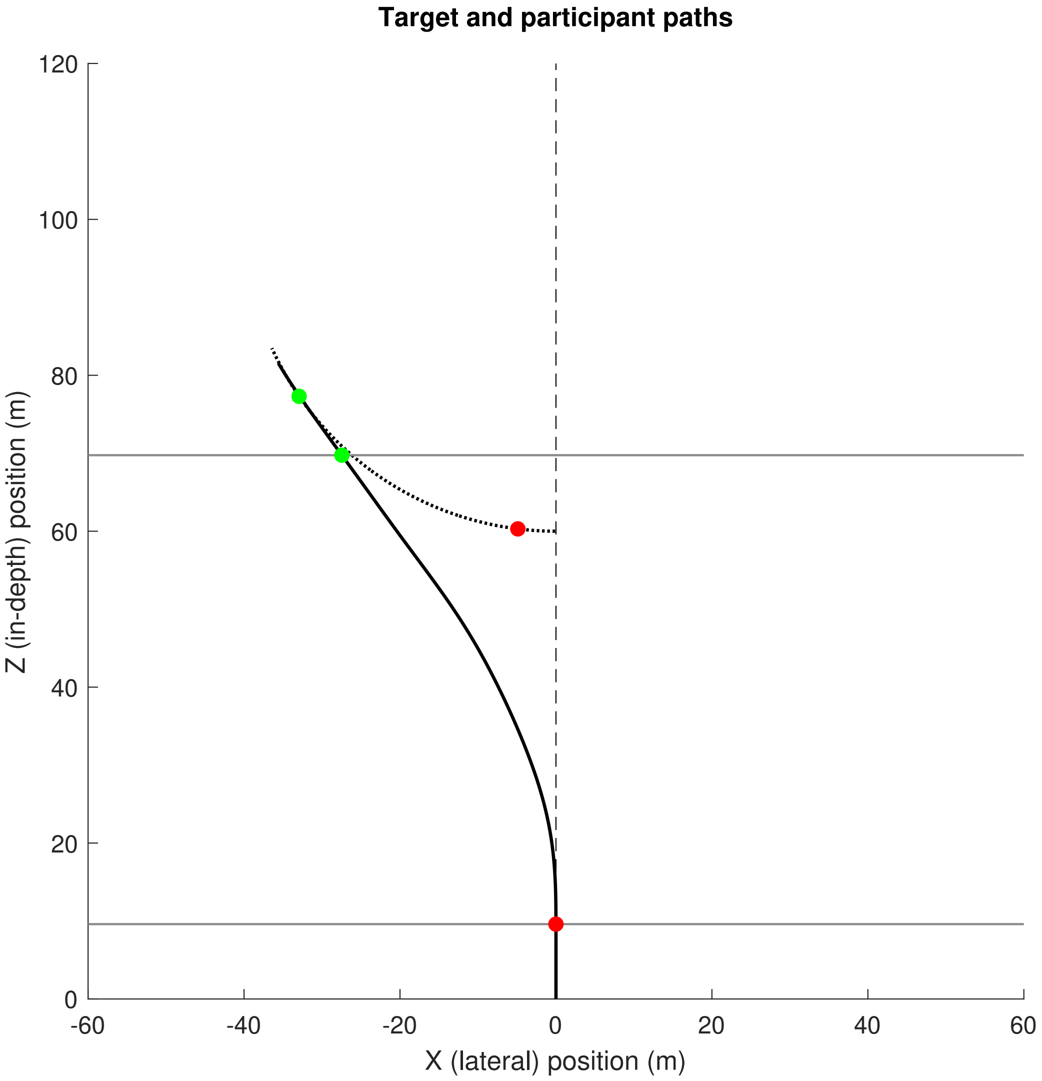

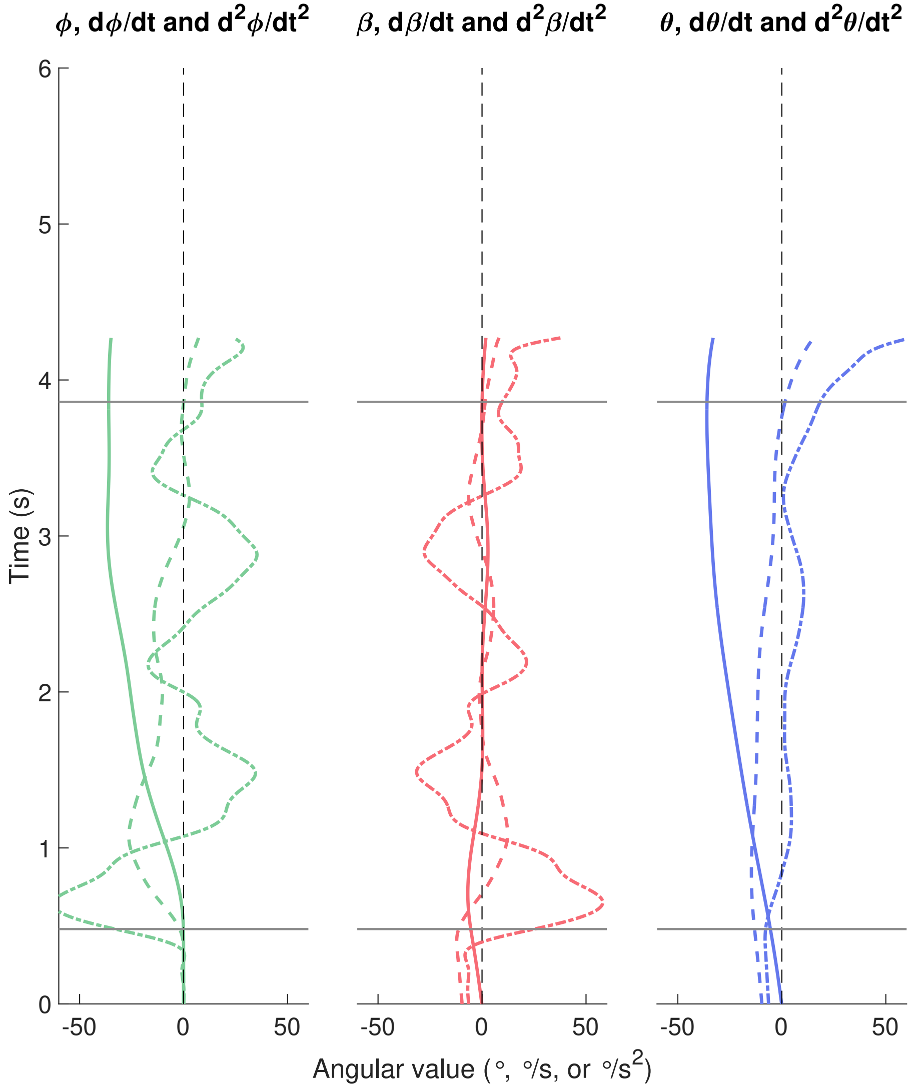

P6/B5
S10/R20-OUT

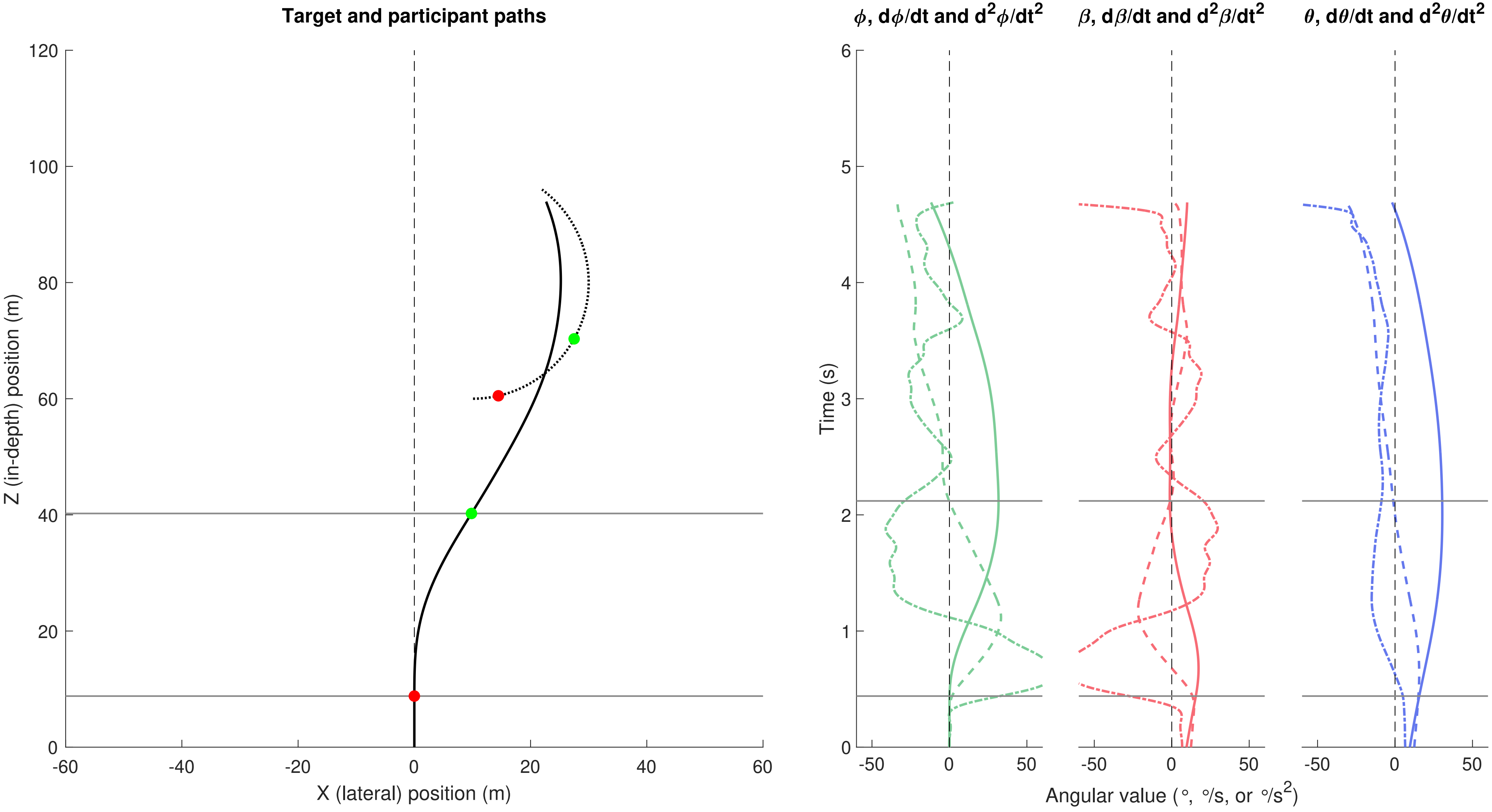

P6/B5
S10/R20-IN

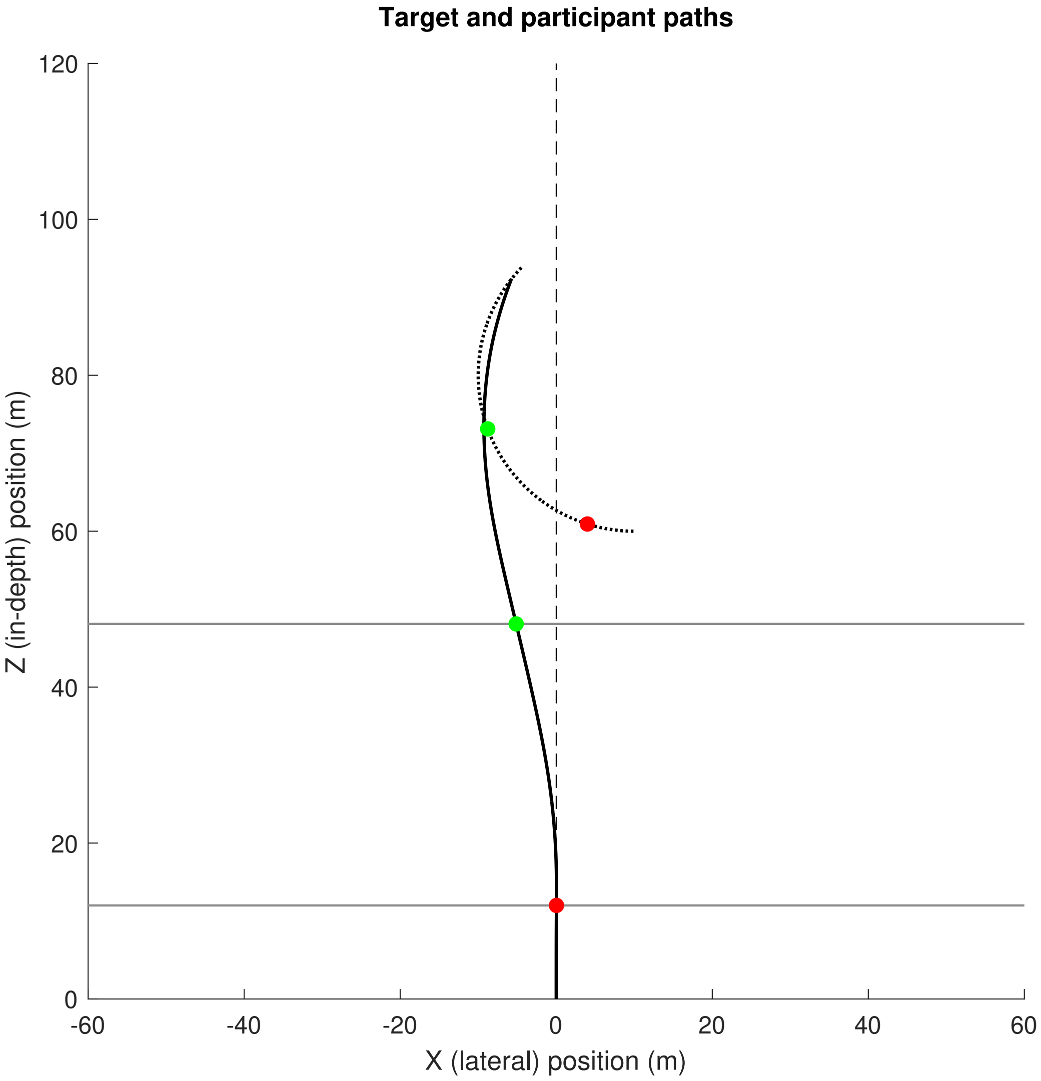

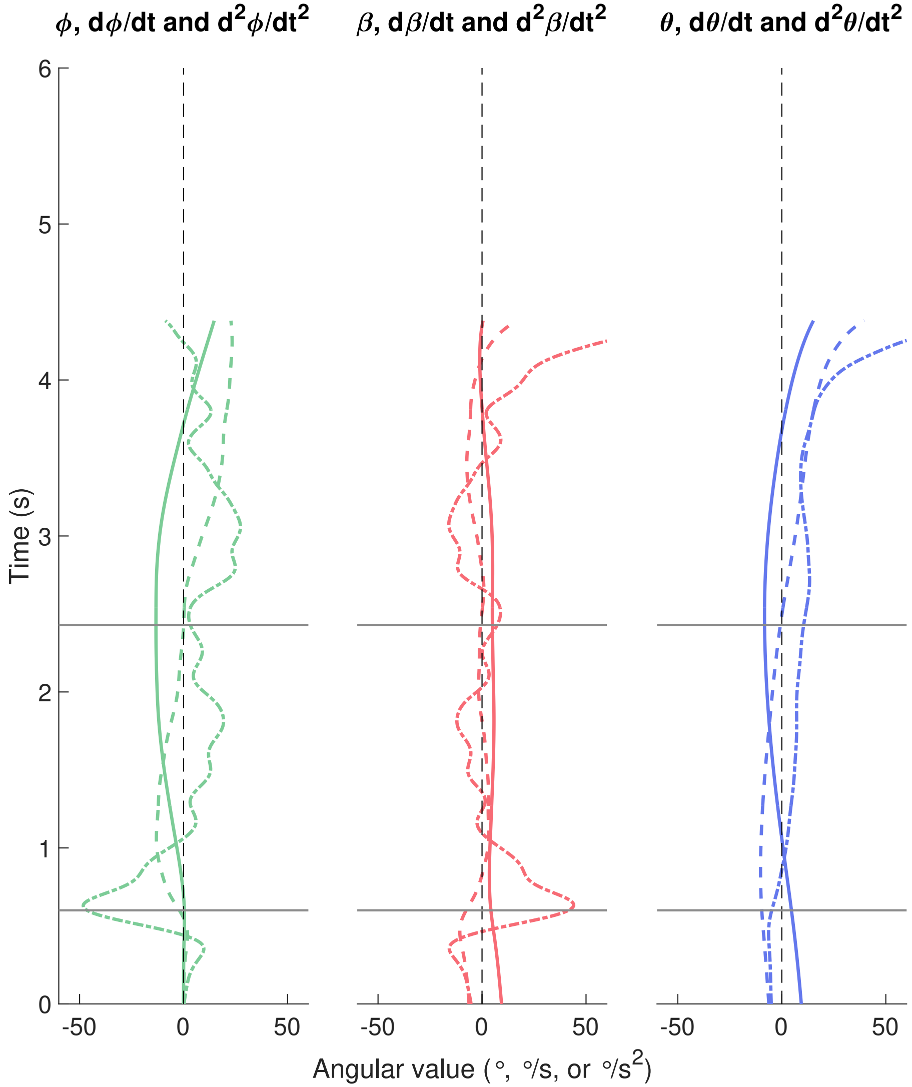

P6/B5
S10/R40-OUT

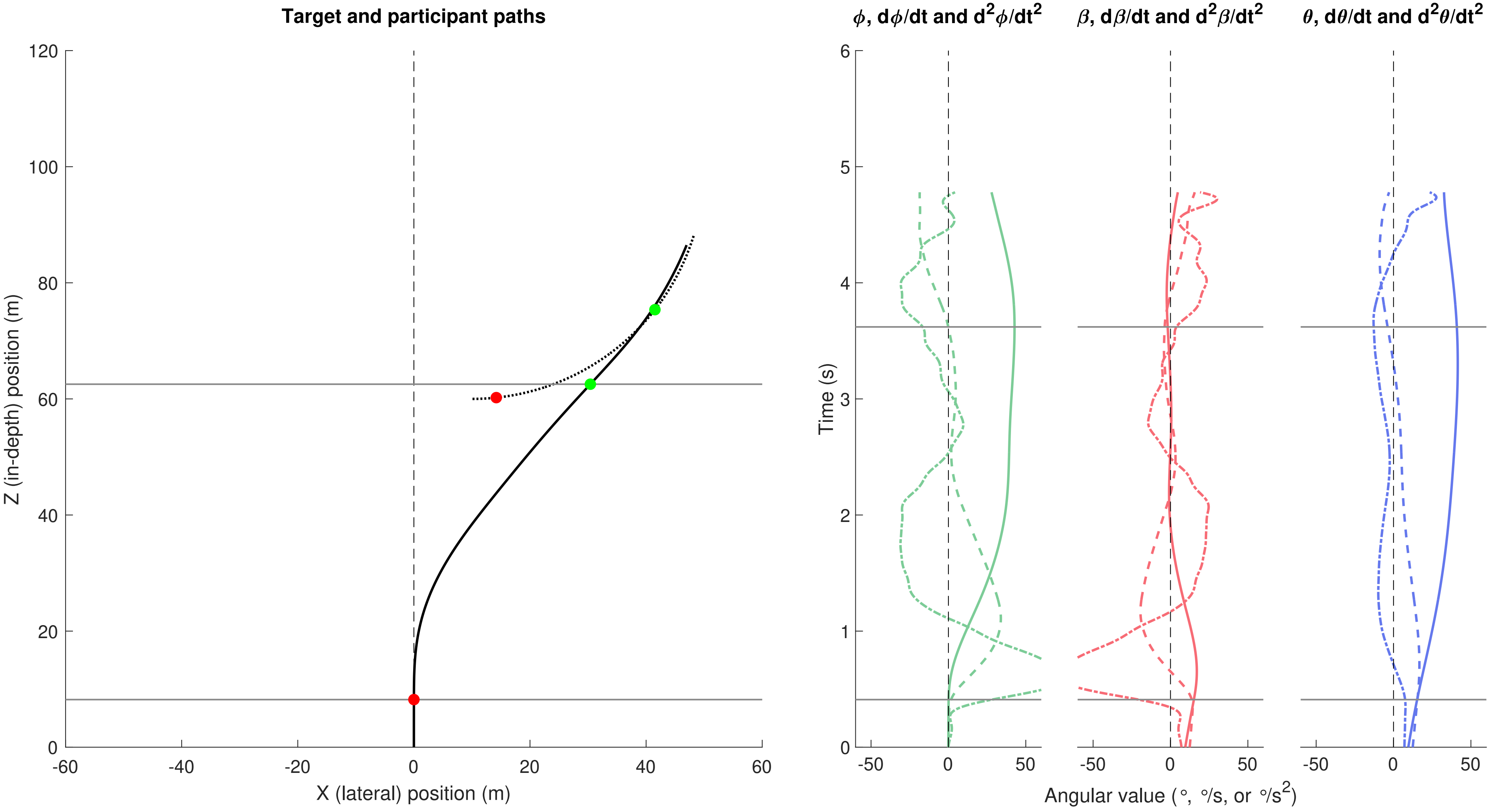

P6/B5
S10/R40-IN

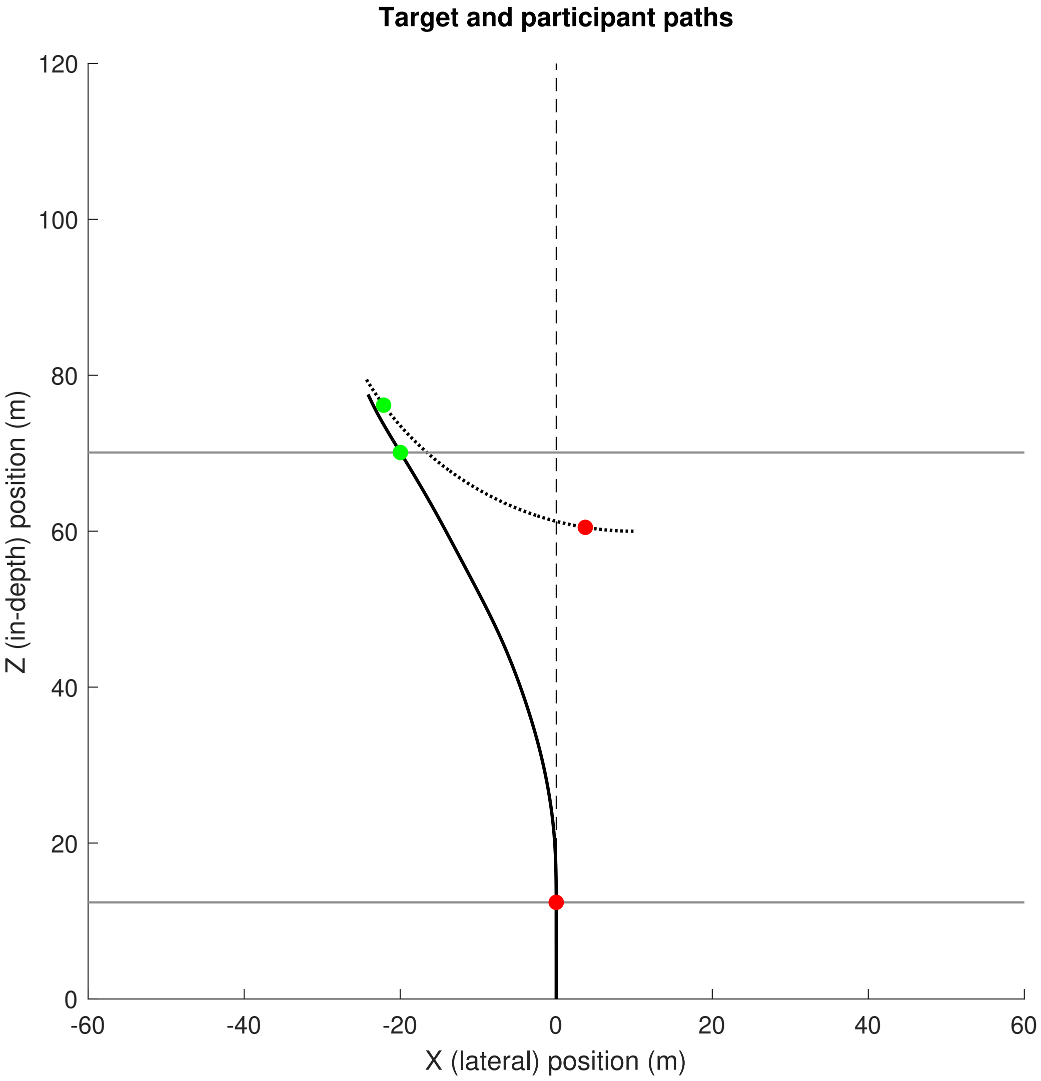

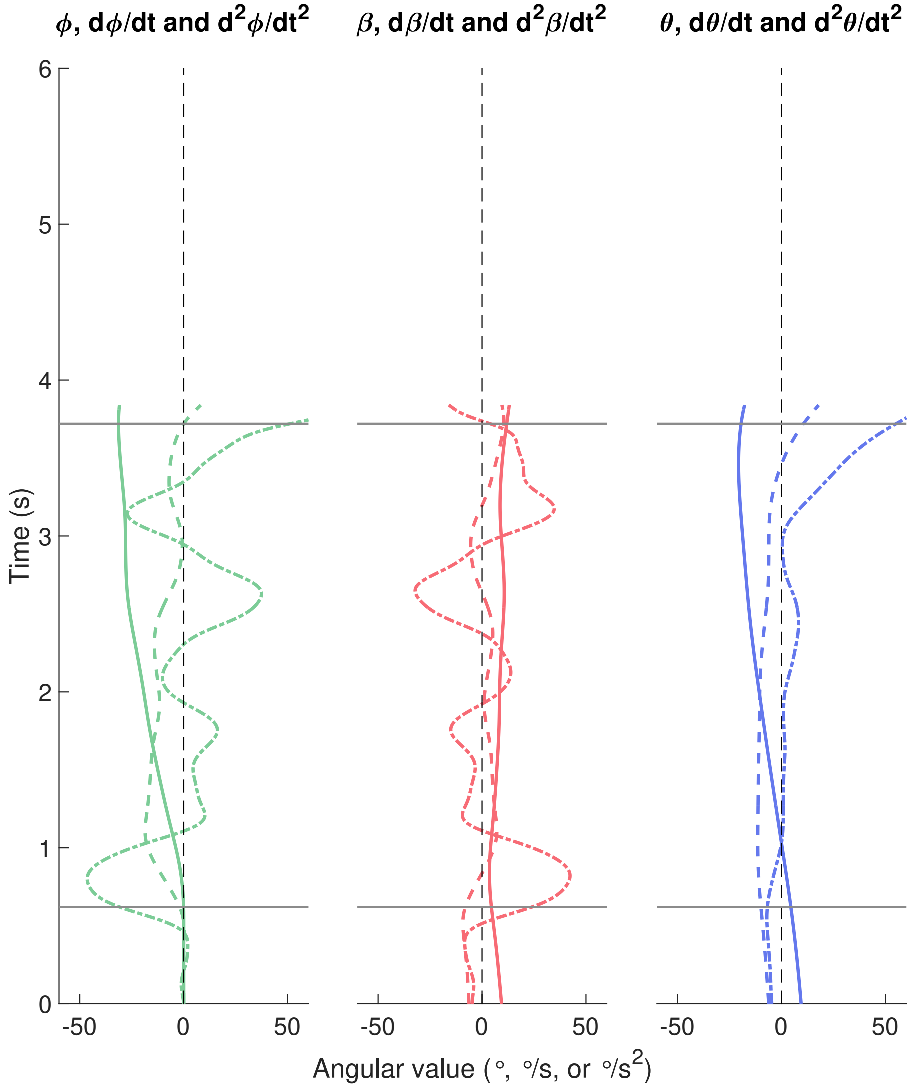

P6/B5
S20/R20-OUT

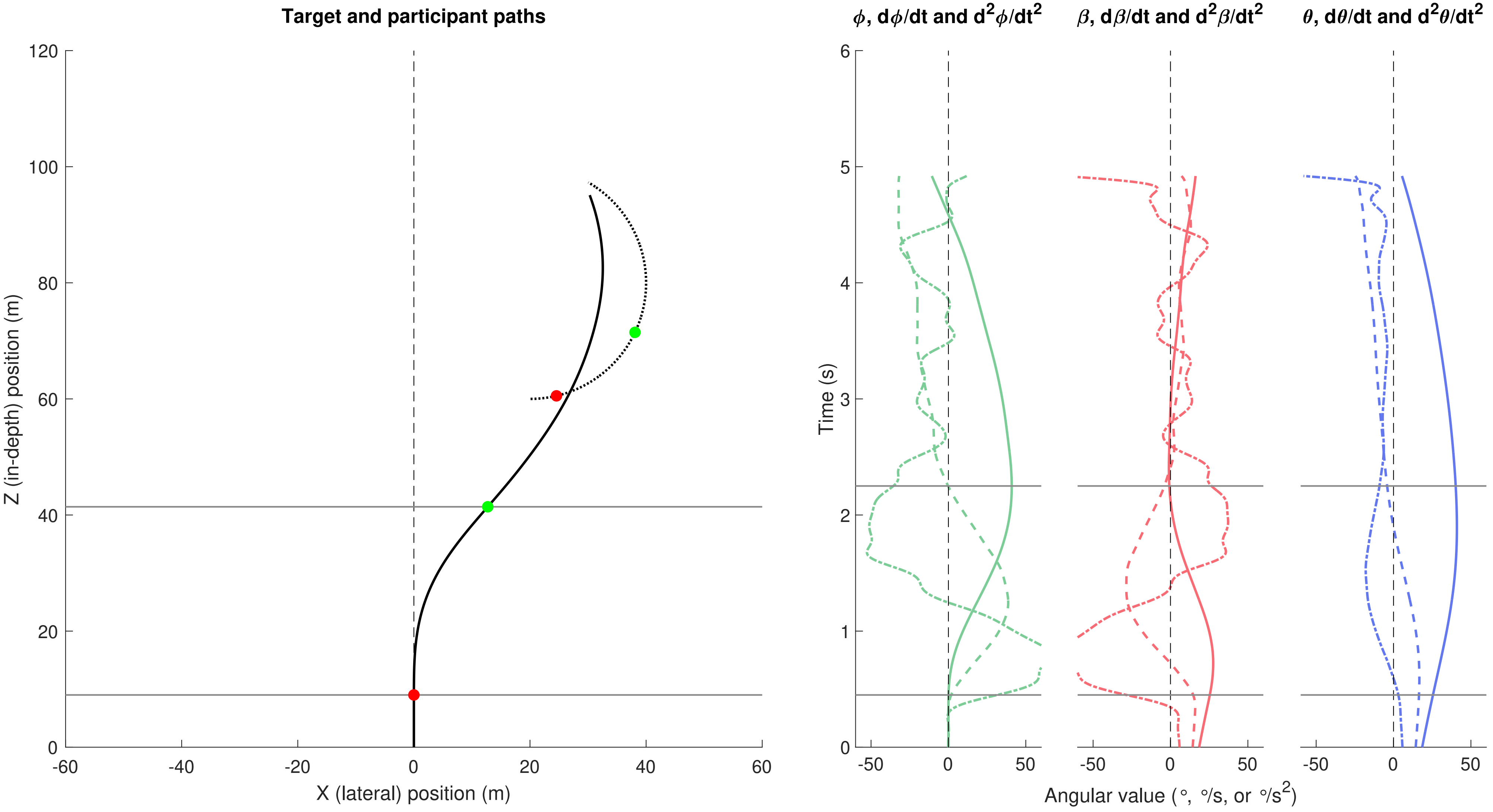

P6/B5
S20/R20-IN

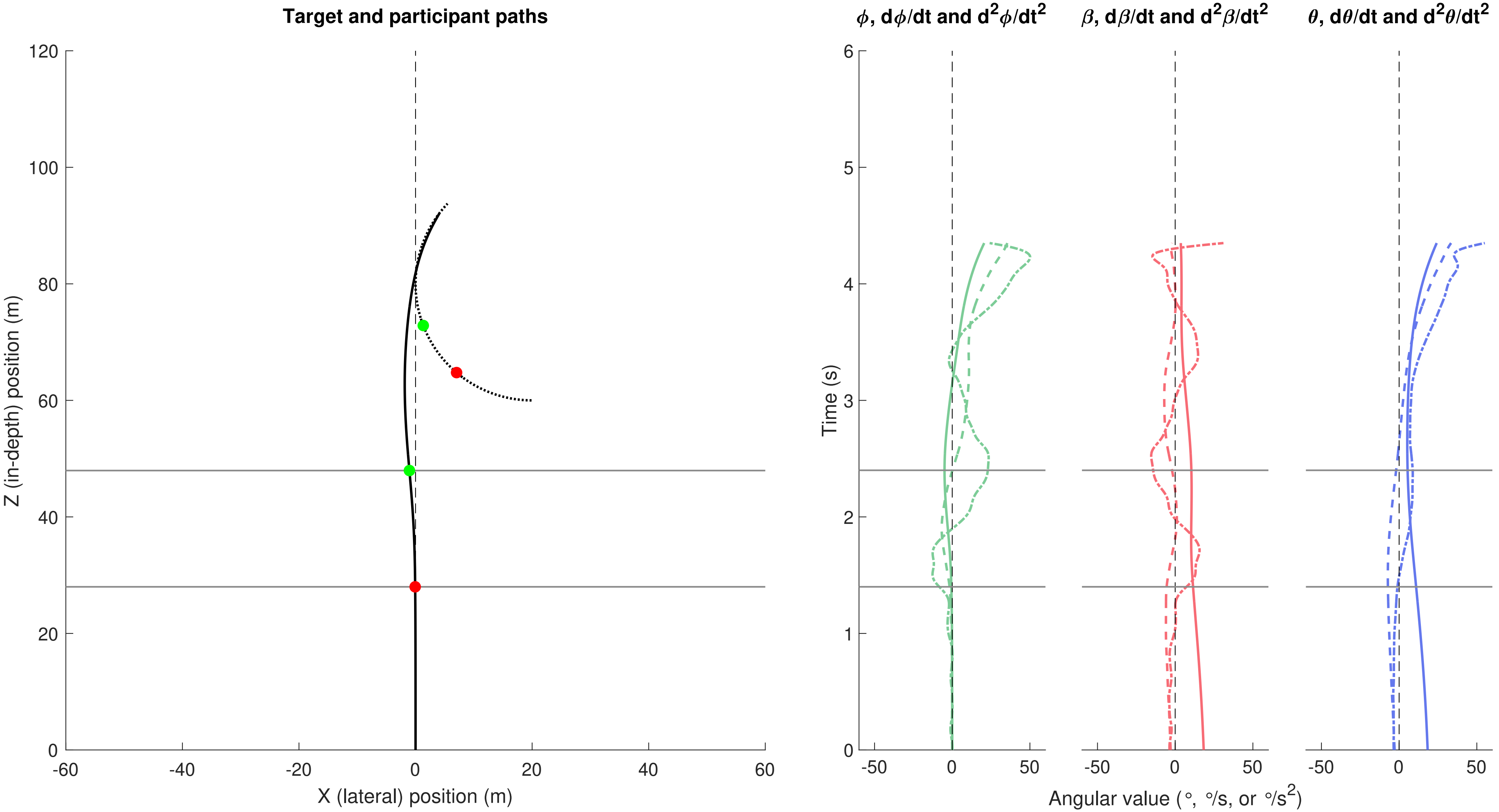

P6/B5
S20/R40-OUT

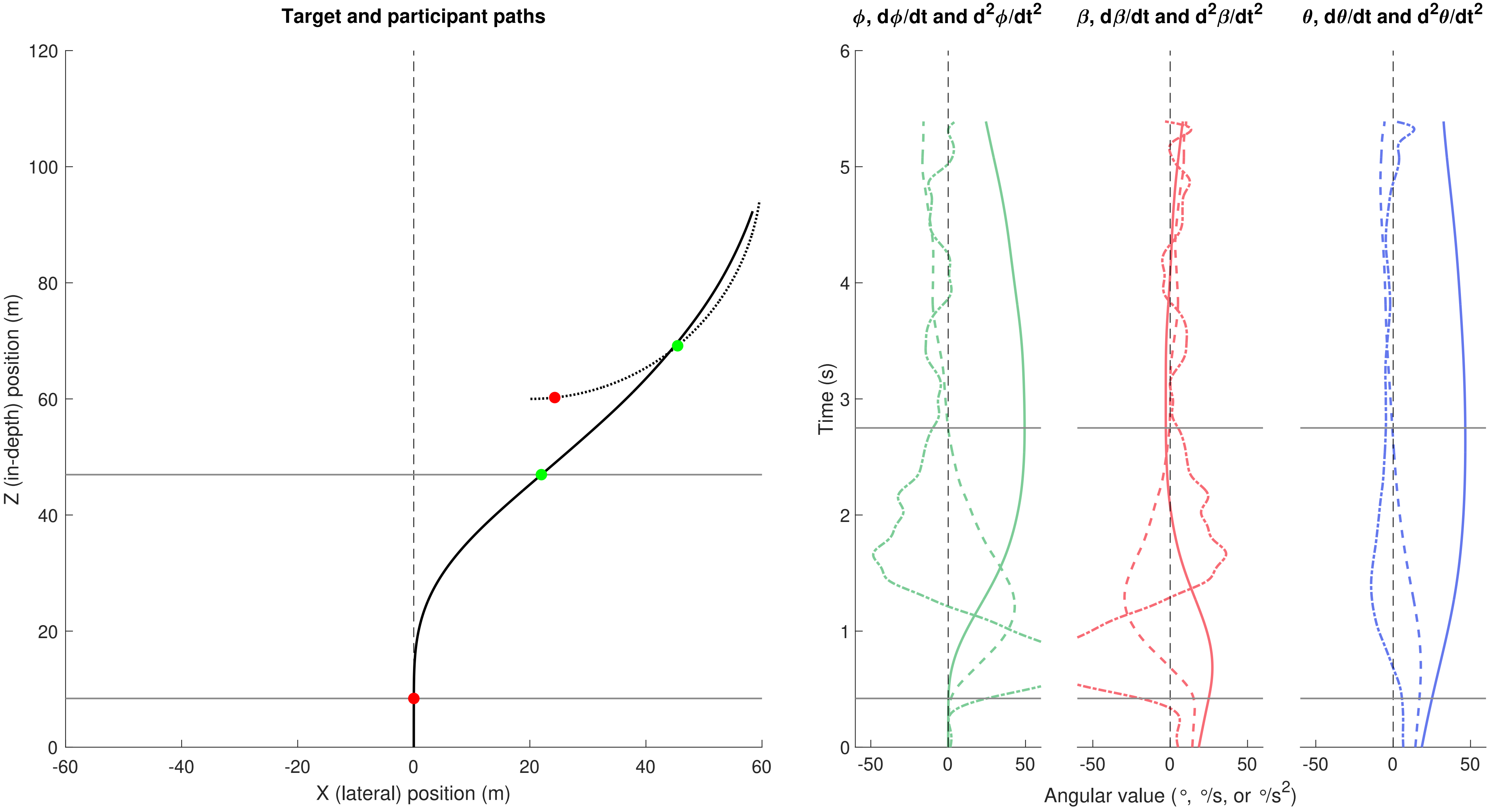

P6/B5
S20/R40-IN

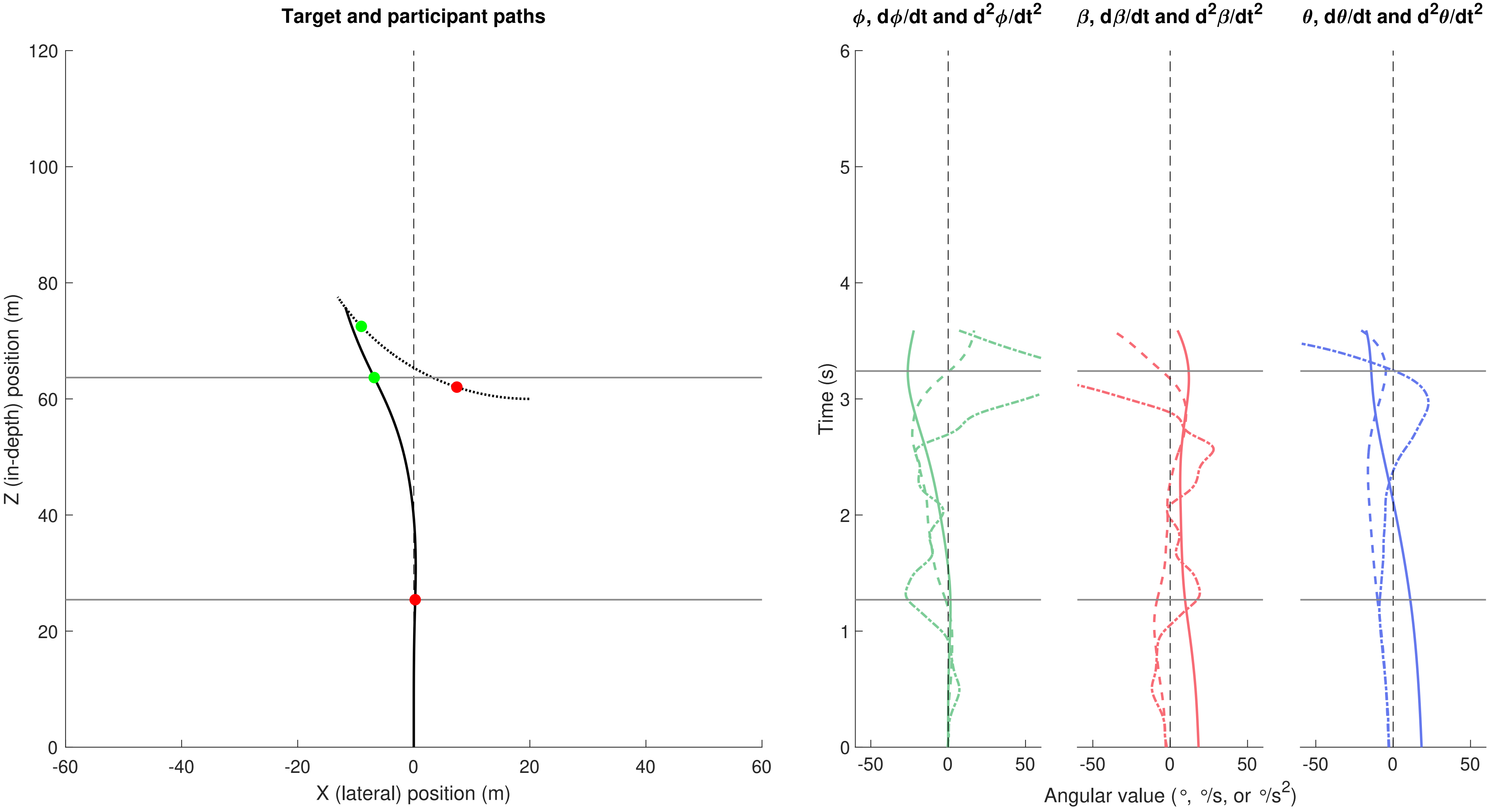

P6/B6
S20/R20-IN

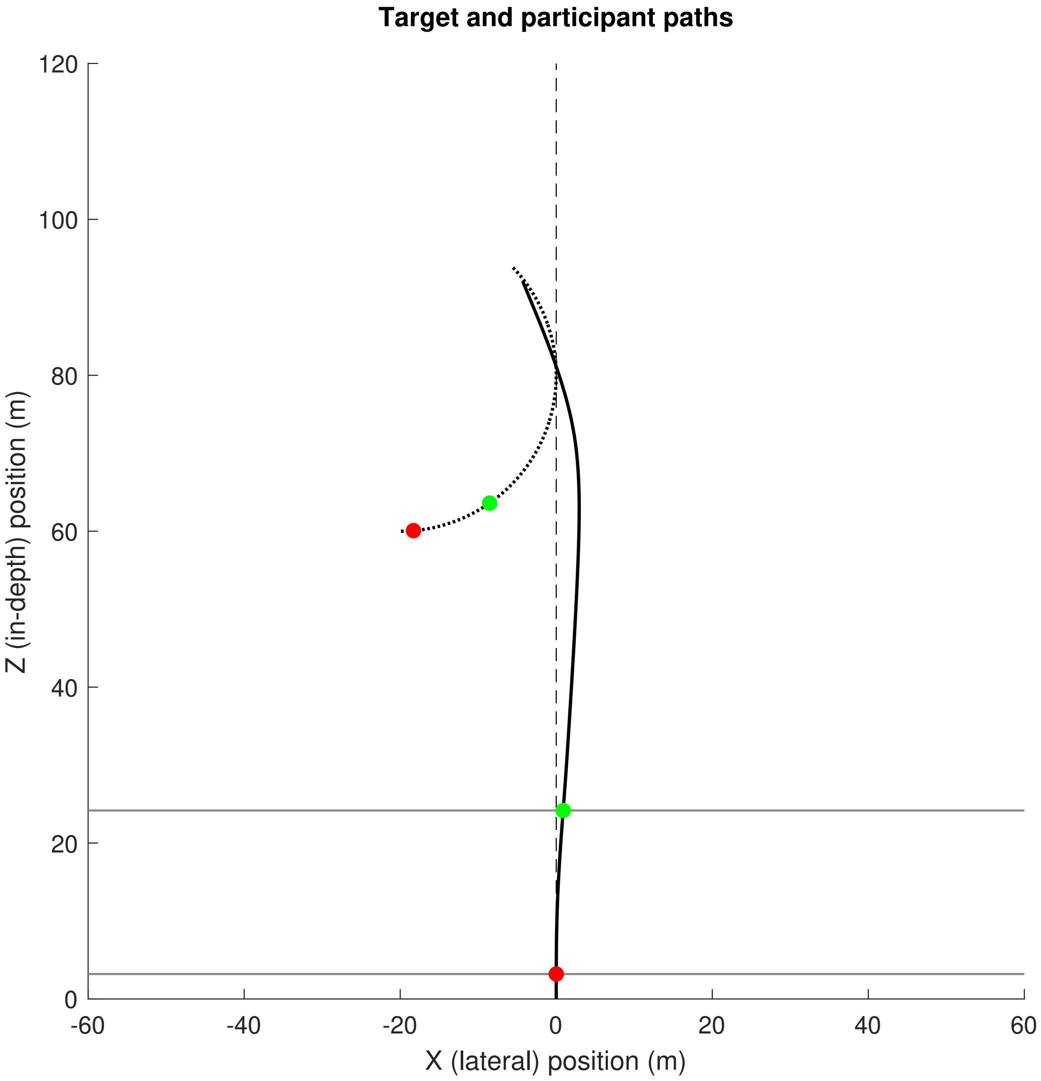

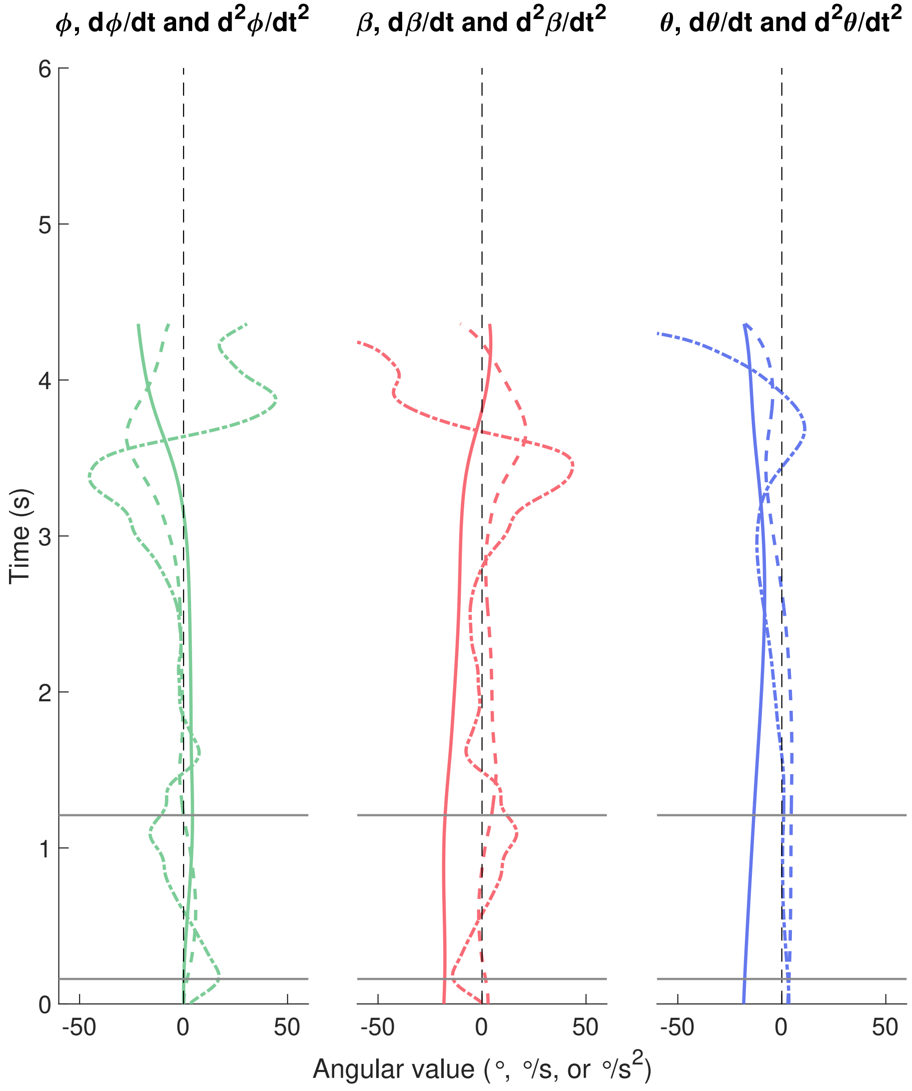

P6/B6
S20/R20-OUT

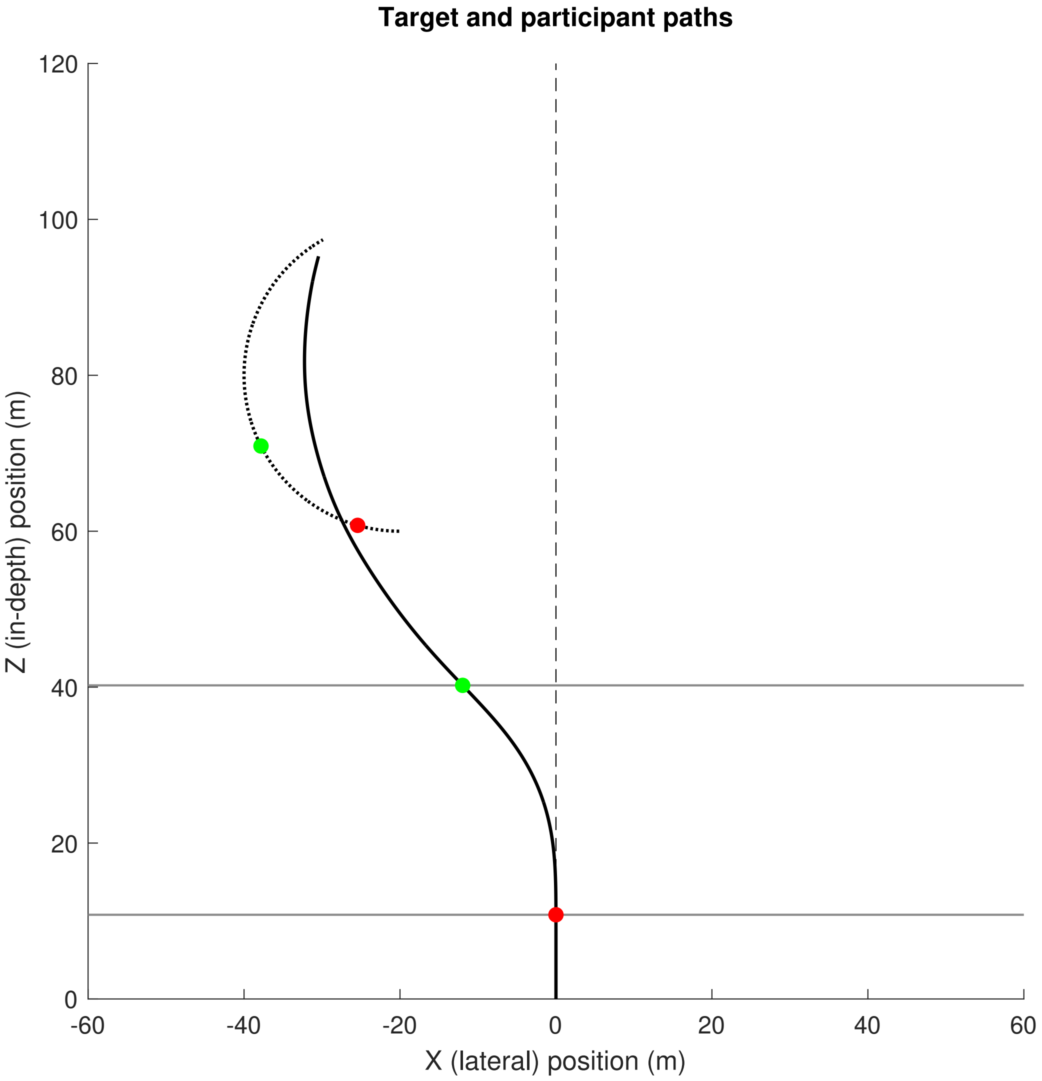

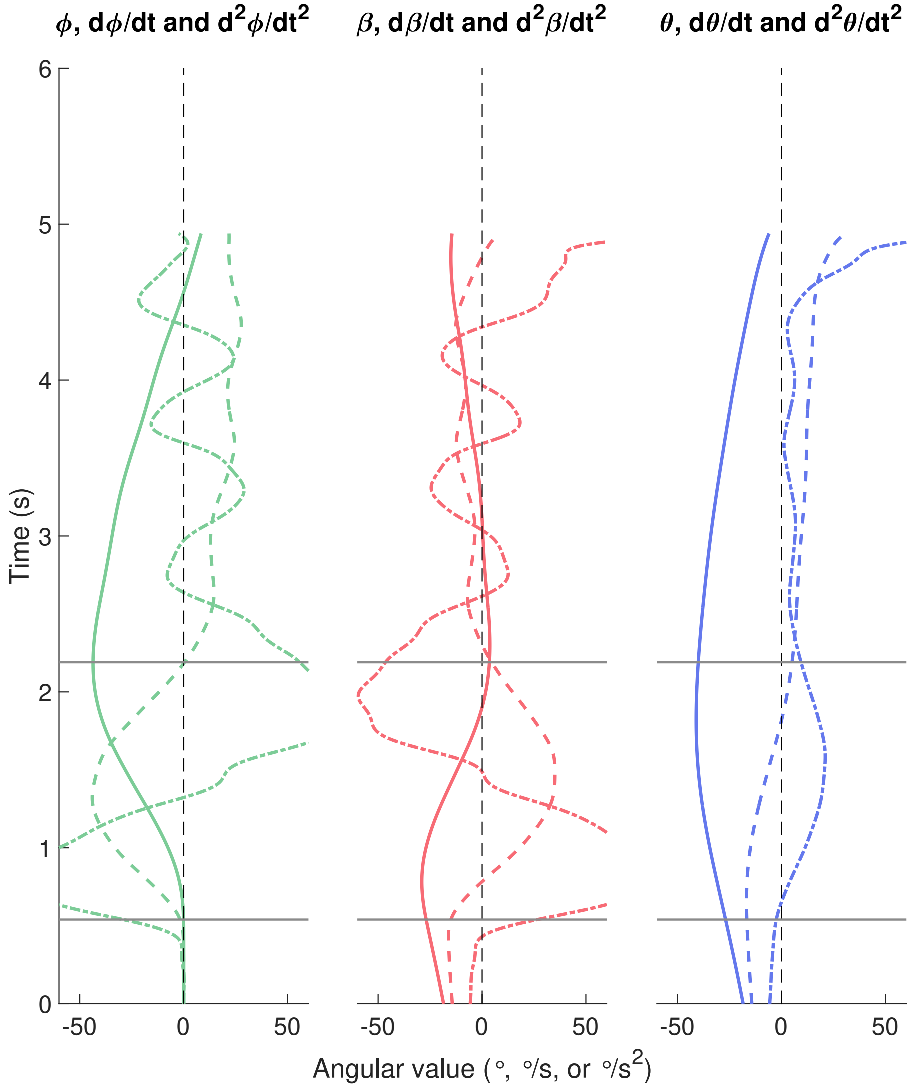

P6/B6
S20/R40-IN

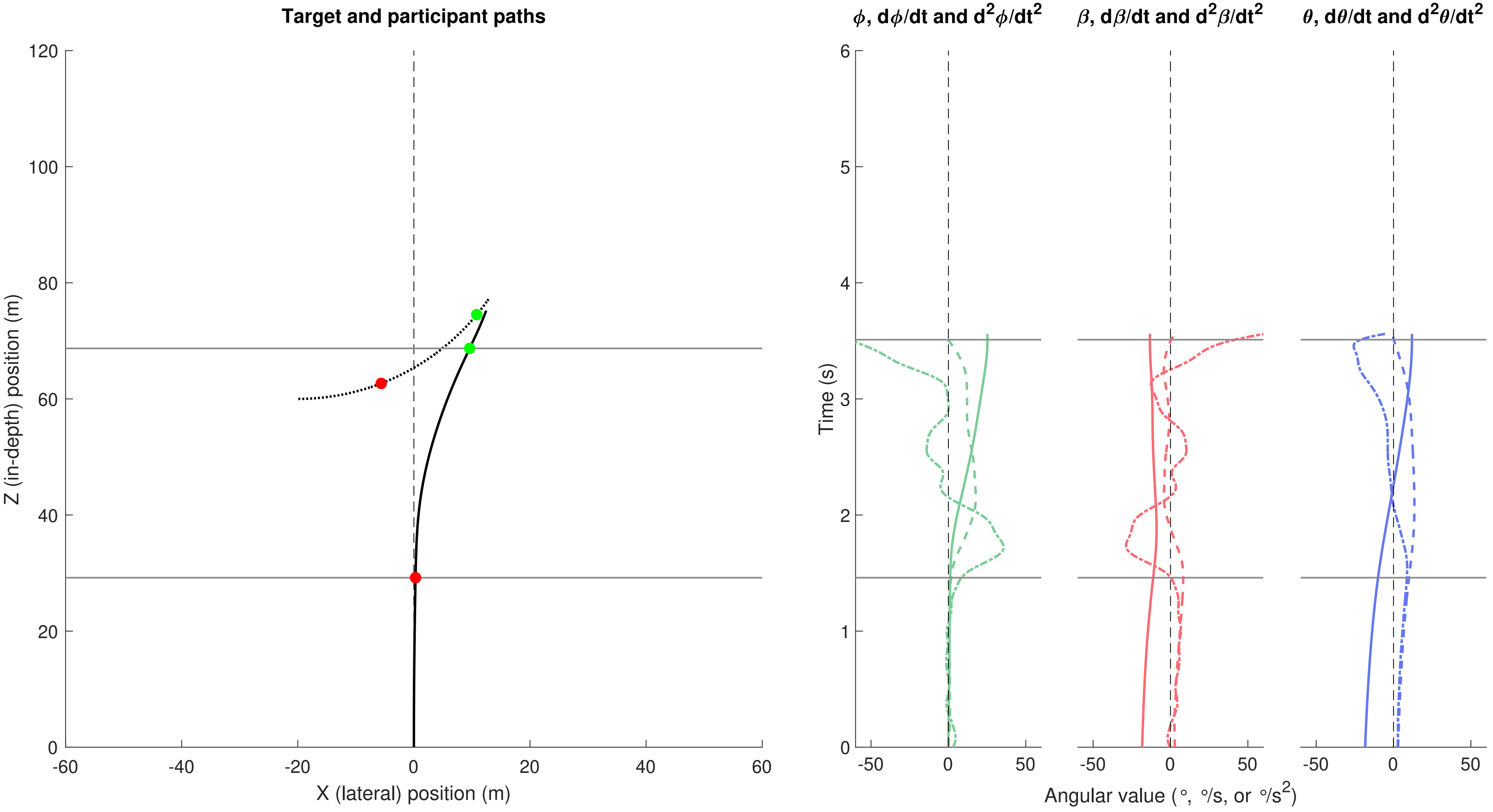

P6/B6
S20/R40-OUT

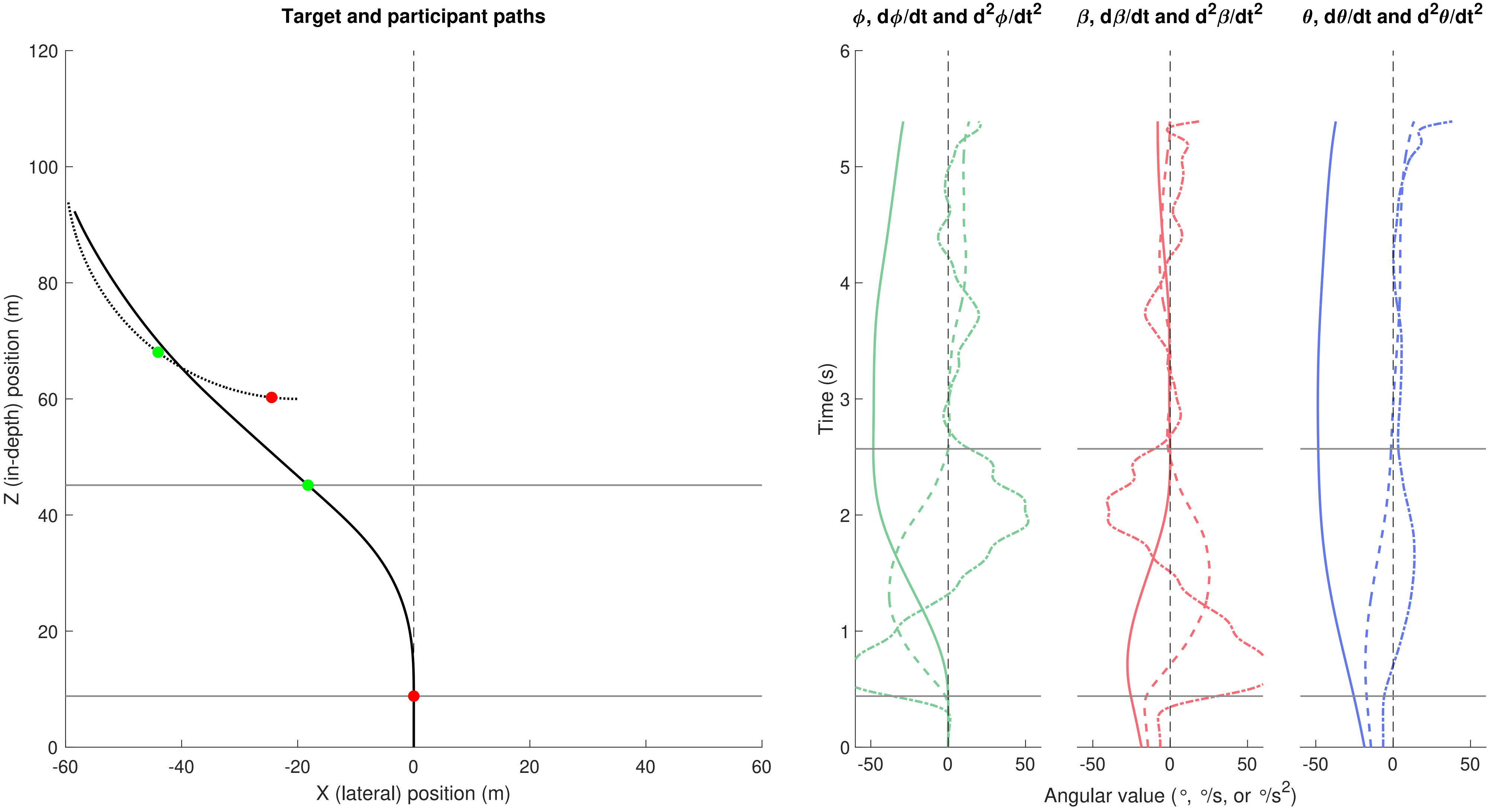

P6/B6
S10/R20-IN

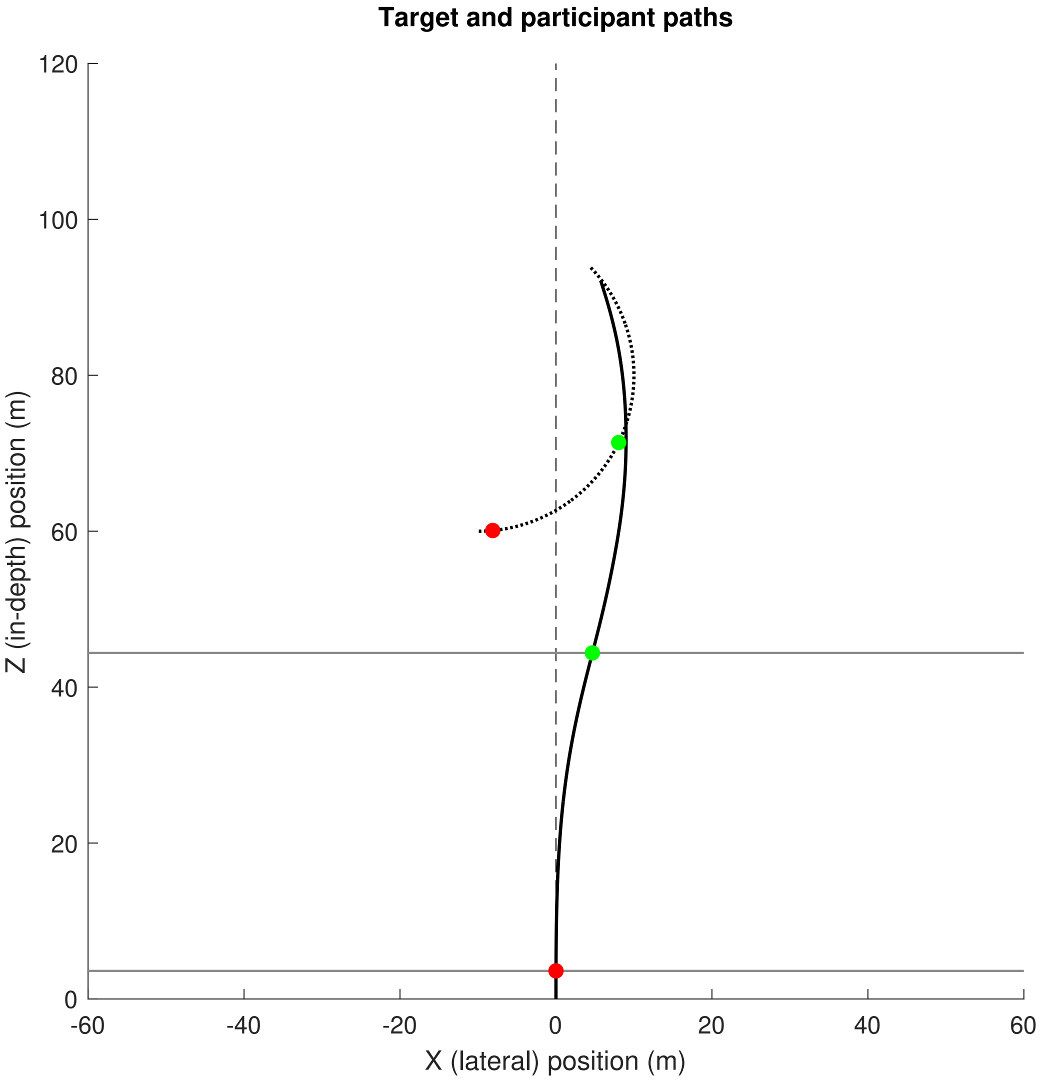

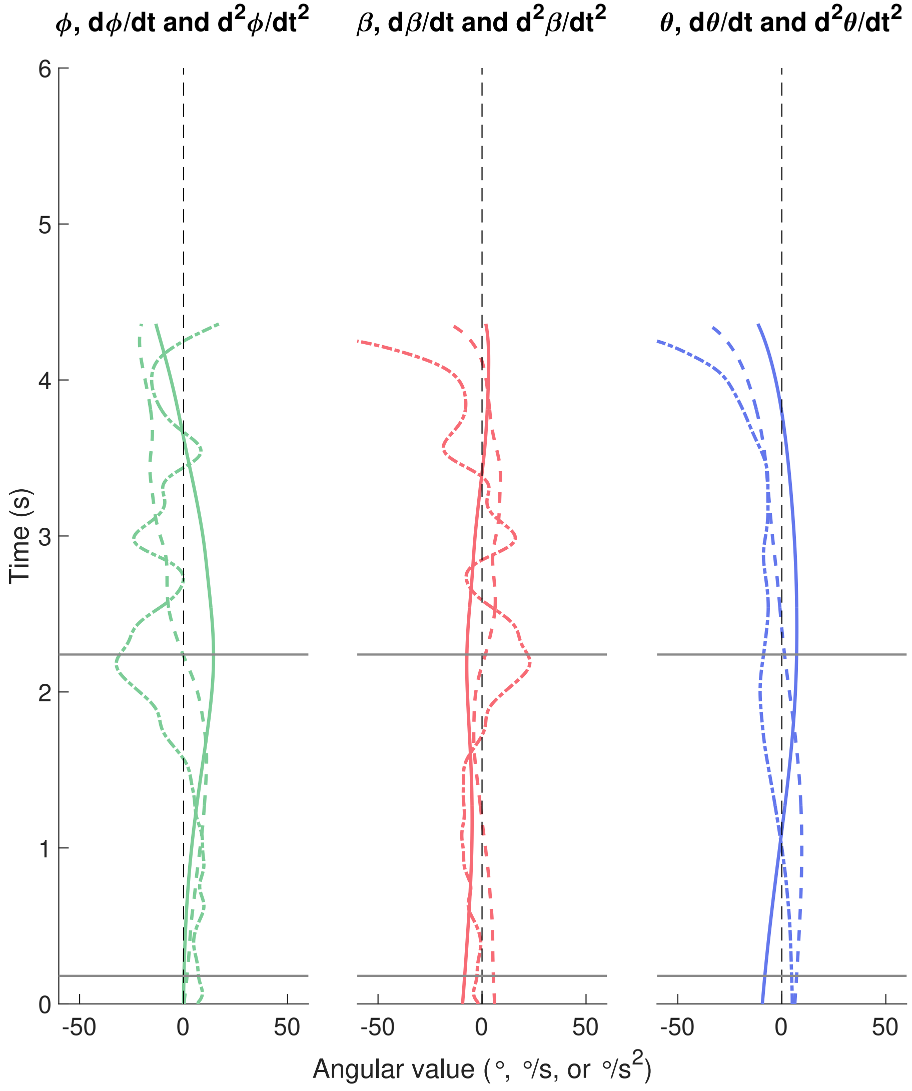

P6/B6
S10/R20-OUT

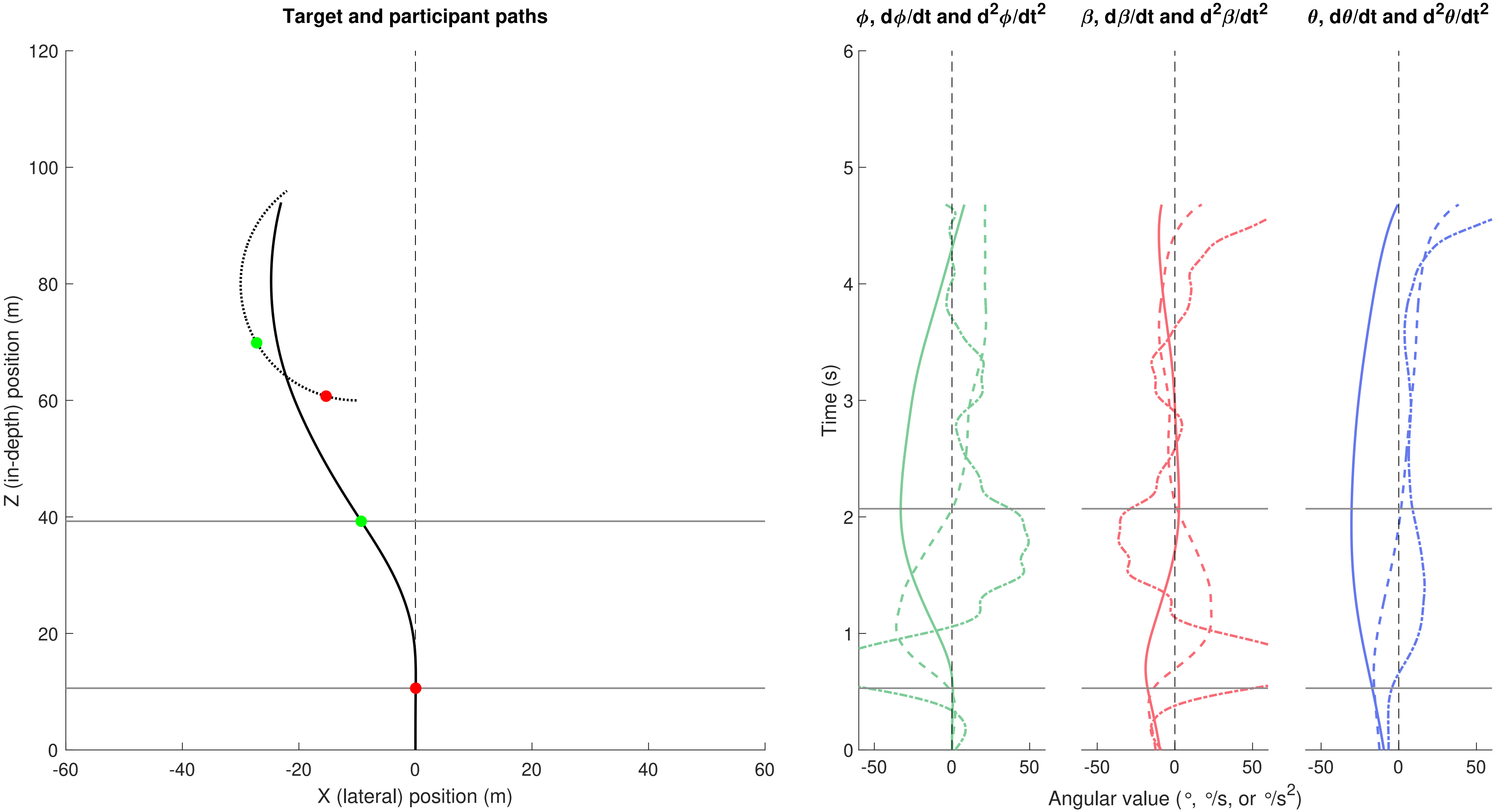

P6/B6
S10/R40-IN

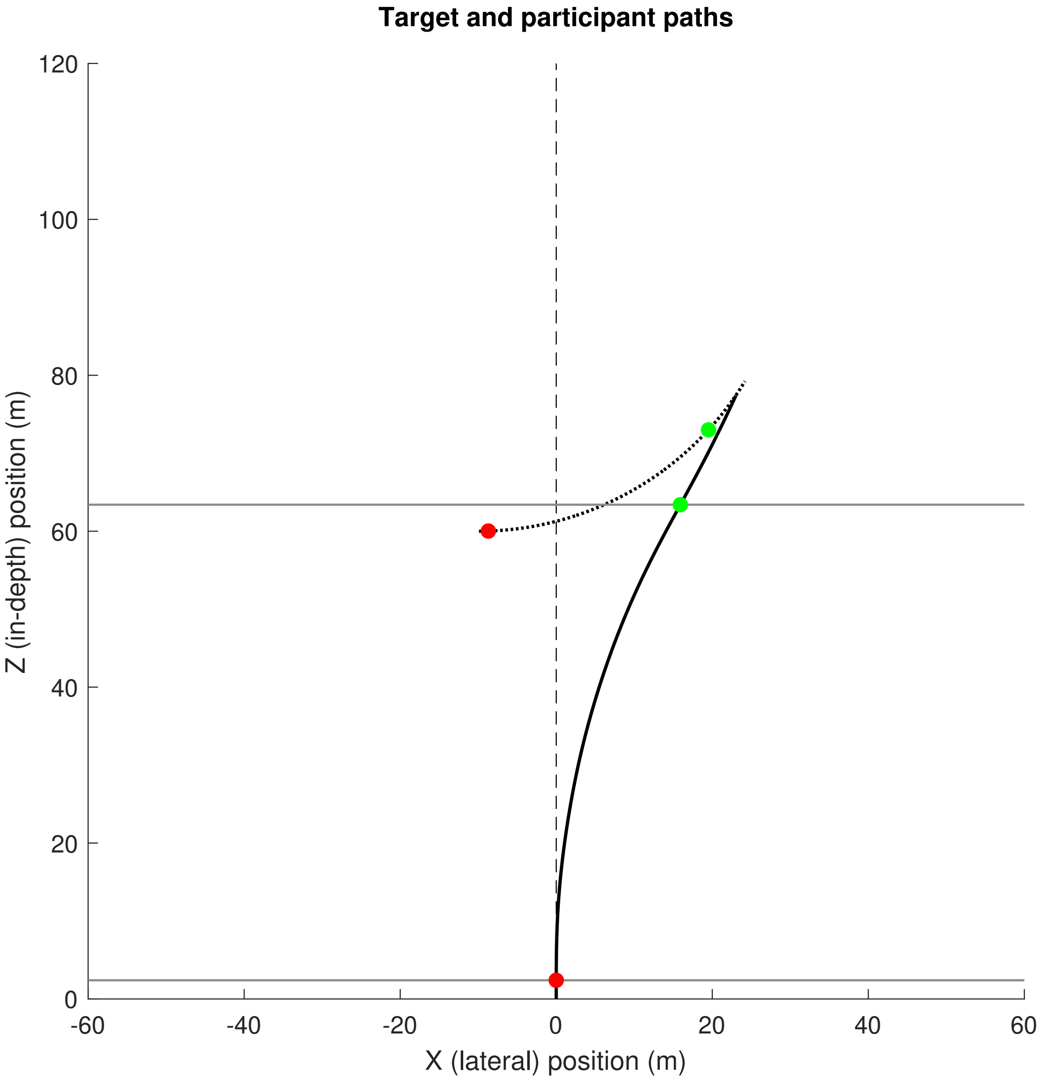

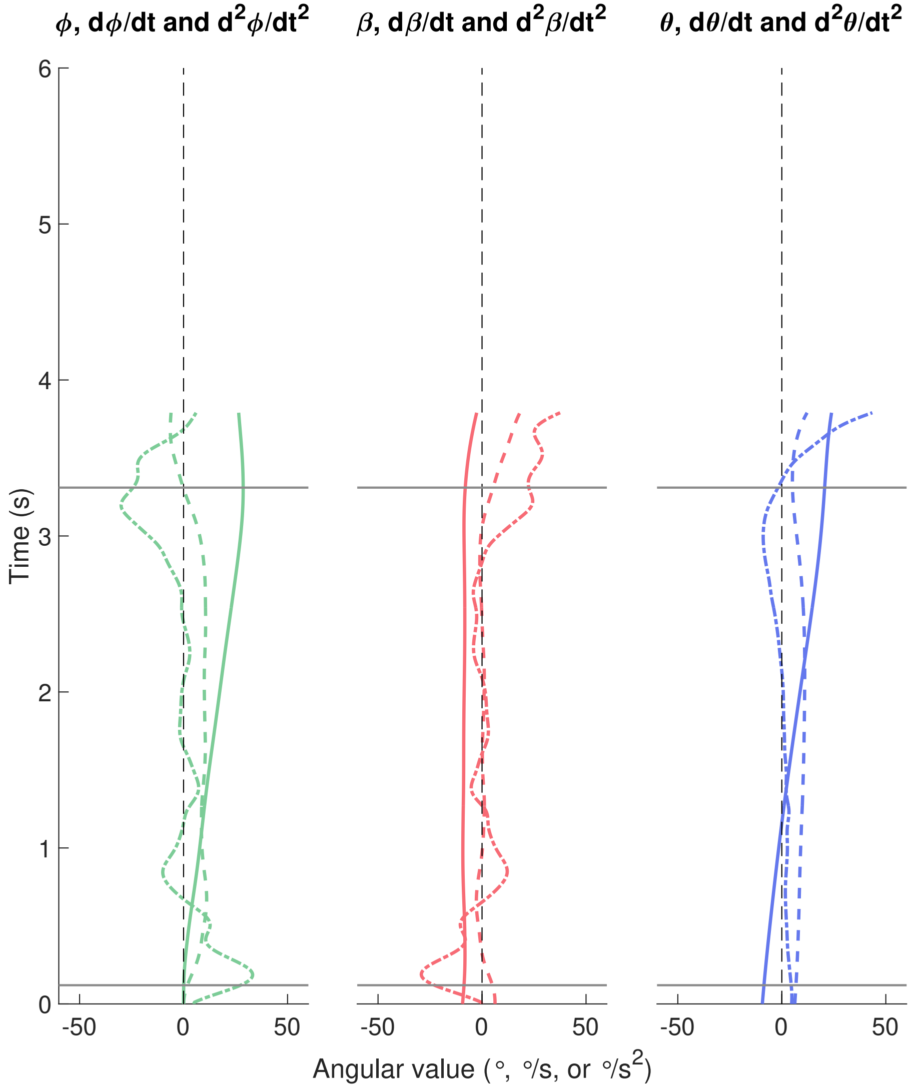

P6/B6
S10/R40-OUT

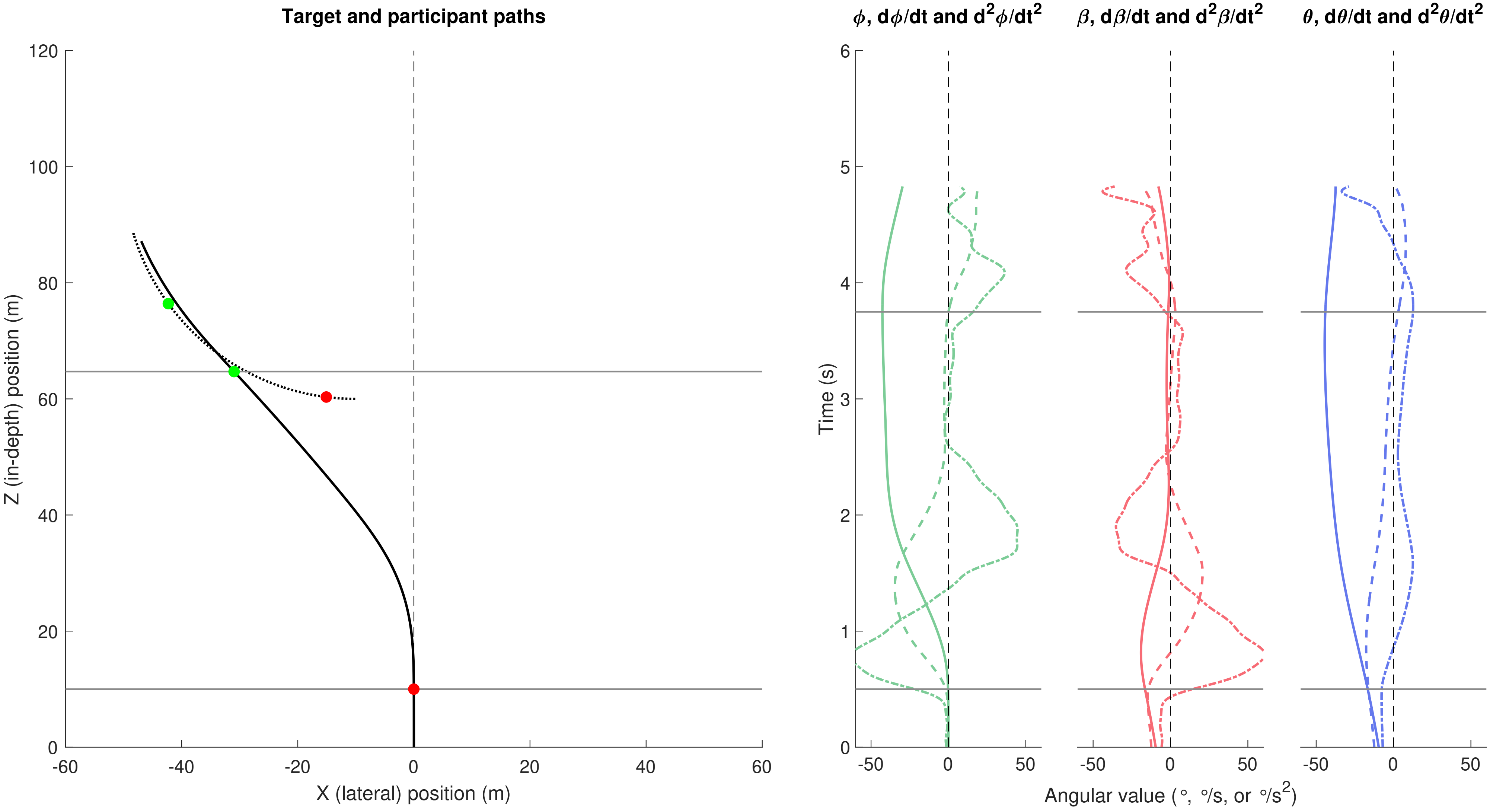

P6/B6
S0/R20-OUT

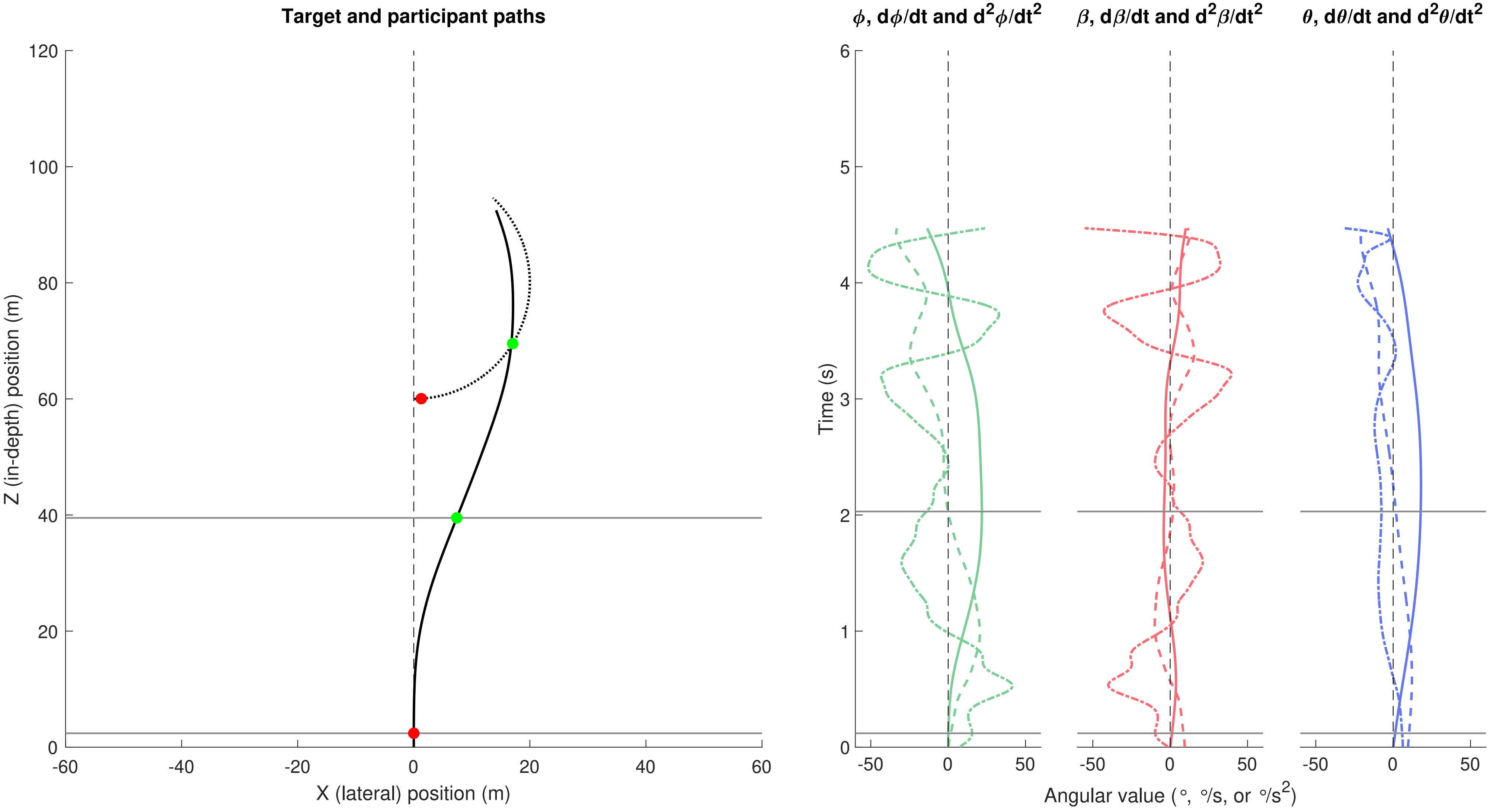

P6/B6
S0/R20-OUT

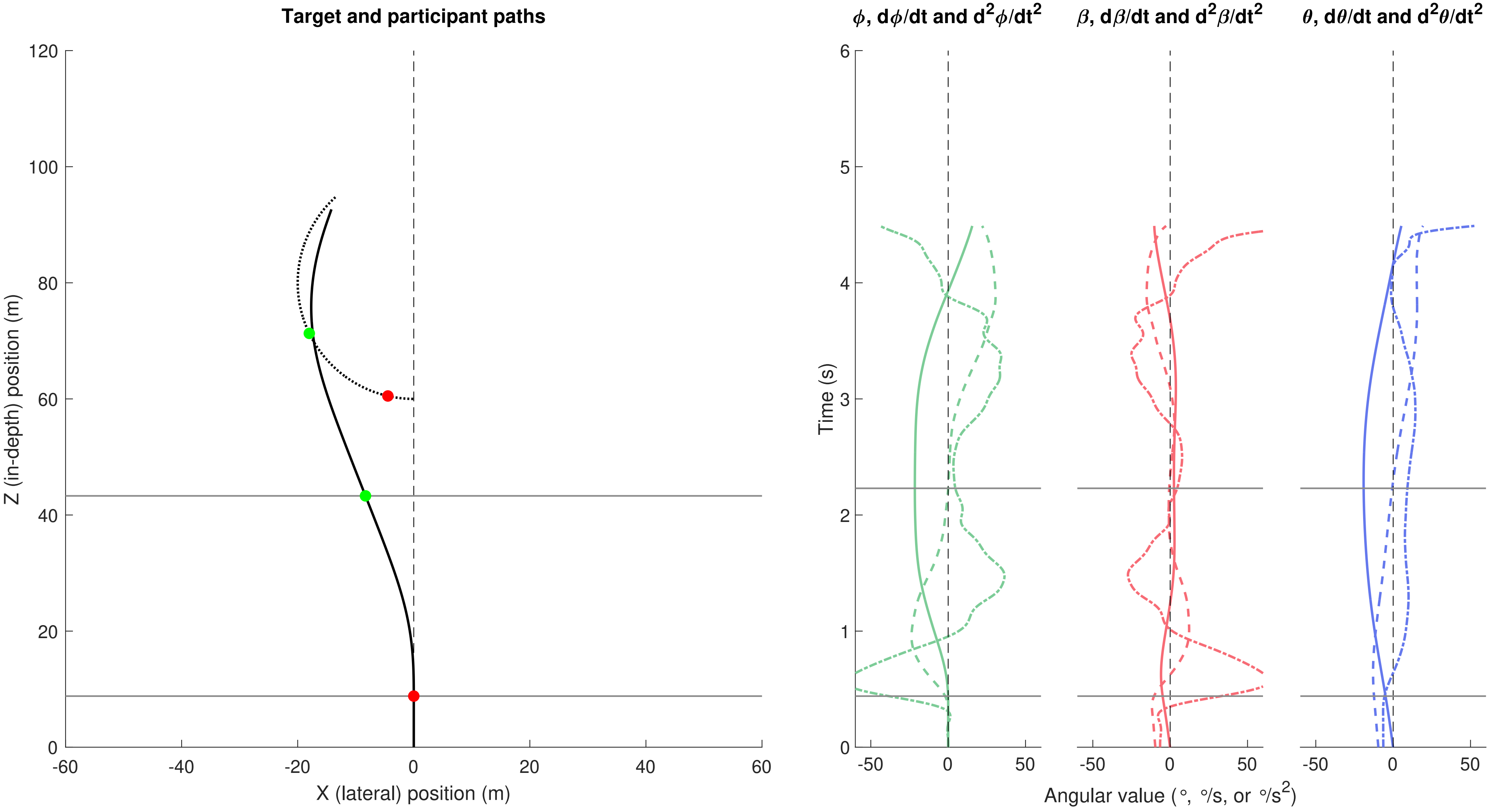

P6/B6
S0/R40-OUT

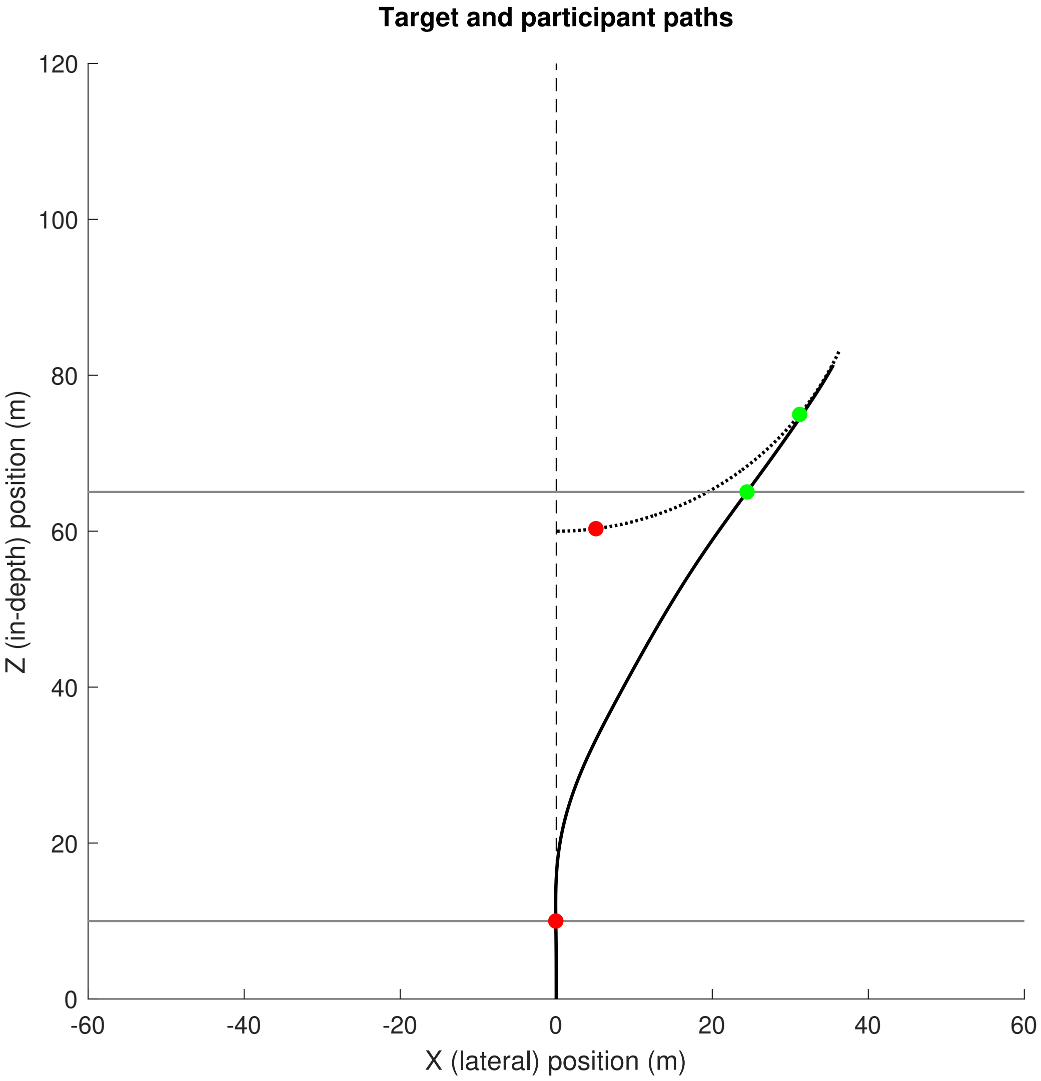

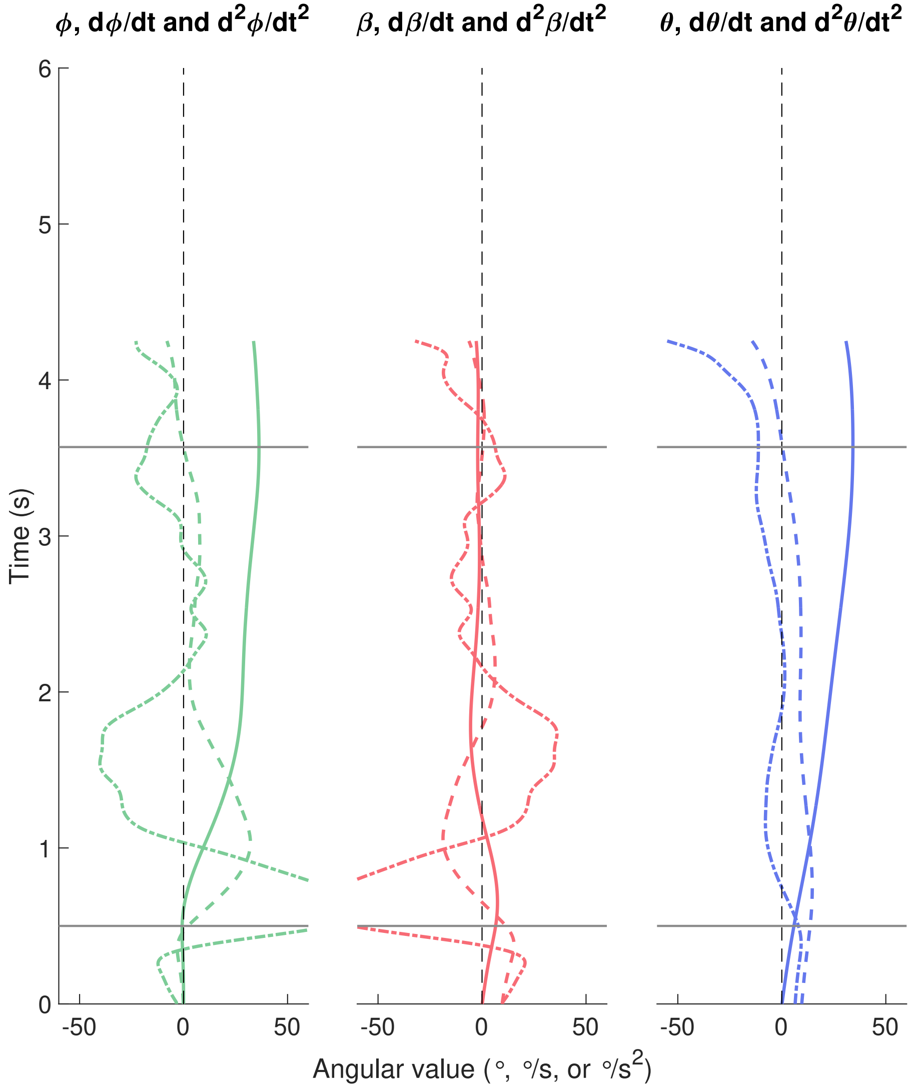

P6/B6
S0/R40-OUT

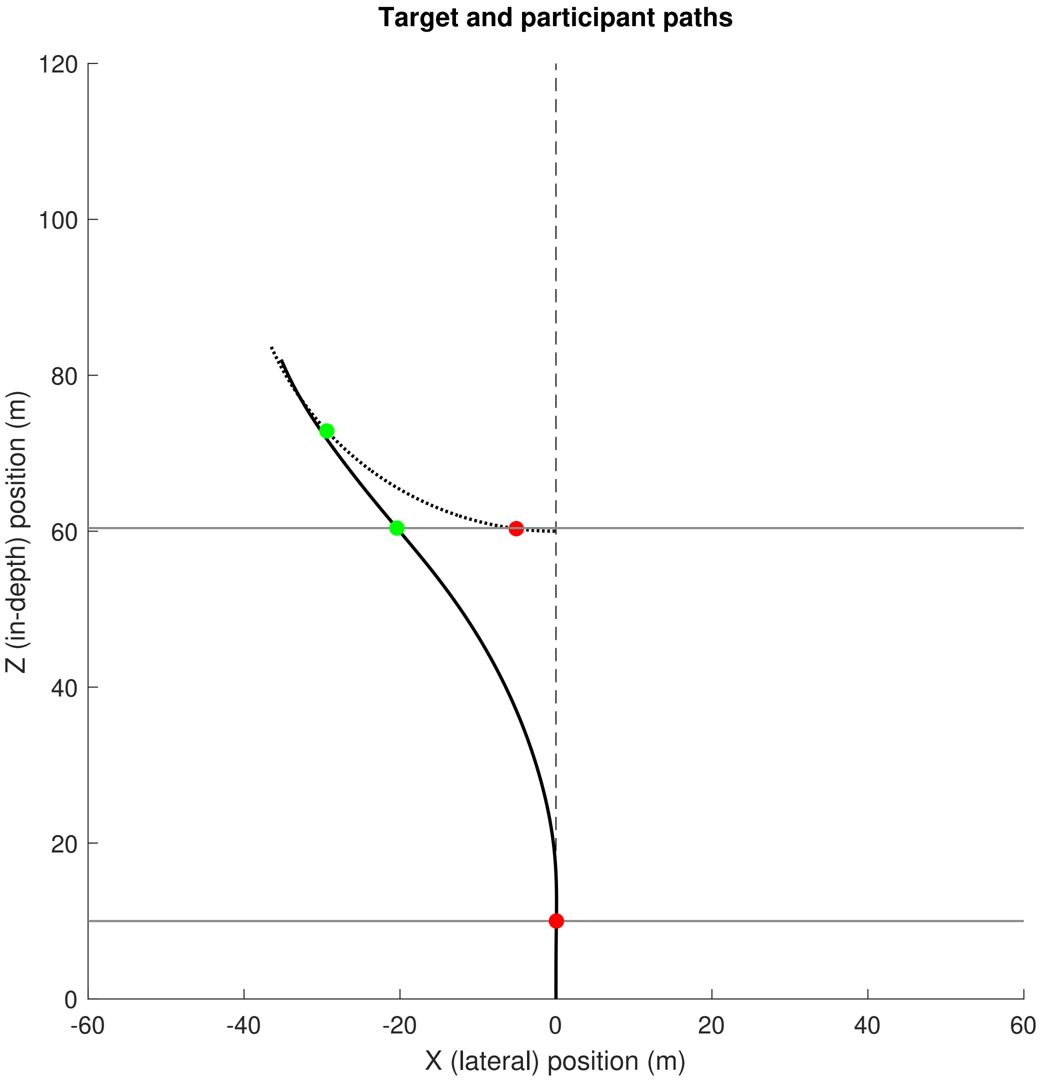

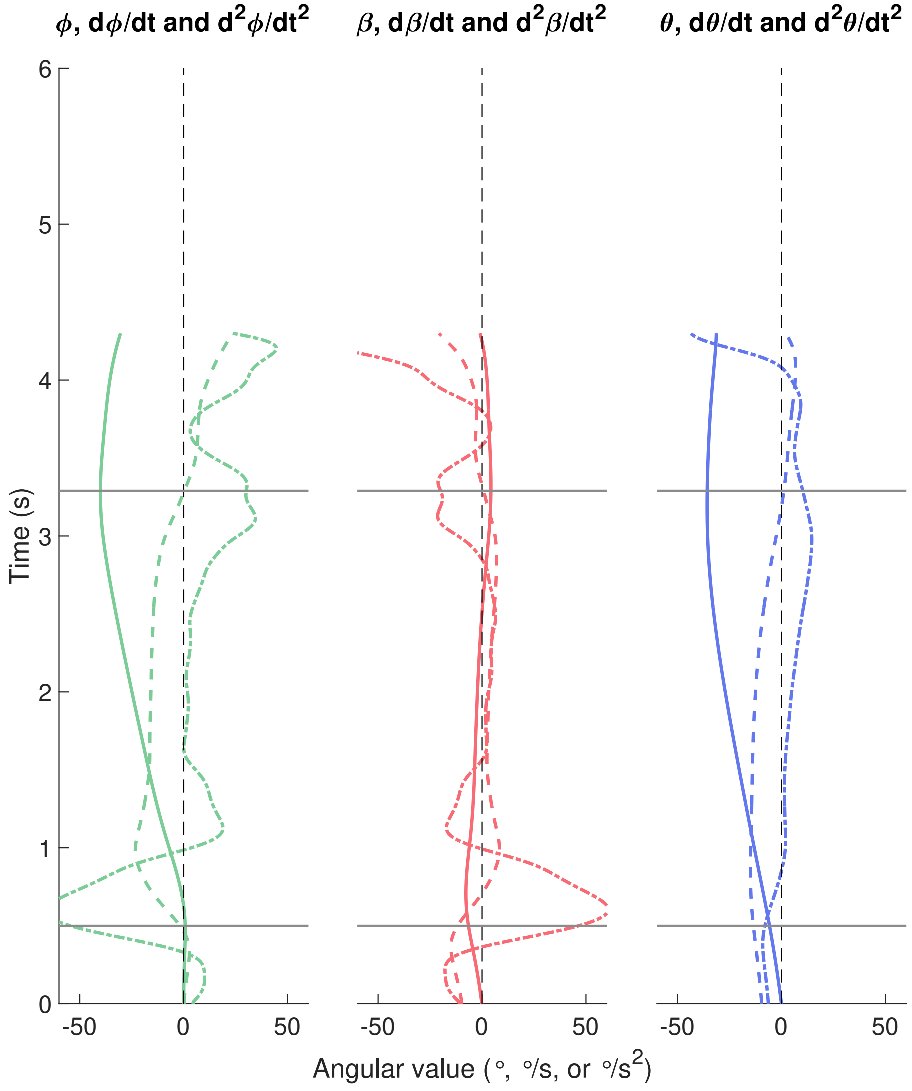

P6/B6
S10/R20-OUT

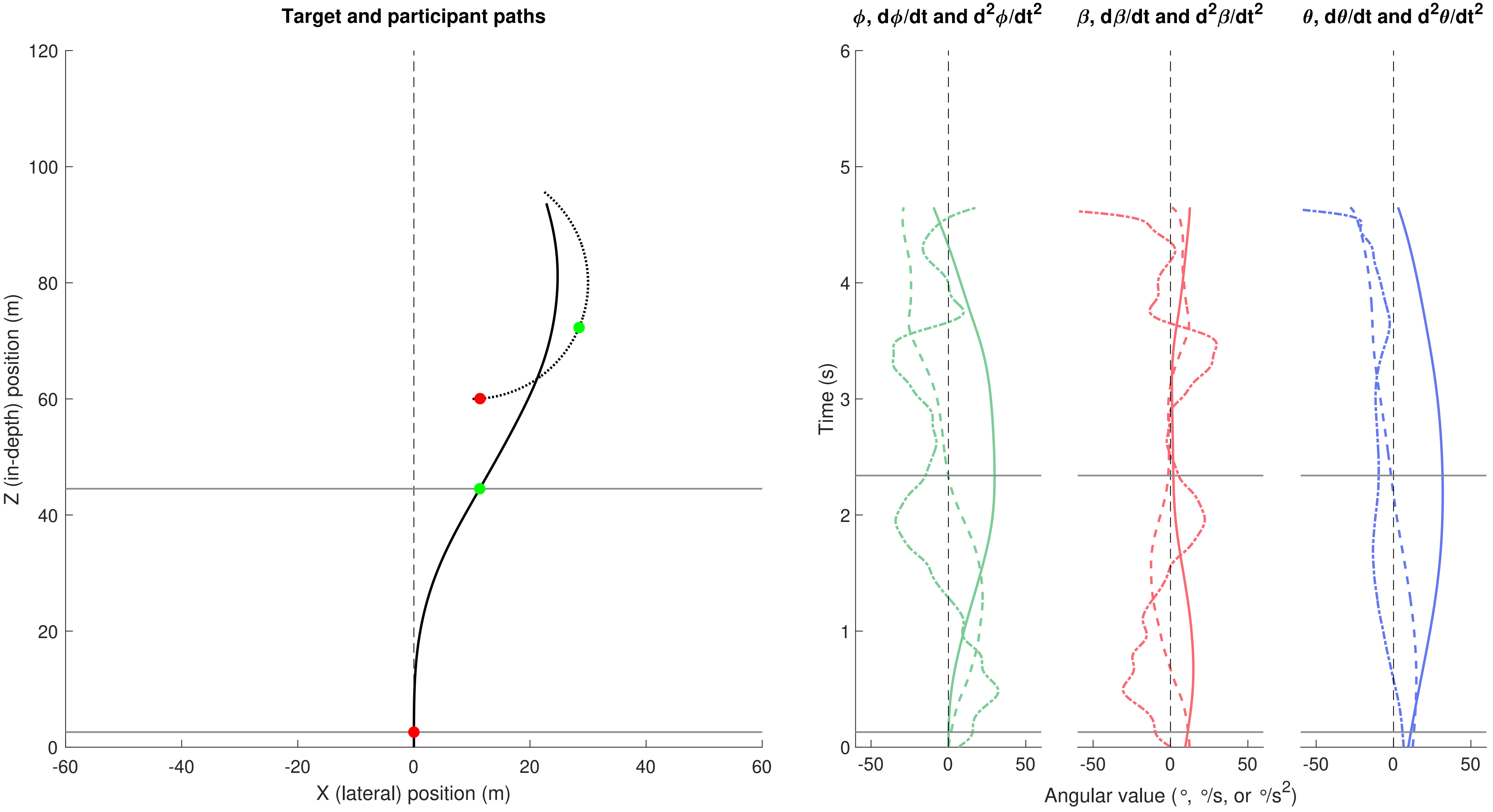

P6/B6
S10/R20-IN

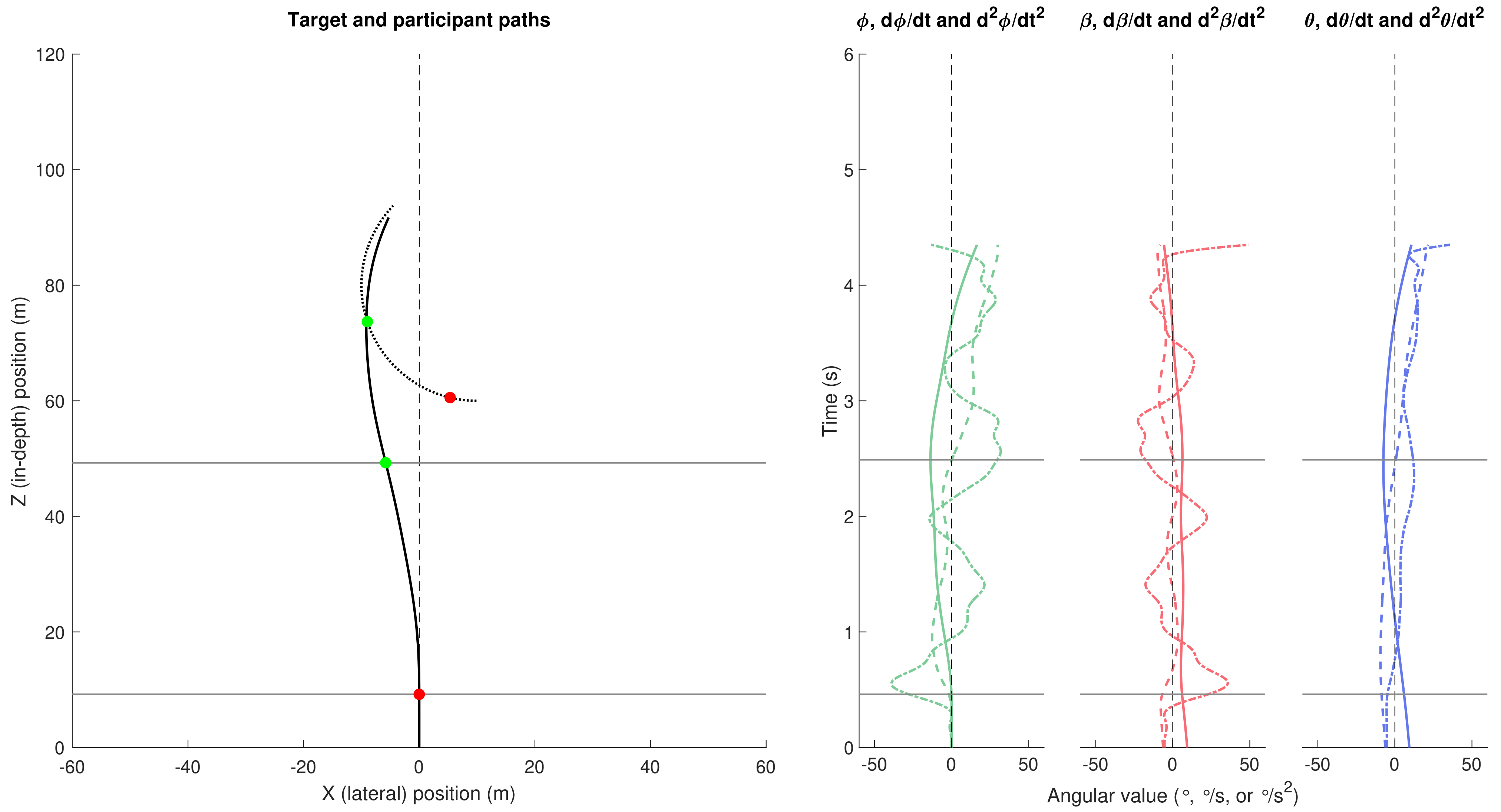

P6/B6
S10/R40-OUT

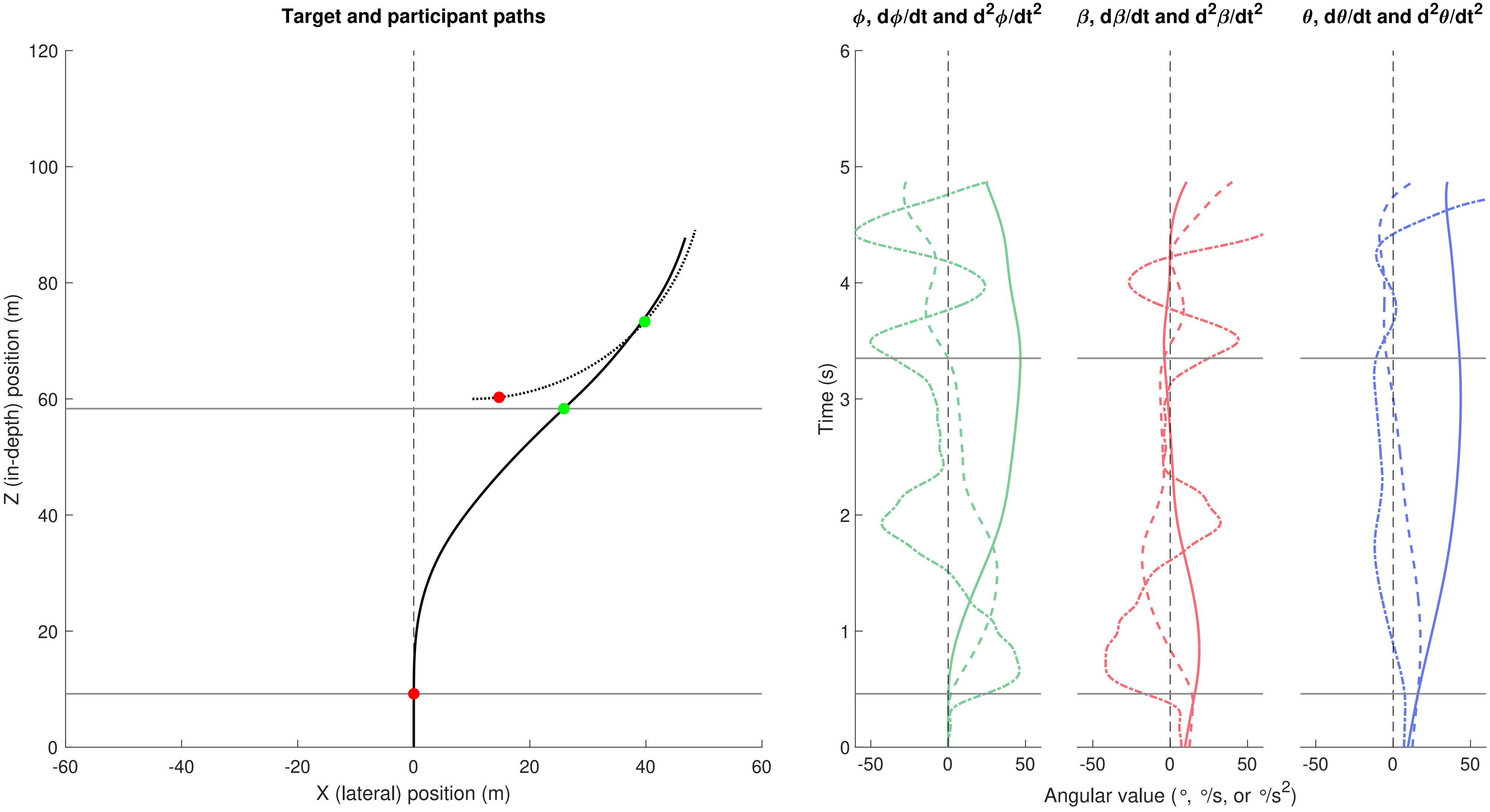

P6/B6
S10/R40-IN

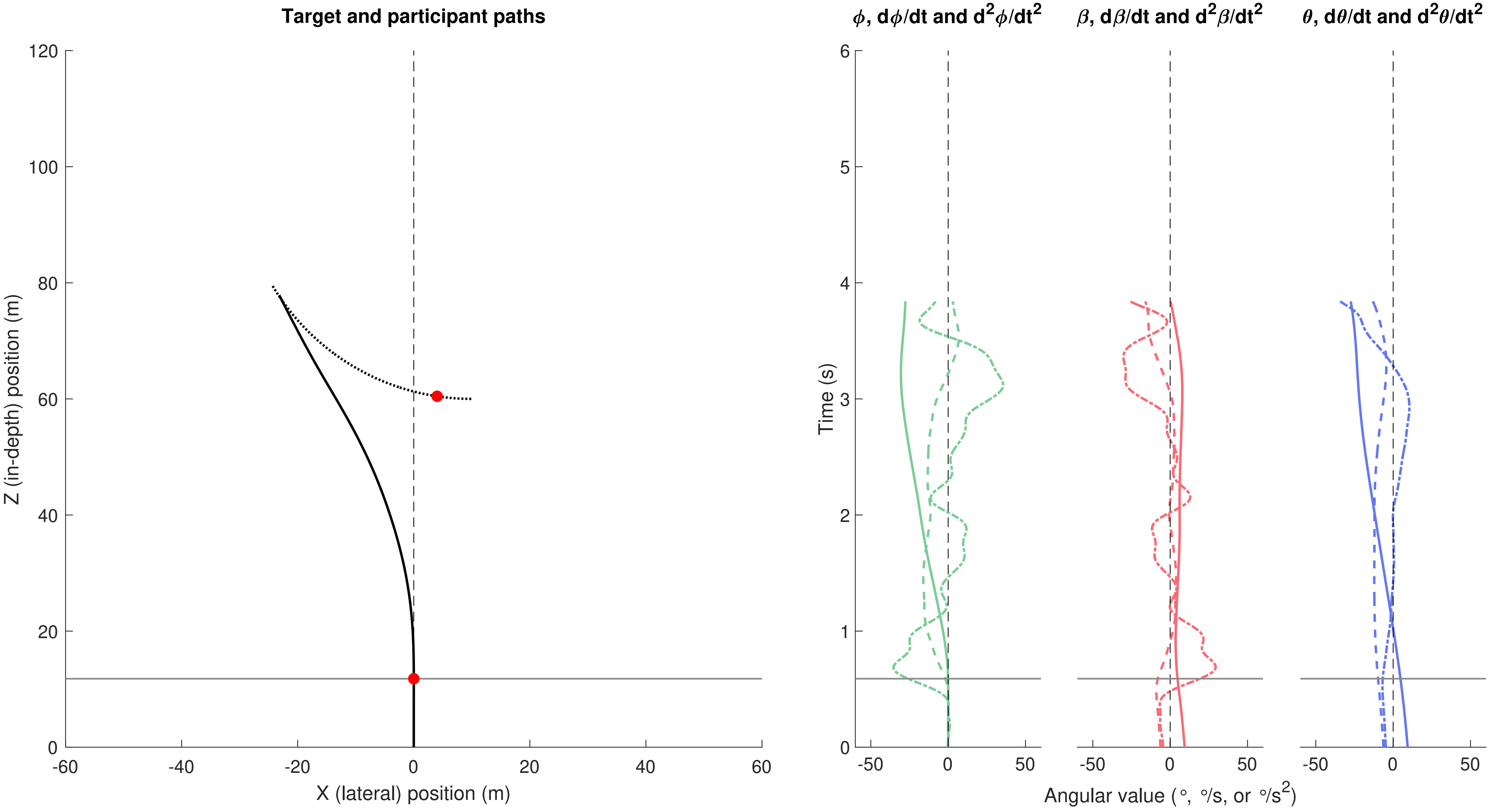

P6/B6
S20/R20-OUT

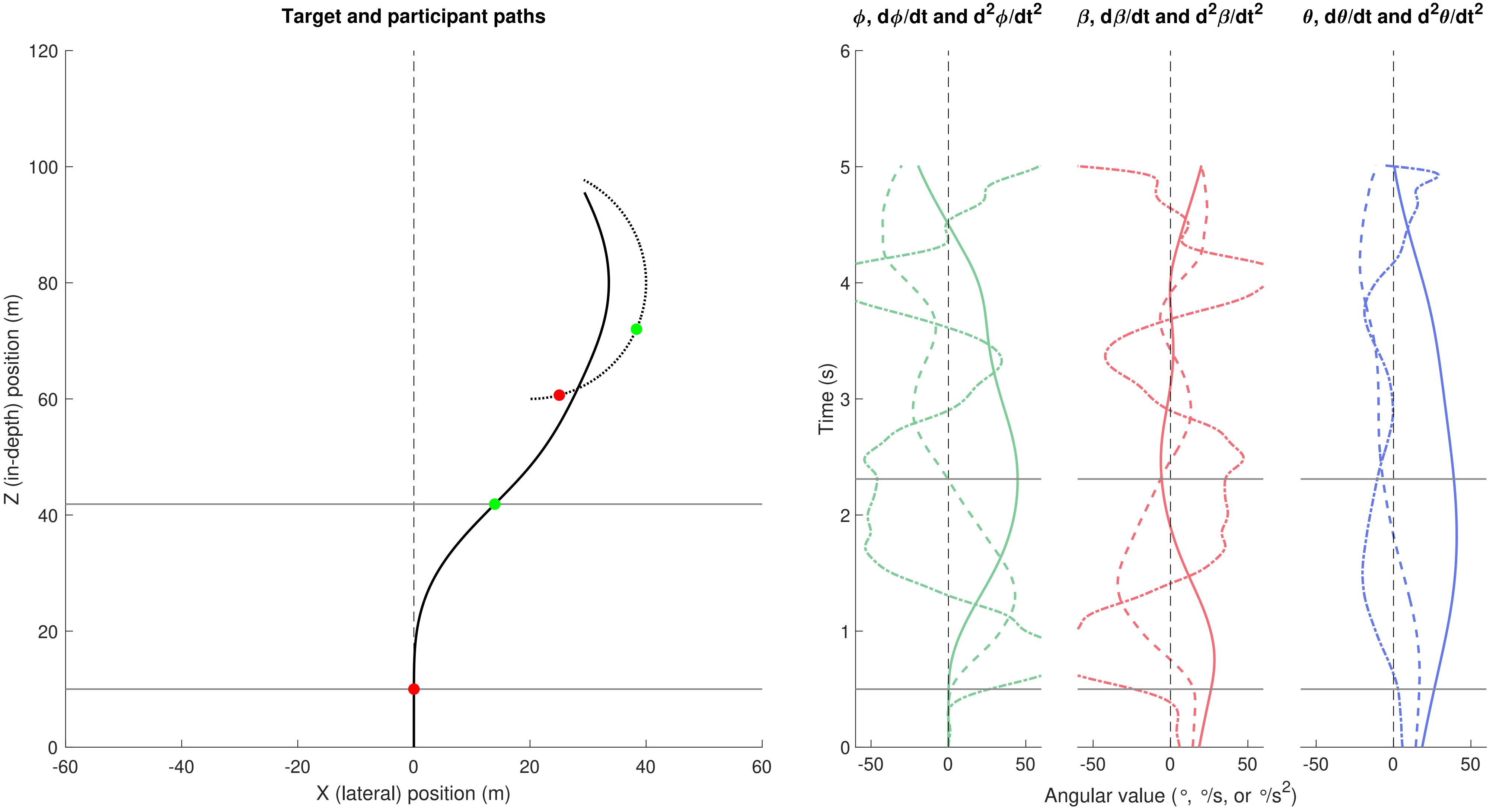

P6/B6
S20/R20-IN

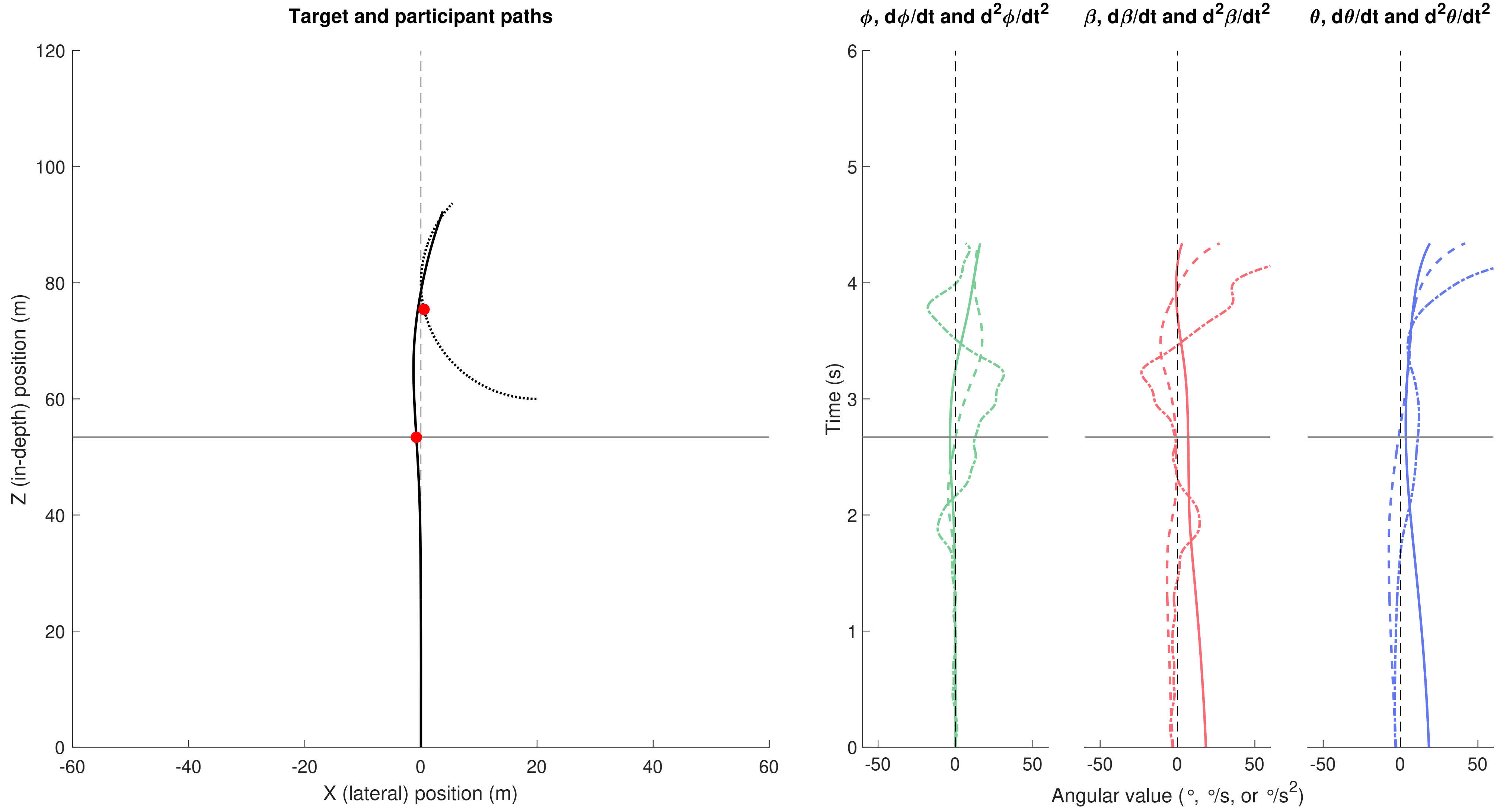

P6/B6
S20/R40-OUT

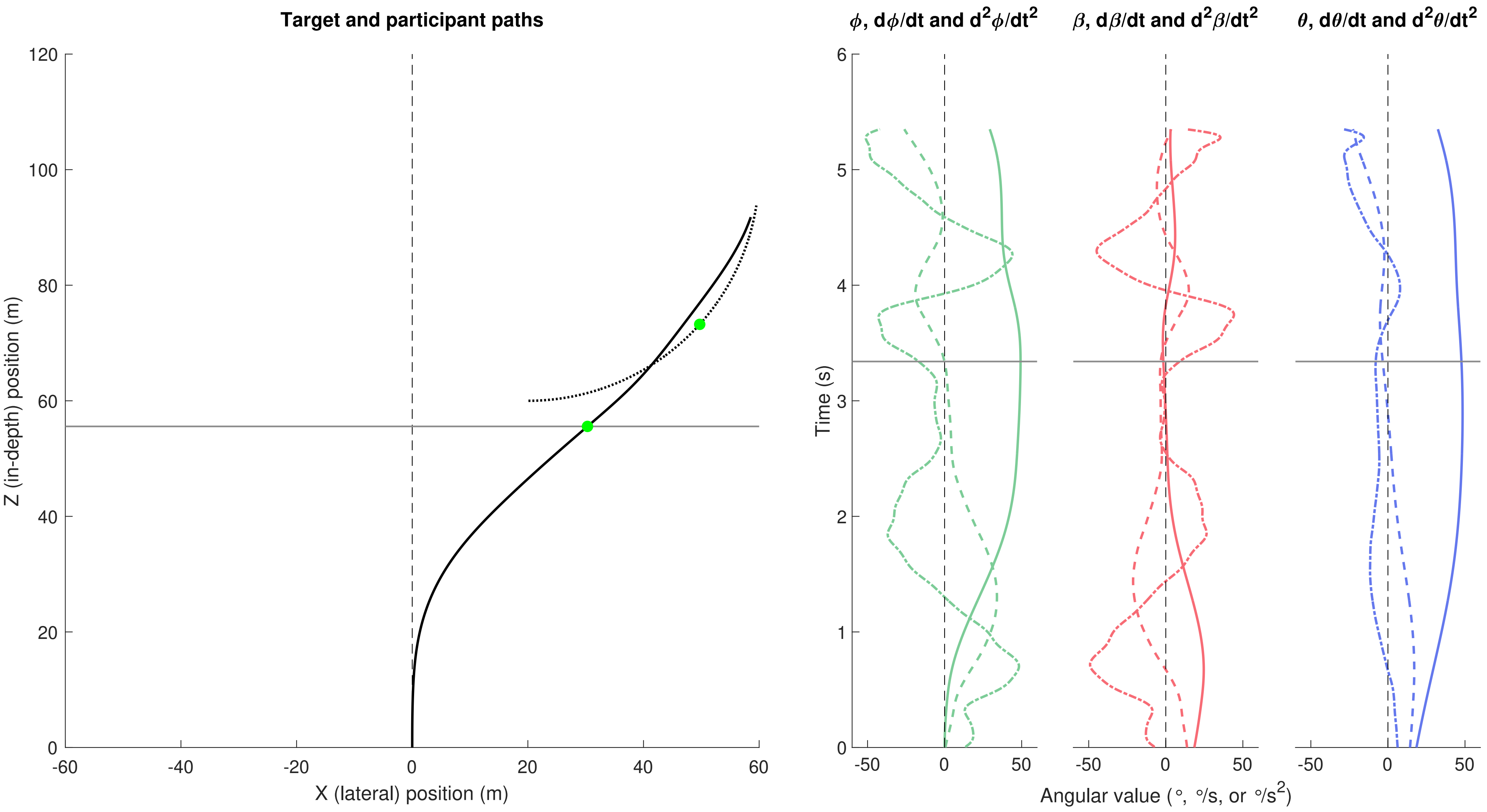

P6/B6
S20/R40-IN

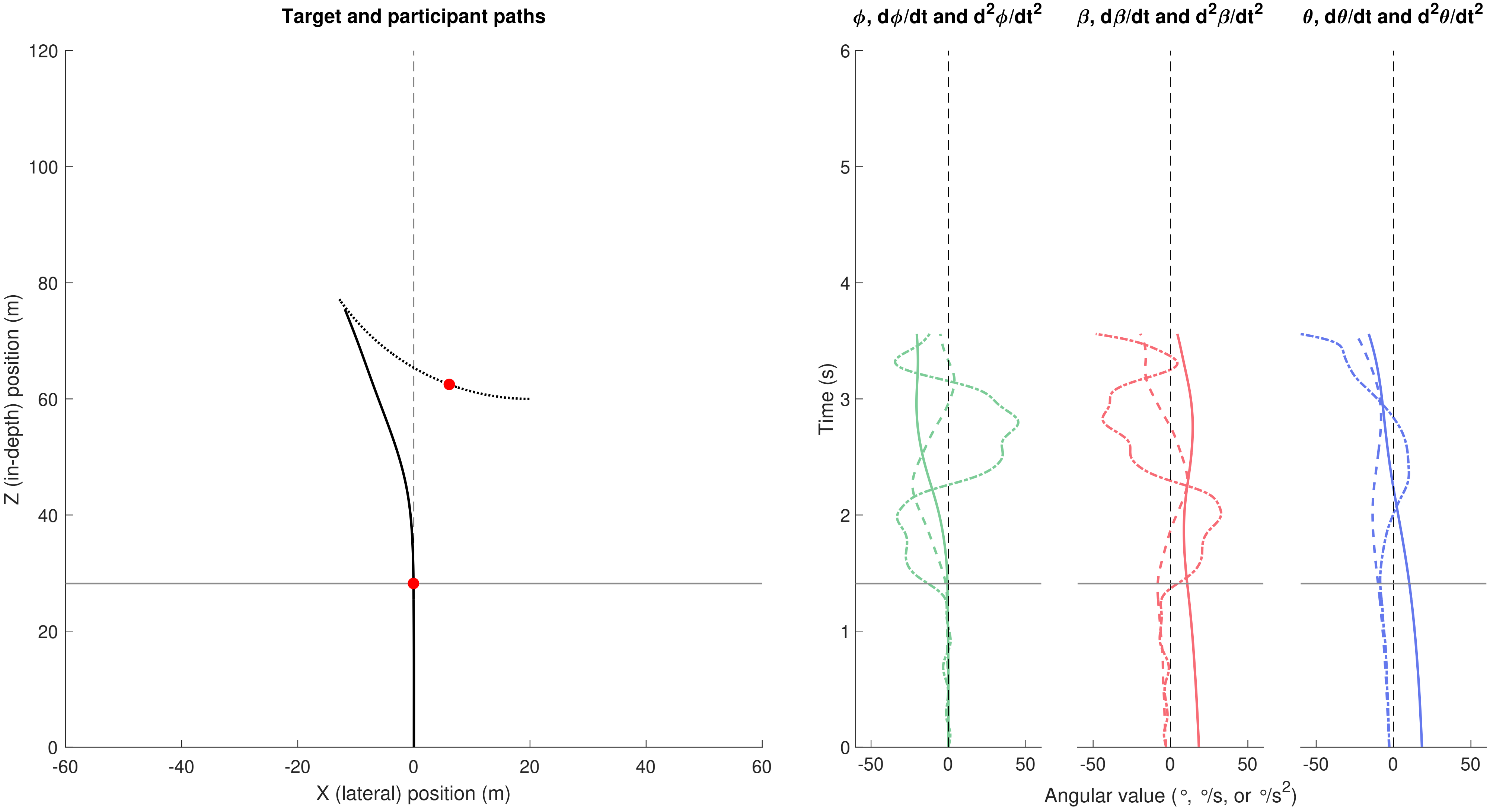

Supplement: Supplementary file 8 — Supplementary Information 8. [file 41598_2022_24625_MOESM8_ESM.pdf]
