# Supplementary material for: Characterisation of visual guidance of steering to intercept targets following curving trajectories using Qualitative Inconsistency Detection
Source: Sci Rep. 2022 Nov 24;12:20246. doi: 10.1038/s41598-022-24625-4 (PMC9691627; doi:10.1038/s41598-022-24625-4)
Supplement: Supplementary file 15 — Supplementary Information 15. [file 41598_2022_24625_MOESM15_ESM.pdf]

Supplementary Figure S2-P13. Page-size landscape-oriented QuID plots for all 120 individual trials of Participant 13 (one QuID plot per page)

Figure (subplot) titles: Located top-centre on each Figure page, with identification code of the specific trial (e.g., p. 2: P13/B1 S20/R20-IN) where P refers to Participant number (1 to 14), B refers to Block number (1 to 6) and S20/R20-IN refers to target trajectory characteristics (see Table 1 in main text).

Figure legends. Identical for all 120 subplots

Left graph on each page: Spatial paths followed by the target (dotted grey line) and the participant (black line). Steering events are marked by colour-coded dots.

Right graphs on each page: Time evolution (bottom to top) over the course of the trial of the participant's heading direction ϕ (in green), the target-heading angle β (in red) and the target's bearing angle θ (in blue) together with their first-order (dashed) and second-order (dash-dotted) time derivatives.

Horizontal grey lines situate the steering events spatially (left graph) and temporally (right graphs).

P13/B1
S20/R20-IN

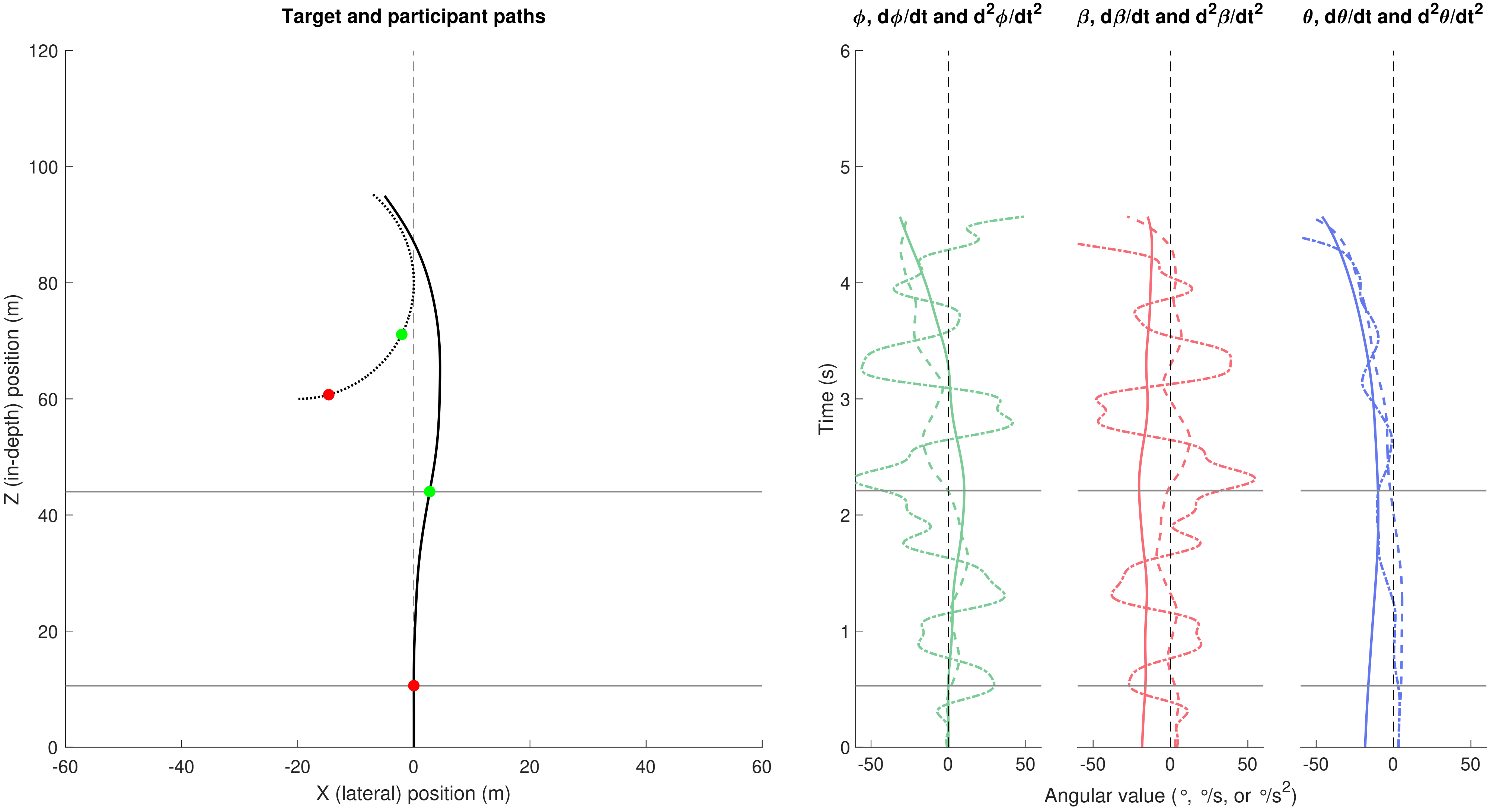

P13/B1
S20/R20-OUT

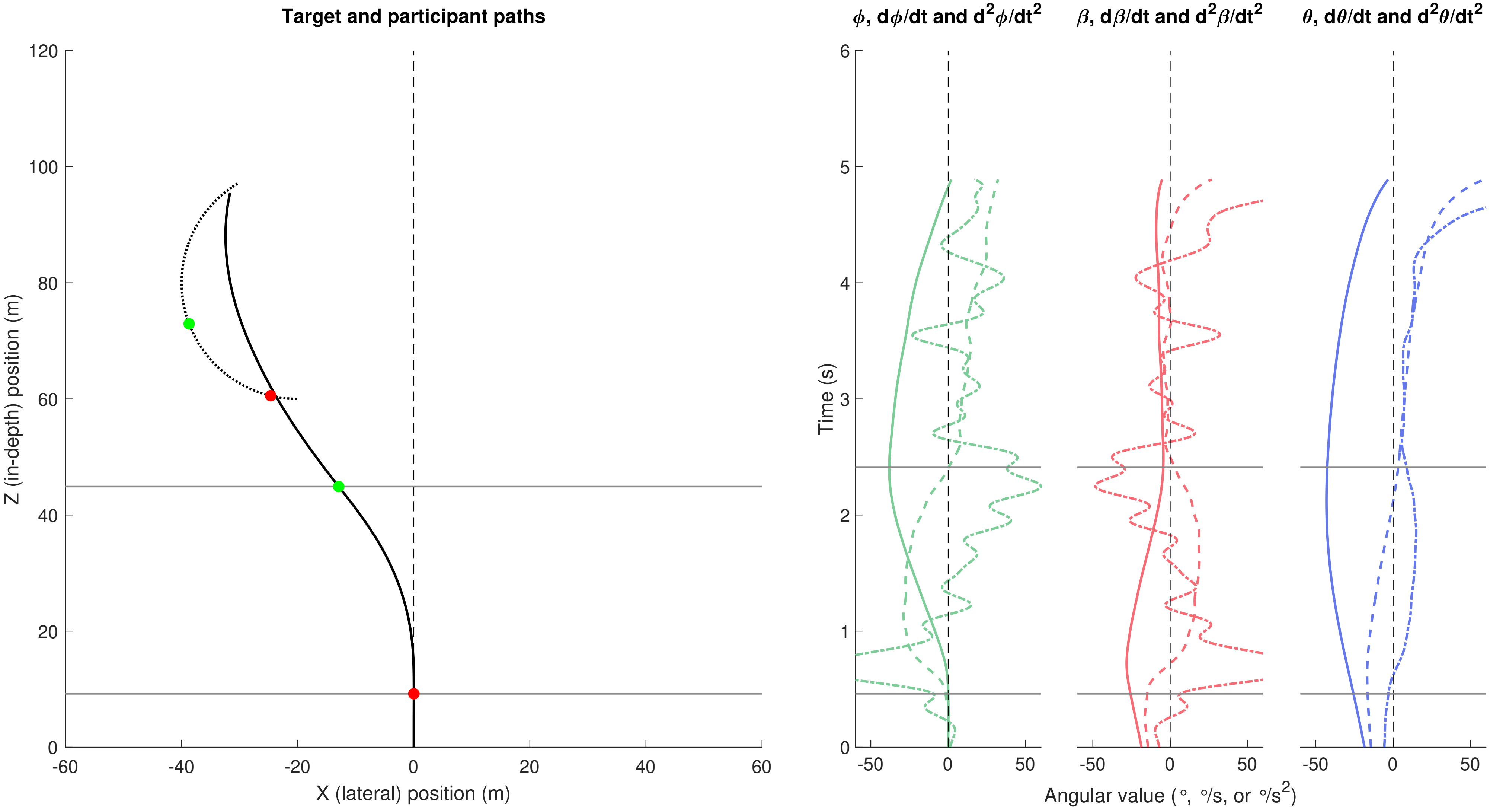

P13/B1
S20/R40-IN

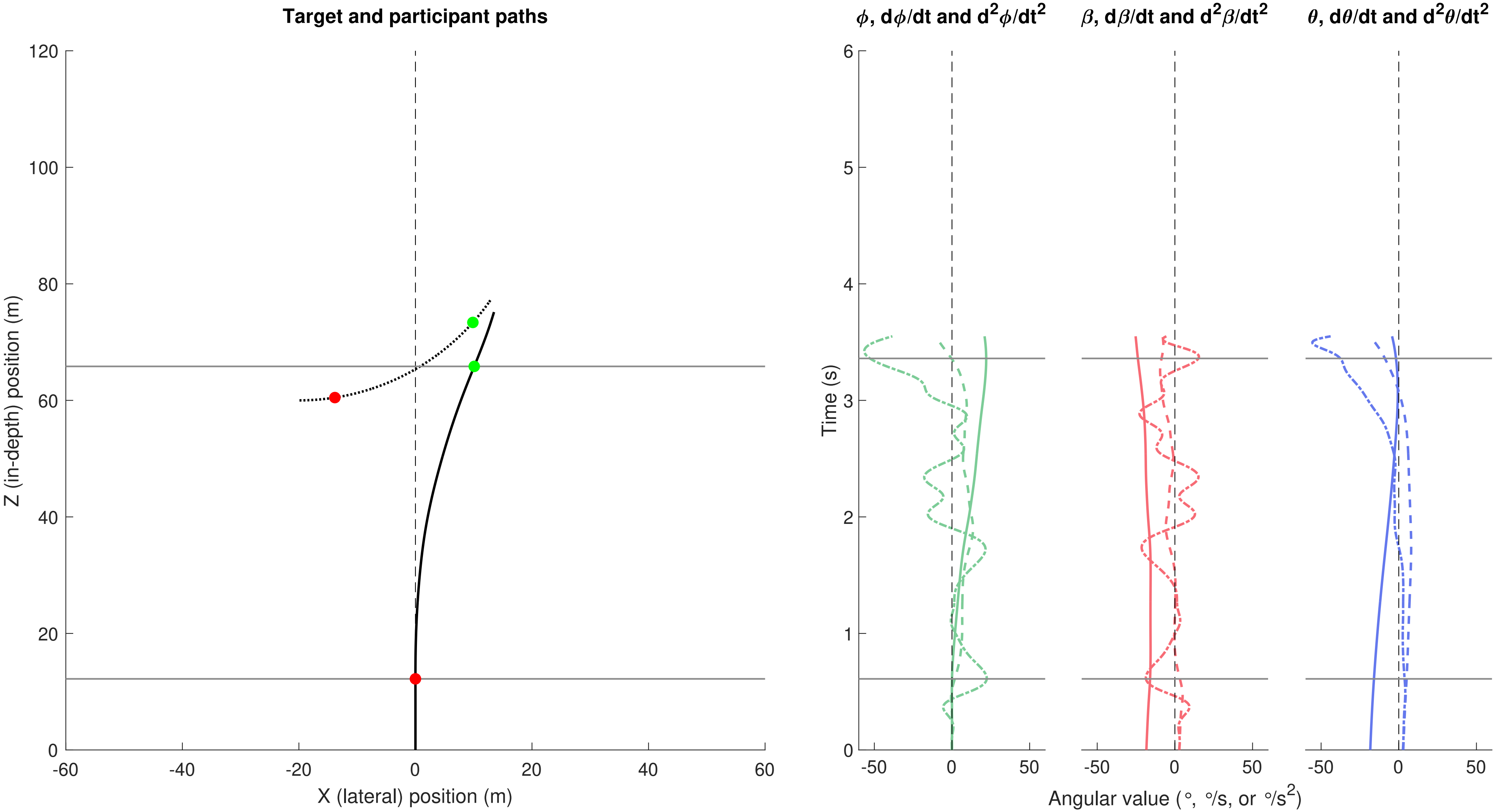

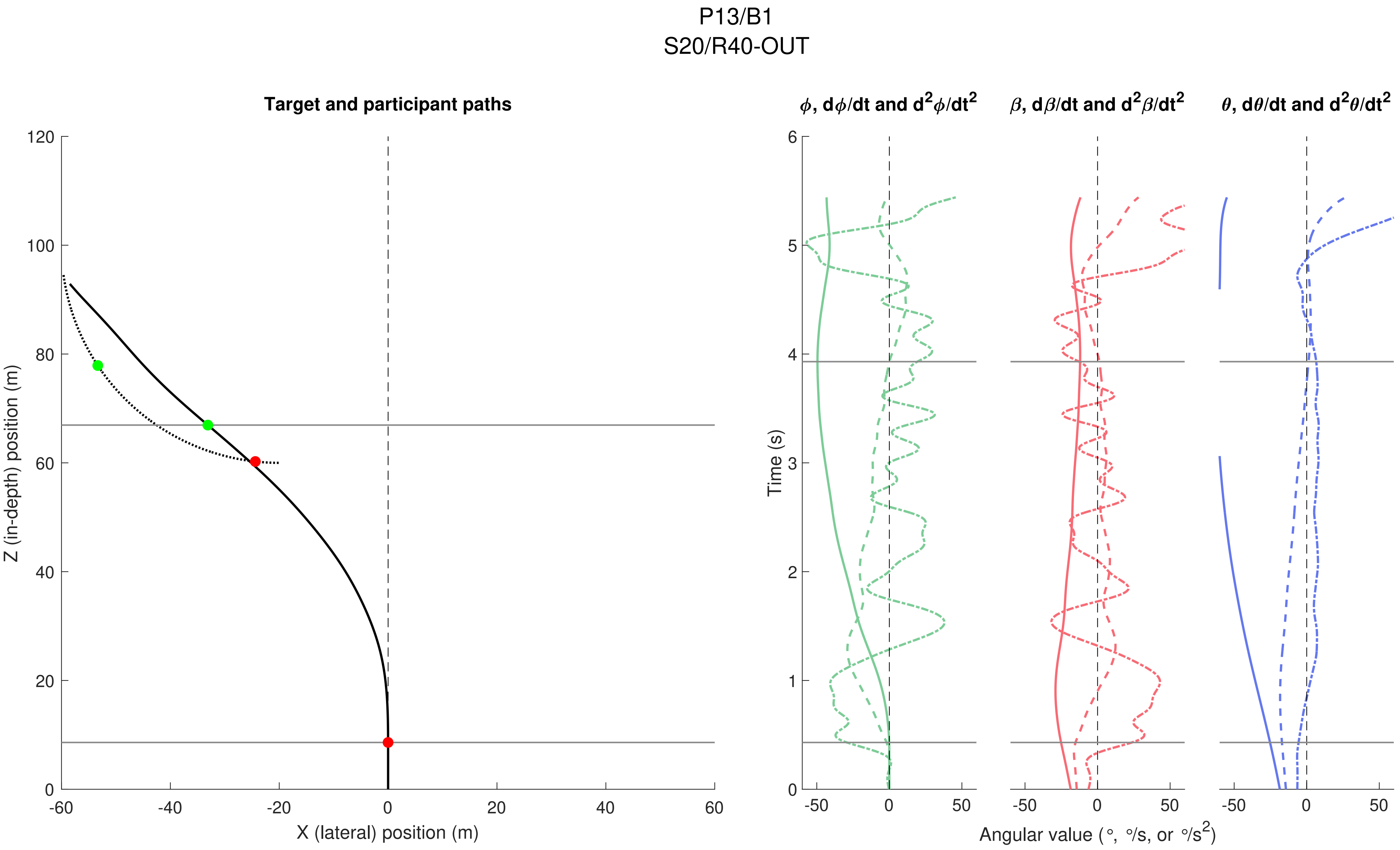
P13/B1
S20/R40-OUT
Target and participant paths
Z (in-depth) position (m)
X (lateral) position (m)
120
100
80
60
40
20
0
-60
-40
-20
0
20
40
60
φ, dφ/dt and d²φ/dt²
β, dβ/dt and d²β/dt²
θ, dθ/dt and d²θ/dt²
Time (s)
6
5
4
3
2
1
0
-50
0
50
Angular value (°, °/s, or °/s²)

P13/B1
S10/R20-IN

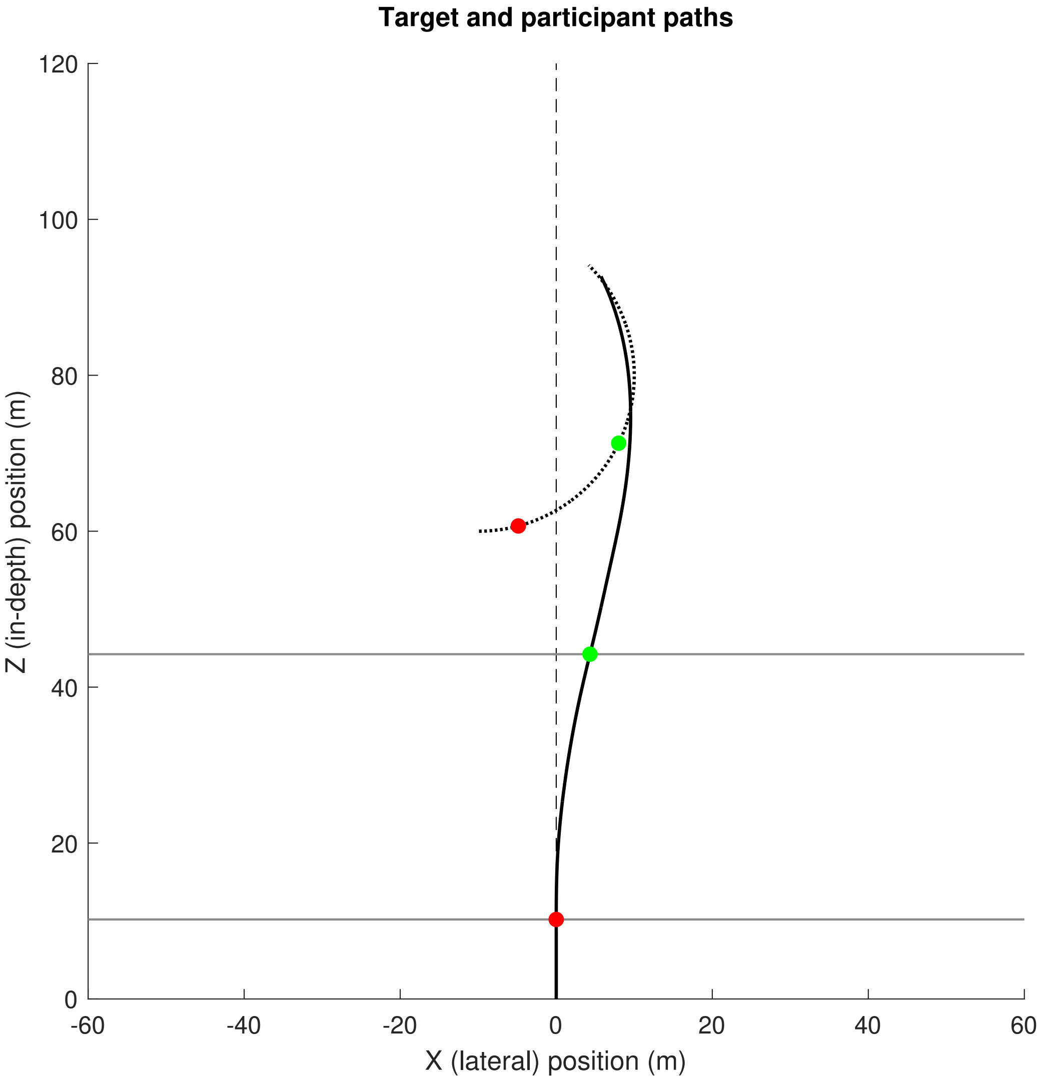

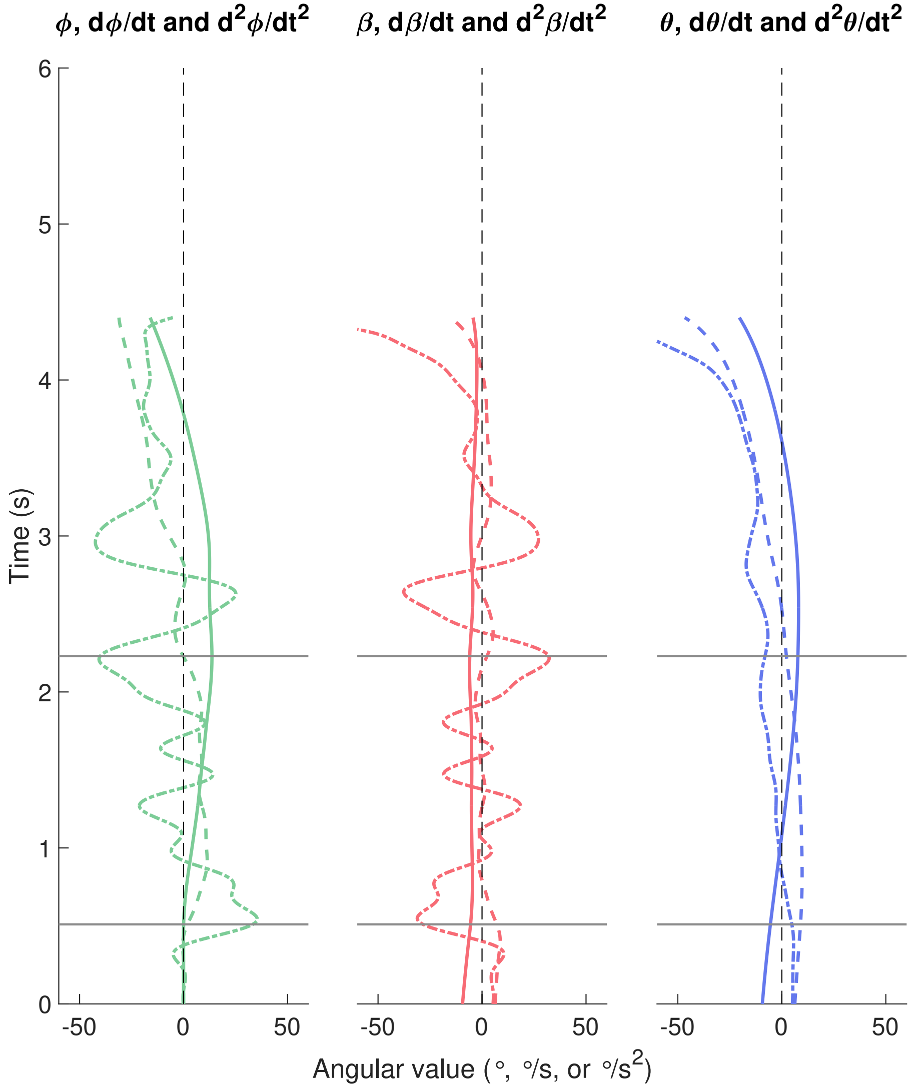

P13/B1
S10/R20-OUT

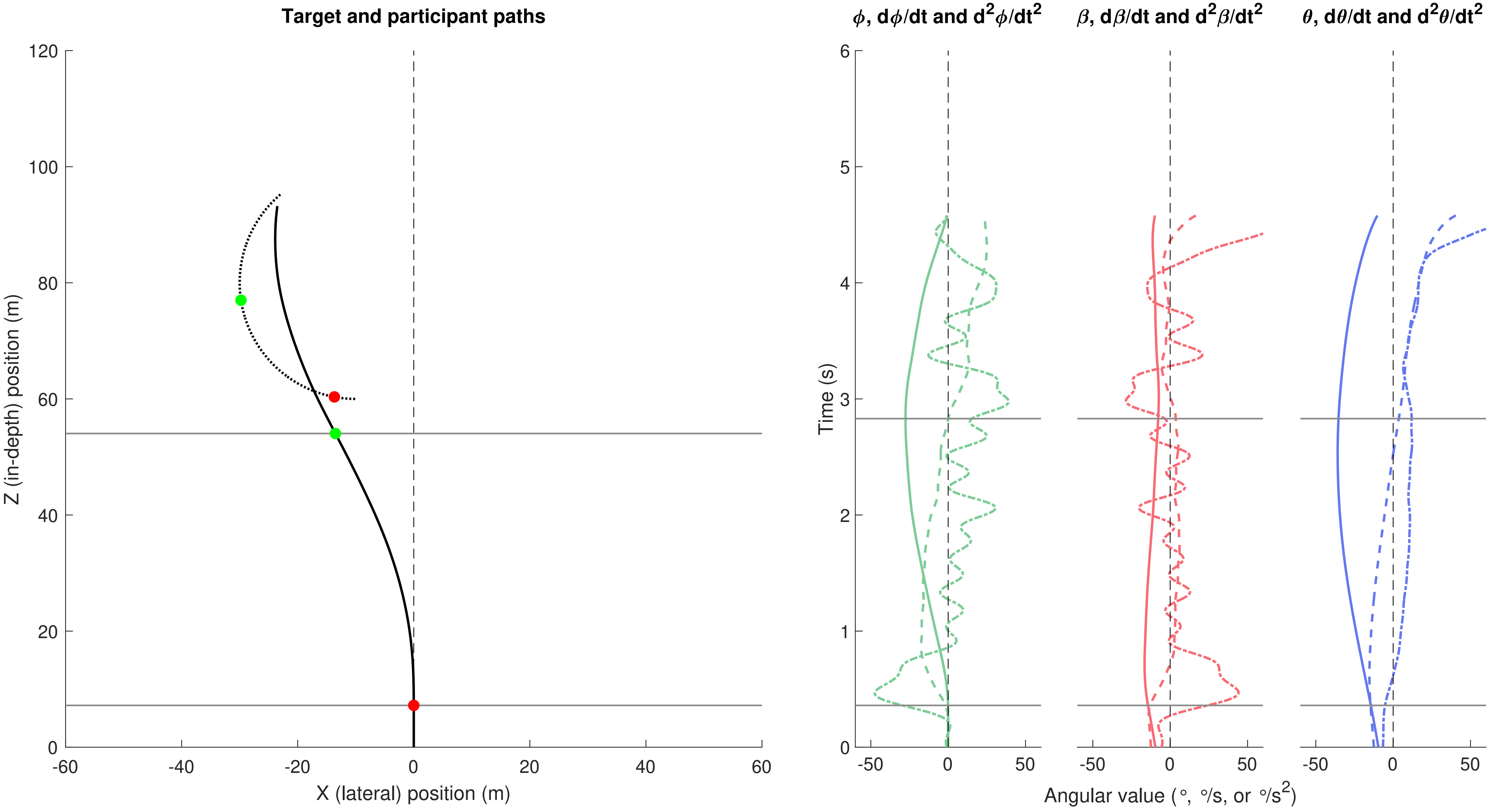

P13/B1
S10/R40-IN

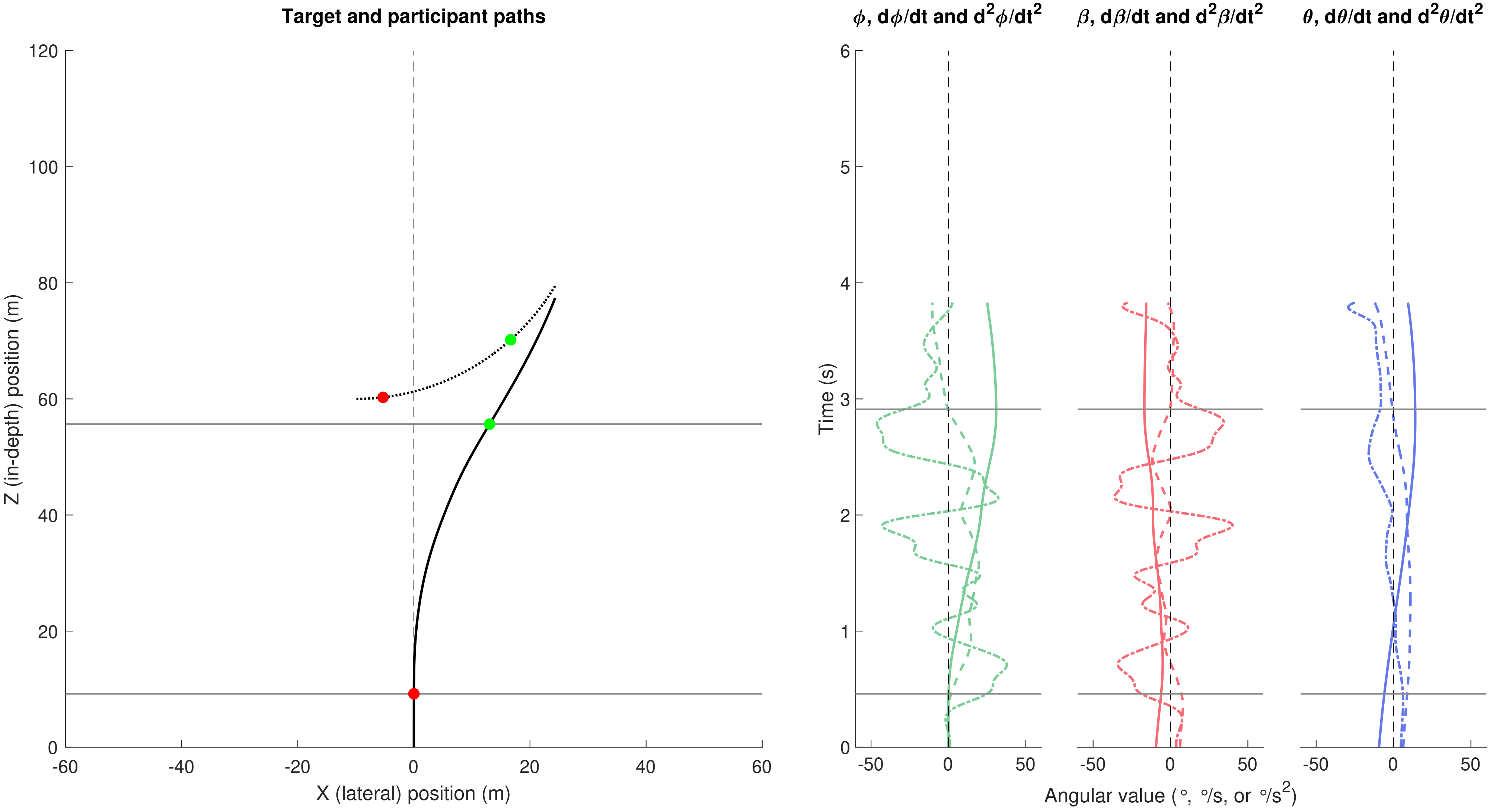

P13/B1
S10/R40-OUT

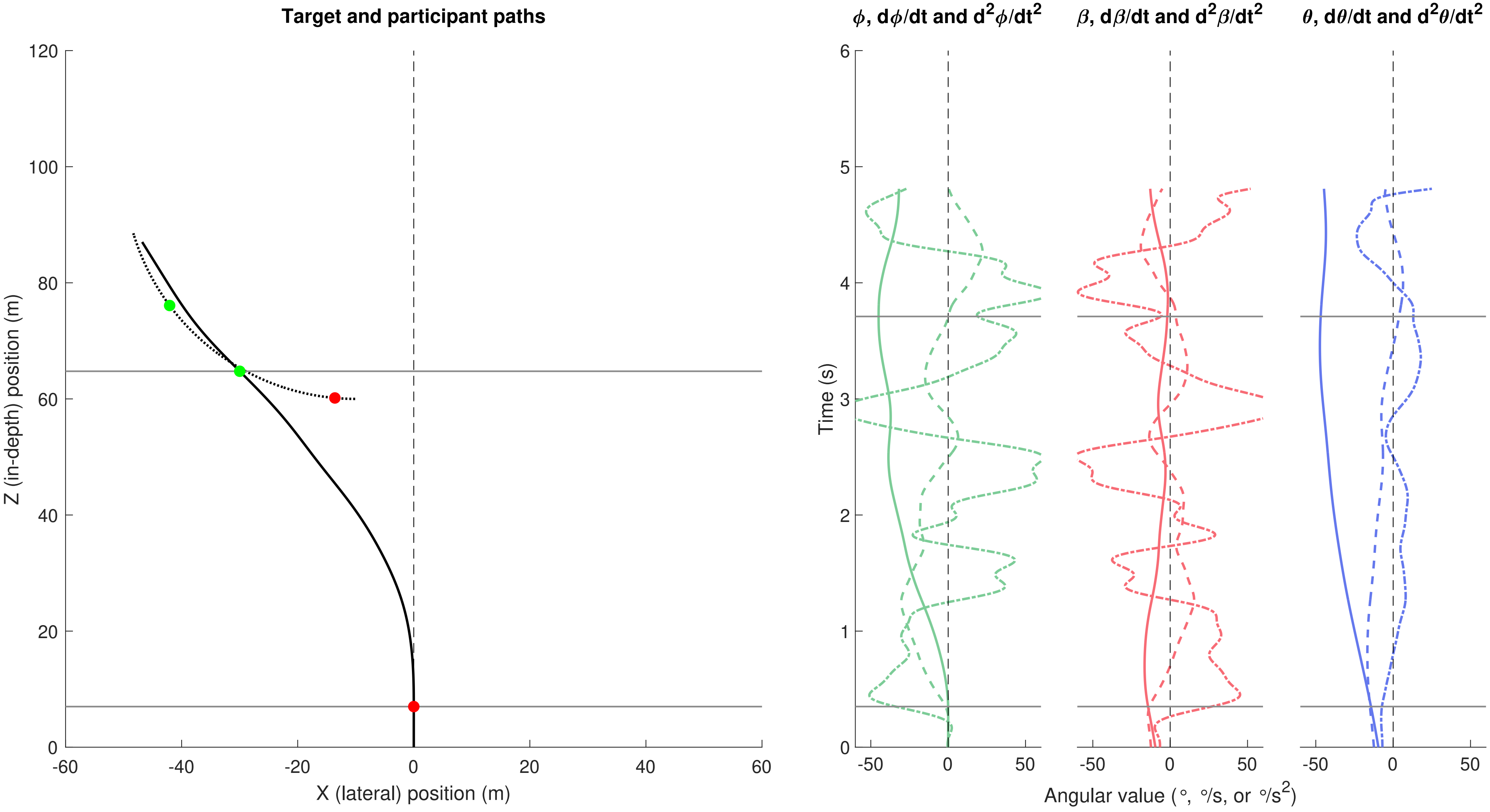

P13/B1
S0/R20-OUT

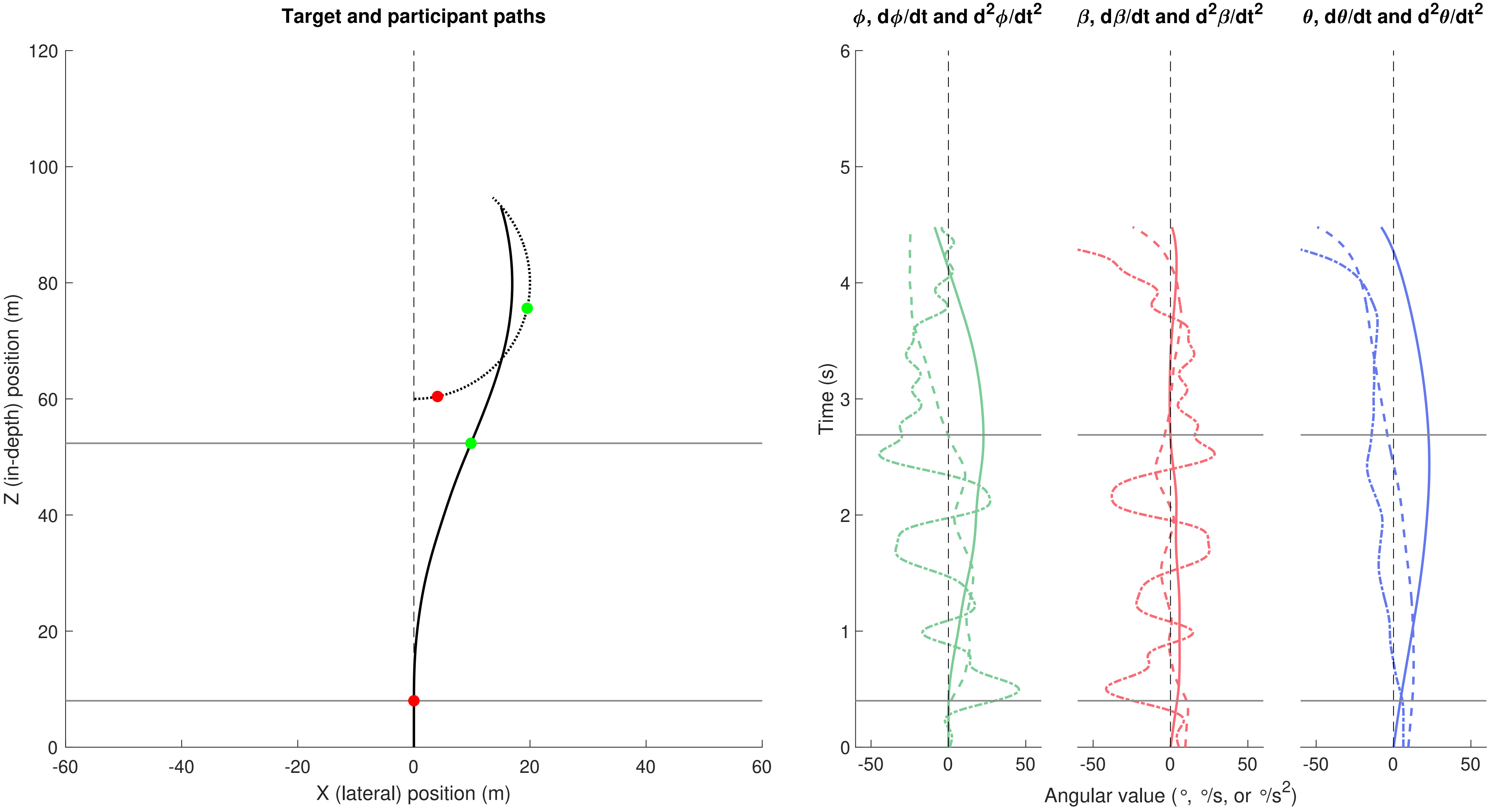

P13/B1
S0/R20-OUT

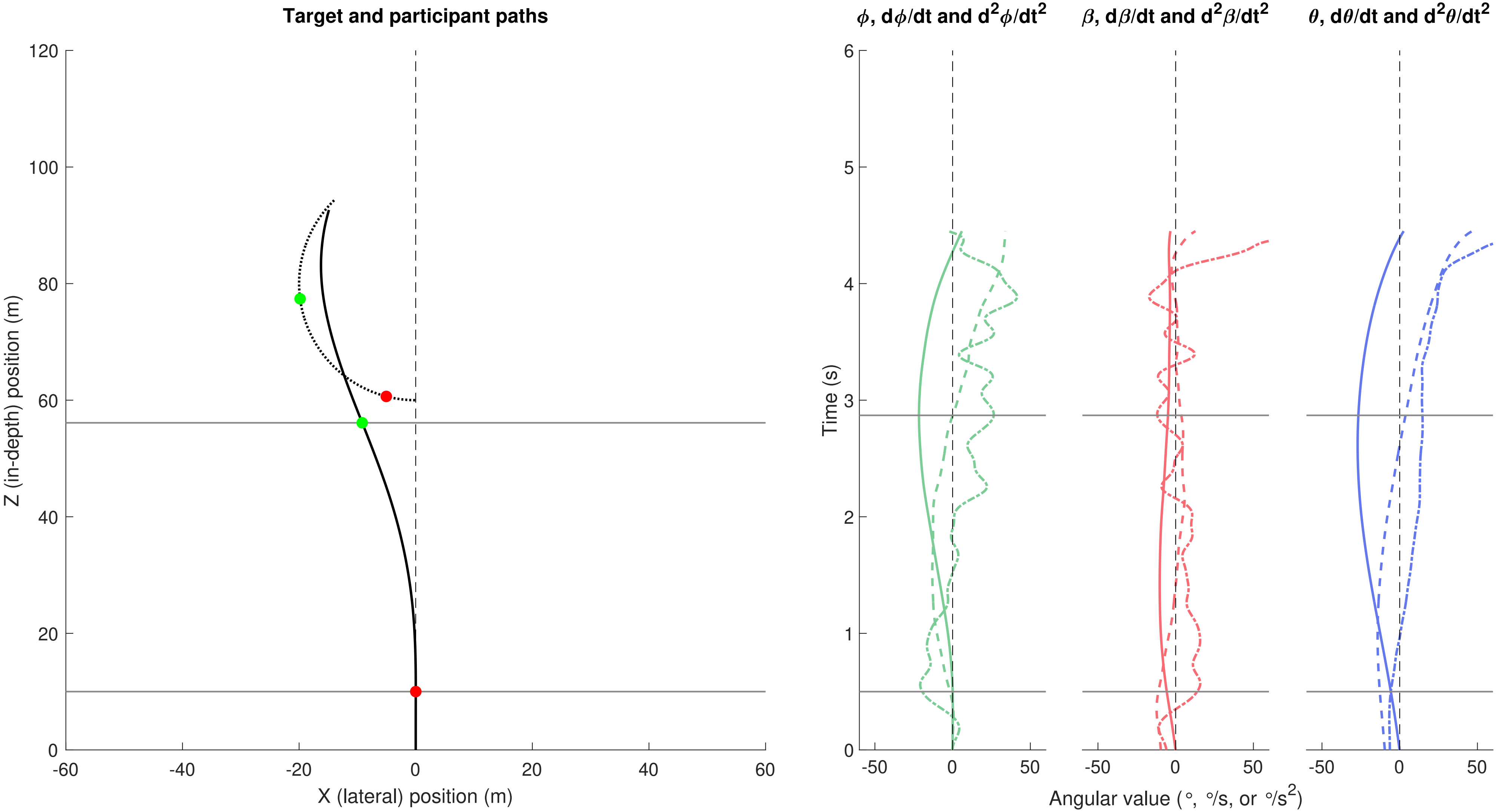

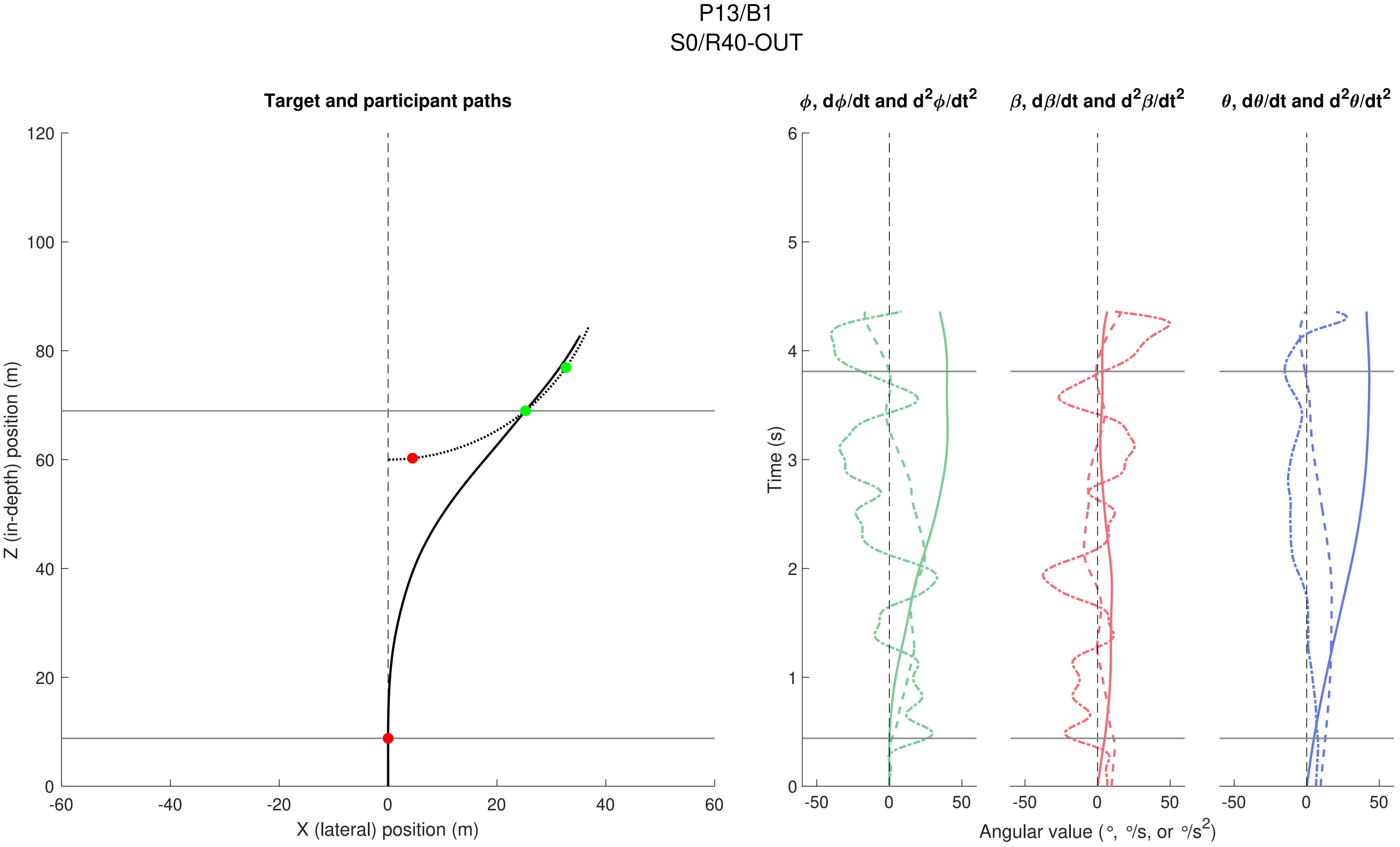

P13/B1
S0/R40-OUT
Target and participant paths
Z (in-depth) position (m)
X (lateral) position (m)
φ, dφ/dt and d²φ/dt²
β, dβ/dt and d²β/dt²
θ, dθ/dt and d²θ/dt²
Time (s)
Angular value (°, °/s, or °/s²)

P13/B1
S0/R40-OUT

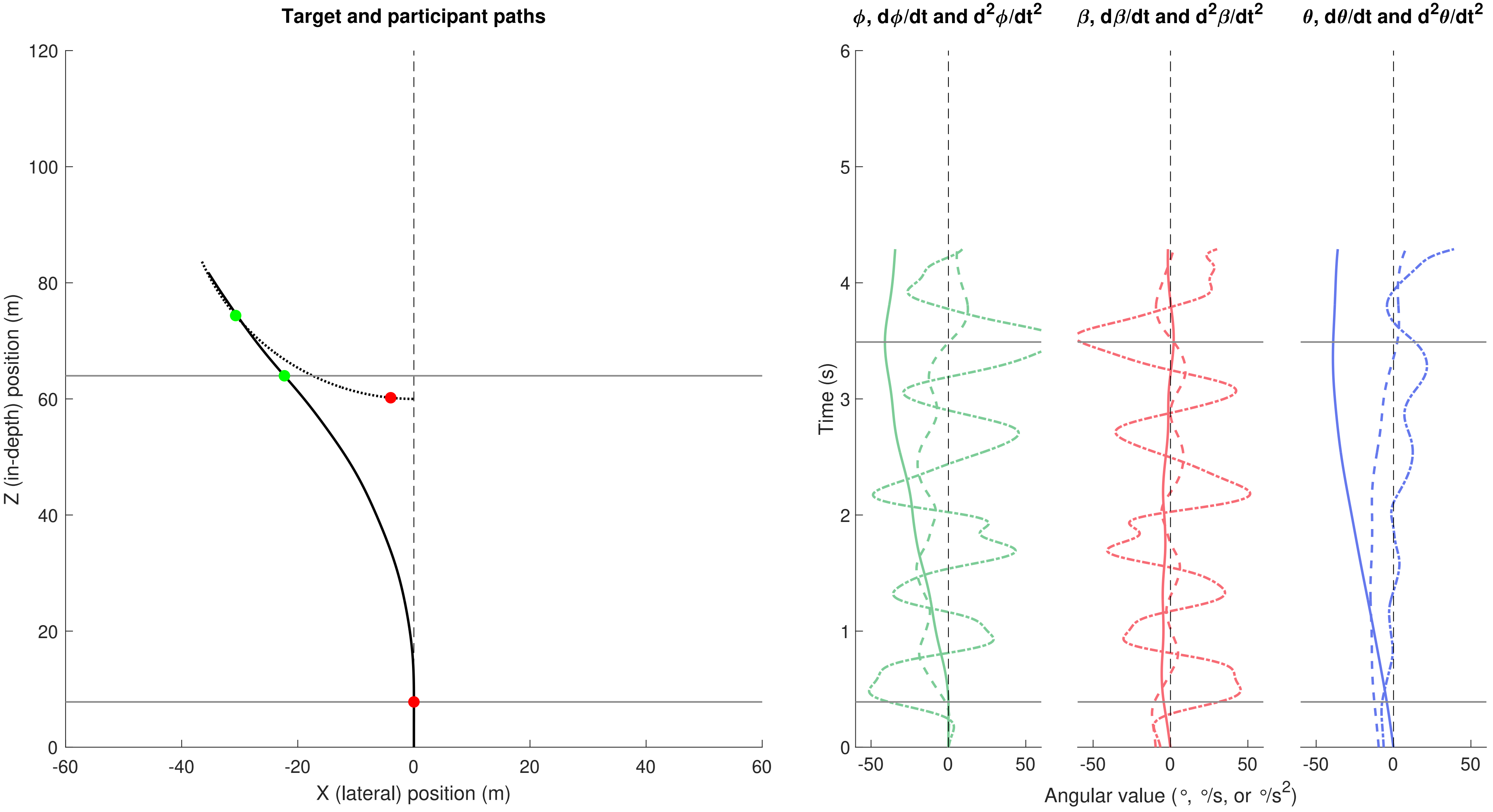

P13/B1
S10/R20-OUT

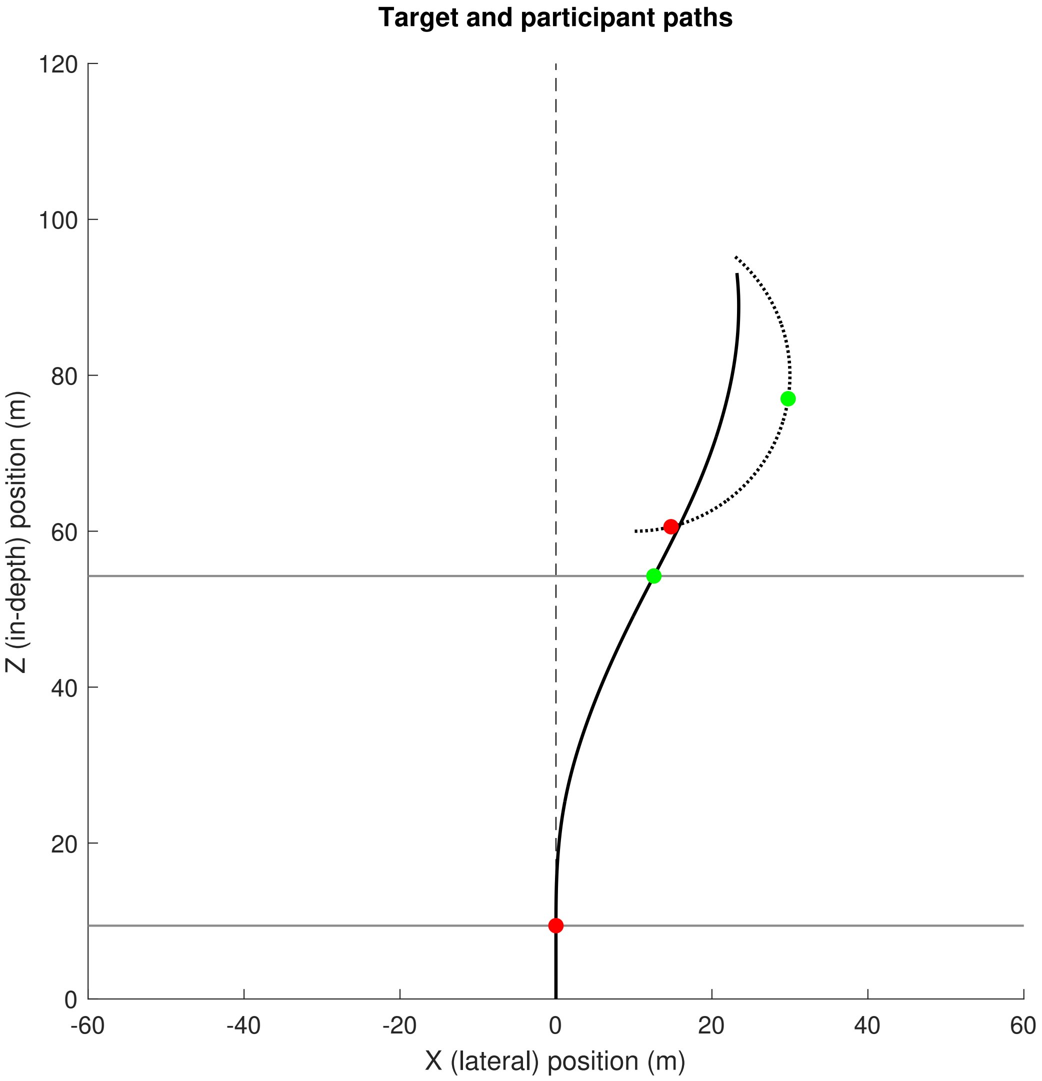

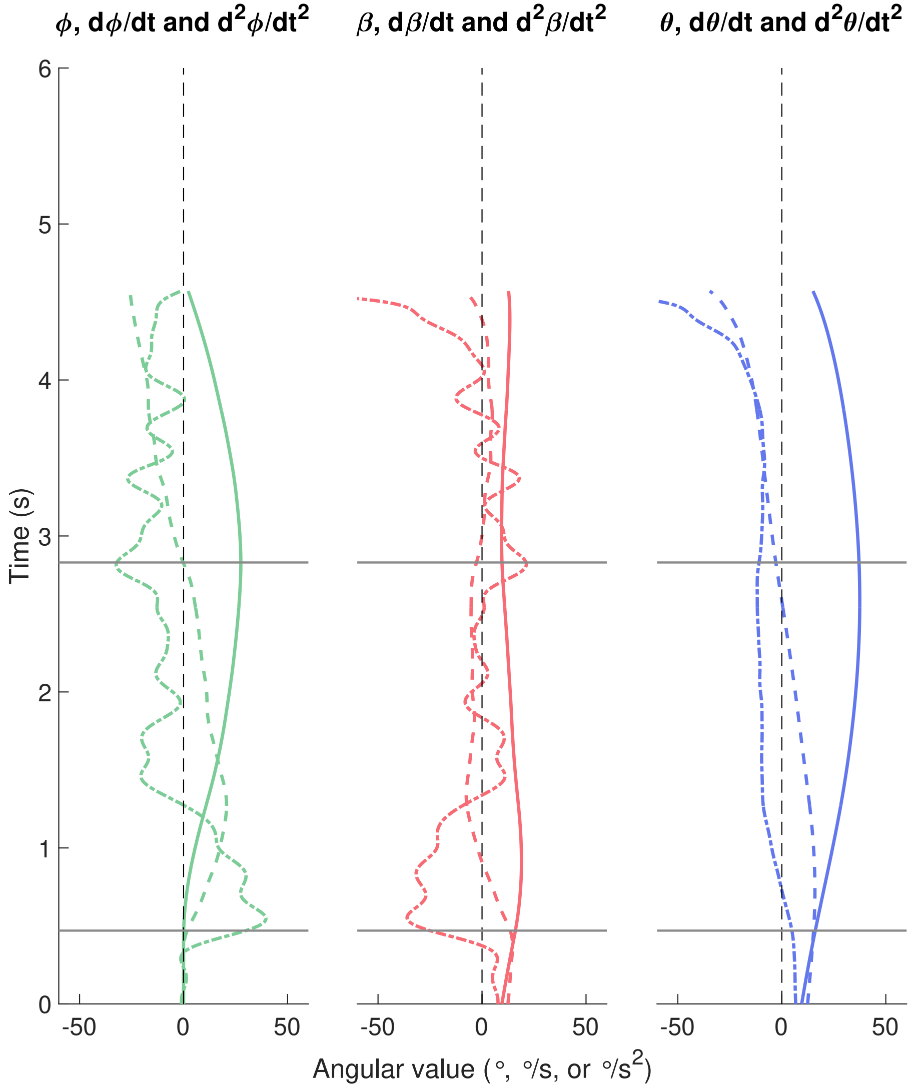

P13/B1
S10/R20-IN

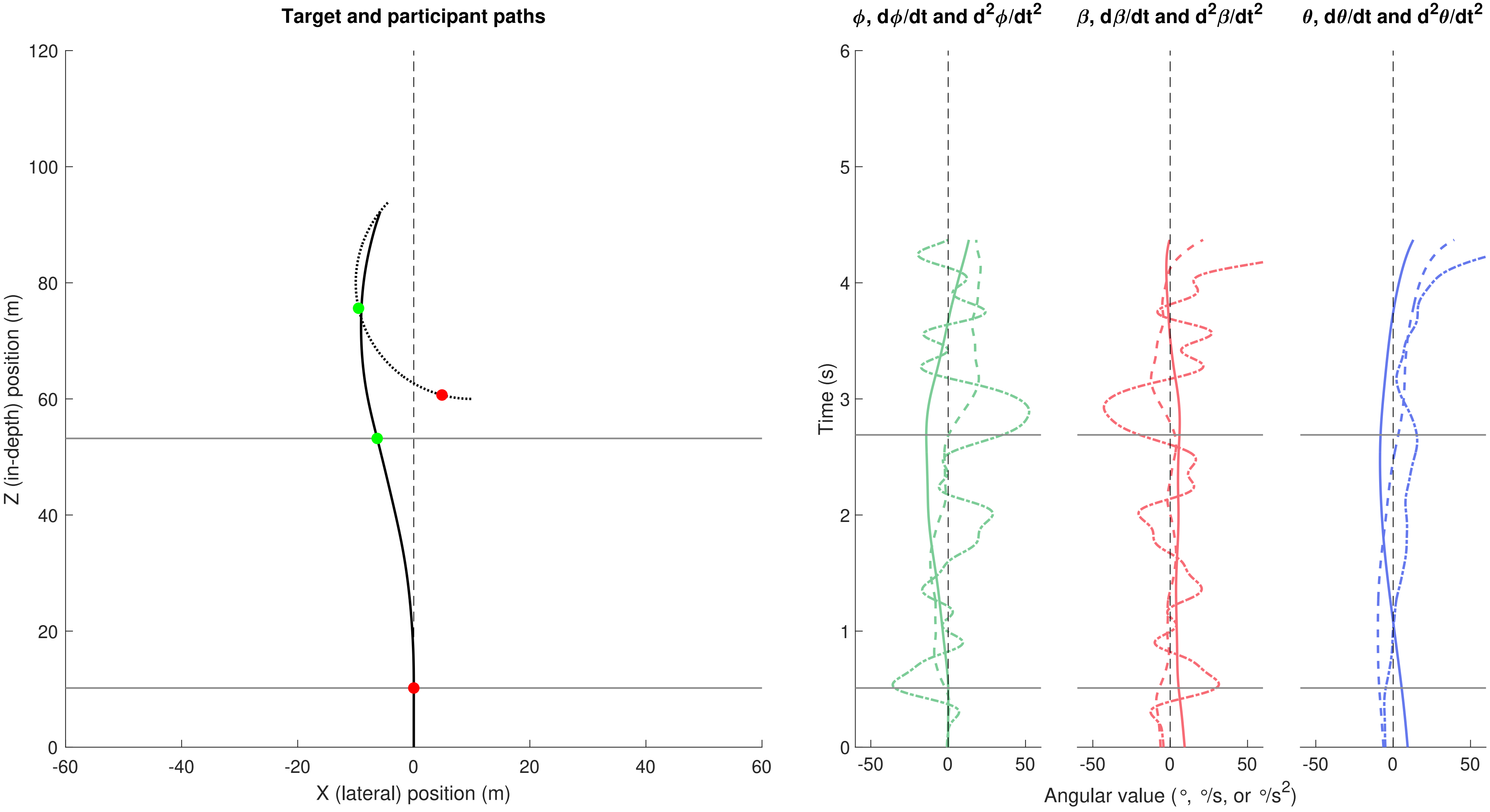

P13/B1
S10/R40-OUT

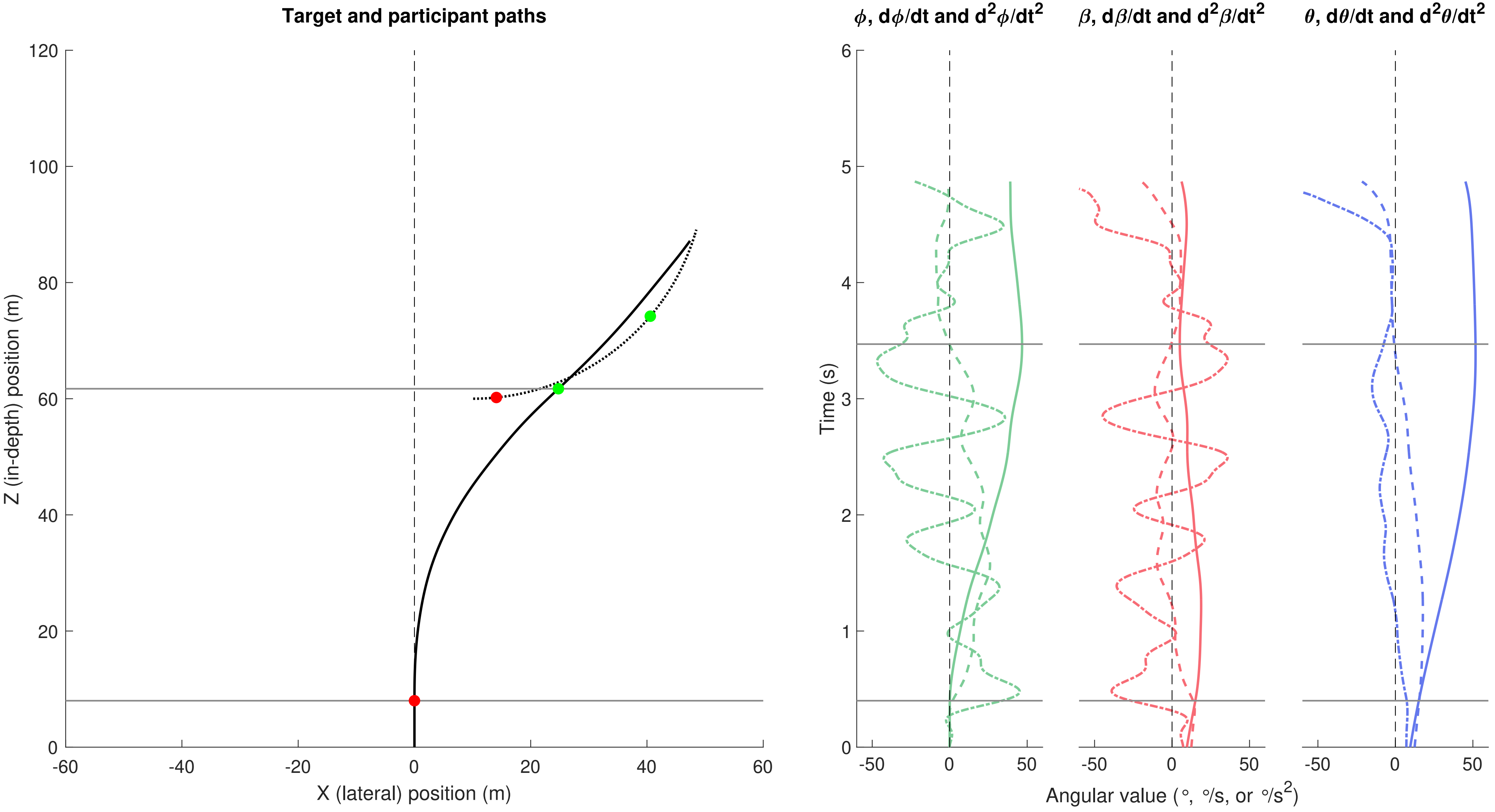

P13/B1
S10/R40-IN

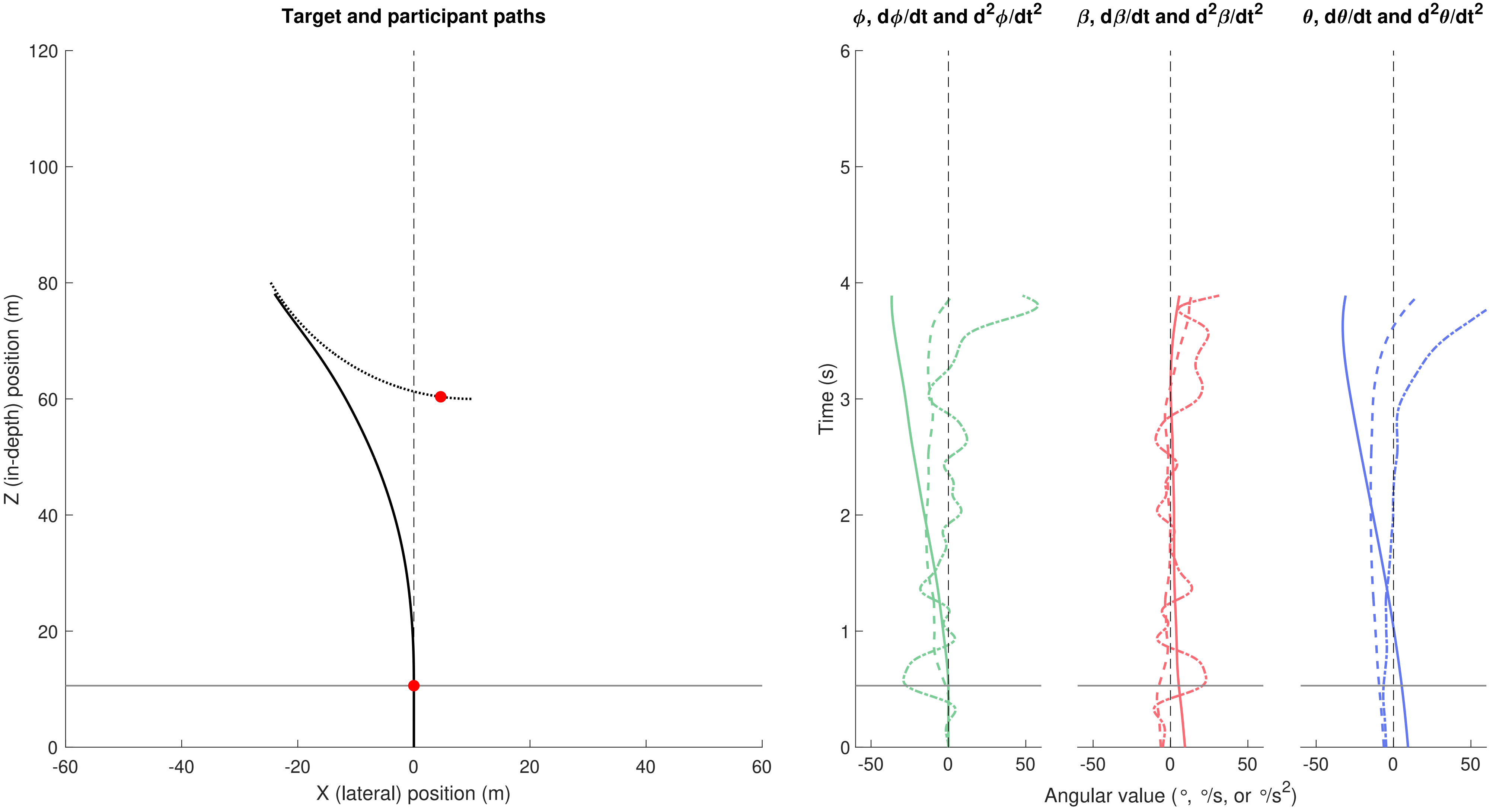

P13/B1
S20/R20-OUT

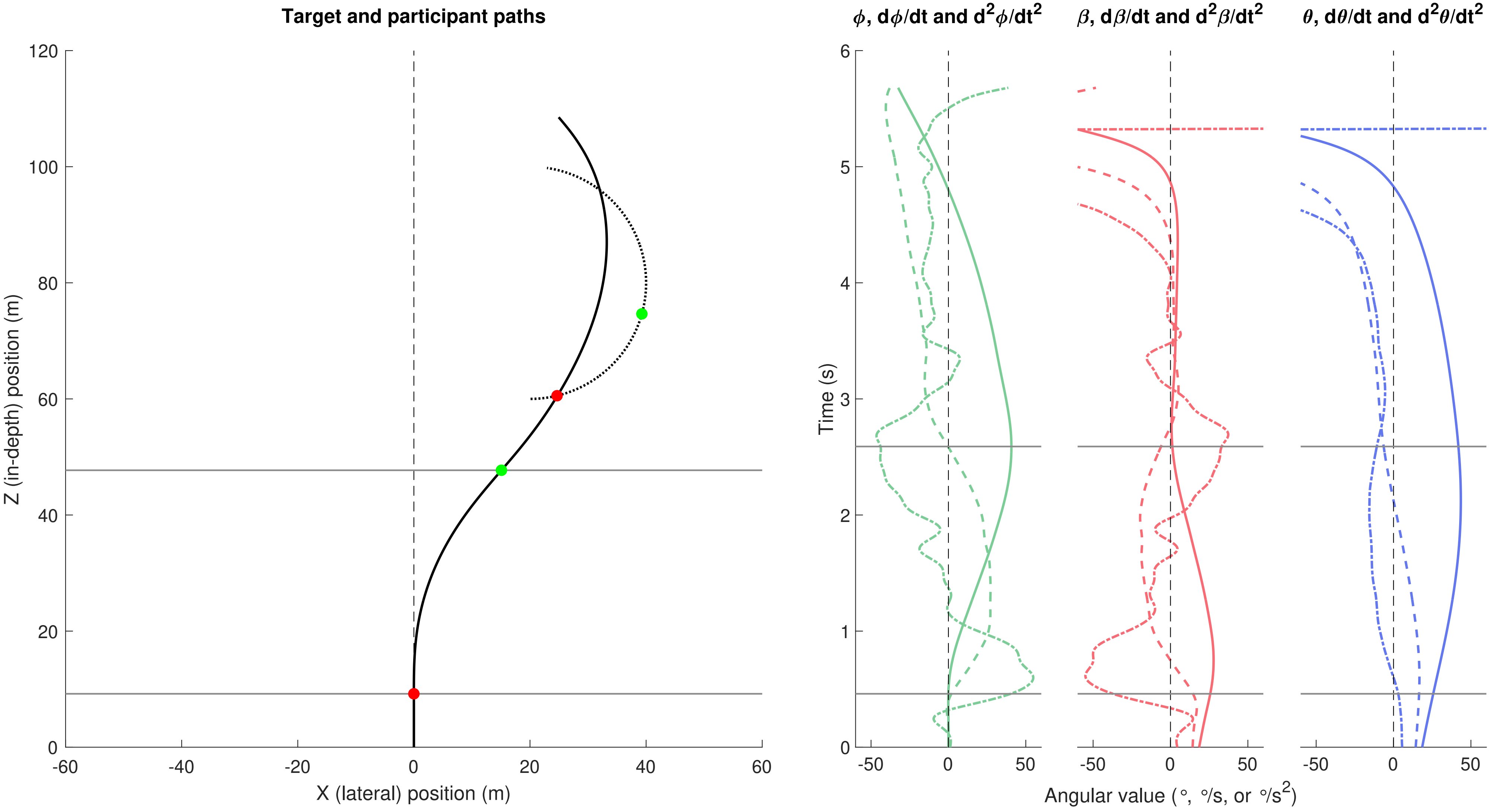

P13/B1
S20/R20-IN

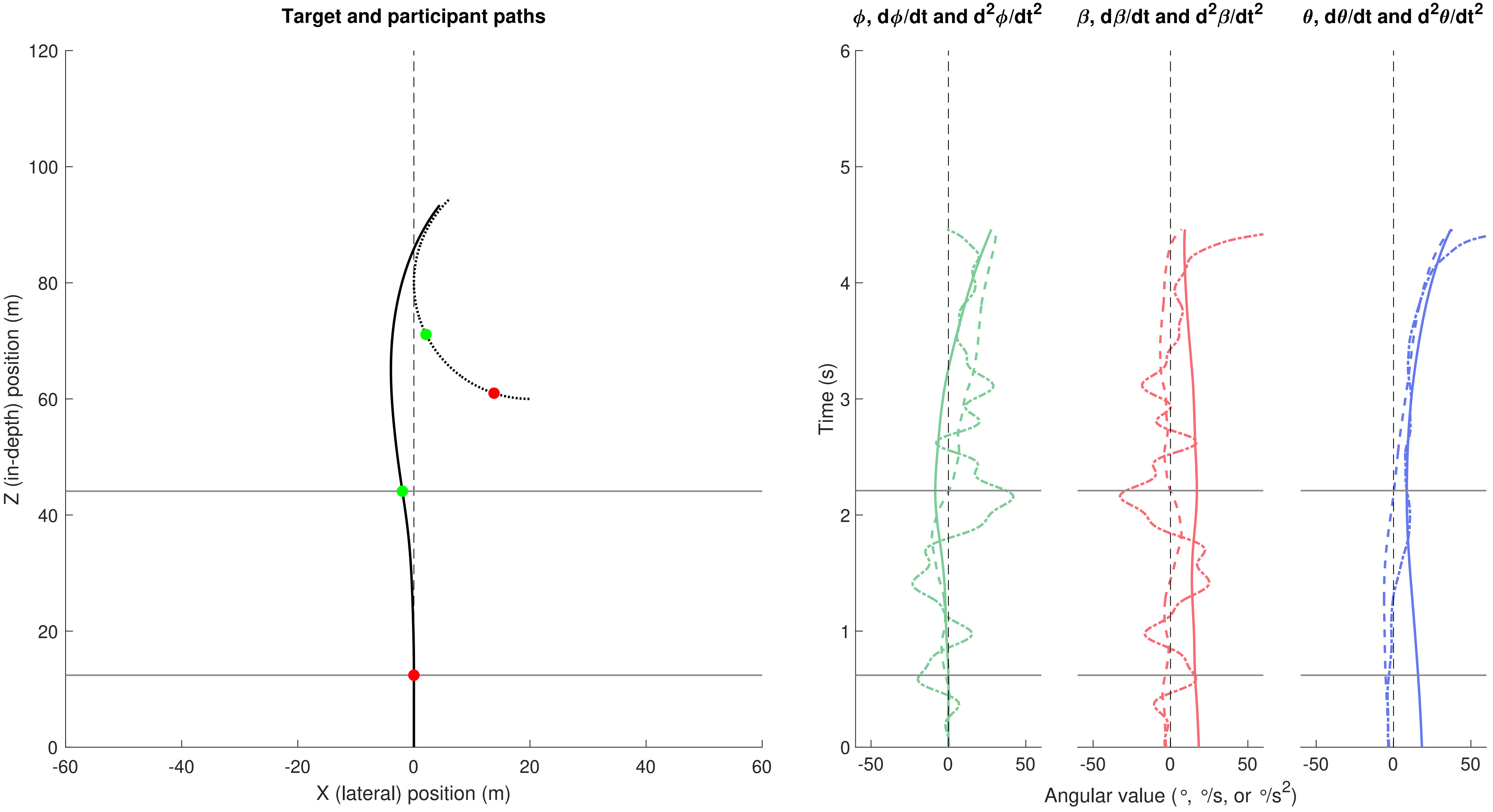

P13/B1
S20/R40-OUT

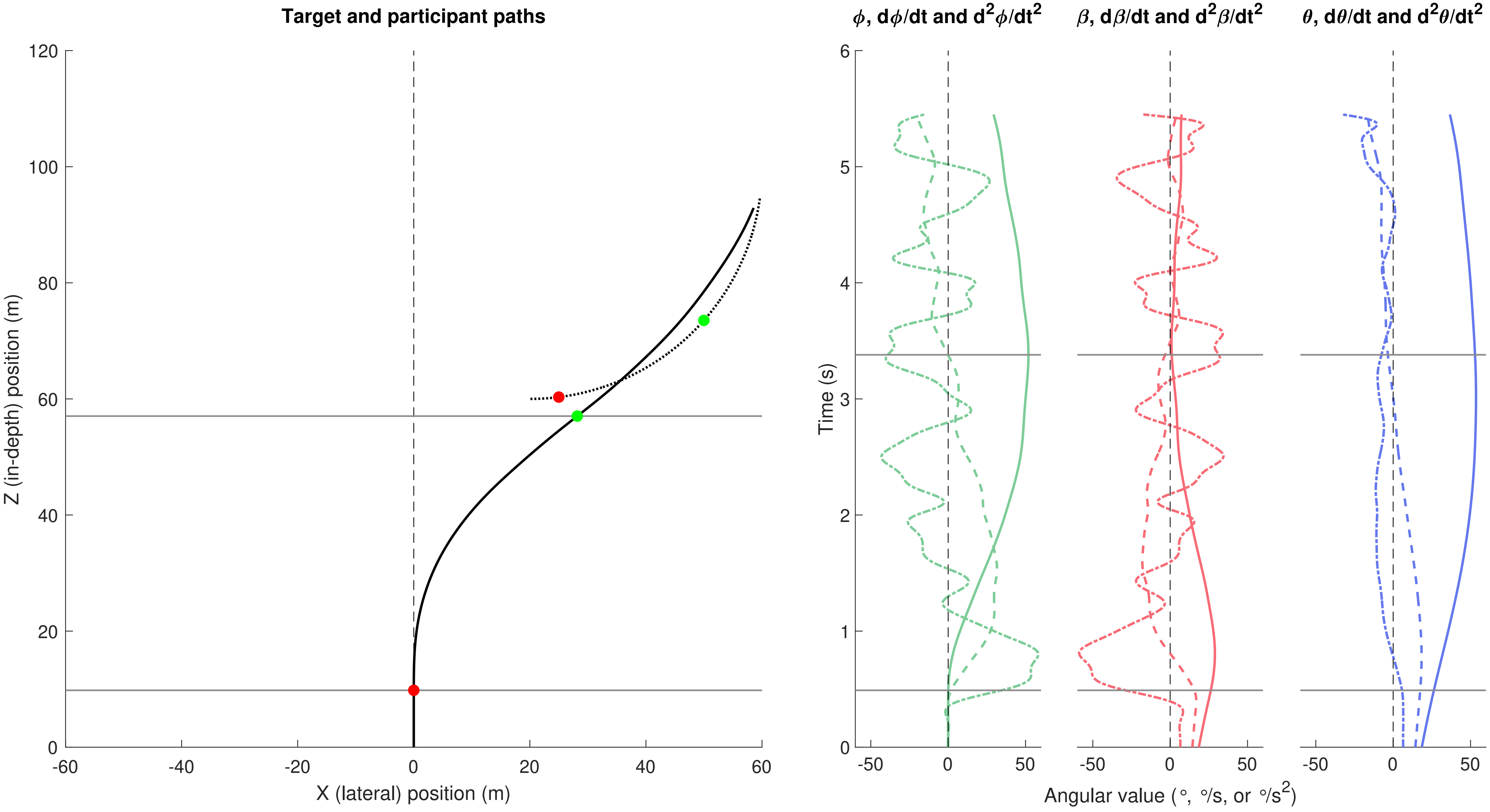

P13/B1
S20/R40-IN

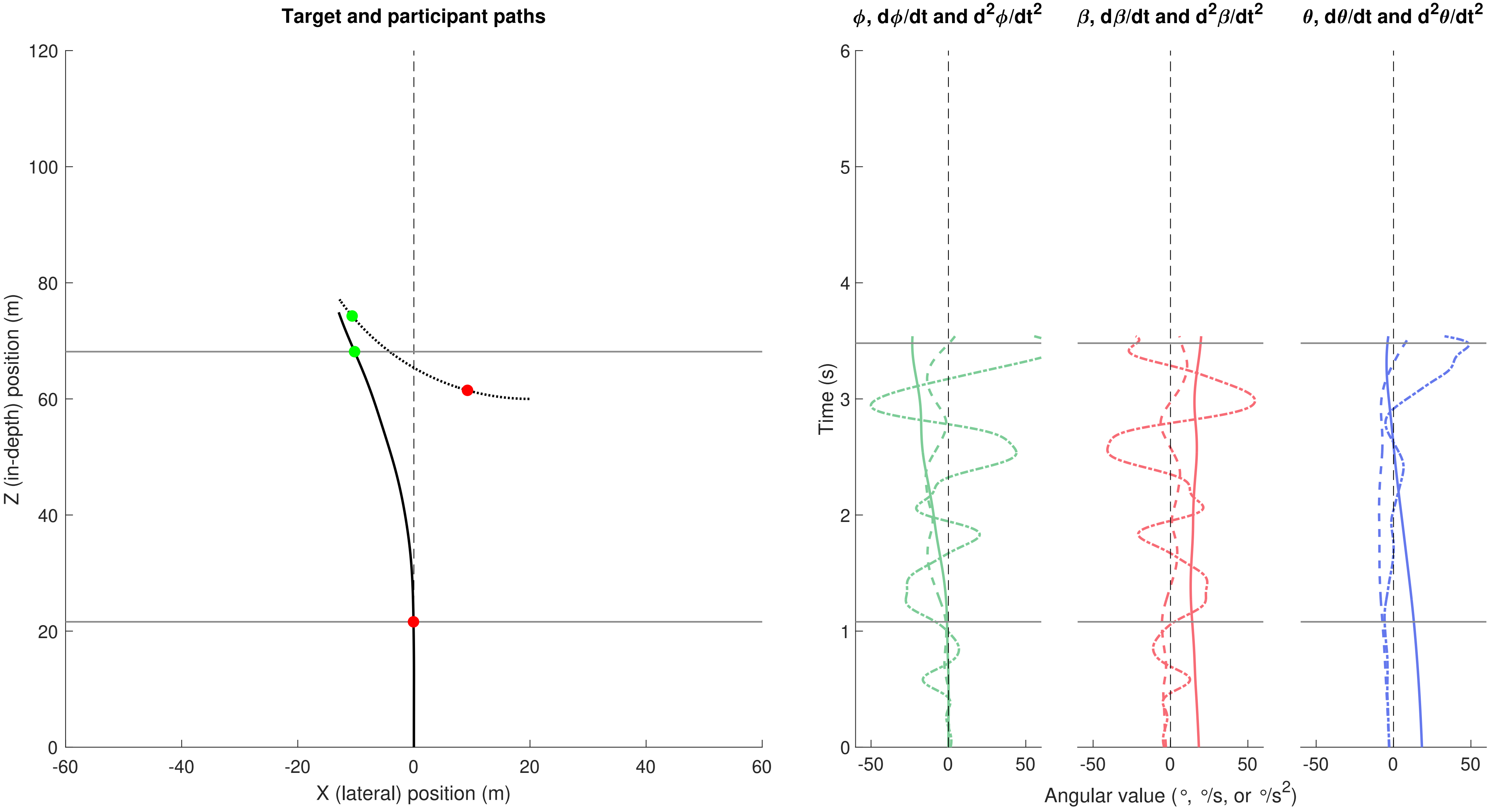

P13/B2
S20/R20-IN

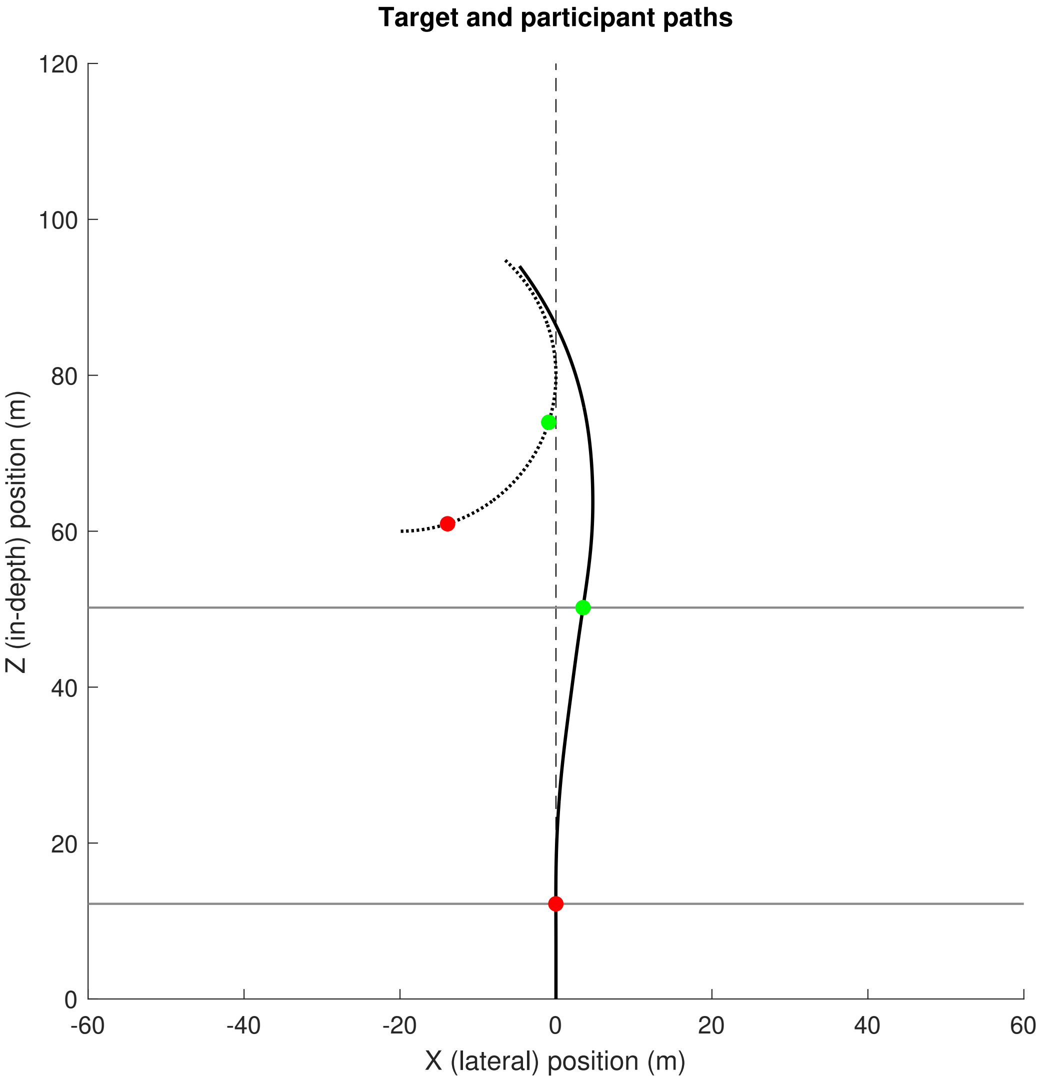

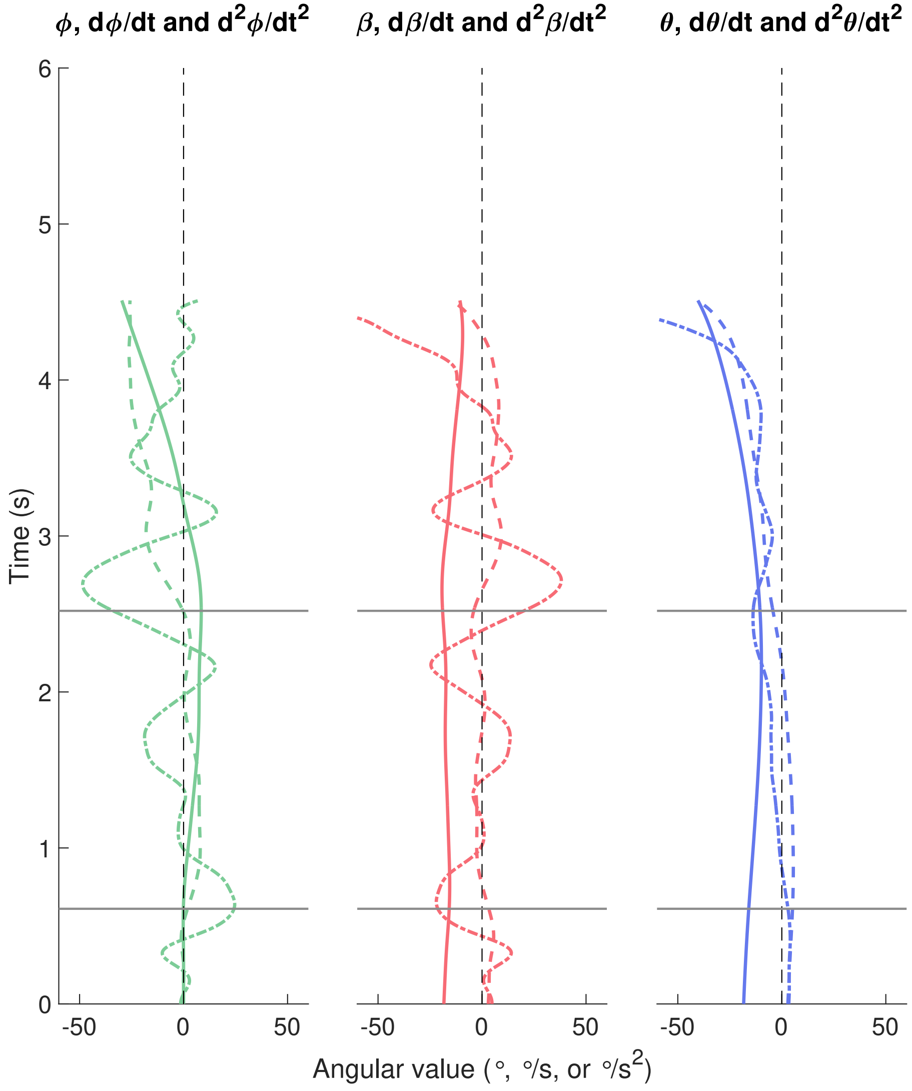

P13/B2
S20/R20-OUT

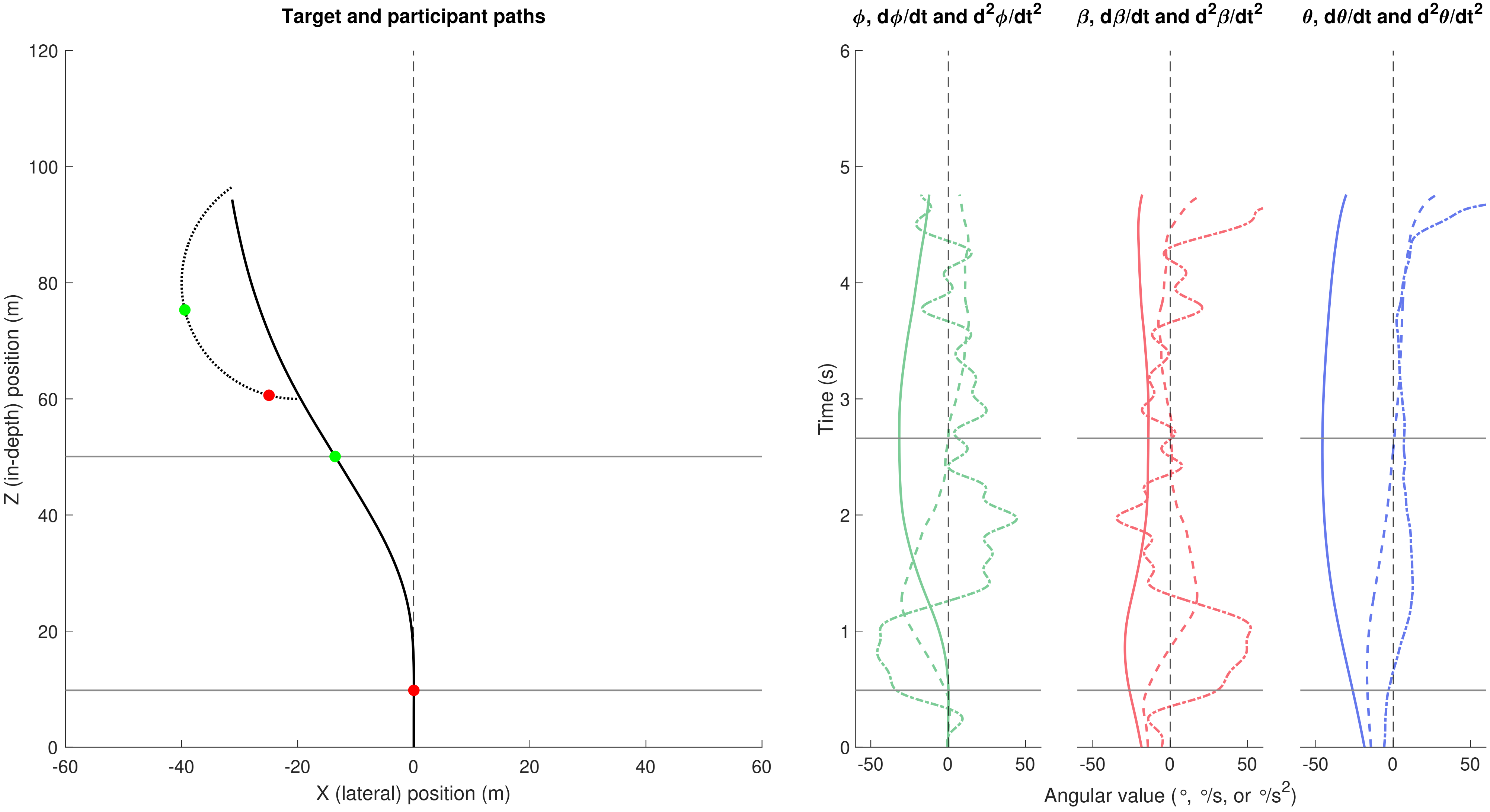

P13/B2
S20/R40-IN

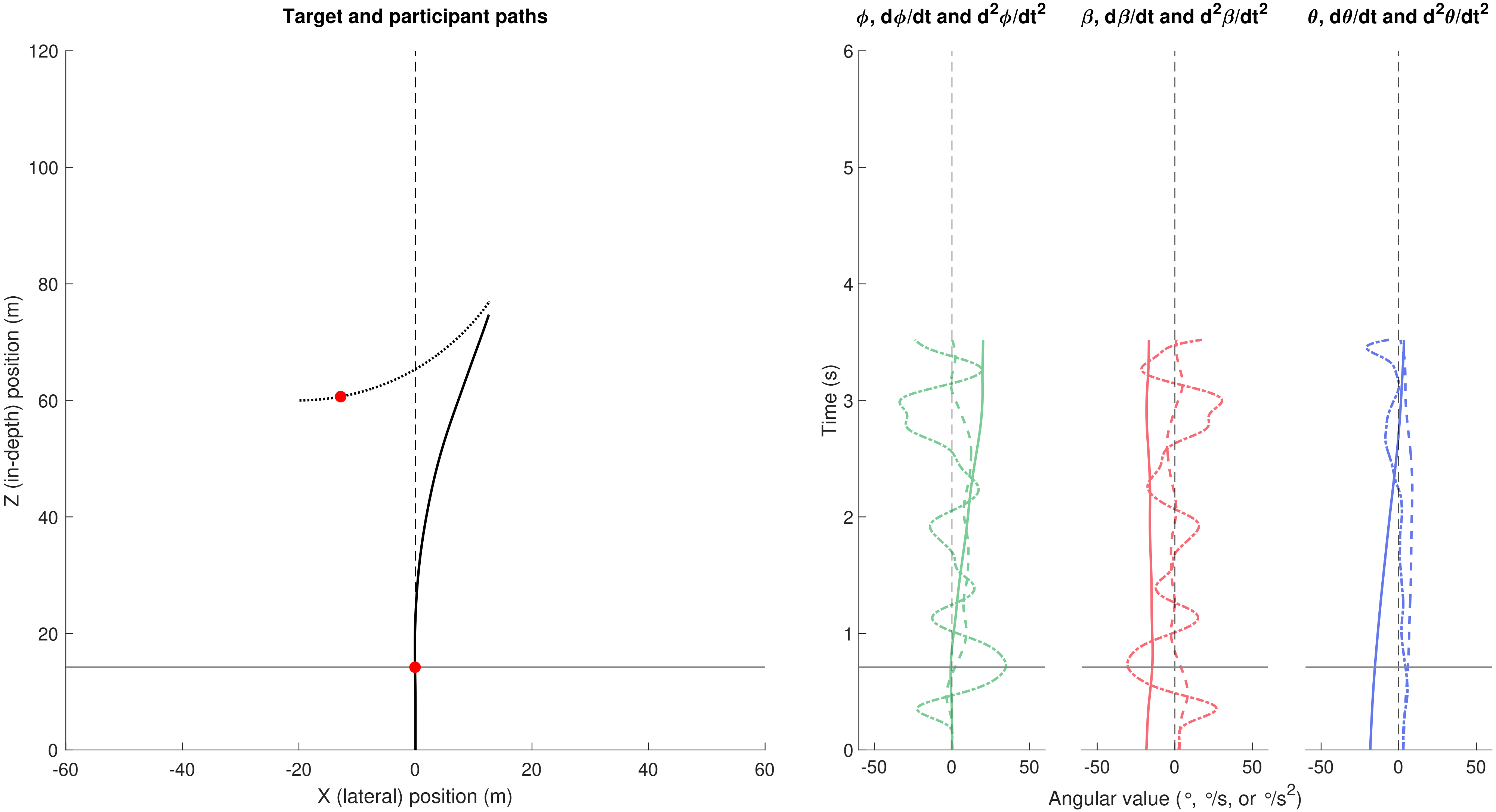

P13/B2
S20/R40-OUT

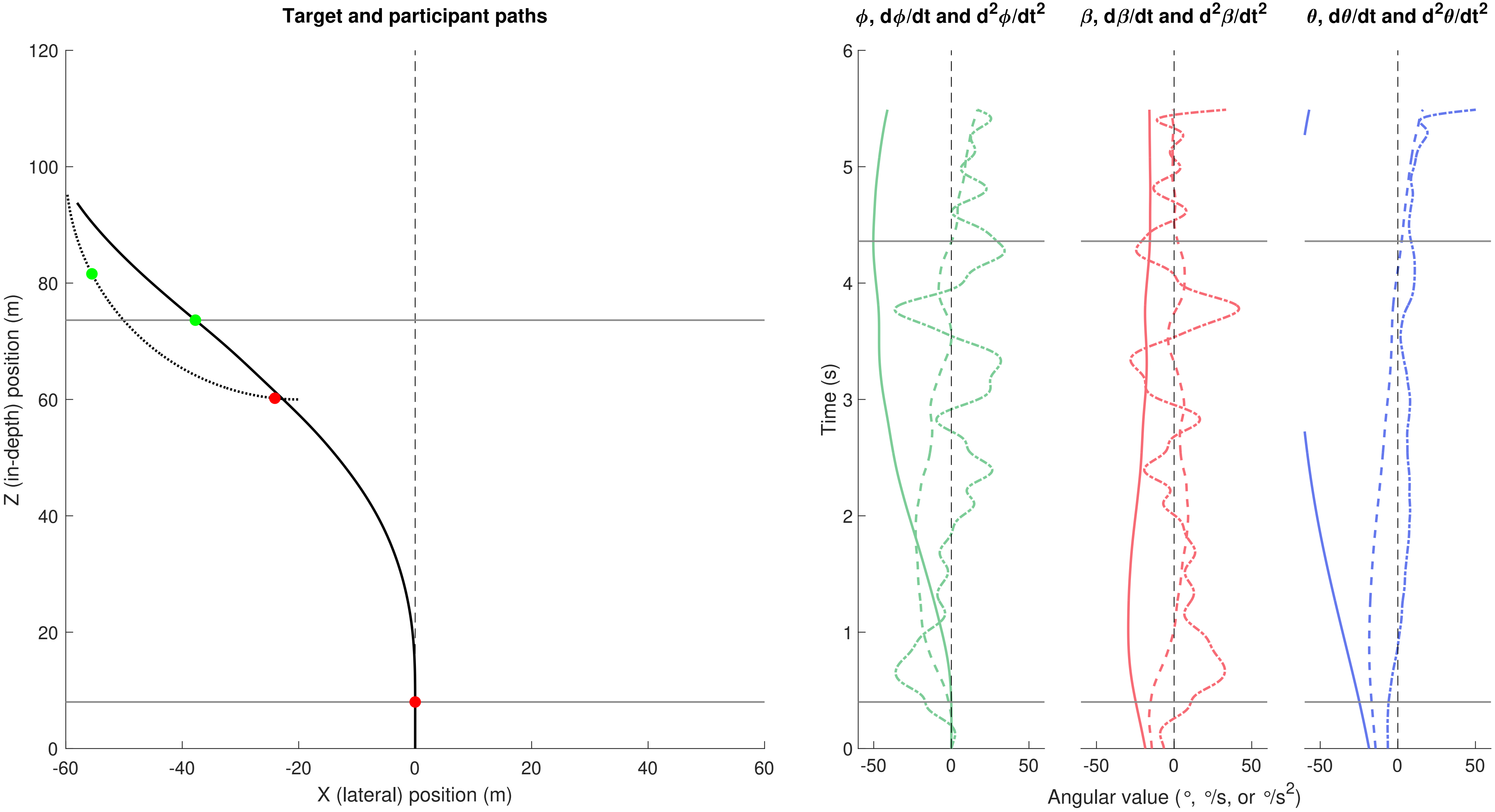

P13/B2
S10/R20-IN

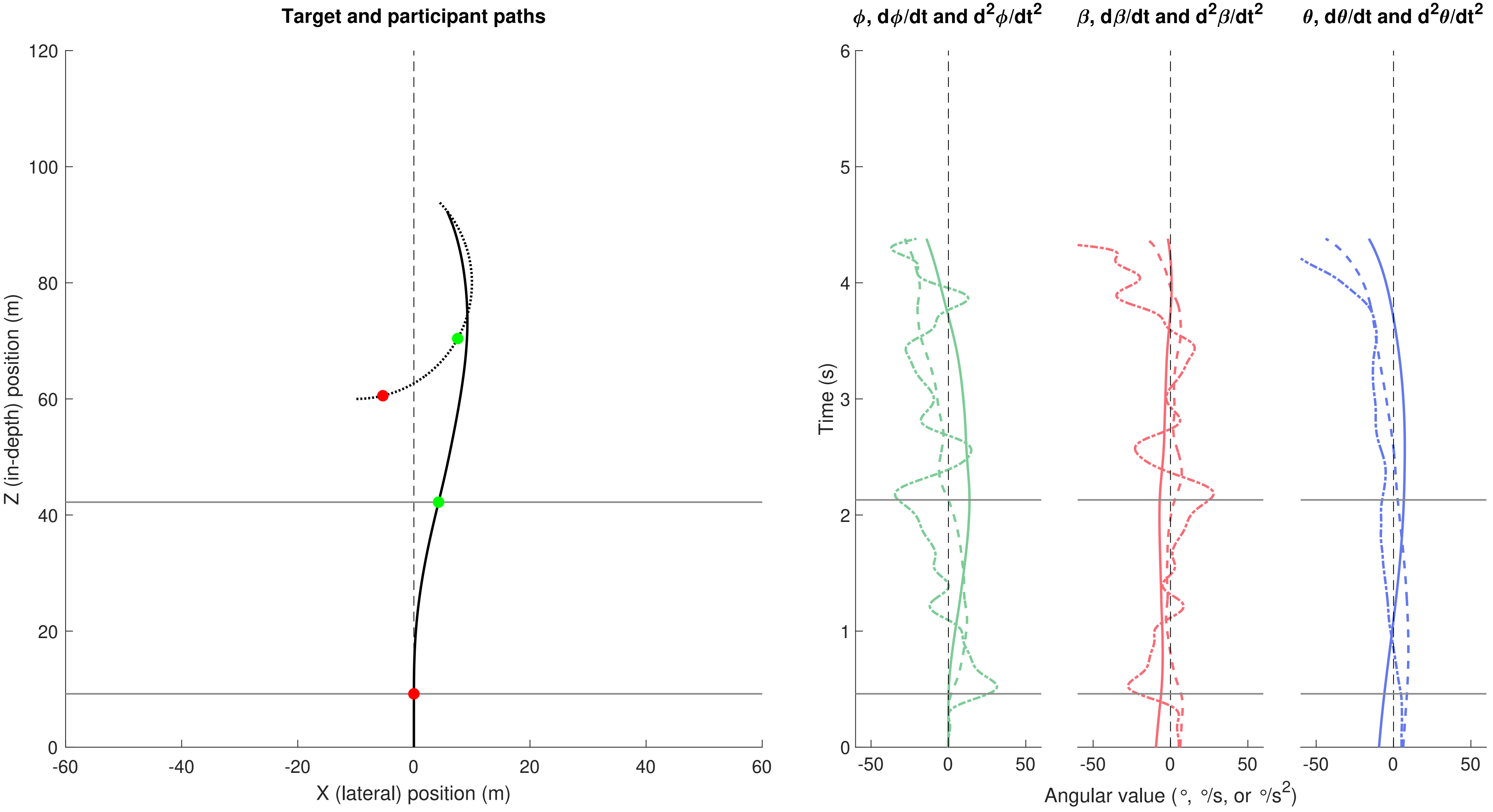

P13/B2
S10/R20-OUT

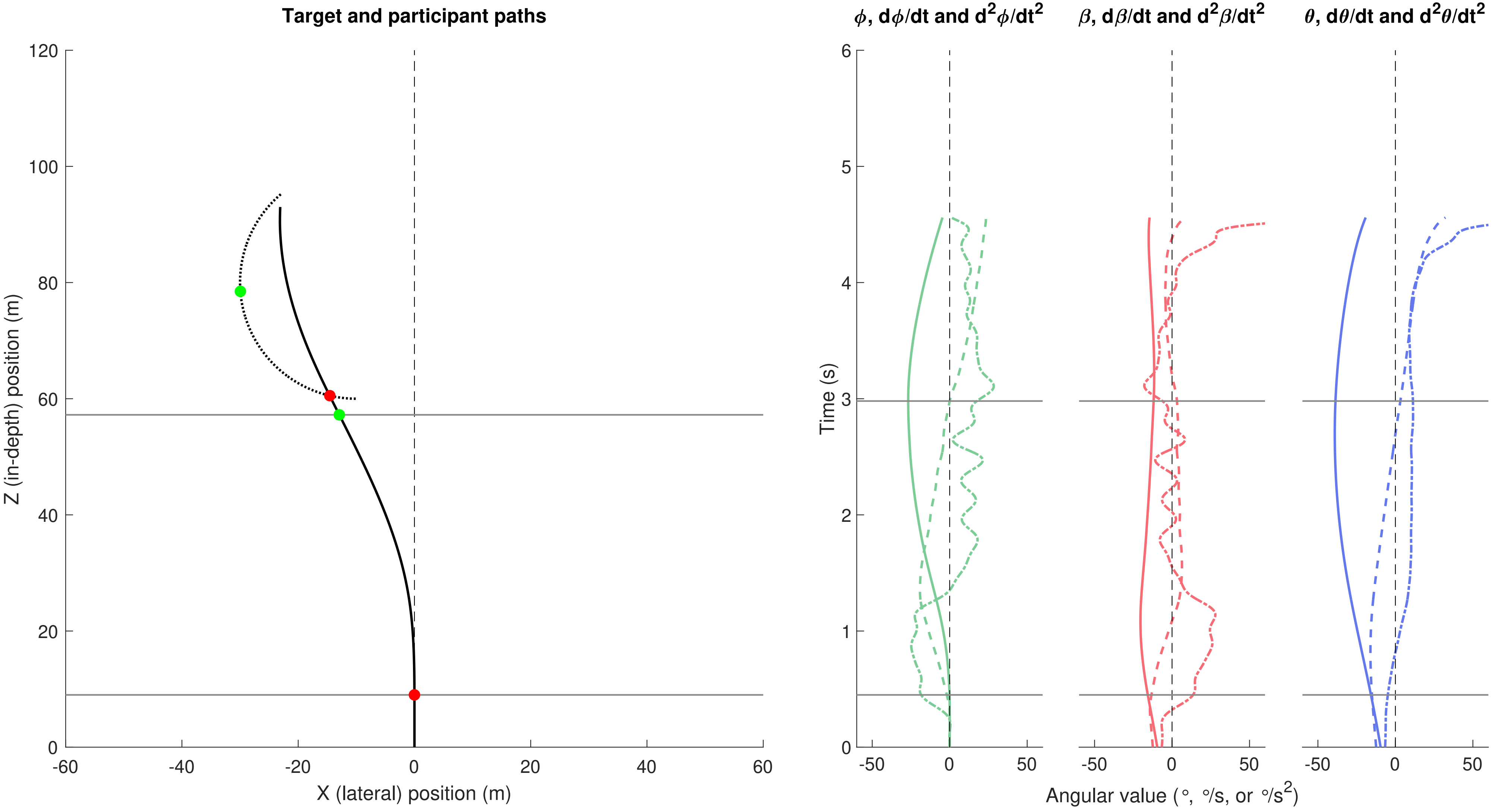

P13/B2
S10/R40-IN

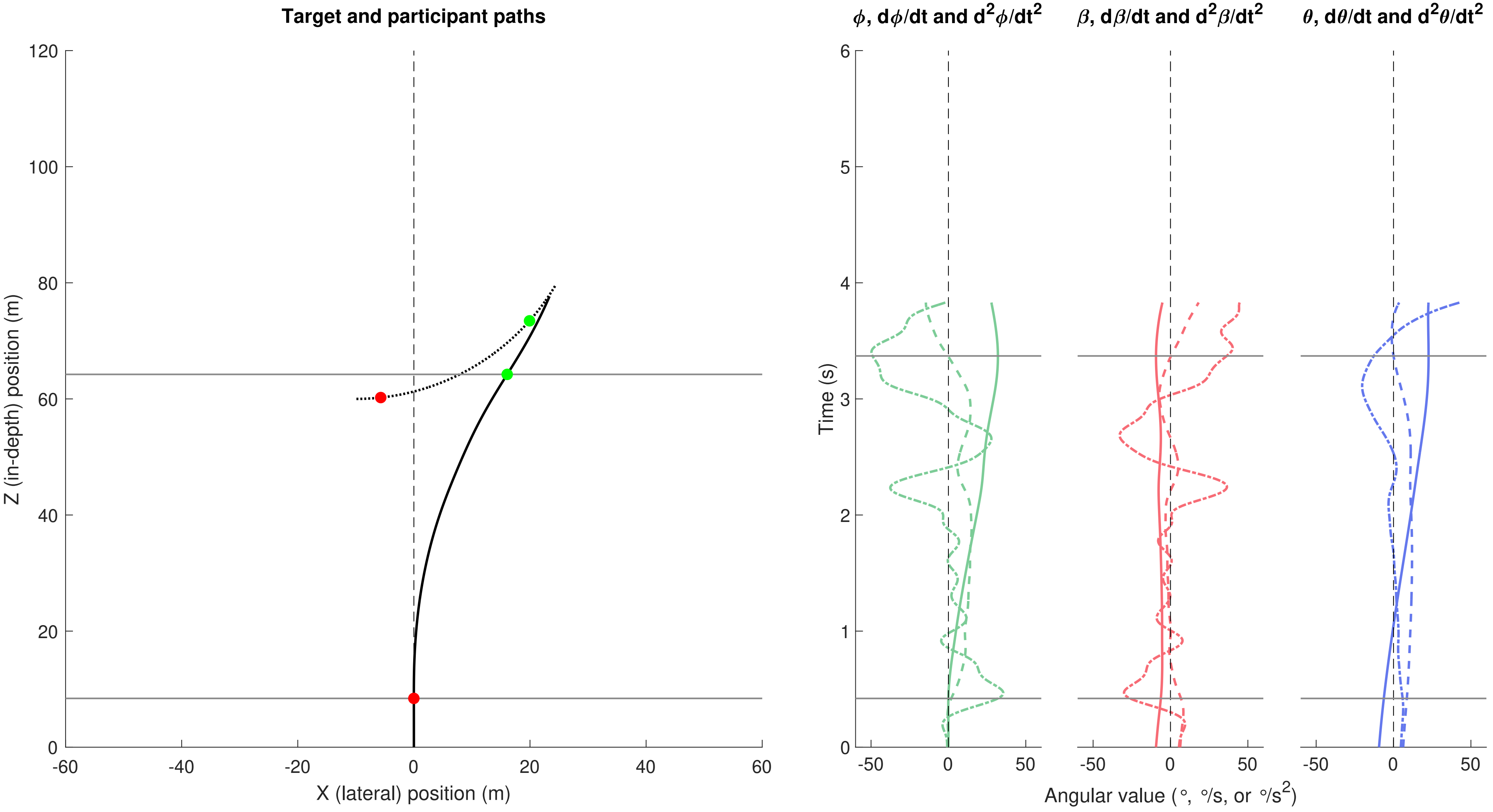

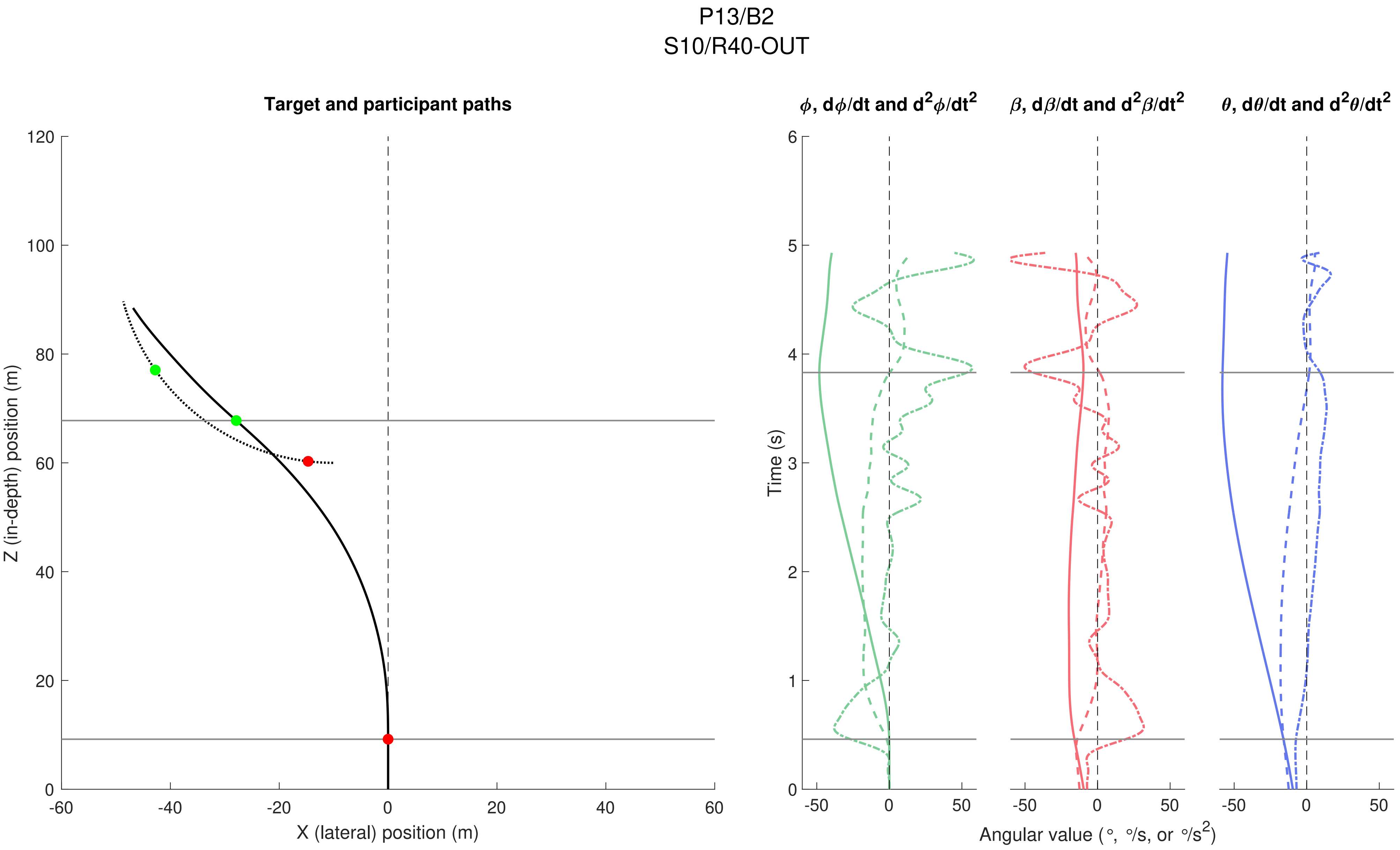
P13/B2
S10/R40-OUT
Target and participant paths
Z (in-depth) position (m)
X (lateral) position (m)
120
100
80
60
40
20
0
-60
-40
-20
0
20
40
60
ϕ, dϕ/dt and d$^2\phi$/dt^2
β, dβ/dt and d$^2\beta$/dt^2
θ, dθ/dt and d$^2\theta$/dt^2
Time (s)
6
5
4
3
2
1
0
-50
0
50
Angular value (°, °/s, or °/s^2)

P13/B2
S0/R20-OUT

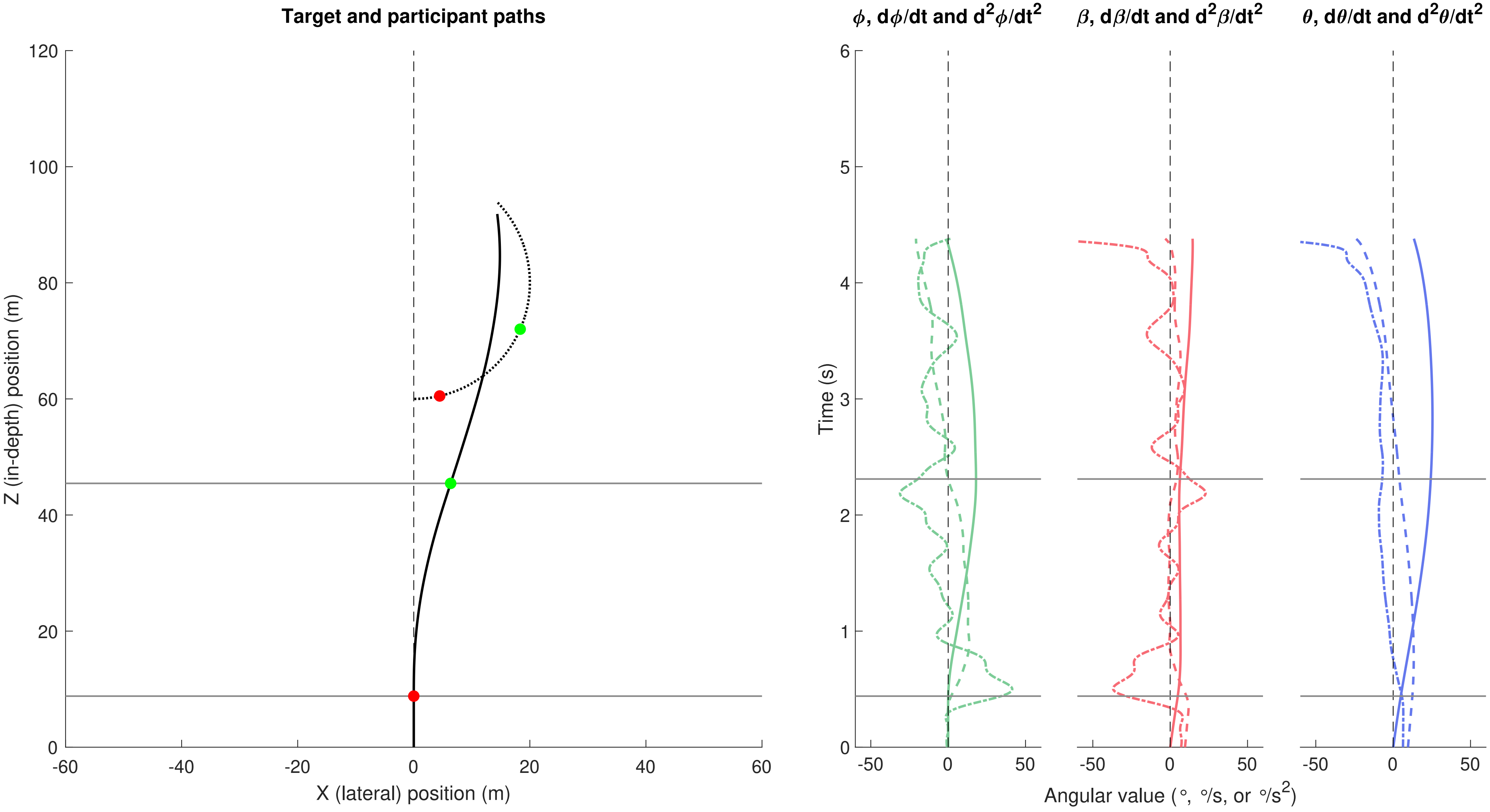

P13/B2
S0/R20-OUT

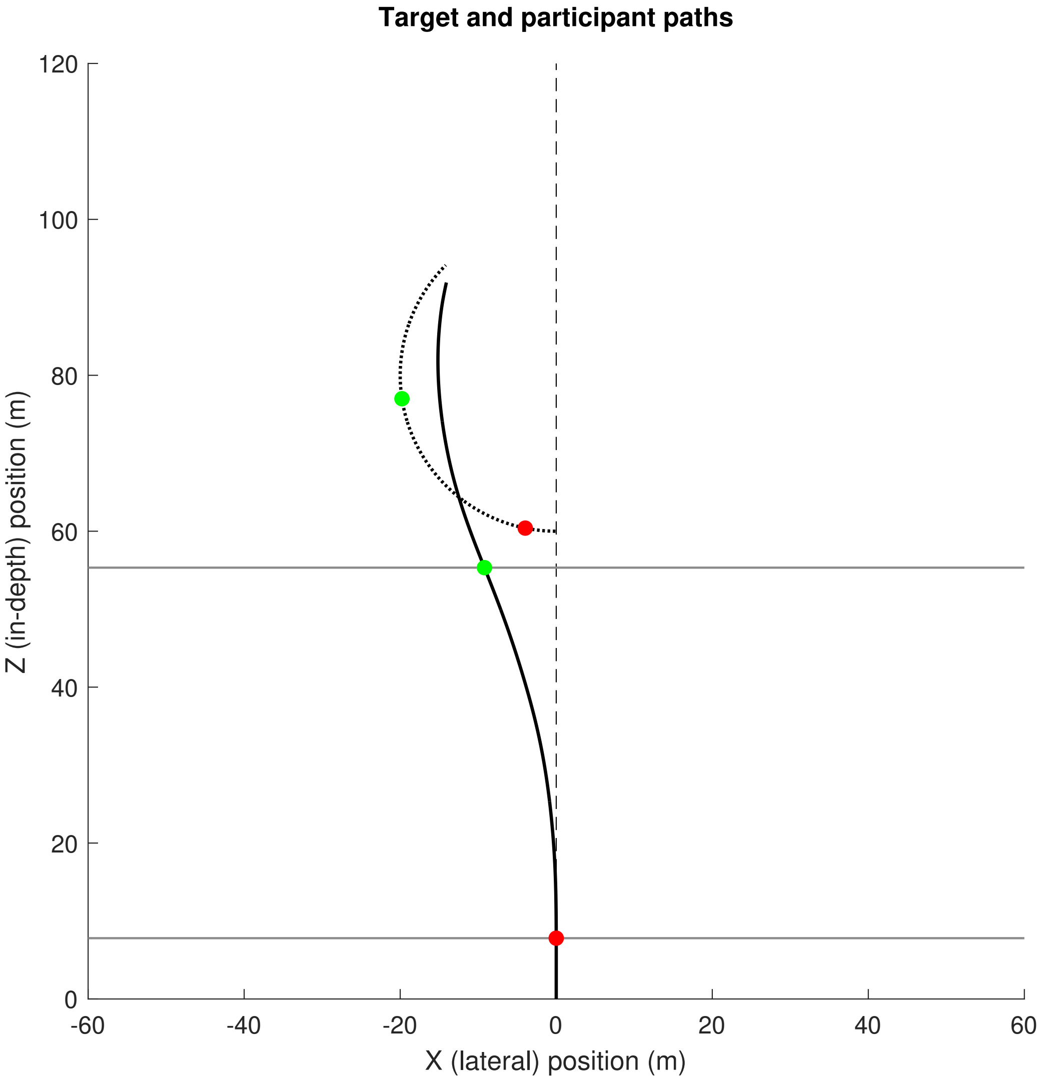

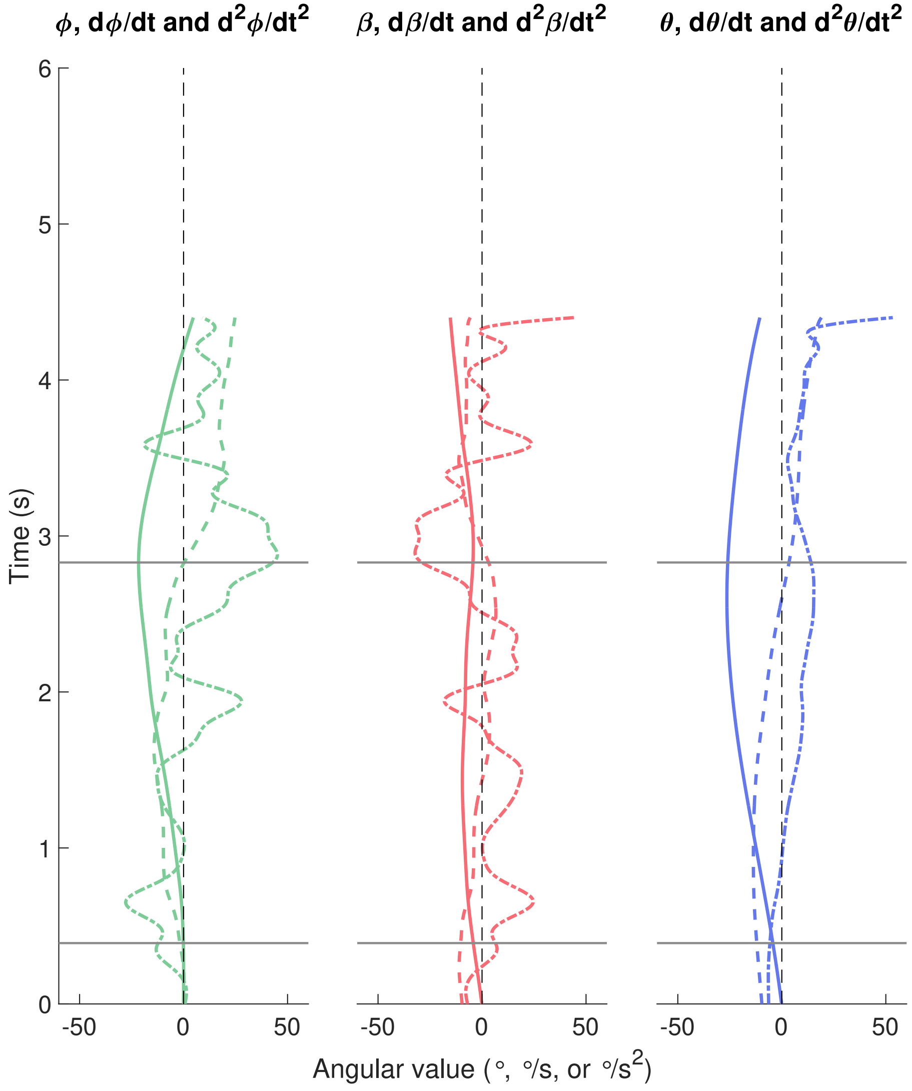

P13/B2
S0/R40-OUT

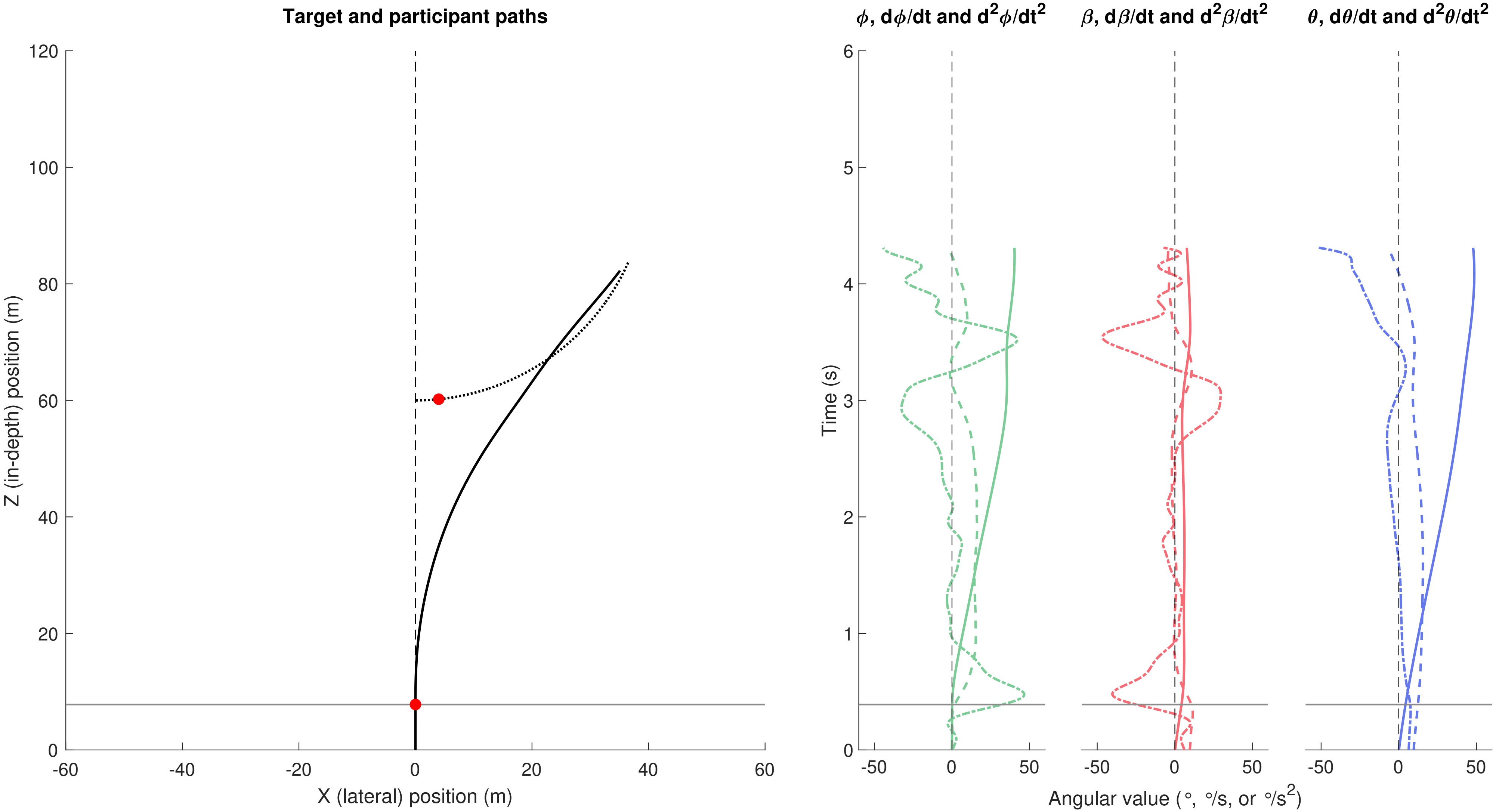

P13/B2
S0/R40-OUT

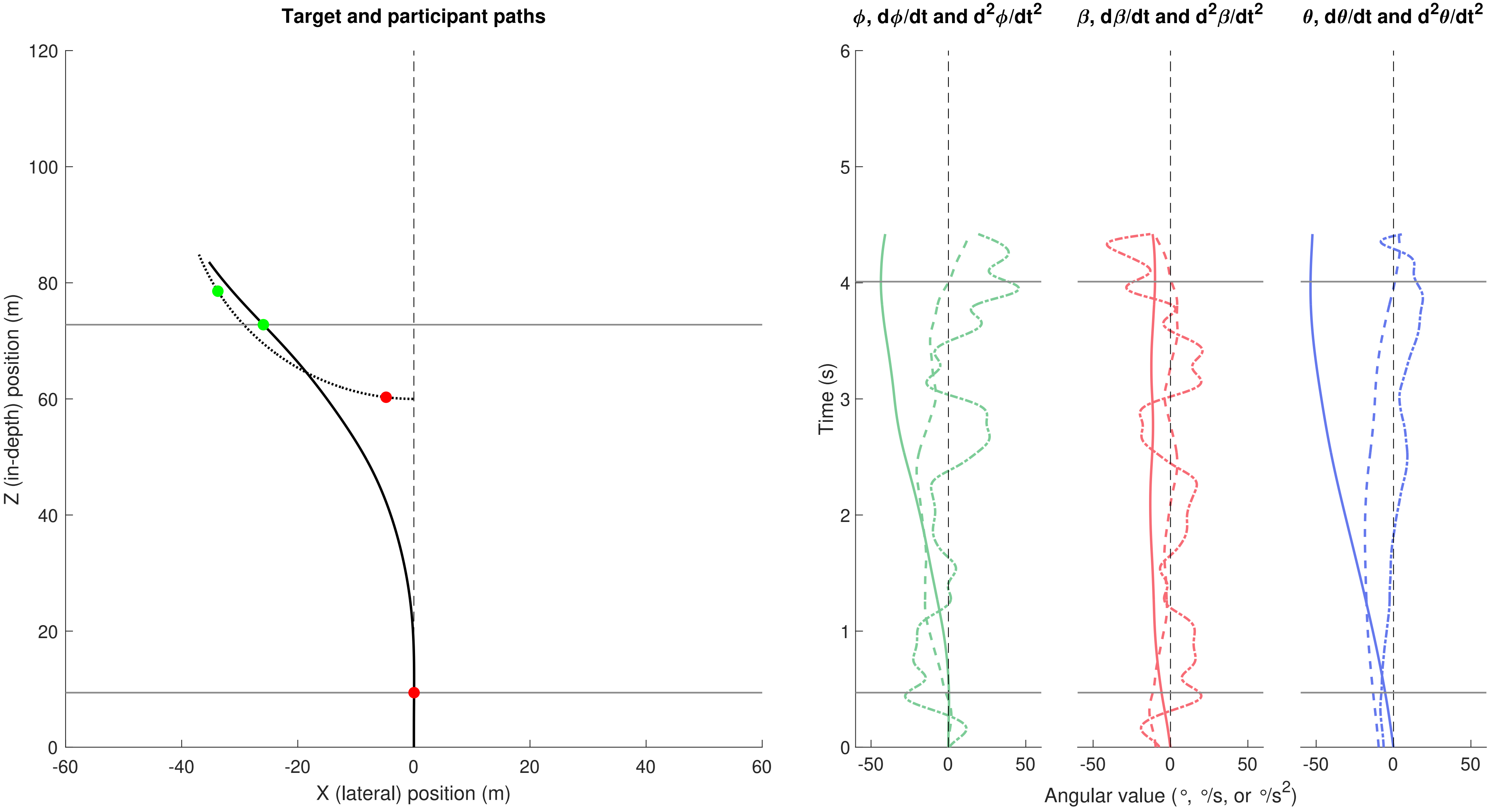

P13/B2
S10/R20-OUT

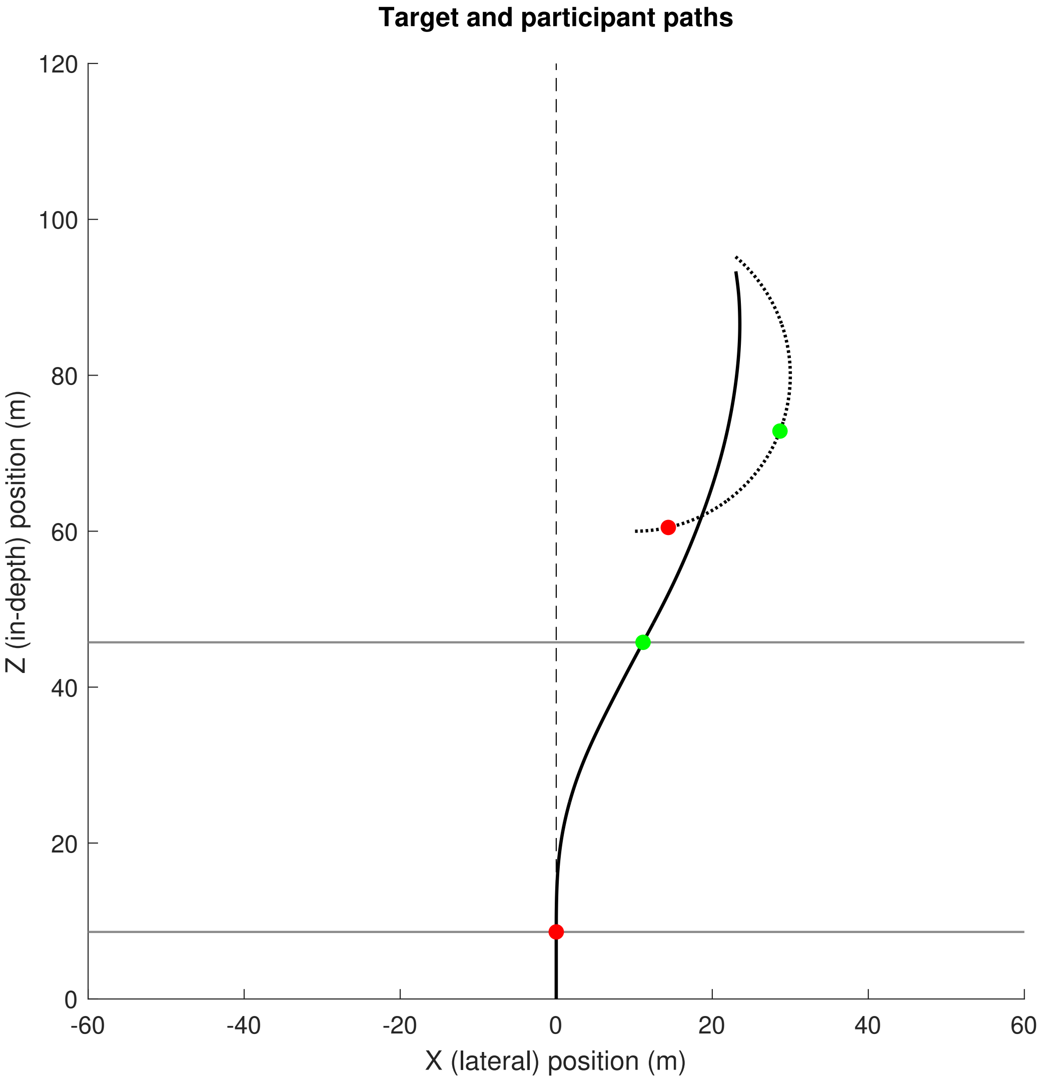

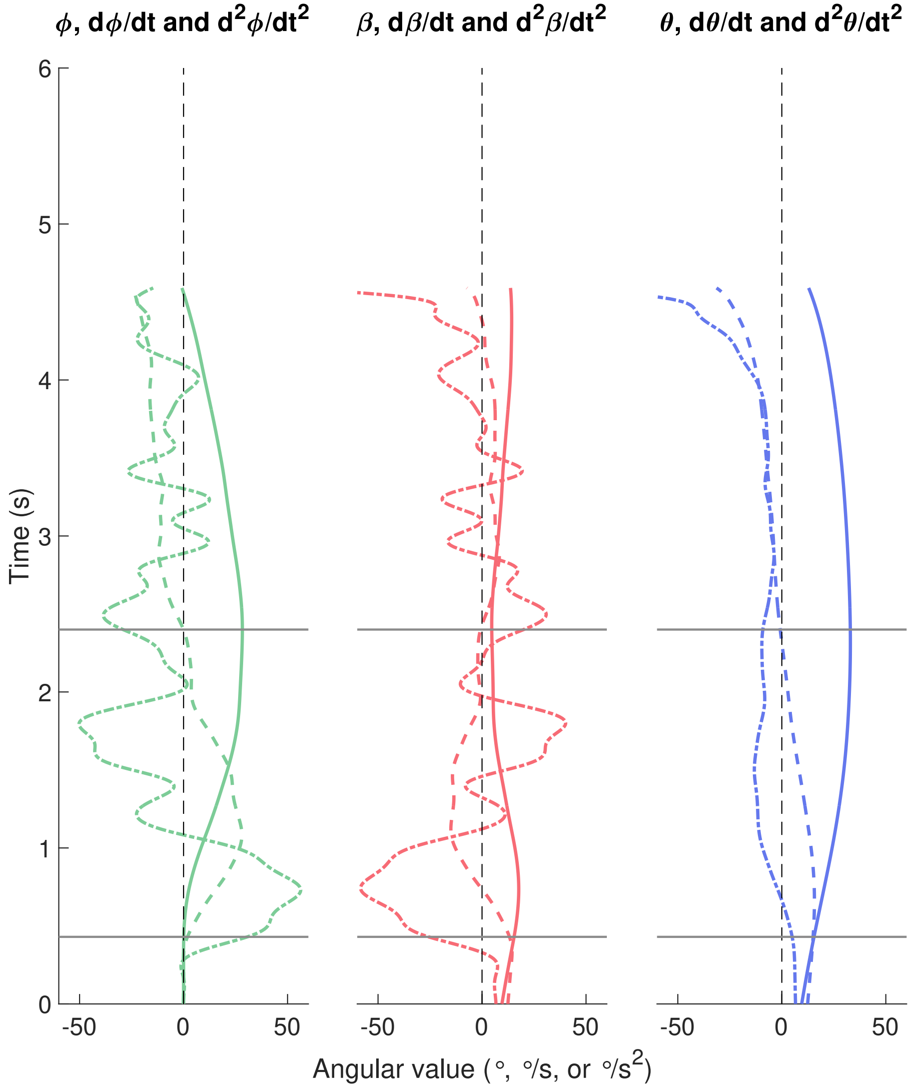

P13/B2
S10/R20-IN

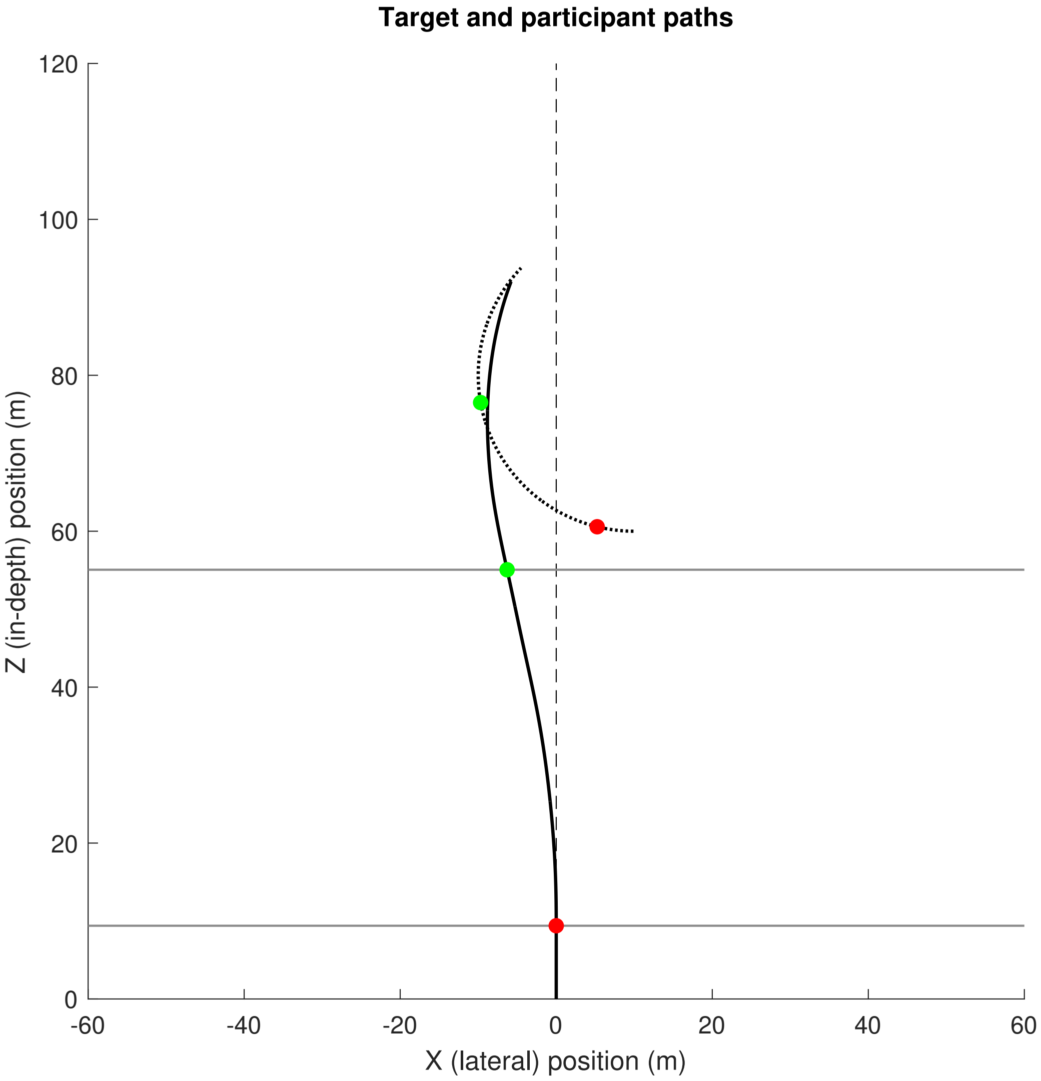

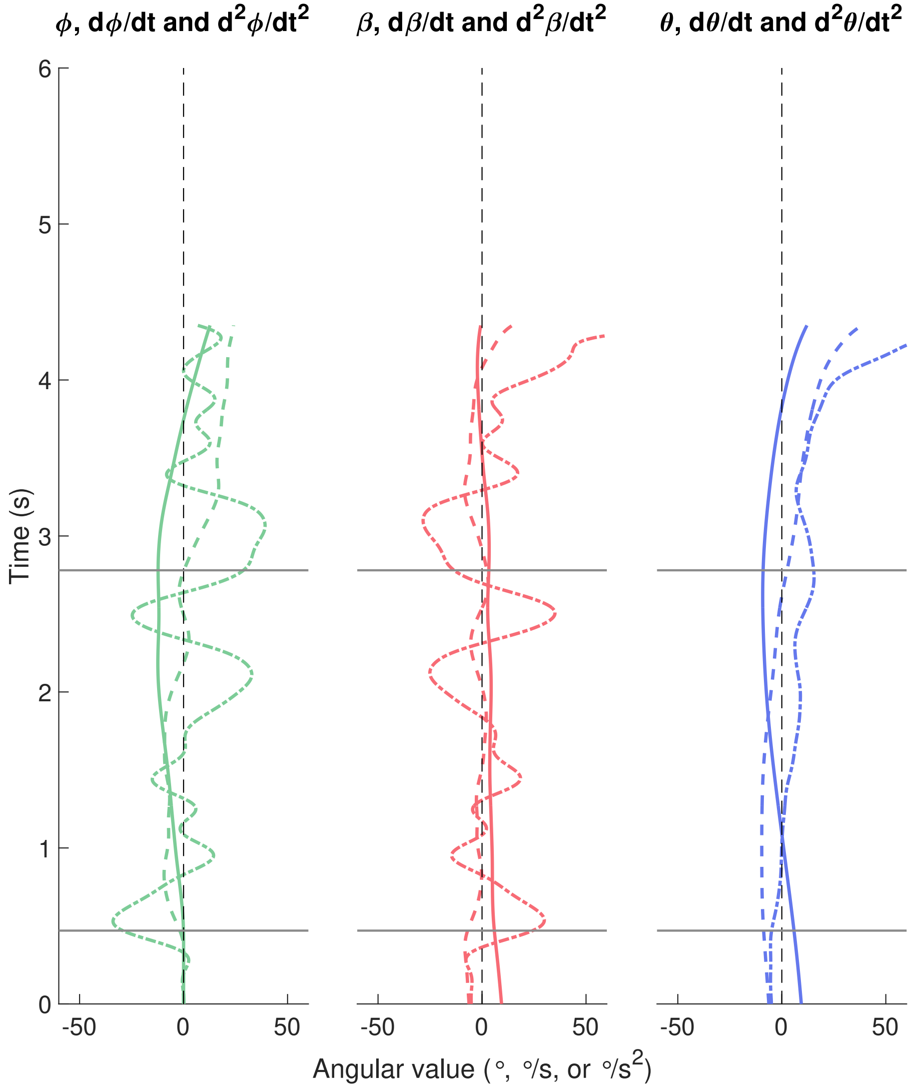

P13/B2
S10/R40-OUT

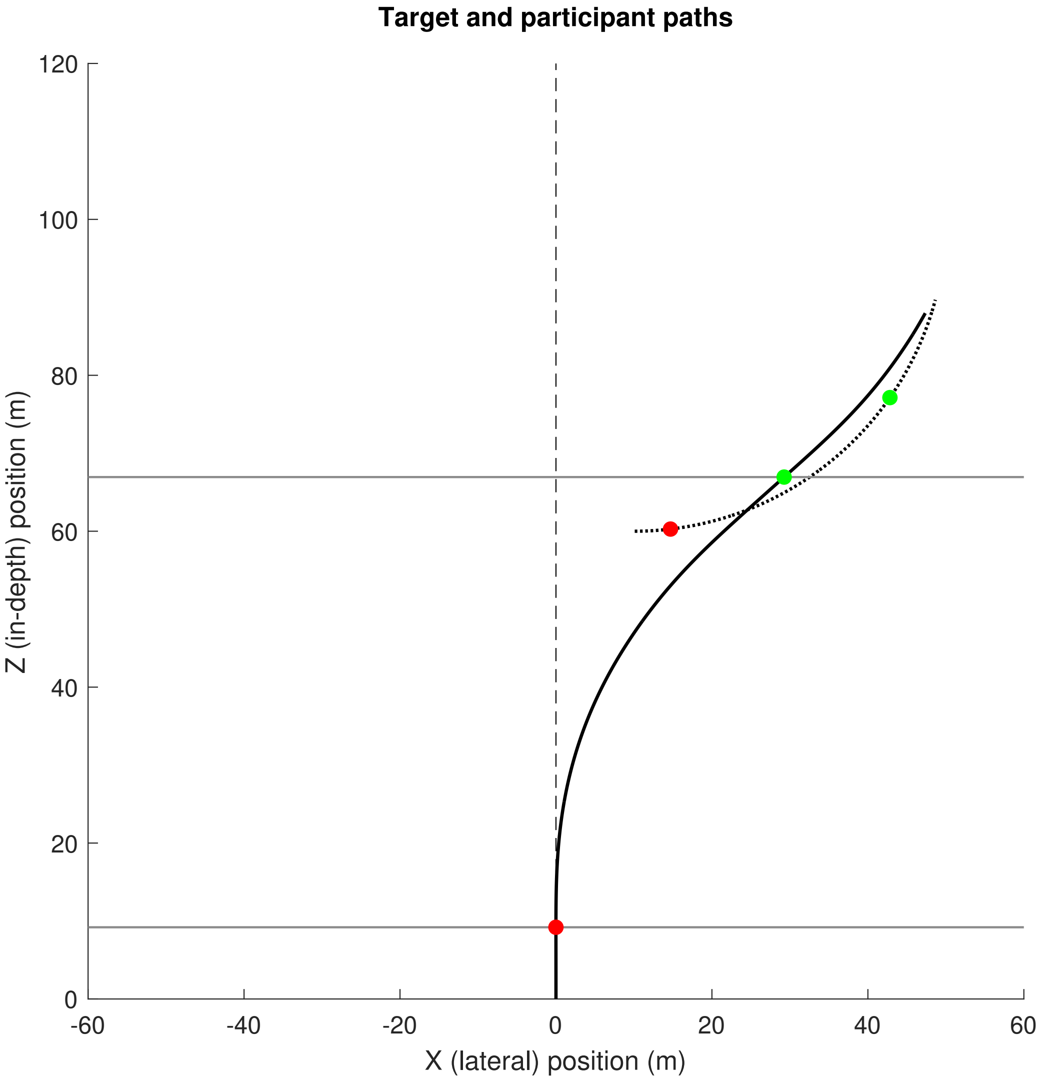

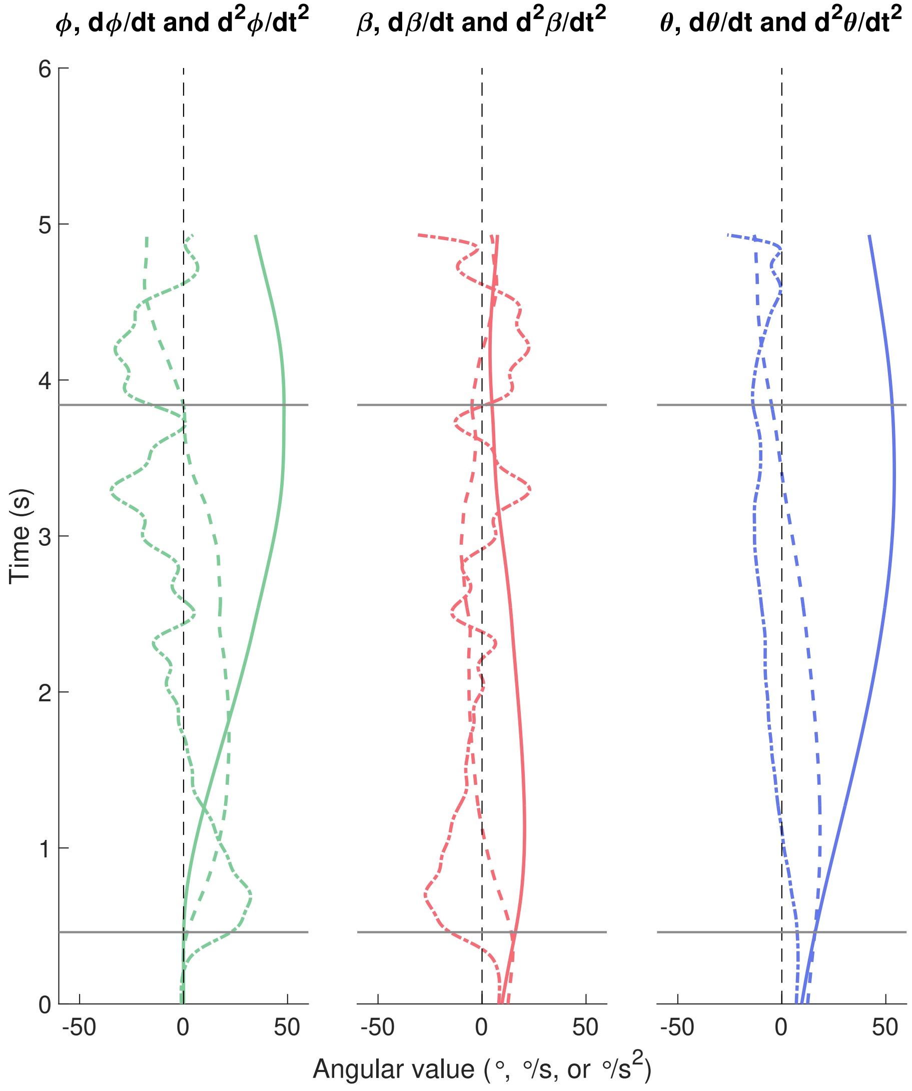

P13/B2
S10/R40-IN

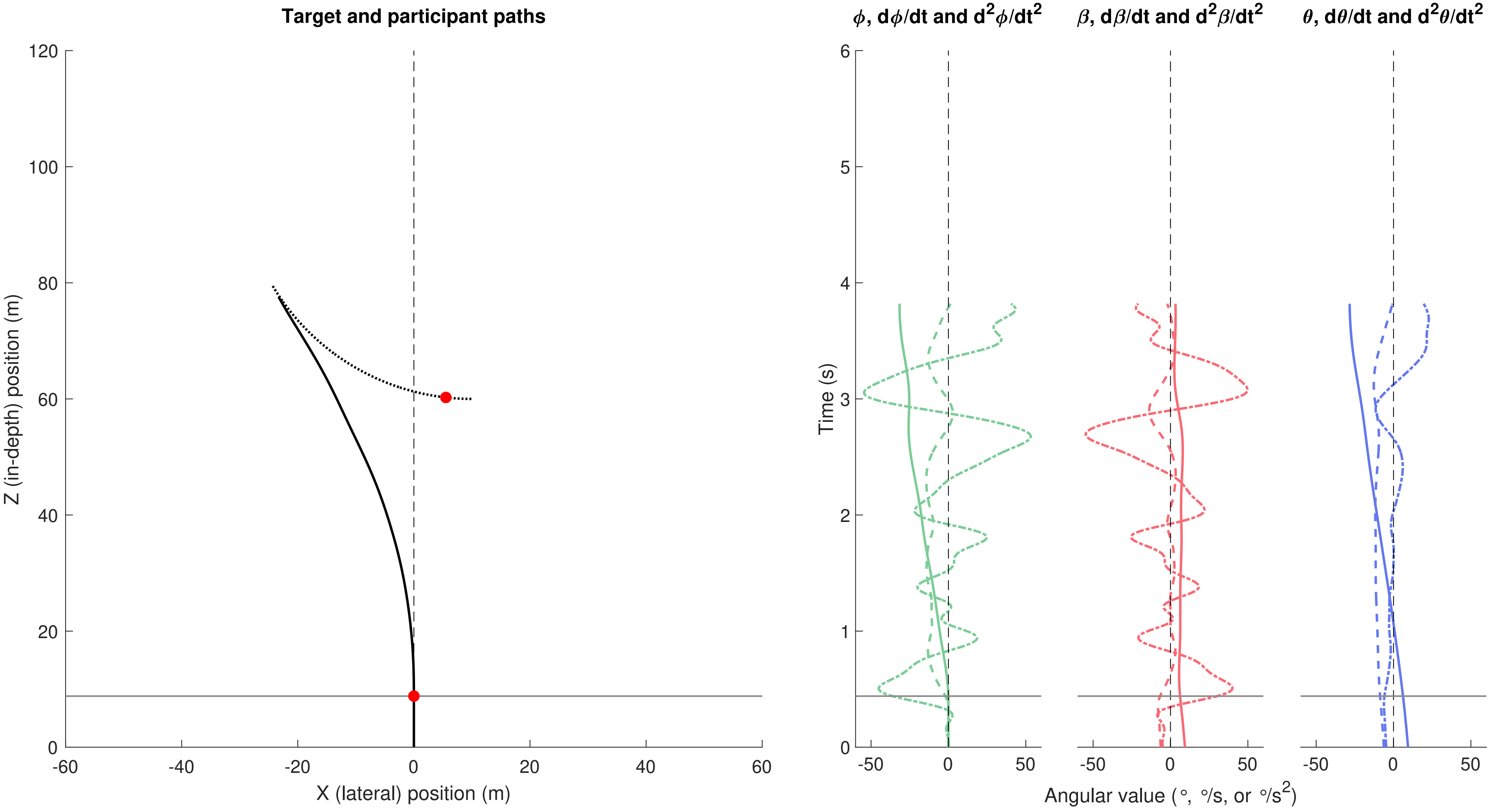

P13/B2
S20/R20-OUT

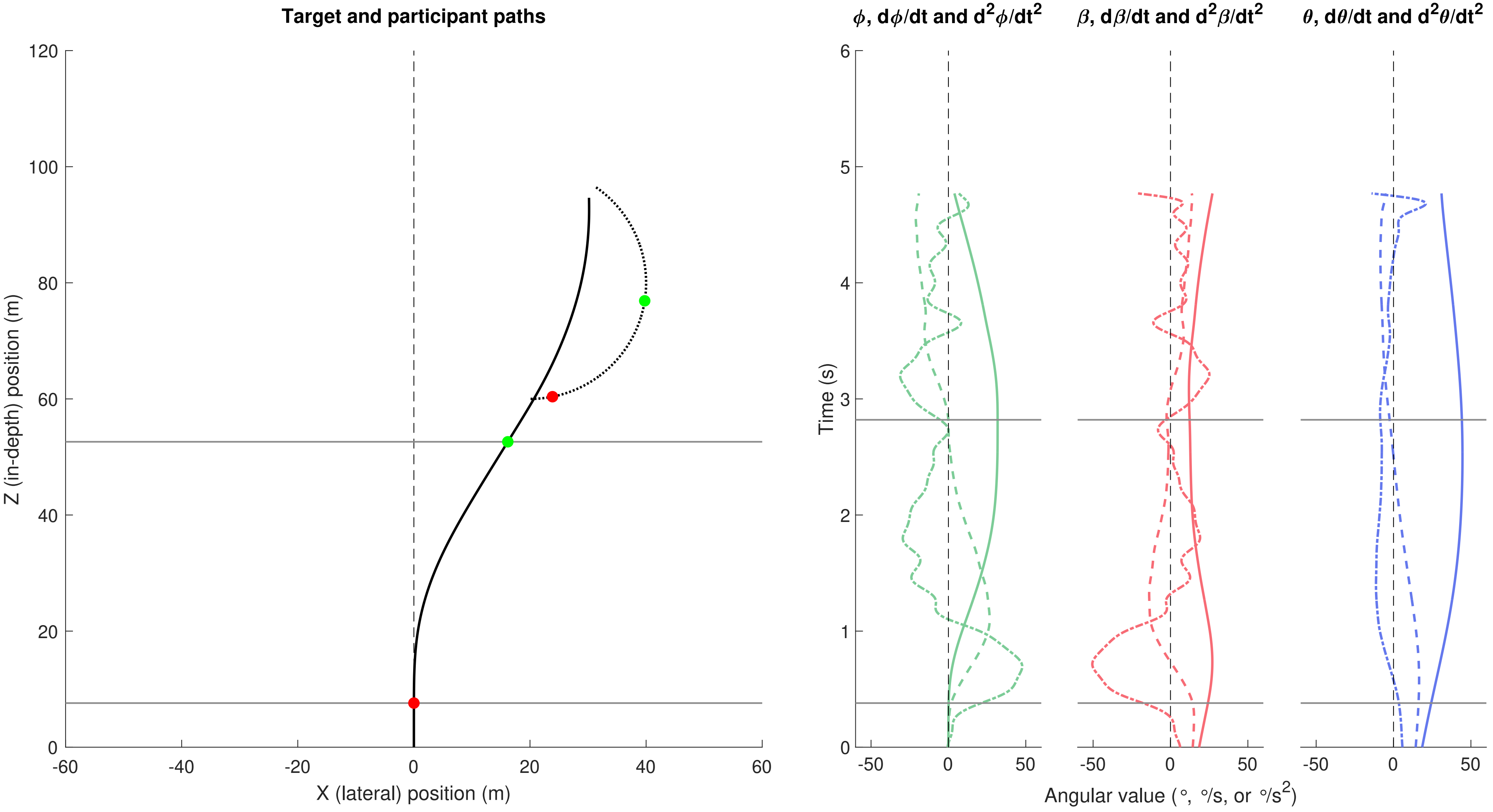

P13/B2
S20/R20-IN

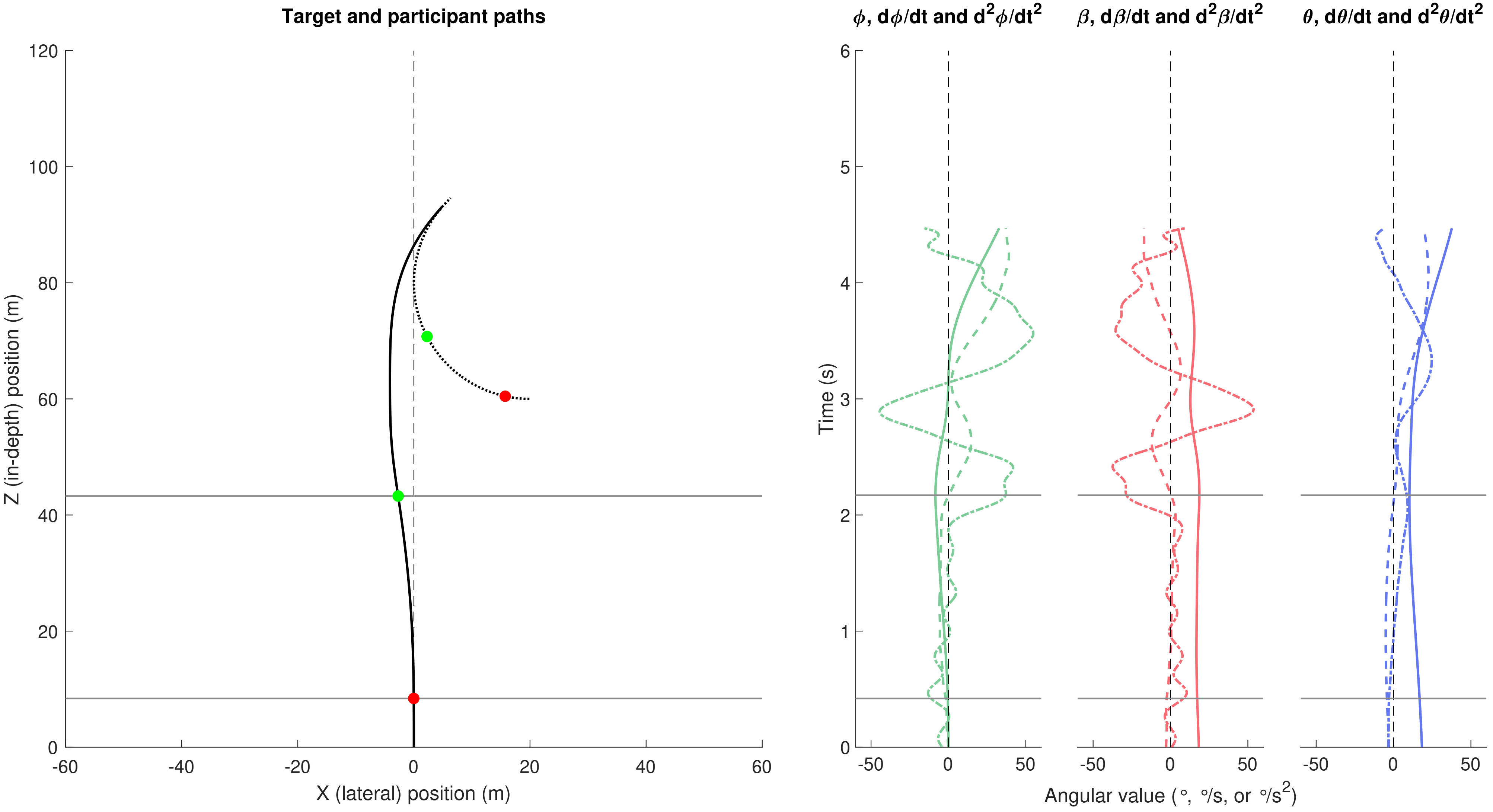

P13/B2
S20/R40-OUT

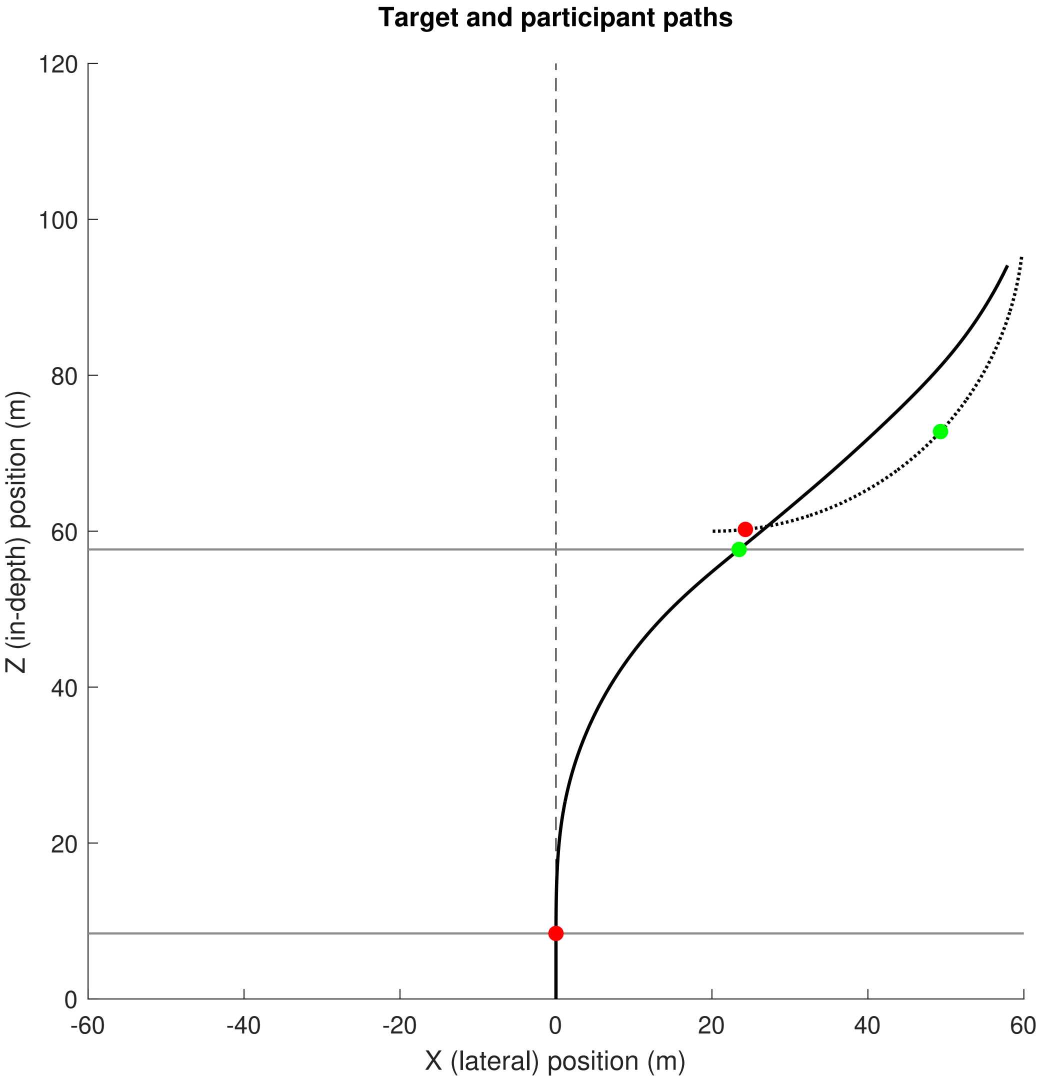

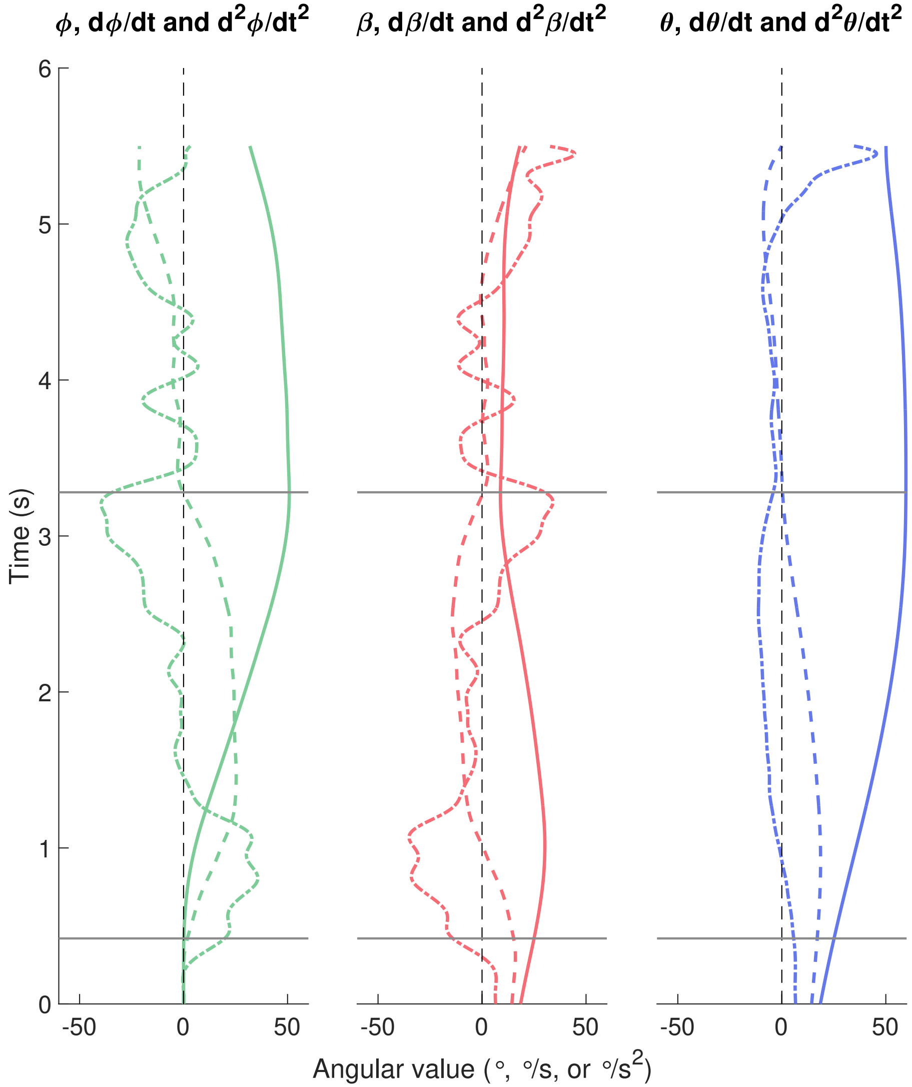

P13/B2
S20/R40-IN

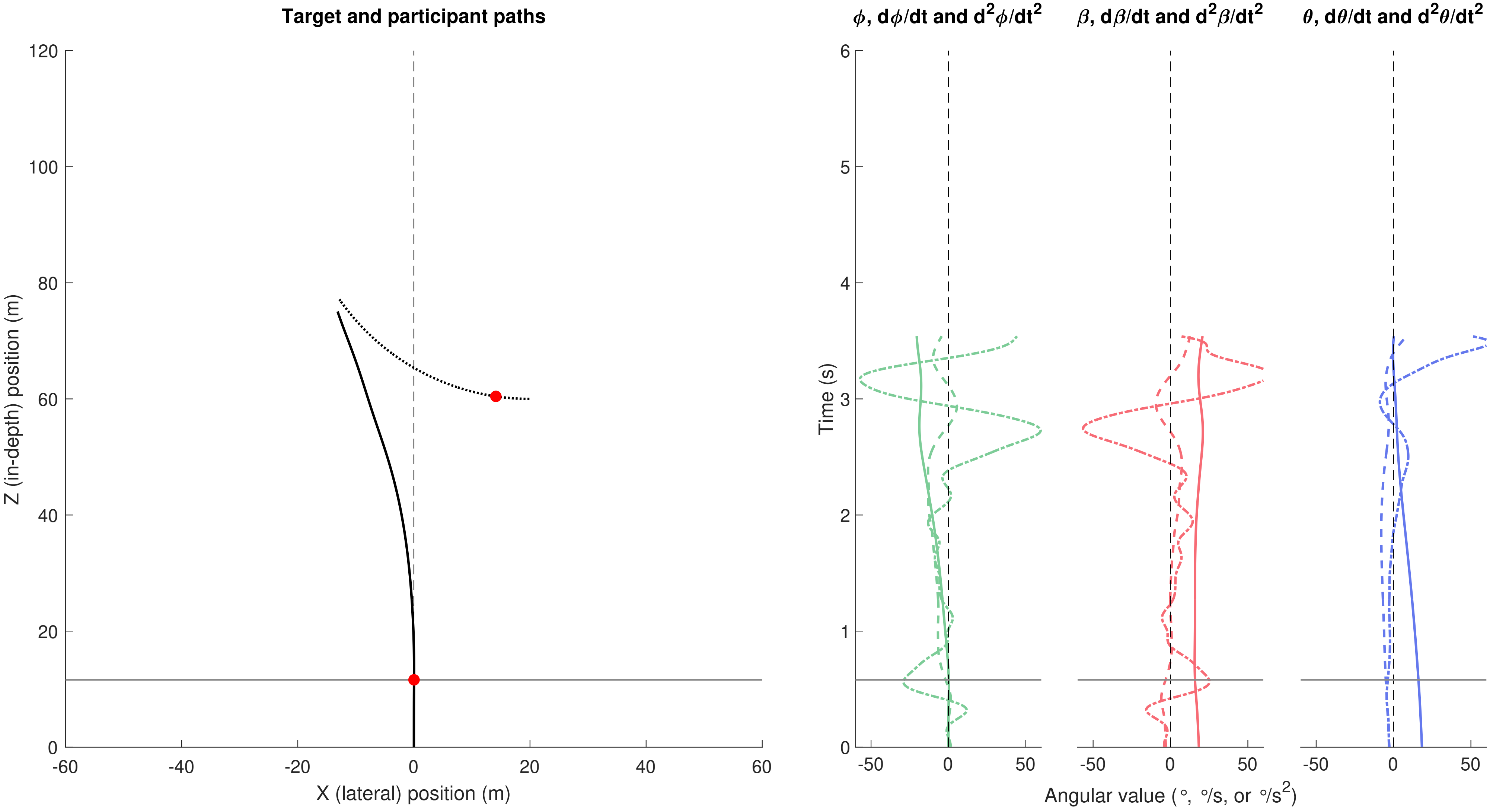

P13/B3
S20/R20-IN

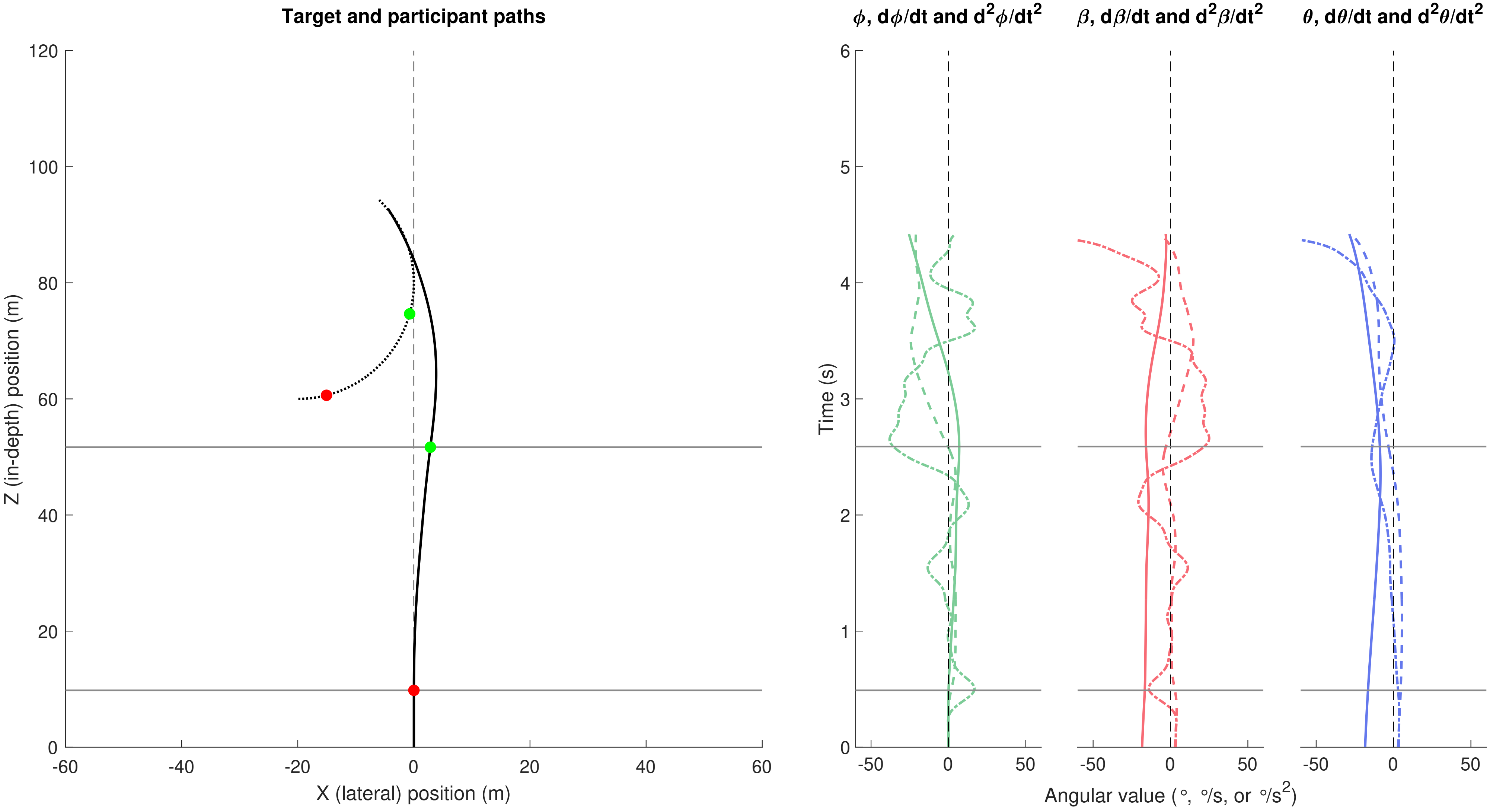

P13/B3
S20/R20-OUT

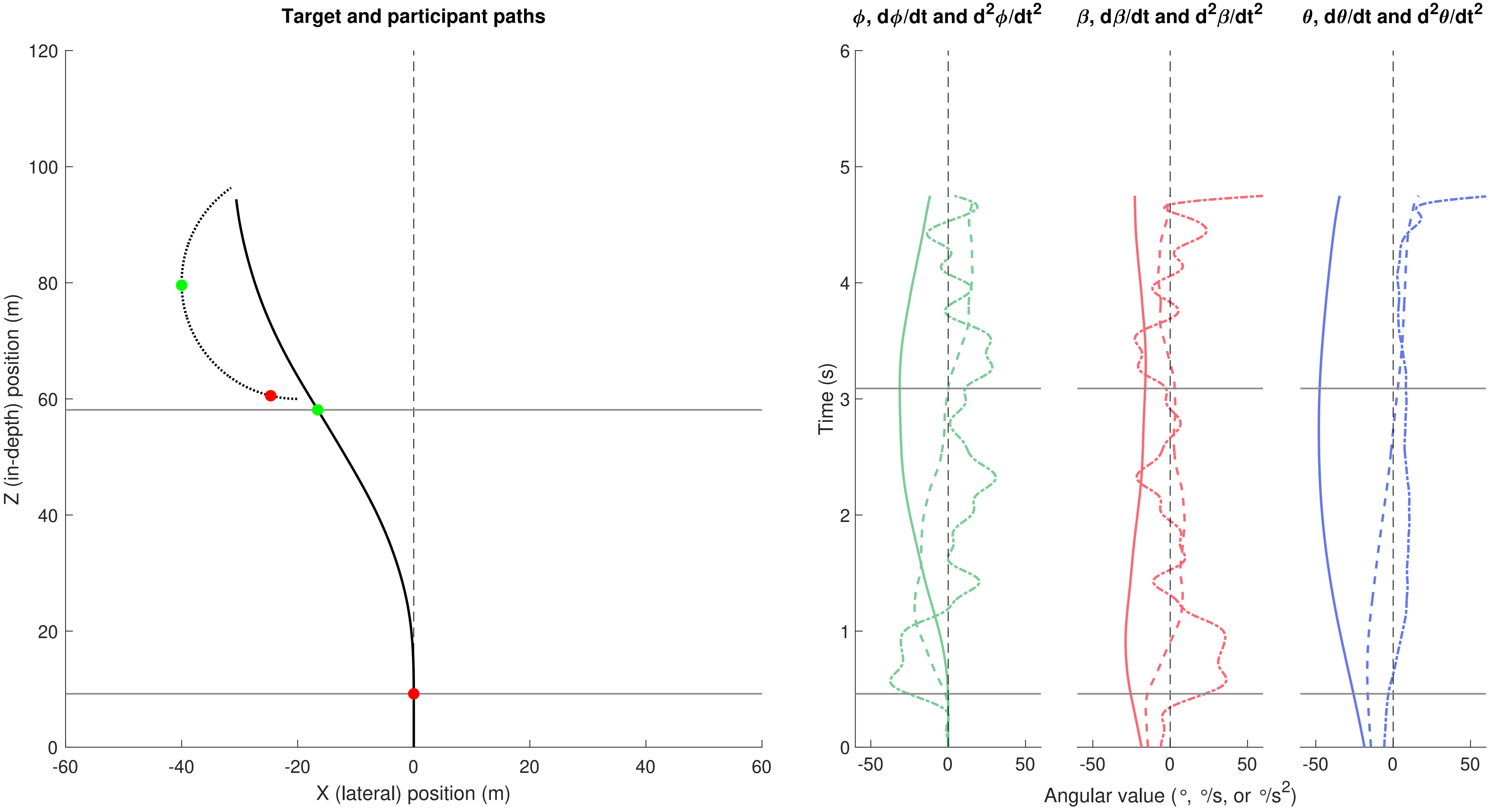

P13/B3
S20/R40-IN

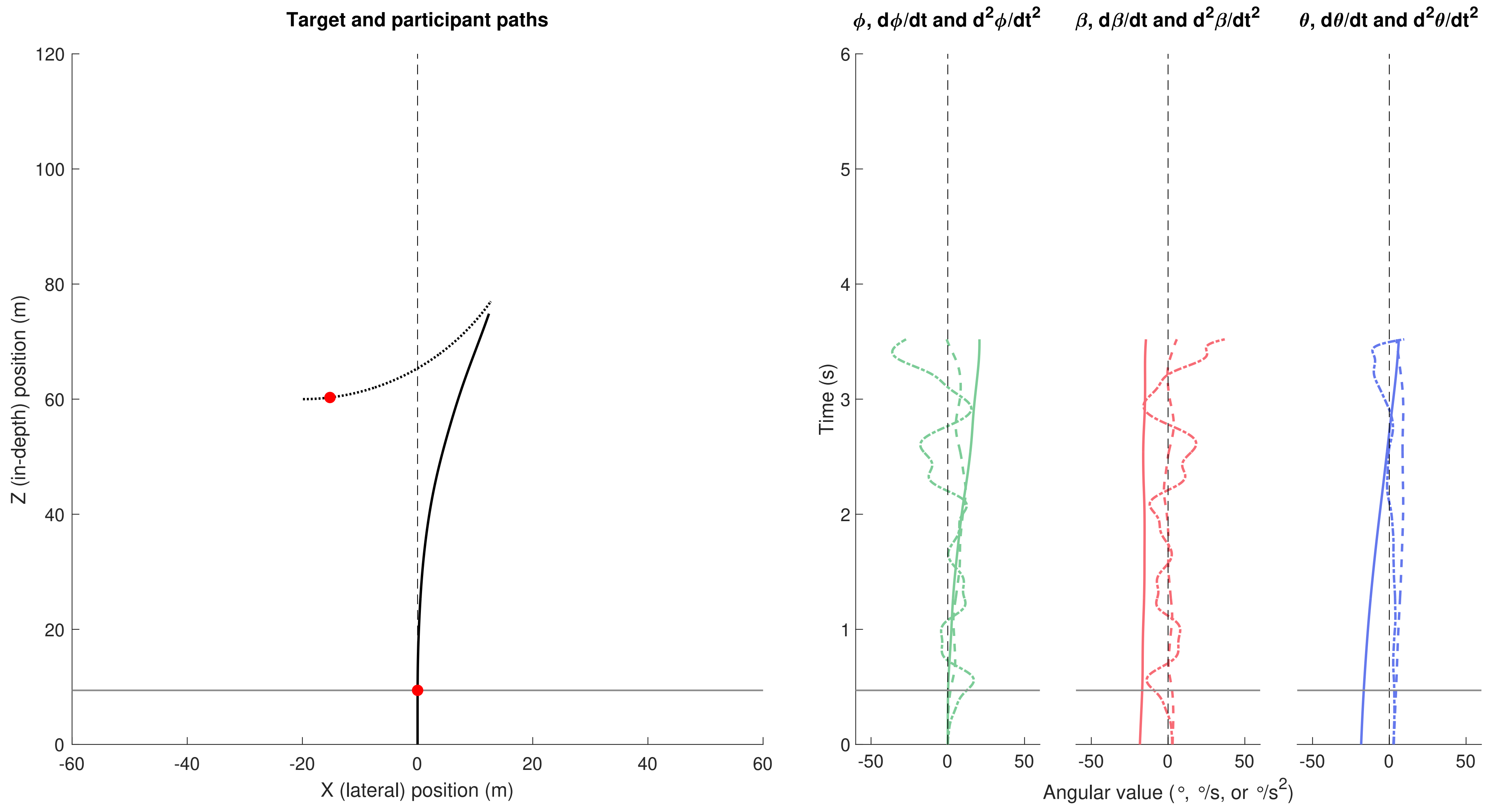

P13/B3
S20/R40-OUT

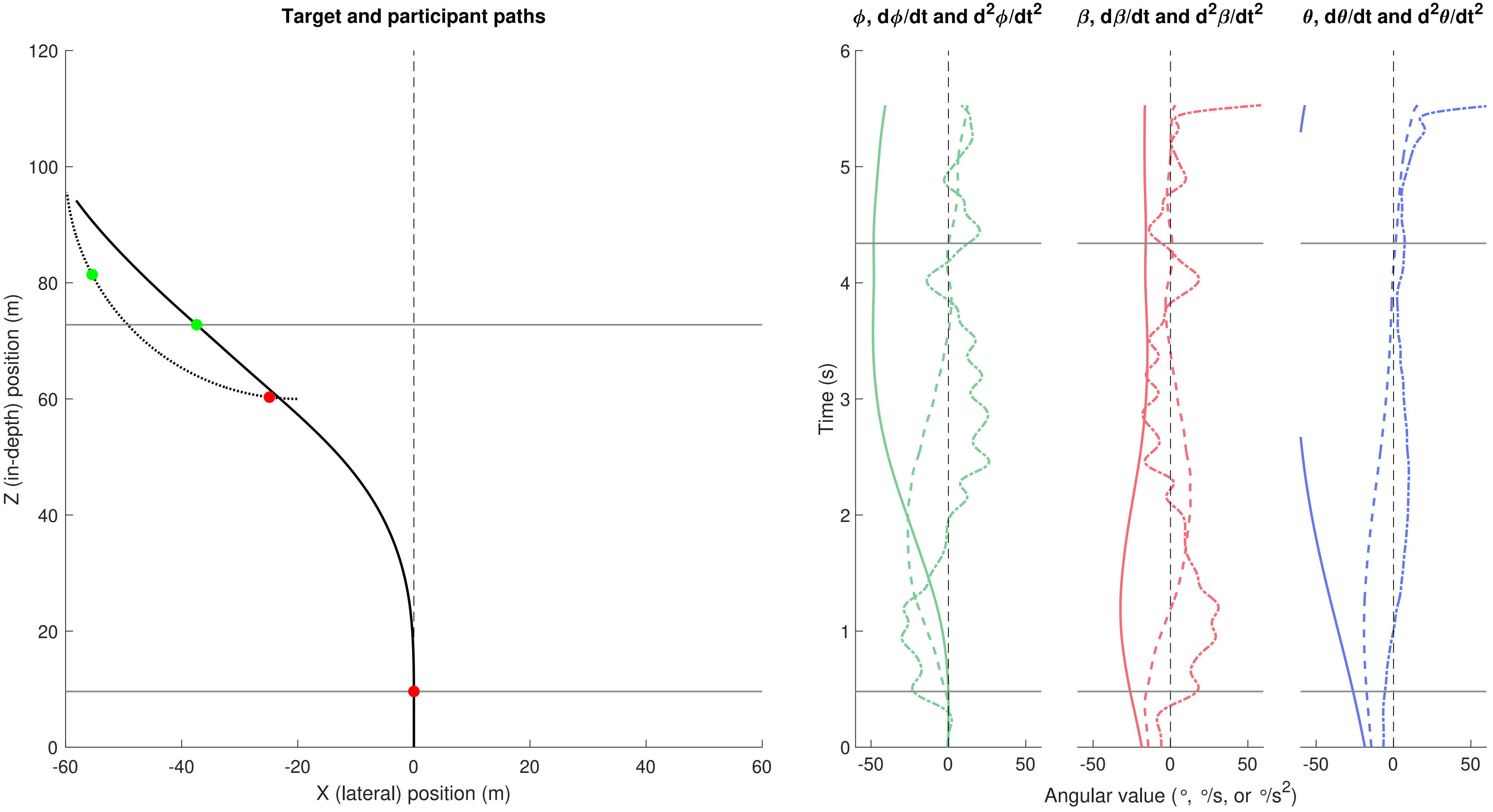

P13/B3
S10/R20-IN

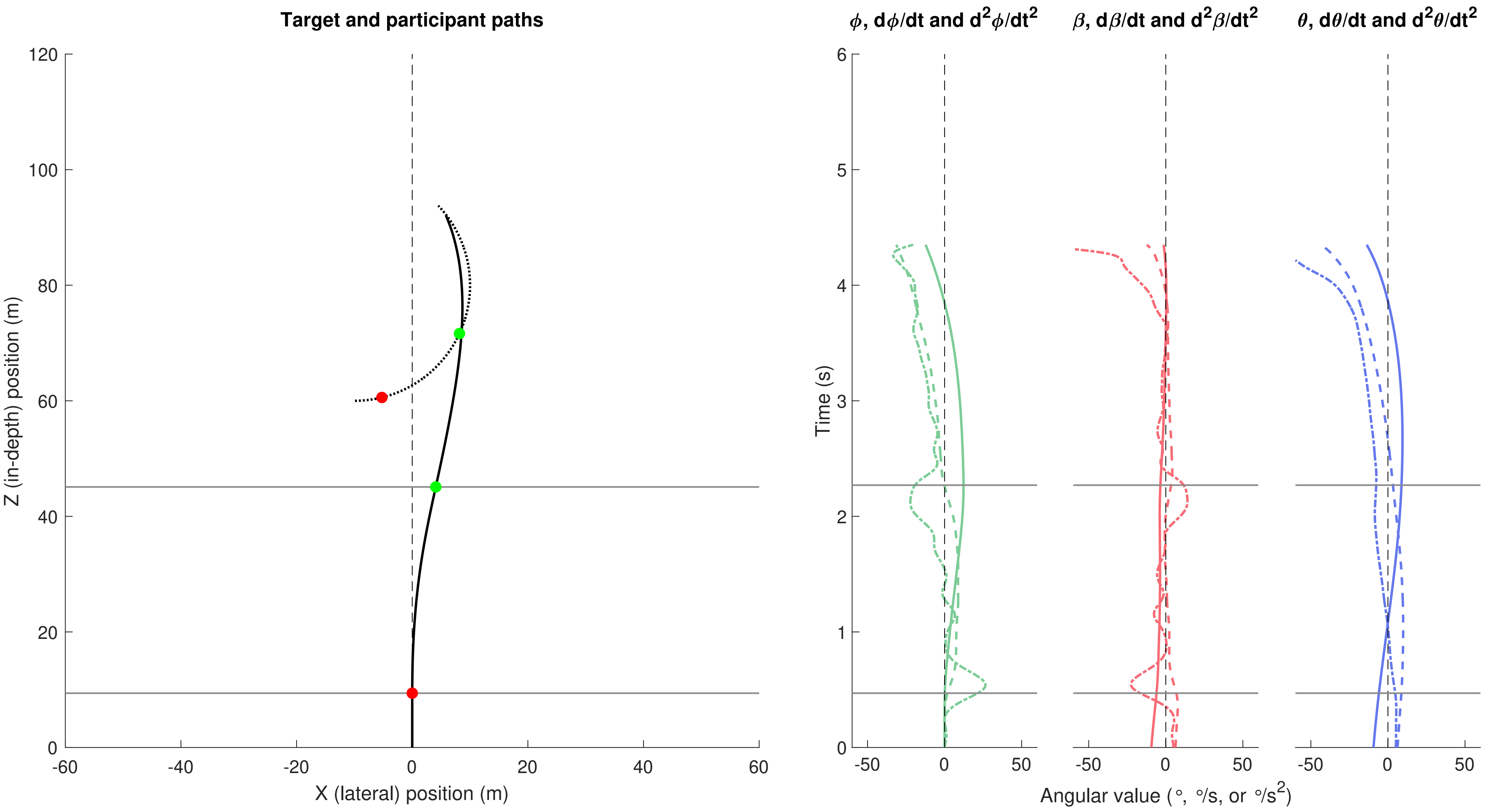

P13/B3
S10/R20-OUT

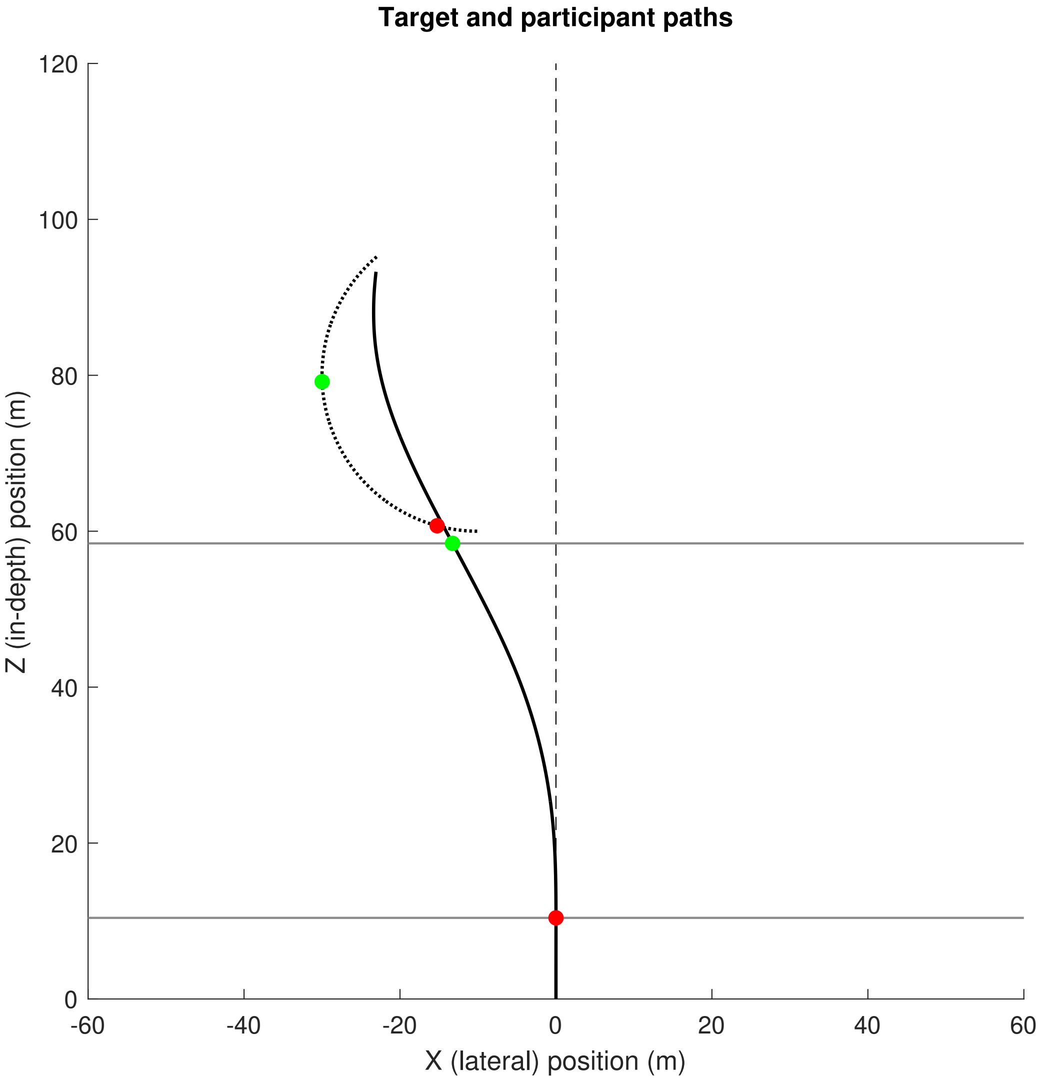

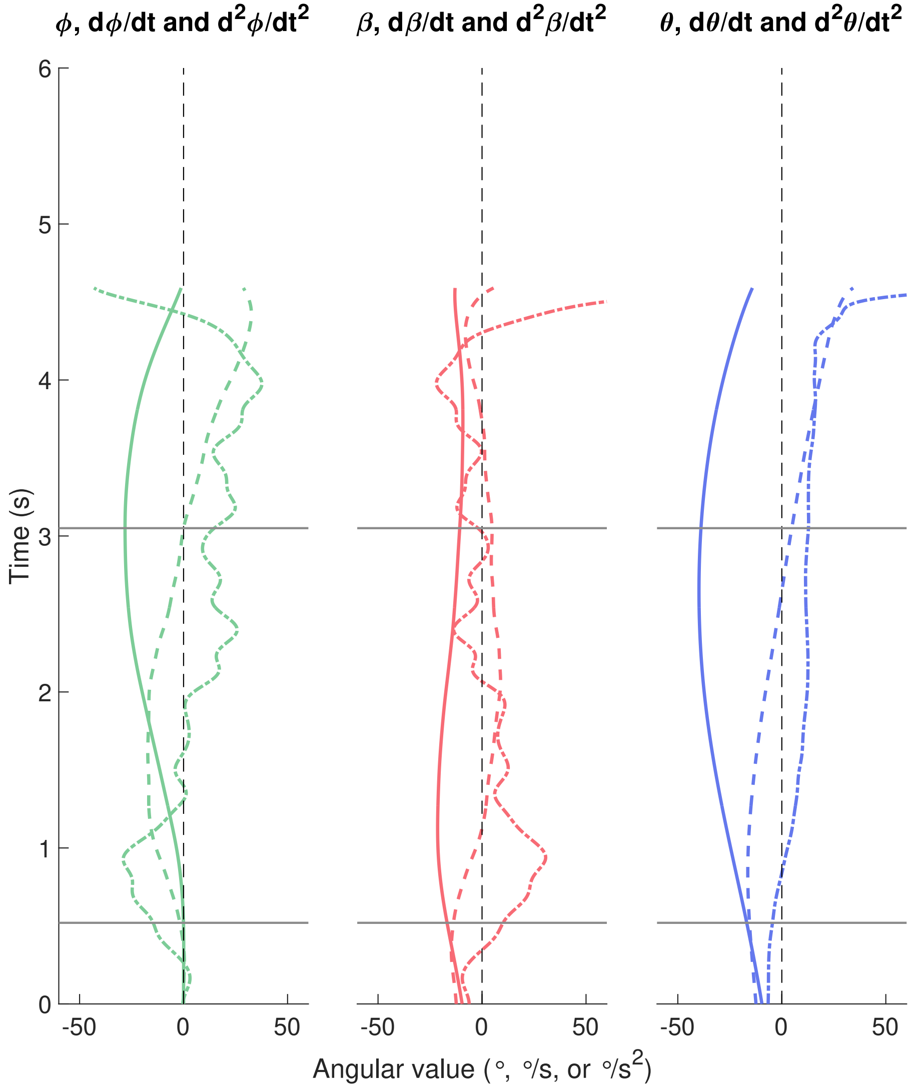

P13/B3
S10/R40-IN

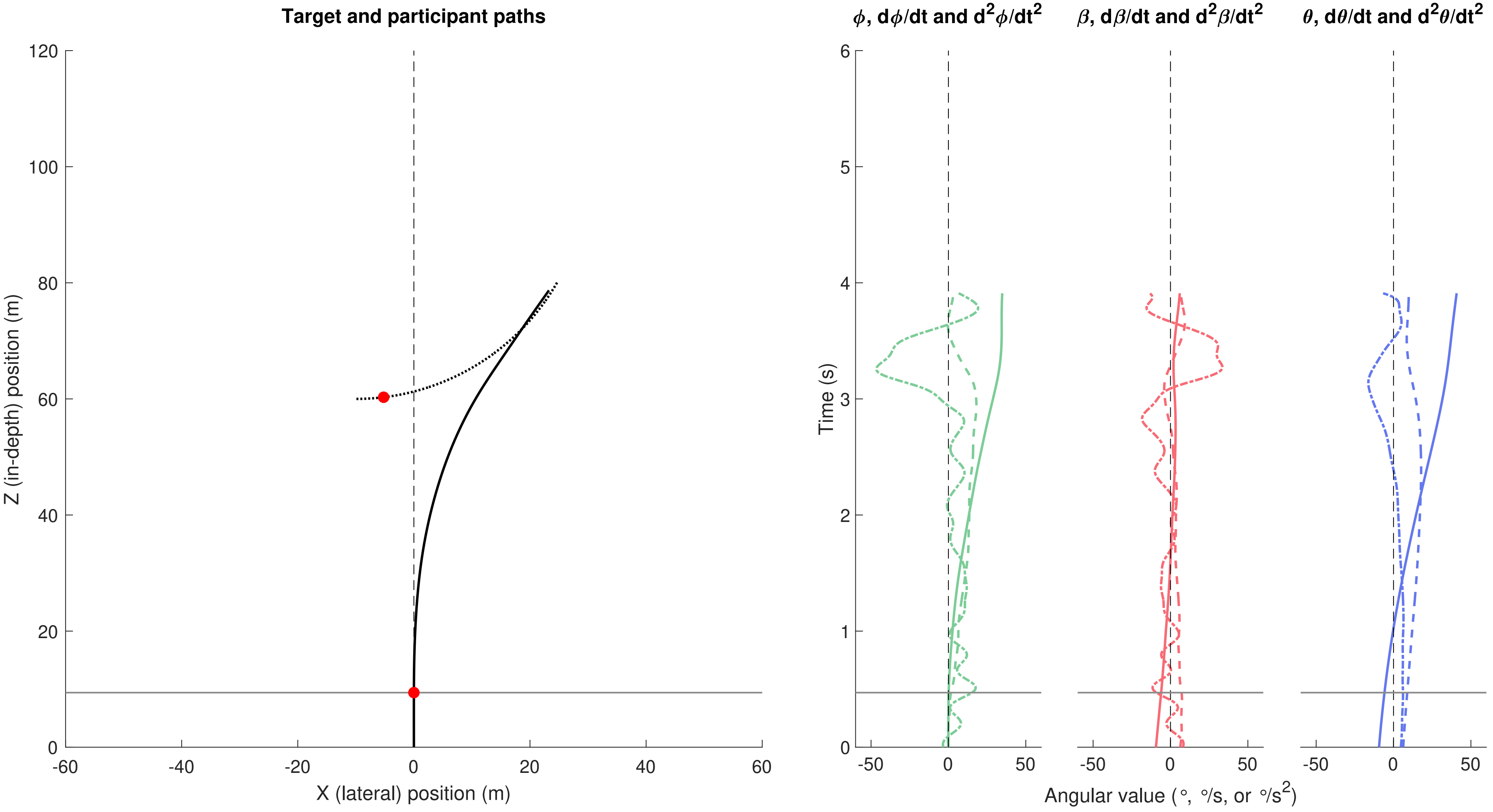

P13/B3
S10/R40-OUT

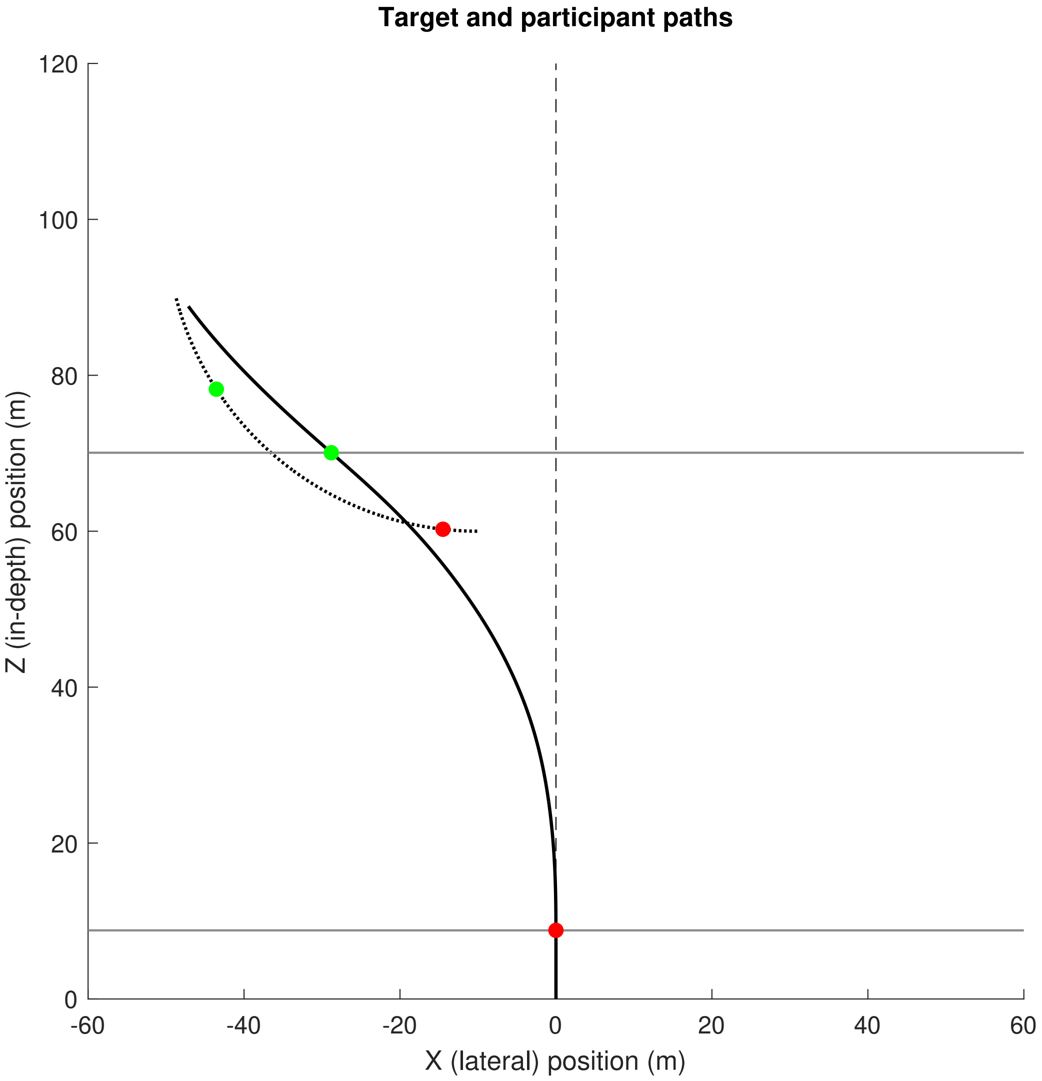

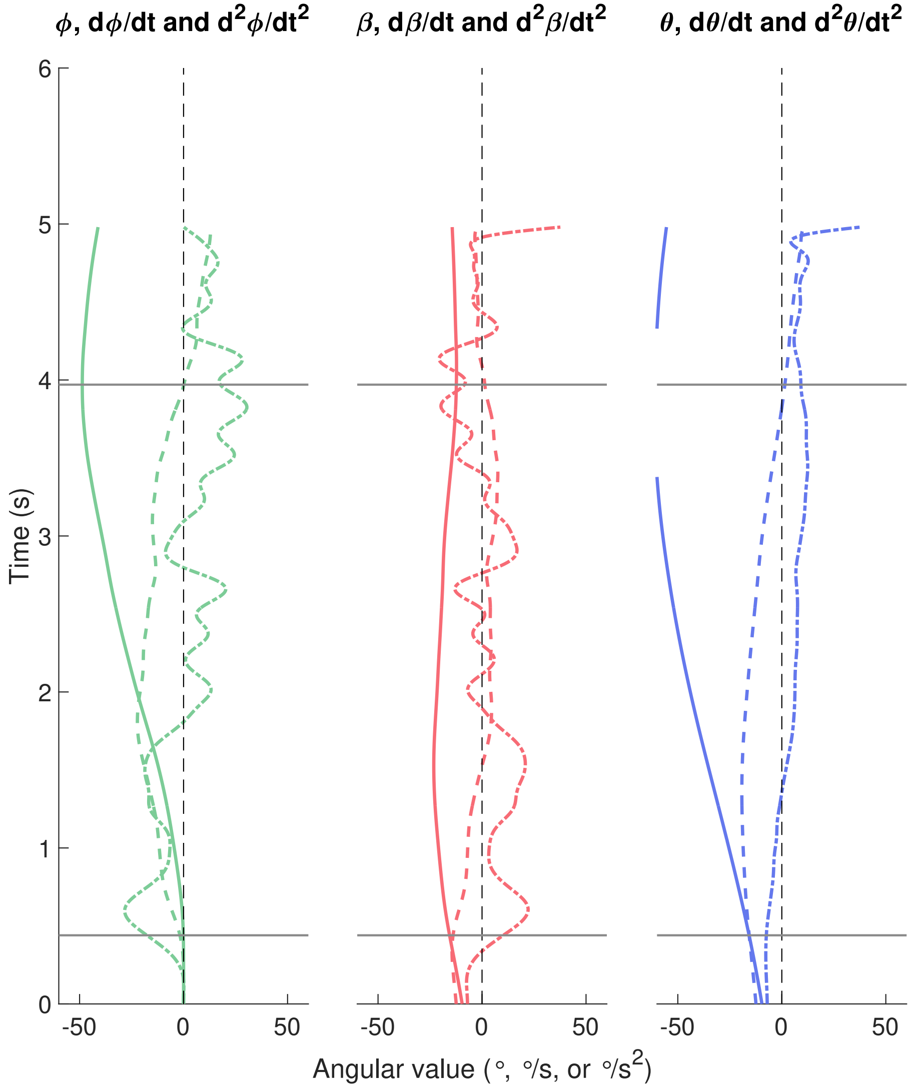

P13/B3
S0/R20-OUT

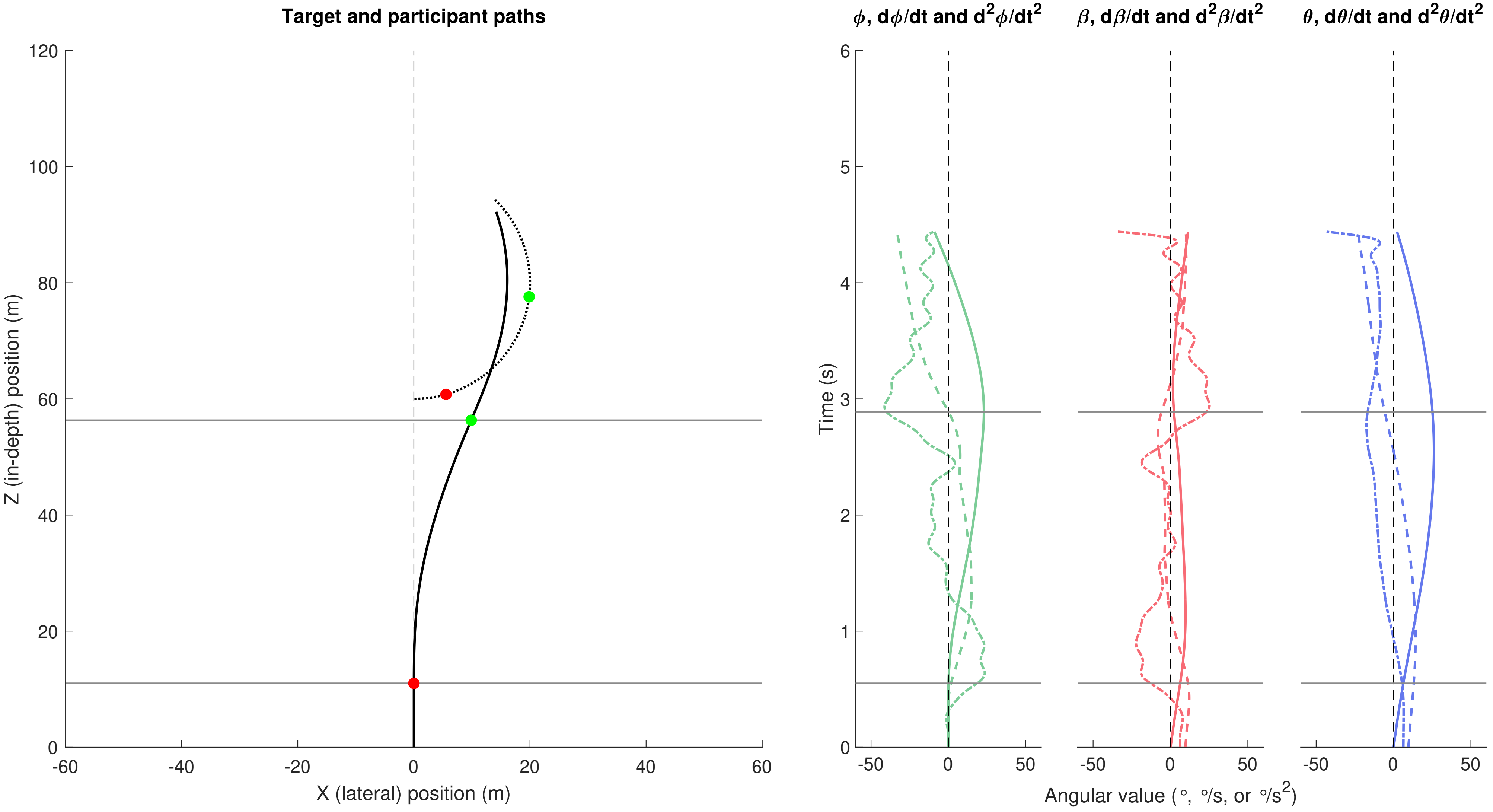

P13/B3
S0/R20-OUT

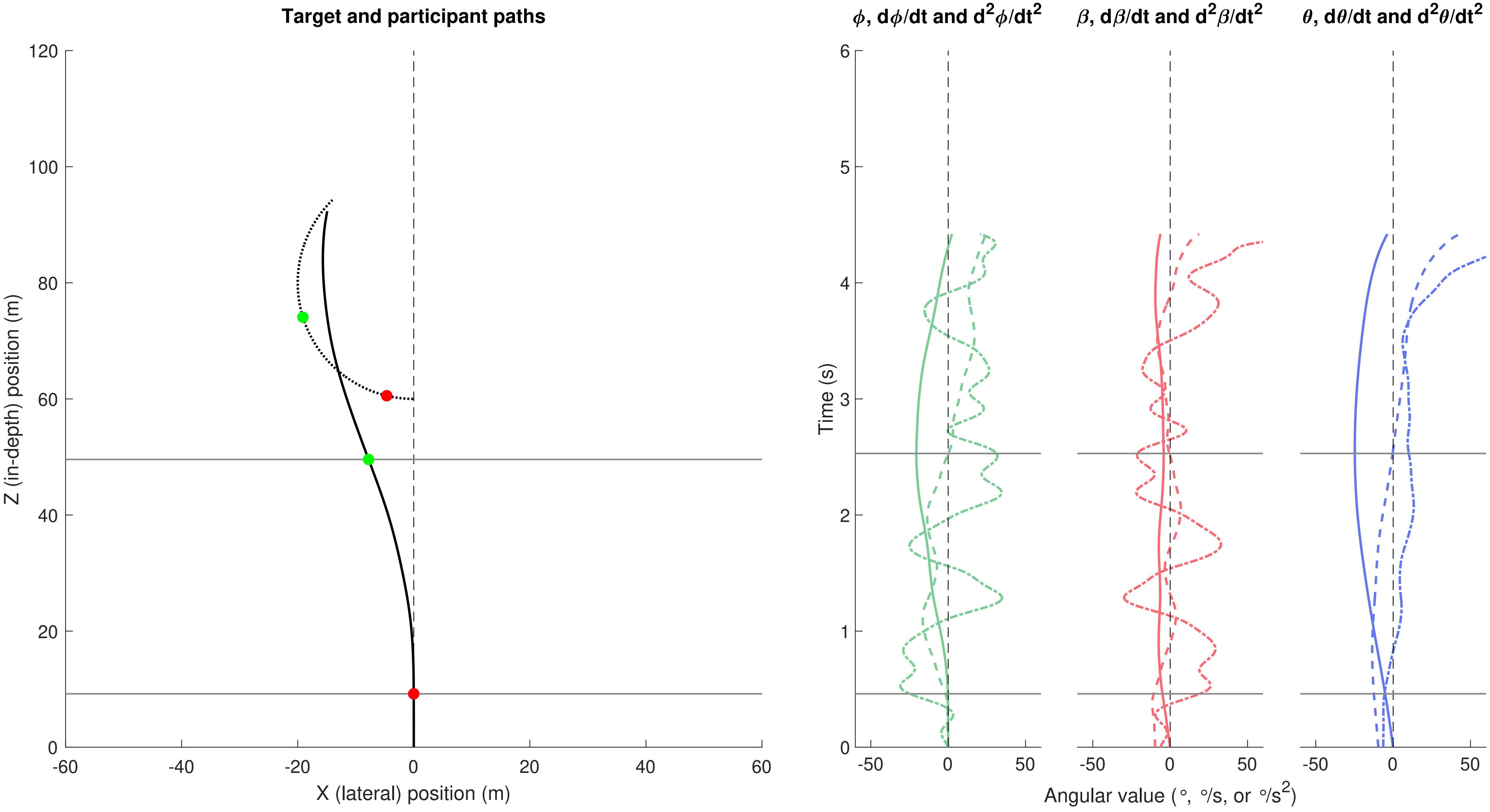

P13/B3
S0/R40-OUT

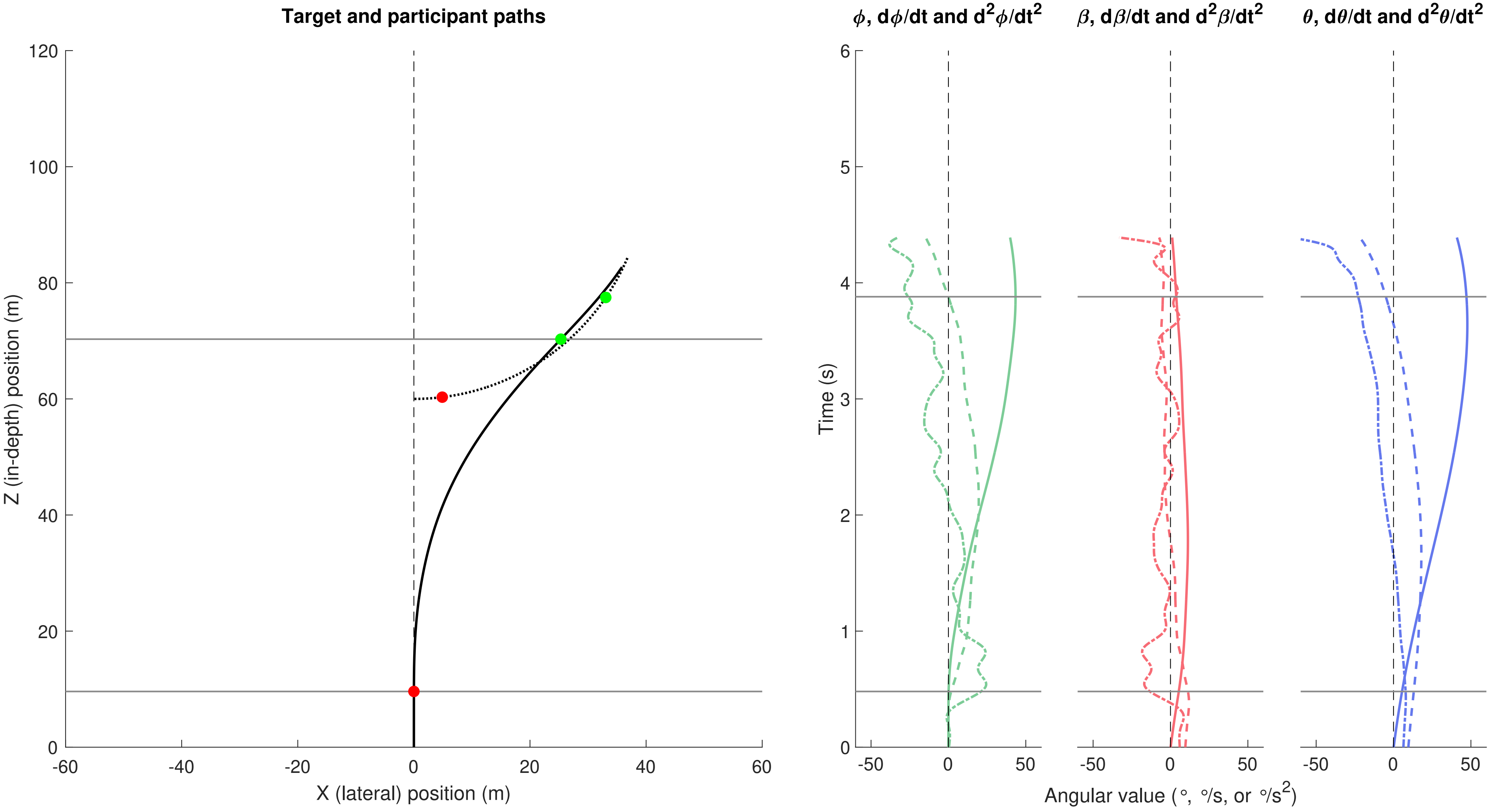

P13/B3
S0/R40-OUT

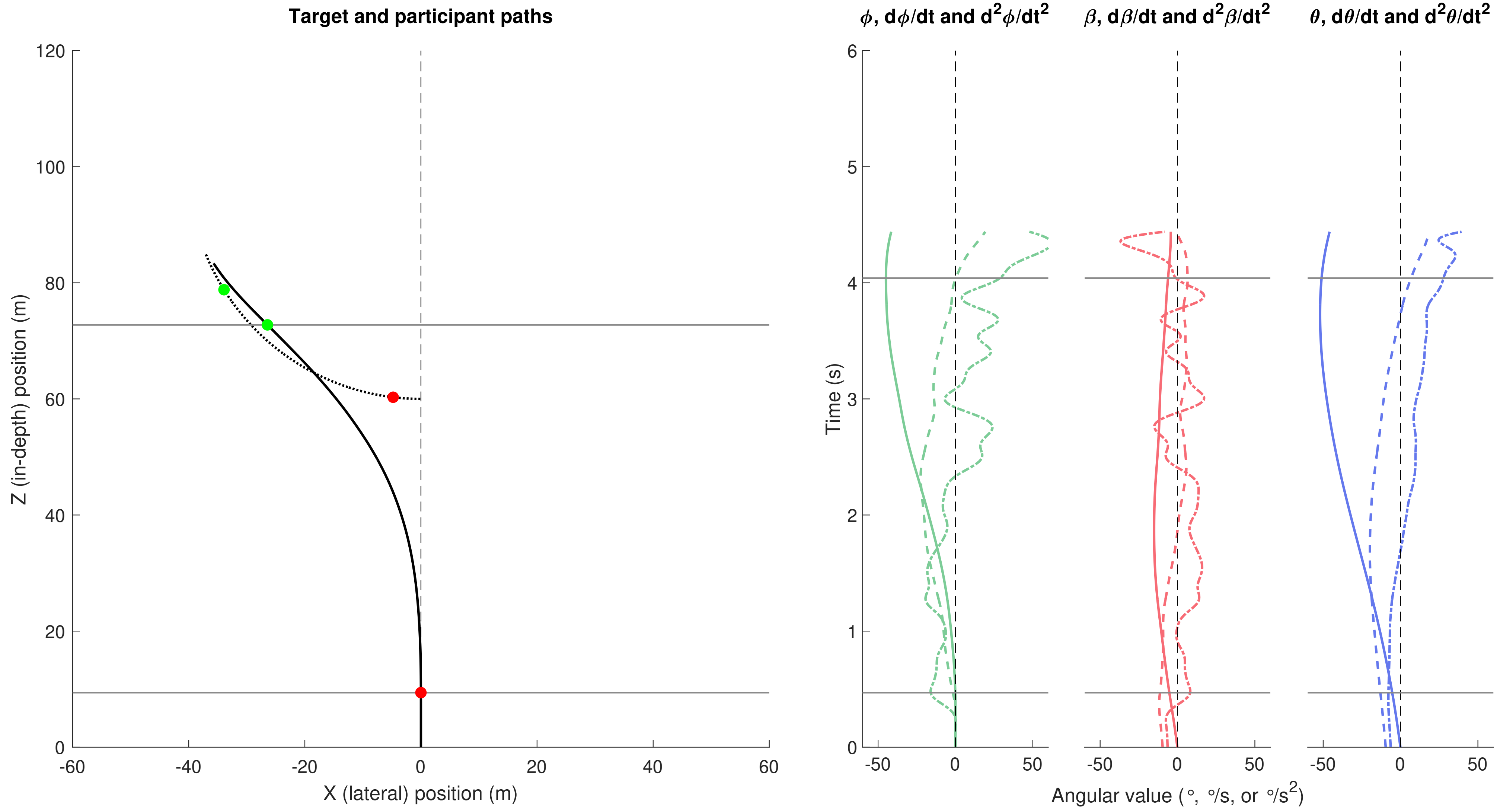

P13/B3
S10/R20-OUT

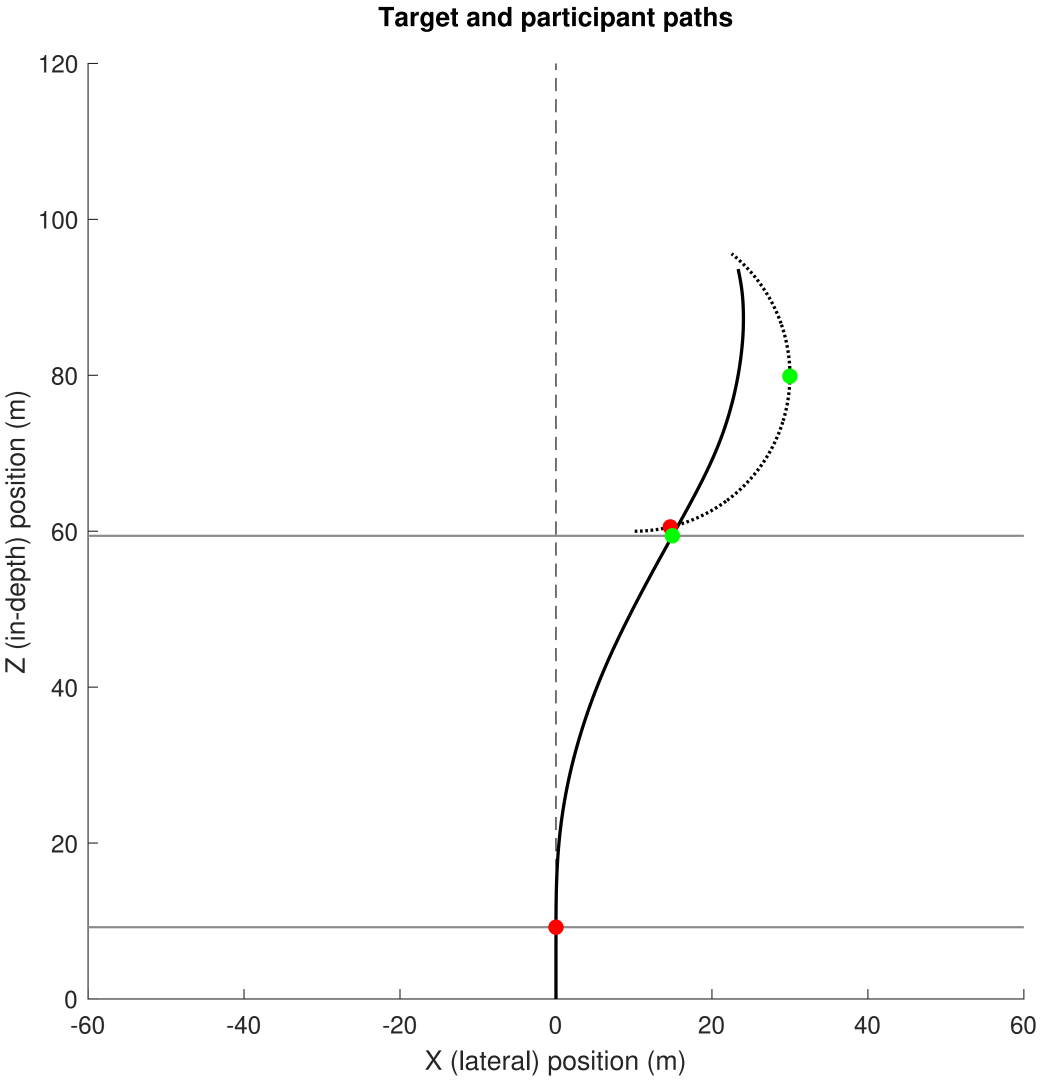

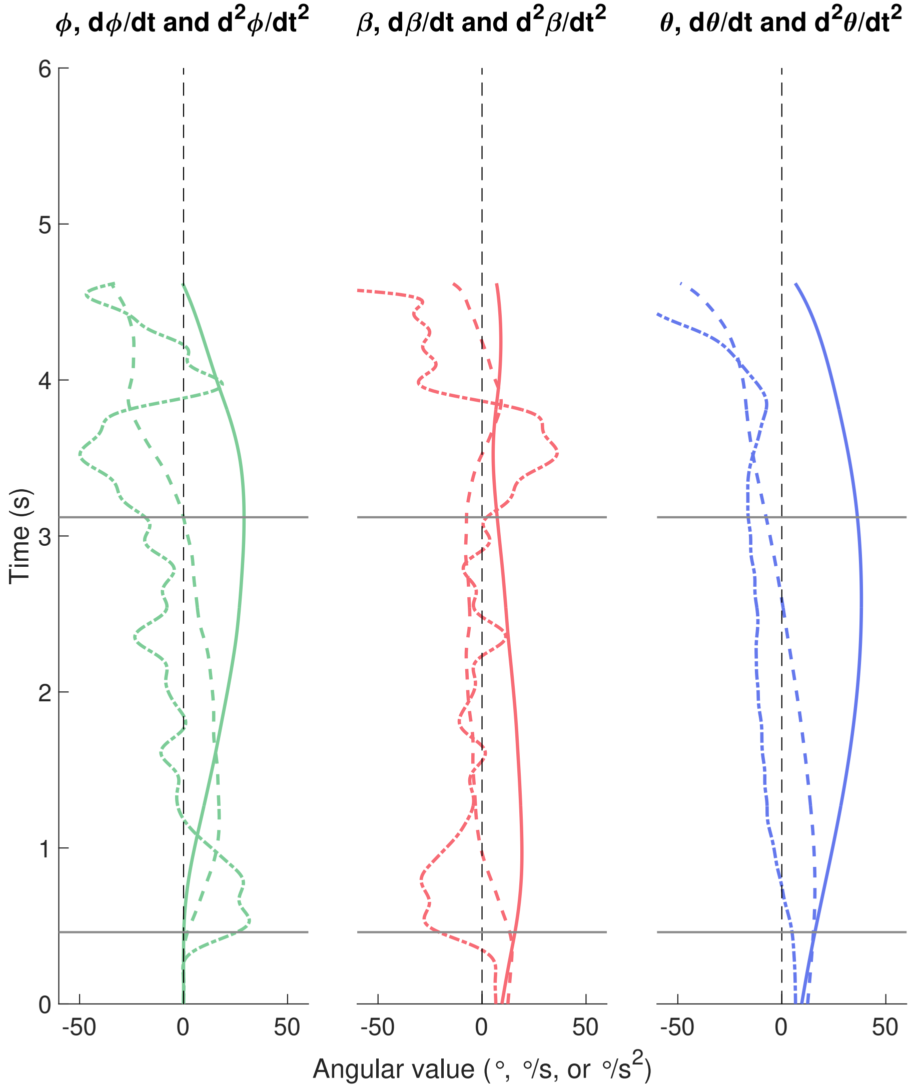

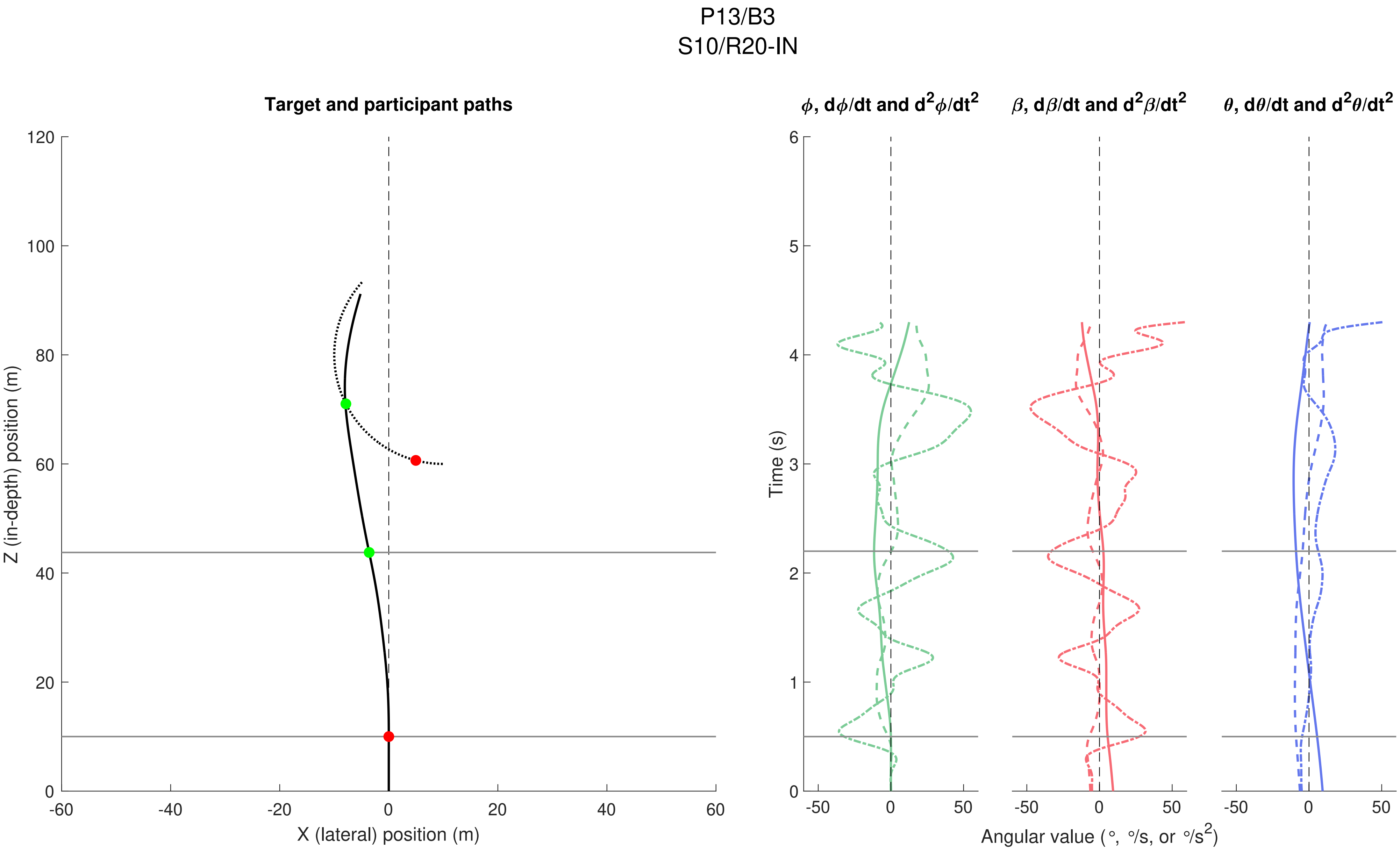
P13/B3
S10/R20-IN
Target and participant paths
Z (in-depth) position (m)
X (lateral) position (m)
120
100
80
60
40
20
0
-60
-40
-20
0
20
40
60
ϕ, dϕ/dt and d$^2\phi$/dt^2
β, dβ/dt and d$^2\beta$/dt^2
θ, dθ/dt and d$^2\theta$/dt^2
Time (s)
6
5
4
3
2
1
0
-50
0
50
Angular value (°, °/s, or °/s^2)

P13/B3
S10/R40-OUT

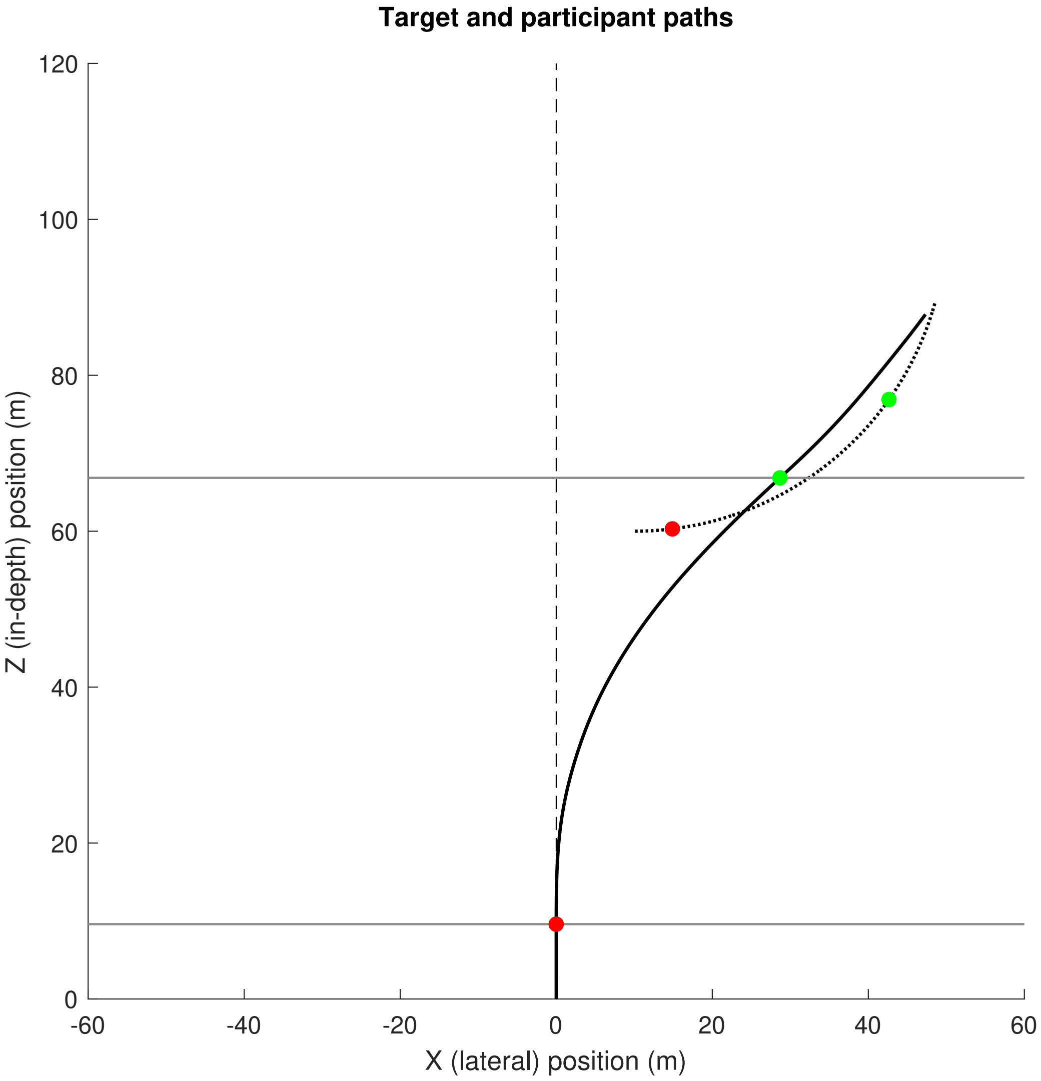

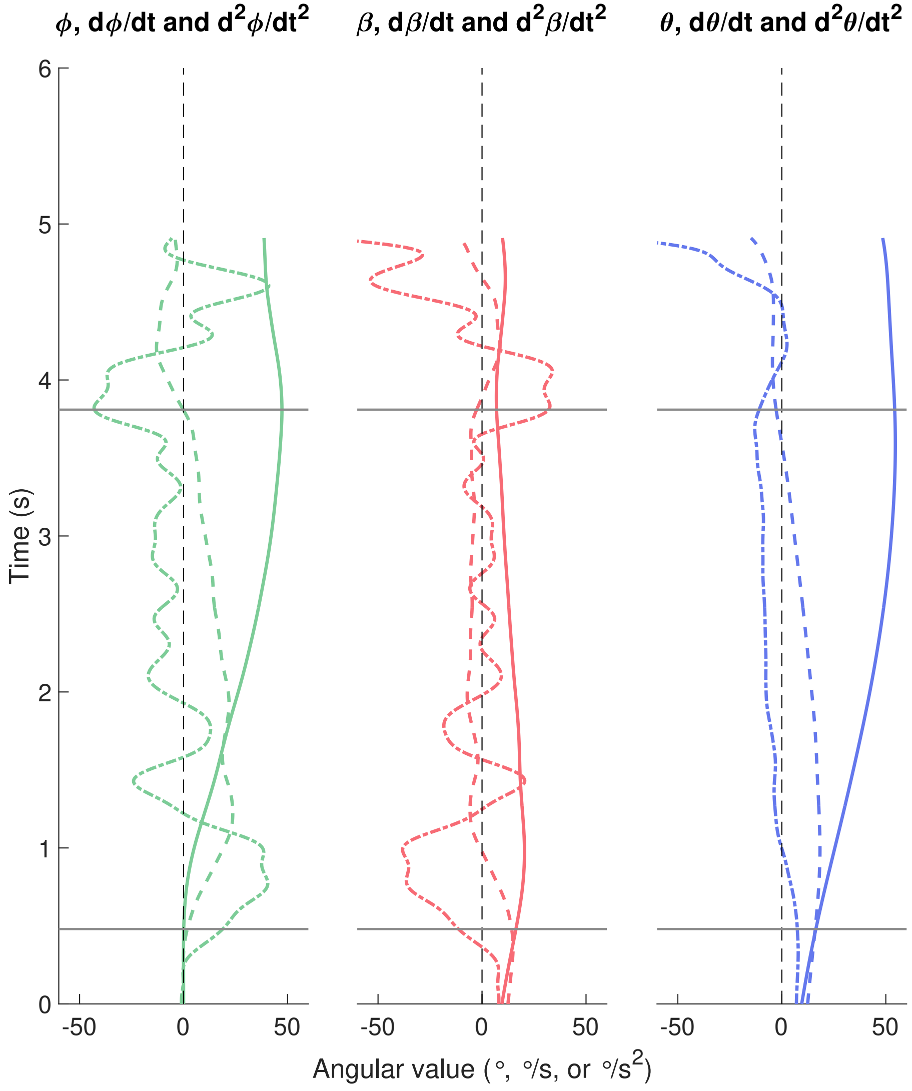

P13/B3
S10/R40-IN

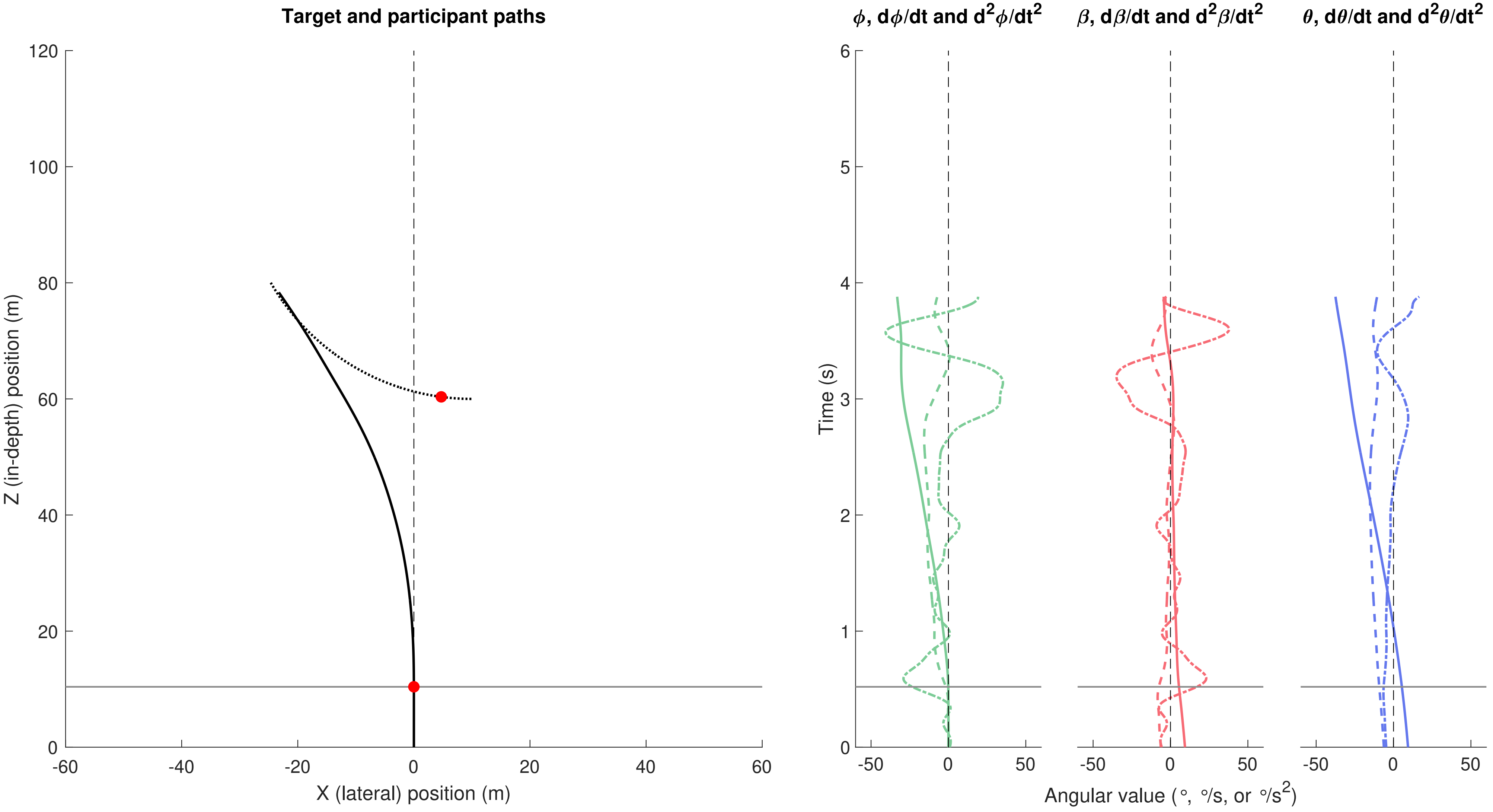

P13/B3
S20/R20-OUT

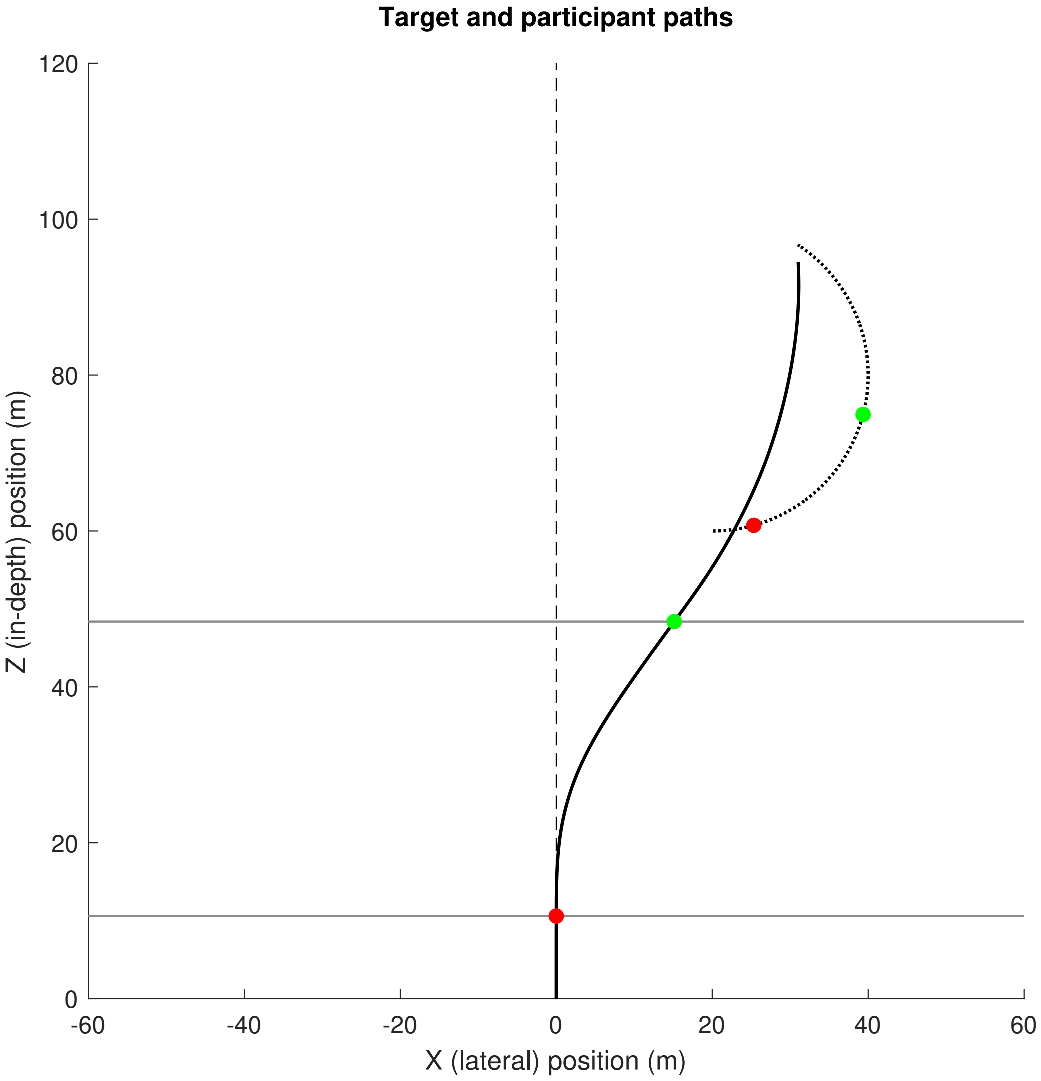

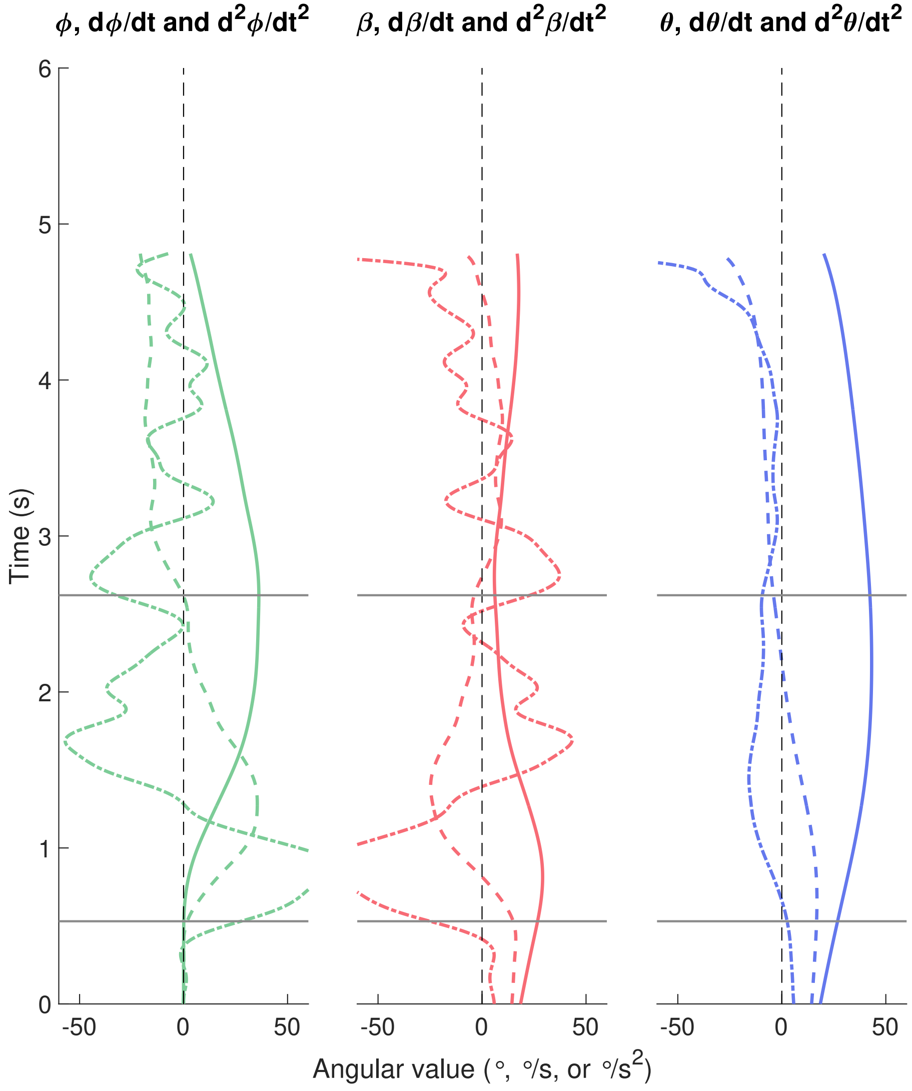

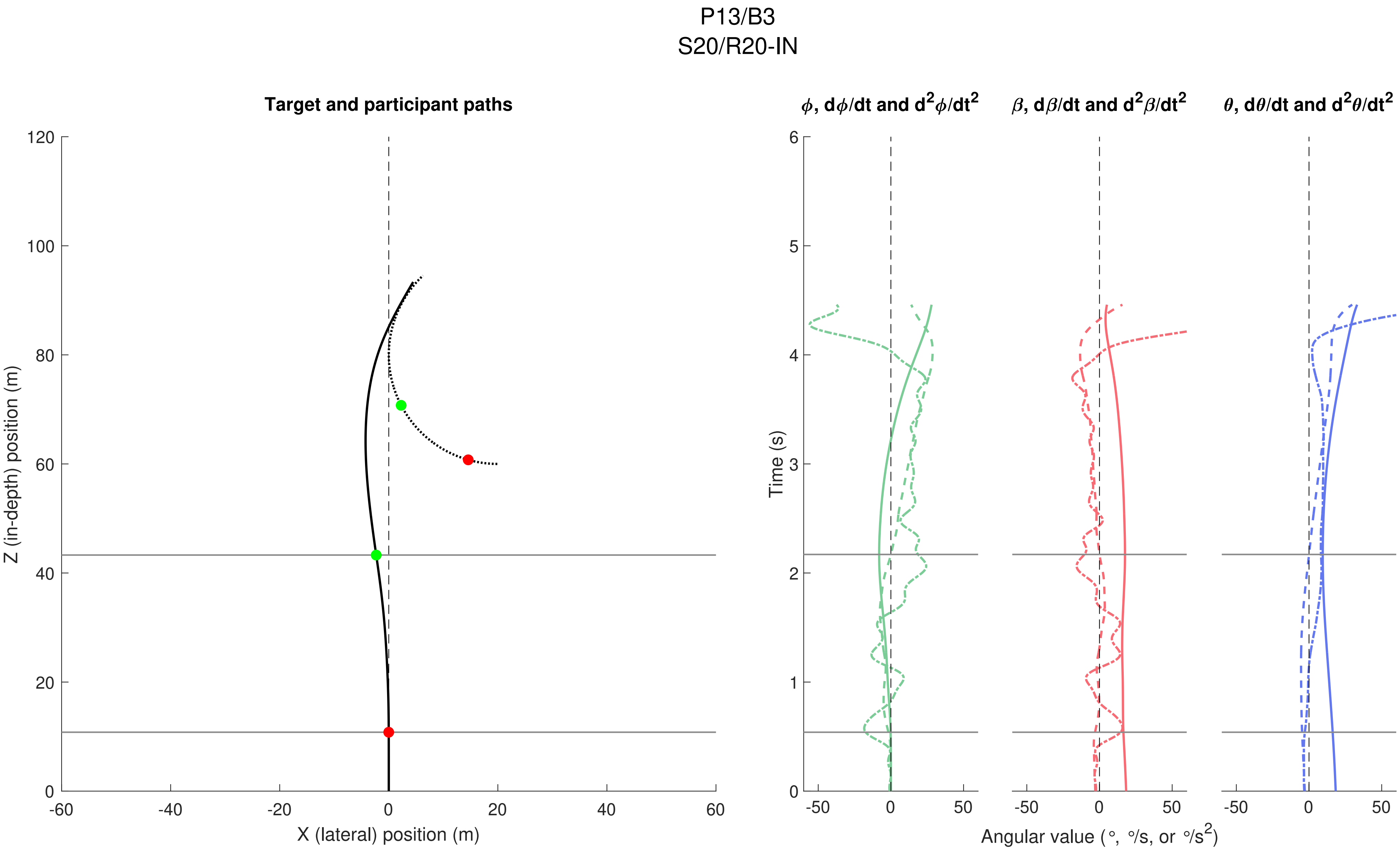
P13/B3
S20/R20-IN
Target and participant paths
Z (in-depth) position (m)
X (lateral) position (m)
120
100
80
60
40
20
0
-60
-40
-20
0
20
40
60
ϕ, dϕ/dt and d²ϕ/dt²
β, dβ/dt and d²β/dt²
θ, dθ/dt and d²θ/dt²
Time (s)
6
5
4
3
2
1
0
-50
0
50
Angular value (°, °/s, or °/s²)

P13/B3
S20/R40-OUT

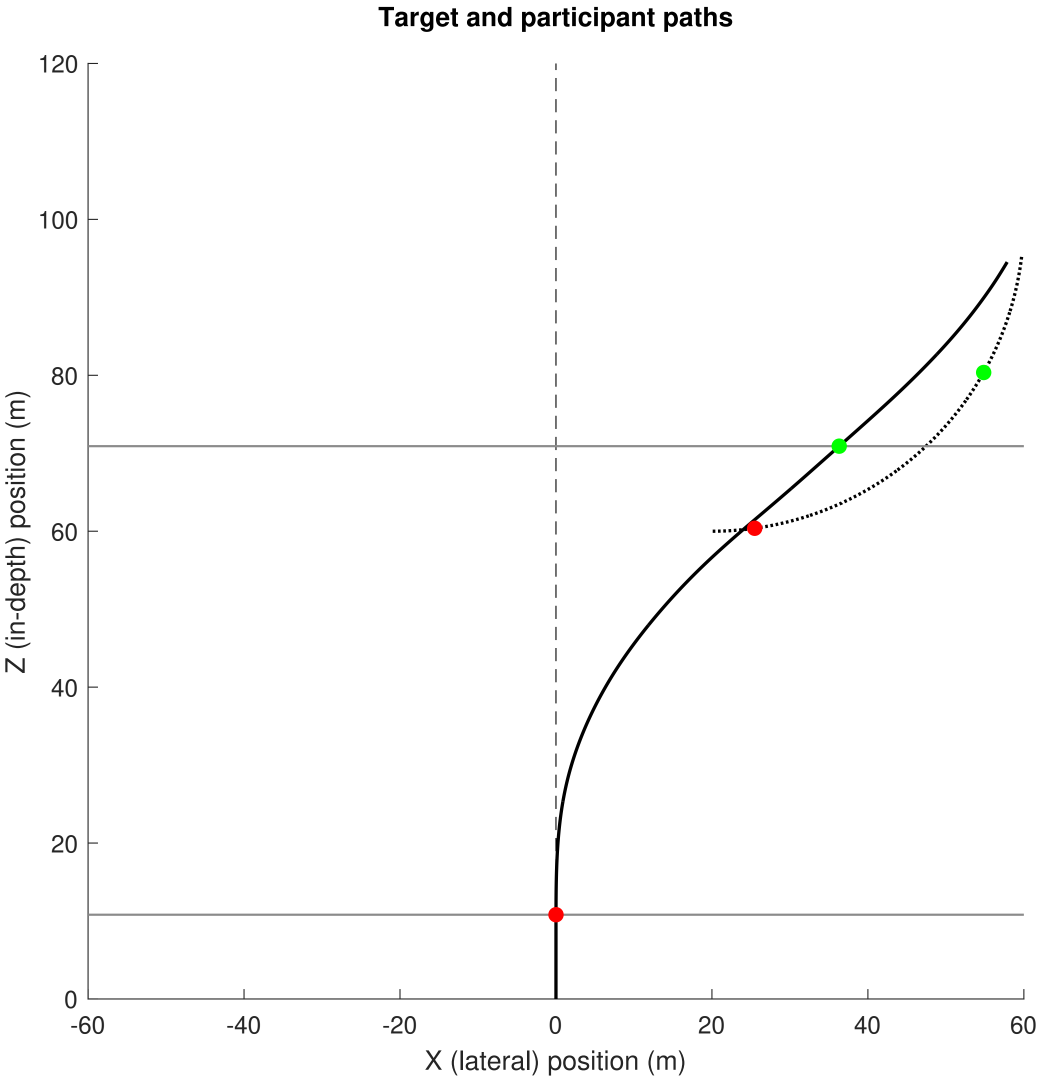

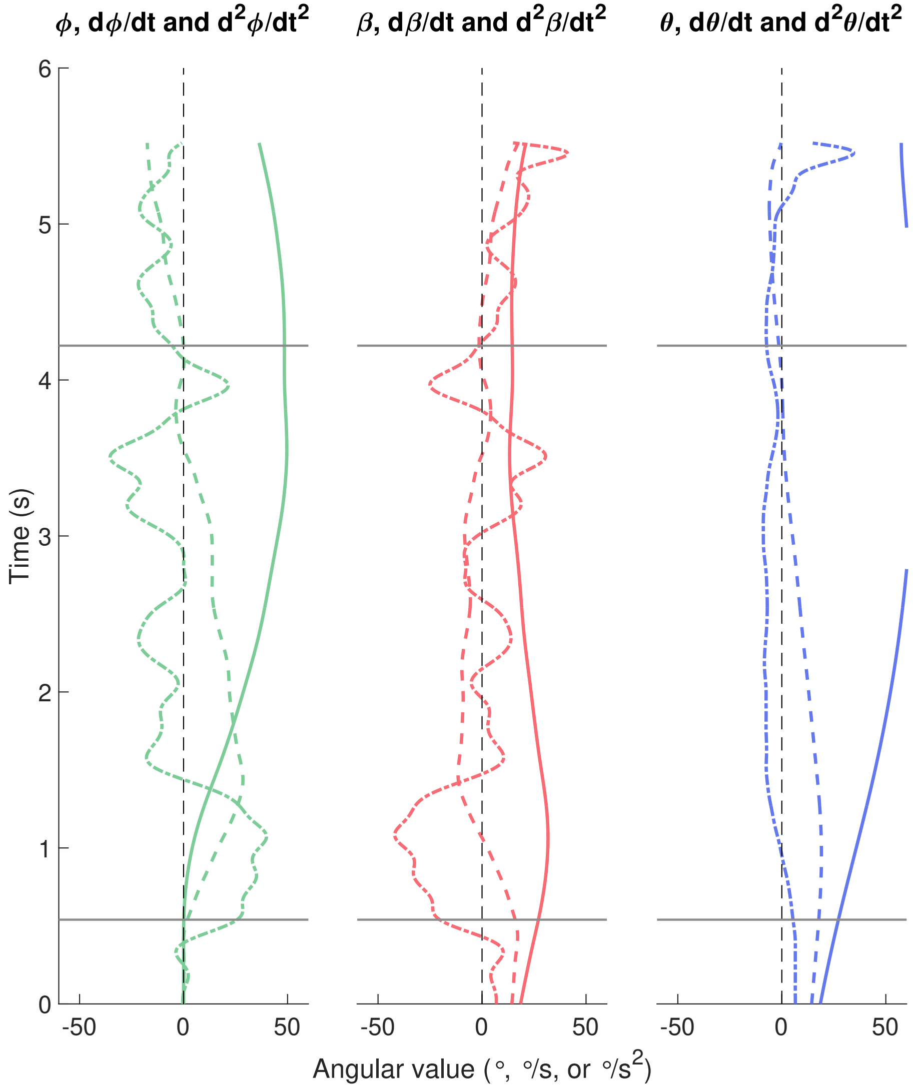

P13/B3
S20/R40-IN

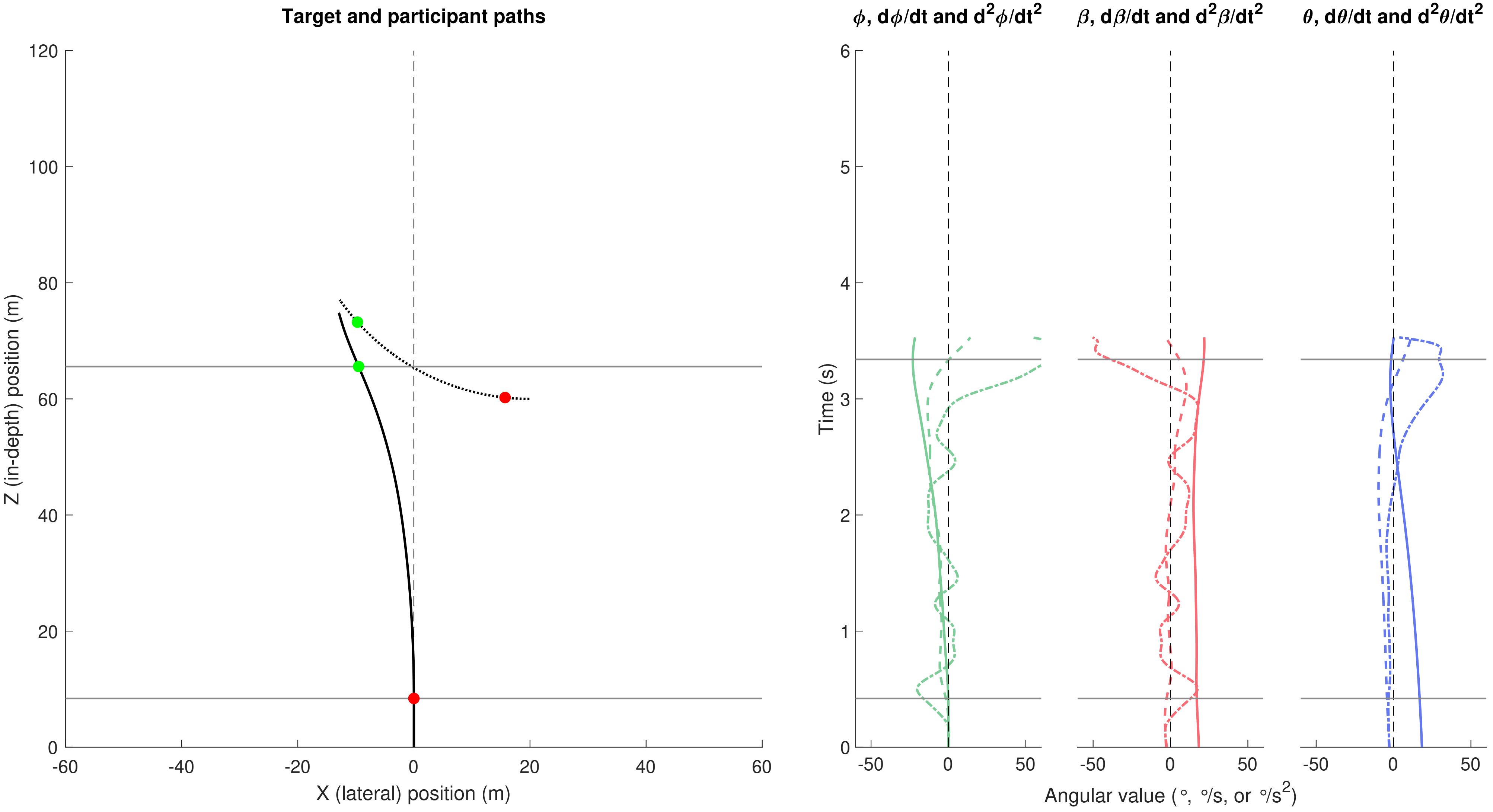

P13/B4
S20/R20-IN

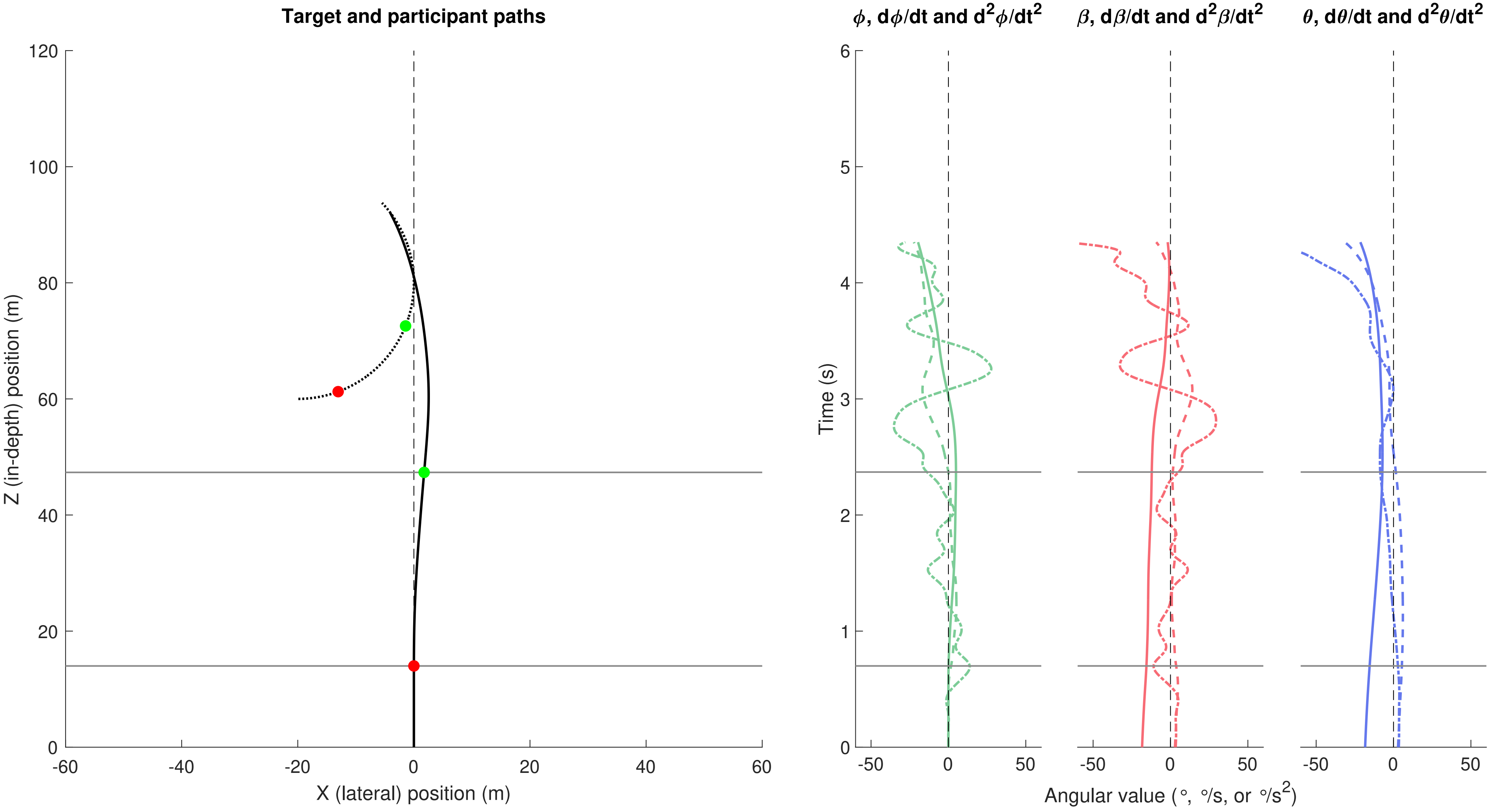

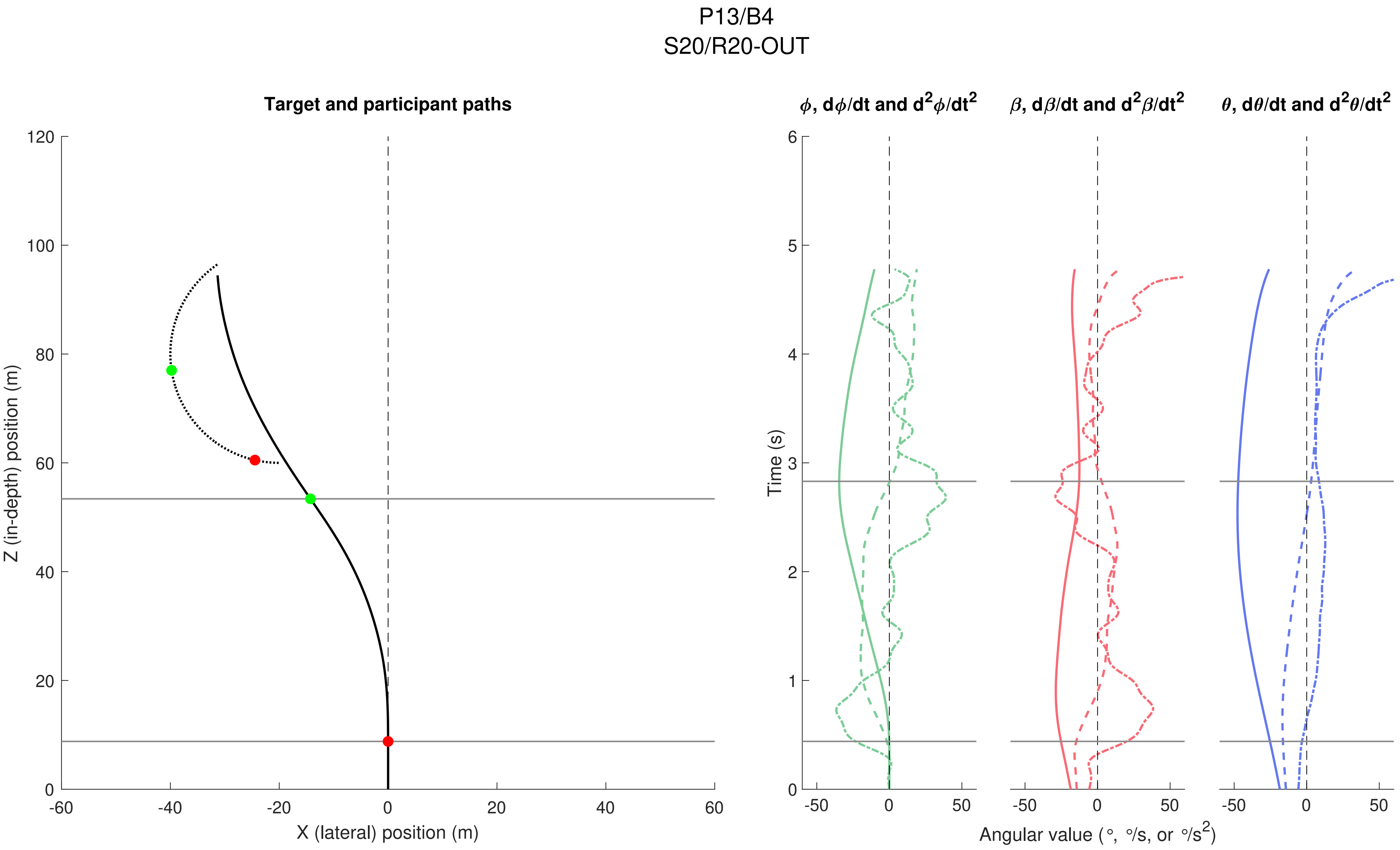

P13/B4
S20/R20-OUT
Target and participant paths
Z (in-depth) position (m)
X (lateral) position (m)
ϕ, dϕ/dt and d$^2\phi$/dt^2
β, dβ/dt and d$^2\beta$/dt^2
θ, dθ/dt and d$^2\theta$/dt^2
Time (s)
Angular value (°, °/s, or °/s^2)

P13/B4
S20/R40-IN

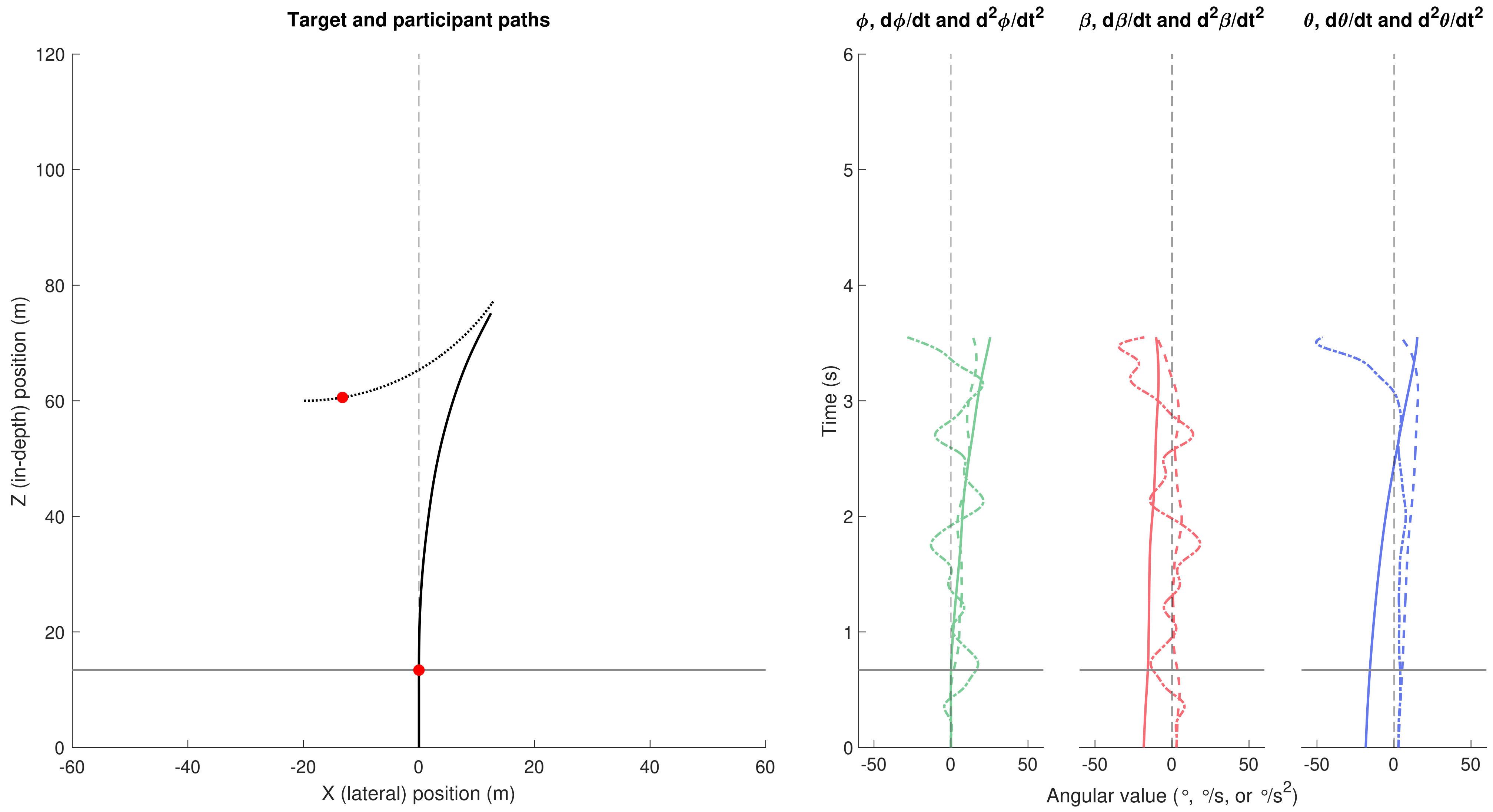

P13/B4
S20/R40-OUT

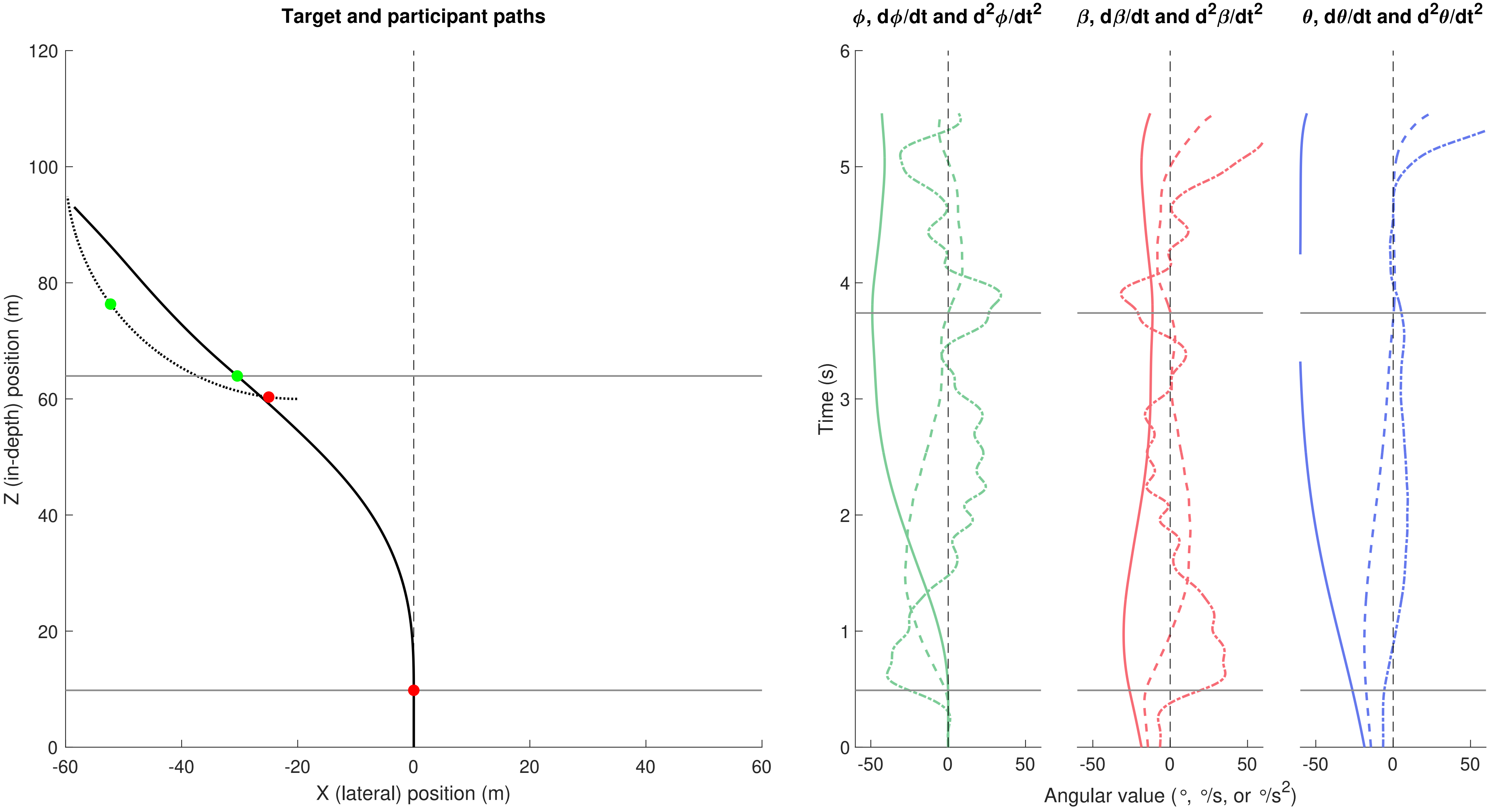

P13/B4
S10/R20-IN

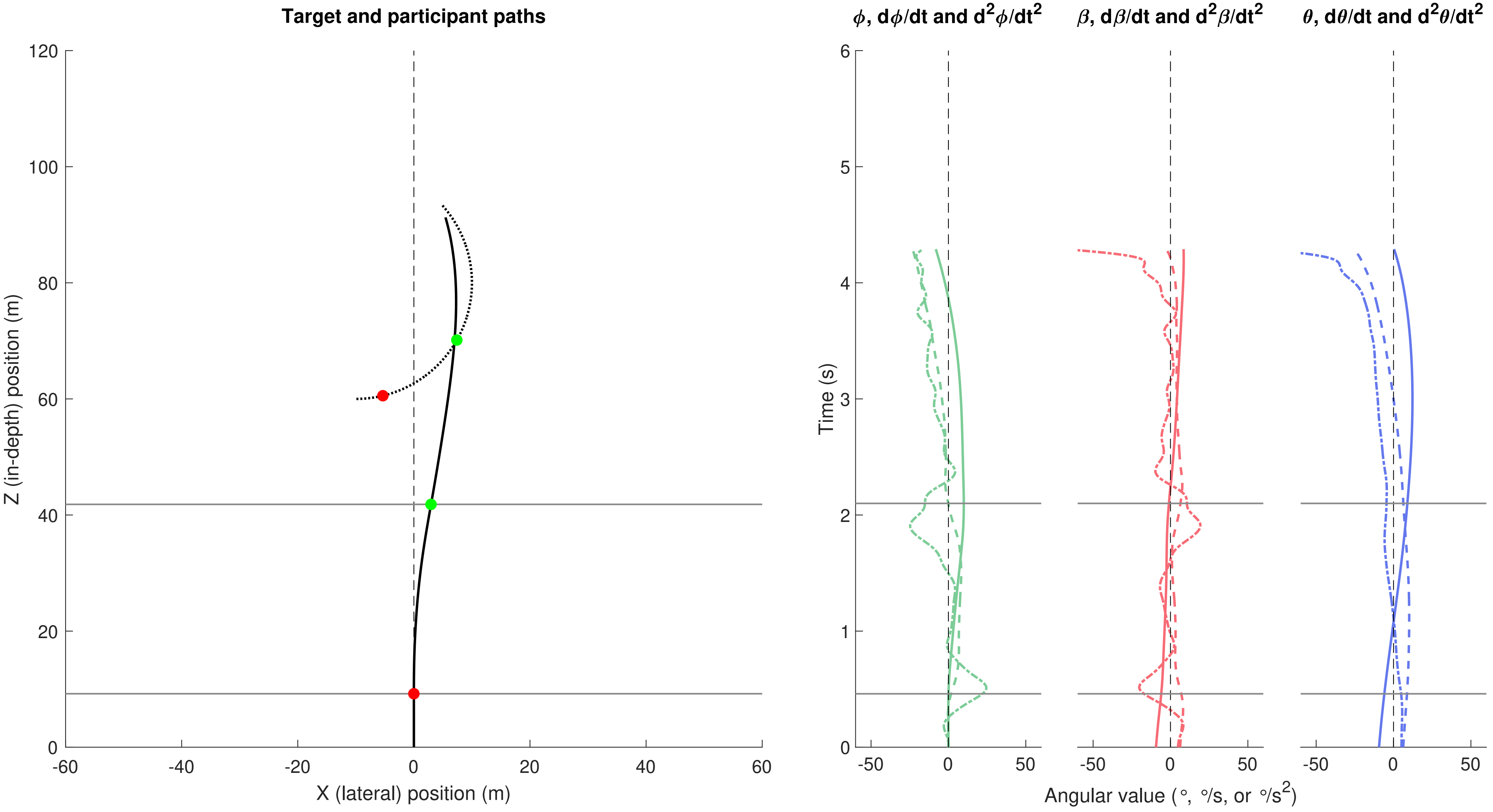

P13/B4
S10/R20-OUT

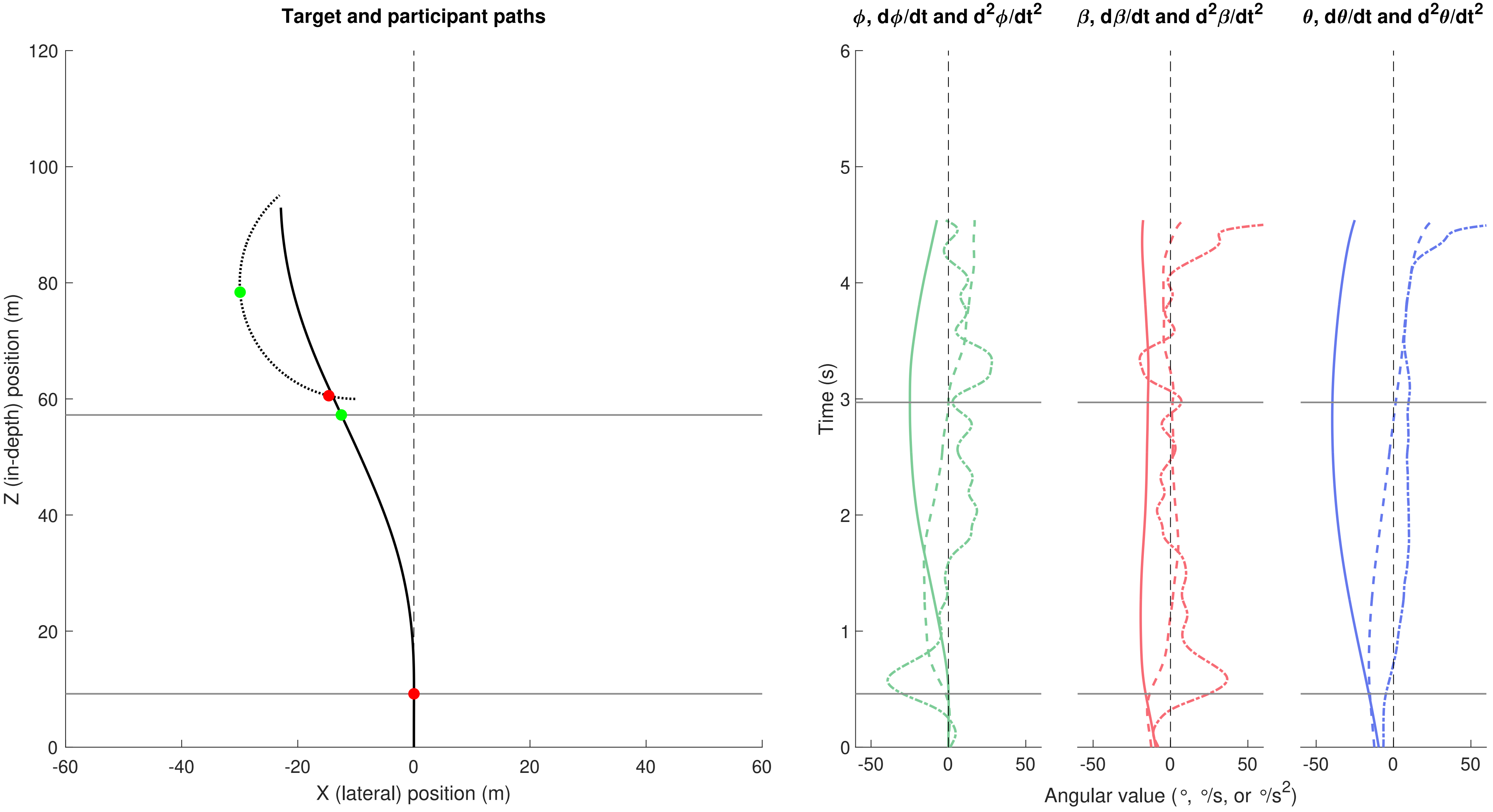

P13/B4
S10/R40-IN

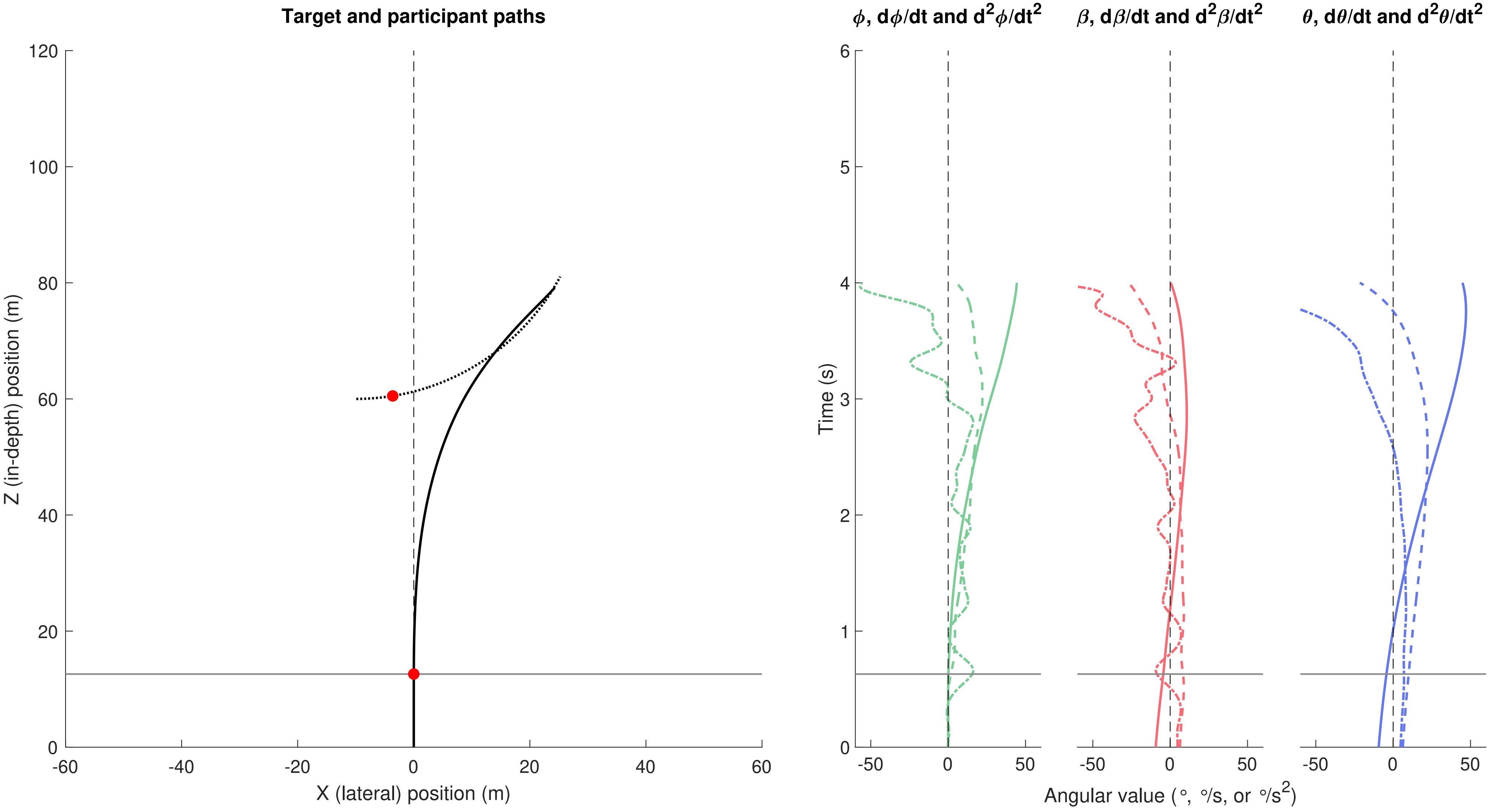

P13/B4
S10/R40-OUT

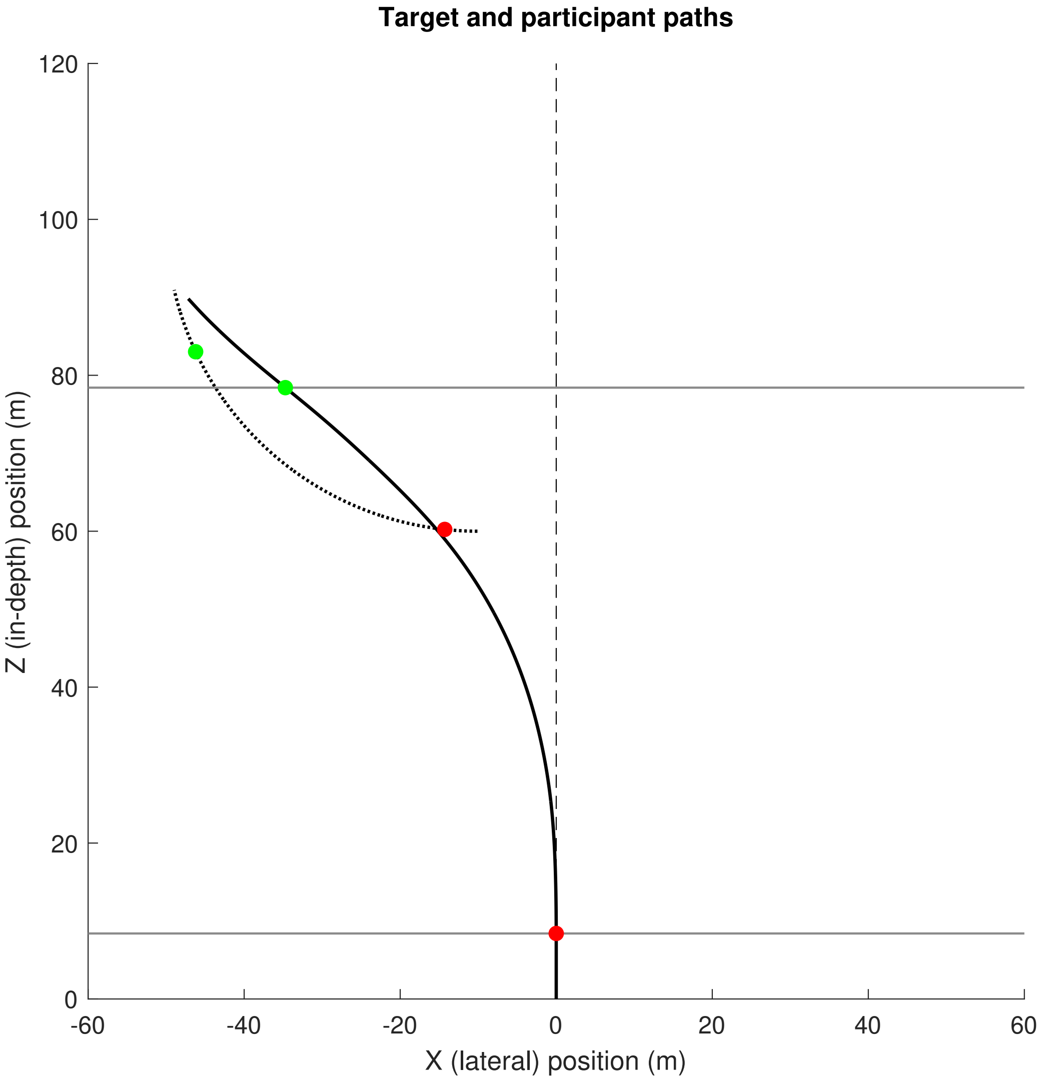

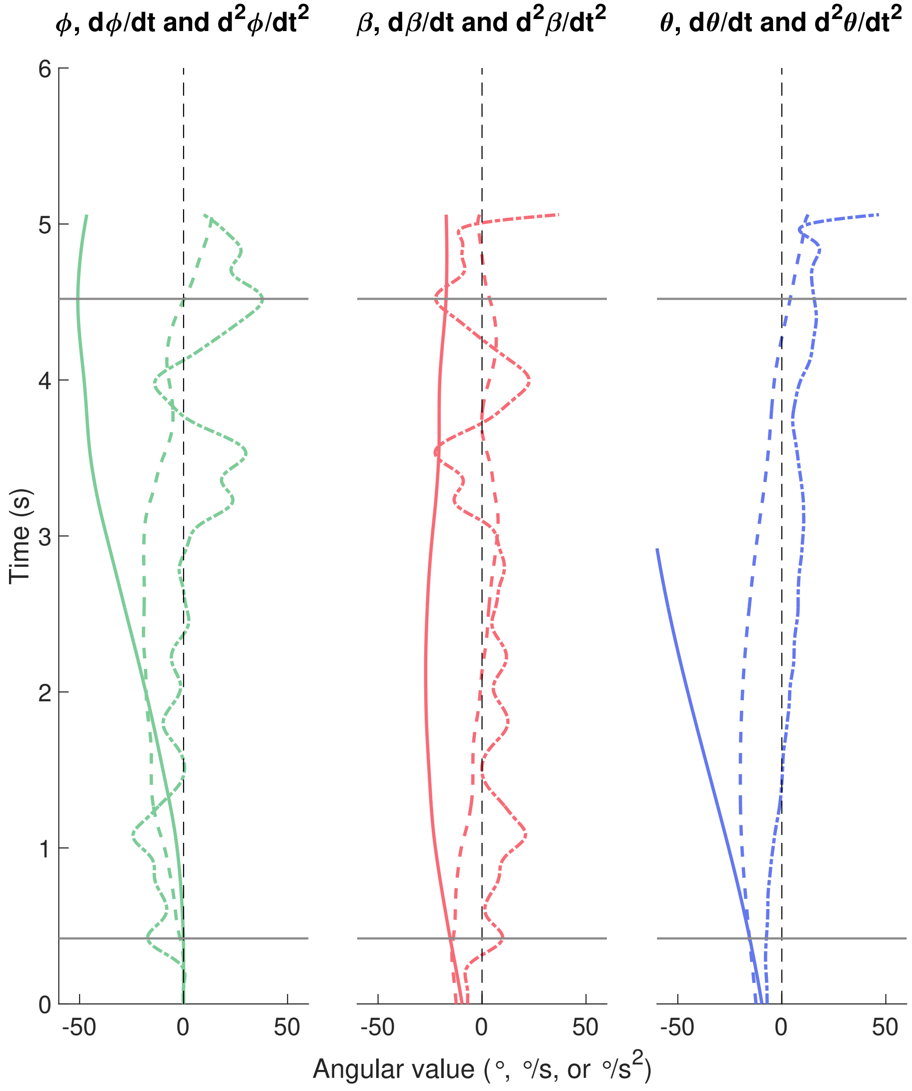

P13/B4
S0/R20-OUT

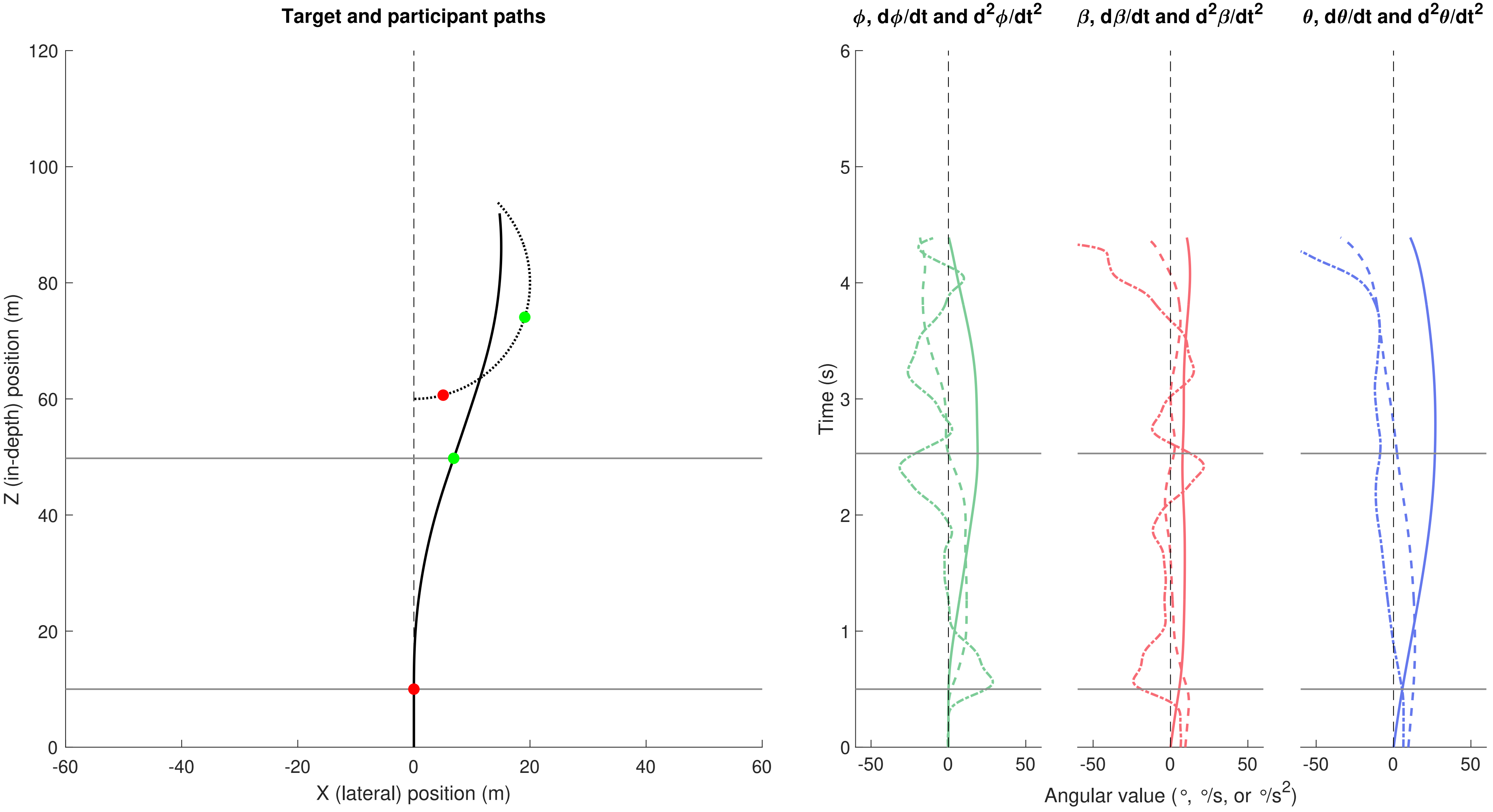

P13/B4
S0/R20-OUT

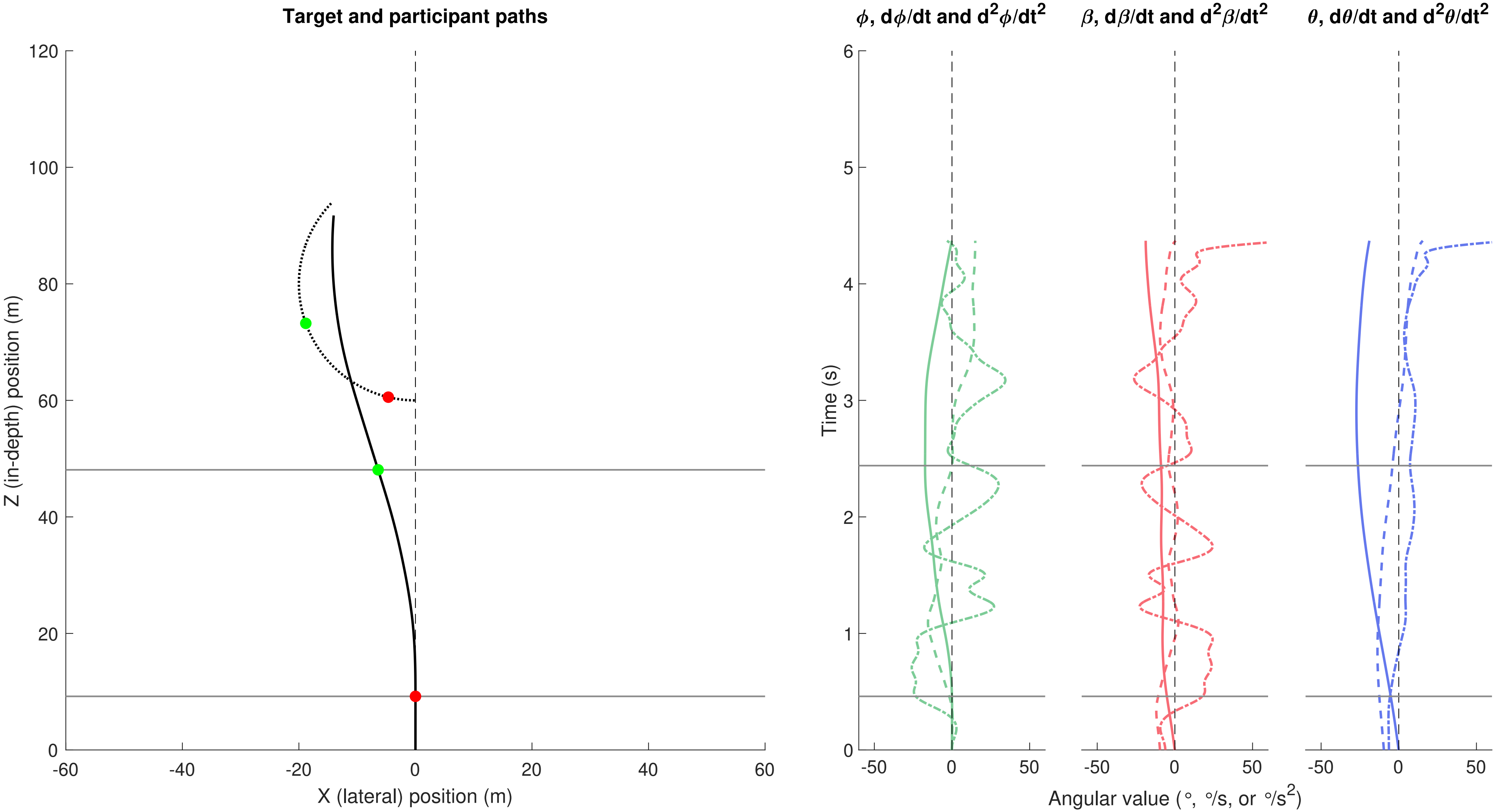

P13/B4
S0/R40-OUT

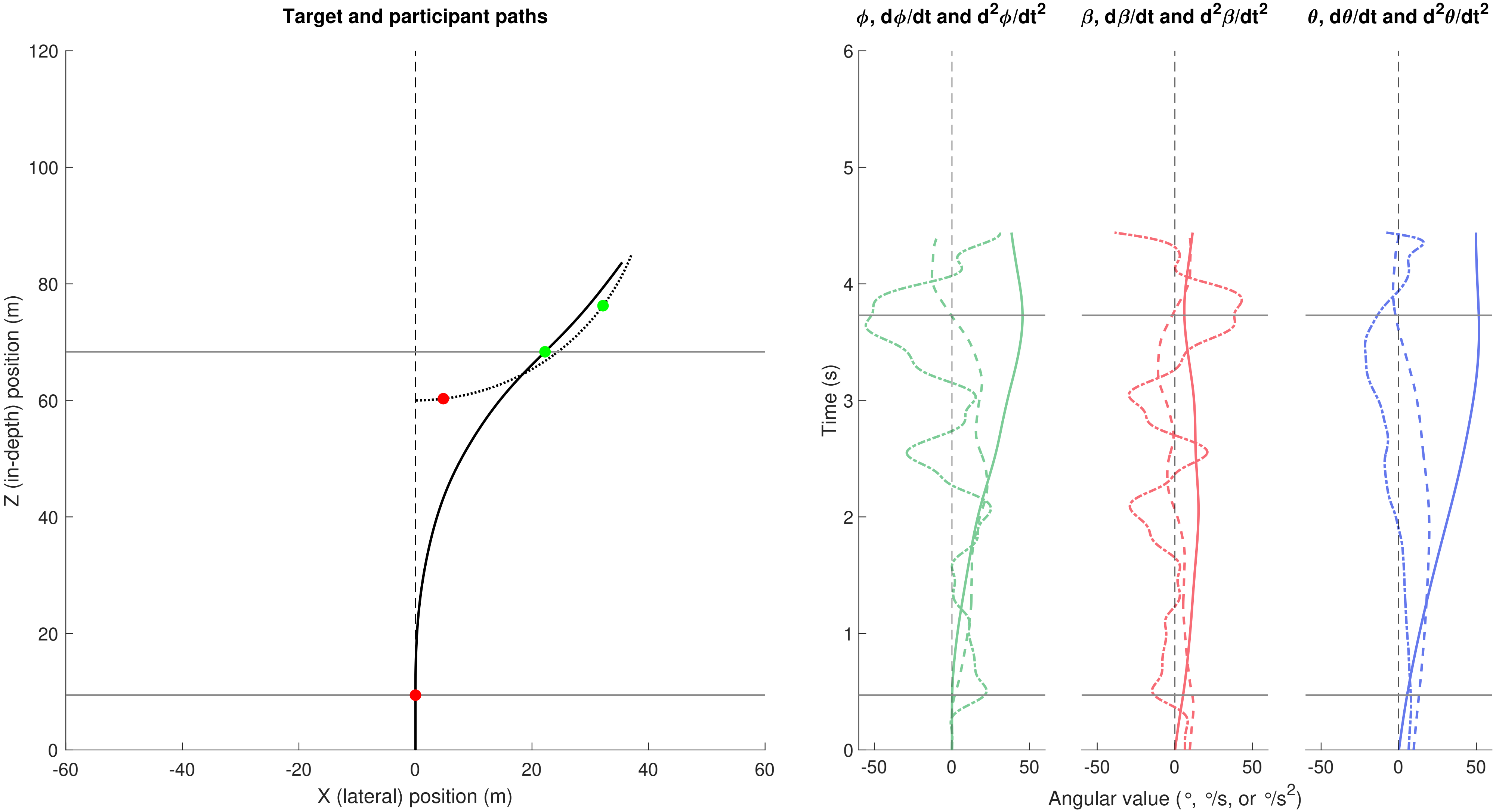

P13/B4
S0/R40-OUT

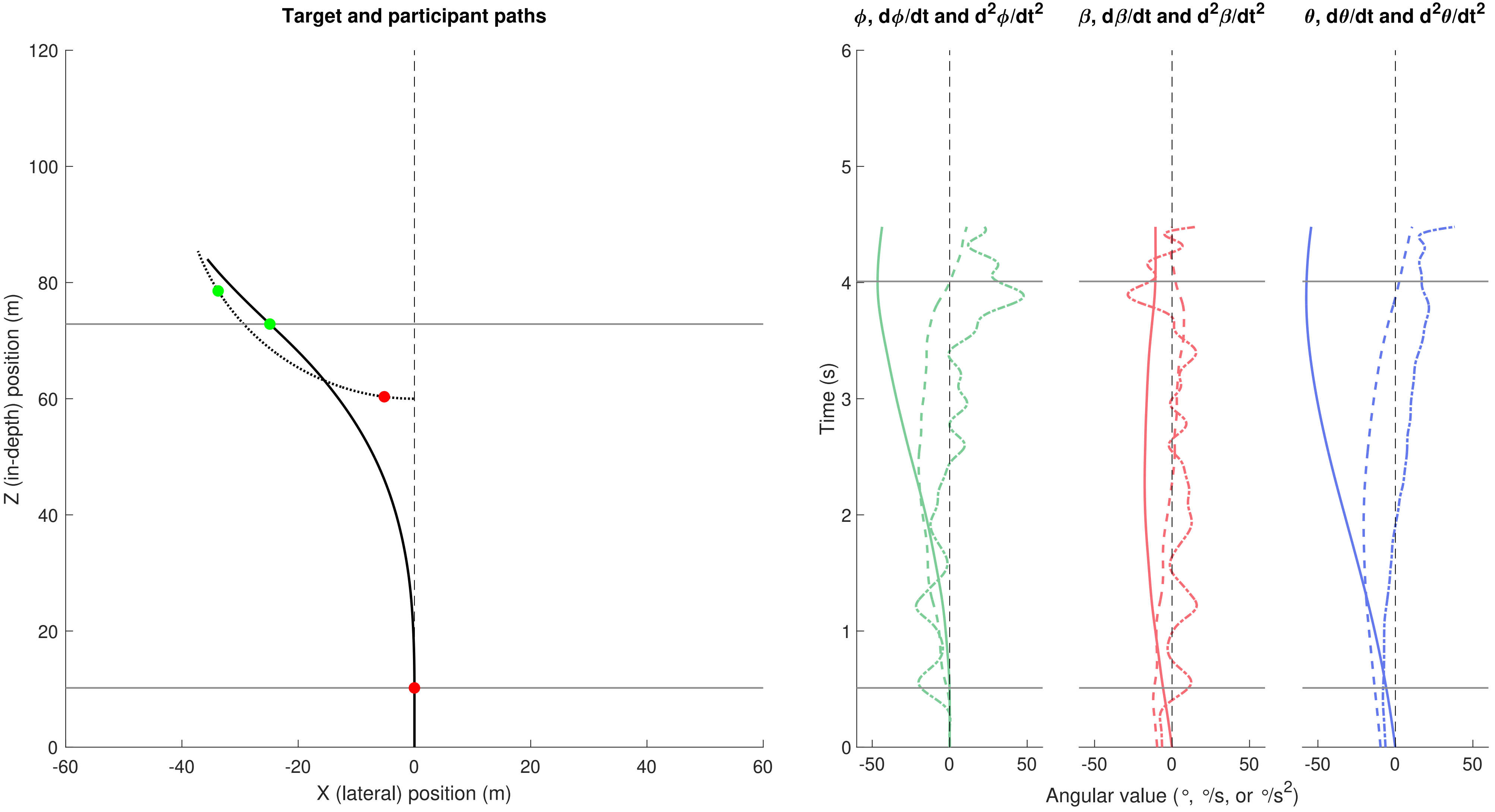

P13/B4
S10/R20-OUT

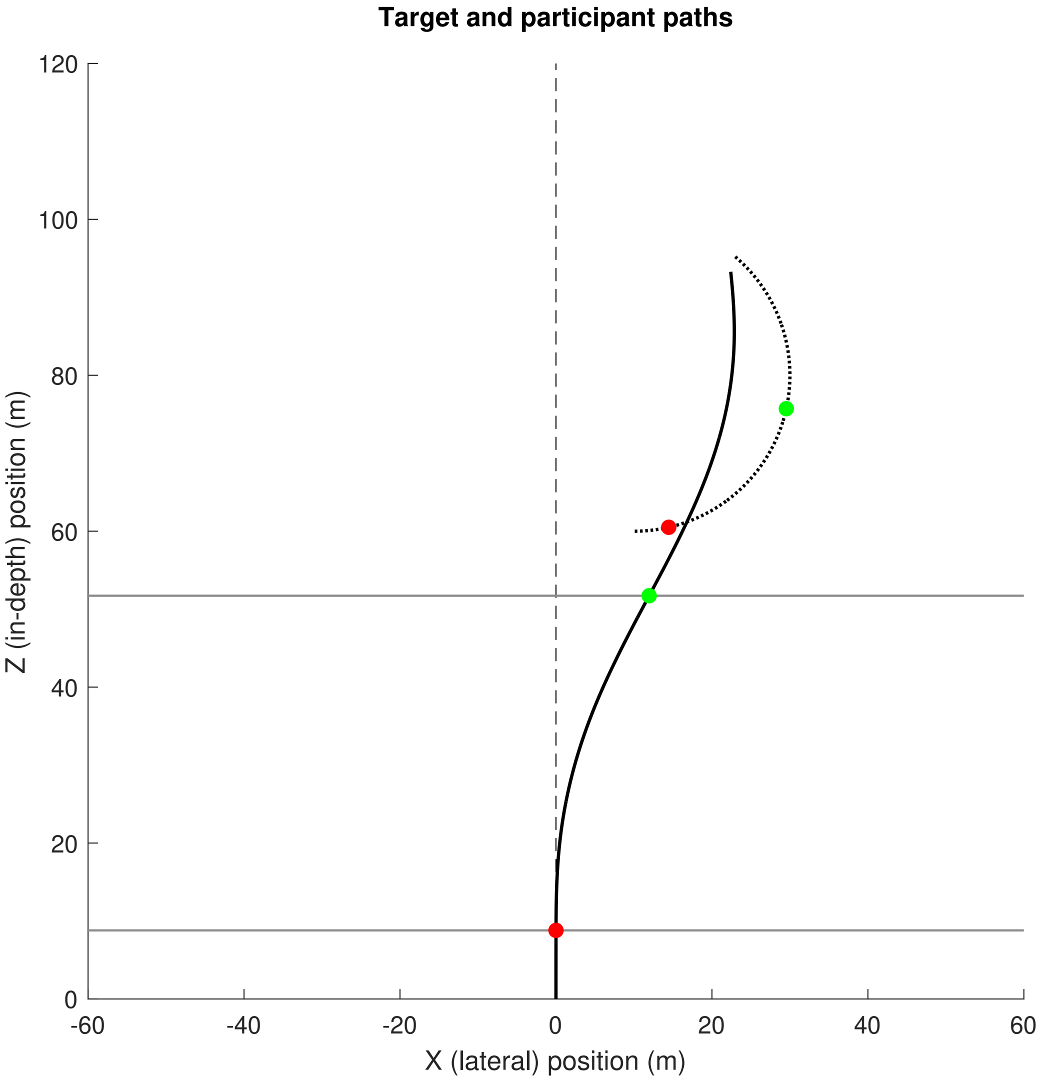

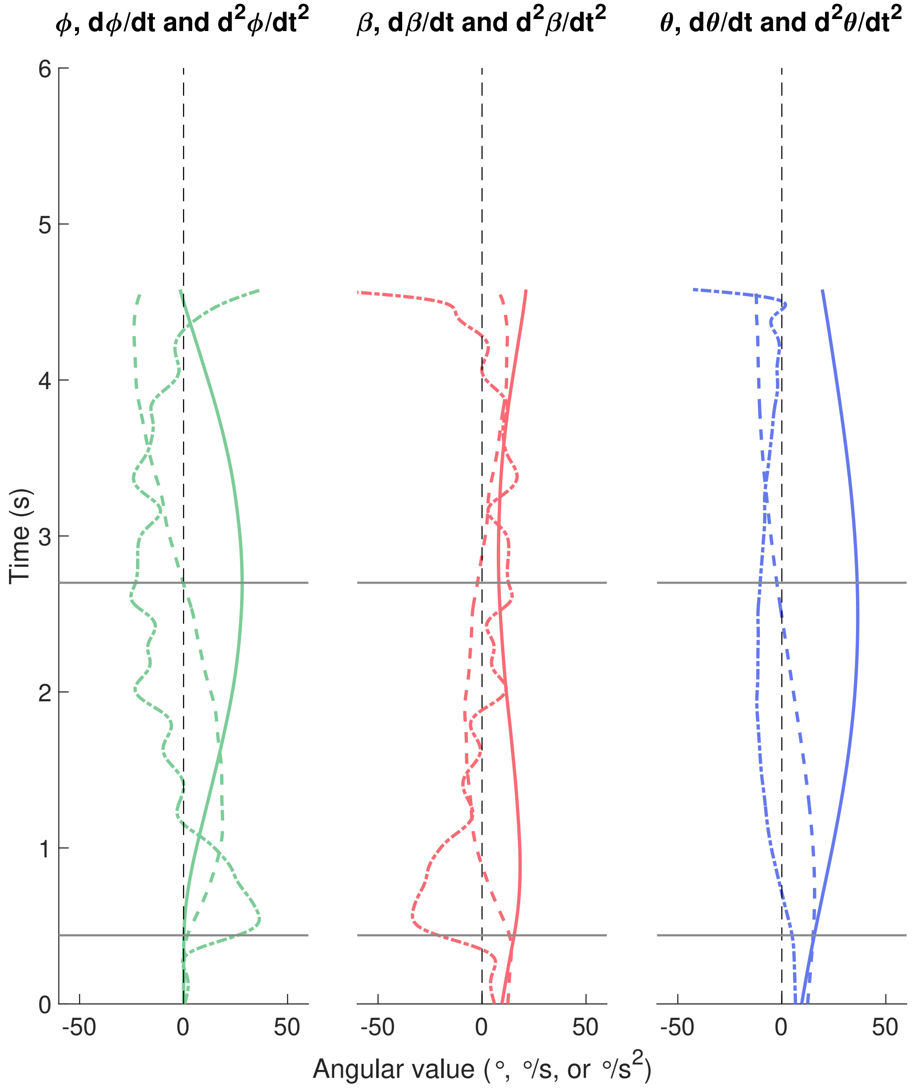

P13/B4
S10/R20-IN

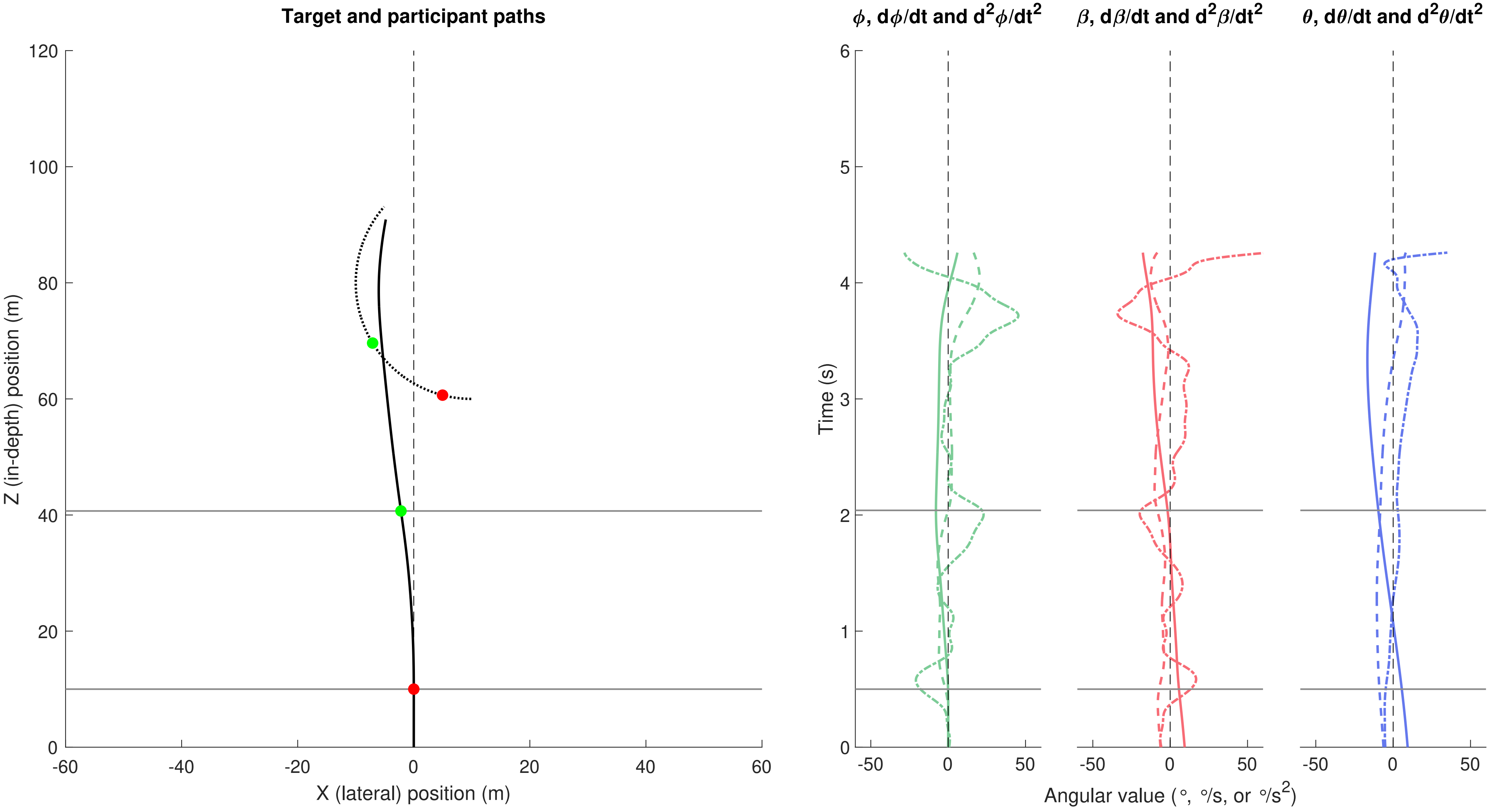

P13/B4
S10/R40-OUT

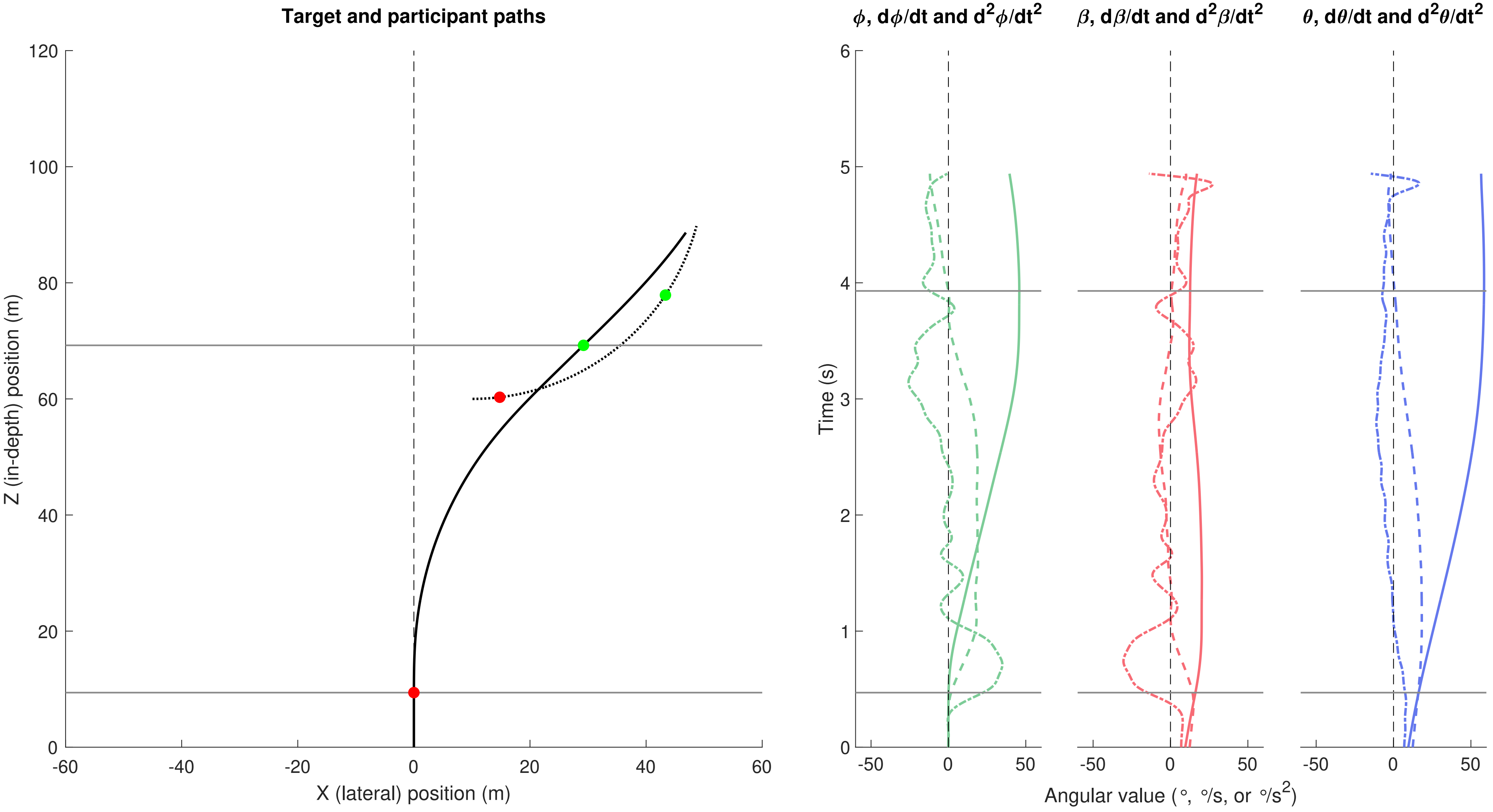

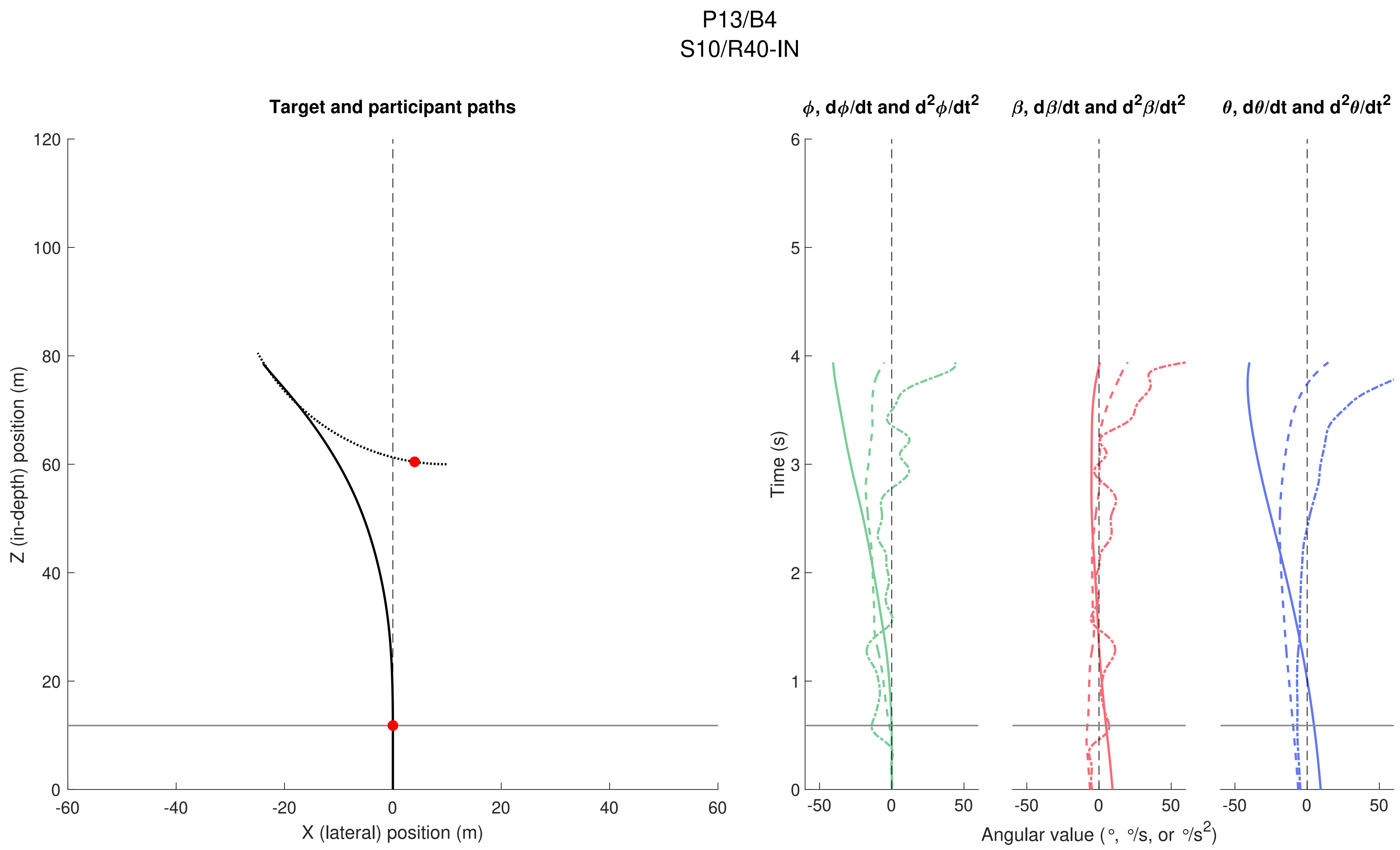
P13/B4
S10/R40-IN
Target and participant paths
Z (in-depth) position (m)
X (lateral) position (m)
120
100
80
60
40
20
0
-60
-40
-20
0
20
40
60
ϕ, dϕ/dt and d2ϕ/dt2
β, dβ/dt and d2β/dt2
θ, dθ/dt and d2θ/dt2
Time (s)
6
5
4
3
2
1
0
-50
0
50
Angular value (°, °/s, or °/s2)

P13/B4
S20/R20-OUT

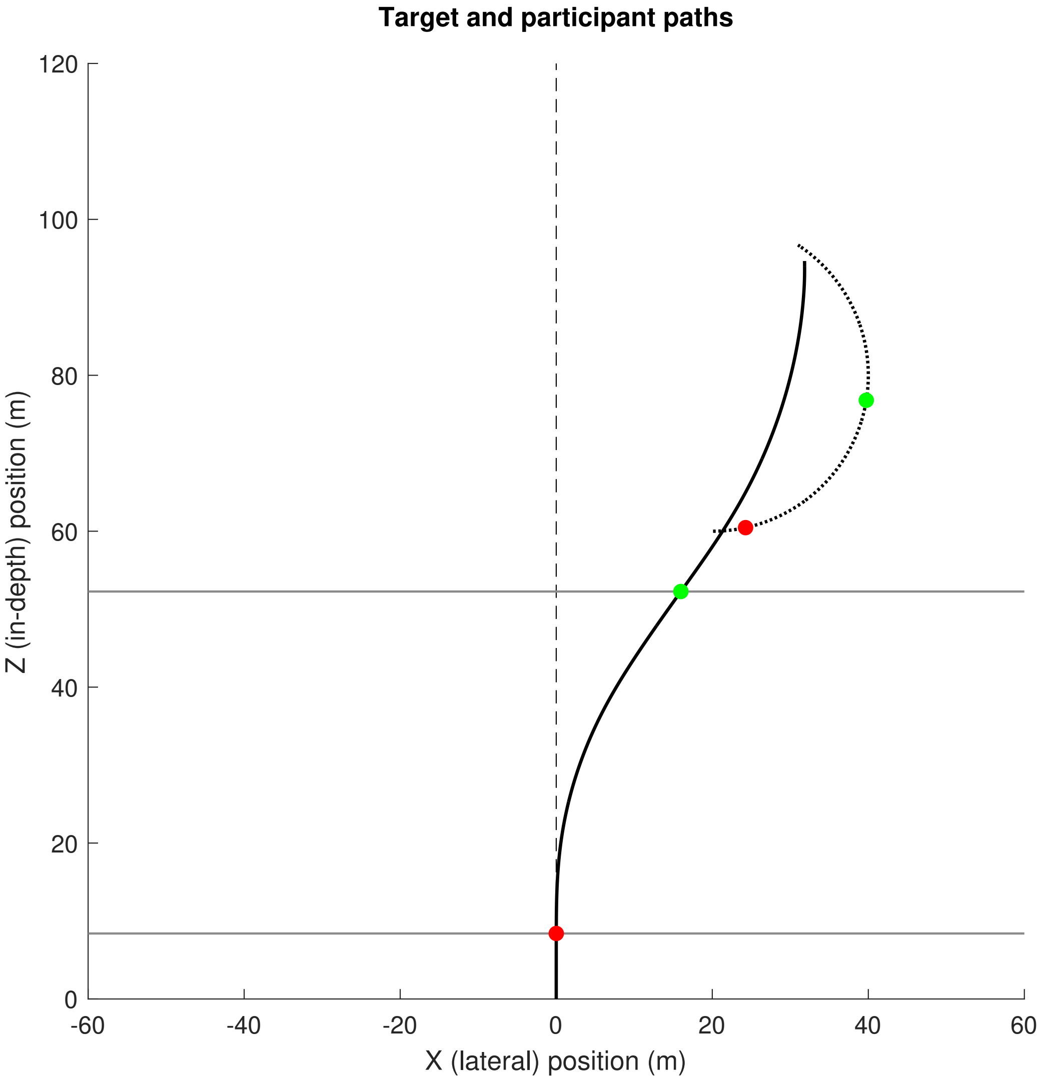

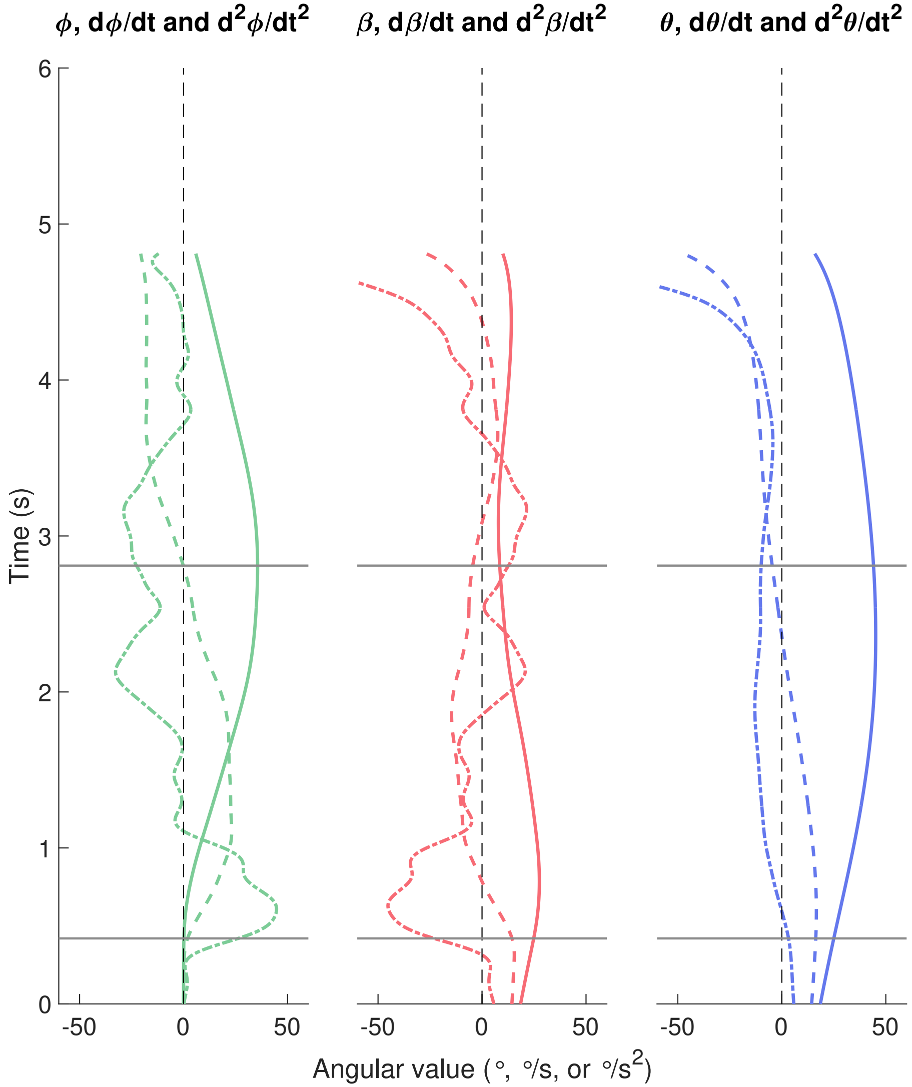

P13/B4
S20/R20-IN

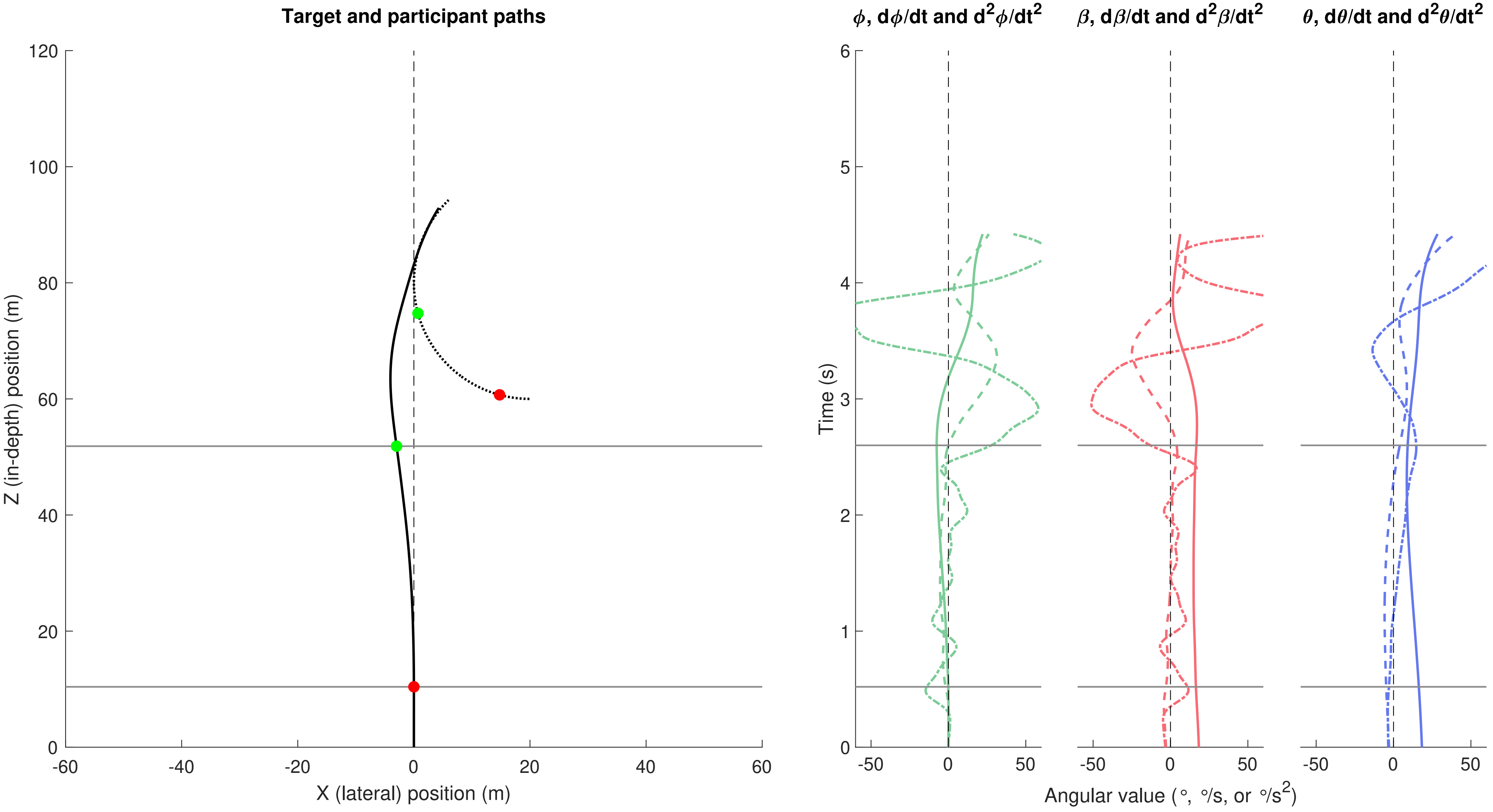

P13/B4
S20/R40-OUT

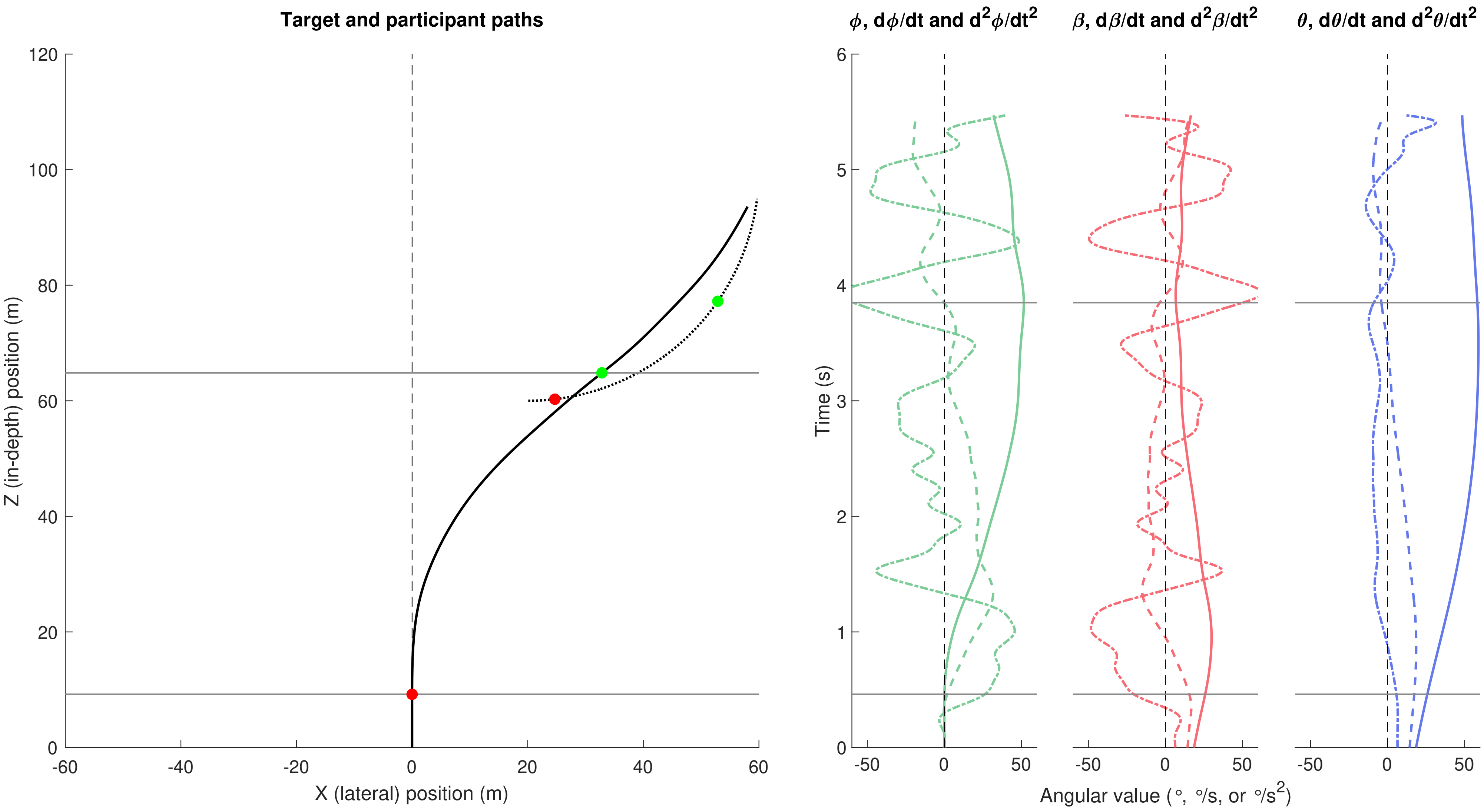

P13/B4
S20/R40-IN

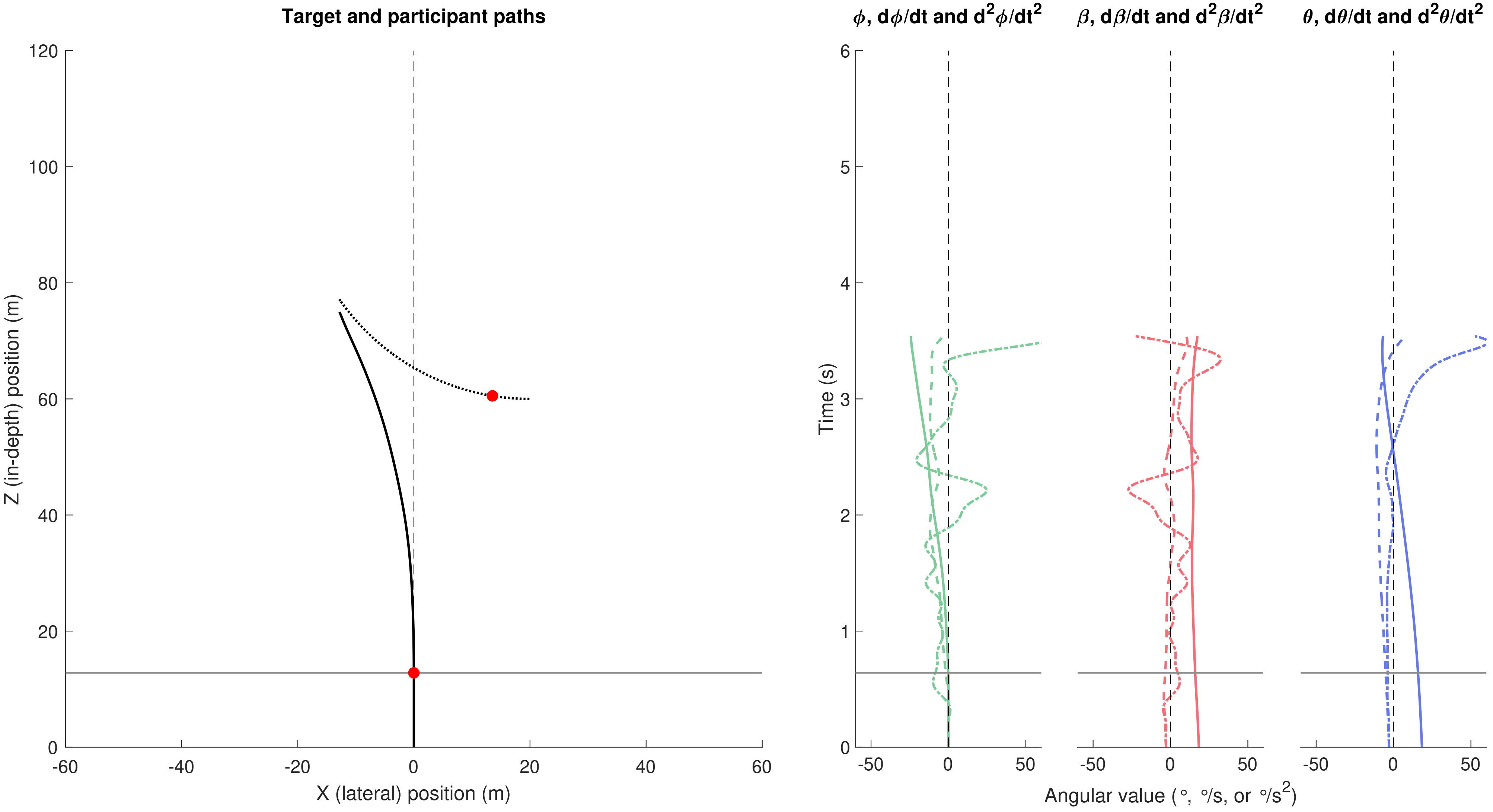

P13/B5
S20/R20-IN

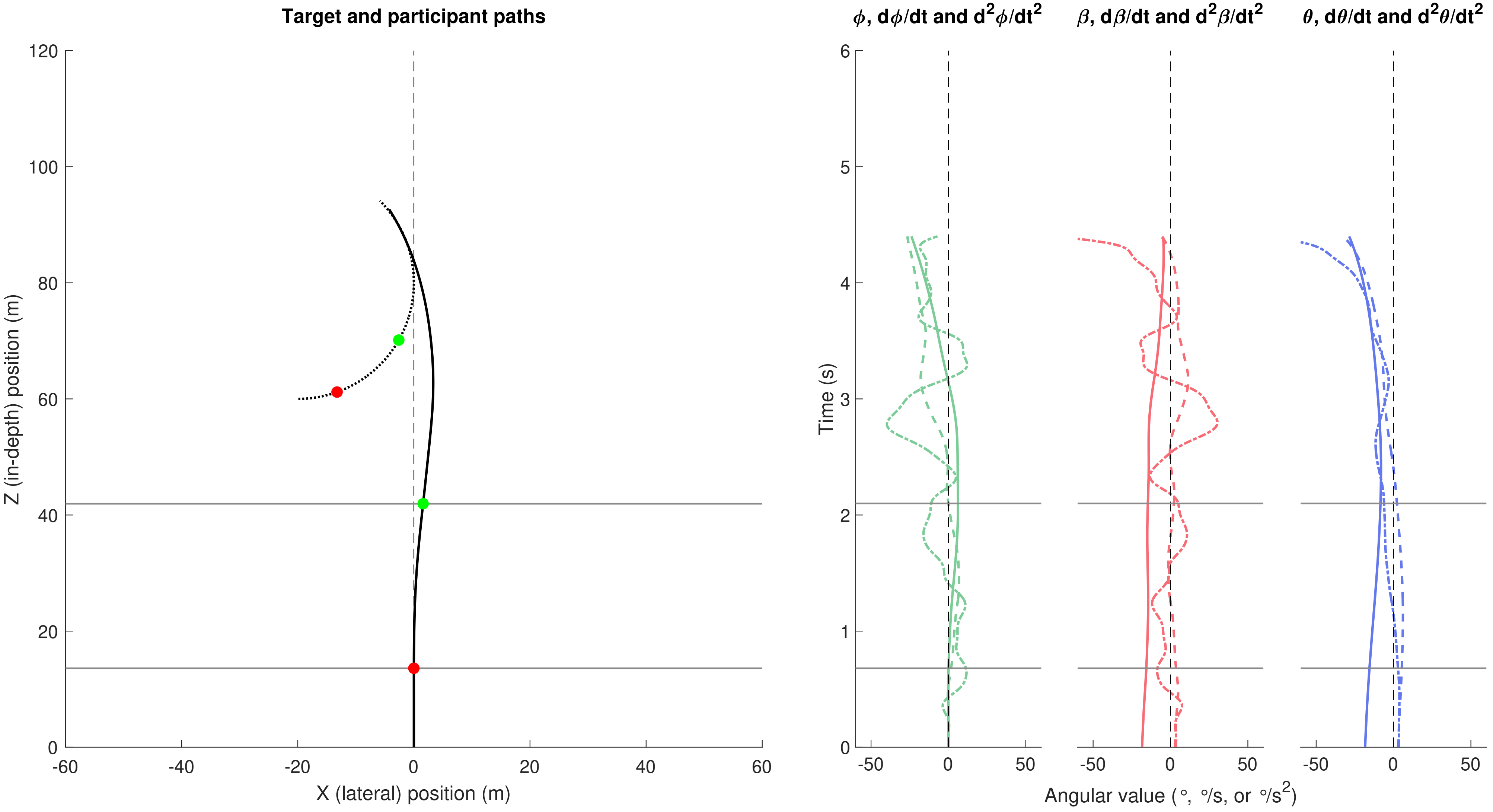

P13/B5
S20/R20-OUT

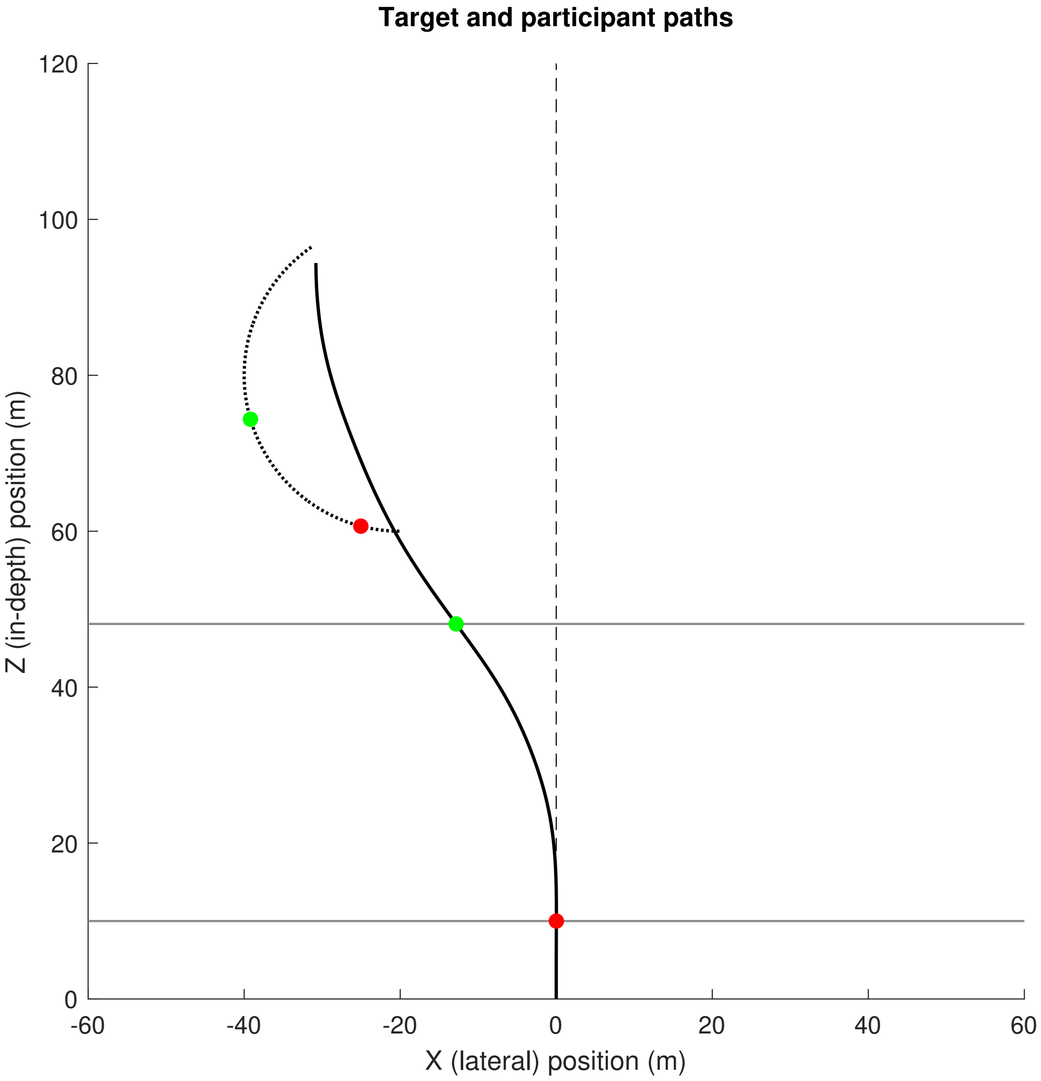

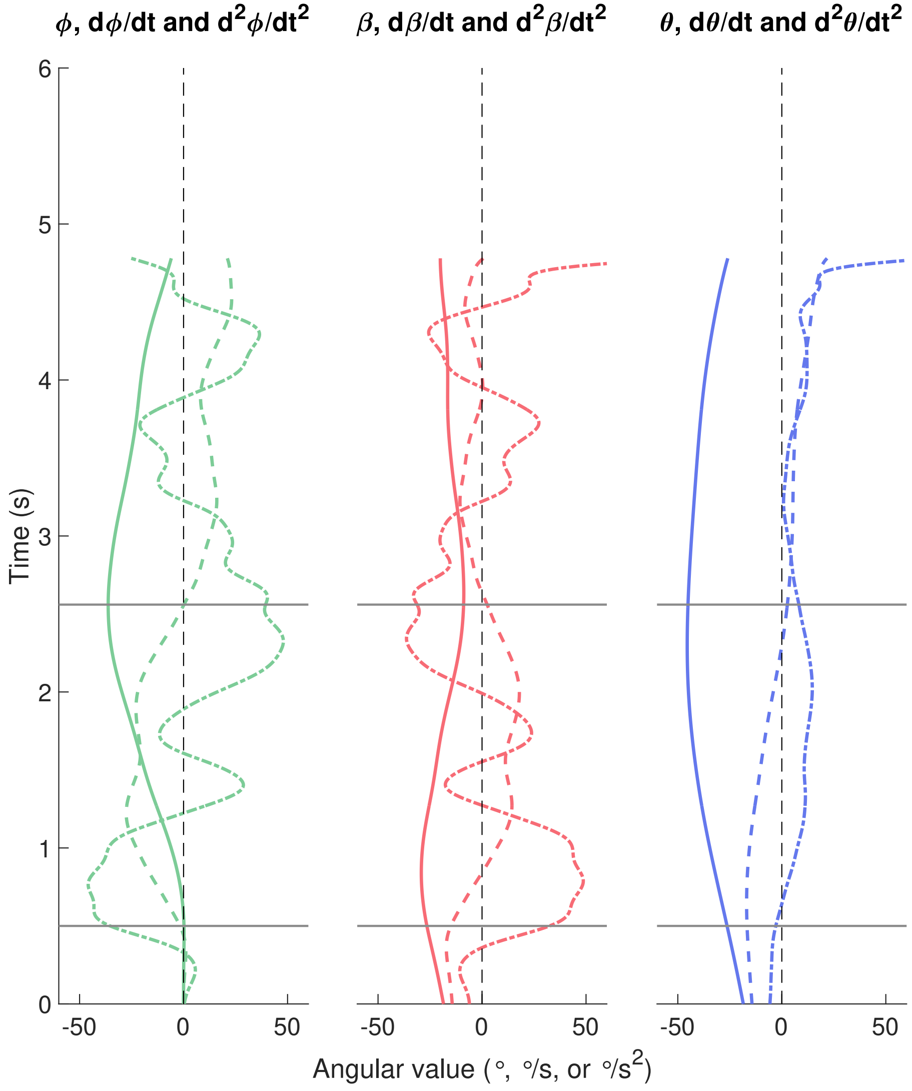

P13/B5
S20/R40-IN

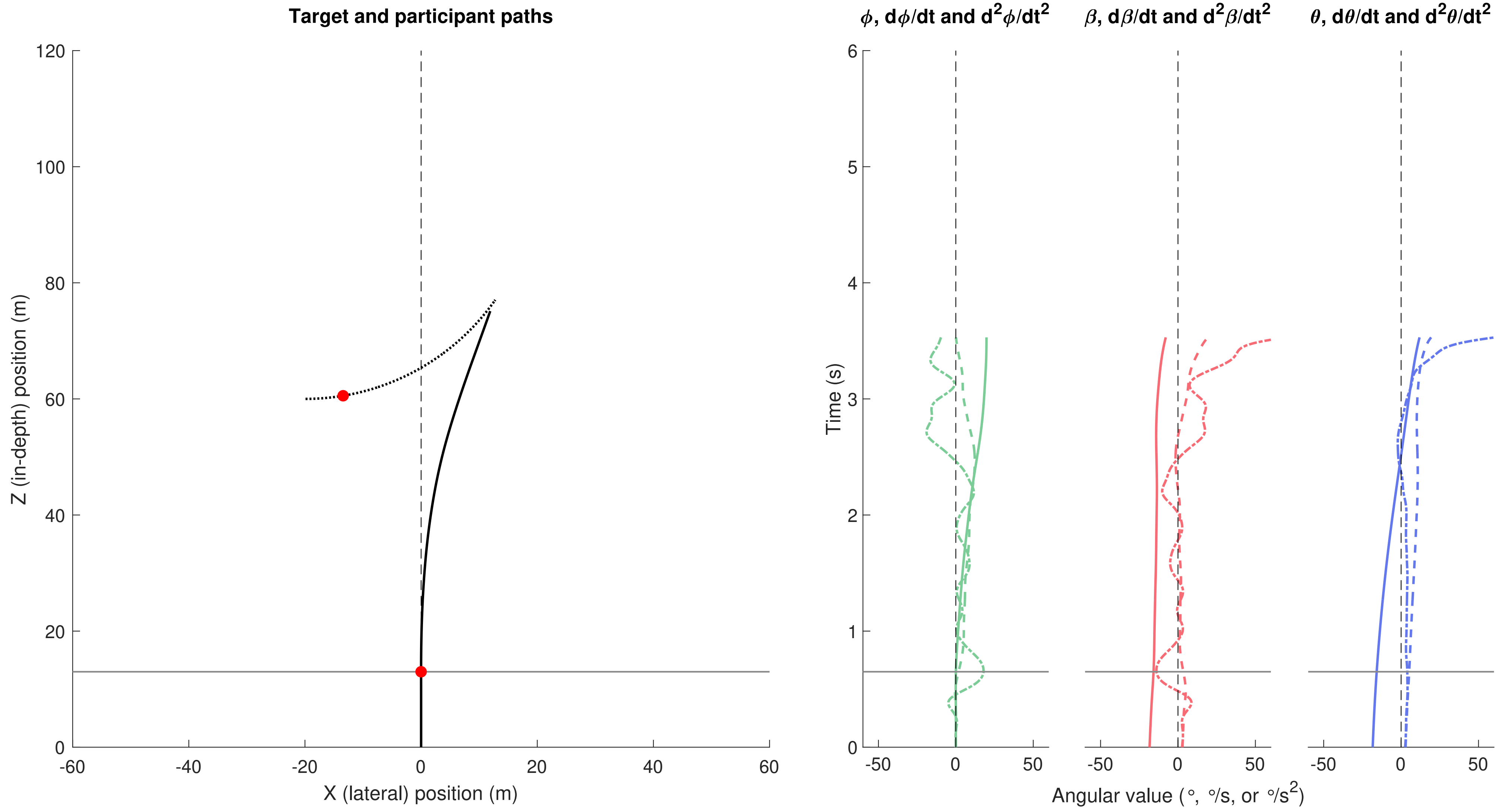

P13/B5
S20/R40-OUT

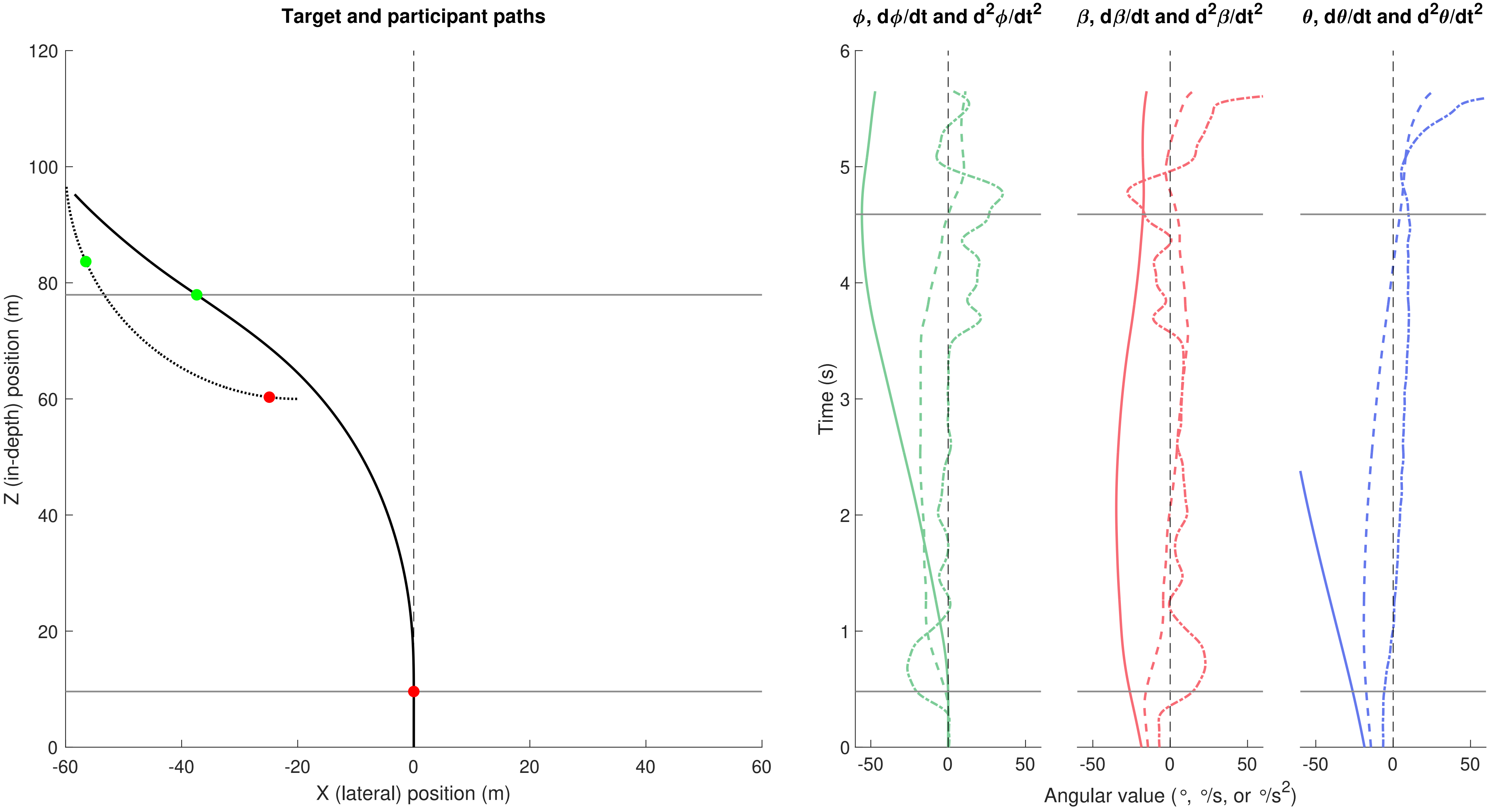

P13/B5
S10/R20-IN

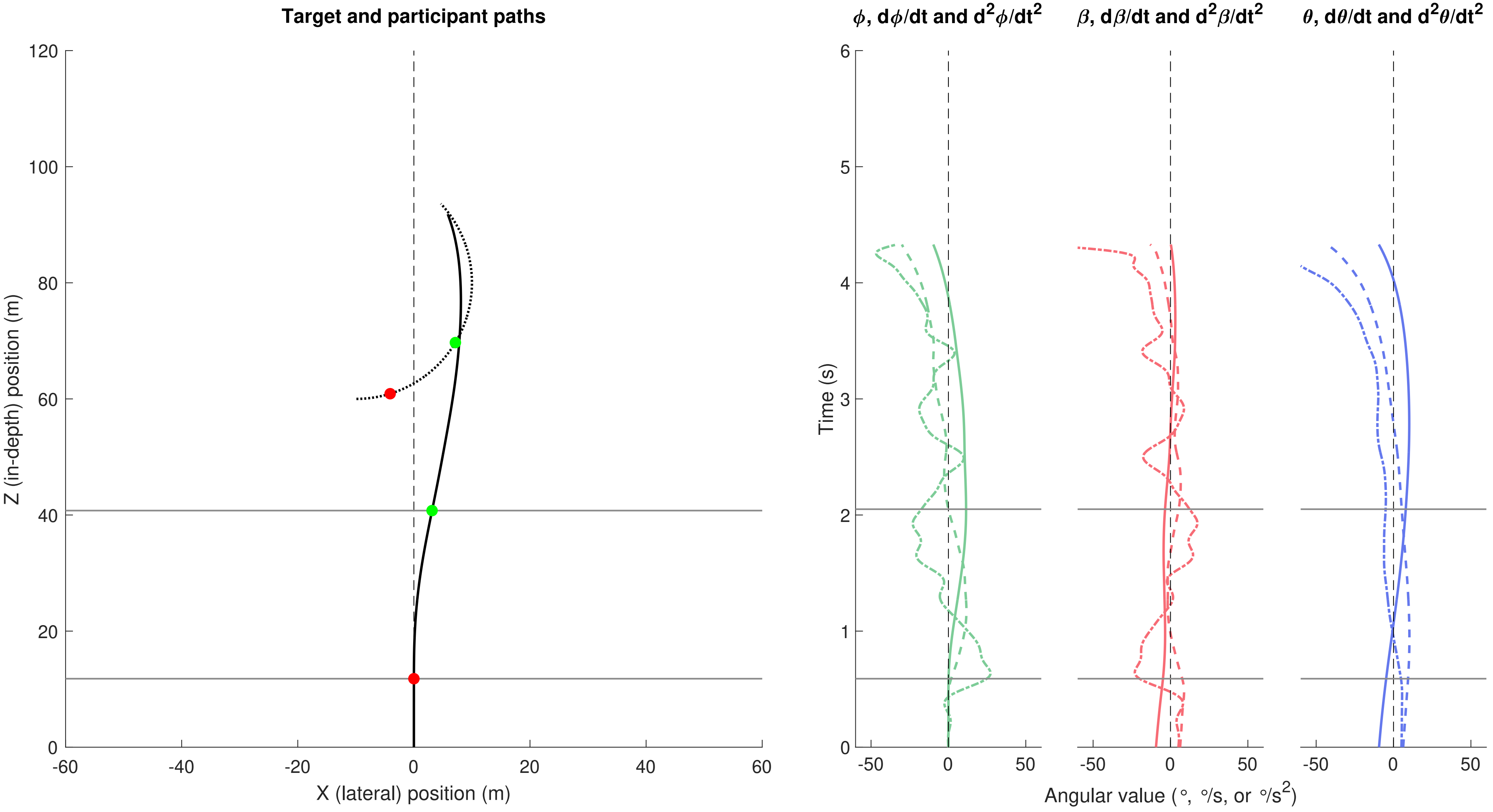

P13/B5
S10/R20-OUT

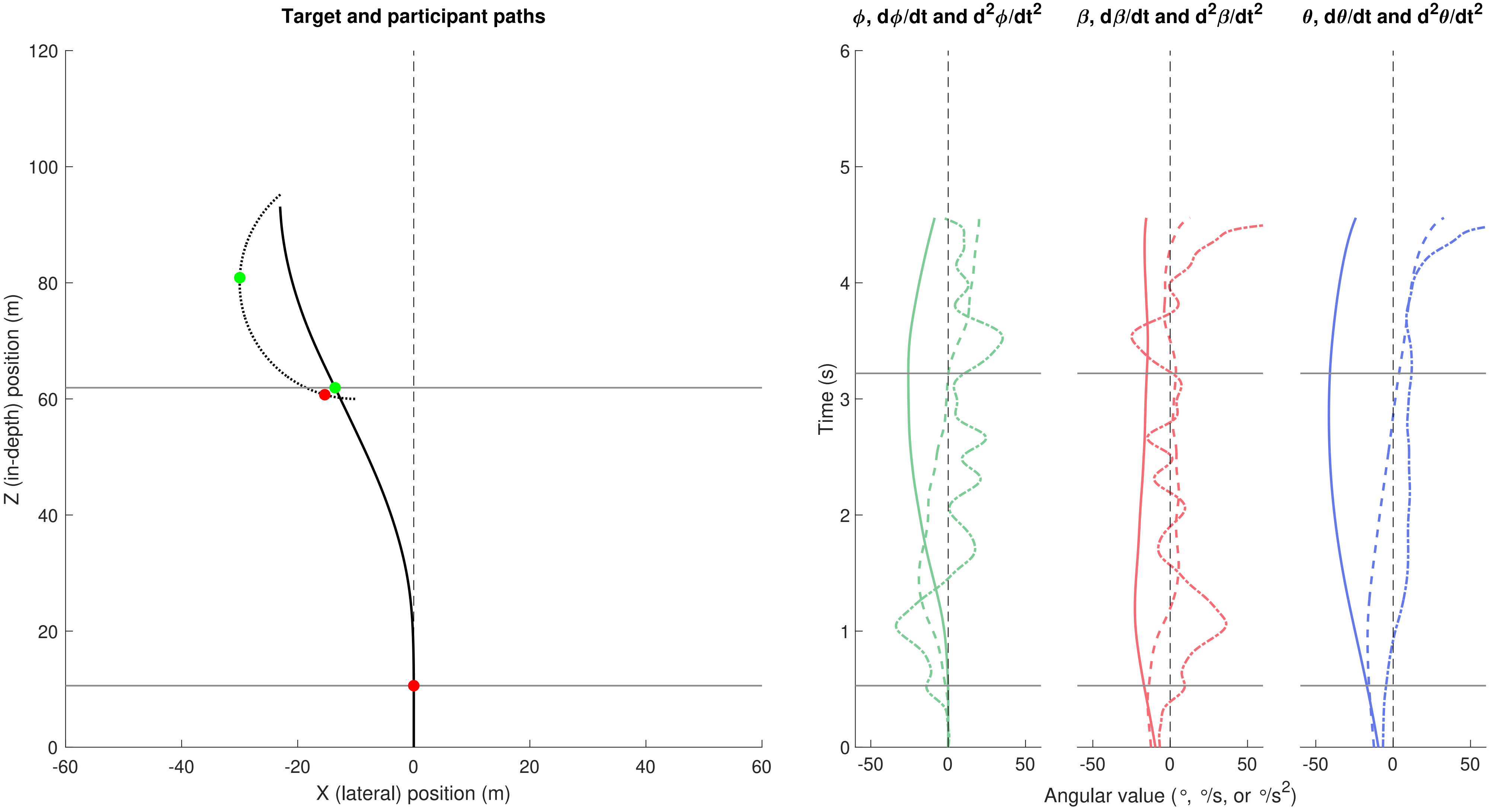

P13/B5
S10/R40-IN

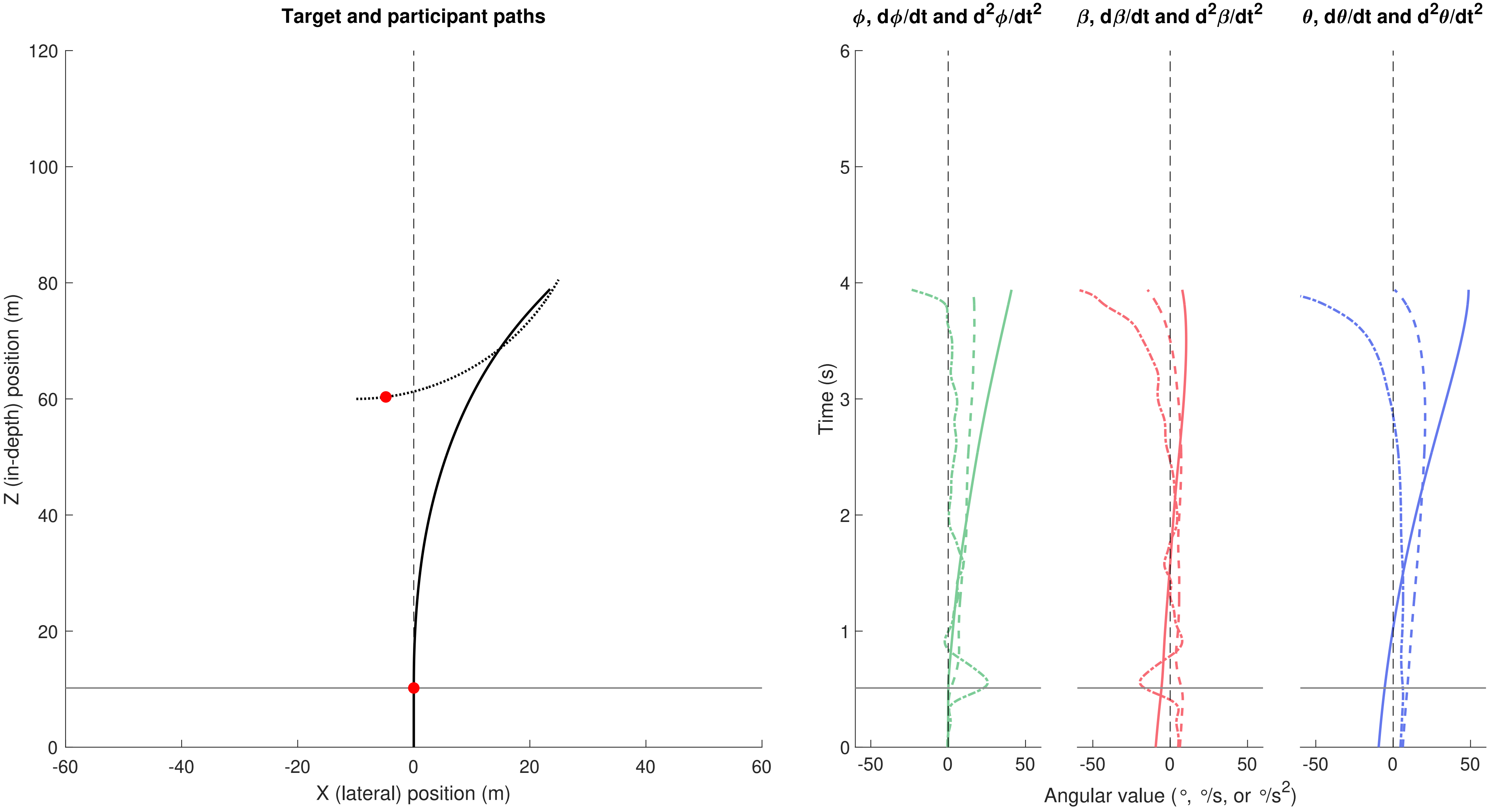

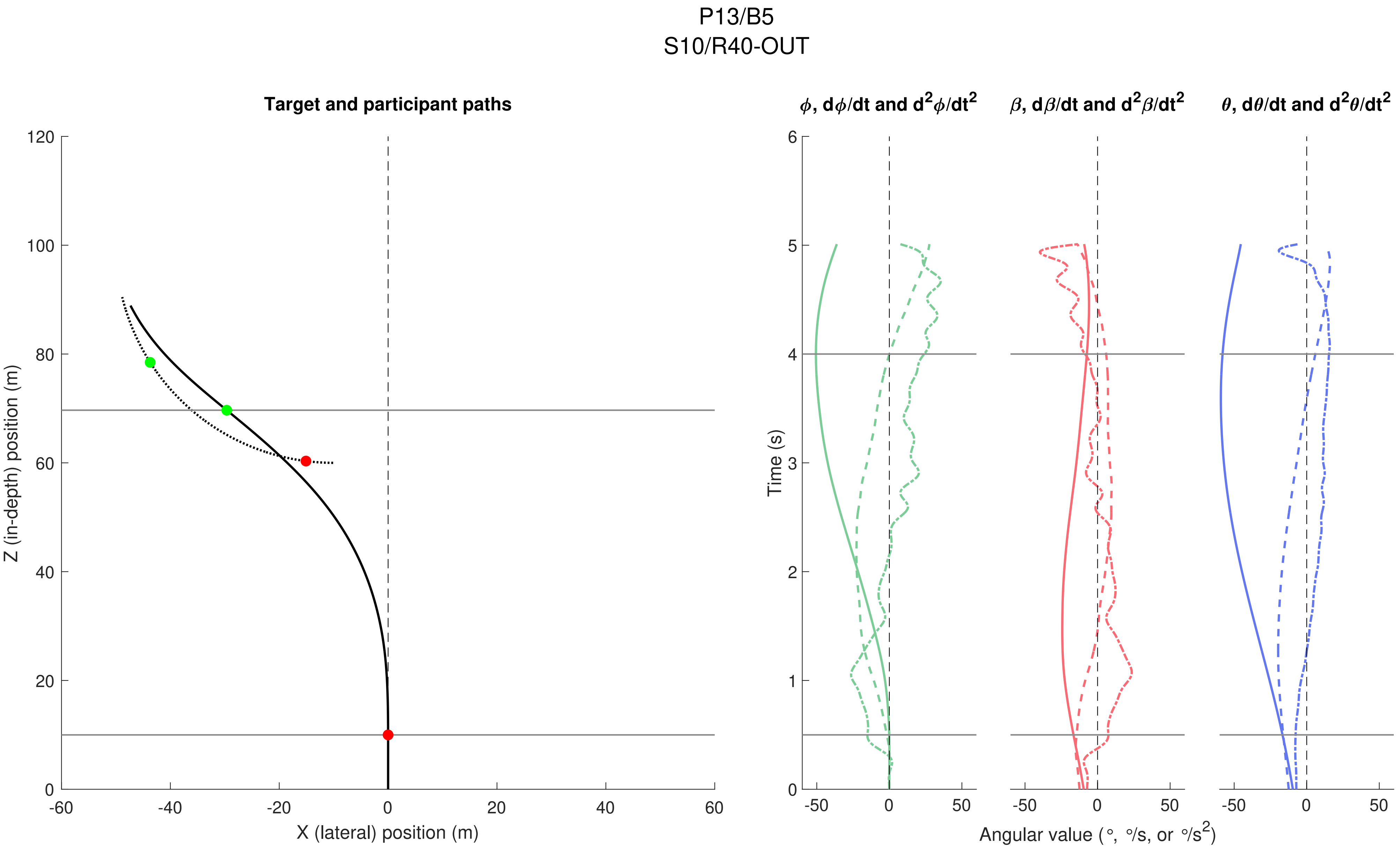
P13/B5
S10/R40-OUT
Target and participant paths
Z (in-depth) position (m)
X (lateral) position (m)
120
100
80
60
40
20
0
-60
-40
-20
0
20
40
60
ϕ, dϕ/dt and d$^2\phi$/dt^2
β, dβ/dt and d$^2\beta$/dt^2
θ, dθ/dt and d$^2\theta$/dt^2
Time (s)
6
5
4
3
2
1
0
-50
0
50
Angular value (°, °/s, or °/s^2)

P13/B5
S0/R20-OUT

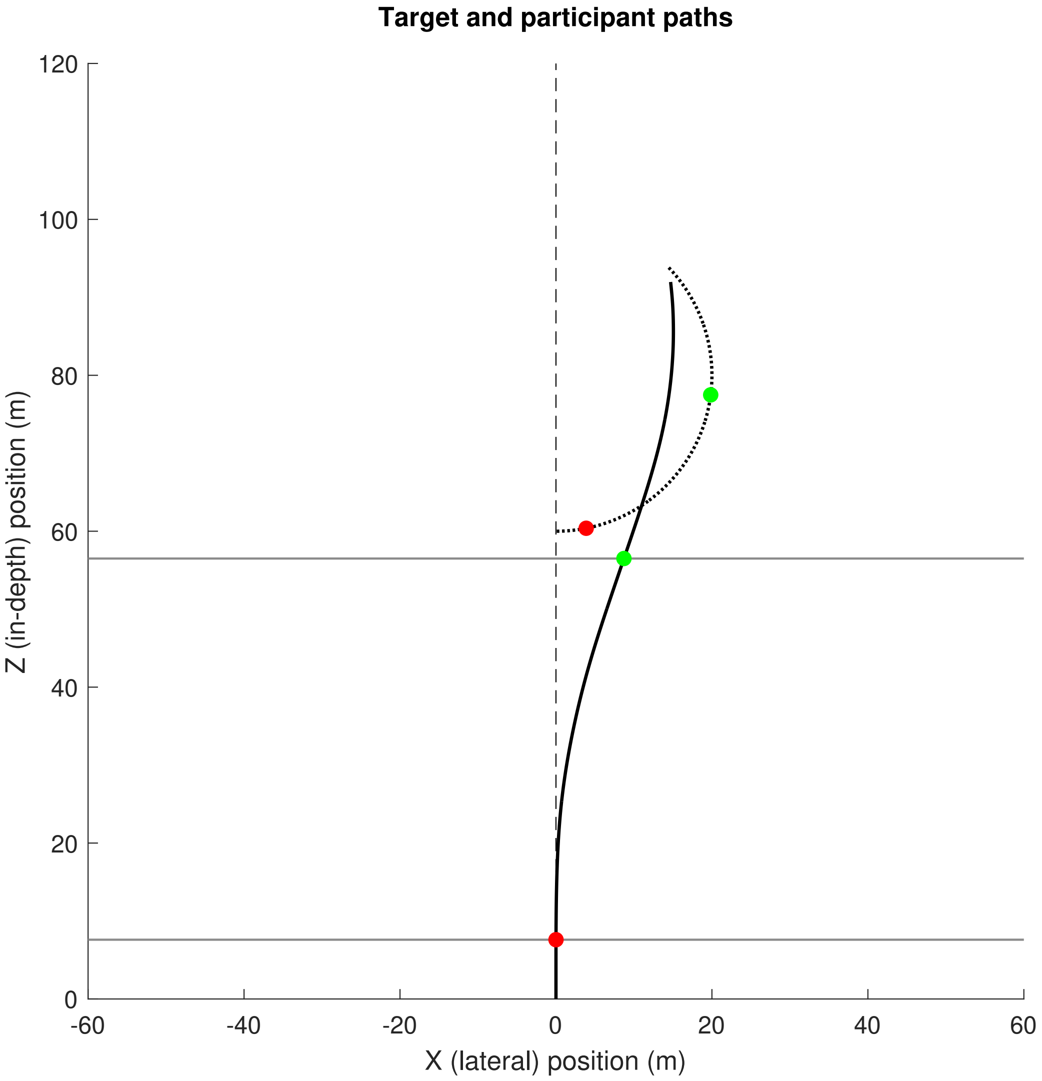

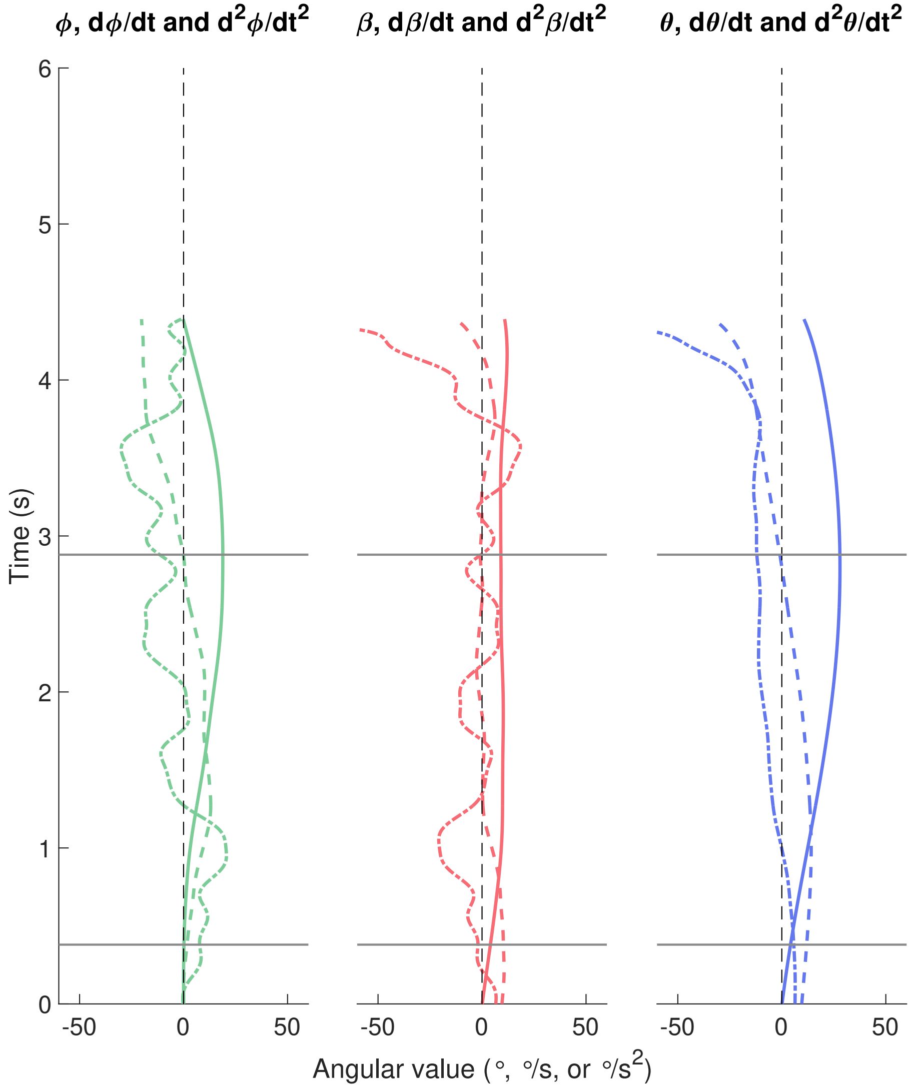

P13/B5
S0/R20-OUT

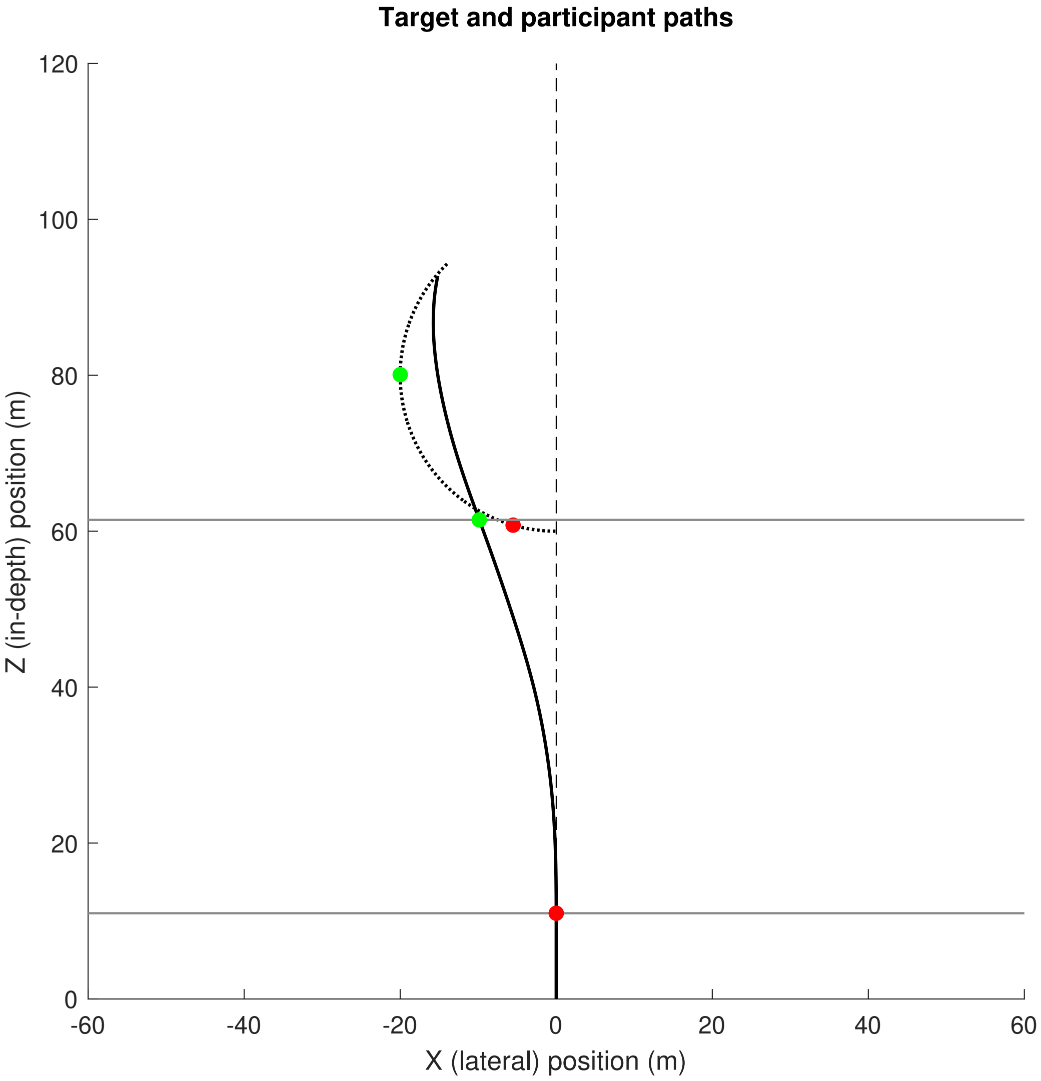

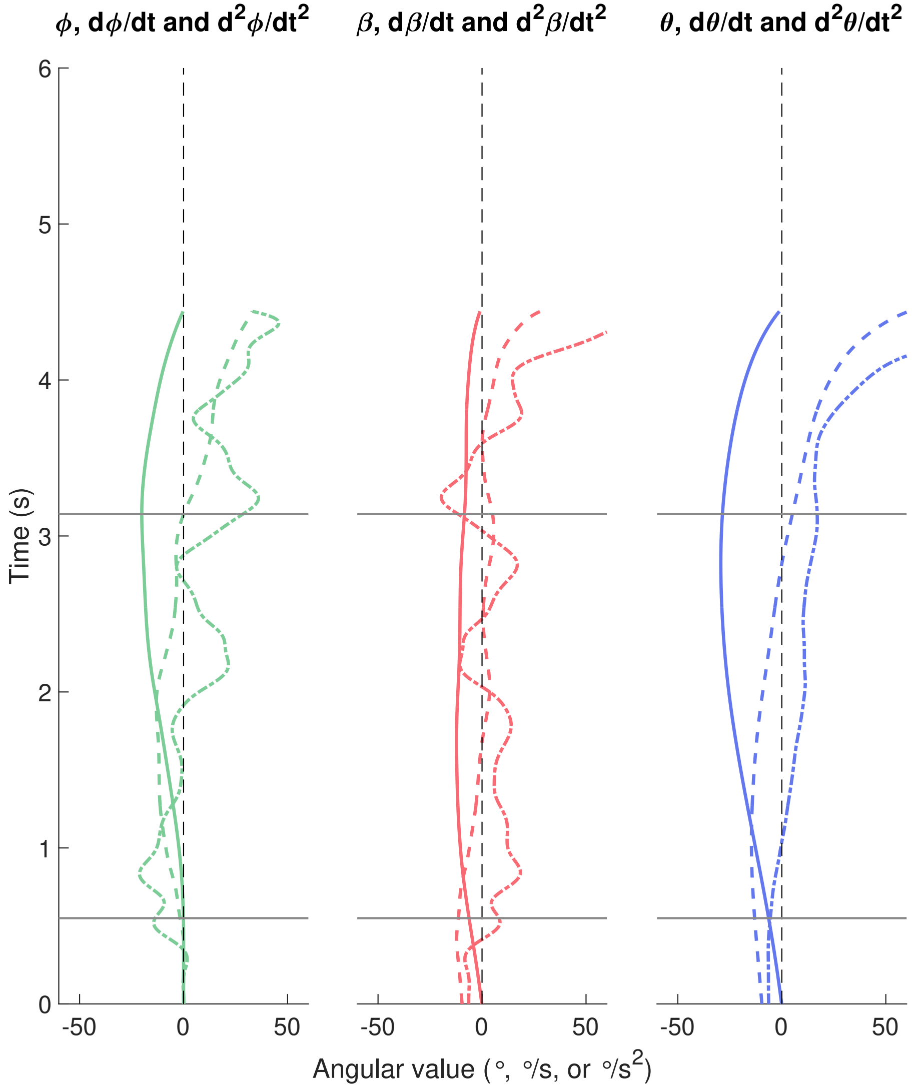

P13/B5
S0/R40-OUT

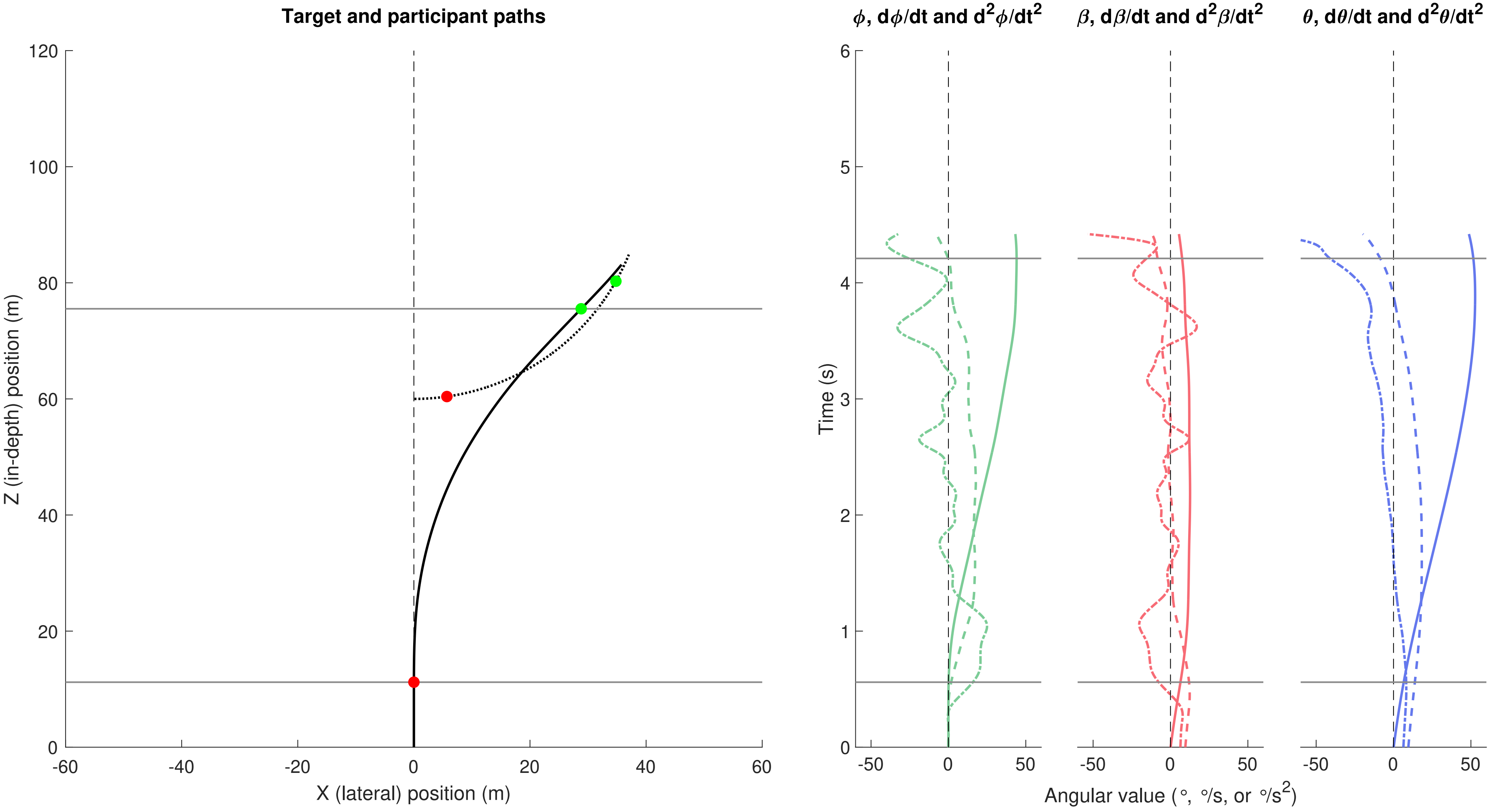

P13/B5
S0/R40-OUT

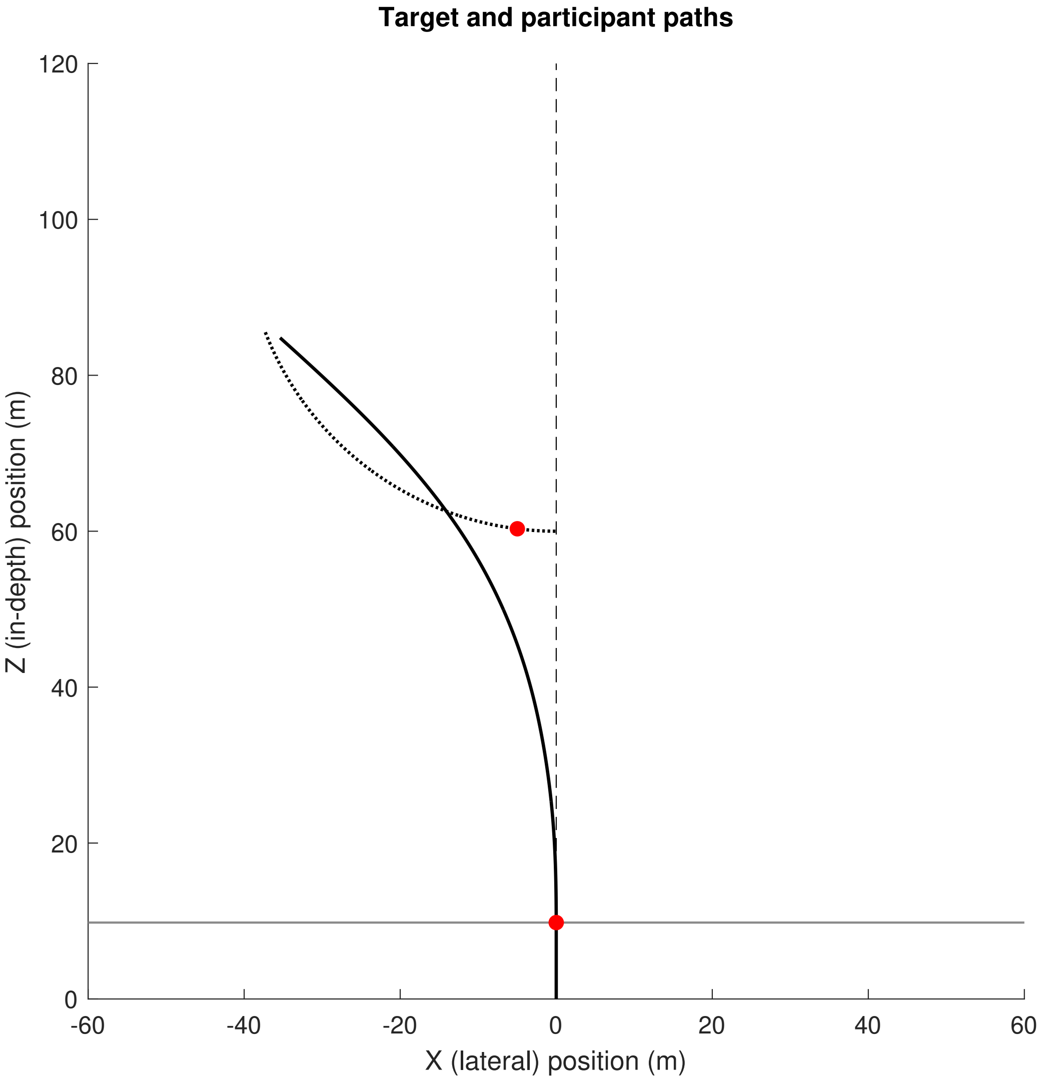

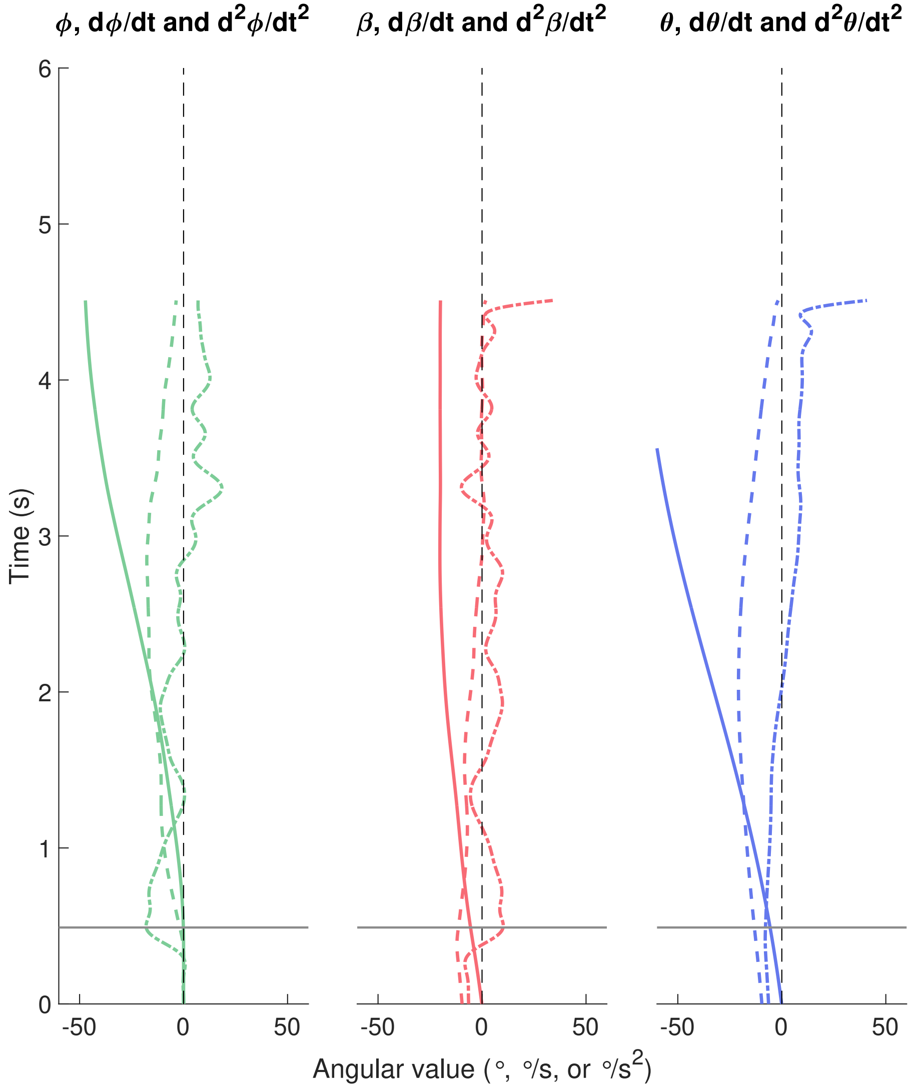

P13/B5
S10/R20-OUT

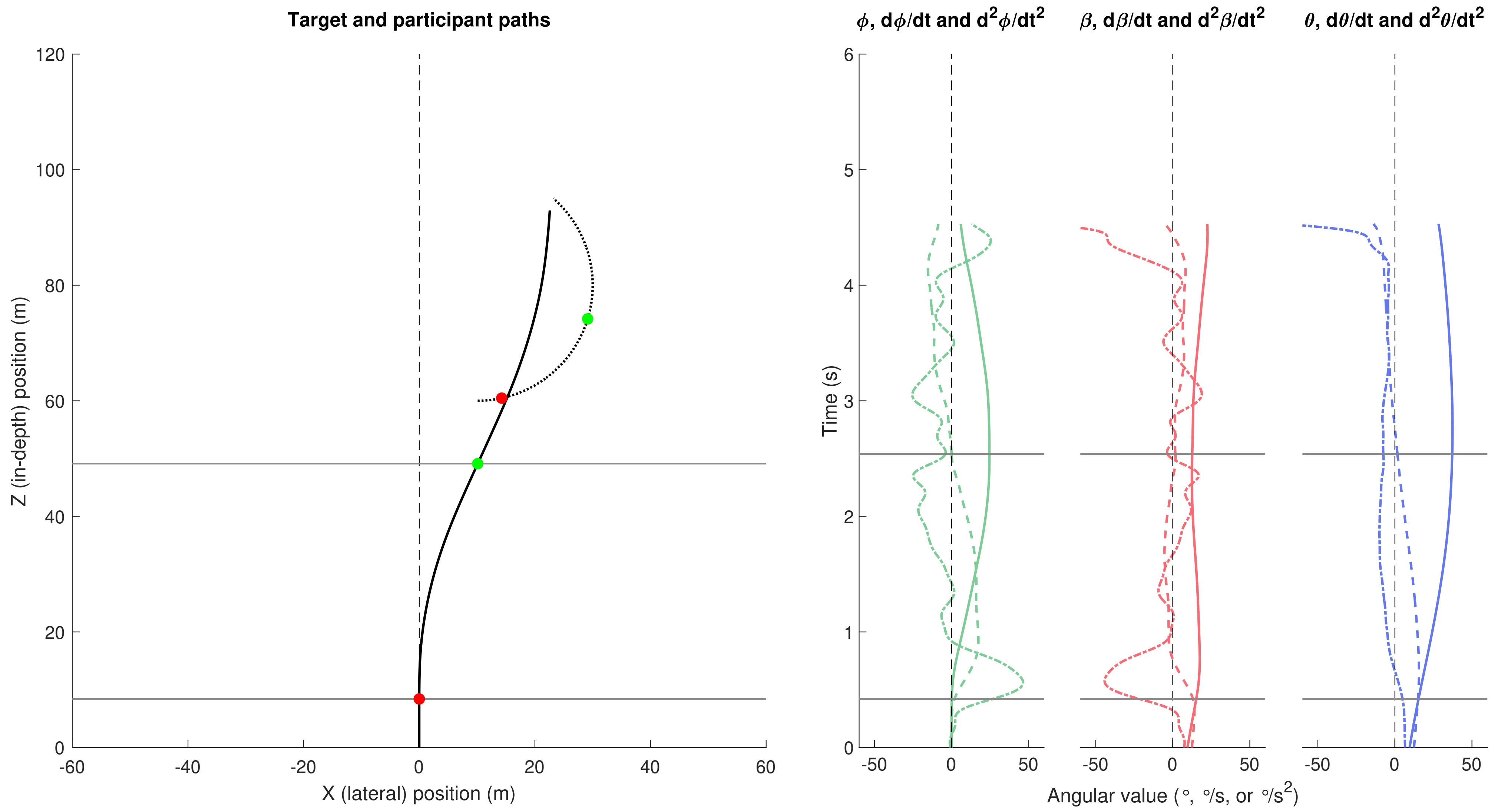

P13/B5
S10/R20-IN

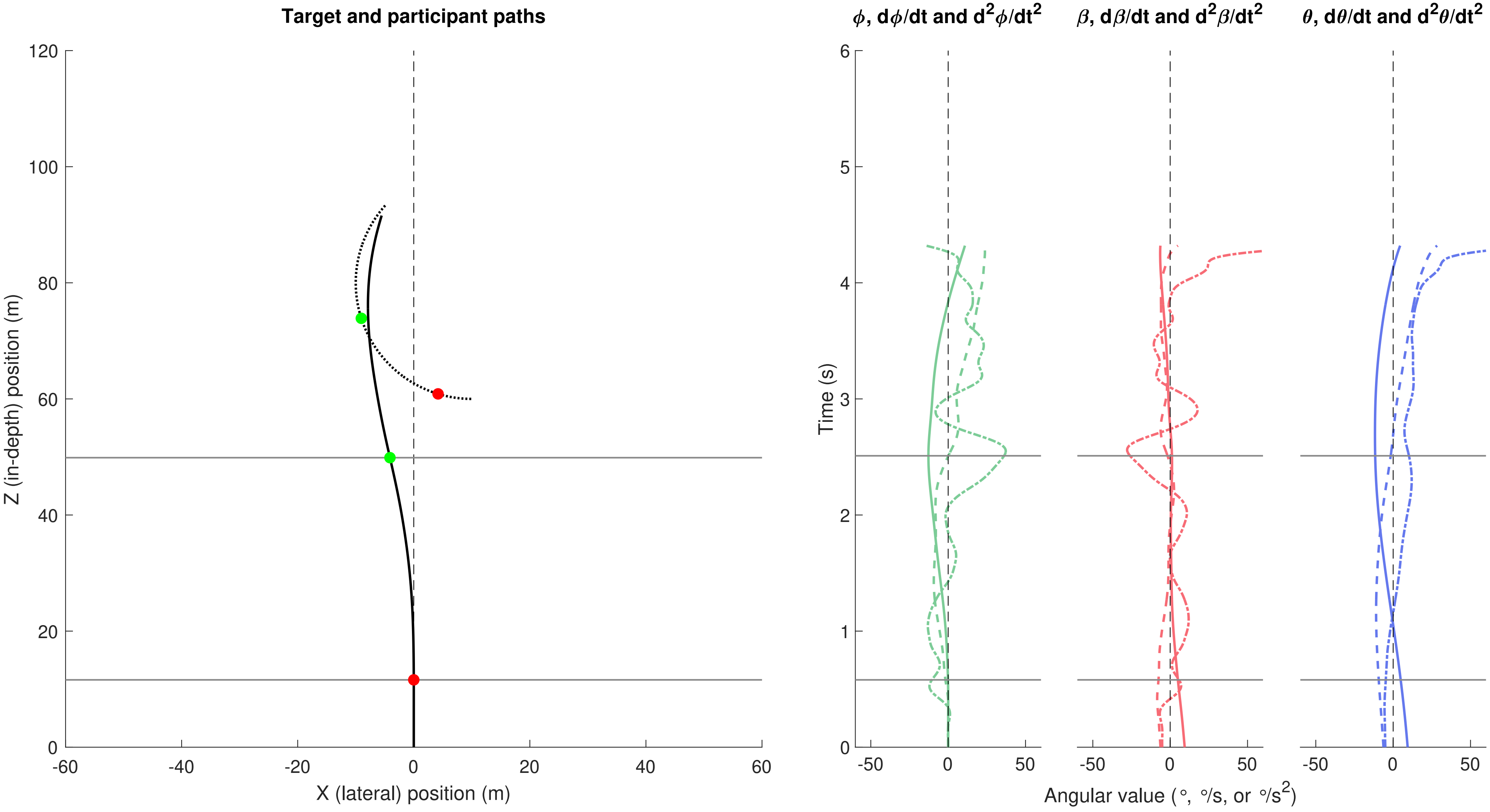

P13/B5
S10/R40-OUT

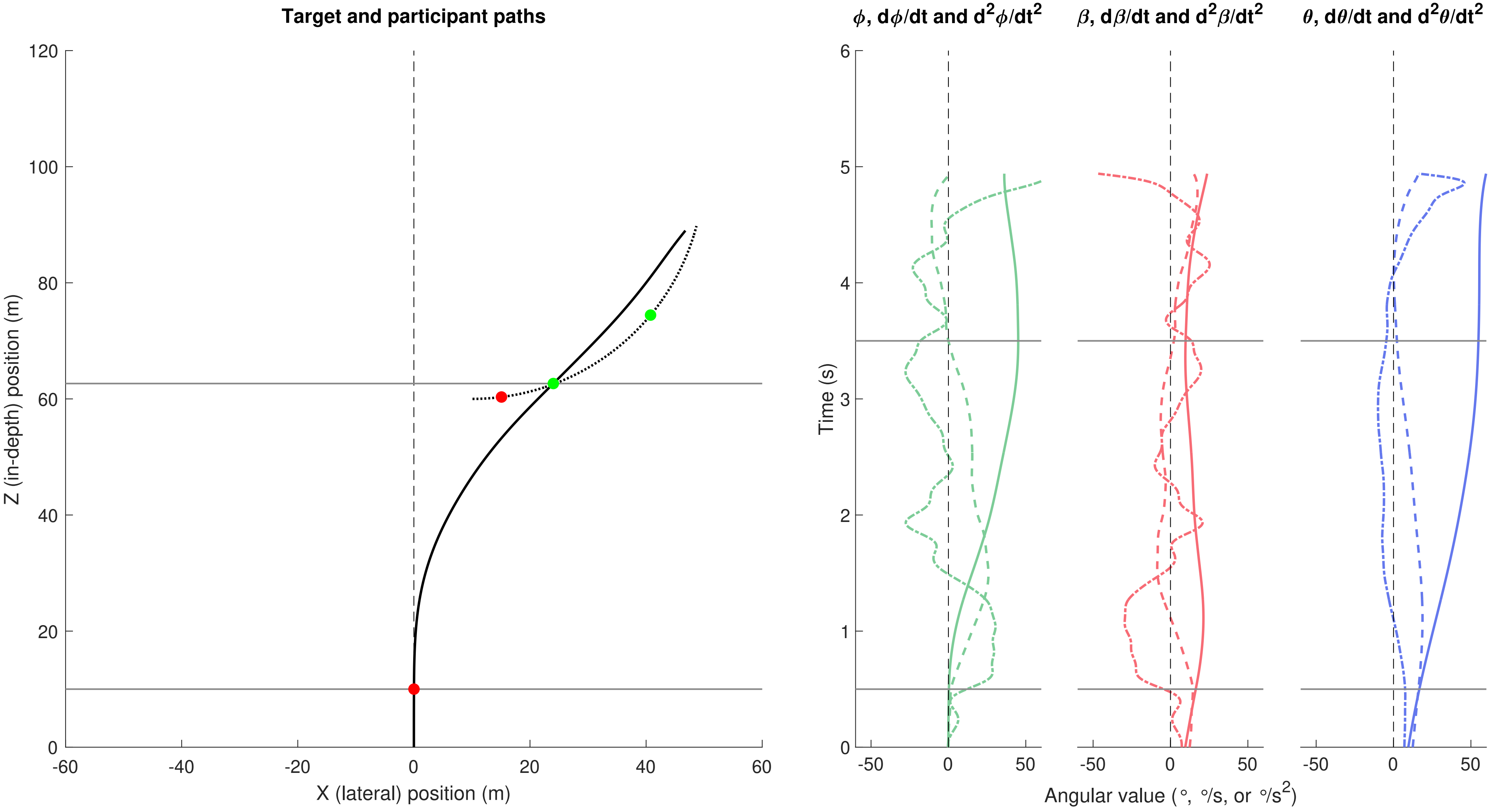

P13/B5
S10/R40-IN

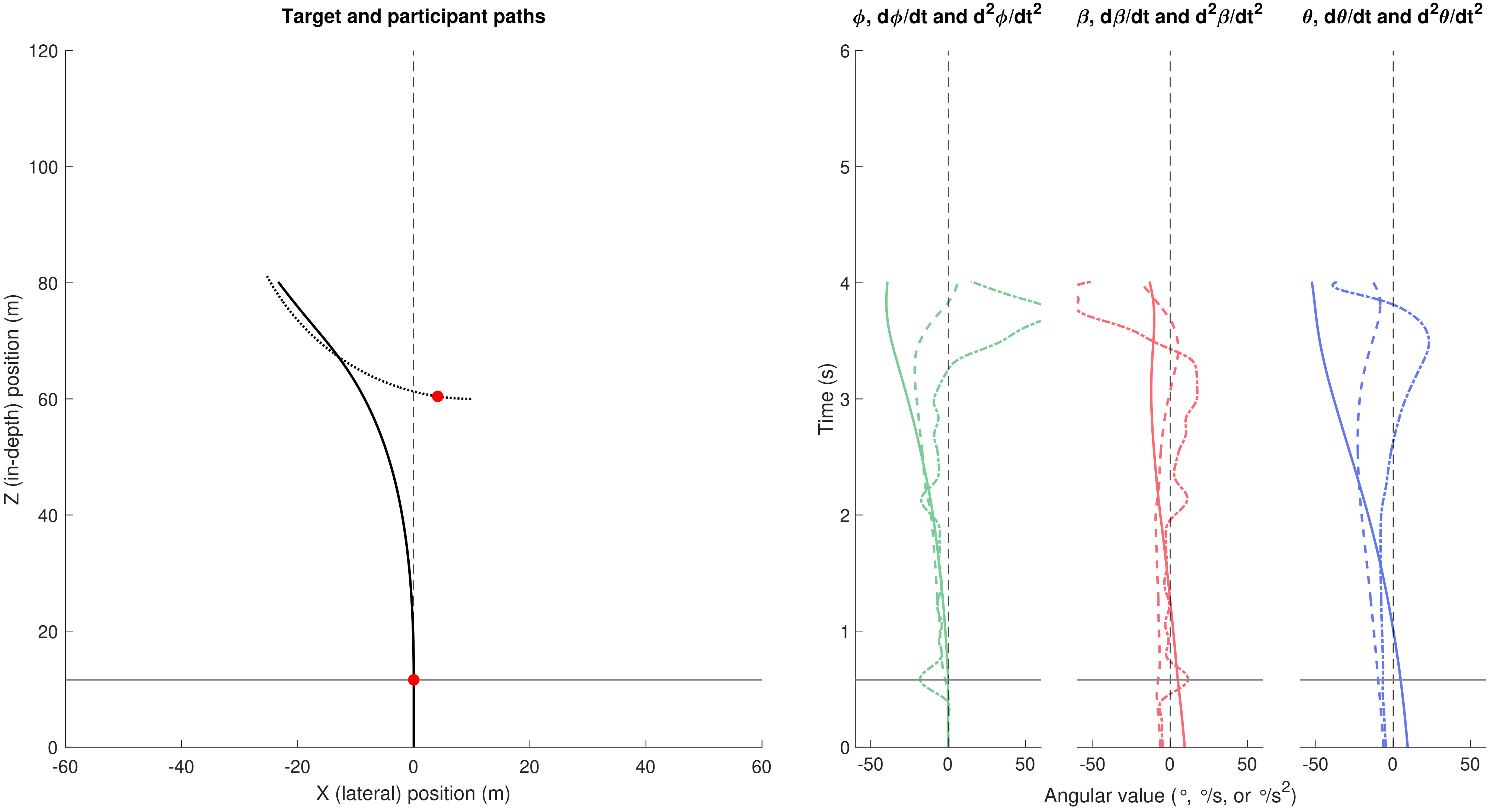

P13/B5
S20/R20-OUT

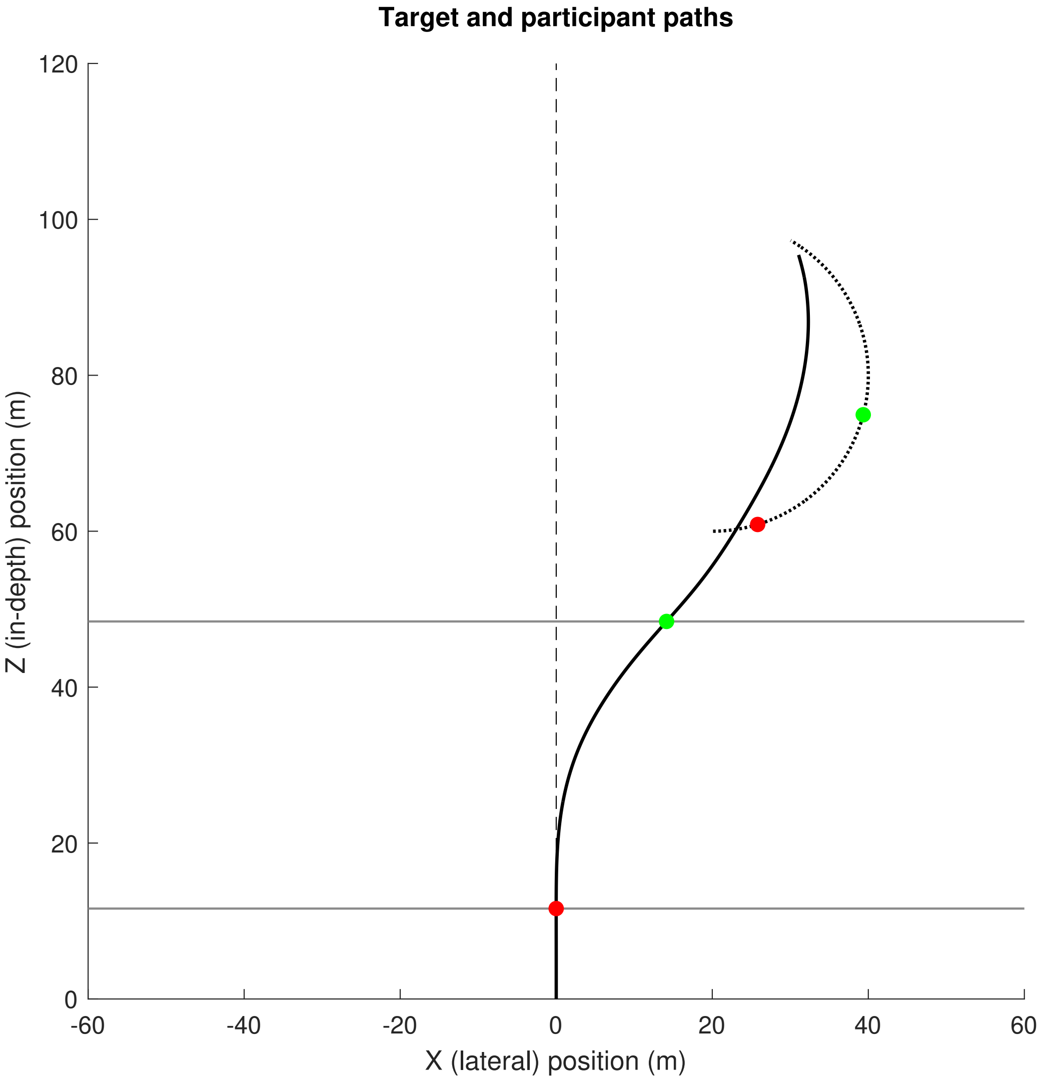

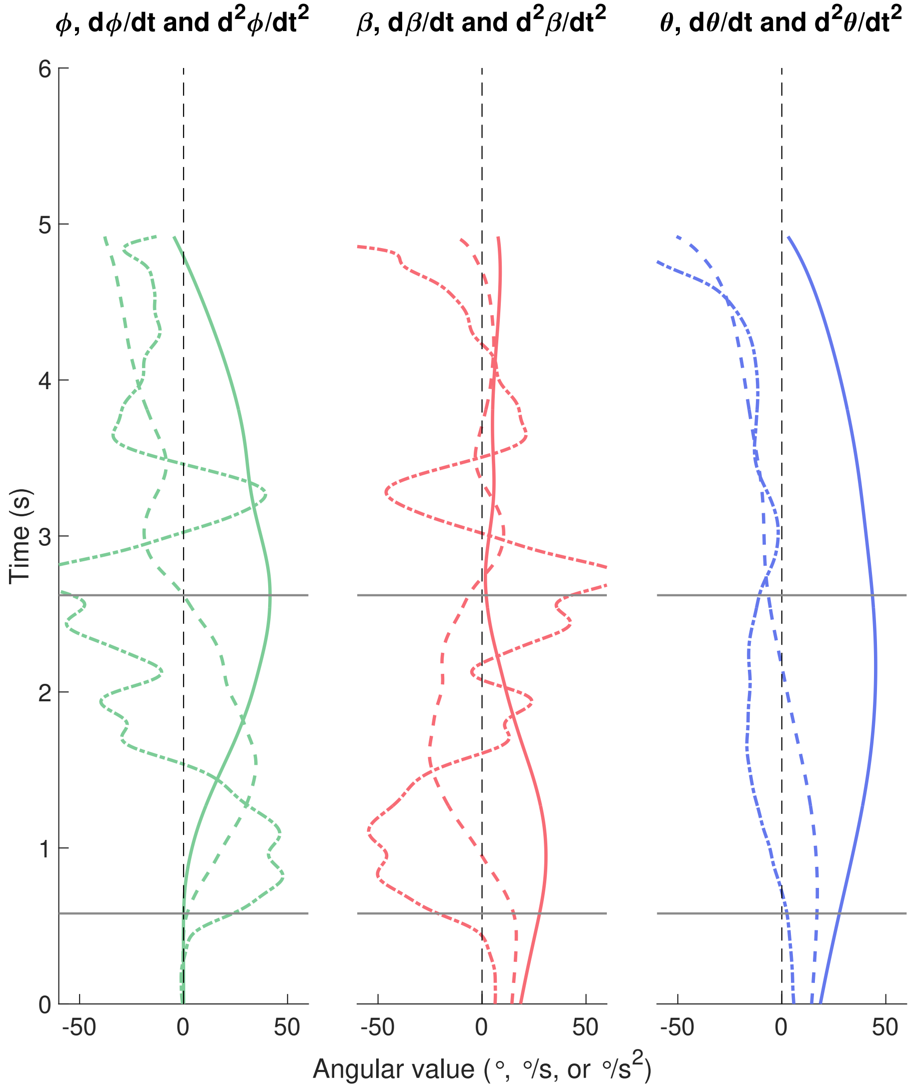

P13/B5
S20/R20-IN

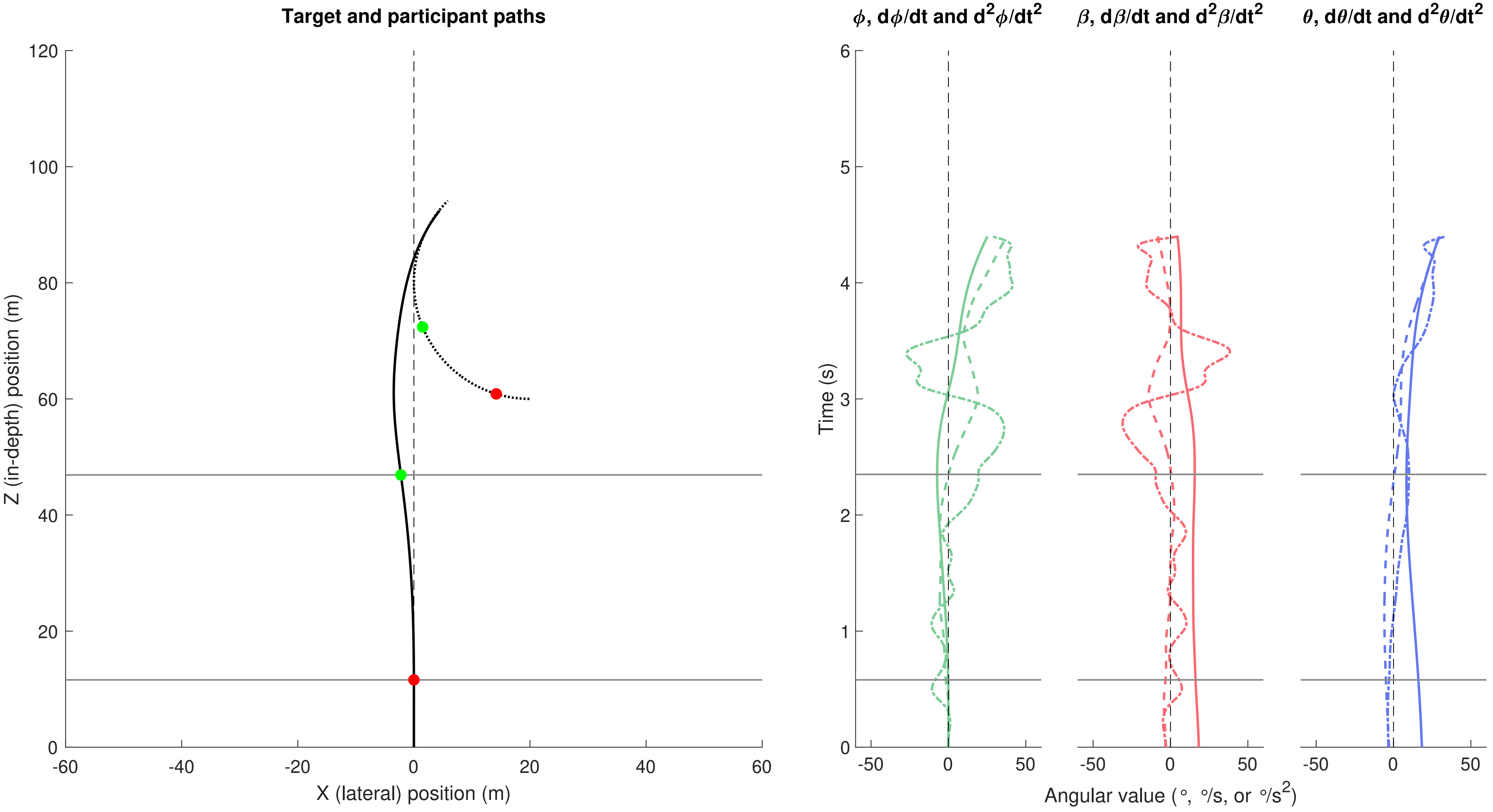

P13/B5
S20/R40-OUT

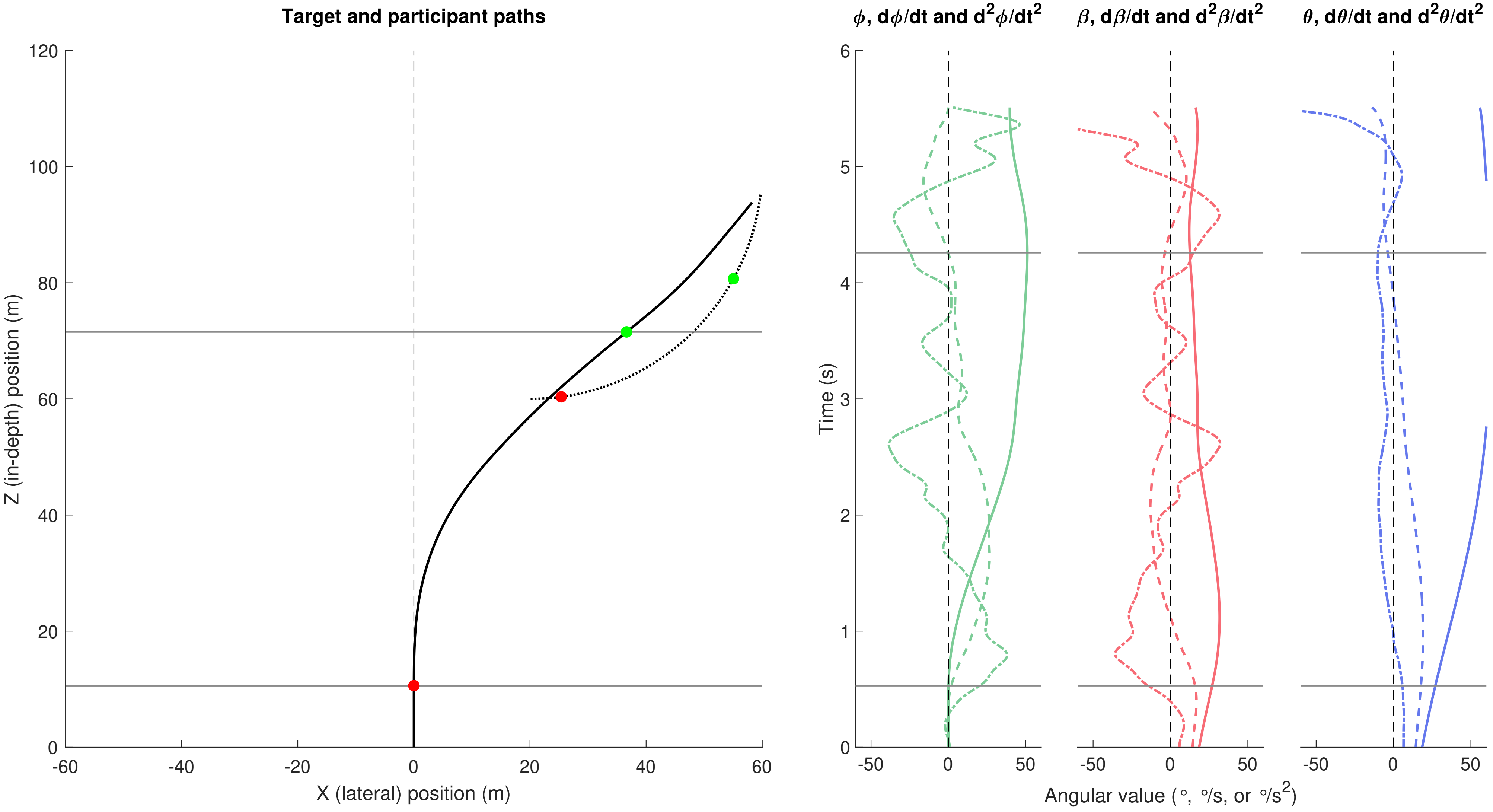

P13/B5
S20/R40-IN

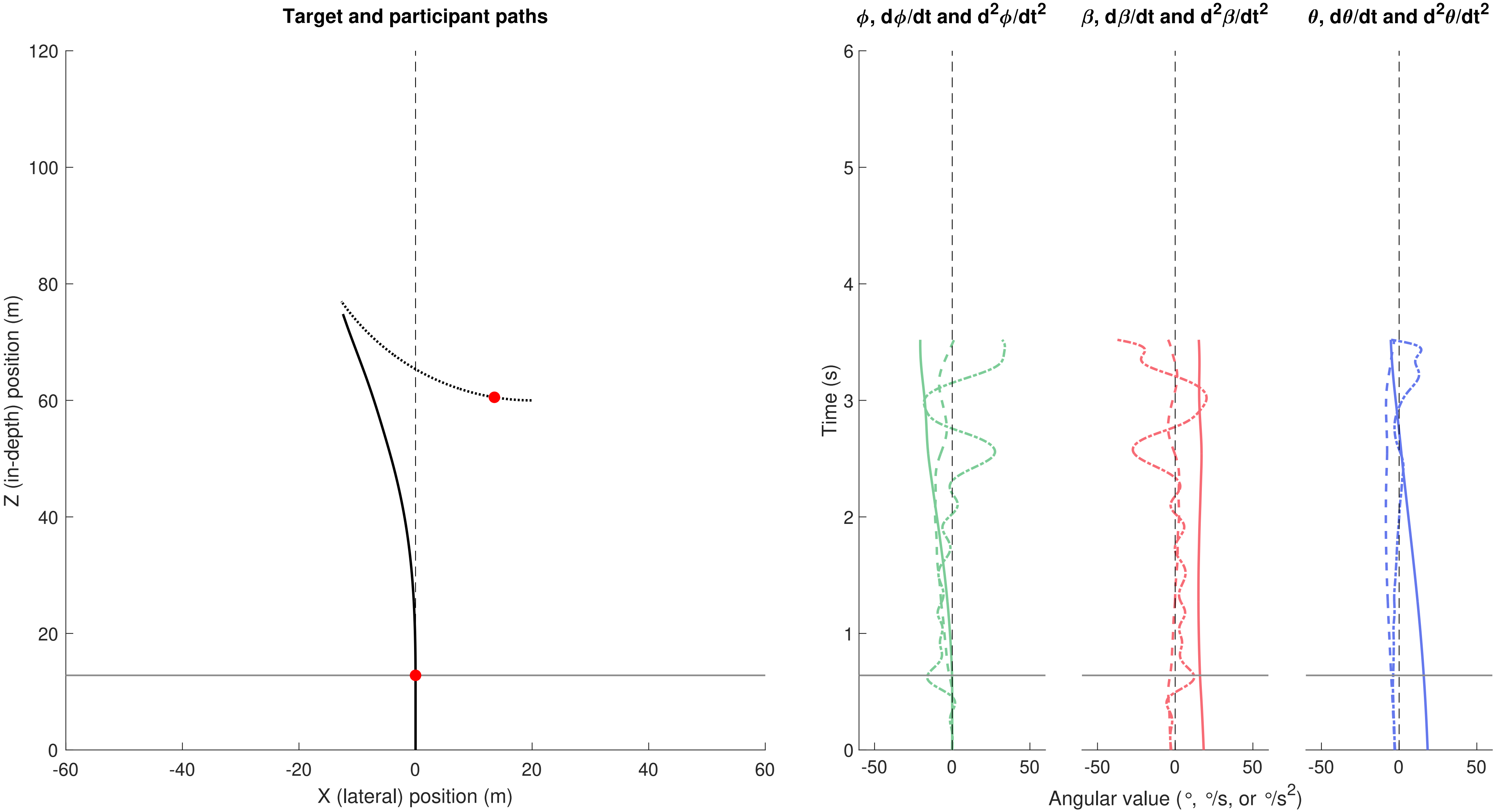

P13/B6
S20/R20-IN

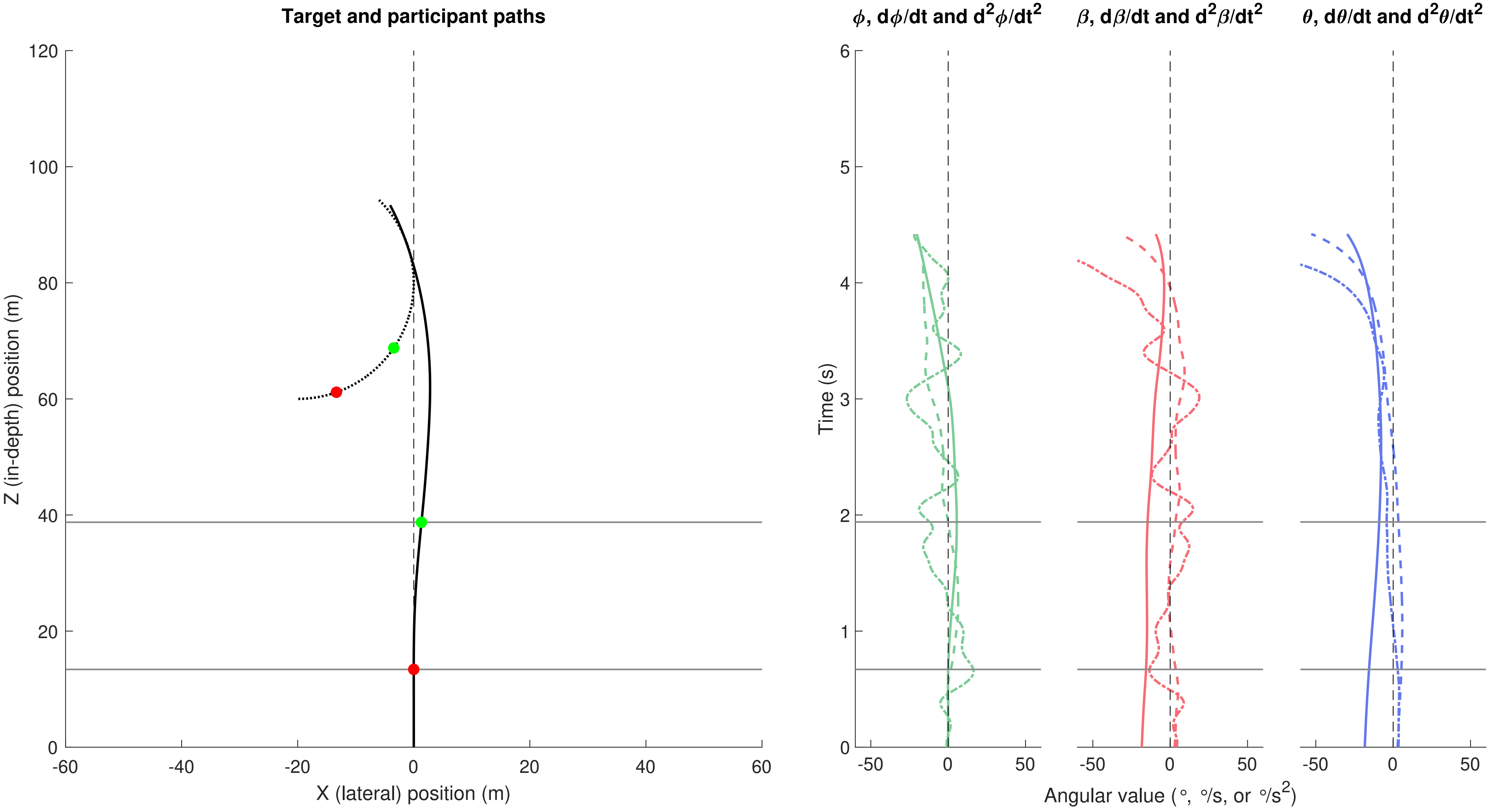

P13/B6
S20/R20-OUT

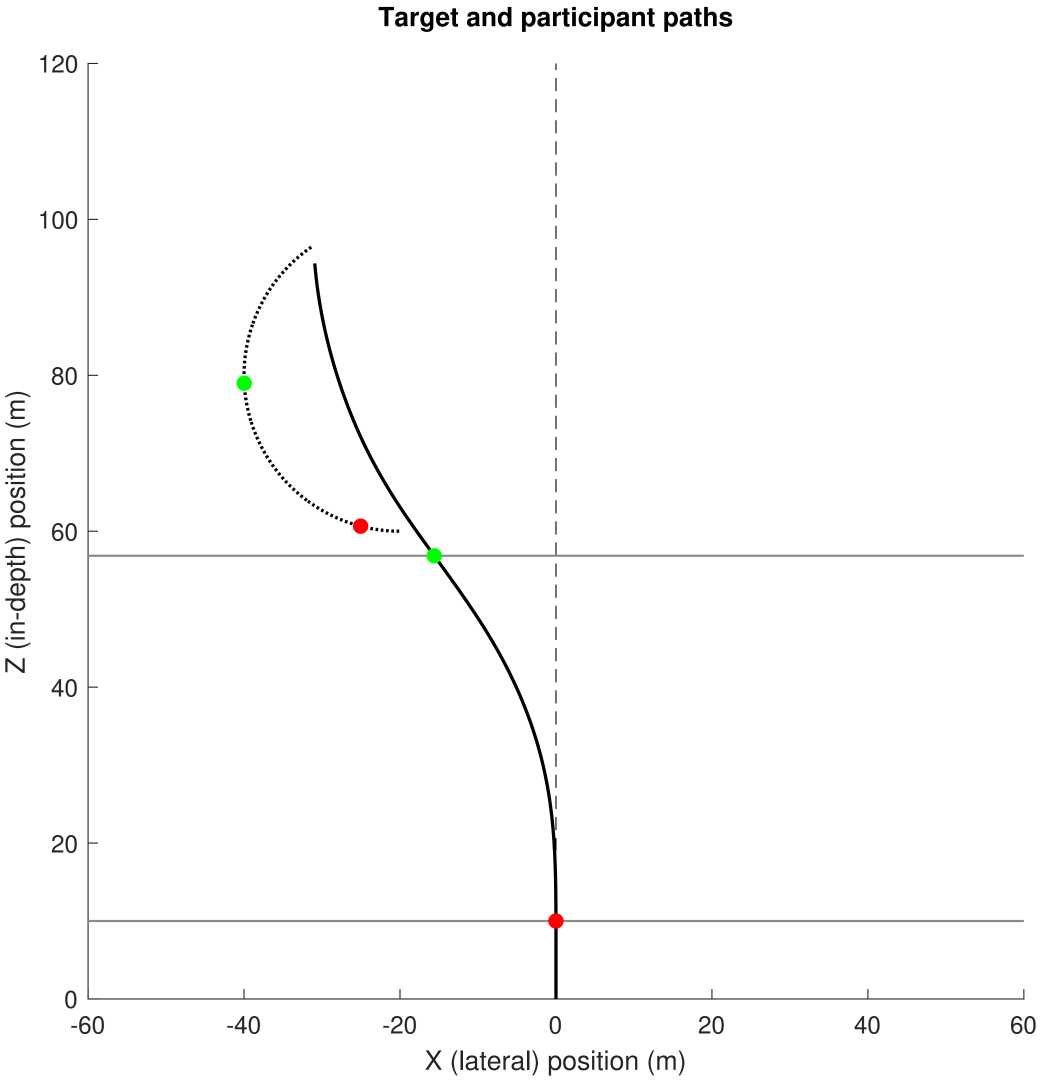

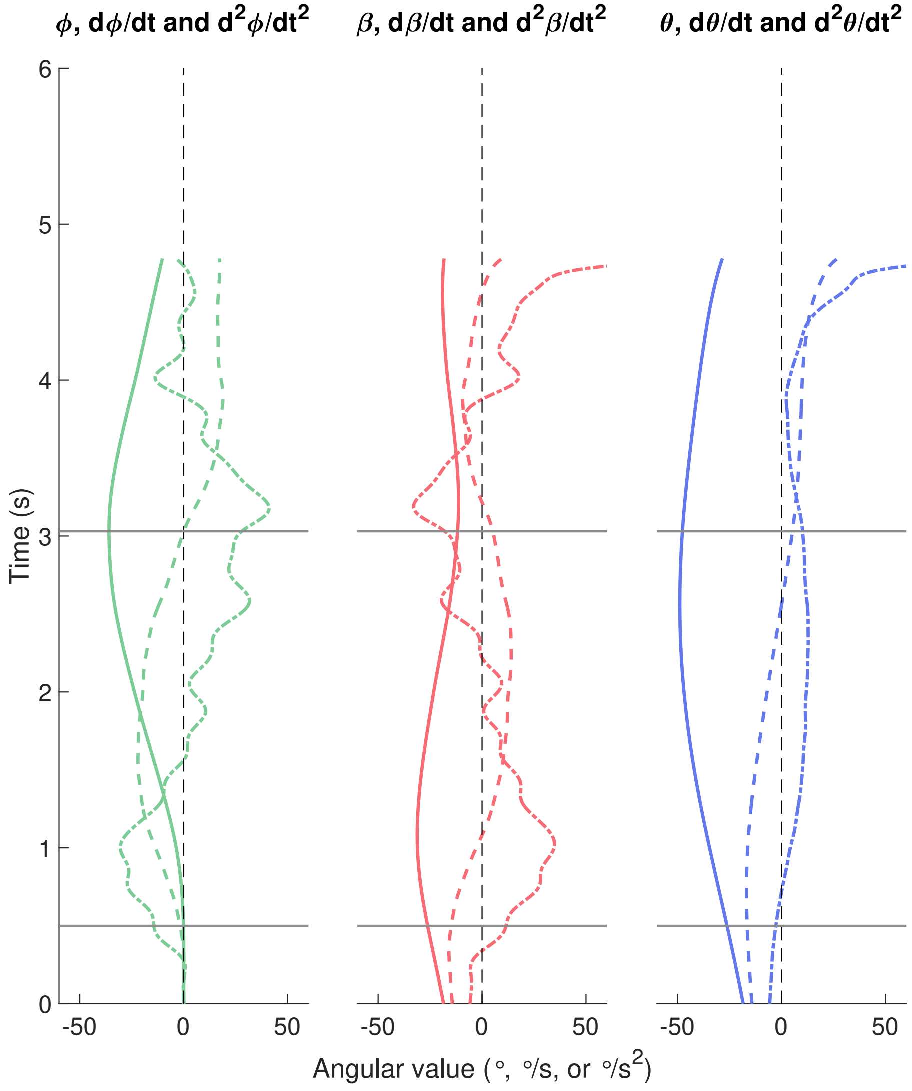

P13/B6
S20/R40-IN

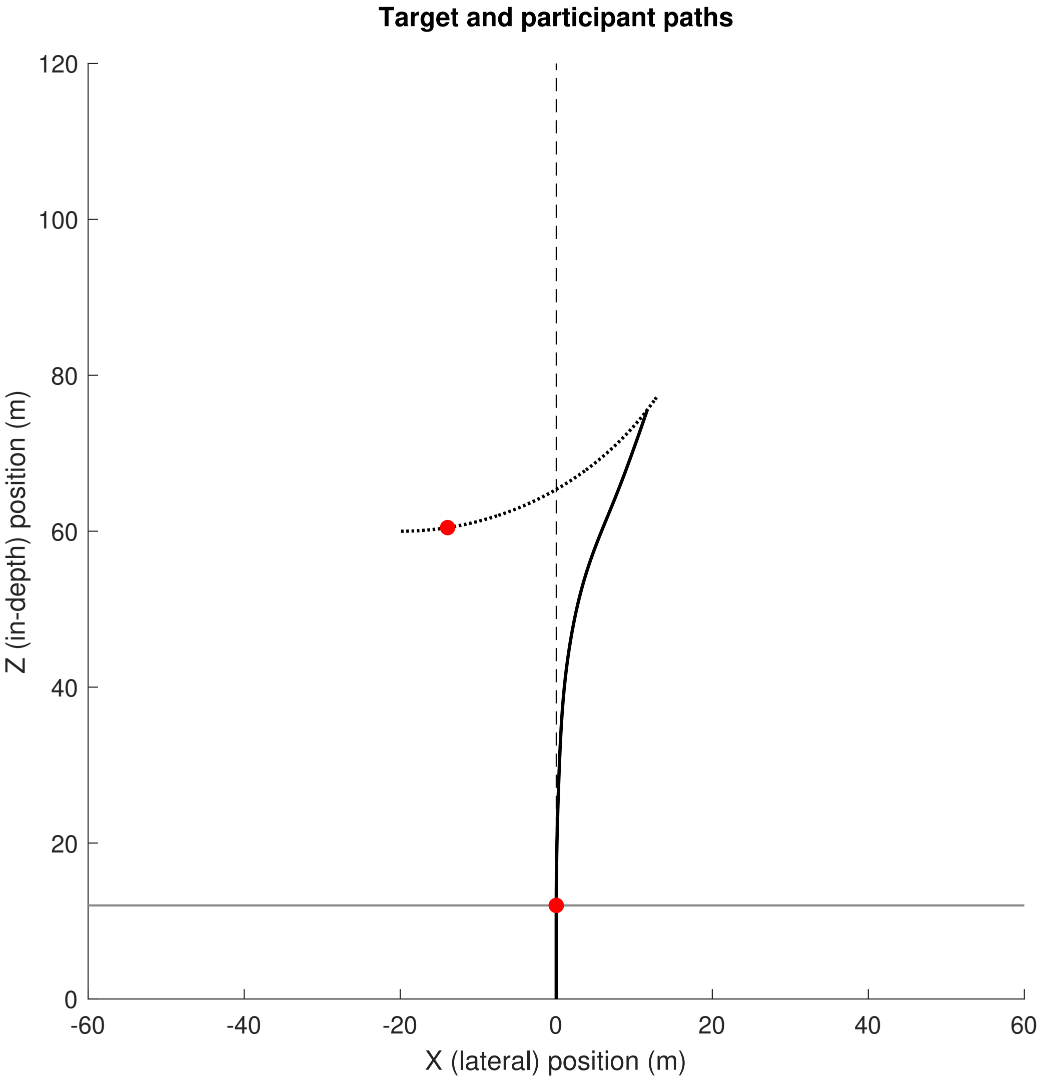

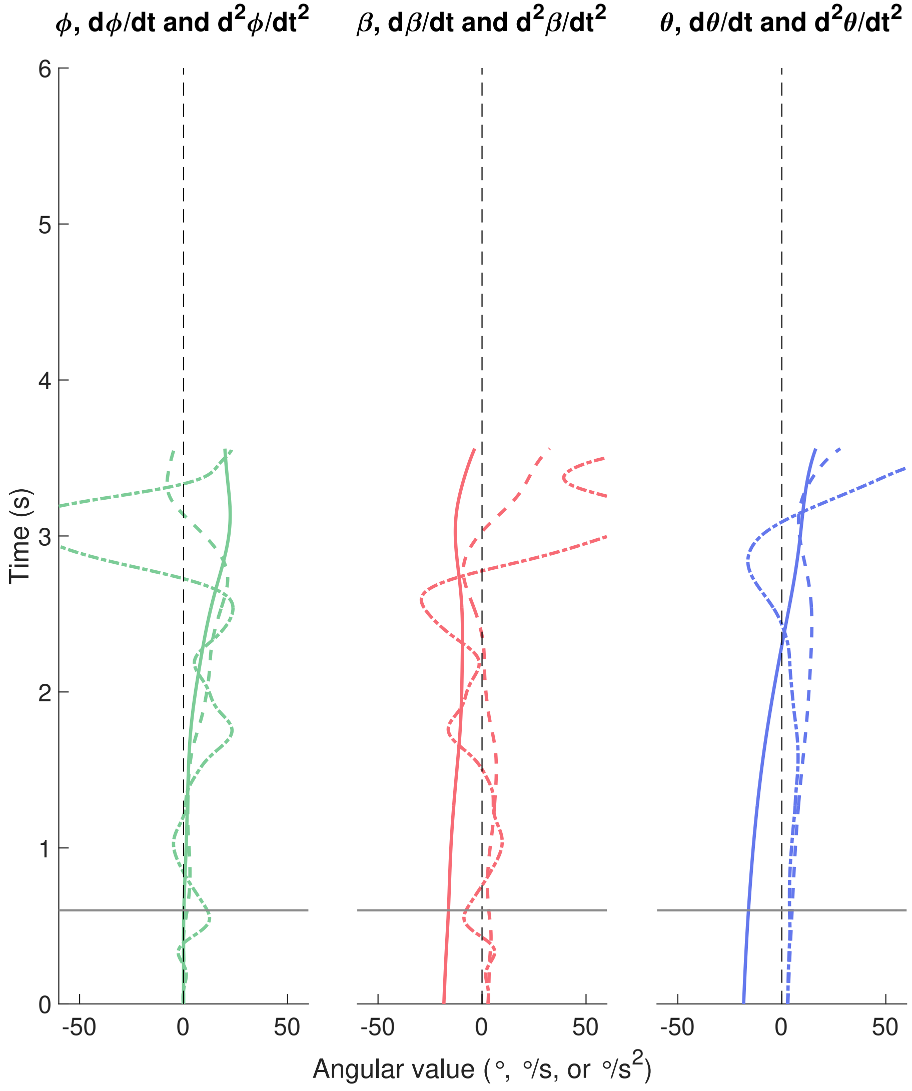

P13/B6
S20/R40-OUT

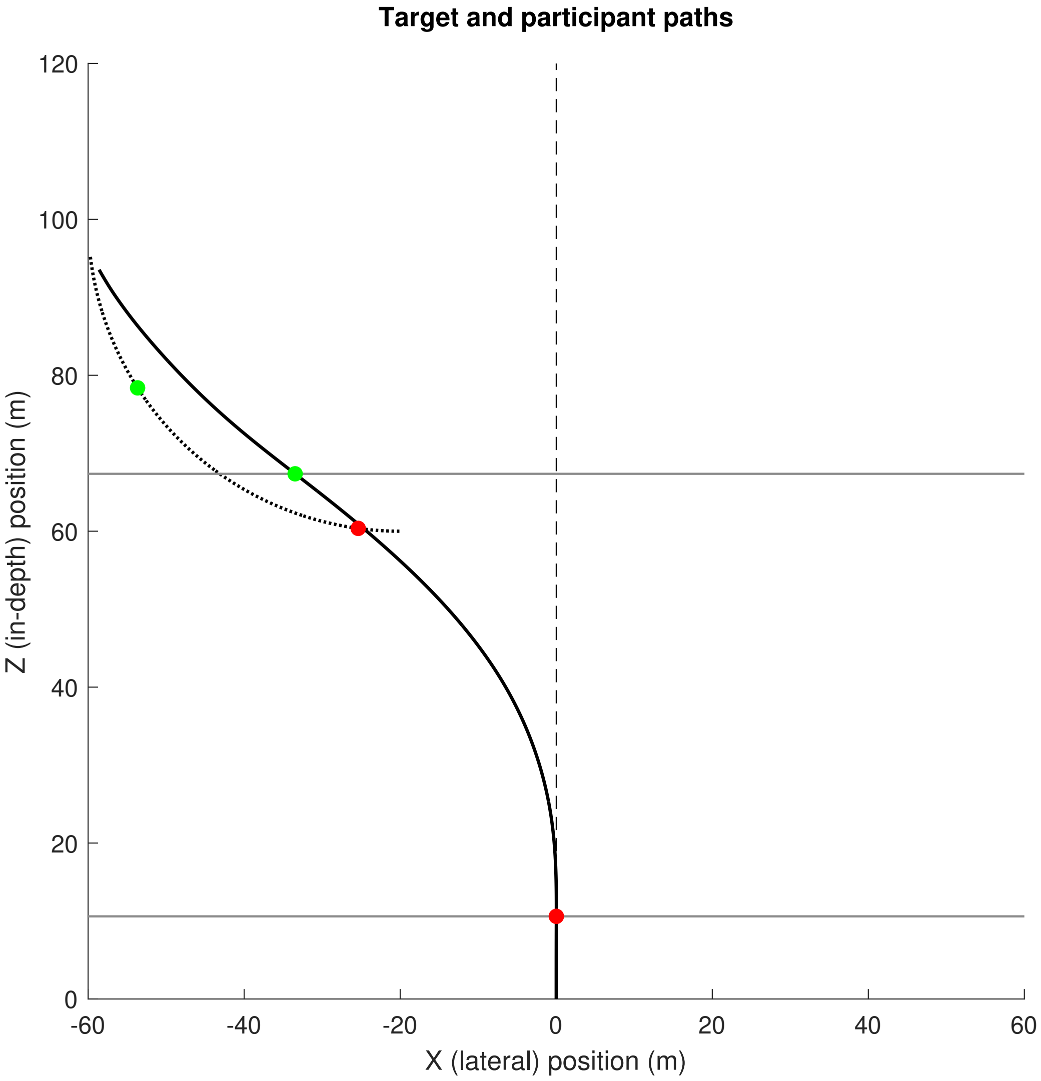

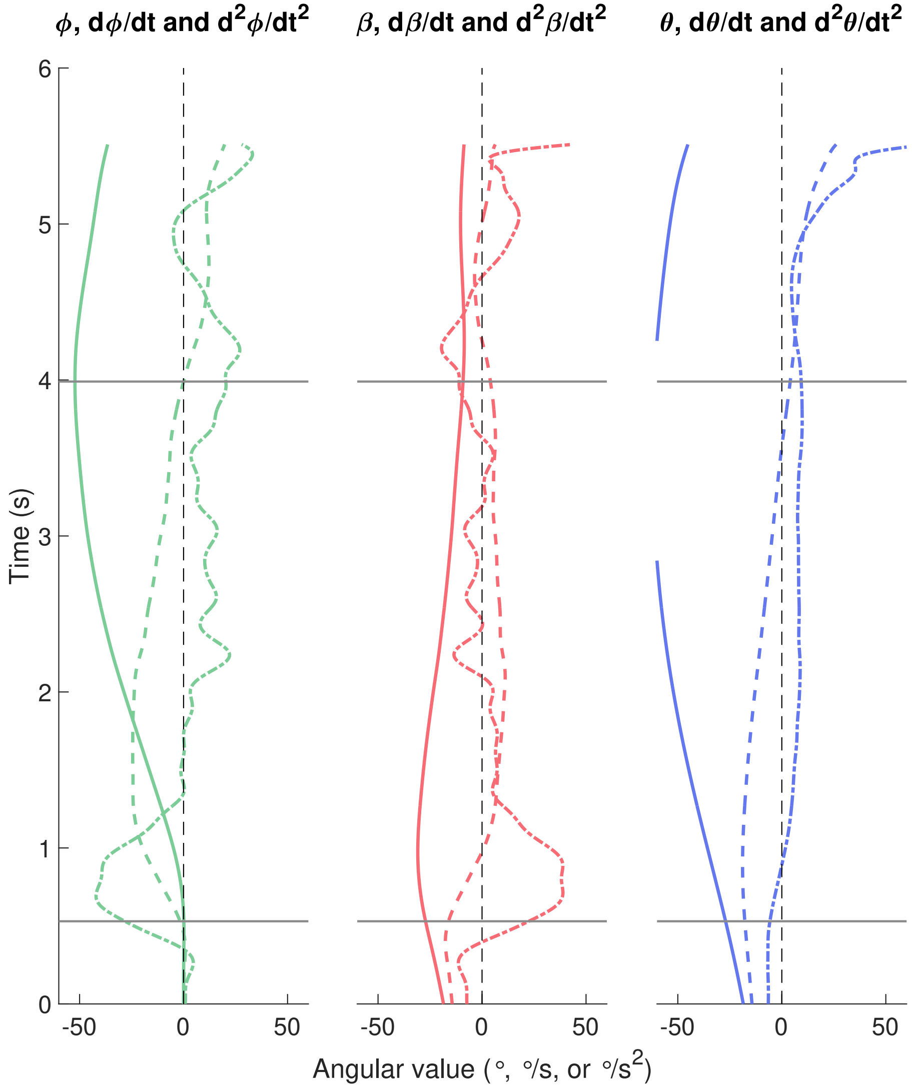

P13/B6
S10/R20-IN

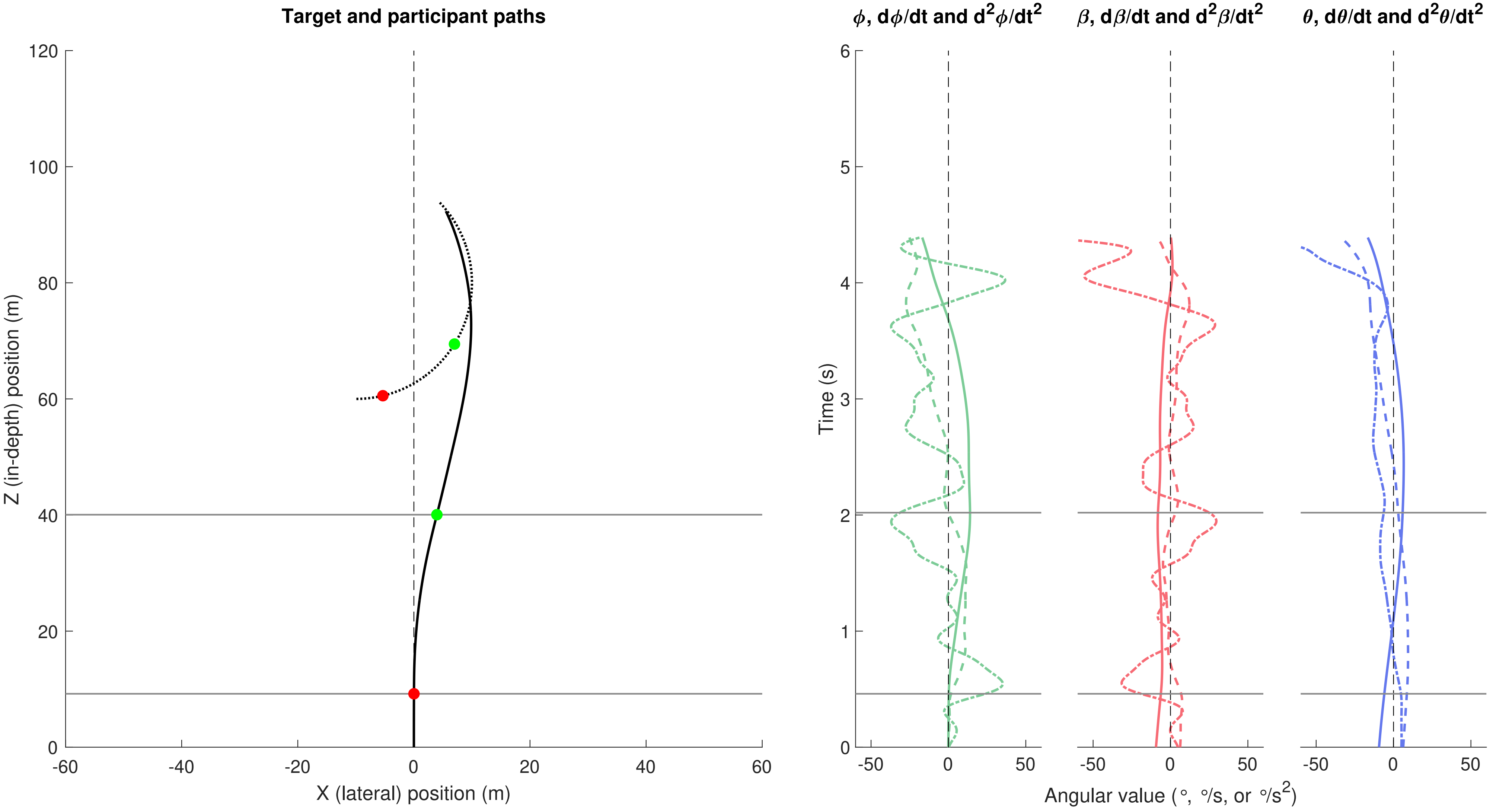

P13/B6
S10/R20-OUT

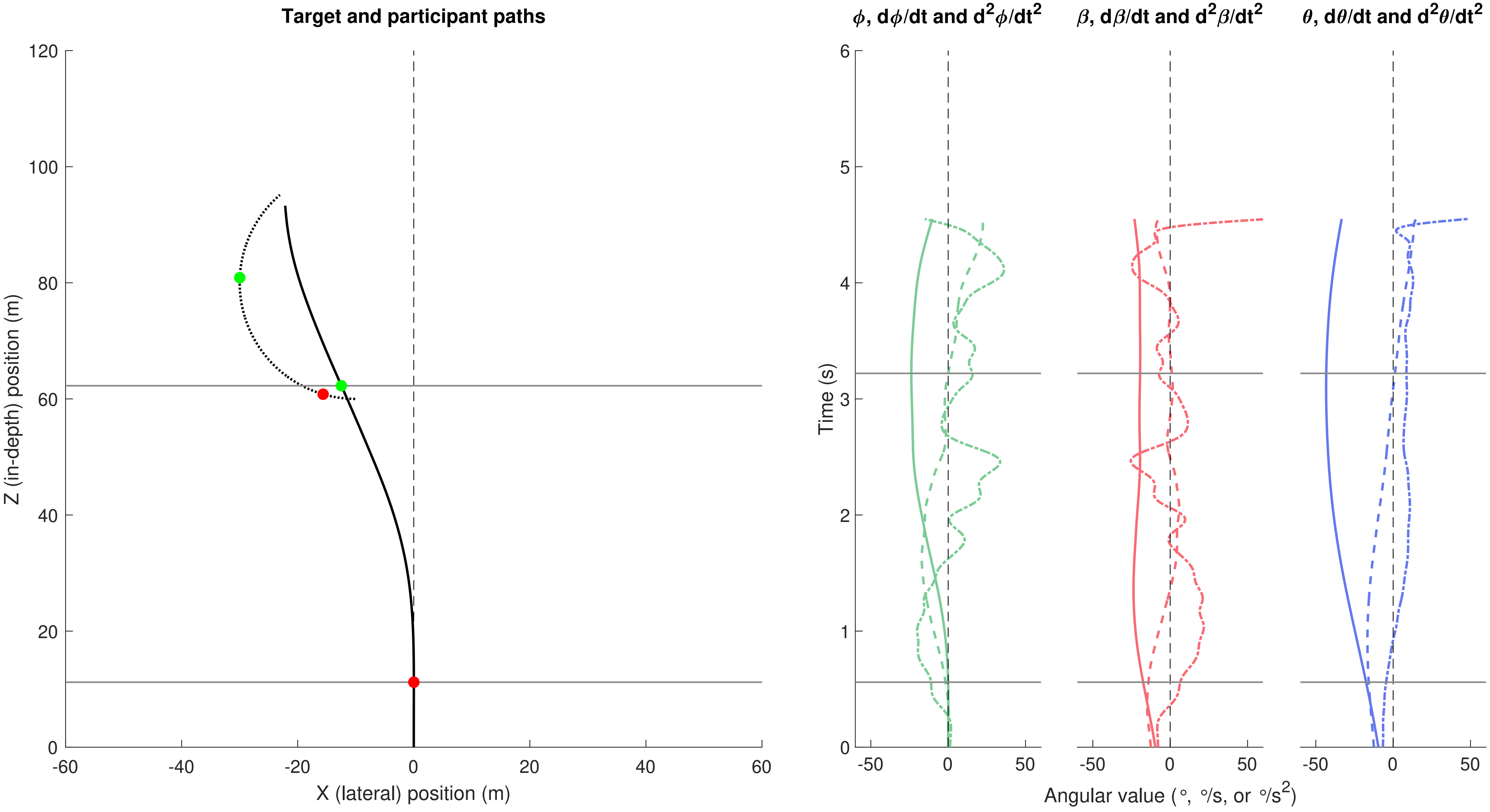

P13/B6
S10/R40-IN

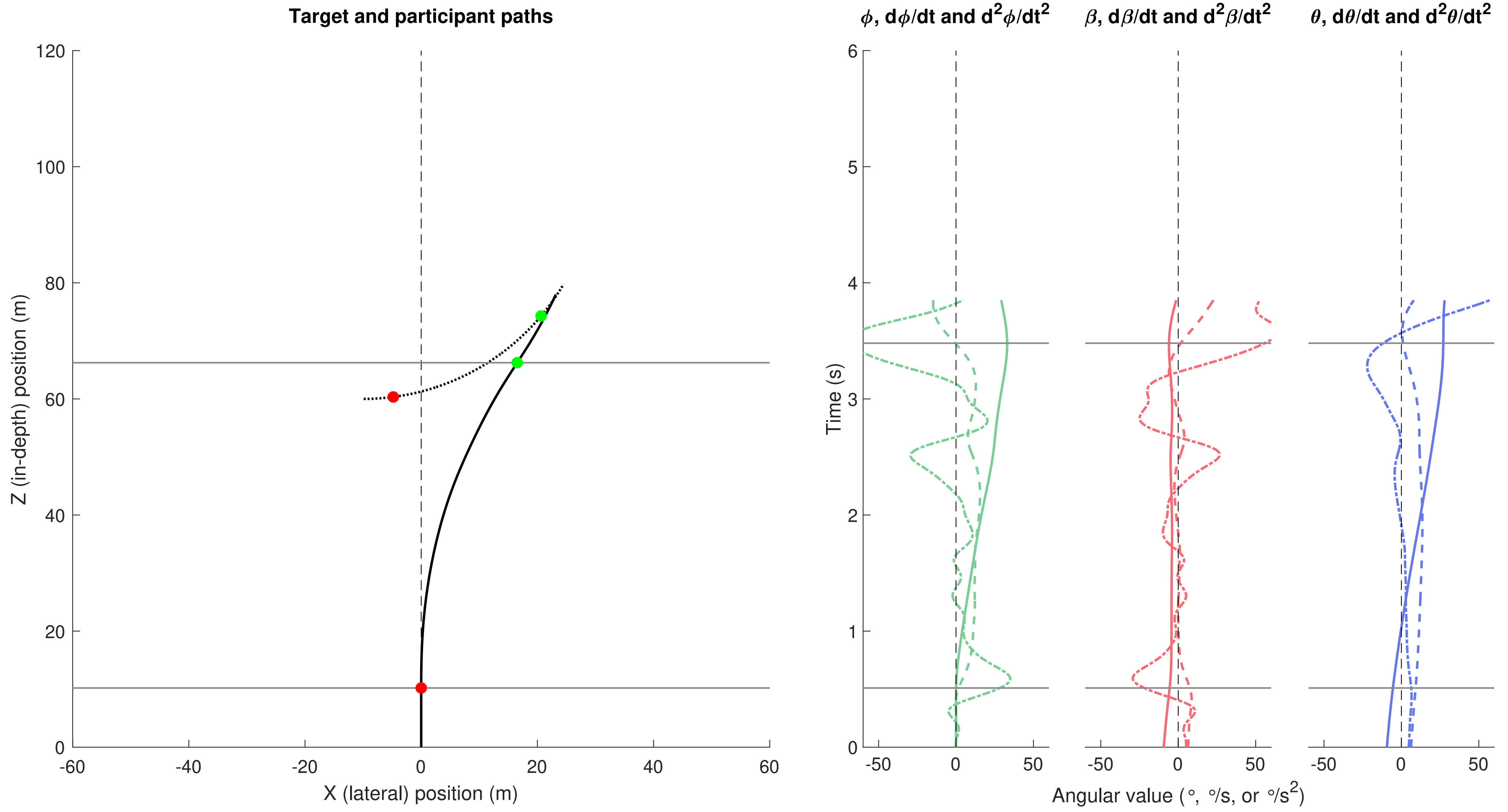

P13/B6
S10/R40-OUT

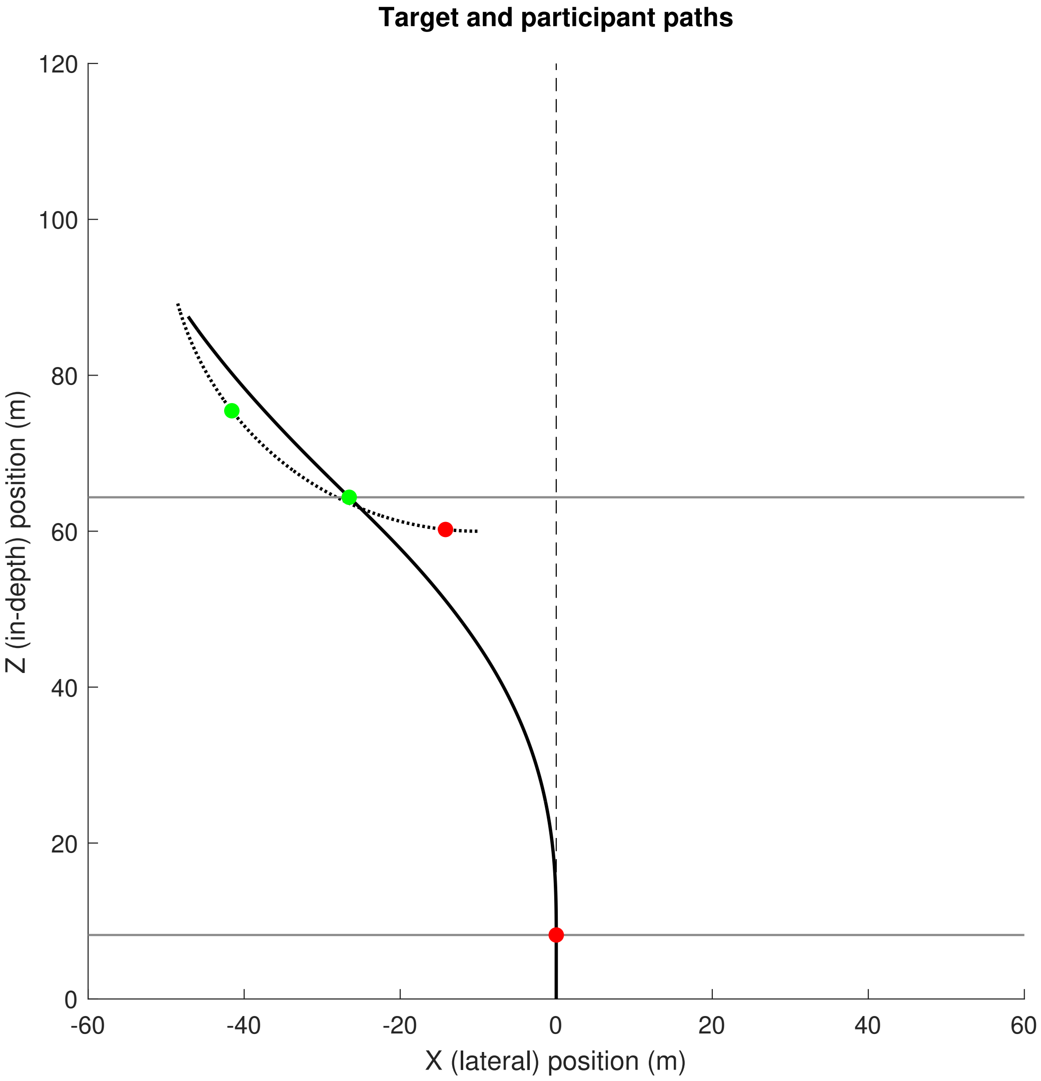

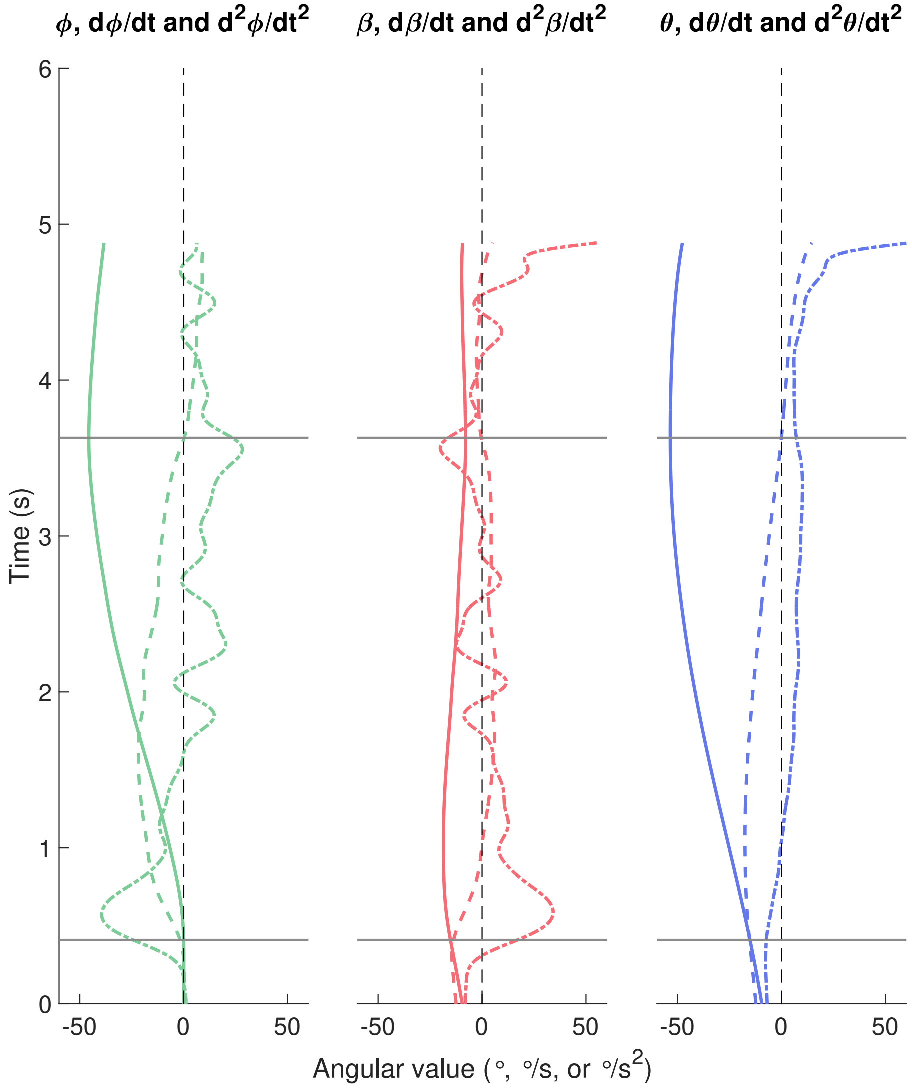

P13/B6
S0/R20-OUT

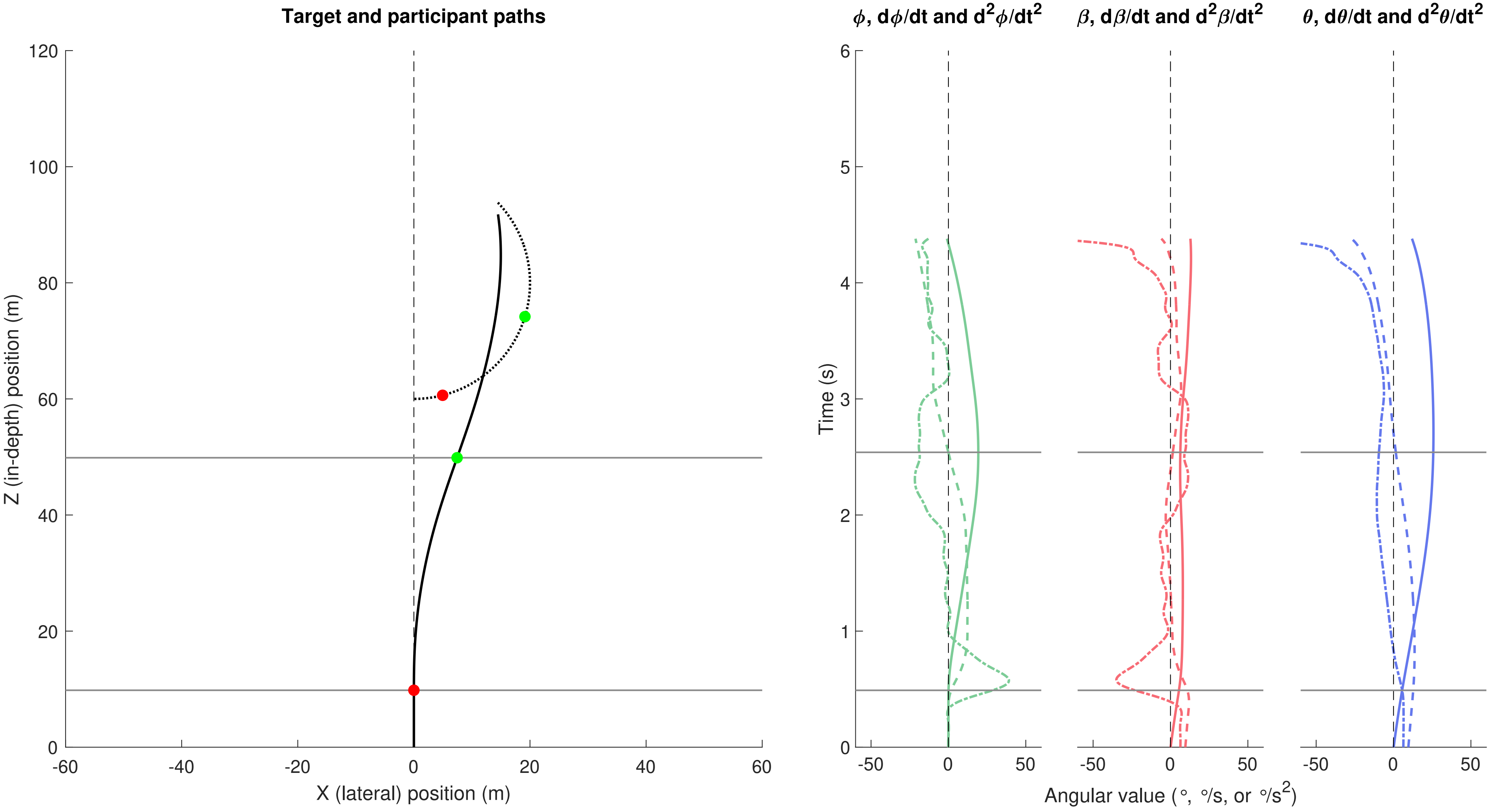

P13/B6
S0/R20-OUT

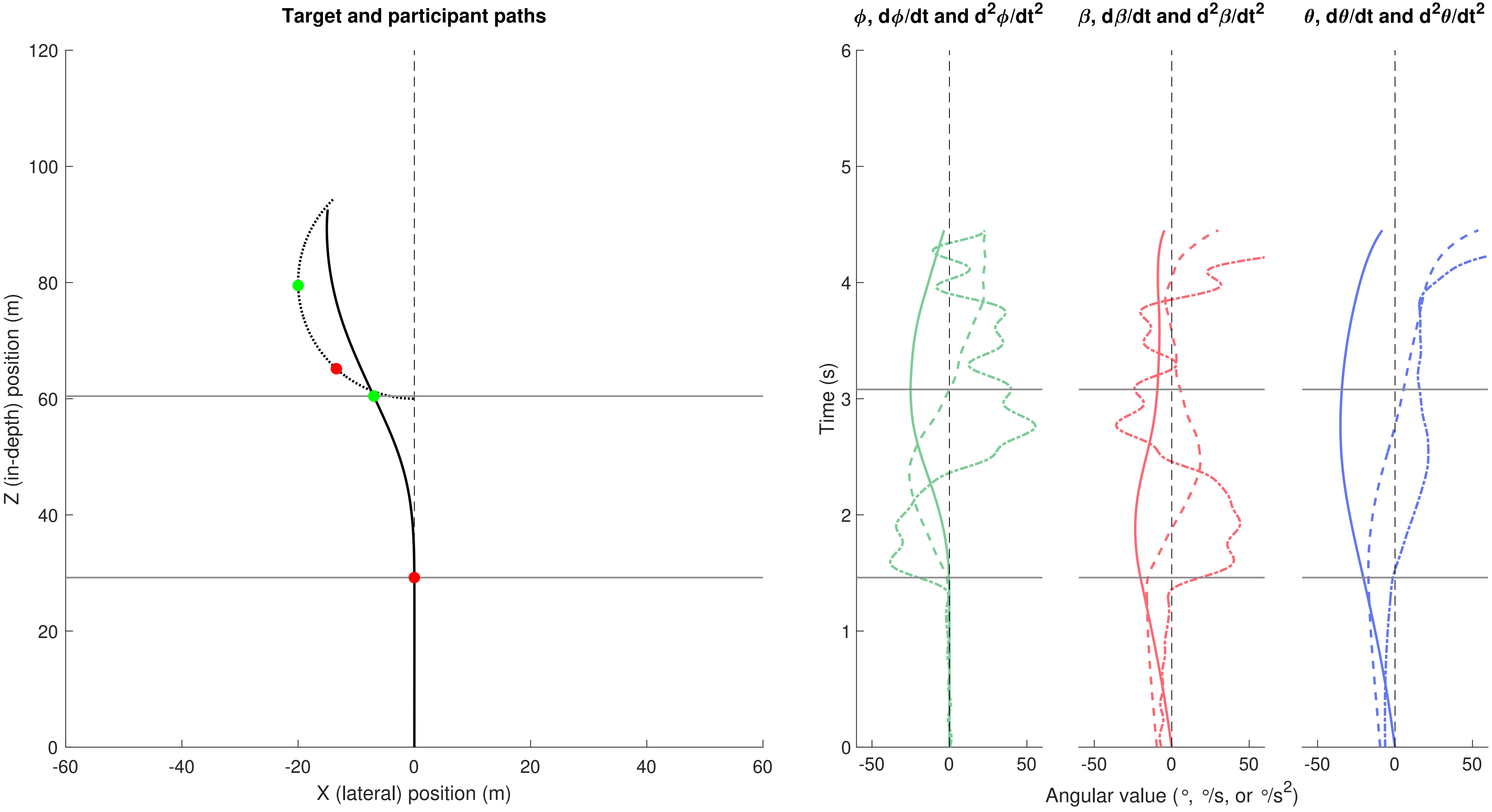

P13/B6
S0/R40-OUT

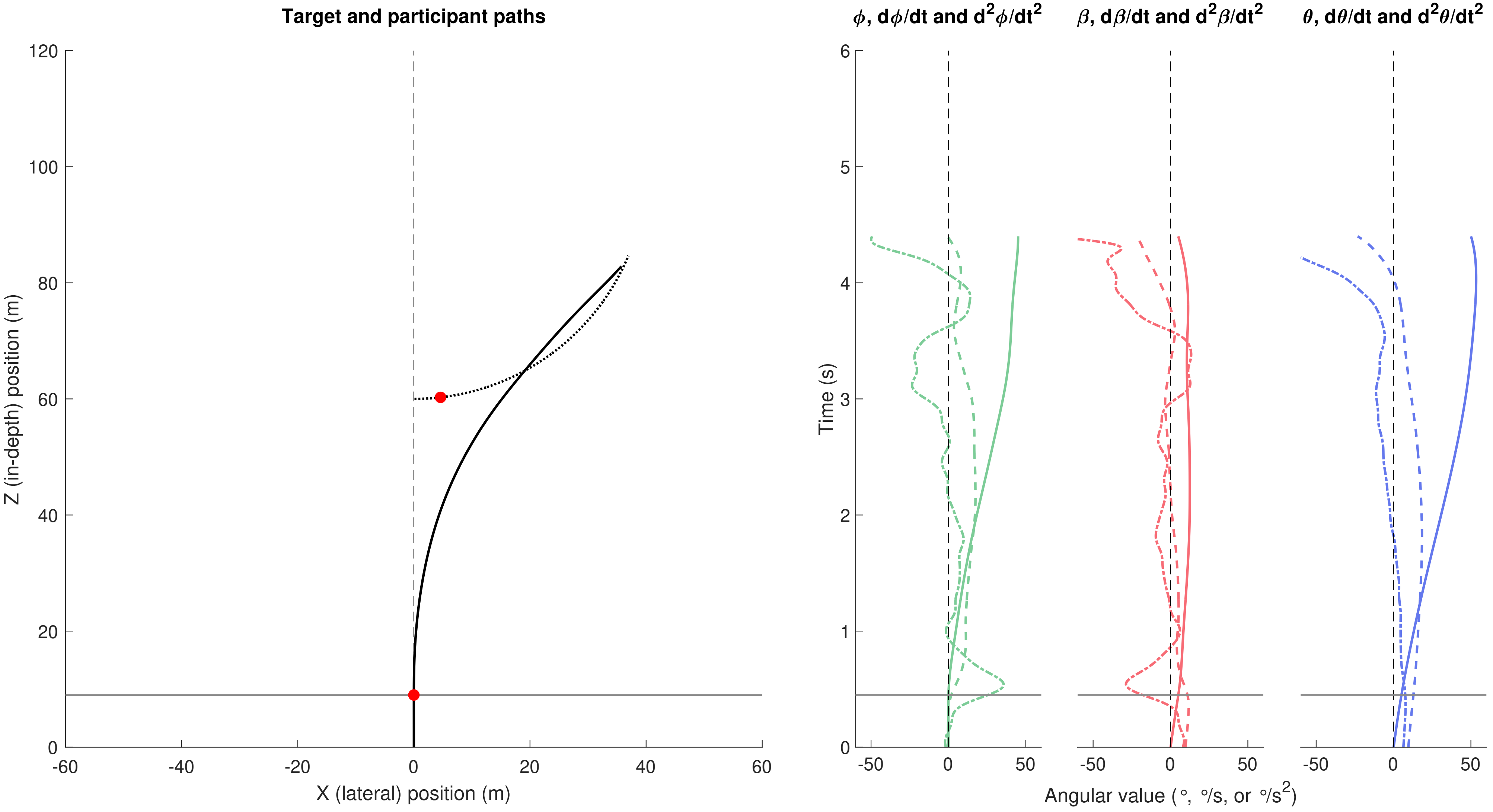

P13/B6
S0/R40-OUT

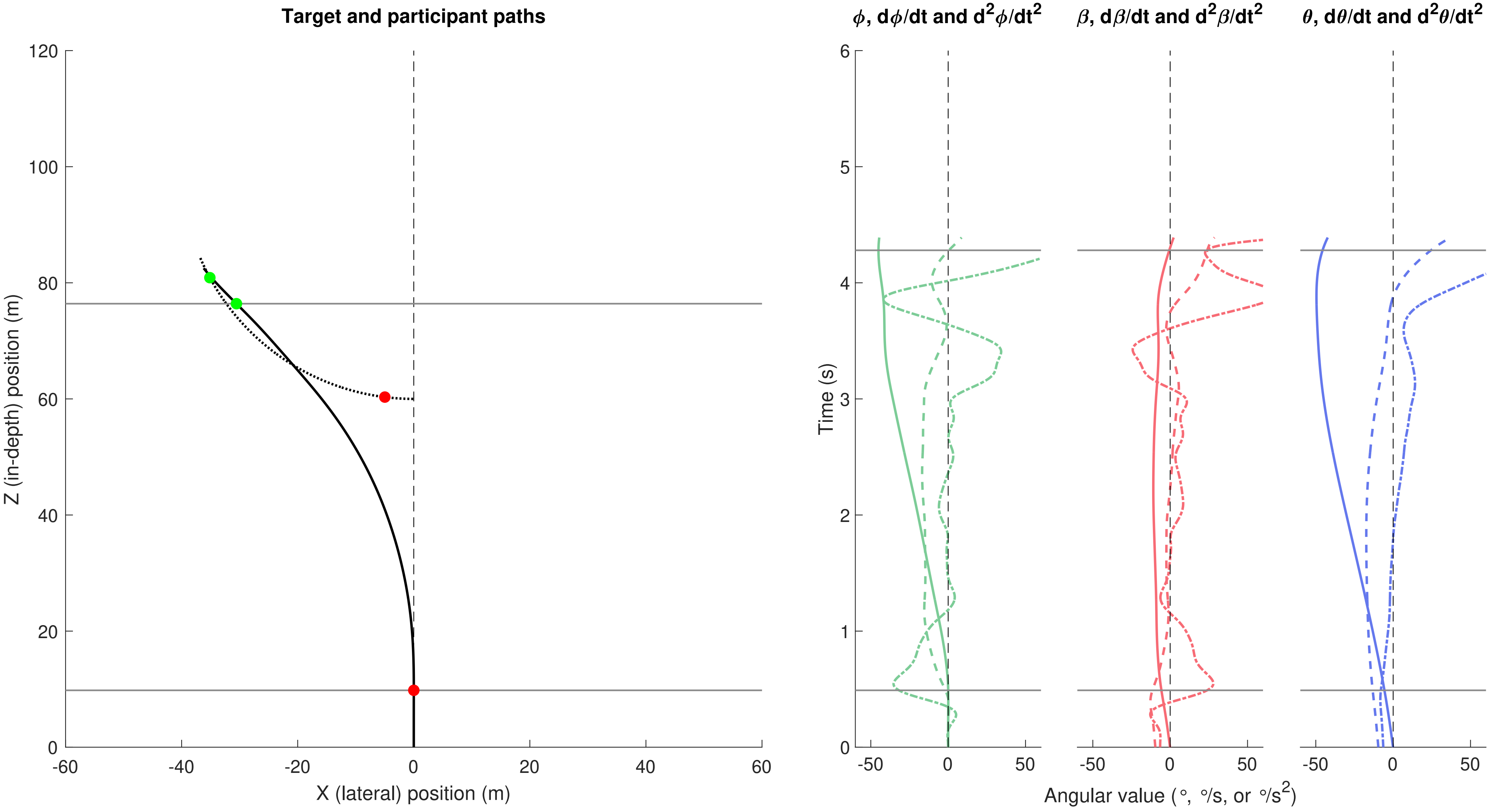

P13/B6
S10/R20-OUT

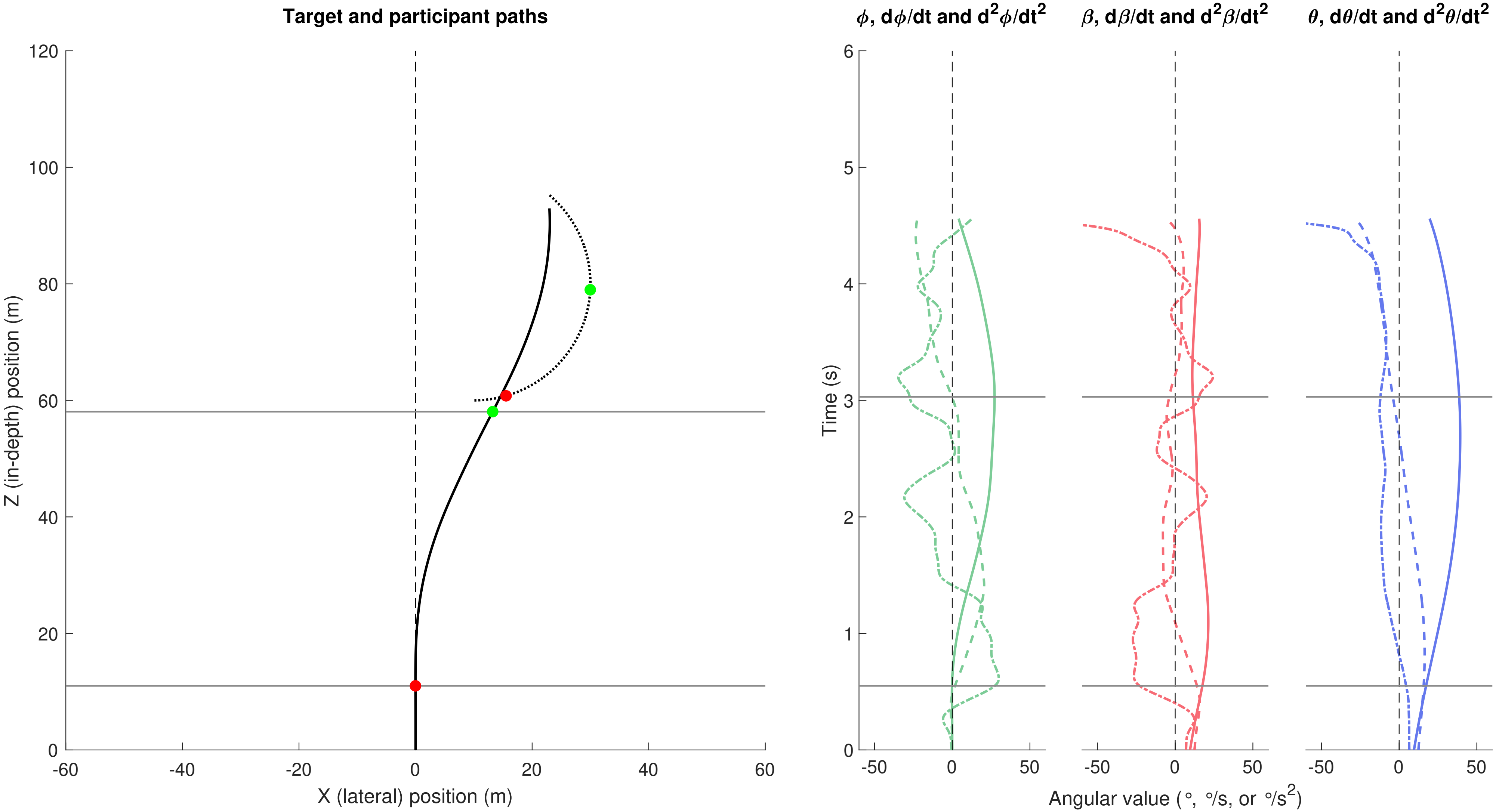

P13/B6
S10/R20-IN

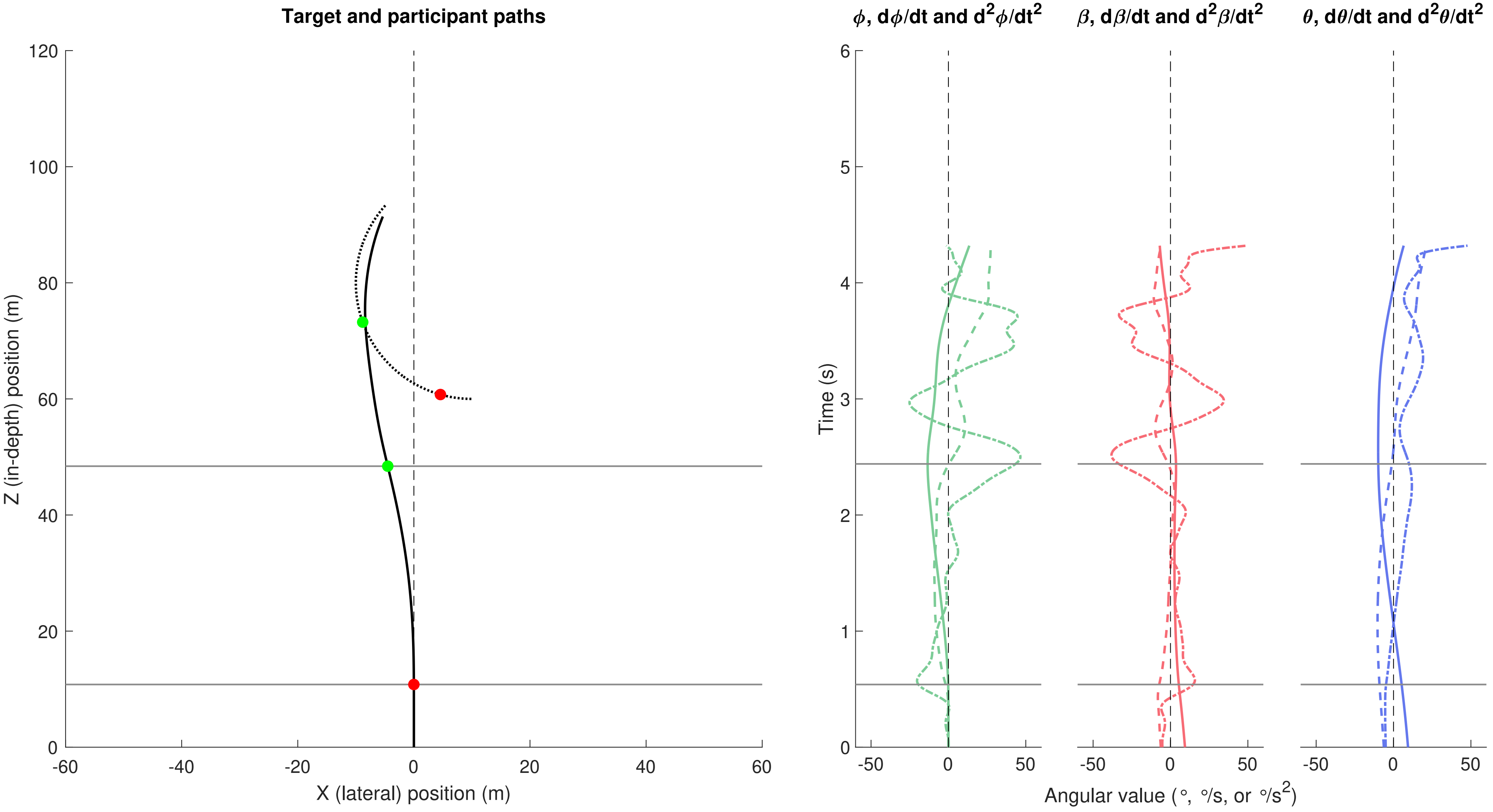

P13/B6
S10/R40-OUT

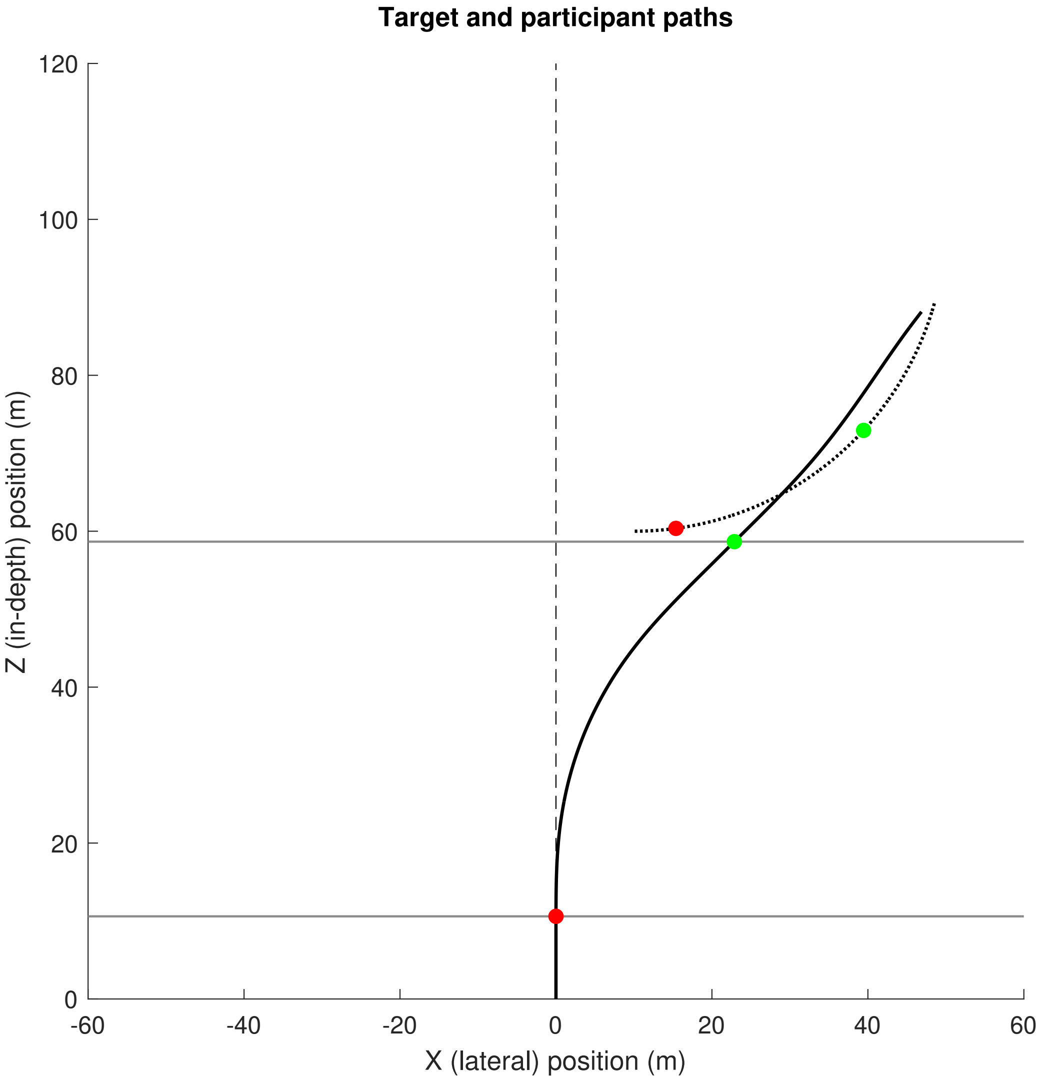

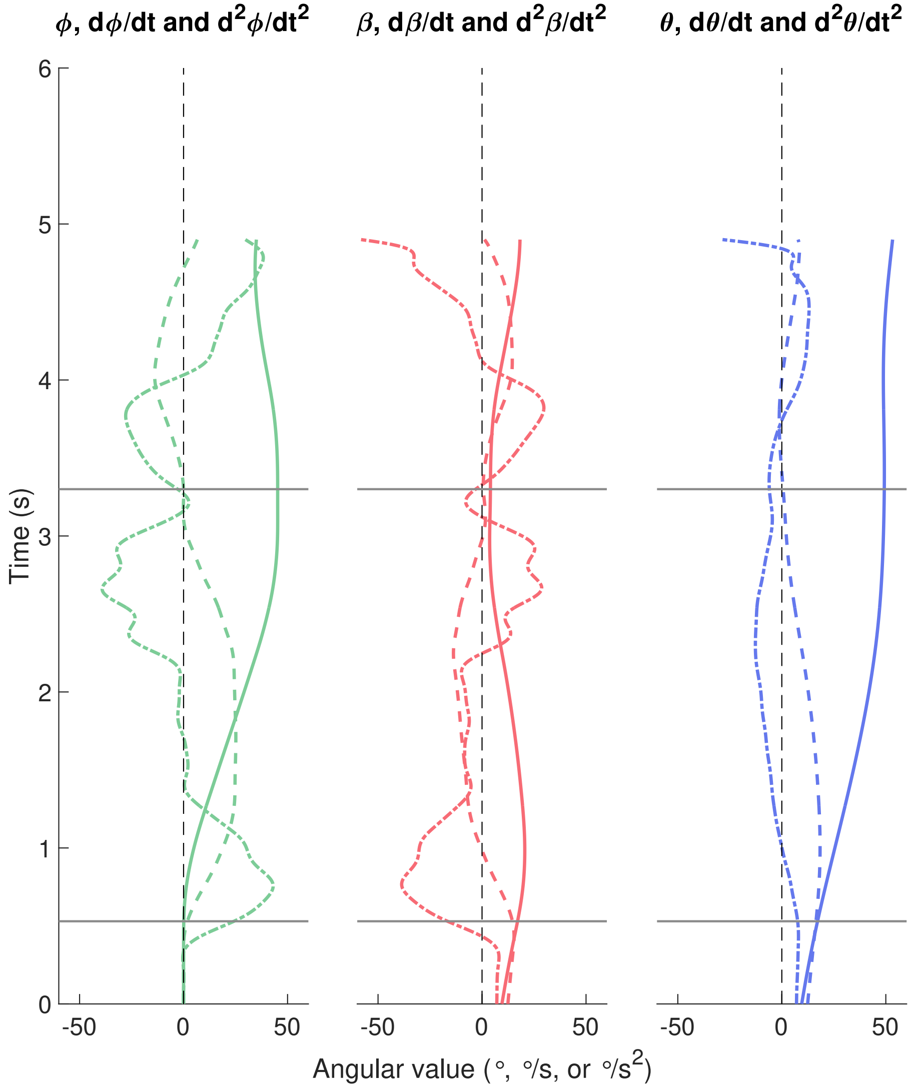

P13/B6
S10/R40-IN

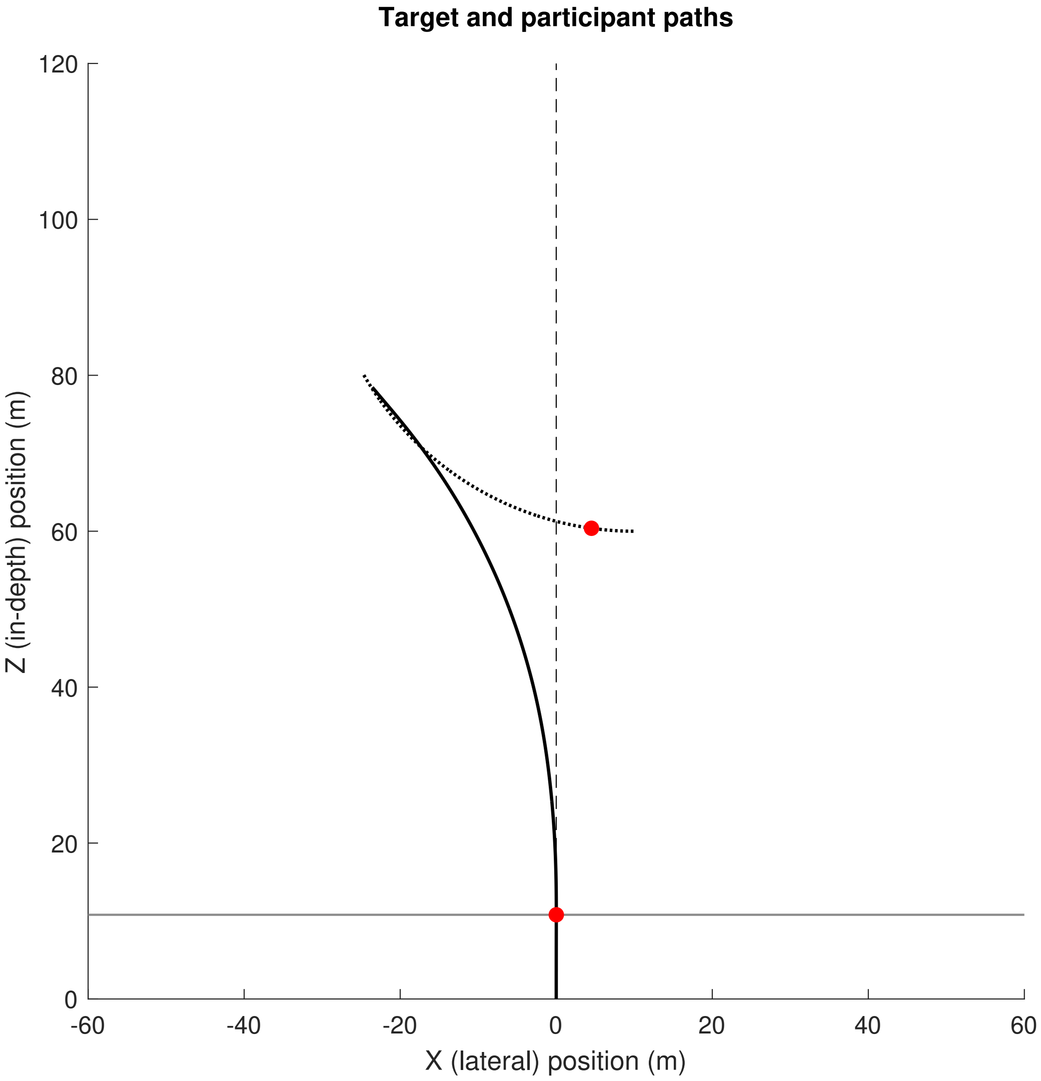

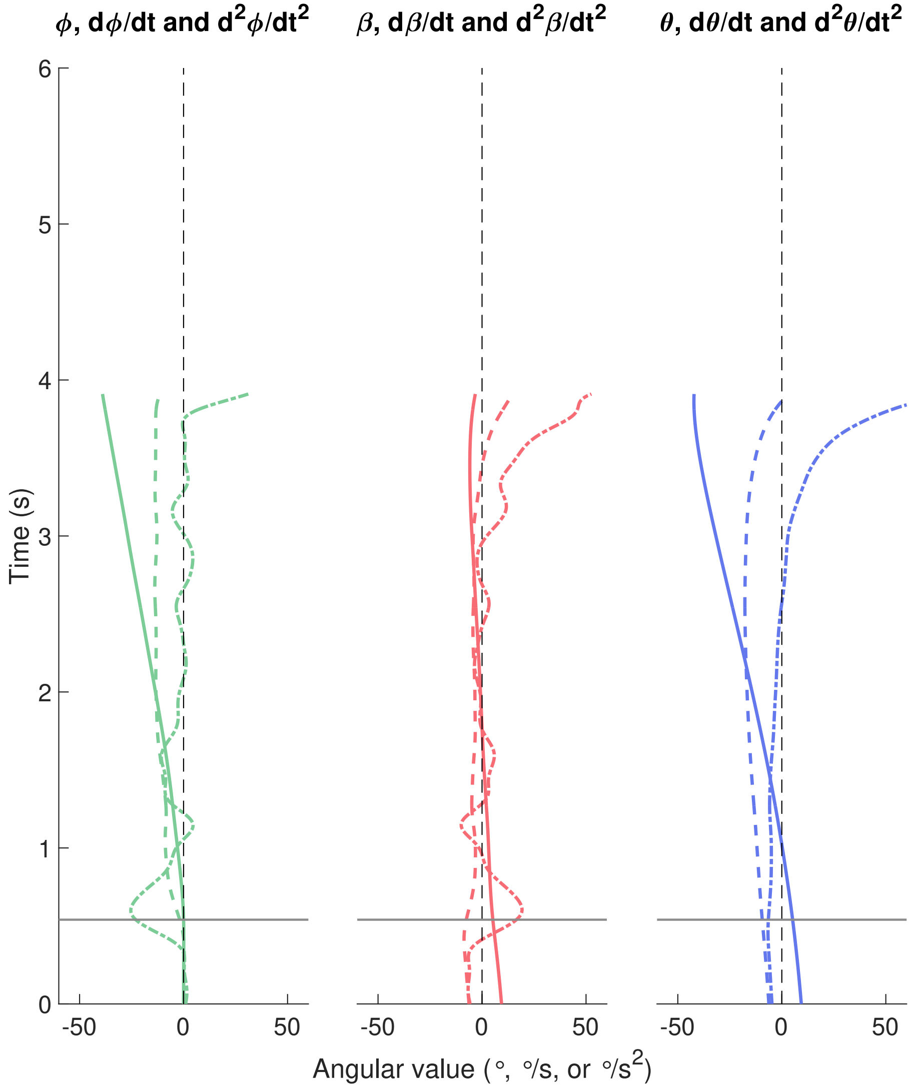

P13/B6
S20/R20-OUT

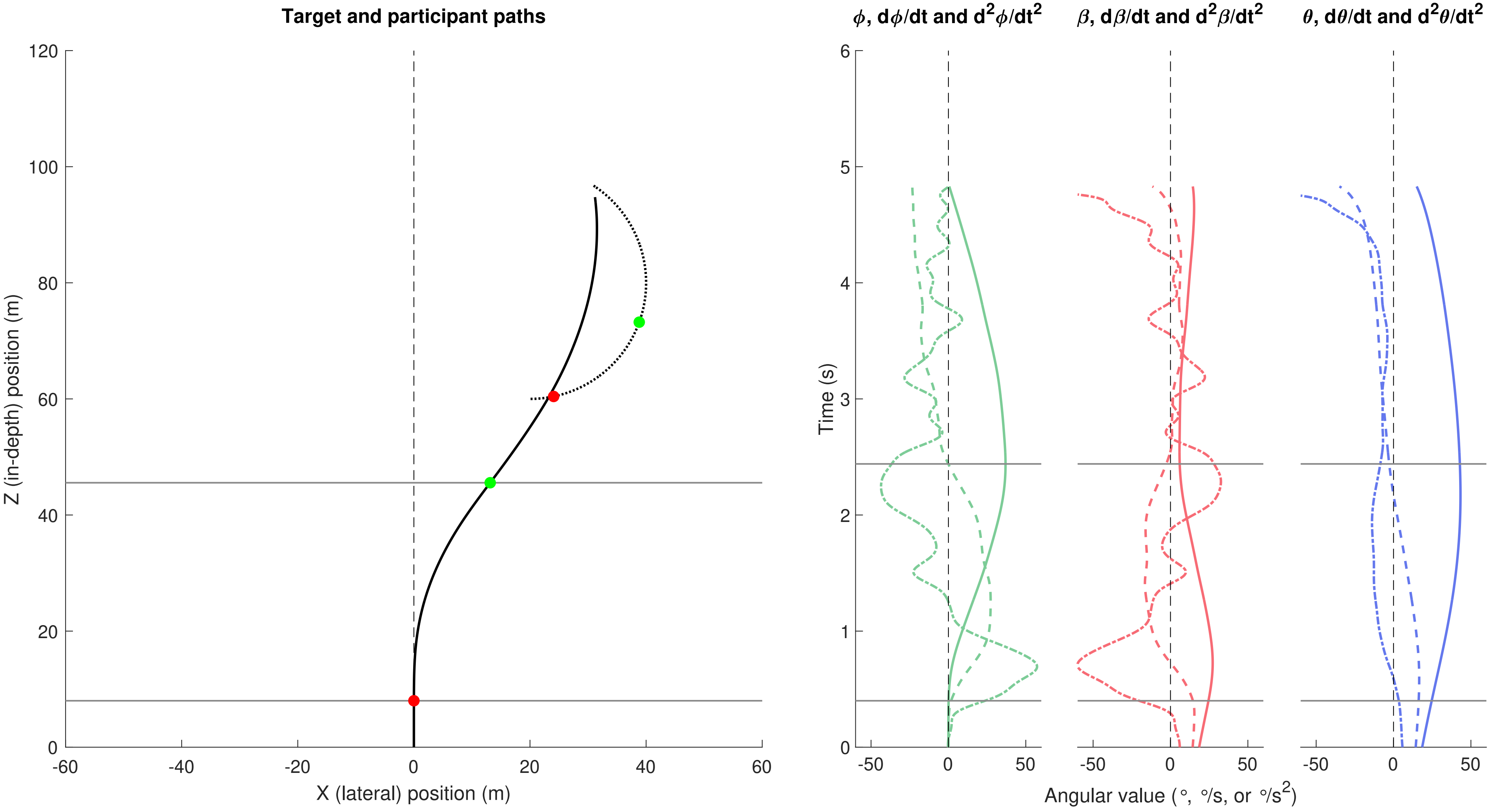

P13/B6
S20/R20-IN

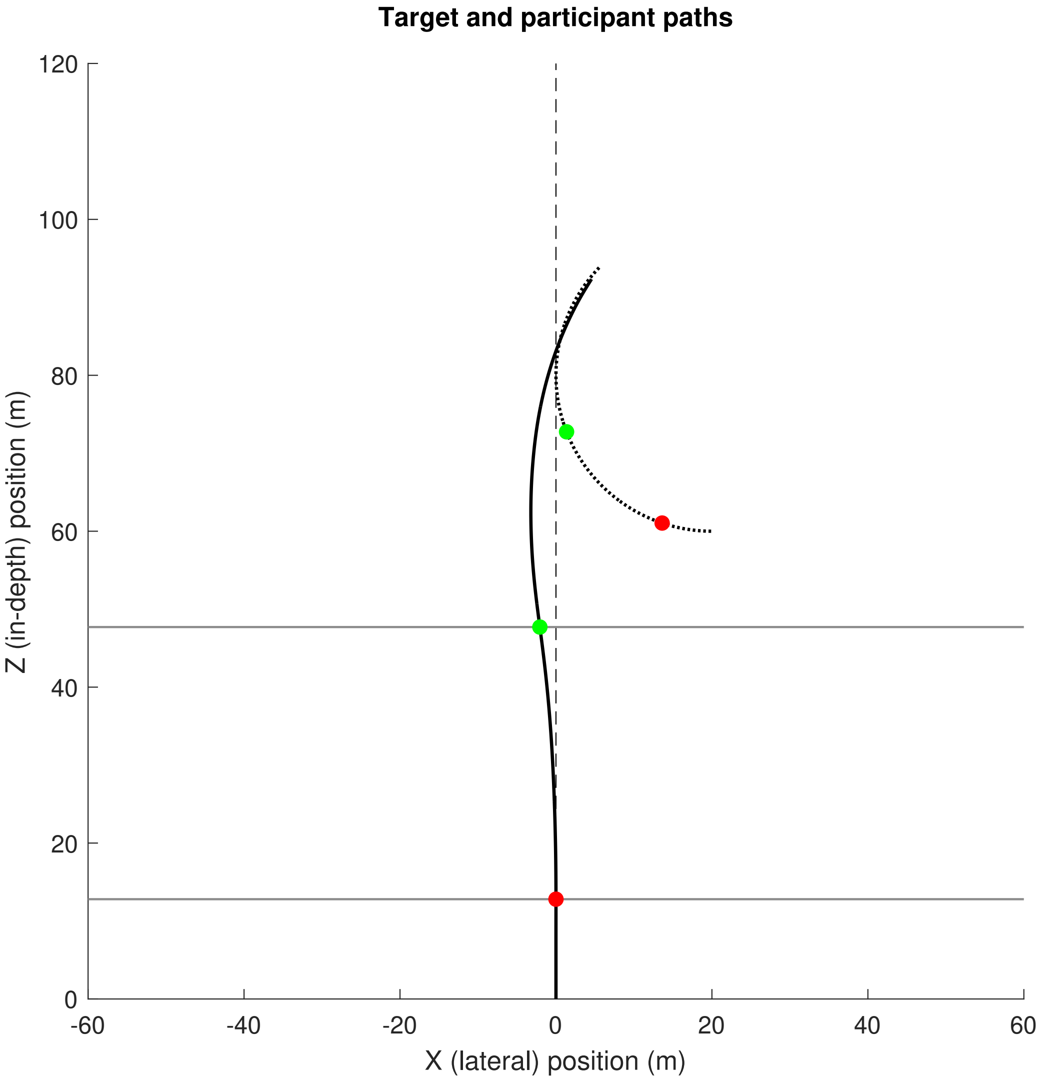

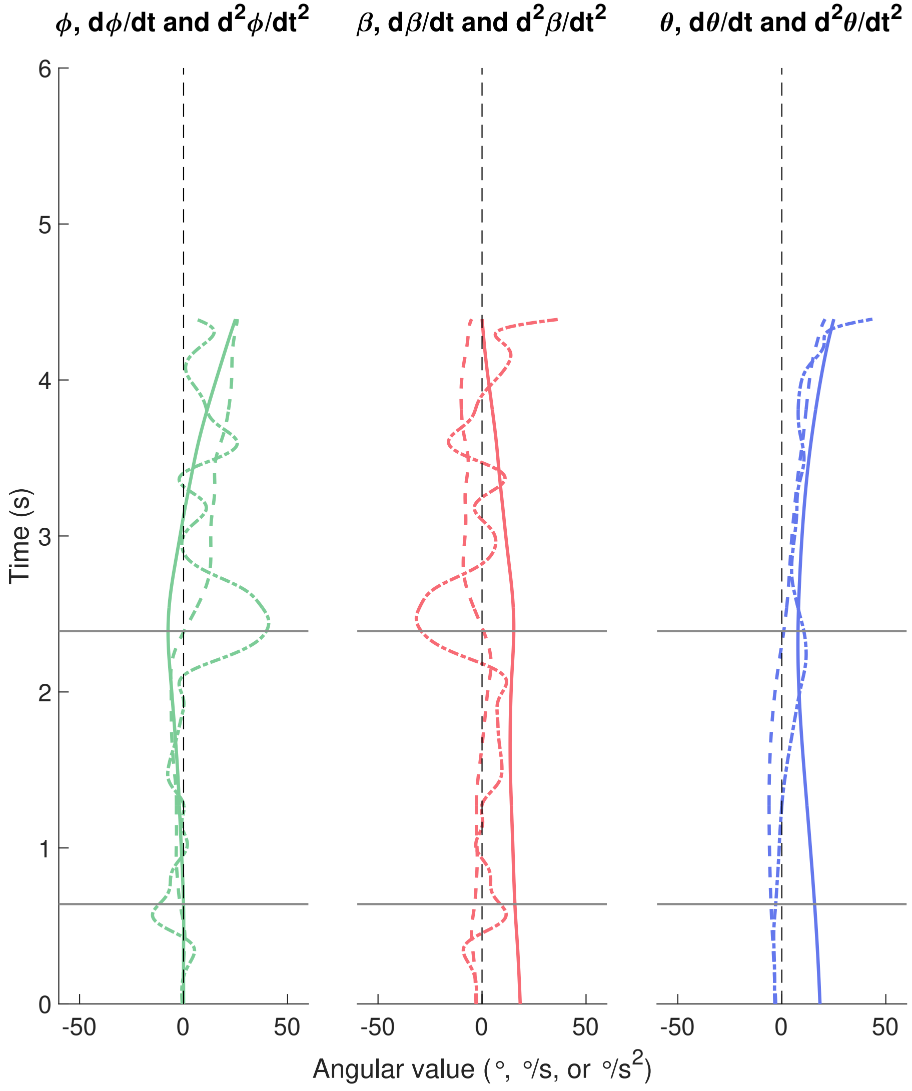

P13/B6
S20/R40-OUT

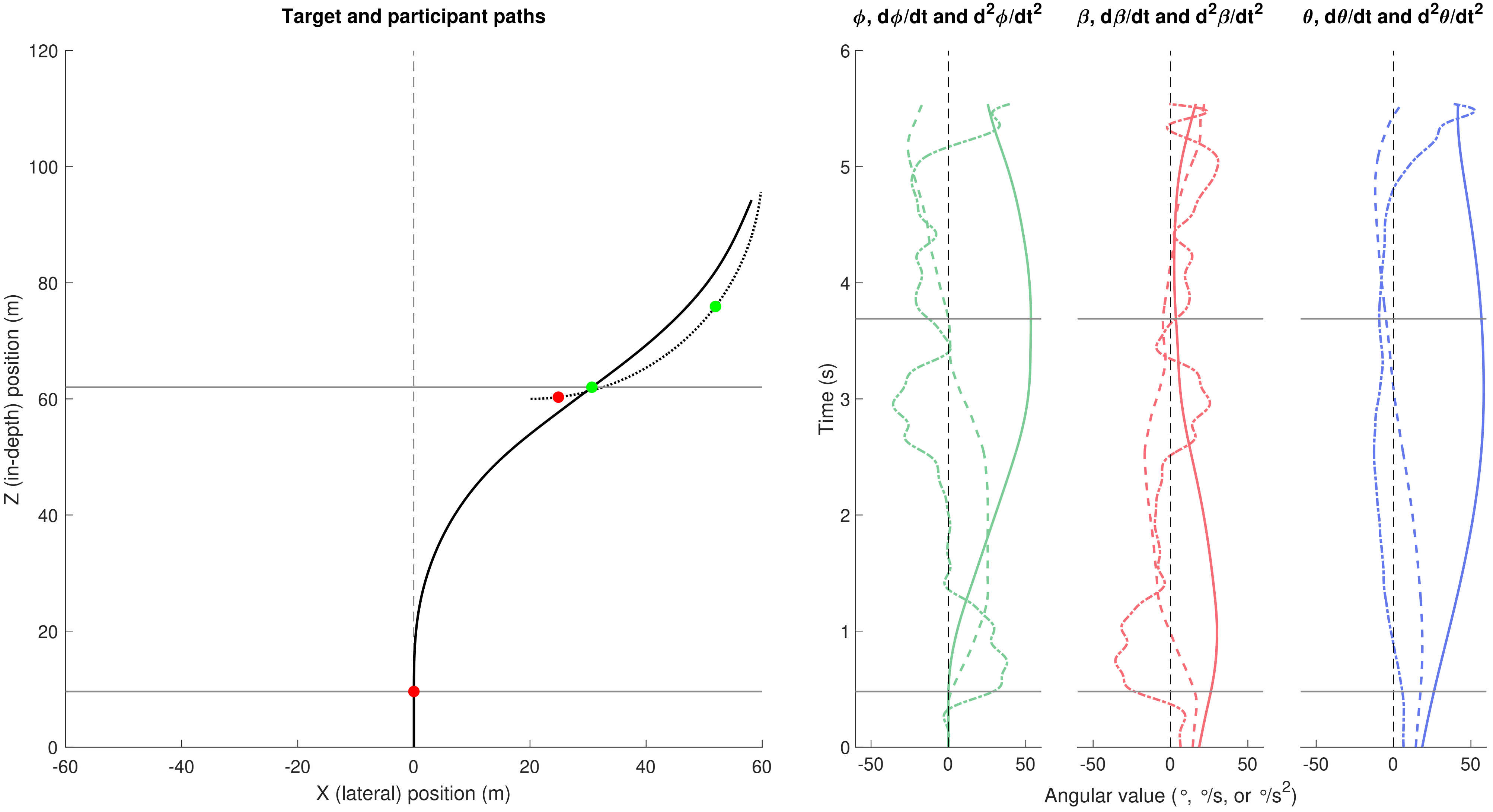

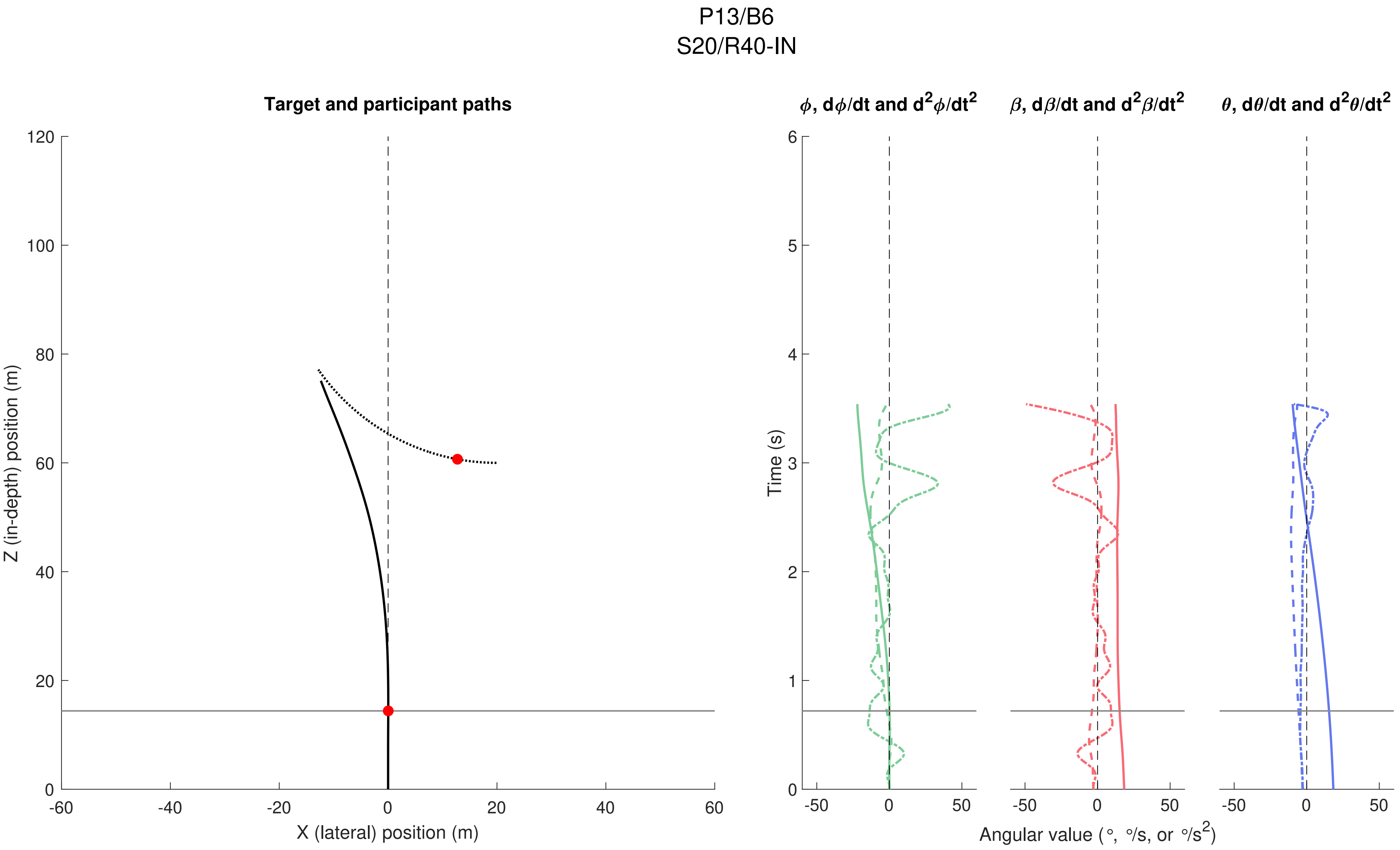
P13/B6
S20/R40-IN
Target and participant paths
Z (in-depth) position (m)
X (lateral) position (m)
120
100
80
60
40
20
0
-60
-40
-20
0
20
40
60
φ, dφ/dt and d²φ/dt²
β, dβ/dt and d²β/dt²
θ, dθ/dt and d²θ/dt²
Time (s)
6
5
4
3
2
1
0
-50
0
50
Angular value (°, °/s, or °/s²)
